# Boron-mediated modular assembly of tetrasubstituted alkenes

**DOI:** 10.1038/s41586-025-09209-2

**Published:** 2025-07-02

**Authors:** Liang Wei, Mihai V. Popescu, Adam Noble, Robert S. Paton, Varinder K. Aggarwal

**Affiliations:** 1 School of Chemistry, University of Bristol, Bristol, UK; 2 Department of Chemistry, Colorado State University, Fort Collins, CO USA

**Keywords:** Synthetic chemistry methodology, Natural product synthesis

## Abstract

Alkenes are a central part of organic chemistry^1–3^. However, although most alkenes are easy to prepare, the controlled synthesis of tetrasubstituted alkenes, those with four groups around the central C=C bond, remains challenging^1–5^. Here we report the boron-mediated assembly of tetrasubstituted alkenes with complete control of the double-bond geometry. The migrating group and electrophile add syn across the alkyne. Mild oxidation leads to intermediate borinic esters^6^, which can be isolated and purified or reacted directly in a range of transformations, including cross-couplings and homologation reactions. In particular, subjecting the intermediate borinic esters to Zweifel^7,8^ olefination conditions can give either retention or inversion of the double-bond geometry, depending on whether base is present or not. Different positional and stereoisomers of the tetrasubstituted alkenes can be easily accessed, highlighting the breadth and versatility of the method. This was showcased through its successful application to the rapid synthesis of drug molecules and natural products with high yield and stereocontrol. Not only does this method provide efficient access to the long-standing challenge of the stereocontrolled synthesis of tetrasubstituted alkenes but it also introduces new concepts related to the intervention of non-classical borenium ions in the Zweifel olefination.

## Abstract (web summary)

A method based on boron-mediated assembly is described for the synthesis of tetrasubstituted alkenes, molecules with four substituents around the central C=C bond, with complete control over the double-bond geometry.

## Main

**Fig. 1:**
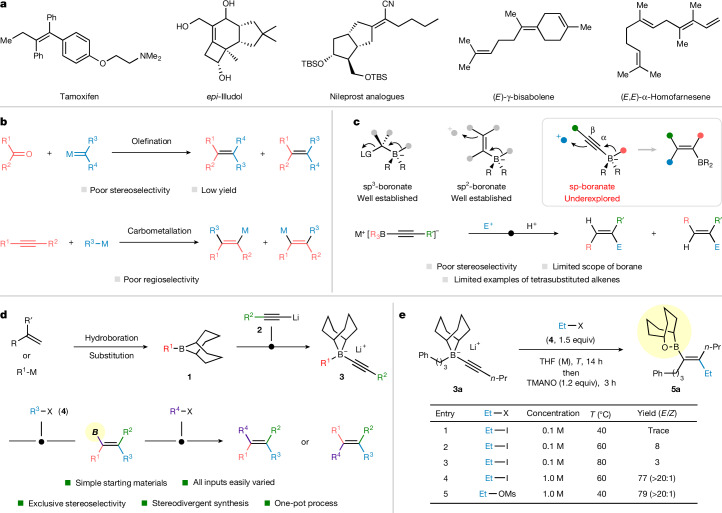
Synthesis of tetrasubstituted alkenes and 1,2-migration of boronate complex. **a**, Representative examples of tetrasubstituted alkenes in natural products and pharmaceuticals. **b**, Traditional methods for making tetrasubstituted alkenes and some of the problems associated with them. **c**, Examples of different classes of 1,2-migration of boronate complexes from different hybridizations of the carbon substituent on boron, including examples of sp-hybridized boronate complexes. **d**, Design concept for tetrasubstituted alkene synthesis (this work): reaction of 9-BBN alkynyl boronate complexes with electrophiles (R^3^-X) followed by transformation of the boron into R^4^. **e**, Preliminary optimization for the reaction of **3a** and **4**. Yields determined by ^1^H NMR using 1,3,5-trimethoxybenzene as internal standard; *E*/*Z* ratio was determined by crude ^1^H NMR. THF, tetrahydrofuran; TMANO, trimethylamine *N*-oxide.

Tetrasubstituted alkenes are commonly encountered in natural products, medicinal and materials chemistry and chemical biology^1–3^ (Fig. 1a). However, they can be challenging to make with high regiocontrol and stereocontrol. Classic olefination methodologies that are well suited to disubstituted and trisubstituted alkenes often fail when applied to the synthesis of tetrasubstituted alkenes owing to unfavourable steric interactions^1–5^ (Fig. 1b). More reliable strategies include the carbometallation/electrophilic trapping of internal alkynes^9,10^ and transition-metal-catalysed cross-coupling of multisubstituted alkenyl (pseudo)halides^11^. However, the former method requires the use of electronically/sterically biased internal alkynes or directing groups to achieve high regioselectivity (Fig. 1b), whereas the latter introduces the extra challenge of preparing the multisubstituted alkenyl (pseudo)halides selectively. Thus, new strategies to construct tetrasubstituted alkenes that involve the modular assembly of simple starting materials would be highly attractive and would complement present olefination methodologies.

The 1,2-metallate shift (1,2-migration) of tetracoordinated boronate complexes, driven by the displacement of an α-leaving group, oxidation of an α-boryl radical or electrophilic activation of a C=C double bond, represents a fundamental reaction mode of boron chemistry^12,13^. This area has been extensively investigated in complexes involving C(sp^3^)–B and C(sp^2^)–B boronates with numerous applications of 1,2-migrations^13,14^. The application of C(sp)–B boronate complexes in related reactions is much rarer, but we recognized that electrophilic activation of such alkynyl boronate complexes would provide access to tetrasubstituted alkenylboron species, which could potentially be converted into a diverse range of tetrasubstituted alkenes through C–B bond functionalization^10^ (Fig. 1c). The limited applications of C(sp)–B boronate complexes are presumably because of their instability. When derived from a pinacol boronic ester, the C(sp)–B boronate complex easily fragments to form a metallated alkyne, which is trapped by an electrophile (α-trapping)^15,16^. This undesired α-trapping can be prevented by generating the C(sp)–B boronate complex from much more electrophilic triarylboranes or trialkylboranes, which increases the stability of the alkynyl boronate complex and allows electrophile trapping at the β-position with concomitant 1,2-migration. These types of reaction received considerable attention in the 1970s, with numerous examples of carbon-based and heteroatom-based electrophiles reported^17–19^. However, the limitations of this chemistry with respect to the poor availability of symmetric triarylboranes or trialkylboranes, the low stereoselectivity observed with several electrophiles and the difficulty in manipulating the unstable alkenylborane products (Fig. 1c) have meant that the application of this strategy to the synthesis of tetrasubstituted alkenes has remained underexplored^20–24^. A notable advance was reported by Ishida et al., who showed that alkynyl-9-BBN-boronate complexes react with aryl halides in the presence of a Pd catalyst to give either *E* or *Z* trisubstituted alkenylboranes, depending on the phosphine ligand used^21^. Notably, subjecting the alkenyl-9-BBN products to Soderquist’s trimethylamine-*N*-oxide-mediated oxidation conditions generated stable alkenylborinic esters^6^, which could then be engaged in Suzuki–Miyaura couplings. Although synthetically useful, these Pd-catalysed reactions lack generality because they are limited to aryl migrating groups and aryl electrophiles. Nonetheless, the use of alkynyl-9-BBN-boronate complexes is highly attractive because of the availability of the borane precursors, specifically through simple hydroborations of abundant alkenes with 9-BBN. Therefore, if we could combine the diversity of electrophilic activation of triarylborane-derived or trialkylborane-derived alkynyl boronate complexes with the broader access to 9-BBN boranes, this could provide a general strategy for the synthesis of trisubstituted alkenylborons, which could potentially be transformed into tetrasubstituted alkenes (Fig. 1d).

We report here the development of a practical and highly stereoselective protocol to construct trisubstituted alkenyl boron compounds by means of electrophile-induced 1,2-migration of alkynyl-9-BBN-boronate complexes and their subsequent elaboration into tetrasubstituted alkenes.

## Reaction design and optimization

We began our investigation with in situ-generated alkynyl boronate complex **3a** derived from 3-phenylpropyl 9-BBN (formed in situ from hydroboration of allylbenzene with 9-*H*-BBN (9-borabicyclo[3.3.1]nonane)) and 1-pentynyllithium (Fig. 1e; see Supplementary Information for more details). We selected a relatively unreactive electrophile, ethyl iodide, reasoning that, if we could succeed with this substrate, then many other electrophiles could potentially be used^25^. Because the alkenylborane formed on 1,2-migration was expected to be sensitive to air and moisture, we decided to add a mild oxidant, trimethylamine *N*-oxide, to convert it to the more stable borinic ester **5** (ref. ^6^). Unfortunately, the reaction gave essentially no 1,2-migration/alkylation product (Fig. 1e, entry 1), but by increasing the reaction temperature, the product **5** was detected (entries 2 and 3). In consideration of other parameters that could accelerate the reaction, we were pleased to find that increasing the concentration tenfold markedly improved the reaction to give **5a** in 77% yield as a single isomer (entry 4). The more concentrated conditions also worked well for ethyl *p*-toluenesulfonate, even at lower temperatures (entry 5). Success with one of the least reactive electrophiles was highly encouraging and, as shown below, heralded a very broad electrophile scope. The very high selectivity observed, in favour of syn addition rather than anti addition, was also noteworthy. Although it is possible to invoke a steric model to account for the selectivity in which the bulky bicyclo[3.3.1]nonane ligand blocks the electrophile from approaching the top face of the alkyne (Fig. 1e), the exact origins of the high selectivity observed are at present being investigated.

**Fig. 2:**
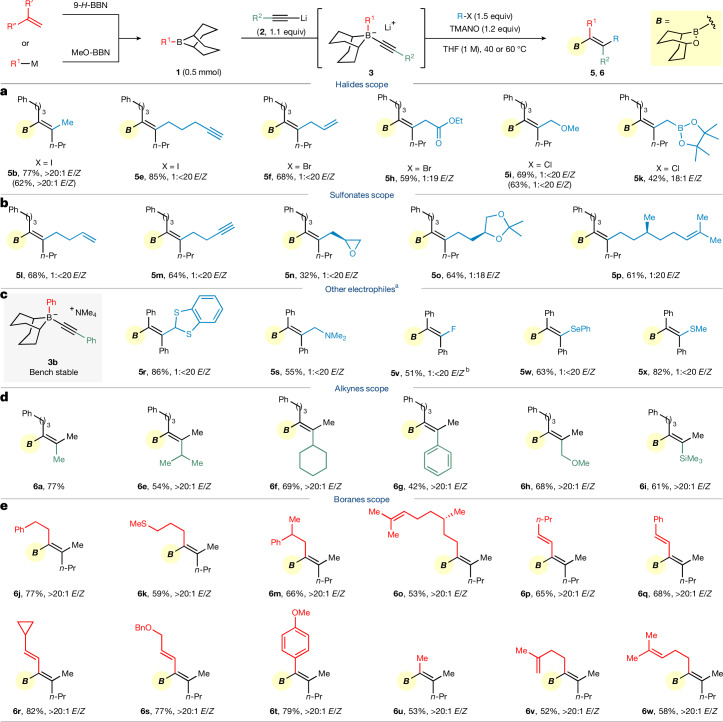
Selected scope electrophiles, alkynes and boranes. **a**–**e**, Conditions: the reactions were conducted with 0.5 mmol of **3** (formed from 0.5 mmol of borane and 0.55 mmol of alkynyllithium; for **6a**–**s**, boranes were formed in situ from 0.5 mmol of alkene/0.5 mmol of 9-*H*-BBN; for **6t**–**w**, boranes were prepared from MeO-BBN and purified by distillation before use) and electrophile (1.5 equiv) in THF (0.5 ml) at 40 °C (for sulfonates) or 60 °C (for halides) for 4–24 h, then 0.6 mmol of TMANO in 1 ml of DCM was added and stirred for 3 h. ^a^Modified conditions with 0.2 mmol of **3b**, 0.24 mmol of electrophile, EtOAc (0.1 M) and 40 °C were used. ^b^THF (0.1 M) was used.

**Extended Data Fig. 1:**
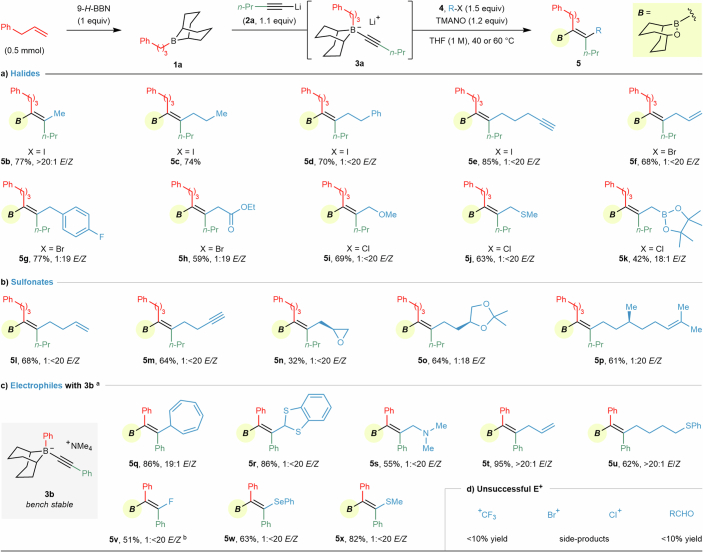
Scope and limitations of electrophiles. **a**–**d**, Conditions: the reactions were conducted with 0.5 mmol of **3a** (formed from 0.5 mmol of allylbenzene, 0.5 mmol of 9-*H*-BBN and 0.55 mmol of 1-pentynyllithium) and electrophile (1.5 equiv) in THF (0.5 ml) at 40 °C (for sulfonates) or 60 °C (for halides) for 4–24 h, then 0.6 mmol of TMANO in 1 ml of DCM was added and stirred for 3 h. ^a^Modified conditions with 0.2 mmol of **3b**, 0.24 mmol of electrophile, EtOAc (0.1 M) and 40 °C were used. ^b^THF (0.1 M) was used.

**Extended Data Fig. 2:**
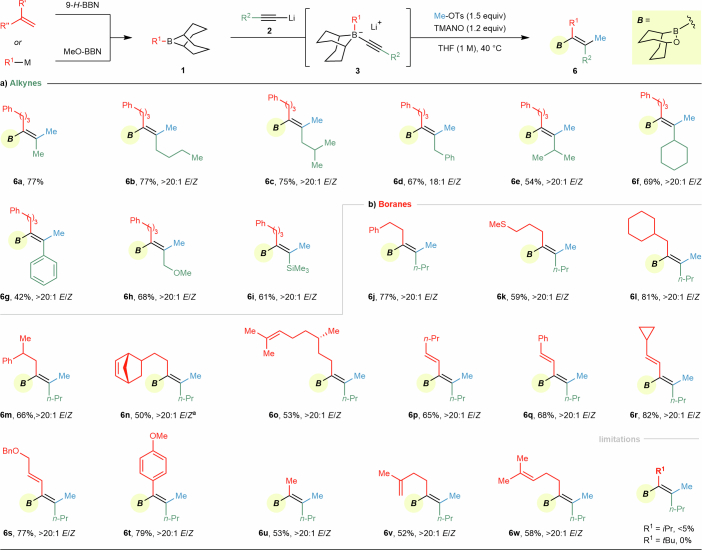
Scope and limitations of alkynes and boranes. **a**,**b**, Conditions: the reactions were conducted with 0.5 mmol of **3** (formed from 0.5 mmol of borane and 0.55 mmol of alkynyllithium; for **6a**–**s**, boranes were formed in situ from 0.5 mmol of alkene/0.5 mmol of 9-*H*-BBN; for **6t**–**w**, boranes were prepared from MeO-BBN and purified by distillation before use) and electrophile (1.5 equiv) in THF (0.5 ml) at 40 °C for 4–24 h, then 0.6 mmol of TMANO in 1 ml of DCM was added and stirred for 3 h. ^a^A mixture of endo/exo-**5n** was obtained and 5-vinyl-2-norborene was used as a mixture of endo/exo isomers.

The scope of the reaction was explored next (Fig. 2; see Extended Data Figs. 1 and 2 for full examples). With boronate complex **3a** as the model nucleophile, a variety of simple and more complex alkyl halides (Fig. 2a and Extended Data Fig. 1) and *p*-toluenesulfonates (Fig. 2b) were explored and, as expected, worked well, affording borinic esters **5b**–**p** in moderate to high yields and usually with >20:1 ratio of alkene isomers in favour of the syn addition product. Reaction with chloromethyl boronic acid pinacol ester gave **5k** in good nuclear magnetic resonance (NMR) yield but low isolated yield owing to partial decomposition of the allylic boronic ester during chromatographic purification. The reaction of **3a** with methyl iodide and chloromethyl methyl ether was scaled up to the 10 mmol scale, giving **5b** and **5i** in similar yield and selectivity.

Having shown that alkyl halides and sulfonates were competent electrophiles, we decided to test a much broader set of diverse electrophiles (Fig. 2c and Extended Data Fig. 1; also see Supplementary Table 2 for more details). In these reactions, the isolable, bench-stable alkynyl boronate complex **3b** with tetramethyl ammonium as the counter cation was used to simplify the protocol. Tropylium tetrafluoroborate, 1,3-benzodithiolylium tetrafluoroborate and Eschenmoser’s salt were well-suited reaction partners in this transformation, giving products **5q**–**s**, respectively. Alkyl sulfonium salts were also effective electrophiles, including allyl di(*p*-tolyl)sulfonium and 1-phenyl tetrahydrothiophenium, which gave the allylation and ring-opening product **5t** and **5u**, respectively, in good yield and with complete stereoselectivity. As well as these carbon-based electrophiles, heteroatom-based electrophiles were also tested. Electrophilic fluorination-induced 1,2-migration with Selectfluor gave fluorinated alkenyl borinic ester **5v** in moderate yield. The stereoselective synthesis of the fully substituted fluoroalkene is noteworthy, as fluoroalkenes have been used as bioisosteres of amides^26^. The reaction with phenylselenyl chloride and DMTSF (dimethyl(methylthio)sulfonium tetrafluoroborate) gave the corresponding alkenyl selenide **5w** and sulfide **5x**, respectively. However, not all electrophiles worked well; some were low yielding (such as Togni’s reagent, chloroformates) and others gave several products (NBS, NCS and aldehydes) (Extended Data Fig. 1d).

We then investigated the scope of boronate complex precursors in the 1,2-migration using MeOTs as the electrophile, as the methyl group (Me) is one of the most common alkene substituents found in natural products and drug candidates^27^ (Fig. 2d,e and Extended Data Fig. 2). Primary (**6a**–**d**) and secondary alkyl-substituted terminal alkynes (**6e**,**f**) as well as phenylacetylene (**6g**) worked effectively. Methyl propargyl ether (**6h**) and trimethylsilyl acetylene (**6i**) were also viable, affording allylic alcohol and alkenyl silane derivatives in good yield and high selectivity. An attractive feature of our approach is the breadth of organoboron compounds that can be used, owing to the availability of a vast range of 9-BBN-based boranes, which is in contrast to triarylboranes and trialkylboranes. A diverse set of terminal alkenes were hydroborated using 9-*H*-BBN and used directly in the subsequent reaction, giving alkenyl borinic esters **6j**–**m**. The terminal alkene on 5-vinyl-2-norborene was hydroborated exclusively and gave **6n** in 50% yield. Hydroboration of (−)-citronellene gave an enantiomerically pure borane, which furnished chiral alkene **6o** in 53% yield. Furthermore, alkenyl boranes, accessed through the hydroboration of alkynes, also proved to be compatible with our methodology, and the desired 1,3-dienes **6p**–**s** were obtained in high yield with excellent stereoselectivity. These substrates are particularly interesting, as the electrophile can react with either the activated alkene or the activated alkyne, leading to distinct products. Even though alkenes are usually more reactive towards electrophiles than alkynes^28^, we found that, in the alkenyl–alkynyl boronate complex, the alkyne reacted exclusively, giving the diene product. As well as preparing the boranes by hydroboration reactions with 9-*H*-BBN, aryl/alkyl-9-BBNs can also be made by substitution of *B*-methoxy BBN with organometallic reagents. Aryl-containing (**6t**), methyl-containing (**6u**) and alkene-containing (**6v**,**w**) boranes all reacted smoothly under our standard conditions. Notably, the four boranes used in the preparation of **6t**–**w** are inaccessible by hydroboration, therefore the combined use of the two complementary methods (hydroboration and substitution) provides an even broader scope of boranes that can be used. Attempts to use the more substituted *i*Pr-9-BBN-boranes and *t*Bu-9-BBN-boranes were unsuccessful, resulting in migration of the bicyclo[3.3.1]nonane ring instead of the borane substituent (see Supplementary Information for details).

## Synthetic transformations and applications

To achieve our initial goal, we wanted to convert the trisubstituted alkenyl borinic esters to tetrasubstituted alkenes, but in comparison with their borane and boronic ester analogues, transforming borinic esters to other functionalities have rarely been investigated. We therefore set about investigating different classes of reactions of borinic esters.

### Suzuki–Miyaura cross-coupling and Zweifel olefination

**Fig. 3:**
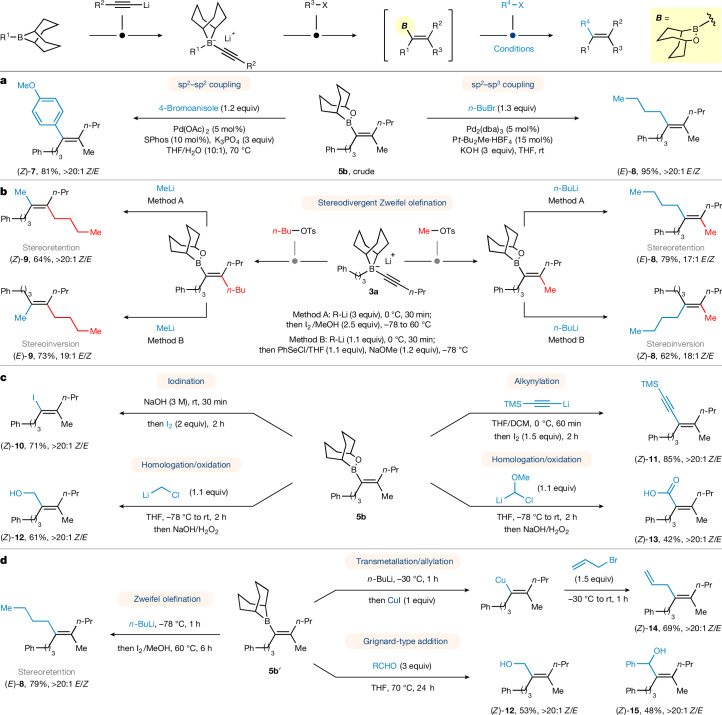
Synthetic transformation of borinic ester intermediates to diverse tetrasubstituted alkenes. **a**, sp^2^–sp^2^ and sp^2^–sp^3^ cross-couplings of borinic ester and organohalides using palladium catalysis. **b**, Zweifel olefination showing how different positional isomers can be obtained by using MeLi and *n-*BuOTs in place of *n-*BuLi and MeOTs and different geometric isomers can be obtained by using different reaction conditions. **c**, Further transformations of borinic esters, including Matteson homologations, iodination and alkynylation. **d**, Direct reactions of borane intermediates, including Zweifel olefination, transmetallation to copper followed by electrophile trapping and direct Grignard-type additions to aldehydes.

We first investigated Pd-catalysed sp^2^–sp^2^ cross-coupling^29,30^ of crude alkenyl borinic ester **5b** with 4-bromoanisole and were pleased to find that the borinic ester coupled smoothly, giving aryl trialkylethylene derivative^31^ **7** in 81% yield (Fig. 3a, left). The sp^2^–sp^3^ cross-coupling with alkyl halides is much more challenging because the alkylpalladium species can easily undergo undesired β-elimination pathway. Nevertheless, Kirchhoff et al.^32^ and Nishihara et al.^24^ showed that C(sp^2^)-boronic acids and boronic esters could be coupled with primary alkyl halides using Pd_2_(dba)_3_/*t*Bu_2_MeP·HBF_4_ as the catalyst. Using their conditions with our alkenyl borinic esters, the desired coupling reaction proceeded efficiently, giving tetraalkyl-substituted alkene (*E*)-**8** in nearly quantitative yield^33^ (Fig. 3a, right).

The Suzuki-type coupling occurs with retention of double-bond geometry and we were interested to see whether the alternative stereoisomer could also be accessed from the same building block using the Zweifel olefination, which is known to occur with stereoinversion^7,8^ (Fig. 3b). Notably, the Zweifel olefination reaction with *n-*BuLi and I_2_ also gave the stereoretention product (*E*)-**8** in 79% yield with 17:1 *E*/*Z* (method A). However, the addition of LiOMe to the same reaction mixture resulted in formation of the opposite isomer (*Z*)-**9** in moderate yield with 1:6 *E*/*Z* selectivity, restoring the normal invertive pathway. This is presumably because of favoured anti elimination of the β-iodoborinic ester intermediate on coordination of the base to boron; however, the 1:6 selectivity showed that competing syn elimination still occurred to a small extent. We reasoned that syn elimination could be disfavoured if iodide was replaced with a poorer leaving group. Hence we used PhSeCl in place of I_2_ and were pleased to observe much higher *Z* selectivity (18:1 *Z*/*E*, method B)^34^. This showed that stereoselectivity could be successfully switched and either *E* or *Z* alkenes could be obtained with high selectivity from the same substrate simply by altering the reaction conditions and reagents used. The mechanism for the retentive Zweifel pathway turned out to be more complex and is discussed later. To further demonstrate the versatility of this method, simply by swapping the nucleophiles and electrophiles (using MeLi and BuOTs in place of *n*-BuLi and MeOTs), we accessed both stereoisomers of tetrasubstituted alkene **9**, the regioisomer of **8**, with essentially complete stereocontrol.

### Iodination

Tetrasubstituted alkenyl monohalides are important chemical linchpins and have been widely used in transition-metal-catalysed cross-coupling reactions, enabling the synthesis of many other valuable tetrasubstituted olefins^35^. The tetrasubstituted alkenyl iodide (*Z*)-**10** was readily obtained by treatment of tetrasubstituted alkenyl borinic ester **5b** with I_2_ and base in 71% yield and complete retention of stereochemistry (Fig. 3c).

### Zweifel-type coupling of borinic esters with alkynes

Stereoretentive Zweifel-type sp^2^–sp coupling was also developed with alkynyllithium and I_2_ producing enyne (*Z*)-**11** in high yield and >20:1 *Z*/*E* (ref. ^36^) (Fig. 3c).

### Matteson-type homologation reactions of borinic esters

By using chloromethyl lithium and methoxychloromethyl lithium^37^, the substituted allylic alcohol (*Z*)-**12** and alkenyl carboxylic acid (*Z*)-**13** were obtained through Matteson homologation/oxidation sequences in 61% and 42% yield, respectively (Fig. 3c).

### Reactions of 9-BBN alkenyl boranes

The direct use of 9-BBN trisubstituted alkenyl borane **5b′** as a synthetic building block was also investigated and found to offer further opportunities in synthesis. The base-free conditions that we developed for the stereoretentive Zweifel olefination of borinic esters (method A) could be extended to the analogous borane substrate, affording tetrasubstituted alkene (*E*)-**8** in even higher yield and higher stereoselectivity (retention again). Unfortunately, the stereoinvertive protocol using base (method B) was not compatible with alkenyl borane **5b′**, as it also gave the stereoretention product but in low yield (28%). The tetracoordinated boronate complex derived from *n-*BuLi and alkenyl borane **5b′** could be transmetallated with CuI and trapped by electrophiles such as allyl bromide, delivering (*Z*)-**14** in 69% yield^38^ (Fig. 3d). Disubstituted alkenyl boranes have been shown to react directly with aldehydes in Grignard-type additions^39^. Despite the added steric hindrance of the fully substituted 9-BBN alkenyl boranes, we found that they reacted smoothly with aldehydes without any other promoters to produce tetrasubstituted allylic alcohols (*Z*)-**12** and (*Z*)-**15** in good yields (Fig. 3d).

**Fig. 4:**
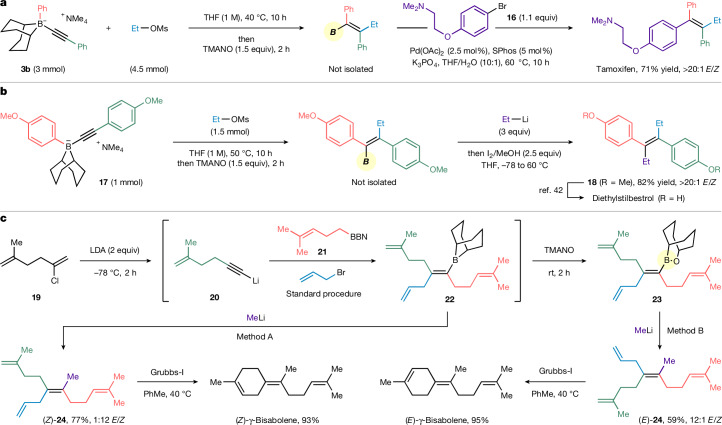
Synthetic applications. **a**, Two-step, one-pot synthesis of tamoxifen through 1,2-migration followed by palladium-catalysed cross-coupling. **b**, Three-step formal synthesis of diethylstilbestrol through 1,2-migration, Zweifel olefination and demethylation. **c**, Synthesis of (*E*)-γ-bisabolene and (*Z*)-γ-bisabolene from diene **19** using a stereodivergent Zweifel olefination and ring-closing metathesis as the key steps.

Finally, we sought to demonstrate the applicability of this methodology to the rapid synthesis of natural products and drug molecules (Fig. 4). Tamoxifen^40^, an anticancer drug, is often used as a target to showcase tetrasubstituted alkene synthesis methodology. Starting from alkynyl boronate complex **3b** and ethyl mesylate, the 1,2-migration/ethylation and cross-coupling with aryl halide **16** delivered tamoxifen in one pot in 71% overall yield and with complete stereocontrol (Fig. 4a). Furthermore, the reaction of boronate complex **17** with EtOMs, followed by stereoretentive Zweifel olefination with EtLi, gave (*E*)-**18**, which is the key precursor to diethylstilbestrol, a synthetic non-steroidal oestrogen medication^41,42^ (Fig. 4b).

As well as drug molecules, the natural products (*E*)-γ-bisabolene and (*Z*)-γ-bisabolene could also be synthesized efficiently with our methodology (Fig. 4c). Chlorodiene **19** (ref. ^43^) was treated with LDA to give alkynyl-Li **20**, which was reacted with borane **21**. Addition of allyl bromide gave alkenyl borane **22**, which was treated with MeLi and I_2_ to promote a stereoretentive Zweifel olefination (method A), giving the tetrasubstituted alkene (*Z*)-**24** in 77% yield with 12:1 *Z*/*E* selectivity. Alternatively, oxidation of **22** to alkenyl borinic ester **23**, followed by our stereoinvertive Zweifel olefination conditions (method B) favoured the *E* isomers of **24** (12:1, *E*/*Z*). Subjecting the two stereoisomers of **24** to ring-closing metathesis using Grubbs-I catalyst gave (*E*)-γ-bisabolene and (*Z*)-γ-bisabolene in almost quantitative yield^44^. The stereodivergent synthesis of bisabolene in just three or four steps (longest linear sequence) from the same set of starting materials highlights the versatility, efficiency and generality of our methodology.

## Computational analysis of stereodivergent Zweifel olefination

**Fig. 5:**
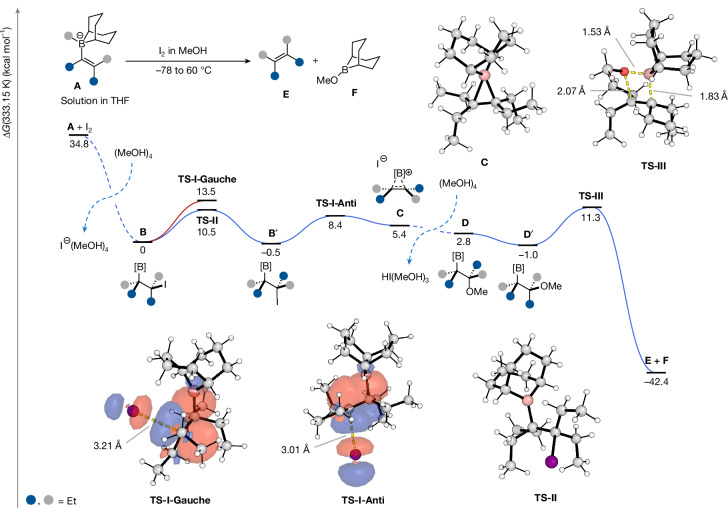
Computational studies. Potential-energy surfaces of Zweifel olefinations using a model tetraethyl-substituted system under neutral conditions, which occurs with overall retention of double-bond geometry. The selectivity is determined by the difference in TS energies between elimination (TS-I-Gauche) and C–C bond rotation (TS-II-rotation) from the gauche conformation A. The σ(C–B) and σ*(C–I) for TS-I-Gauche and TS-I-Anti obtained from natural bond orbital calculations are highlighted. Density functional theory calculations were performed using the Gaussian 16 package^51^ and ORCA 6 (ref. ^52^) at the PBE0-DH-D3(BJ)/def2-TZVPD,SMD(THF)//M06-2X-D3/def2-SVPD;def2-TZVPD[I],SMD(THF) level of theory.

The origin of the stereodivergent Zweifel olefination was intriguing. The Zweifel olefination is normally a stereoinvertive process, occurring through anti elimination of the β-iodoboranes/borinic esters^45^. Stereoretention was unexpected and is rare, and had been assumed to occur through a syn elimination of the same β-iodoboranes/borinic esters. To shed more light on this explanation, we carried out computational analysis using density functional theory and, in the process, discovered a new mechanism to account for the stereoretentive pathway^46^ (Fig. 5).

Under neutral conditions, retention of double-bond geometry was observed for the borane (Fig. 2b, method A), which had previously been rationalized to occur by means of a syn elimination pathway, through extrusion of an iodoboron species^45,47^. However, all attempts to model this pathway were unsuccessful and no transition structure (TS) could be found, presumably because of the very weak B–I bond (computationally, the B–I bond dissociation enthalpy is 33.5 kcal mol^−1^ weaker than the corresponding B–O bond), and so alternative pathways were investigated. Instead, we found that, in MeOH, β-iodoboranes such as **B′**, could undergo solvolysis through an unprecedented, non-classical borenium ion^48^ **C**, which could be trapped by MeOH with overall retention of configuration to give **D**. Heterolysis of the anti-orientation **B′** was found to be easily surmountable, with an activation energy barrier of only 8.4 kcal mol^−1^ through TS-I-Anti. Adduct **D** then undergoes bond rotation followed by syn elimination through a bora-Wittig-type process^49^ through TS-III, resulting in overall retention of configuration. The gauche conformer of β-iodoborane **A** can also undergo solvolysis but with a higher activation energy barrier of 13.5 kcal mol^−1^ through TS-I-Gauche. Further natural bond orbital^50^ analysis of the TS-I competing TSs revealed a strong donation from the C–B σ bonding orbital to the C–I σ* antibonding orbital of 43.7 kcal mol^−1^ within the anti conformation, assisting the formation of the three-centre two-electron (3c2e) bond in the emerging non-classical carbocation **C**. This interaction was found to be decreased by 9.3 kcal mol^−1^ in the gauche analogue, as evidenced by the poorer orbital overlap. Subsequent trapping by MeOH and syn elimination would furnish the opposite alkene isomer. Overall, for the model system studied, the selectivity is determined by the difference in TS energies between heterolysis (TS-I-Gauche) and C–C bond rotation (TS-II-rotation) from the gauche conformation **A**; however, these may change as a function of the substituent identity (see Supplementary Information for profile comparison with the tetramethyl analogue). This new mechanism could apply to other Zweifel olefinations, in which competing retention of configuration is observed^43,45,47^ (for computational studies on the stereoinvertive Zweifel olefination, see Supplementary Information).

## Online content

Any methods, additional references, Nature Portfolio reporting summaries, source data, extended data, supplementary information, acknowledgements, peer review information; details of author contributions and competing interests; and statements of data and code availability are available at 10.1038/s41586-025-09209-2.

## Supplementary information

Supplementary Information Supplementary Materials and General information, Experimental Data, Computational Data and Supplementary References – see Contents for details.

## Data Availability

The data supporting the findings of this study are available in the paper and its Supplementary Information.
