# Supplementary material for: Boron-mediated modular assembly of tetrasubstituted alkenes
Source: Nature. 2025 Jul 2;643(8073):975–82. doi: 10.1038/s41586-025-09209-2 (PMC12286843; doi:10.1038/s41586-025-09209-2)
Supplement: Supplementary file 1 — Supplementary Materials and General information, Experimental Data, Computational Data and Supplementary References – see Contents for details. [file 41586_2025_9209_MOESM1_ESM.pdf]

nature portfolio

https://doi.org/10.1038/s41586-025-09209-2

Supplementary information

Boron-mediated modular assembly of tetrasubstituted alkenes

In the format provided by the authors and unedited

Supplementary Information

Stereocontrolled, Boron-Mediated, Modular Assembly of Tetrasubstituted Alkenes

Liang Wei, Mihai V. Popescu, Adam Noble, Robert S. Paton, Varinder K. Aggarwal[†*]

School of Chemistry, University of Bristol, Cantock's Close, Bristol BS8 1TS, UK.

**email: v.aggarwal@bristol.ac.uk*

TABLE OF CONTENTS

List of Supplementary Schemes, Figures and Tables..... Error! Bookmark not defined.

List of Characterised Products 3

1. MATERIALS AND GENERAL INFORMATION 5

2. EXPERIMENTAL DATA.. 7

2.1. Reaction Optimization 7

2.2. Summarized electrophiles in this reaction 8

2.3. General Procedures 8

2.3.1 General Procedure A for product 5a-p and 6a-s... 8

2.3.2 General Procedure B for product 5q-x.. 9

2.3.3 General Procedure C for product 6t-w 10

2.4. Reaction of Steric Hindered Boranes... 10

2.5. Comparison of Ph-9-BBN and Ph_3B as borane sources 12

2.6. Synthesis of Starting Materials.. 13

2.7. Determination of Stereochemistry of 5a and 5b... 13

2.8.1 *sp*^{2}-*sp*^{2} and *sp*^{2}-*sp*^{3} cross coupling of 5b[5,6] 16

2.8.2 Zweifel Olefination of Borinic Ester[7] 17

2.8.3 Iodination to (*Z*)-10.. 18

2.8.4 Alkynylation to (*Z*)-11 18

2.8.5 Matteson Homologation to (*Z*)-12 19

2.8.6 Carboxylation to (*Z*)-13.. 19

2.8.7 Zweifel Olefination of Borane 20

2.8.8 Transmetallation of Borane to Copper(I) and Subsequent Allylation[12]................ 20

2.8.9 Grignard-type Addition of Borane to Aldehyde[13]... 21

2.8.10 Synthesis of *cis*-Tamoxifen 21

2.8.11 Synthesis of diethylstilbestrol precursor 18... 22

2.8.12 Synthesis of (*E*)- and (*Z*)-γ-bisabolene.. 23

2.9. Characterization Data for Products. 26

2.10. References 61

2.11 NMR Spectra.. 62

3. COMPUTATIONAL DATA 131

3.1. Methods 131

3.2. Benchmark... 134

3.3. Computational results 136

3.4. Thermochemistry 144
3.5 XYZ Coordinates: 161
3.6 References 300

List of Characterised Products

(*E*)-10-(5-ethyl-1-phenyloct-4-en-4-yl)-9-oxa-10-borabicyclo[3.3.2]decane (5a) 26
(*E*)-10-(5-methyl-1-phenyloct-4-en-4-yl)-9-oxa-10-borabicyclo[3.3.2]decane (5b) 26
10-(1-phenyl-5-propyloct-4-en-4-yl)-9-oxa-10-borabicyclo[3.3.2]decane (5c) 27
(*Z*)-10-(5-phenethyl-1-phenyloct-4-en-4-yl)-9-oxa-10-borabicyclo[3.3.2]decane (5d) 27
(*Z*)-10-(1-phenyl-5-propyldec-4-en-9-yn-4-yl)-9-oxa-10-borabicyclo[3.3.2]decane (5e) 28
(*Z*)-10-(1-phenyl-5-propyldec-4-en-9-yn-4-yl)-9-oxa-10-borabicyclo[3.3.2]decane (5f) 28
(*Z*)-10-(1-phenyl-5-propyldec-4-en-9-yn-4-yl)-9-oxa-10-borabicyclo[3.3.2]decane (5g) 29
ethyl (Z)-4-(9-oxa-10-borabicyclo[3.3.2]decan-10-yl)-7-phenyl-3-propylhept-3-enoate (5h)..... 29
(*Z*)-10-(5-((methylthio)methyl)-1-phenyloct-4-en-4-yl)-9-oxa-10-borabicyclo[3.3.2]decane (5i) 30
(*Z*)-10-(5-((methylthio)methyl)-1-phenyloct-4-en-4-yl)-9-oxa-10-borabicyclo[3.3.2]decane (5j) 30
(*E*)-10-(1-phenyl-5-((4,4,5,5-tetramethyl-1,3,2-dioxaborolan-2-yl)methyl)oct-4-en-4-yl)-9-oxa-10-borabicyclo[3.3.2]decane (5k) 31
(Z)-10-(1-phenyl-5-propylnona-4,8-dien-4-yl)-9-oxa-10-borabicyclo[3.3.2]decane (5l) 32
(Z)-10-(1-phenyl-5-propylnon-4-en-8-yn-4-yl)-9-oxa-10-borabicyclo[3.3.2]decane (5m) 32
(*S*,*Z*)-10-(5-(oxiran-2-ylmethyl)-1-phenyloct-4-en-4-yl)-9-oxa-10-borabicyclo[3.3.2]decane (5n) 33
(*S*,*Z*)-10-(5-(2-(2,2-dimethyl-1,3-dioxolan-4-yl)ethyl)-1-phenyloct-4-en-4-yl)-9-oxa-10-borabicyclo[3.3.2]decane (5o) 33
(*R*,*Z*)-10-(8,12-dimethyl-1-phenyl-5-propyltrideca-4,11-dien-4-yl)-9-oxa-10-borabicyclo[3.3.2]decane (5p) 34
(*E*)-10-(1,2-diphenylbut-1-en-1-yl)-9-oxa-10-borabicyclo[3.3.2]decane (5q) 35
(Z)-10-(2-(benzo[d][1,3]dithiol-2-yl)-1,2-diphenylvinyl)-9-oxa-10-borabicyclo[3.3.2]decane (5r)35
(*Z*)-3-(9-oxa-10-borabicyclo[3.3.2]decan-10-yl)-*N*,*N*-dimethyl-2,3-diphenylprop-2-en-1-amine (5s) 36
(*E*)-10-(1,2-diphenylpenta-1,4-dien-1-yl)-9-oxa-10-borabicyclo[3.3.2]decane (5t) 36
(*E*)-10-(1,2-diphenyl-5-(phenylthio)pent-1-en-1-yl)-9-oxa-10-borabicyclo[3.3.2]decane (5u).... 37
(*Z*)-10-(2-fluoro-1,2-diphenylvinyl)-9-oxa-10-borabicyclo[3.3.2]decane (5v) 37
(*Z*)-10-(1,2-diphenyl-2-(phenylselanyl)vinyl)-9-oxa-10-borabicyclo[3.3.2]decane (5w)........ 38
(*Z*)-10-(2-(methylthio)-1,2-diphenylvinyl)-9-oxa-10-borabicyclo[3.3.2]decane (5x)........ 39
10-(2-methyl-6-phenylhex-2-en-3-yl)-9-oxa-10-borabicyclo[3.3.2]decane (6a) 39
(*E*)-10-(5-methyl-1-phenylnon-4-en-4-yl)-9-oxa-10-borabicyclo[3.3.2]decane (6b) 40

(*E*)-10-(5,7-dimethyl-1-phenyloct-4-en-4-yl)-9-oxa-10-borabicyclo[3.3.2]decane (6c) 40
(*E*)-10-(2-methyl-1,6-diphenylhex-2-en-3-yl)-9-oxa-10-borabicyclo[3.3.2]decane(6d) 41
(*E*)-10-(2,3-dimethyl-7-phenylhept-3-en-4-yl)-9-oxa-10-borabicyclo[3.3.2]decane (6e) 41
(*E*)-10-(2-cyclohexyl-6-phenylhex-2-en-3-yl)-9-oxa-10-borabicyclo[3.3.2]decane (6f) 42
(*E*)-10-(2,6-diphenylhex-2-en-3-yl)-9-oxa-10-borabicyclo[3.3.2]decane (6g) 42
(*E*)-10-(1-methoxy-2-methyl-6-phenylhex-2-en-3-yl)-9-oxa-10-borabicyclo[3.3.2]decane (6h) . 43
(*E*)-(3-(9-oxa-10-borabicyclo[3.3.2]decan-10-yl)-6-phenylhex-2-en-2-yl)trimethylsilane (6i) 43
(*E*)-10-(4-methyl-1-phenylhept-3-en-3-yl)-9-oxa-10-borabicyclo[3.3.2]decane (6j) 44
(*E*)-10-(5-methyl-1-(methylthio)oct-4-en-4-yl)-9-oxa-10-borabicyclo[3.3.2]decane (6k) 44
(*E*)-10-(1-cyclohexyl-3-methylhex-2-en-2-yl)-9-oxa-10-borabicyclo[3.3.2]decane (6l)............. 45
(*E*)-10-(5-methyl-2-phenyloct-4-en-4-yl)-9-oxa-10-borabicyclo[3.3.2]decane(6m) 45
(*E*)-10-(1-(bicyclo[2.2.1]hept-5-en-2-yl)-4-methylhept-3-en-3-yl)-9-oxa-10-borabicyclo[3.3.2]decane (6n) 46
(*R*,*E*)-10-(4,8,12-trimethyltrideca-4,11-dien-5-yl)-9-oxa-10-borabicyclo[3.3.2]decane Spectroscopy (6o) 46
10-((4*E*,6*E*)-4-methyldeca-4,6-dien-5-yl)-9-oxa-10-borabicyclo[3.3.2]decane (6p)................. 47
10-((1*E*,3*E*)-4-methyl-1-phenylhepta-1,3-dien-3-yl)-9-oxa-10-borabicyclo[3.3.2]decane (6q)... 48
10-((1*E*,3*E*)-1-cyclopropyl-4-methylhepta-1,3-dien-3-yl)-9-oxa-10-borabicyclo[3.3.2]decane (6r) 48
10-((2E,4E)-1-methoxy-5-methylocta-2,4-dien-4-yl)-9-oxa-10-borabicyclo[3.3.2]decane(6s) .. 49
(*E*)-10-(1-(4-methoxyphenyl)-2-methylpent-1-en-1-yl)-9-oxa-10-borabicyclo[3.3.2]decane (6t) 50
(*E*)-10-(3-methylhex-2-en-2-yl)-9-oxa-10-borabicyclo[3.3.2]decane (6u)............................... 50
(*E*)-10-(2,6-dimethylnona-1,5-dien-5-yl)-9-oxa-10-borabicyclo[3.3.2]decane (6v) 51
(*E*)-10-(4,9-dimethyldeca-4,8-dien-5-yl)-9-oxa-10-borabicyclo[3.3.2]decane (6w) 51
(*Z*)-1-methoxy-4-(5-methyl-1-phenyloct-4-en-4-yl)benzene ((*Z*)-7) 52
(*E*)-(4-butyl-5-methyloct-4-en-1-yl)benzene ((*E*)-8) 52
(*Z*)-(4-butyl-5-methyloct-4-en-1-yl)benzene ((*Z*)-8) 53
(*Z*)-(4-iodo-5-methyloct-4-en-1-yl)benzene ((*Z*)-10) 54
(*Z*)-trimethyl(4-methyl-3-(3-phenylpropyl)hept-3-en-1-yn-1-yl)silane ((*Z*)-11).......................... 52
(*Z*)-3-methyl-2-(3-phenylpropyl)hex-2-en-1-ol ((*Z*)-12) 55
(*Z*)-3-methyl-2-(3-phenylpropyl)hex-2-enoic acid ((*Z*)-13) 56
(*Z*)-(4-allyl-5-methyloct-4-en-1-yl)benzene ((*Z*)-14)............ 56
(*Z*)-3-methyl-1-phenyl-2-(3-phenylpropyl)hex-2-en-1-ol ((*Z*)-16) 57
cis-Tamoxifen............ 57
(*E*)-4,4'-(hex-3-ene-3,4-diyl)bis(methoxybenzene) (20) 58
(*Z*)-5-allyl-2,6,10-trimethylundeca-1,5,9-triene ((*Z*)-26) 58

(*Z*)-γ-bisabolene 59
(*E*)-5-allyl-2,6,10-trimethylundeca-1,5,9-triene ((*E*)-26) 59
(*E*)-γ-bisabolene 60

1. MATERIALS AND GENERAL INFORMATION

Solvents, Reagents, Glassware and Reaction Setup. Unless otherwise stated, all reactions were conducted under an inert atmosphere of nitrogen in flame dried glassware using standard Schlenk techniques. Air- and moisture-sensitive liquids and solutions were transferred via syringe into the reaction vessels through a rubber septum. Unless otherwise specified, all reagents were purchased at highest commercial quality and used as received. Non-anhydrous solvents were purchased (unless specified) at the highest commercial quality and used as received. CH_2Cl_2, Et_2O and THF were dried on an Anhydrous Engineering alumina column drying system. Temperatures described below −10 °C were achieved using Thermo Scientific EK-90 or Huber TC100E cryostats or appropriate solvent/dry ice baths.

Thin layer chromatography (TLC) was performed using Merck Kieselgel 60 F254 fluorescent treated silica, which was visualised under UV light, or by staining with aqueous basic potassium permanganate followed by heating, or Hanessian's stain (CAM stain) followed by heating, or *p*-anisaldehyde solution followed by heating, as stated.

Preparative Thin layer chromatography (PLC) was performed using Merck Z513032-1PAK TLC plates, Silica gel, which was visualised under UV light.

Chromatography was carried out using Sigma-Aldrich silica gel (60 Å, 230-400 mesh, 40-63 µm) or a Biotage Isolera One automated flash purification system, as indicated. Reactions were followed by thin-layer chromatography (TLC) where practical, using aluminium-backed Merck Kieselgel 60 F254 fluorescent treated silica gel plates, which were visualised under UV light or by staining with aqueous basic $KMnO_4$, acidic *p*-anisaldehyde solution in ethanol, or phosphomolybdic acid solution in ethanol.

NMR were recorded at various field strengths, as indicated, using Bruker 400 MHz, Varian VNMR 400 MHz, or Bruker Cryo 500 MHz for ^{1}H, ^{11}B, and ^{13}C acquisitions. All NMR spectra were recorded at 25 °C unless otherwise stated. Chemical shifts (δ) are reported in parts per million (ppm) and referenced to $CDCl_3$ (^{1}H: 7.26 ppm; ^{13}C: 77.16 ppm) or CD_2Cl_2 (^{1}H: 5.32

ppm; ^{13}C: 53.84 ppm). Coupling constants (*J*) are given in Hertz (Hz) and refer to apparent multiplicities (s = singlet, d = doublet, t = triplet, q = quartet, quin = quintet, hex = hextet, h = heptet, m = multiplet, brs = broad signal, dd = doublet of doublets, etc.). The ^{1}H NMR spectra are reported as follows: chemical shift (multiplicity, coupling constants, number of protons).

HRMS (high resolution mass spectra) were recorded on a Bruker Daltonics MicrOTOF II by Electrospray Ionisation (ESI); a Thermo Scientific QExactive by Electron Ionisation (EI); a Thermo Scientific Orbitrap Elite by ESI or Atmospheric Pressure Chemical Ionisation (APCI); or a Bruker UltrafleXtreme by Matrix-assisted Laser Desorption/Ionisation (MALDI). Only molecular ions ($[M+H]^+$, or $[M+Na]^+$) are reported.

Naming of compounds. Compound names are generated by ChemDraw Professional 20.0 software (PerkinElmer), following the IUPAC nomenclature.

2. EXPERIMENTAL DATA

2.1. Reaction Optimization

Table S1: Detailed reaction optimization for the 1,2-migration of alkynyl boronate complex **3a** with ethylating reagent

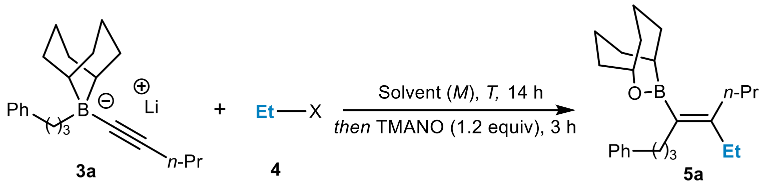

Entry	Et-X	solvent	*M*	*T*	yield%[a] (*Z/E*[b])
1	Et-I	THF	0.1 M	40 °C	trace
2	Et-I	THF	0.1 M	60 °C	8
3	Et-I	THF	0.1 M	80 °C	3
4	Et-OTs	THF	0.1 M	60 °C	9
5	Et_2SO_4	THF	0.1 M	60 °C	11
6	$Et_3O^+BF_4^-$	THF	0.1 M	40 °C	69 (>20:1)
7	Et-I	THF	0.5 M	60 °C	35 (>20:1)
**8**	Et-I	**THF**	**1 M**	**60 °C**	**77** (>20:1)
9	Et-OTs	THF	1 M	60 °C	79 (>20:1)
**10**	Et-OTs	**THF**	**1 M**	**40 °C**	**79** (>20:1)
11	Et-OTs	PhMe	1 M	40 °C	59 (>20:1)
12	Et-OTs	EtOAc	1 M	40 °C	68 (>20:1)
13	Et-OTs	DCM	1 M	40 °C	21 (>20:1)
14	Et-OTs	MeCN	1 M	40 °C	19 (>20:1)

The reaction was conducted with 0.5 mmol of **2a** (based on allylbenzene), 0.75 mmol of **3**. TMANO (0.60 mmol) in 1 mL of DCM was added via syringe. [a] yields refer to isolated yield. [b] *Z/E* ratio was determined by crude ^1H NMR. TMANO = trimethylammonium *N*-oxide; EtOAc = ethyl acetate; THF = tetrahydrofuran.

2.2. Summarized electrophiles in this reaction

Table S2: Structure of electrophiles used in Figure 2 and Extended Data Figure 1

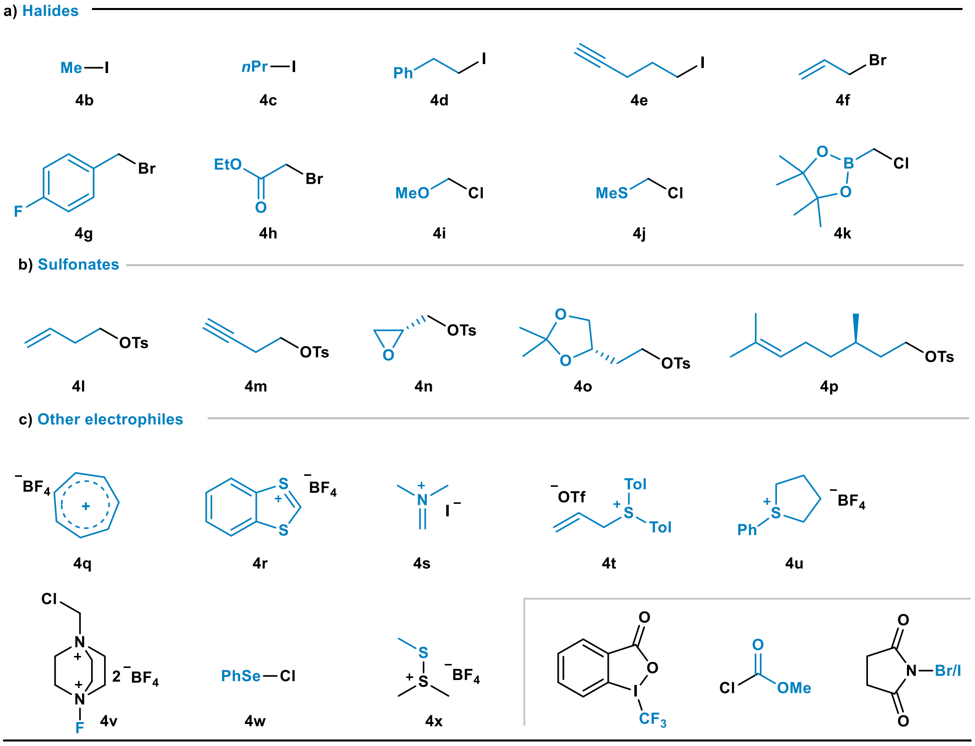

2.3. General Procedures

2.3.1 General Procedure A for product 5a-p and 6a-s

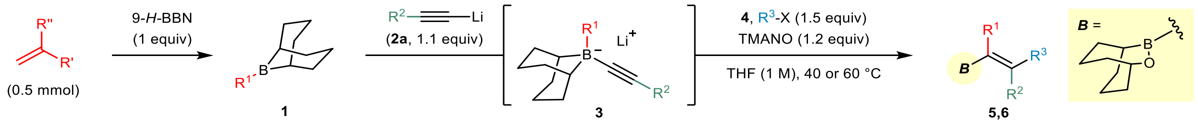

Step 1. Hydroboration: A flame dried 8 mL Biotage® microwave reaction **vial-1** equipped with a magnetic stir bar was purged with nitrogen (N_2) three times. To this vial was added alkene or alkyne (0.5 mmol, 1.0 equiv) under nitrogen. The solution was cooled to 0 °C using an ice bath and 9-*H*-BBN solution (0.5 M in THF, 0.5 mmol, 1.0 equiv) was slowly added. The mixture was allowed to warm to 25 °C and stirred for 3 hours at this temperature to ensure complete hydroboration. **Step 2. Formation of alkynyl-ate complex :** In a separated, flame dried 8 mL Biotage® microwave reaction **vial-2** equipped with a magnetic stir bar was purged with nitrogen (N_2) three times. To this vial was added alkyne (0.55 mmol, 1.1 equiv) and anhydrous THF (2.0 mL) under nitrogen. The solution was cooled to -78 °C using a dry ice/acetone bath and *n*-BuLi (1.6 M in hexane, 0.55 mmol, 1.1 equiv) was added dropwise. The reaction mixture was stirred for 30 minutes at this temperature, then borane in **vial-1** was

carefully transferred to **vial-2** via syringe. The mixture was allowed to warm to 25 °C and stirred for 1 hour at this temperature to ensure complete formation of alkynyl ate complex. **Step 3. 1,2-Migration of alkynyl-ate complex:** All solvents were carefully evaporated under reduce pressure, then 0.5 mL of anhydrous THF (c = 1.0 M) and alkyl halide/sulfonate (0.075 mmol, 1.5 equiv) were sequentially added. After stirring at 40 °C (for alkyl sulfonates) or 60 °C (for alkyl halides) for 12-24 hours, the reaction was cooled to 0 °C and trimethylammonium *N*-oxide (TMANO, 0.6 mmol, 1.2 equiv) in DCM (1.0 mL) was added dropwise to oxidise alkenyl borane to more stable alkenyl borinic ester. After stirring for 2 hours, the crude reaction mixture was filtered through a celite pad, washed with pentane (3 x 10 mL) and the filtrate was concentrated under reduced pressure. The resulting crude material was purified by flash column chromatography (aluminum oxide neutral) or preparative thin-layer chromatography to isolate the desired product.

Note: **1)** After **Step 3**, 1,3,5-trimethoxybenzene was added as an internal standard, and the NMR yield was determined.

2) Some borinic esters were less stable and would decompose using silica gel or neutral aluminum oxide for flash column chromatography, giving a mixture of borinic ester and protodeboronation product which are difficult to separate. In these cases, fast flash column chromatography (less than 10 min) using a short column (1.5 cm diameter and 7 cm length, for 0.5 mmol scale) or preparative TLC are recommended.

4) Borinic esters were found to be slightly unstable in $CDCl_3$, probably due to the presence of a trace amount of HCl in $CDCl_3$. To avoid decomposition that leading to impure NMR spectra, K_2CO_3-neutralized $CDCl_3$ or other non-acidic deuterated solvents (e.g. CD_2Cl_2, *d8*-toluene) are recommended.

2.3.2 General Procedure B for product 5q-x

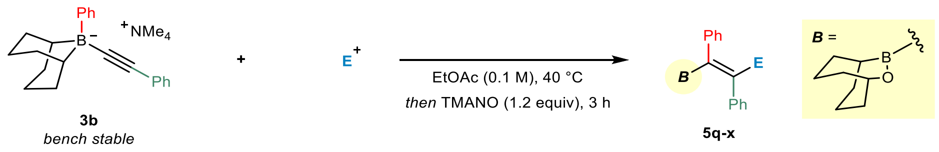

A flame dried 8 mL Biotage® microwave reaction vial equipped with a magnetic stir bar was charged with **3b** (0.2 mmol, 1.0 equiv). The vial was transferred into an anhydrous, argon-filled glovebox where anhydrous EtOAc was added (2.0 mL, c = 0.10 M, when E^+ = selectfluor, THF was employed instead of EtOAc) followed by the electrophile (0.24 mmol, 1.2 equiv).

The vial was sealed with a cap with septum, removed from the glovebox. The mixture was stirred at 40 °C for 4-12 hours before cooling down to 0 °C. Then, trimethylammonium *N*-oxide (TMANO, 0.24 mmol, 1.2 equiv) in DCM (1.0 mL) was added dropwise to oxidise alkenyl borane to alkenyl borinic ester. After stirring the mixture for 2 hours, the crude reaction mixture was filtered through a celite pad, washed with Et_2O (3 x 10 mL) and the filtrate was concentrated under reduced pressure. The resulting crude material was purified by flash column chromatography (aluminum oxide neutral) or preparative thin-layer chromatography to isolate the desired product.

2.3.3 General Procedure C for product 6t-w

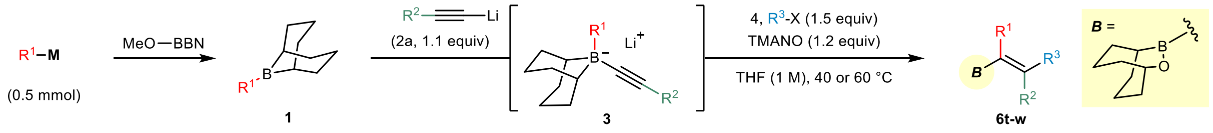

Step 1. Formation of alkynyl-ate complex : In a flame dried 8 mL Biotage® microwave reaction vial equipped with a magnetic stir bar was purged with nitrogen (N_2) three times. To this vial was added alkyne (0.55 mmol, 1.1 equiv) and anhydrous THF (2.0 mL) under nitrogen. The solution was cooled to -78 °C using a dry ice/acetone bath and *n*-BuLi (1.6 M in hexane, 0.55 mmol, 1.1 equiv) was added dropwise. The reaction mixture was stirred for 30 min at this temperature, then borane (0.5 mmol, prepared from organometal reagent and *B*-methoxyl-BBN and purified via distillation) was added dropwise via syringe. The mixture was allowed to warm to 25 °C and stirred for 1 hour at this temperature to ensure complete formation of alkynyl-ate complex. **Step 2. 1,2-Migration of alkynyl-ate complex:** All solvents were carefully evaporated under reduce pressure, then 0.5 mL of anhydrous THF (c = 1.0 M) and Me-OTs (0.075 mmol, 1.5 equiv) were sequentially added. After stirring at 40 °C for 12 hours, the reaction was cooled down to 0 °C and trimethylammonium *N*-oxide (TMANO, 0.6 mmol, 1.2 equiv) in DCM (1.0 mL) was added dropwise to oxidise alkenyl borane to more stable alkenyl borinic ester. After stirring the mixture for 2 hours, the crude reaction mixture was filtered through a celite pad, wash with pentane (3 x 10 mL) and the filtrate was concentrated under reduced pressure. The resulting crude material was purified by preparative thin-layer chromatography to isolate the desired product.

2.4. Reaction of Steric Hindered Boranes

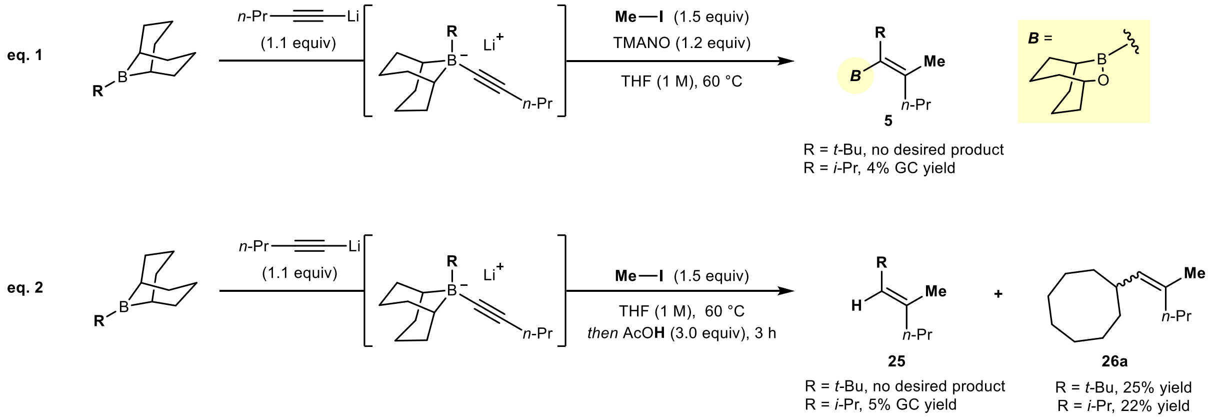

When *i*-Pr-9-BBN and *t*-Bu-9-BBN were used, while the formation of "ate" complexes were confirmed to be versatile by ^{11}B NMR, the reactions were found to be sluggish giving a mixture of unidentified products (equation 1, S1-Figure 1). For *t*-Bu-9-BBN, no desired product was detected by GC-MS while 4% GC yield was determined for *i*-Pr-9-BBN).

When we treated the reaction mixture with acetic acid, the major side product was identified to be the ring migration product **26a** (*E/Z*-mixture), which must be formed from migration of the bicyclo[3.3.1]nonane ring (equation 2, S1-Figure 1).

It is proposed that when bulky boranes are used, the steric repulsion between the bulky migration group and the electrophile is less favored and instead, bicyclo[3.3.1]nonane ligand migrates to give **27**, which is converted to **26** after acid workup (see S1-Figure S2).

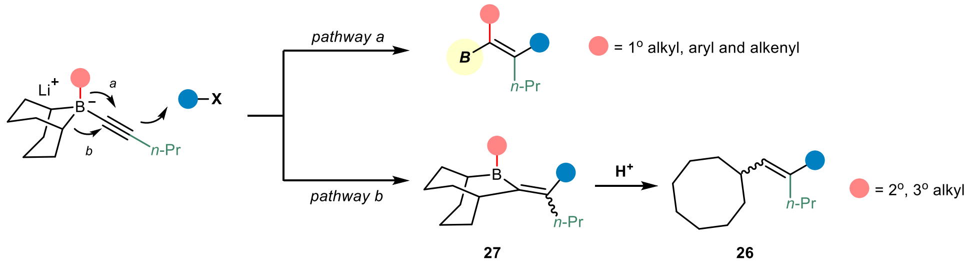

Figure S1: Proposed reaction pathway for different 9-BBN boranes.

^{11}B NMR (400 MHz, THF) of ate complex from *i*Pr-BBN

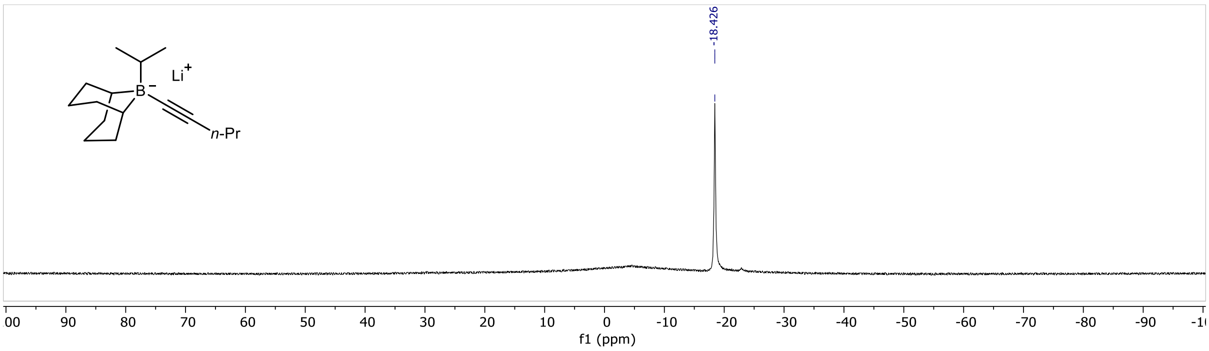

^{11}B NMR (400 MHz, THF) of ate complex from *t*Bu-BBN

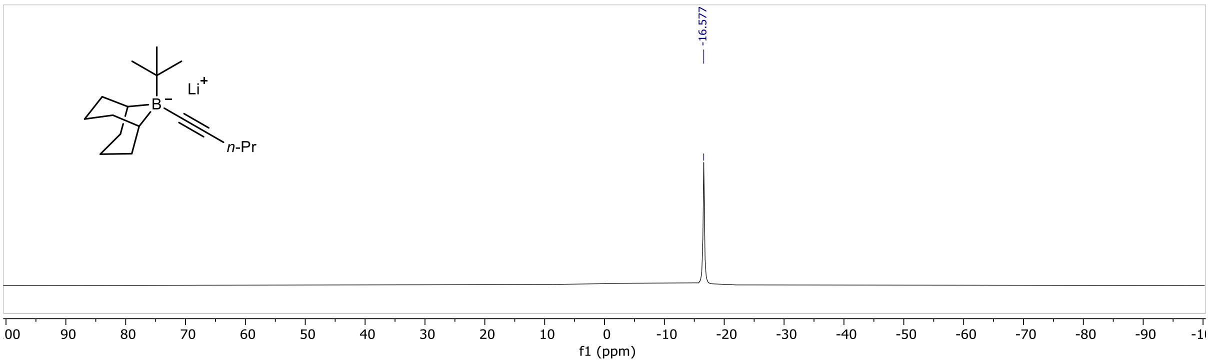

^{1}H NMR (400 MHz, $CDCl_3$) of **26a**

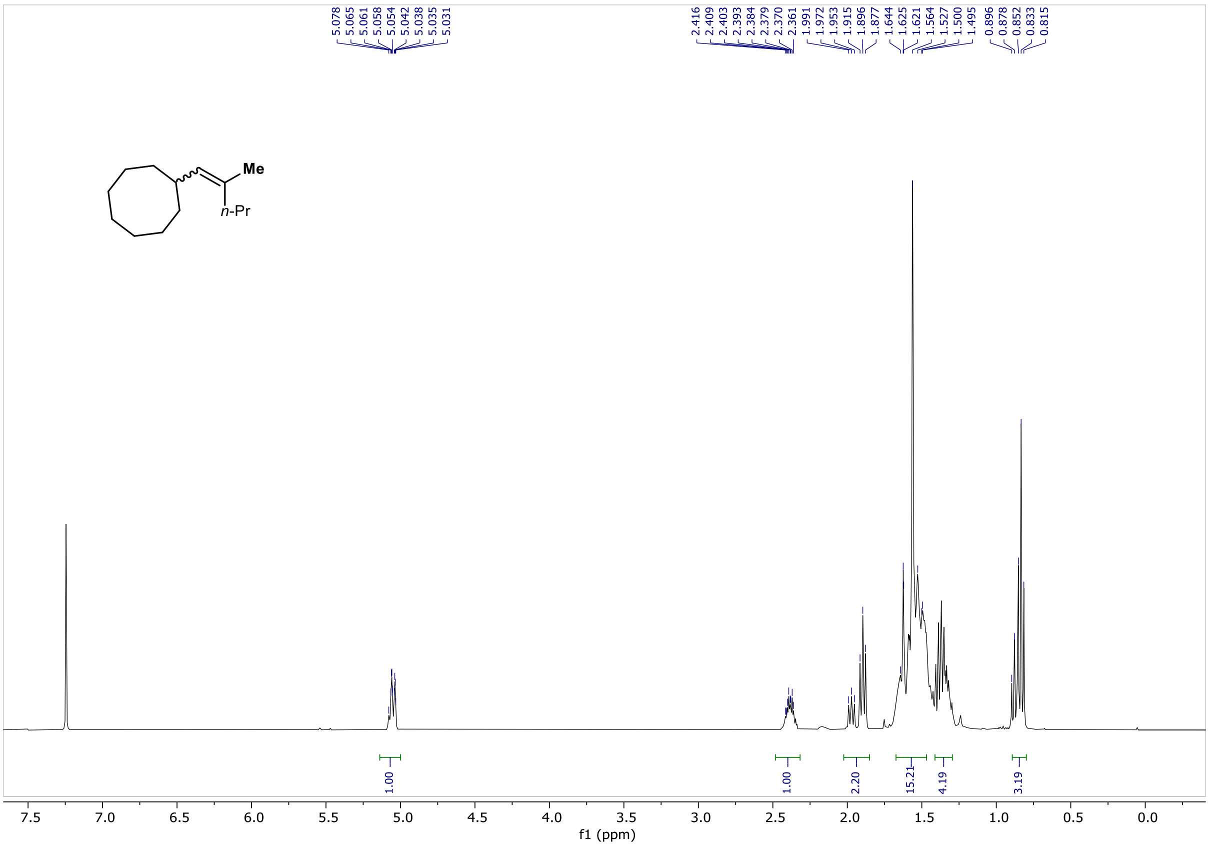

2.5. Comparison of Ph-9-BBN and Ph_3B as borane sources

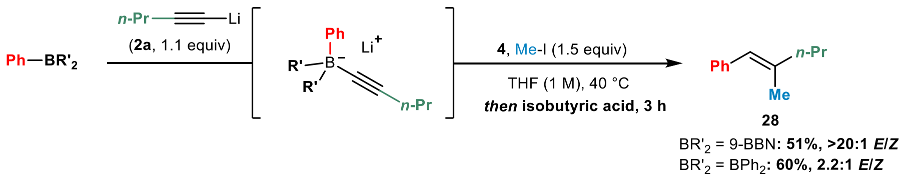

We tested two different borane Ph-9-BBN and Ph_3B, in the methylation reaction under identical reaction conditions. The results showed that these two reactions gave similar yield, while much greater stereoselectivity was obtained with 9-BBN borane. It is believed that the bulky and non-migrating bicyclo[3.3.1]nonane ring plays a crucial role in stereocontrol of this reaction.

^{1}H NMR (400 MHz, $CDCl_3$) of **28** from different borane sources

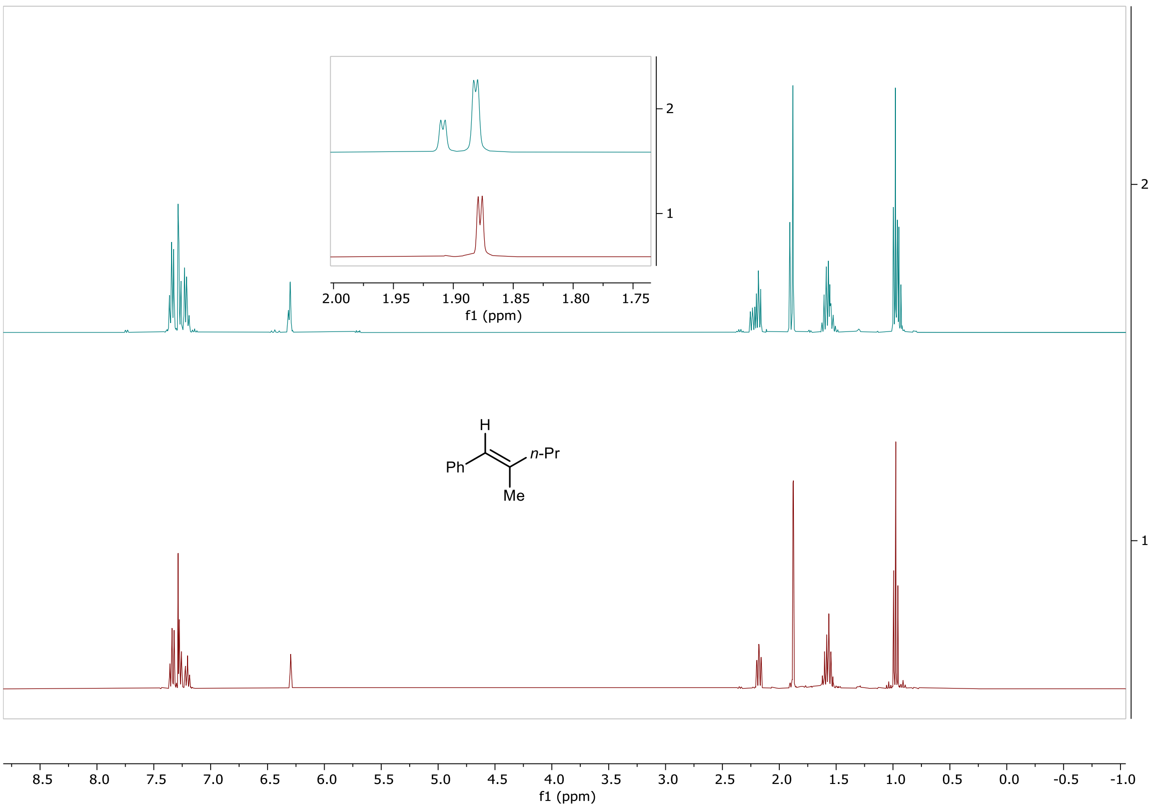

2.6. Synthesis of Starting Materials

All alkenes, alkynes, alkyl halides/sulfonates (unless specified), compound **4q-4s** and **4v-x** were purchased at the highest commercial quality and used as received. Alkyl sulfonates **4l-p**,[1] allyl di(*p*-tolyl)sulfonium,[2] 1-phenyl tetrahydrothiophenium[3] and boranes[4] were synthesized according to relevant literatures.

2.7. Determination of Stereochemistry of 5a and 5b

The stereochemistry of compound **5a** was determined through 1D selective NOESY NMR experiment. In the NMR spectra that shown below, the noe effect of **H1-H5** and **H1-H6** were clearly observed. Meanwhile, no noe effect of **H1-H8**, **H1-H9** or **H1-H10** were observed. These results indicates that (*E*)-**5a** was obtained from our methodology.

1D selective NOESY (500 MHz, CD_2Cl_2) of **5a**

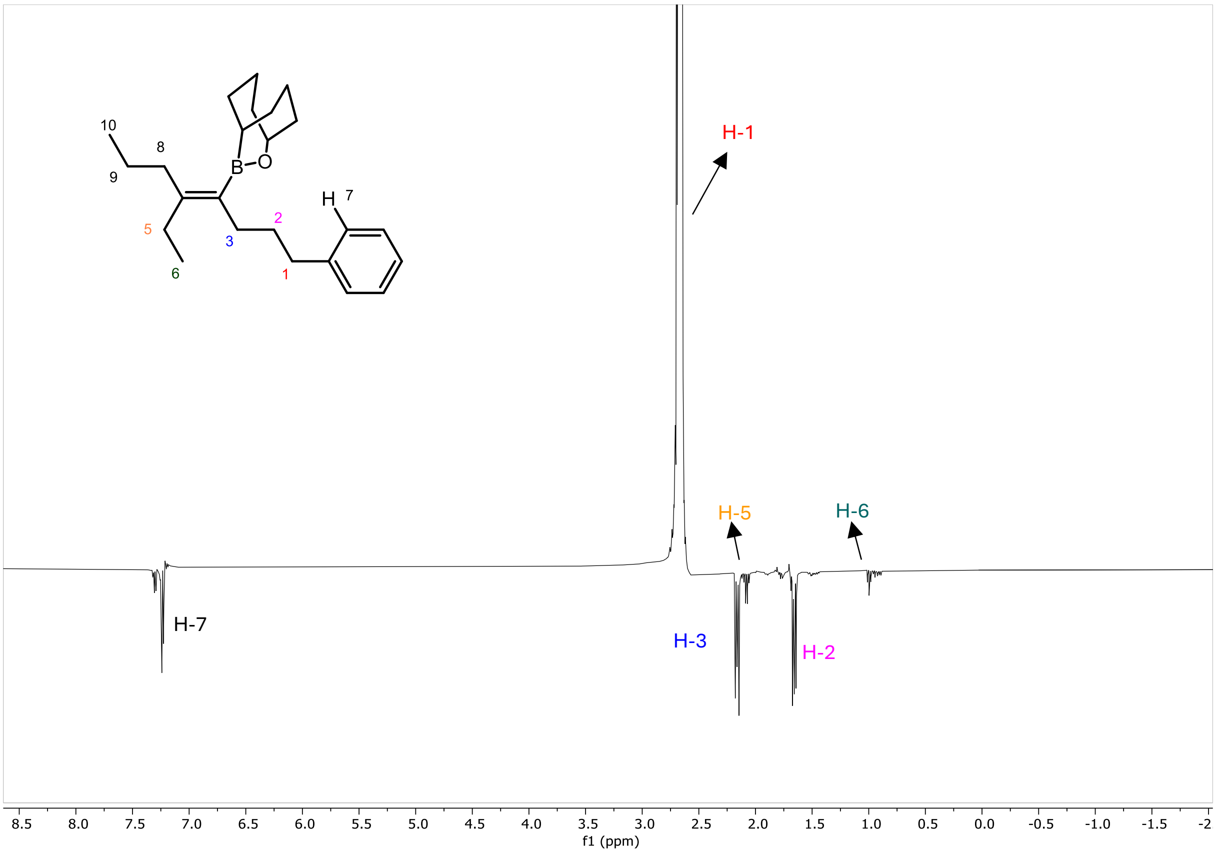

The stereochemistry of **5** was further confirmed by the protodeboronation product of **5b**. Hydroboration of trisubstituted alkenyl borane will give trisubstituted alkene in a stereoretentive manner.[5] Therefore, determination the stereochemistry of protodeboronation product will provide stereochemical information of initially formed trisubstituted alkenyl borane. In the NMR spectra that shown below, the noe effect of **H1-H2**, **H1-H3** and **H1-H4** were clearly observed. Meanwhile, no noe effect of **H1-H5** was observed. These results also confirmed (*E*)-isomer was obtained from our methodology.

COSY (500 MHz, $CDCl_3$) of **S5b-H**

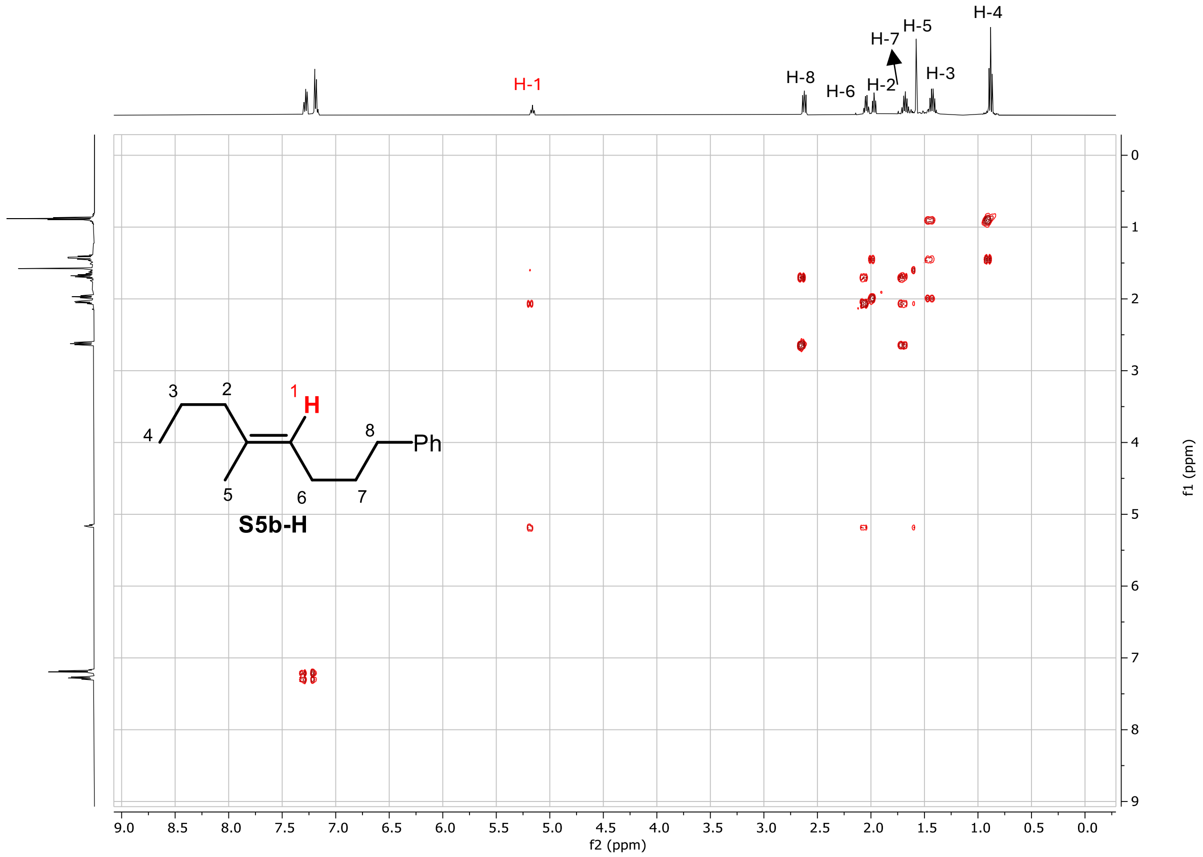

1D selective NOESY (500 MHz, $CDCl_3$) of **S5b-H**

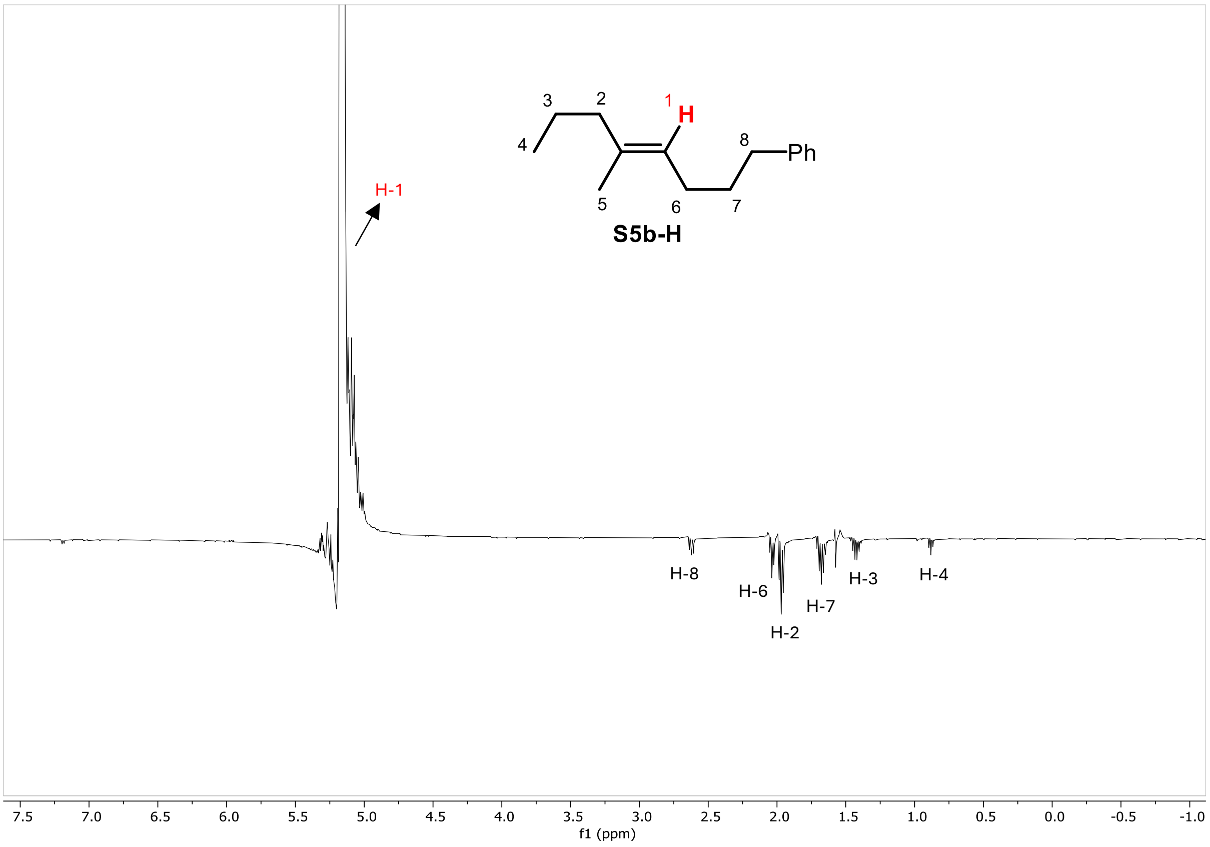

2.8. Procedures for Transformation of Products

2.8.1 *sp*^{2}-*sp*^{2} and *sp*^{2}-*sp*^{3} cross coupling of 5b[5,6]

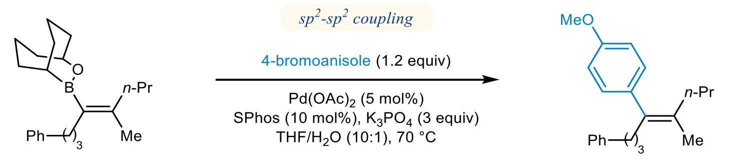

5b, crude (*Z*)-**7**, 81%, >20:1 *Z*/*E*

The borinic ester **5b** was prepared following **General Procedure A** in 1.0 mmol scale and used directly without chromatography purification for transformation. The amount of **5b** in crude mixture was quantified via crude ^{1}H NMR using CH_2Br_2 as internal standard.

A 25 mL Schlenk flask equipped with a magnetic stir bar was charged with $Pd(OAc)_2$ (0.01 mmol, 5 mol%), SPhos (0.02 mmol, 10 mol%) and K_3PO_4 (0.60 mmol, 3.0 equiv) then purged with nitrogen (N_2) three times. To this flask was sequentially added crude **5b** (0.20 mmol, 1.0 equiv) in THF (degassed, 2 mL), H_2O (degassed, 0.2 mL) and 4-bromoanisole (0.24 mmol, 1.2 equiv). The mixture was stirred for overnight at 70 °C before cooling to ambient temperature. H_2O (5 mL) and Et_2O (5 mL) were added to the mixture. The organic layer was separated, and the aqueous layer was extracted with Et_2O (3 x 5 mL). The combined organic layers were dried over anhydrous $MgSO_4$, filtered and concentrated under reduced pressure. Purification of the yellow oil residue by silica gel chromatography (pentane/ethyl acetate = 30/1) afforded (*Z*)-**7** as a colorless oil (49.9 mg, 81%, >20:1 *Z*/*E*).

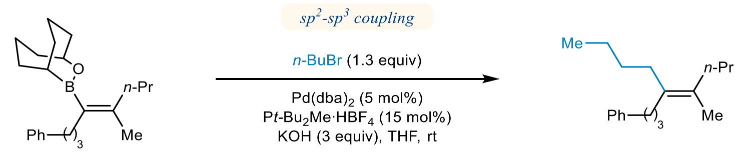

5b, crude (*E*)-**8**, 95%, >20:1 *E*/*Z*

The borinic ester **5b** was prepared following **General Procedure A** in 1.0 mmol scale and used directly without chromatography purification for transformation. The amount of **5b** in crude mixture was quantified via crude ^{1}H NMR using CH_2Br_2 as internal standard.

A 25 mL Schlenk flask equipped with a magnetic stir bar was charged with $Pd(dba)_2$ (0.01 mmol, 5 mol%), *t*-$Bu_2MeP{\cdot}HBF_4$ (0.03 mmol, 15 mol%) and KOH (0.60 mmol, 3.0 equiv) then purged with nitrogen (N_2) three times. To this flask was sequentially added crude **5b** (0.20 mmol, 1.0 equiv) in anhydrous THF (degassed, 1.0 mL) and *n*-butylbromide (0.26 mmol, 1.3 equiv). The mixture was then stirred for 24 hours at ambient temperature. After evaporating all

volatile materials, the residue was directly purified by silica gel chromatography (pentane), yielding (*E*)-**8** as a colorless oil (49.0 mg, 95%, >20:1 *E*/*Z*).

2.8.2 Zweifel Olefination of Borinic Ester[7]

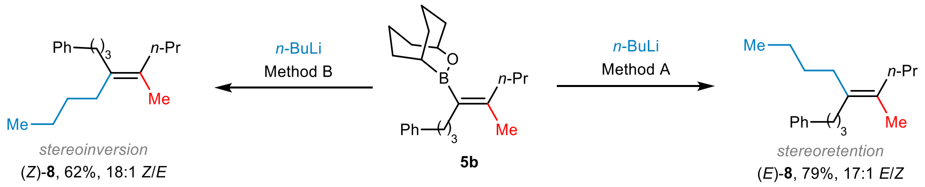

The borinic ester **5b** and **5y** were prepared following **General Procedure A** in 1.0 mmol scale and purified by flash column chromatography (aluminum oxide, neutral for chromatography)

Synthesis of (*E*)-8 (Method A): A flame-dried 25 mL Schlenk flask equipped with a magnetic stir bar was purged with nitrogen (N_2) three times. To this flask was added **5b** (0.20 mmol, 1.0 equiv) in anhydrous THF (1.0 mL) and the solution was cooled down to -78 °C using a dry ice/acetone bath. *n*-BuLi (1.6 M in hexane, 0.60 mmol, 3.0 equiv) was added dropwise then the mixture was allowed to stir at 0 °C for 1 hour before cooling to -78 °C. A solution of I_2 (0.50 mmol, 2.5 equiv) in MeOH (1.0 mL) was added dropwise and the resulting mixture was allowed to warm to 60 °C and stir at this temperature for 12 hours. The reaction was quenched by an addition of a saturated aqueous solution of $Na_2S_2O_3$ (2.0 mL). The aqueous layer was washed with Et_2O (3 × 5 mL), and the combined organic layers were dried over $MgSO_4$ and concentrated in vacuo. Purification of the yellow oil residue by silica gel chromatography (pentane) affords (*E*)-**8** as a colorless oil (40.8 mg, 79% yield, 17:1 *E*/*Z*).

Synthesis of (*Z*)-8 (Method B): A flame-dried 25 mL Schlenk flask equipped with a magnetic stir bar was purged with nitrogen (N_2) three times. To this flask was added **5b** (0.20 mmol, 1 equiv) in anhydrous THF (1.0 mL) and the solution was cooled down to -78 °C using a dry ice/acetone bath. *n*-BuLi (1.6 M in hexane, 0.60 mmol, 3.0 equiv) was added dropwise then the mixture was allowed to stir at 0 °C for 1 hour before cooling to -78 °C. A solution of PhSeCl (0.22 mmol, 1.1 equiv) in THF (0.2 mL) was added dropwise. The resulting solution was stirred at -78 °C for 1 hour and then warmed to room temperature and stirred for a further 2 hours and then cooled to -78 °C. A solution of sodium methoxide (0.5 M in MeOH, 5.0 equiv) was added dropwise and the resulting solution was stirred at -78 °C for 1 hour and a further 2 hours at 0 °C. The reaction was quenched by an addition of a saturated aqueous solution of $Na_2S_2O_3$ (2.0 mL). The aqueous layer was washed with Et_2O (3 × 5 mL), and the combined organic layers were dried over $MgSO_4$ and concentrated in vacuo. Purification of the yellow oil residue

by silica gel chromatography (pentane) affords (*Z*)-**8** as a colorless oil (32.0 mg, 62%, 18:1 *Z*/*E*).

Synthesis of (*Z*)-9 (Method A): (*Z*)-9 was prepared following **General Procedure A** and **Method A** from **3a**, *n*-BuOTs and MeLi and isolated as a colorless oil (37.7 mg, 73%, >20:1 *Z*/*E*).

Synthesis of (*E*)-9 (Method B): (*E*)-9 was prepared following **General Procedure A** and **Method B** from **3a**, *n*-BuOTs and MeLi and isolated as a colorless oil (27.9 mg, 54%, 19:1 *E*/*Z*).

2.8.3 Iodination to (*Z*)-10

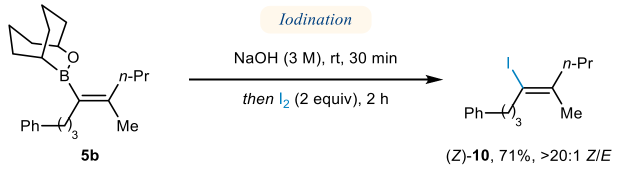

Prepared following a literature procedure[8] with modifications. A solution of **5b** (0.20 mmol) in Et_2O (1.0 mL) was treated with aqueous NaOH (3 M, 0.60 mmol, 3.0 equiv) at 0 °C and stirred at this temperature for 30 minutes. Then a solution of I_2 (0.40 mmol, 2.0 equiv) in Et_2O (1.0 mL) was added to the reaction mixture. After stirring for 2 hours at 0 °C, the reaction was quenched by an addition of a saturated aqueous solution of $Na_2S_2O_3$ (2.0 mL). The aqueous layer was washed with pentane (3 × 5 mL), and the combined organic layers were dried over $MgSO_4$ and concentrated in vacuo. Purification of the brown oil residue by silica gel chromatography (pentane) affords (*Z*)-**10** as a pale-yellow oil (46.6 mg, 71%, >20:1 *Z*/*E*).

Note: Alkenyl iodide (*Z*)-**10** is sensitive to light and should be stored in a dark place.

2.8.4 Alkynylation to (*Z*)-11

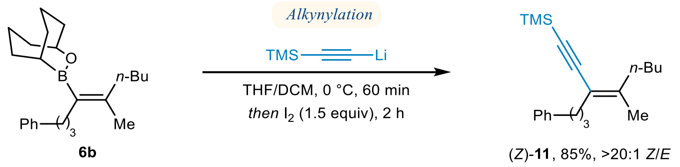

Prepared following a literature procedure[9] with modifications. A solution of trimethylsilylacetylene (0.30 mmol, 1.5 equiv) in anhydrous THF (1.0 mL) was treated with *n*-BuLi (1.6 M in hexane, 0.30 mmol, 1.5 equiv) at -78 °C and stirred at this temperature for 45 minutes. Then a solution of **6b** (0.20 mmol, 1.0 equiv) in THF (1.0 mL) was added dropwise

to the reaction mixture, and the mixture was allowed to warm to ambient temperature. After stirring for 1 hour, the reaction was cooled to -78 °C, and a solution of I_2 (0.30 mmol) in THF (1.5 mL) was added dropwise. The solution was stirred at -78 °C for an additional 20 minutes and aqueous NaOH (1 M, 1.0 mL) was added followed by 30% H_2O_2 (0.3 mL). The cold bath was removed, and the mixture was warmed to ambient temperature then diluted with H_2O/DCM (10 mL 1:1). The organic layer was washed with brine, dried over $MgSO_4$ and concentrated in vacuo. Purification of the brown oil residue by silica gel chromatography (pentane) affords (*Z*)-**11** as a colorless oil (53.0 mg, 85%, >20:1 *Z*/*E*).

2.8.5 Matteson Homologation to (*Z*)-12

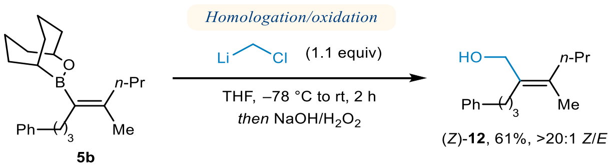

Prepared following a literature procedure[10] with modifications. A 10 mL Schlenk tube was charged with **5b** (0.20 mmol, 1.0 equiv), anhydrous THF (1.0 mL) and ICH_2Cl (0.30 mmol, 1.5 equiv) under nitrogen. *n*-BuLi (1.6 M in hexane, 0.22 mmol, 1.1 equiv) was added very slowly to this solution at -78 °C using a dry ice/acetone bath. The reaction mixture was allowed to warm to ambient temperature in 6 hours. Then, aqueous NaOH (2 M, 0.5 mL) and 30% H_2O_2 (0.5 mL) was added in sequence at 0 °C. After stirring at 0 °C for 2 hours, the reaction was diluted with brine and extracted with pentane/ethyl acetate (5:1). The organic phase was dried over anhydrous $MgSO_4$ and concentrated in vacuo. Purification of the colorless oil residue by silica gel chromatography (pentane/ethyl acetate, 20:1 to 10:1) affords (*Z*)-**12** as a colorless oil (28.3 mg, 61%, >20:1 *Z*/*E*).

2.8.6 Carboxylation to (*Z*)-13

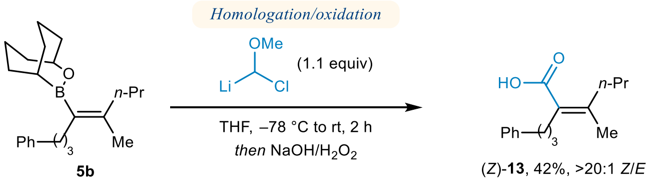

Prepared following a literature procedure[11] with modifications. A 10 mL Schlenk tube was charged with **5b** (0.50 mmol, 1.0 equiv), anhydrous THF (0.5 mL) and α,α-dichloromethyl methyl ether (0.75 mmol, 1.5 equiv) at 0 °C, a lithium *tert*-butoxide solution prepared from *tert*-butanol (2.50 mmol, 5.0 equiv) and *n*-BuLi (1.6 M in hexane, 2.50 mmol, 5.0 equiv) was

added dropwise via syringe. After the addition, the reaction mixture was allowed to warm to ambient temperature, which produced a white precipitate (Li salt). After stirring 4 hours at this temperature, the solvents were removed in vacuo. Ethanol (2.0 mL) and solid NaOH (6.0 mmol) were added, followed by the dropwise addition of 30% H_2O_2 (1.0 mL) at 0 °C. The mixture was heated at 50 °C for 3 hours to destroy the excess of H_2O_2, cooled to room temperature, acidified with 3 N HCl, saturated with sodium chloride, and extracted with ethyl acetate (3 x 10 mL). The organic phase was dried over anhydrous $MgSO_4$ and concentrated in vacuo. Purification of the yellow oil residue by silica gel chromatography (pentane/ethyl acetate, 10:1 to 5:1) affords (*Z*)-**13** as a colorless oil (51.7 mg, 42%, >20:1 *Z*/*E*).

2.8.7 Zweifel Olefination of Borane

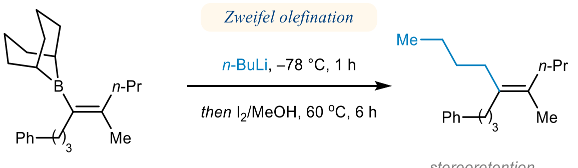

5b'

(*E*)-**8**, 79%, >20:1 *E*/*Z*

To a THF (0.5 mL) solution of alkynyl-ate complex **3a** (0.50 mmol, prepared in situ from **General Procedure A**, **step 1** and **step 2**) was added MeI (0.75 mmol, 1.5 equiv). The mixture was stirred at 60 °C for 14 hours, cooled to 0 °C and all volatile materials (THF and excess MeI) were carefully removed under reduced pressure. To this crude borane **5b'** residue was added anhydrous THF (3.0 mL) and the mixture was cooled to -78 °C using a dry ice/acetone bath. *n*-BuLi (1.6 M in hexane, 1.5 mmol, 3.0 equiv) was added dropwise then the mixture was stirred at -78 °C for 1 hour. Then, a solution of I_2 (1.25 mmol, 2.5 equiv) in MeOH (2.5 mL) was added dropwise and the resulting mixture was allowed to warm to 60 °C and stir for further 6 hours. The reaction was quenched by an addition of a saturated aqueous solution of $Na_2S_2O_3$ (2.0 mL). The aqueous layer was washed with Et_2O (3 × 5 mL), and the combined organic layers were dried over $MgSO_4$ and concentrated in vacuo. Purification of the yellow oil residue by silica gel chromatography (pentane) affords (*E*)-**8** as a colorless oil (101.9 mg, 79%, >20:1 *E*/*Z*).

2.8.8 Transmetallation of Borane to Copper(I) and Subsequent Allylation[12]

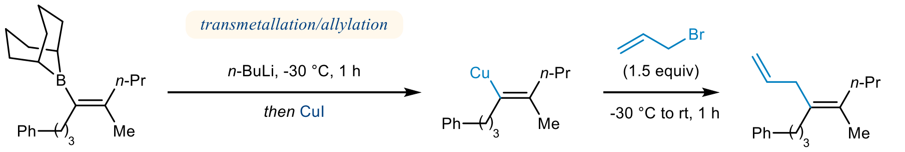

5b'

(*Z*)-**14**, 69%, >20:1 *Z*/*E*

To a THF (0.5 mL) solution of alkynyl-ate complex **3a** (0.50 mmol, prepared in situ from **General Procedure A**, **step 1** and **step 2**) was added MeI (0.75 mmol, 1.5 equiv). The mixture was stirred at 60 °C for 14 hours, cooled to 0 °C and all volatile materials (THF and excess MeI) were carefully removed under reduced pressure. To this crude borane residue was added anhydrous THF (1.5 mL) and the mixture was cooled to -30 °C. *n*-BuLi (1.6 M in hexane, 1.0 mmol, 2.0 equiv) was added dropwise then the mixture was stirred at -30 °C for 1 hour. Copper(I) iodide (0.55 mmol, 1.1 equiv) was added to the red/orange solution in one portion, quickly resulting a brown/black solution. Then, allylbromide (1.5 mmol, 3.0 equiv) was added dropwise via syringe and the cold bath was removed. After stirring at ambient temperature for 1 hour, the mixture was cooled to 0 °C followed by sequential addition of aqueous NaOH (3 M, 1.0 mL) and 30% H_2O_2 (0.5 mL). After stirring for 30 minutes, excess H_2O_2 was quenched by an addition of a saturated aqueous solution of $Na_2S_2O_3$ (1.0 mL). The aqueous layer was washed with Et_2O (3 × 5 mL), and the combined organic layers were dried over $MgSO_4$ and concentrated in vacuo. Purification of the yellow oil residue by silica gel chromatography (pentane) affords (*E*)-**8** as a colorless oil (83.5 mg, 69%, >20:1 *Z*/*E*).

2.8.9 Grignard-type Addition of Borane to Aldehyde[13]

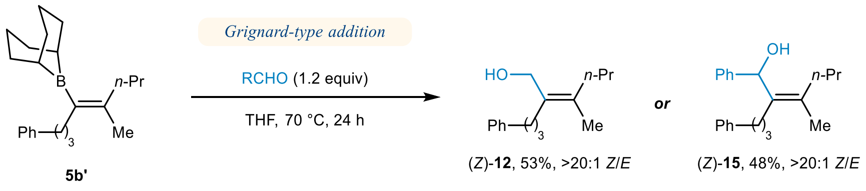

To a THF (0.5 mL) solution of alkynyl-ate complex **3a** (0.50 mmol, prepared in situ from **General Procedure A**, **step 1** and **step 2**) was added MeI (1.0 mmol, 2.0 equiv) and formaldehyde monomer or benzaldehyde (0.60 mmol, 1.2 equiv). The mixture was stirred at 60 °C for 14 hours before cooled to 0 °C. Dilute the solution with ethyl acetate (5.0 mL), then aqueous NaOH (3 M, 1.0 mL) and 30% H_2O_2 (0.5 mL) were added in sequence. After stirring for 30 minutes, excess H_2O_2 was quenched by an addition of a saturated aqueous solution of $Na_2S_2O_3$ (1.0 mL). The aqueous layer was washed with Et_2O (3 × 5 mL), and the combined organic layers were dried over $MgSO_4$ and concentrated in vacuo. Purification of the yellow oil residue by silica gel chromatography (pentane/ethyl acetate, 20:1 to 10:1) affords (*Z*)-**12** (61.5 mg, 53% yield, >20:1 *E*/*Z*) and (*Z*)-**15** (73.9 mg, 49%, >20:1 *E*/*Z*) as a colorless oil.

2.8.10 Synthesis of *cis*-Tamoxifen

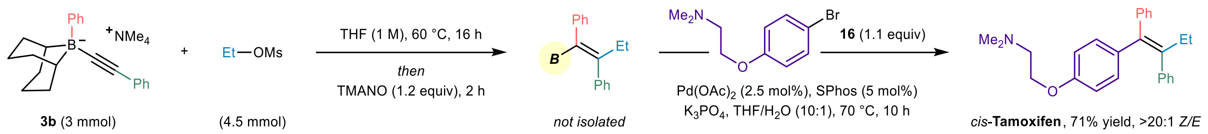

A 25 mL flame-dried Schlenk tube was sequentially added **3b** (3.0 mmol, 1.0 equiv), anhydrous THF (3.0 mL) and ethyl mesylate (4.5 mmol, 1.5 equiv) under nitrogen. The reaction mixture was allowed to stir at 60 °C for 16 hours before cooled to 0 °C. Then, trimethylammonium *N*-oxide (3.6 mmol, 1.2 equiv) was added in one portion and the reaction mixture was stirred at ambient temperature for 2 hours to oxidise alkenyl borane to more stable alkenyl borinic ester, which was used directly for the next step.

To the reaction mixture was added $Pd(OAc)_2$ (0.15 mmol, 5 mol%), SPhos (0.30 mmol, 10 mol%), K_3PO_4 (9.0 mmol, 3.0 equiv), THF (degassed, 7.0 mL), H_2O (degassed, 1.0 mL) and arylbromide **16** (3.0 mmol, 1.2 equiv). The solution was degassed for 30 minutes and then stirred for overnight at 70 °C before cooling to ambient temperature. H_2O (20 mL) and Et_2O (20 mL) were added to the mixture. The organic layer was separated, and the aqueous layer was extracted with Et_2O (3 x 20 mL). The combined organic layers were dried over anhydrous $MgSO_4$, filtered and concentrated under reduced pressure. Purification of the yellow oil residue by silica gel chromatography (pentane/ethyl acetate, 9:1 to 3:1) afforded *cis*-tamoxifen as a white solid (790.1 mg, 71%, >20:1 *Z*/*E*).

2.8.11 Synthesis of diethylstilbestrol precursor 18

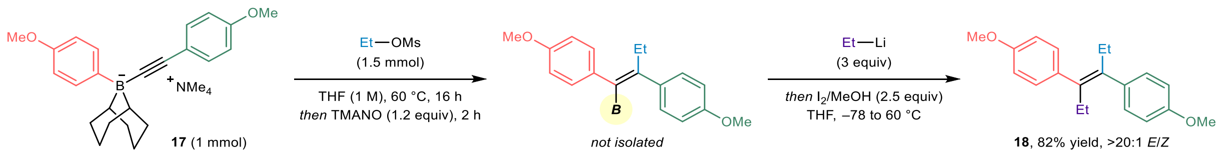

A 25 mL flame-dried Schlenk tube was sequentially added **17** (1.0 mmol, 1.0 equiv), anhydrous THF (1.0 mL) and ethyl mesylate (1.5 mmol, 1.5 equiv) under nitrogen. The reaction mixture was allowed to stir at 60 ºC for 16 hours before cooled to 0 ºC. Then, trimethylammonium *N*-oxide (3.6 mmol, 1.2 equiv) in DCM (3.0 mL) was added and the reaction mixture was stirred at ambient temperature for 3 hours to oxidise alkenyl borane to more stable alkenyl borinic ester. All solvents were evaporated and re-dissolved in *ca.* 10 mL of anhydrous diethyl ether. The solution was filtered through a celite pad to remove all solid, washed with anhydrous diethyl ether (3 x 10 mL) and the filtrate was concentrated under reduced pressure. The resulting crude material was dissolved in anhydrous THF (5.0 mL) and transferred to a flame-dried 25 mL Schlenk flask equipped with a magnetic stir bar under nitrogen.

The solution was cooled to 0 °C before dropwise addition of ethyllithium (1.3 M in diethyl ether, 3.0 mmol, 3.0 equiv). The reaction was stirred at this temperature for 30 minutes then cooled to -78 °C followed by slow addition I_2 (2.5 mmol, 2.5 equiv) in MeOH (4.0 mL). Then the solution was allowed to warm to 60 °C and stirred for further 12 hours. Quenched the reaction by adding a saturated aqueous solution of $Na_2S_2O_3$ (5.0 mL) at ambient temperature. H_2O (20 mL) and Et_2O (20 mL) were added to the mixture, and the aqueous layer was extracted with Et_2O (3 x 20 mL). The combined organic layers were dried over anhydrous $MgSO_4$, filtered and concentrated under reduced pressure. Purification of the yellow solid residue by silica gel chromatography (pentane/ethyl acetate, 20:1 to 9:1) afforded **18** as a colorless oil (242.7 mg, 82%, >20:1 *Z*/*E*).

2.8.12 Synthesis of (*E*)- and (*Z*)-γ-bisabolene

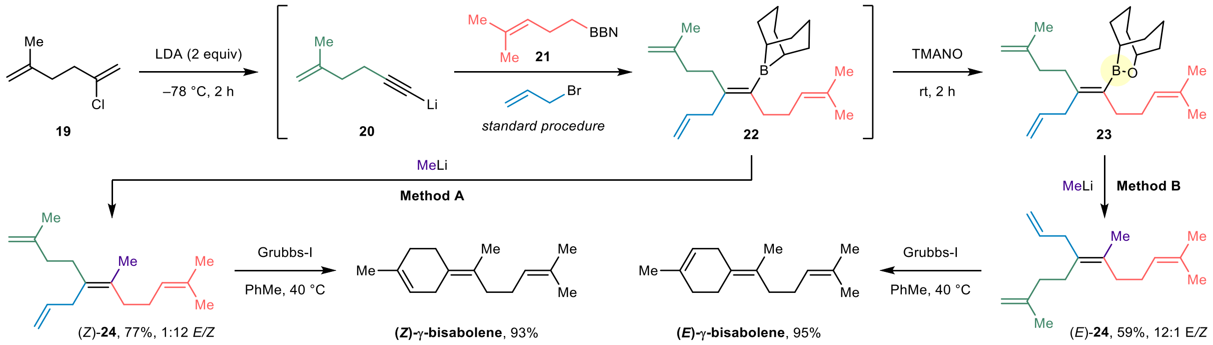

Synthesis of (*Z*)-24: To a LDA (1.0 mmol, 2.0 equiv, prepared in situ from 1.0 mmol of diisopropylamine and 1.0 mmol of *n*-BuLi) solution in THF (3.0 mL) was slowly added chlorodiene **19**[14] (0.5 mmol, 1.0 equiv) in THF (1.0 mL) under nitrogen at 0 °C. The solution was stirred at 0 °C for 30 minutes and further 1.5 hours at ambient temperature to complete the formation of alkynyllithium **20**. The borane **21** was then added dropwise to the reaction mixture at 0 °C. After stirring at 0 °C for 1 hour, allylbromide (1.0 mmol, 2.0 equiv) was added and the reaction was allowed to warm to 60 °C and stirred for further 10 hours at which time all volatile materials were carefully removed under reduced pressure to give crude trisubstituted alkenyl borane **22**, which was then re-dissolved in anhydrous THF and used for the Zweifel reaction.

Methyllithium (1.3 M in diethyl ether, 1.5 mmol. 3.0 equiv) was added dropwise to the THF solution of **22** at -78 °C under nitrogen. Then reaction mixture was stirred for 1 hour at this temperature followed by dropwise addition of I_2 (1.25 mmol, 2.5 equiv) in MeOH (2 mL). Then the solution was allowed to warm to 60 °C and stirred for further 6 hours. Quenched the reaction by adding a saturated aqueous solution of $Na_2S_2O_3$ (5 mL) at ambient temperature.

H_2O (20 mL) and hexane (20 mL) were added to the mixture, and the aqueous layer was extracted with hexane (3 x 20 mL). The combined organic layers were dried over anhydrous $MgSO_4$, filtered and concentrated in vacuo. Purification of the yellow solid residue by silica gel chromatography (pentane) afforded (*Z*)-**24** as a colorless oil (89.4 mg, 77%, 12:1 *Z*/*E*).

Synthesis of (*Z*)-γ-bisabolene: Prepared following a known procedure[15] with modifications. To a flamed-dried 25 mL Schlenk flask was added tetrasubstituted alkene (*Z*)-**24** (46.4 mg, 0.2 mmol, 1.0 equiv), anhydrous toluene (15.0 mL) and Grubb 1st catalyst [$Cl_2(PCy_3)_2$ Ru=CHPh] (0.02 mmol, 10 mol%) in sequence. The resulting dark purple solution was stirred under argon for 24 hours at 40 °C, and then exposed to air for 5 hours to destroy the catalyst. Evaporate solvents in vacuo and the residue was purified by silica gel chromatography (pentane) to give (*Z*)-γ-bisabolene as a colorless oil (37.9 mg, 93%, 12:1 *Z*/*E*)

Synthesis of (*E*)-24: To a LDA (1.0 mmol, 2.0 equiv, prepared in situ from 1.0 mmol of diisopropylamine and 1.0 mmol of *n*-BuLi) solution in THF (3.0 mL) was slowly added chlorodiene **19** (0.5 mmol, 1.0 equiv) in THF (1.0 mL) under nitrogen at 0 °C. The solution was stirred at 0 °C for 30 minutes and further 1.5 hours to complete the formation of alkynyllithium **20**. The borane **21** was then added dropwise to the reaction mixture at 0 °C. After stirring at 0 °C for 1 hour, allylbromide (1.0 mmol, 2.0 equiv) and trimethylammonium *N*-oxide (0.6 mmol, 1.2 equiv) was added and the reaction was allowed to warm to 60 °C and stirred for further 10 hours at which time all volatile materials were carefully removed under reduced pressure. The crude alkenyl borinic ester **23** was re-dissolved in *ca.* 10 mL of anhydrous diethyl ether and filtered through a celite pad to remove all solid, washed with anhydrous diethyl ether (3 x 10 mL) and the filtrate was concentrated under reduced pressure. The resulting crude material was used directly for the next step without further purification.

Methyllithium (1.3 M in diethyl ether, 0.75 mmol. 1.5 equiv) was added dropwise to the THF solution of **23** at -78 °C under nitrogen. The mixture was then allowed to stir at 0 °C for 1 hour before cooling to -78 °C. A solution of PhSeCl (0.60 mmol, 1.2 equiv) in THF (0.5 mL) was added dropwise. The resulting solution was stirred at -78 °C for 1 hour and then warmed to room temperature and stirred for further 2 hours and then cooled to -78 °C. A solution of sodium methoxide (0.5 M in MeOH, 5.0 equiv) was added dropwise, and the resulting solution was stirred at -78 °C for 1 hour and a further 2 hours at 0 °C. The reaction was quenched by an addition of a saturated aqueous solution of $Na_2S_2O_3$ (3.0 mL). H_2O (20 mL) and hexane (20 mL) were added to the mixture, the organic layer was separated, and the aqueous layer was

extracted with hexane (3 x 20 mL). The combined organic layers were dried over anhydrous $MgSO_4$, filtered and concentrated under reduced pressure. Purification of the yellow solid residue by silica gel chromatography (pentane) afforded (*E*)-**24** as a colorless oil (68.4 mg, 59%, 12:1 *E*/*Z*).

Synthesis of (*E*)-γ-bisabolene: Prepared following a known procedure[15] with modifications. To a flamed-dried 25 mL Schlenk flask was added tetrasubstituted alkene (*E*)-**24** (46.4 mg, 0.2 mmol, 1.0 equiv), anhydrous toluene (15.0 mL) and Grubb 1st catalyst [$Cl_2(PCy_3)_2$ Ru=CHPh] (0.02 mmol, 10 mol%) in sequence. The resulting dark purple solution was stirred under argon for 24 hours at 40 °C, and then exposed to air for 5 hours to destroy the catalyst. Evaporate solvents in vacuo and the residue was purified by silica gel chromatography (pentane) to give (*Z*)-γ-bisabolene as a colorless oil (38.8 mg, 95%, 12:1 *E*/*Z*)

2.9. Characterization Data for Products.

(*E*)-10-(5-ethyl-1-phenyloct-4-en-4-yl)-9-oxa-10-borabicyclo[3.3.2]decane (5a)

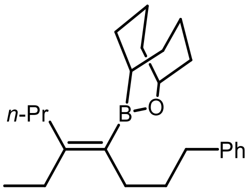

Prepared following **General Procedure A**. Purification by flash column chromatography (aluminum oxide, neutral for chromatography, 5-8% DCM in pentane) gave the title compound (139.0 mg, 79%, >20:1 *E*/*Z*).

R*f* = 0.60 (20:1 pentane: EtOAc, CAM)

^{1}H NMR (500 MHz, CD_2Cl_2) δ 7.28 – 7.25 (m, 2H), 7.20 – 7.14 (m, 3H), 4.58 (tt, *J* = 5.5, 3.0 Hz, 1H), 2.62 (t, *J* = 7.5 Hz, 2H), 2.13 – 2.07 (m, 4H), 2.04 (q, *J* = 8.0 Hz, 2H), 1.91 – 1.82 (m, 4H), 1.73 – 1.60 (m, 7H), 1.51 – 1.38 (m, 6H), 0.95 (t, *J* = 8.0 Hz, 3H), 0.91 (d, *J* = 7.5 Hz, 3H).

^{13}C NMR (126 MHz, CD_2Cl_2) δ 146.5, 143.4, 128.8, 128.5, 125.9, 73.8, 38.3, 36.7, 33.2, 32.1, 30.3, 26.8, 23.9, 23.5, 22.8, 14.6, 13.8.

HRMS (ESI) m/z calculated for $C_{24}H_{37}BONa$ $[M+Na]^+$, 375.2830, found: 375.2836.

(*E*)-10-(5-methyl-1-phenyloct-4-en-4-yl)-9-oxa-10-borabicyclo[3.3.2]decane (5b)

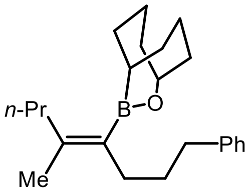

Prepared following **General Procedure A**. Purification by flash column chromatography (aluminum oxide, neutral for chromatography, 5-8% DCM in pentane) gave the title compound (130.1 mg, 77%, >20:1 *E*/*Z*).

R*f* = 0.58 (20:1 pentane: EtOAc, CAM)

^{1}H NMR (400 MHz, CD_2Cl_2) ^{1}H NMR (400 MHz, CD_2Cl_2) δ 7.30 – 7.25 (m, 2H), 7.22 – 7.15 (m, 3H), 4.59 (tt, *J* = 5.6, 3.2 Hz, 1H), 2.63 (t, *J* = 7.6 Hz, 2H), 2.15 – 2.09 (m, 4H), 1.92 – 1.82 (m, 4H), 1.75 – 1.60 (m, 7H), 1.64 (s, 3H), 1.54 – 1.40 (m, 6H), 0.91 (t, *J* = 7.2 Hz, 3H).

^{13}**C NMR** (101 MHz, CD_2Cl_2) δ 143.5, 141.1, 128.8, 128.5, 125.9, 73.7, 41.5, 36.6, 32.5, 32.2, 30.9, 26.8, 22.9, 22.8, 17.5, 14.4.

HRMS (ESI) m/z calculated for $C_{23}H_{35}BONa$ $[M+Na]^+$, 361.2673, found: 361.2670.

10-(1-phenyl-5-propyloct-4-en-4-yl)-9-oxa-10-borabicyclo[3.3.2]decane (5c)

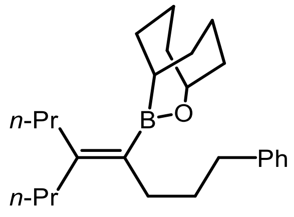

Prepared following **General Procedure A**. Purification by flash column chromatography (aluminum oxide, neutral for chromatography, 5-8% DCM in pentane) gave the title compound (135.5 mg, 74%).

$\mathbf{R}_f$ = 0.60 (20:1 pentane: EtOAc, CAM)

^{1}**H NMR** (400 MHz, CD_2Cl_2) δ 7.28 – 7.24 (m, 2H), 7.19 – 7.15 (m, 3H), 4.57 (tt, *J* = 5.6, 3.2 Hz, 1H), 2.61 (t, *J* = 7.6 Hz, 2H), 2.14 – 2.04 (m, 4H), 2.00 – 1.93 (m, 2H), 1.89 – 1.81 (m, 4H), 1.71 – 1.58 (m, 8H), 1.47 – 1.34 (m, 8H), 0.89 – 0.86 (m, 6H).

^{13}**C NMR** (101 MHz, CD_2Cl_2) δ 144.9, 143.4, 128.8, 128.5, 125.8, 73.7, 38.8, 36.7, 33.3, 33.1, 32.1, 30.4, 26.8, 23.5, 22.8, 22.6, 17.7, 14.7, 14.5.

HRMS (ESI) m/z calculated for $C_{25}H_{39}BONa$ $[M+Na]^+$, 389.2986, found: 389.2986.

(*Z*)-10-(5-phenethyl-1-phenyloct-4-en-4-yl)-9-oxa-10-borabicyclo[3.3.2]decane (5d)

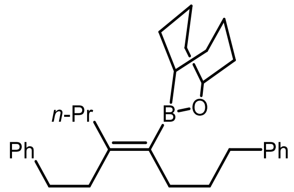

Prepared following **General Procedure A**. Purification by preparative TLC (8% ethyl acetate in pentane) gave the title compound (149.8 mg, 70%, >20:1 *Z*/*E*).

$\mathbf{R}_f$ = 0.52 (20:1 pentane: EtOAc, CAM)

^{1}**H NMR** (400 MHz, CD_2Cl_2) δ 7.29 – 7.24 (m, 4H), 7.19 – 7.14 (m, 6H), 4.56 (tt, *J* = 5.2, 3.2 Hz, 1H), 2.60 – 2.54 (m, 4H), 2.09 – 1.99 (m, 6H), 1.88 – 1.79 (m, 4H), 1.70 – 1.54 (m, 8H), 1.48 – 1.35 (m, 6H), 0.87 (t, *J* = 7.2 Hz, 3H).

^{13}C NMR (101 MHz, CD_2Cl_2) δ 144.6, 143.4, 143.3, 128.85, 128.79, 128.6, 128.5, 125.94, 125.87, 73.8, 38.8, 36.7, 33.2, 32.1, 31.3, 30.8, 30.4, 26.8, 23.5, 22.8, 14.5.

HRMS (ESI) m/z calculated for $C_{30}H_{41}BONa$ $[M+Na]^+$, 451.3143, found: 451.3149

(*Z*)-10-(1-phenyl-5-propyldec-4-en-9-yn-4-yl)-9-oxa-10-borabicyclo[3.3.2]decane (5e)

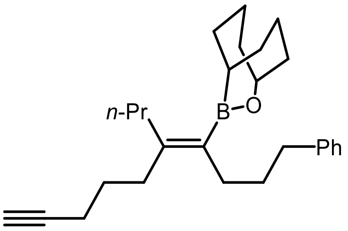

Prepared following **General Procedure A**. Purification by preparative TLC (10% ethyl acetate in pentane) gave the title compound (107.3 mg, 55%, >20:1 *Z*/*E*).

R_f = 0.50 (20:1 pentane: EtOAc, CAM)

^{1}H NMR (400 MHz, CD_2Cl_2) δ 7.28 – 7.23 (m, 2H), 7.19 – 7.13 (m, 3H), 4.57 (tt, *J* = 5.6, 3.2 Hz, 1H), 2.61 (t, *J* = 7.6 Hz, 2H), 2.15 – 2.04 (m, 8H), 1.98 (t, *J* = 2.8 Hz, 1H), 1.89 – 1.80 (m, 4H), 1.71 – 1.53 (m, 9H), 1.49 – 1.38 (m, 6H), 0.88 (t, *J* = 7.2 Hz, 3H)..

^{13}C NMR (101 MHz, CD_2Cl_2) δ 143.6, 143.3, 128.8, 128.5, 125.9, 85.1, 73.8, 68.5, 38.7, 36.6, 33.1, 32.1, 30.4, 30.2, 28.3, 26.8, 23.4, 22.7, 19.0, 14.5.

HRMS (ESI) m/z calculated for $C_{27}H_{39}BONa$ $[M+Na]^+$, 413.2986, found: 413.2978

(*Z*)-10-(1-phenyl-5-propyldec-4-en-9-yn-4-yl)-9-oxa-10-borabicyclo[3.3.2]decane (5f)

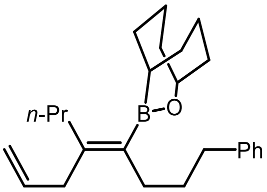

Prepared following **General Procedure A**. Purification by preparative TLC (8% ethyl acetate in pentane) gave the title compound (123.9 mg, 68%, >20:1 *Z*/*E*).

R_f = 0.60 (20:1 pentane: EtOAc, CAM)

^{1}H NMR (400 MHz, CD_2Cl_2) ^{1}H NMR (400 MHz, CD_2Cl_2) δ 7.28 – 7.23 (m, 2H), 7.18 – 7.13 (m, 3H), 5.75 (ddt, *J* = 16.8, 10.0, 6.4 Hz, 1H), 5.00 – 4.92 (m, 2H), 4.57 (tt, *J* = 5.2, 3.2 Hz,

1H), 2.78 (d, J = 6.4 Hz, 2H), 2.60 (t, J = 7.6 Hz, 2H), 2.13 – 2.04 (m, 4H), 1.90 – 1.80 (m, 4H), 1.72 – 1.60 (m, 7H), 1.51 – 1.38 (m, 6H), 0.88 (t, J = 7.2 Hz, 3H).

^{13}C NMR (101 MHz, CD_2Cl_2) δ 143.3, 141.6, 137.7, 128.8 128.5, 125.9, 114.9, 73.9, 38.8, 36.7, 35.8, 32.9, 32.1, 30.5, 26.8, 23.2, 22.8, 14.4.

HRMS (ESI) m/z calculated for $C_{25}H_{37}BONa$ [M+Na]$^+$, 387.2830, found: 387.2822

(*Z*)-10-(1-phenyl-5-propyldec-4-en-9-yn-4-yl)-9-oxa-10-borabicyclo[3.3.2]decane (5g)

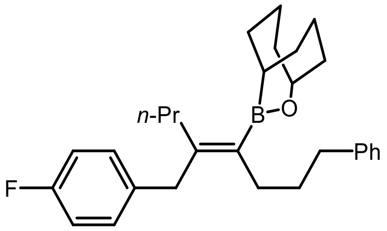

Prepared following **General Procedure A**. Purification by preparative TLC (8% ethyl acetate in pentane) gave the title compound (166.4 mg, 77%, 19:1 *Z*/*E*).

R*f* = 0.60 (20:1 pentane: EtOAc, CAM)

^{1}H NMR (400 MHz, CD_2Cl_2) δ 7.31 – 7.22 (m, 2H), 7.21 – 7.06 (m, 5H), 6.98 – 6.93 (m, 2H), 4.61 (tt, J = 5.2, 3.2 Hz, 1H), 3.38 (s, 2H), 2.61 (t, J = 7.6 Hz, 2H), 2.24 – 2.15 (m, 2H), 2.05 – 1.95 (m, 2H), 1.95 – 1.82 (m, 4H), 1.79 – 1.61 (m, 7H), 1.55 – 1.44 (m, 4H), 1.44 – 1.34 (m, 2H), 0.85 (t, J = 7.2 Hz, 3H).

^{13}C NMR (101 MHz, CD_2Cl_2) δ 160.8 (J_{CF} = 243.0 Hz), 142.8, 141.3, 136.7 (J_{CF} = 3.2 Hz), 129.9 (J_{CF} = 7.7 Hz), 128.4, 128.2, 125.5, 114.7 (J_{CF} = 21.2 Hz), 73.6, 38.3, 36.2, 35.2, 32.4, 31.6, 30.5, 26.4, 22.9, 22.4, 14.0.

^{19}F NMR (377 MHz, CD_2Cl_2) δ -118.98.

HRMS (ESI) m/z calculated for $C_{29}H_{38}BFONa$ [M+Na]$^+$, 455.2892, found: 455.2899.

ethyl (*Z*)-4-(9-oxa-10-borabicyclo[3.3.2]decan-10-yl)-7-phenyl-3-propylhept-3-enoate (5h)

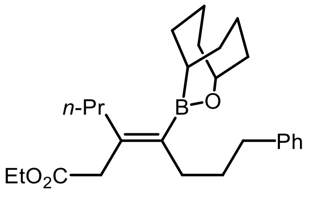

Prepared following **General Procedure A**. Purification by flash column chromatography (aluminum oxide, neutral for chromatography, 5-10% DCM in pentane) gave the title compound (121.0 mg, 59%, 19:1 *Z*/*E*).

R*f* = 0.55 (20:1 pentane: EtOAc, CAM)

^{1}H NMR (400 MHz, CD_2Cl_2) δ 7.27 – 7.23 (m, 2H), 7.20 – 7.14 (m, 3H), 4.59 (tt, *J* = 5.6, 3.2 Hz, 1H), 4.08 (q, *J* = 7.2 Hz, 2H), 3.03 (s, 2H), 2.60 (t, *J* = 7.6 Hz, 2H), 2.18 – 2.04 (m, 4H), 1.89 – 1.80 (m, 4H), 1.71 – 1.58 (m, 7H), 1.48 – 1.38 (m, 6H), 1.23 (t, *J* = 7.2 Hz, 3H), 0.88 (t, *J* = 7.2 Hz, 3H).

^{13}C NMR (101 MHz, CD_2Cl_2) δ 172.3, 143.2, 136.2, 128.8, 128.5, 125.9, 74.0, 60.7, 39.6, 36.7, 36.5, 32.3, 32.0, 30.9, 26.8, 22.8, 22.7, 14.4, 14.3.

HRMS (ESI) m/z calculated for $C_{26}H_{39}BO_3Na$ $[M+Na]^+$, 433.2884, found: 433.2880.

<u>(*Z*)-10-(5-((methylthio)methyl)-1-phenyloct-4-en-4-yl)-9-oxa-10-borabicyclo[3.3.2]decane (5i)</u>

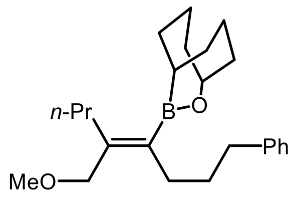

Prepared following **General Procedure A**. Purification by flash column chromatography (aluminum oxide, neutral for chromatography, 5-10% DCM in pentane) gave the title compound (127.0 mg, 69%, >20:1 *Z*/*E*).

R*f* = 0.50 (20:1 pentane: EtOAc, CAM)

^{1}H NMR (400 MHz, CD_2Cl_2) δ 7.29 – 7.24 (m, 2H), 7. 19 – 7.14 (m, 3H), 4.59 (tt, *J* = 5.6, 3.2 Hz, 1H), 3.84 (s, 2H), 3.22 (s, 3H), 2.61 (t, *J* = 7.6 Hz, 2H), 2.17 – 2.07 (m, 4H), 1.90 – 1.83 (m, 4H), 1.71 – 1.58 (m, 7H), 1.50 – 1.39 (m, 6H), 0.89 (t, *J* = 7.6 Hz, 3H).

^{13}C NMR (101 MHz, CD_2Cl_2) δ 143.2, 140.0, 128.8, 128.6, 125.9, 74.0, 70.3, 58.0, 37.4, 36.5, 33.0, 32.0, 30.0, 26.8, 23.3, 22.7, 14.5.

HRMS (ESI) m/z calculated for $C_{24}H_{37}BO_2Na$ $[M+Na]^+$, 391.2779, found: 391.2781.

<u>(*Z*)-10-(5-((methylthio)methyl)-1-phenyloct-4-en-4-yl)-9-oxa-10-borabicyclo[3.3.2]decane (5j)</u>

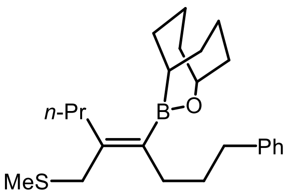

Prepared following **General Procedure A**. Purification by preparative TLC (10% EtOAc in pentane) gave the title compound (121.1 mg, 63%, >20:1 *Z*/*E*).

R*f* = 0.53 (20:1 pentane: EtOAc, CAM)

^{1}H NMR (400 MHz, CD_2Cl_2) δ 7.29 – 7.24 (m, 2H), 7.20 – 7.14 (m, 3H), 4.58 (tt, *J* = 5.6, 3.2 Hz, 1H), 3.13 (s, 2H), 2.62 (t, *J* = 7.6 Hz, 2H), 2.20 – 2.12 (m, 4H), 1.98 (s, 3H), 1.90 – 1.81 (m, 4H), 1.72 – 1.60 (m, 7H), 1.51 – 1.40 (m, 6H), 0.90 (t, *J* = 7.2 Hz, 3H).

^{13}C NMR (101 MHz, CD_2Cl_2) δ 143.2, 138.6, 128.8, 128.6, 125.9, 74.0, 37.6, 36.5, 33.8, 32.8, 32.0, 30.5, 26.8, 23.4, 22.7, 15.4, 14.4.

HRMS (ESI) m/z calculated for $C_{24}H_{37}BSONa$ $[M+Na]^+$, 407.2550, found: 407.2552.

(*E*)-10-(1-phenyl-5-((4,4,5,5-tetramethyl-1,3,2-dioxaborolan-2-yl)methyl)oct-4-en-4-yl)-9-oxa-10-borabicyclo[3.3.2]decane (5k)

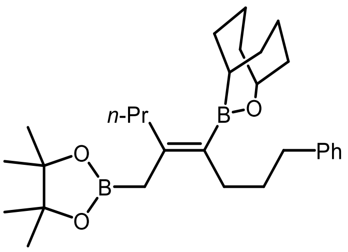

Prepared following **General Procedure A**. Purification by preparative TLC (8% EtOAc in pentane) gave the title compound (97.5 mg, 42%, 18:1 *E*/*Z*).

R*f* = 0.55 (20:1 pentane: EtOAc, CAM)

^{1}H NMR (400 MHz, C_6D_6) δ 7.24 – 7.19 (m, 3H), 7.14 – 7.06 (m, 2H), 4.48 (tt, *J* = 5.6, 2.8 Hz, 1H), 2.72 (t, *J* = 7.6 Hz, 2H), 2.51 (t, *J* = 8.0 Hz, 2H), 2.45 (t, *J* = 8.0 Hz, 2H), 1.98 – 1.84 (m, 4H), 1.74 – 1.66 (m, 7H), 1.45 – 1.34 (m, 6H), 1.06 – 1.02 (m, 17H).

^{13}C NMR (101 MHz, C_6D_6) δ 143.5, 142.8, 128.9, 128.5, 125.8, 73.1, 41.3, 36.9, 32.9, 32.0, 31.8, 26.9, 25.0, 24.9, 23.2, 22.8, 14.7.

HRMS (ESI) m/z calculated for $C_{29}H_{46}B_2O_3Na$ $[M+Na]^+$, 487.3525, found: 487.3526.

(*Z*)-10-(1-phenyl-5-propylnona-4,8-dien-4-yl)-9-oxa-10-borabicyclo[3.3.2]decane (5l)

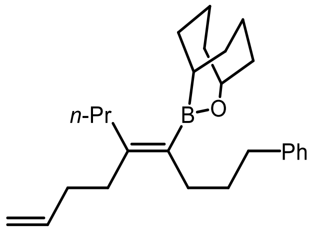

Prepared following **General Procedure A**. Purification by flash column chromatography (aluminum oxide, neutral for chromatography, 5-10% DCM in pentane) gave the title compound (128.5 mg, 68%, >20:1 *Z*/*E*).

R*f* = 0.60 (20:1 pentane: EtOAc, CAM)

^{1}H NMR (400 MHz, CD_2Cl_2) δ 7.24 – 7.14 (m, 2H), 7.19 – 7.13 (m, 3H), 5.82 (ddt, *J* = 16.4, 10.0, 3.2 Hz, 1H), 5.02 – 4.91 (m, 2H), 4.57 (tt, *J* = 5.6, 3.2 Hz, 1H), 2.61 (t, *J* = 7.6 Hz, 2H), 2.12 – 2.05 (m, 8H), 1.90 – 1.82 (m, 5H), 1.71 – 1.58 (m, 8H), 1.51 – 1.38 (m, 7H), 0.89 (t, *J* = 7.2 Hz, 3H).

^{13}C NMR (101 MHz, CD_2Cl_2) δ 144.0, 143.3, 139.6, 128.8, 128.54, 128.49, 125.9, 114.1, 73.8, 38.7, 36.7, 33.6, 33.1, 32.1, 30.6, 30.4, 26.8, 23.5, 22.8, 14.5.

HRMS (ESI) m/z calculated for $C_{26}H_{39}BONa$ $[M+Na]^+$, 401.2986, found: 401.2995.

(*Z*)-10-(1-phenyl-5-propylnon-4-en-8-yn-4-yl)-9-oxa-10-borabicyclo[3.3.2]decane (5m)

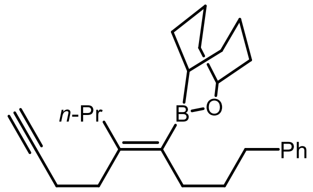

Prepared following **General Procedure A**. Purification by flash column chromatography (aluminum oxide, neutral for chromatography, 5-10% DCM in pentane) gave the title compound (120.4 mg, 64%, >20:1 *Z*/*E*).

R*f* = 0.53 (20:1 pentane: EtOAc, CAM)

^{1}H NMR (400 MHz, CD_2Cl_2) δ 7.28 – 7.24 (m, 2H), 7.20 – 7.13 (m, 3H), 4.57 (tt, *J* = 5.6, 3.2 Hz, 1H), 2.61 (t, *J* = 7.6 Hz, 2H), 2.30 – 2.22 (m, 2H), 2.21 – 2.18 (m, 2H), 2.15 – 2.02 (m, 4H), 1.98 (t, *J* = 2.4 Hz, 1H), 1.88 – 1.80 (m, 4H), 1.72 – 1.56 (m, 7H), 1.50 – 1.34 (m, 6H), 0.89 (t, *J* = 7.2 Hz, 3H).

^{13}C NMR (101 MHz, CD_2Cl_2) δ 143.5, 142.1, 128.8, 128.6, 125.9, 85.1, 73.9, 68.2, 38.4, 36.6, 33.0, 32.0, 30.4, 30.1, 26.8, 23.4, 22.7, 14.4.

HRMS (ESI) m/z calculated for $C_{26}H_{38}BO$ $[M+H]^+$, 377.3010, found: 377.3011.

(*S*,*Z*)-10-(5-(oxiran-2-ylmethyl)-1-phenyloct-4-en-4-yl)-9-oxa-10-borabicyclo[3.3.2]decane (5n)

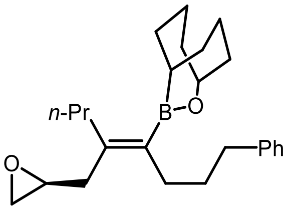

Prepared following **General Procedure A**. Purification by preparative TLC (15% EtOAc in pentane) gave the title compound (60.9 mg, 32%, >20:1 *Z*/*E*).

R*f* = 0.45 (20:1 pentane: EtOAc, CAM)

^{1}H NMR (400 MHz, CD_2Cl_2) δ 7.28 – 7.23 (m, 2H), 7.18 – 7.13 (m, 3H), 4.61 – 4.55 (m, 1H), 2.84 (tt, *J* = 5.6, 2.8 Hz, 1H), 2.68 (t, *J* = 4.4 Hz, 1H), 2.61 (t, *J* = 7.6 Hz, 2H), 2.41 (dd, *J* = 5.2, 2.8 Hz, 1H), 2.35 (dd, *J* = 14.0, 5.2 Hz, 1H), 2.16 – 2.09 (m, 5H), 1.90 – 1.80 (m, 4H), 1.73 – 1.59 (m, 7H), 1.50 – 1.39 (m, 6H), 0.88 (t, *J* = 7.2 Hz, 3H).

^{13}C NMR (101 MHz, CD_2Cl_2) δ 143.2, 139.3, 128.8, 128.6, 125.9, 74.0, 52.0, 47.6, 39.6, 36.6, 34.0, 32.9, 32.1, 32.0, 30.6, 26.8, 23.3, 22.8, 22.7, 14.4.

Specific rotation $[\alpha]_D^{22}$ = 170.6 (c = 0.34, CH_2Cl_2)

HRMS (ESI) m/z calculated for $C_{25}H_{37}BO_2Na$ $[M+Na]^+$, 403.2779, found: 403.2789.

(*R*,*Z*)-10-(5-(2-(2,2-dimethyl-1,3-dioxolan-4-yl)ethyl)-1-phenyloct-4-en-4-yl)-9-oxa-10-borabicyclo[3.3.2]decane (5o)

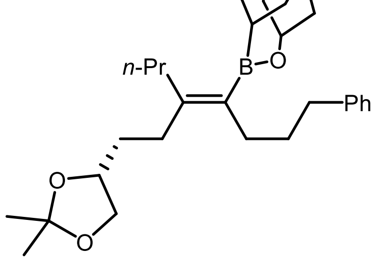

Prepared following **General Procedure A**. Purification by preparative TLC (10% EtOAc in pentane) gave the title compound (144.6 mg, 64%, 18:1 *Z*/*E*).

R*f* = 0.40 (20:1 pentane: EtOAc, CAM)

^{1}H NMR (400 MHz, CD_2Cl_2) δ 7.28 – 7.24 (m, 2H), 7.19 – 7.15 (m, 3H), 4.57 (tt, *J* = 5.6, 3.2 Hz, 1H), 4.05 – 3.87 (m, 2H), 3.50 – 3.36 (m, 1H), 2.61 (t, *J* = 7.6 Hz, 2H), 2.10 – 2.04 (m, 5H), 2.00 – 1.92 (m, 1H), 1.87 – 1.79 (m, 4H), 1.72 – 1.59 (m, 8H), 1.54 – 1.20 (m, 9H), 1.37 (s, 3H), 1.31 (s, 3H), 0.90 (t, *J* = 6.8 Hz, 3H).

^{13}C NMR (101 MHz, CD_2Cl_2) δ 144.0, 143.3, 128.8, 128.5, 125.9, 108.8, 76.5, 73.8, 69.8, 36.6, 36.4, 33.5, 33.0, 32.6, 32.1, 32.0, 30.3, 27.2, 27.1, 26.8, 25.9, 23.6, 22.80, 22.78, 14.4.

Specific rotation $[\alpha]_D^{22}$ = 10.9 (c = 0.55, CH_2Cl_2)

HRMS (ESI) m/z calculated for $C_{29}H_{45}BO_3Na$ $[M+Na]^+$, 475.3354, found: 475.3358.

<u>(*S*,*Z*)-10-(8,12-dimethyl-1-phenyl-5-propyltrideca-4,11-dien-4-yl)-9-oxa-10-borabicyclo[3.3.2]decane (5p)</u>

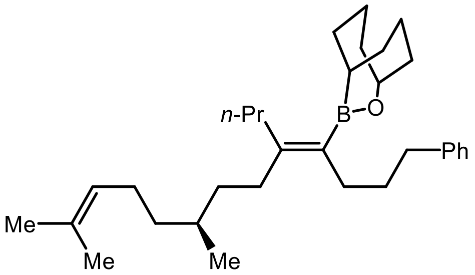

Prepared following **General Procedure A**. Purification by preparative TLC (10% EtOAc in pentane) gave the title compound (141.0 mg, 61%, 20:1 *Z*/*E*).

R*f* = 0.40 (20:1 pentane: EtOAc, CAM)

^{1}H NMR (400 MHz, CD_2Cl_2) δ 7.27 – 7.23 (m, 2H), 7.18 – 7.12 (m, 3H), 5.12 (t, *J* = 7.2 Hz, 1H), 4.57 (tt, *J* = 5.2, 3.2 Hz, 1H), 2.61 (t, *J* = 7.6 Hz, 2H), 2.11 – 2.04 (m, 4H), 2.03 – 1.91 (m, 4H), 1.90 – 1.79 (m, 4H), 1.73 – 1.57 (m, 7H), 1.69 (s, 3H), 1.61 (s, 3H), 1.51 – 1.26 (m, 9H), 1.19 – 1.09 (m, 2H), 0.90 – 0.86 (m, 6H).

^{13}C NMR (101 MHz, CD_2Cl_2) δ 145.6, 143.4, 131.3, 128.8, 128.5, 125.9, 125.4, 73.8, 38.8, 37.4, 36.7, 36.6, 33.5, 33.2, 32.1, 30.4, 28.7, 26.8, 26.0, 25.8, 23.5, 22.8, 19.8, 17.8, 14.5.

Specific rotation $[\alpha]_D^{22}$ = 10.0 (c = 0.40, CH_2Cl_2)

HRMS (ESI) m/z calculated for $C_{32}H_{51}BONa$ $[M+Na]^+$, 485.3925, found: 485.3922.

(*E*)-10-(1,2-diphenylbut-1-en-1-yl)-9-oxa-10-borabicyclo[3.3.2]decane (5q)

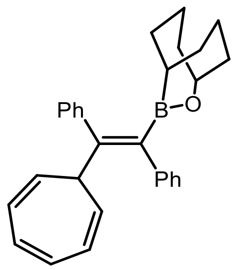

Prepared following **General Procedure B**. Purification by flash column chromatography (aluminum oxide, neutral for chromatography, 5-15% DCM in pentane) gave the title compound (69.9 mg, 86%, 19:1 *E*/*Z*).

R_f = 0.42 (20:1 pentane: EtOAc, CAM)

^{1}H NMR (400 MHz, $CDCl_3$) δ 7.50 – 7.44 (m, 2H), 7.39 – 7.33 (m, 2H), 7.33 – 7.29 (m, 1H), 7.26 – 7.19 (m, 2H), 7.20 – 7.09 (m, 3H), 6.46-6.40 (m, 2H), 6.13 – 5.96 (m, 2H), 5.25 (dd, *J* = 9.2, 5.6 Hz, 2H), 4.45 (tt, *J* = 5.2, 3.2 Hz, 1H), 2.62 – 2.49 (m, 1H), 1.60 – 1.45 (m, 7H), 1.14 – 1.04 (m, 4H), 1.00 – 0.90 (m, 2H).

^{13}C NMR (101 MHz, $CDCl_3$) δ 145.0, 142.7, 141.3, 130.4, 130.3, 128.4, 128.0, 127.9, 127.2, 125.8, 124.4, 123.5, 73.9, 42.7, 31.3, 26.6, 22.0.

HRMS (ESI) m/z calculated for $C_{29}H_{32}BO$ $[M+H]^+$, 407.2541, found: 407.2543.

(*Z*)-10-(2-(benzo[d][1,3]dithiol-2-yl)-1,2-diphenylvinyl)-9-oxa-10-borabicyclo[3.3.2]decane (5r)

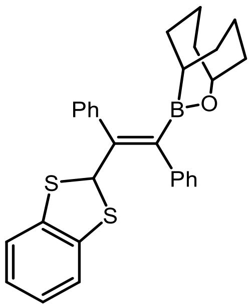

Prepared following **General Procedure B**. Purification by flash column chromatography (aluminum oxide, neutral for chromatography, 10% DCM in pentane) gave the title compound (80.6 mg, 86%, >20:1 *Z*/*E*).

R*f* = 0.52 (20:1 pentane: EtOAc, CAM)

^{1}H NMR (500 MHz, CD_2Cl_2) δ 7.50 (dd, *J* = 8.0, 1.5 Hz, 2H), 7.42 – 7.37 (m, 2H), 7.31 – 7.27 (m, 1H), 7.24 (dd, *J* = 8.0, 1.5 Hz, 2H), 7.21 – 7.16 (m, 3H), 6.92 (dd, *J* = 6.0, 3.0 Hz, 2H), 6.80 (dd, *J* = 6.0, 3.0 Hz, 2H), 6.11 (s, 1H), 4.40 (tt, *J* = 5.5, 3.0 Hz, 1H), 1.53 – 1.41 (m, 7H), 1.11 – 1.02 (m, 2H), 1.00 – 0.91 (m, 2H), 0.89 – 0.80 (m, 2H).

^{13}C NMR (126 MHz, CD_2Cl_2) δ 140.6, 140.0, 139.3, 138.6, 132.0, 128.9, 128.4, 127.7, 127.3, 126.9, 125.3, 121.8, 74.5, 54.9, 31.4, 26.5, 22.2.

HRMS (ESI) m/z calculated for $C_{29}H_{30}BS_2O$ $[M+H]^+$, 469.1826, found: 469.1824.

(*Z*)-3-(9-oxa-10-borabicyclo[3.3.2]decan-10-yl)-*N*,*N*-dimethyl-2,3-diphenylprop-2-en-1-amine (5s)

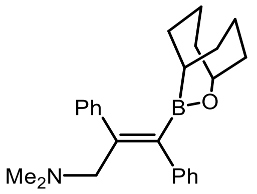

Prepared following **General Procedure B**. Purification by flash column chromatography (aluminum oxide, neutral for chromatography, 10% EtOAc in pentane) gave the title compound (41.1 mg, 55%, >20:1 *Z*/*E*).

R*f* = 0.28 (10:1 pentane: EtOAc, CAM)

^{1}H NMR (400 MHz, $CDCl_3$) δ 7.40 – 7.33 (m, 2H), 7.30 – 7.22 (m, 4H), 7.21 – 7.11 (m, 2H), 7.11 – 7.03 (m, 2H), 4.38 (tt, *J* = 5.2, 3.2 Hz, 1H), 3.09 (s, 2H), 1.97 (s, 6H), 1.61 – 1.36 (m, 6H), 1.30-1.27 (m, 1H), 1.14 – 0.98 (m, 4H), 0.96 – 0.84 (m, 2H).

^{13}C NMR (101 MHz, $CDCl_3$) δ 143.9, 142.3, 128.7, 128.5, 128.2, 128.0, 127.4, 125.7, 74.1, 61.1, 45.4, 31.3, 26.8, 22.0.

HRMS (ESI) m/z calculated for $C_{25}H_{33}BNO$ $[M+H]^+$, 374.2650, found: 374.2655.

(*E*)-10-(1,2-diphenylpenta-1,4-dien-1-yl)-9-oxa-10-borabicyclo[3.3.2]decane (5t)

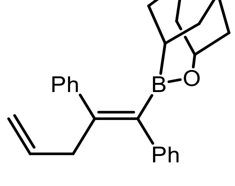

Prepared following **General Procedure B**. Purification by flash column chromatography (aluminum oxide, neutral for chromatography, 10% DCM in pentane) gave the title compound (67.7 mg, 95%, >20:1 *E*/*Z*).

R*f* = 0.45 (20:1 pentane: EtOAc, CAM)

^{1}H NMR (400 MHz, $CDCl_3$) δ 7.38 – 7.27 (m, 7H), 7.24 – 7.17 (m, 3H), 5.74 – 5.52 (m, 1H), 4.87 (dd, *J* = 4.0, 2.0 Hz, 1H), 4.84 (dd, *J* = 3.6, 2.0 Hz, 1H), 4.42 (tt, *J* = 5.6, 3.2 Hz, 1H), 3.06 (d, *J* = 6.6 Hz, 2H), 1.66 – 1.58 (m, 4H), 1.51-1.44 (m, 2H), 1.39 – 1.34 (m, 1H), 1.32 – 1.20 (m, 2H), 1.19 – 1.06 (m, 4H).

^{13}C NMR (101 MHz, $CDCl_3$) δ 145.1, 144.3, 143.2, 136.7, 129.1, 128.5, 128.2, 128.1, 127.2, 125.75, 115.6, 73.9, 39.3, 31.3, 29.8, 26.8, 22.1.

HRMS (ESI) m/z calculated for $C_{25}H_{30}BO$ $[M+H]^+$, 357.2384, found: 357.2383.

<u>(*E*)-10-(1,2-diphenyl-5-(phenylthio)pent-1-en-1-yl)-9-oxa-10-borabicyclo[3.3.2]decane (5u)</u>

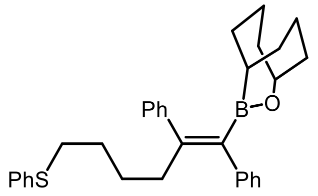

Prepared following **General Procedure B**. Purification by flash column chromatography (aluminum oxide, neutral for chromatography, 10% DCM in pentane) gave the title compound (59.5 mg, 62%, >20:1 *E*/*Z*).

R*f* = 0.40 (20:1 pentane: EtOAc, CAM)

^{1}H NMR (500 MHz, $CDCl_3$) δ 7.38 – 7.31 (m, 6H), 7.30 – 7.27 (m, 2H), 7.26 – 7.21 (m, 4H), 7.21 – 7.13 (m, 3H), 4.43 (tt, *J* = 6.0, 3.5 Hz, 1H), 2.68 (t, *J* = 7.5 Hz, 2H), 2.32 (t, *J* = 7.5 Hz, 2H), 1.66 – 1.61 (m, 4H), 1.53 – 1.43 (m, 4H), 1.37 – 1.32 (m, 3H), 1.26 – 1.17 (m, 2H), 1.16 – 1.10 (m, 2H), 1.07 – 0.98 (m, 2H).

^{13}C NMR (126 MHz, $CDCl_3$) δ 147.1, 144.3, 143.2, 137.0, 129.1, 128.89, 128.87, 128.5, 128.2, 127.1, 125.7, 125.6, 73.9, 33.6, 33.4, 31.3, 28.8, 27.8, 26.7, 22.1.

HRMS (ESI) m/z calculated for $C_{32}H_{38}BSO$ $[M+H]^+$, 481.2731, found: 481.2733.

<u>(*Z*)-10-(2-fluoro-1,2-diphenylvinyl)-9-oxa-10-borabicyclo[3.3.2]decane (5v)</u>

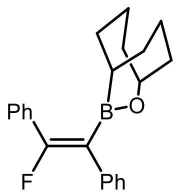

Prepared following **General Procedure B**. Purification by preparative TLC (5% EtOAc in pentane) gave the title compound (34.1 mg, 51%, >20:1 *Z*/*E*).

R*f* = 0.60 (20:1 pentane: EtOAc, CAM)

^{1}H NMR (400 MHz, CD_2Cl_2) δ 7.57 – 7.53 (m, 2H), 7.45 – 7.43 (m, 2H), 7.39 – 7.35 (m, 3H), 7.30 – 7.23 (m, 3H), 4.50 (tt, *J* = 5.6, 2.8 Hz, 1H), 1.87 – 1.78 (m, 4H), 1.66 – 1.60 (m, 2H), 1.50 – 1.36 (m, 2H), 1.38 – 1.26 (m, 5H).

^{13}C NMR (101 MHz, CD_2Cl_2) δ 161.2 (d, *J* = 266.1 Hz), 138.7 (d, *J* = 6.0 Hz), 134.8 (d, *J* = 31.7 Hz), 131.9, 130.1 (d, *J* = 1.5 Hz), 129.7 (d, *J* = 2.1 Hz), 129.0 (d, *J* = 4.2 Hz), 128.8, 128.5, 128.4, 126.5, 74.6, 31.6, 27.2, 22.6.

^{19}F NMR (377 MHz, CD_2Cl_2) δ -84.52

HRMS (ESI) m/z calculated for $C_{22}H_{25}BFO$ $[M+H]^+$, 335.1977, found: 335.1979.

<u>(*Z*)-10-(1,2-diphenyl-2-(phenylselanyl)vinyl)-9-oxa-10-borabicyclo[3.3.2]decane (5w)</u>

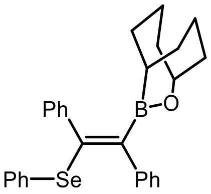

Prepared following **General Procedure B**. Purification by flash column chromatography (aluminum oxide, neutral for chromatography, 10% DCM in pentane) gave the title compound (59.5 mg, 63%, >20:1 *Z*/*E*).

R*f* = 0.55 (20:1 pentane: EtOAc, CAM)

^{1}H NMR (400 MHz, $CDCl_3$) δ 7.37 (dd, *J* = 7.6, 2.0 Hz, 2H), 7.21 – 7.02 (m, 10H), 6.99 – 6.88 (m, 3H), 4.65 (tt, *J* = 5.2, 3.2 Hz, 1H), 2.04 – 1.79 (m, 7H), 1.71 – 1.48 (m, 6H).

^{13}C NMR (101 MHz, $CDCl_3$) δ 142.2, 140.3, 134.6, 131.9, 131.6, 130.5, 128.9, 128.8, 128.1, 127.4, 126.7, 126.6, 126.1, 74.5, 31.4, 28.1, 22.4.

HRMS (ESI) m/z calculated for $C_{28}H_{30}BSeO$ $[M+H]^+$, 473.1549, found: 473.1540.

(*Z*)-10-(2-(methylthio)-1,2-diphenylvinyl)-9-oxa-10-borabicyclo[3.3.2]decane (5x)

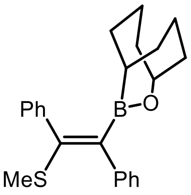

Prepared following **General Procedure B**. Purification by flash column chromatography (aluminum oxide, neutral for chromatography, 10% DCM in pentane) gave the title compound (59.4 mg, 82%, >20:1 *Z*/*E*).

R*f* = 0.55 (20:1 pentane: EtOAc, CAM)

^{1}H NMR (500 MHz, $CDCl_3$) δ 7.23 (dd, *J* = 8.0, 1.5 Hz, 2H), 7.16 – 7.10 (m, 5H), 7.06 – 7.05 (m, 1H), 6.98 (dd, *J* = 8.0, 1.5 Hz, 2H), 4.63 (tt, *J* = 6.0, 3.5 Hz, 1H), 2.00 – 1.86(m, 7H), 1.95 (s, 3H), 1.74 – 1.64 (m, 4H), 1.59 – 1.54 (m, 2H).

^{13}C NMR (126 MHz, $CDCl_3$) δ 142.0, 140.6, 138.3, 130.6, 129.3, 128.0, 127.9, 127.1, 125.7, 74.3, 31.5, 29.8, 28.0, 22.4, 16.3.

HRMS (ESI) m/z calculated for $C_{23}H_{28}BSONa$ $[M+H]^+$, 363.1948, found: 363.1947.

10-(2-methyl-6-phenylhex-2-en-3-yl)-9-oxa-10-borabicyclo[3.3.2]decane (6a)

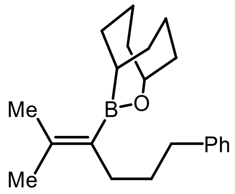

Prepared following **General Procedure A**. Purification by flash column chromatography (aluminum oxide, neutral for chromatography, 5-10% DCM in pentane) gave the title compound (119.4 mg, 77%).

R*f* = 0.60 (20:1 pentane: EtOAc, CAM)

^{1}H NMR (400 MHz, CD_2Cl_2) δ 7.27 – 7.24 (m, 2H), 7.22 – 7.09 (m, 3H), 4.63 – 4.52 (m, 1H), 2.60 (t, *J* = 7.6 Hz, 2H), 2.11 (t, *J* = 8.0 Hz, 2H), 1.89 – 1.80 (m, 4H), 1.79 (s, 3H), 1.62 – 1.56 (m, 7H), 1.64 (s, 3H), 1.50 – 1.39 (m, 4H).

^{13}C NMR (101 MHz, CD_2Cl_2) δ 143.4, 137.7, 128.8, 128.5, 125.8, 73.7, 36.5, 32.6, 32.1, 30.9, 26.6, 24.8, 22.8, 20.3.

HRMS (ESI) m/z calculated for $C_{21}H_{31}BONa$ $[M+Na]^+$, 333.2360, found: 333.2361.

(*E*)-10-(5-methyl-1-phenylnon-4-en-4-yl)-9-oxa-10-borabicyclo[3.3.2]decane (6b)

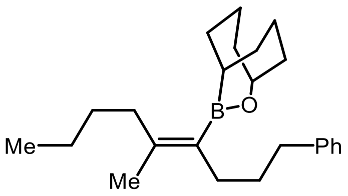

Prepared following **General Procedure A**. Purification by flash column chromatography (aluminum oxide, neutral for chromatography, 5-10% DCM in pentane) gave the title compound (135.6 mg, 77%, >20:1 *E*/*Z*).

R*f* = 0.60 (20:1 pentane: EtOAc, CAM)

^{1}H NMR (400 MHz, CD_2Cl_2) δ 7.28 – 7.23 (m, 2H), 7.20 – 7.13 (m, 3H), 4.56 (tt, *J* = 5.6, 3.2 Hz, 1H), 2.61 (t, *J* = 7.6 Hz, 2H), 2.16 – 2.07 (m, 4H), 1.87 – 1.81 (m, 4H), 1.71 – 1.57 (m, 7H), 1.62 (s, 3H), 1.50 – 1.27 (m, 9H), 0.90 (t, *J* = 7.2 Hz, 3H).

^{13}C NMR (101 MHz, CD_2Cl_2) δ 143.4, 141.5, 128.8, 128.5, 125.8, 73.7, 39.2, 36.6, 32.5, 32.1, 30.8, 26.8, 23.4, 22.8, 22.5, 17.6, 14.4.

HRMS (ESI) m/z calculated for $C_{24}H_{37}BONa$ $[M+Na]^+$, 375.2830, found: 375.2831.

(*E*)-10-(5,7-dimethyl-1-phenyloct-4-en-4-yl)-9-oxa-10-borabicyclo[3.3.2]decane (6c)

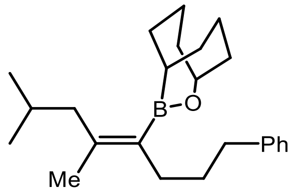

Prepared following **General Procedure A**. Purification by flash column chromatography (aluminum oxide, neutral for chromatography, 5-10% DCM in pentane) gave the title compound (132.1 mg, 75%, >20:1 *E*/*Z*).

R*f* = 0.62 (20:1 pentane: EtOAc, CAM)

^{1}H NMR (400 MHz, CD_2Cl_2) δ 7.28 – 7.24 (m, 2H), 7.20 – 7.13 (m, 3H), 4.56 (tt, *J* = 5.6, 3.2 Hz, 1H), 2.62 (t, *J* = 8.0 Hz, 2H), 2.14 – 2.09 (m, 2H), 2.02 (d, *J* = 7.2 Hz, 2H), 1.89 – 1.78 (m, 5H), 1.74 – 1.60 (m, 7H), 1.59 (s, 3H), 1.52 – 1.39 (m, 4H), 0.85 (d, *J* = 6.8 Hz, 6H).

^{13}C NMR (101 MHz, CD_2Cl_2) δ 139.8, 128.8, 125.8, 73.7, 48.3, 36.6, 32.5, 32.1, 30.8, 27.2, 26.9, 22.8, 22.6, 17.4.

HRMS (ESI) m/z calculated for $C_{24}H_{37}BONa$ $[M+Na]^+$, 375.2830, found: 375.2832.

<u>(*E*)-10-(2-methyl-1,6-diphenylhex-2-en-3-yl)-9-oxa-10-borabicyclo[3.3.2]decane(6d)</u>

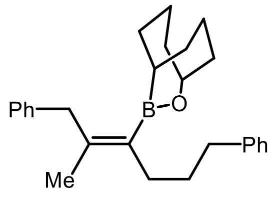

Prepared following **General Procedure A**. Purification by flash column chromatography (aluminum oxide, neutral for chromatography, 5-10% DCM in pentane) gave the title compound (129.4 mg, 67%, 18:1 *E*/*Z*).

R$_f$ = 0.52 (20:1 pentane: EtOAc, CAM)

^{1}H NMR (400 MHz, CD_2Cl_2) δ 7.26 – 7.15 (m, 10H), 4.61 (tt, *J* = 5.6, 3.2 Hz, 1H), 3.47 (s, 2H), 2.64 (t, *J* = 7.6 Hz, 2H), 2.24 – 2.14 (m, 2H), 1.91 – 1.75 (m, 6H), 1.71 – 1.57 (m, 7H), 1.50 – 1.42 (m, 7H).

^{13}C NMR (101 MHz, CD_2Cl_2) δ 143.3, 141.7, 138.8, 129.2, 128.8, 128.54, 128.50, 126.0, 125.9, 73.9, 44.9, 36.6, 32.5, 32.1, 30.8, 26.7, 22.8, 17.5.

HRMS (ESI) m/z calculated for $C_{27}H_{35}BONa$ $[M+Na]^+$, 409.2673, found: 409.2677.

<u>(*E*)-10-(2,3-dimethyl-7-phenylhept-3-en-4-yl)-9-oxa-10-borabicyclo[3.3.2]decane (6e)</u>

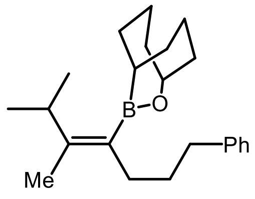

Prepared following **General Procedure A**. Purification by preparative TLC (8% EtOAc in pentane) gave the title compound (91.3 mg, 54%, >20:1 *E*/*Z*).

R$_f$ = 0.58 (20:1 pentane: EtOAc, CAM)

^{1}H NMR (400 MHz, CD_2Cl_2) δ 7.29 – 7.24 (m, 2H), 7.22 – 7.13 (m, 3H), 4.57 (tt, *J* = 5.6, 3.2 Hz, 1H), 2.70 – 2.60 (m, 3H), 2.12 – 2.06 (m, 2H), 1.90 – 1.82 (m, 4H), 1.73 – 1.59 (m, 8H), 1.52 (s, 3H), 1.46 – 1.41 (m, 4H), 0.99 (d, *J* = 6.8 Hz, 6H).

^{13}**C NMR** (101 MHz, CD_2Cl_2) δ 145.2, 143.5, 128.8, 128.5, 125.9, 73.8, 36.6, 36.4, 32.4, 32.1, 30.5, 26.8, 22.8, 21.6, 11.2.

HRMS (ESI) m/z calculated for $C_{23}H_{35}BONa$ $[M+Na]^+$, 361.2673, found: 361.2671.

(*E*)-10-(2-cyclohexyl-6-phenylhex-2-en-3-yl)-9-oxa-10-borabicyclo[3.3.2]decane (6f)

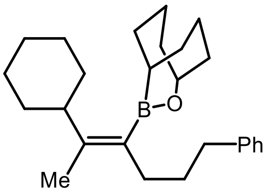

Prepared following **General Procedure A**. Purification by preparative TLC (8% EtOAc in pentane) gave the title compound (130.5 mg, 69%, >20:1 *E*/*Z*).

R*f* = 0.60 (20:1 pentane: EtOAc, CAM)

^{1}**H NMR** (400 MHz, CD_2Cl_2) δ 7.28 – 7.24 (m, 2H), 7.20 – 7.13 (m, 3H), 4.57 (tt, *J* = 5.6, 3.2 Hz, 1H), 2.61 (t, *J* = 7.6 Hz, 2H), 2.27 (tt, *J* = 11.6, 3.2 Hz, 1H), 2.09 (t, *J* = 8.0 Hz, 2H), 1.92 – 1.83 (m, 4H), 1.74 – 1.61 (m, 10H), 1.52 (s, 3H), 1.50 – 1.44 (m, 5H), 1.39 – 1.13 (m, 6H).

^{13}**C NMR** (101 MHz, CD_2Cl_2) δ 145.1, 143.5, 128.8, 128.5, 125.8, 73.7, 47.8, 36.6, 32.4, 32.12, 32.11, 30.5, 27.2, 26.9, 26.7, 22.8, 12.7.

HRMS (ESI) m/z calculated for $C_{26}H_{39}BONa$ $[M+Na]^+$, 401.2986, found: 401.2985.

(*E*)-10-(2,6-diphenylhex-2-en-3-yl)-9-oxa-10-borabicyclo[3.3.2]decane (6g)

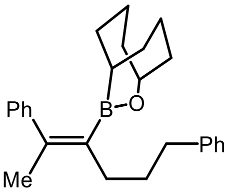

Prepared following **General Procedure A**. Purification by flash column chromatography (aluminum oxide, neutral for chromatography, 5-10% DCM in pentane) gave the title compound (78.2 mg, 42%, >20:1 *E*/*Z*).

R*f* = 0.55 (20:1 pentane: EtOAc, CAM)

^{1}**H NMR** (400 MHz, $CDCl_3$) δ 7.29 – 7.18 (m, 10H), 4.50 (tt, *J* = 6.0, 3.2 Hz, 1H), 2.71 (t, *J* = 7.6 Hz, 2H), 2.34 – 2.28 (m, 2H), 2.00 (s, 3H), 1.81 – 1.61 (m, 7H), 1.58 – 1.51 (m, 2H), 1.39 – 1.31 (m, 2H), 1.09 – 0.97 (m, 4H).

^{13}C NMR (101 MHz, $CDCl_3$) δ 147.5, 143.9, 143.0, 128.6, 128.40, 128.36, 128.0, 126.7, 125.7, 73.6, 36.5, 31.9, 31.8, 31.6, 26.5, 22.3, 20.4.

HRMS (ESI) m/z calculated for $C_{26}H_{33}BONa$ $[M+Na]^+$, 395.2517, found: 395.2518.

(*E*)-10-(1-methoxy-2-methyl-6-phenylhex-2-en-3-yl)-9-oxa-10-borabicyclo[3.3.2]decane (6h)

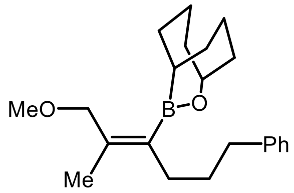

Prepared following **General Procedure A**. Purification by flash column chromatography (aluminum oxide, neutral for chromatography, 5-10% DCM in pentane) gave the title compound (115.7 mg, 68%, 18:1 *E*/*Z*).

R*f* = 0.50 (20:1 pentane: EtOAc, CAM)

^{1}H NMR (400 MHz, CD_2Cl_2) δ 7.27 – 7.23 (m, 2H), 7.20 – 7.14 (m, 3H), 4.57 – 4.52 (m, 1H), 3.86 (s, 2H), 3.25 (s, 3H), 2.62 (t, J = 7.6 Hz, 2H), 2.13 (t, J = 8.0 Hz, 2H), 1.89 – 1.81 (m, 4H), 1.71 – 1.60 (m, 7H), 1.62 (s, 3H), 1.48 – 1.41 (m, 4H).

^{13}C NMR (101 MHz, CD_2Cl_2) δ 143.2, 135.9, 128.8, 128.5, 125.9, 77.0, 73.9, 57.6, 36.6, 32.0, 31.9, 30.4, 26.9, 22.7, 15.3.

HRMS (ESI) m/z calculated for $C_{22}H_{33}BO_2Na$ $[M+Na]^+$, 363.2466, found: 363.2461.

(*E*)-(3-(9-oxa-10-borabicyclo[3.3.2]decan-10-yl)-6-phenylhex-2-en-2-yl)trimethylsilane (6i)

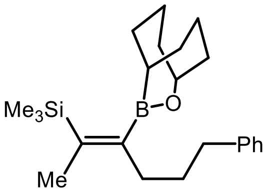

Prepared following **General Procedure A**. Purification by preparative TLC (5% EtOAc in pentane) gave the title compound (93.8 mg, 51%, >20:1 *E*/*Z*).

R*f* = 0.78 (20:1 pentane: EtOAc, CAM)

^{1}**H NMR** (400 MHz, CD_2Cl_2) δ 7.28 – 7.24 (m, 2H), 7.23 – 7.15 (m, 3H), 4.58 (tt, J = 6.0, 3.2 Hz, 1H), 2.65 (t, J = 7.6 Hz, 2H), 2.28 – 2.21 (m, 2H), 1.97 – 1.79 (m, 5H), 1.76 (s, 3H), 1.67 – 1.56 (m, 6H), 1.48 – 1.39 (m, 4H), 0.11 (s, 9H).

^{13}**C NMR** (101 MHz, CD_2Cl_2) δ 145.2, 143.2, 128.9, 128.5, 126.0, 73.8, 36.9, 32.0, 31.7, 31.6, 27.1, 22.7, 18.7, 1.4.

HRMS (ESI) m/z calculated for $C_{23}H_{37}BSiONa$ $[M+Na]^+$, 391.2559, found: 391.2562.

(*E*)-10-(4-methyl-1-phenylhept-3-en-3-yl)-9-oxa-10-borabicyclo[3.3.2]decane (6j)

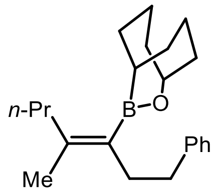

Prepared following **General Procedure A**. Purification by flash column chromatography (aluminum oxide, neutral for chromatography, 5-10% DCM in pentane) gave the title compound (108.6 mg, 67%, >20:1 *E*/*Z*).

R_f = 0.56 (20:1 pentane: EtOAc, CAM)

^{1}**H NMR** (400 MHz, CD_2Cl_2) δ 7.26 – 7.24 (m, 2H), 7.21 – 7.14 (m, 3H), 4.61 (tt, J = 5.6, 3.2 Hz, 1H), 2.60 – 2.55 (m, 2H), 2.37 – 2.31 (m, 2H), 2.13 – 2.08 (m, 2H), 1.93 – 1.86 (m, 4H), 1.75 – 1.64 (m, 5H), 1.63 (s, 3H), 1.52 – 1.41 (m, 6H), 0.89 (t, J = 7.2 Hz, 3H).

^{13}**C NMR** (101 MHz, CD_2Cl_2) δ 143.6, 142.1, 128.8, 128.5, 125.9, 73.8, 41.4, 37.0., 33.8, 32.1, 26.8, 22.8, 17.4, 14.3.

HRMS (ESI) m/z calculated for $C_{22}H_{33}BONa$ $[M+Na]^+$, 347.2517, found: 347.2527.

(*E*)-10-(5-methyl-1-(methylthio)oct-4-en-4-yl)-9-oxa-10-borabicyclo[3.3.2]decane (6k)

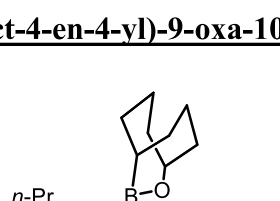

Prepared following **General Procedure A**. Purification by flash column chromatography (aluminum oxide, neutral for chromatography, 5-10% DCM in pentane) gave the title compound (90.9 mg, 59%, >20:1 *E*/*Z*).

R_f = 0.60 (20:1 pentane: EtOAc, CAM)

^{1}H NMR (400 MHz, CD_2Cl_2) δ 4.57 (tt, *J* = 5.2, 3.2 Hz, 1H), 2.46 (t, *J* = 7.6 Hz, 2H), 2.15 (m, *J* = 8.0 Hz, 2H), 2.10 – 2.06 (m, 2H), 2.07 (s, 3H), 1.91 – 1.81 (m, 4H), 1.72 – 1.63 (m, 5H), 1.65 (s, 3H), 1.60 – 1.55 (m, 2H), 1.50 – 1.40 (m, 6H), 0.88 (t, *J* = 7.6 Hz, 3H).

^{13}C NMR (101 MHz, CD_2Cl_2) δ 141.6, 73.8, 41.4, 34.7, 32.1, 30.3, 30.1, 26.8, 22.84, 22.81, 17.5, 15.6, 14.3.

HRMS (ESI) m/z calculated for $C_{18}H_{33}BSONa$ $[M+Na]^+$, 331.2237, found: 331.2235.

<u>(*E*)-10-(1-cyclohexyl-3-methylhex-2-en-2-yl)-9-oxa-10-borabicyclo[3.3.2]decane (6l)</u>

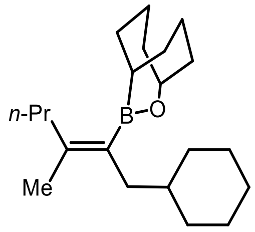

Prepared following **General Procedure A**. Purification by flash column chromatography (aluminum oxide, neutral for chromatography, 5-8% DCM in pentane) gave the title compound (128.1 mg, 81%, >20:1 *E*/*Z*).

R_f = 0.66 (20:1 pentane: EtOAc, CAM)

^{1}H NMR (400 MHz, CD_2Cl_2) δ 4.56 (tt, *J* = 5.4, 3.2 Hz, 1H), 2.09 (t, *J* = 8.0 Hz, 2H), 1.97 (d, *J* = 7.2 Hz, 2H), 1.91 – 1.82 (m, 4H), 1.74 – 1.62 (m, 10H), 1.61 (s, 3H), 1.54 – 1.35 (m, 7H), 1.25 – 1.13 (m, 4H), 0.89 (d, *J* = 7.2 Hz, 3H), 0.86 – 0.79 (m, 2H).

^{13}C NMR (101 MHz, CD_2Cl_2) δ 141.0, 73.7, 41.4, 39.5, 39.0, 34.1, 32.1, 27.2, 27.1, 26.9, 23.0, 18.1, 14.4.

HRMS (ESI) m/z calculated for $C_{21}H_{37}BONa$ $[M+Na]^+$, 339.2830, found: 339.2833.

<u>(*E*)-10-(5-methyl-2-phenyloct-4-en-4-yl)-9-oxa-10-borabicyclo[3.3.2]decane(6m)</u>

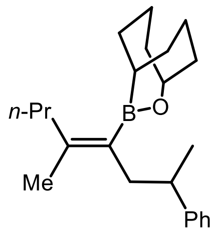

Prepared following **General Procedure A**. Purification by flash column chromatography (aluminum oxide, neutral for chromatography, 5-10% DCM in pentane) gave the title compound (111.5 mg, 66%, >20:1 *E*/*Z*).

R*f* = 0.60 (20:1 pentane: EtOAc, CAM)

^{1}H NMR (400 MHz, CD_2Cl_2) δ 7.30 – 7.20 (m, 4H), 7.17 – 7.13 (m, 1H), 4.60 (tt, *J* = 6.0, 3.2 Hz, 1H), 2.73 (tq, *J* = 7.2, 7.2 Hz, 1H), 2.33 (d, *J* = 7.6 Hz, 2H), 2.13 – 2.06 (m, 2H), 1.94 – 1.79 (m, 5H), 1.76 – 1.67 (m, 4H), 1.56 (s, 3H), 1.50 – 1.39 (m, 6H), 1.21 (d, *J* = 6.8 Hz, 3H), 0.89 (t, *J* = 7.2 Hz, 3H).

^{13}C NMR (101 MHz, CD_2Cl_2) δ 148.8, 142.4, 128.5, 127.4, 126.0, 73.8, 41.5, 41.0, 40.5, 32.6, 31.6, 30.1, 26.9, 26.8, 23.1, 22.9, 22.6, 21.0, 18.1, 14.4.

HRMS (ESI) m/z calculated for $C_{23}H_{35}BONa$ $[M+Na]^+$, 361.2673, found: 361.2675.

<u>(*E*)-10-(1-(bicyclo[2.2.1]hept-5-en-2-yl)-4-methylhept-3-en-3-yl)-9-oxa-10-borabicyclo[3.3.2]decane (6n)</u>

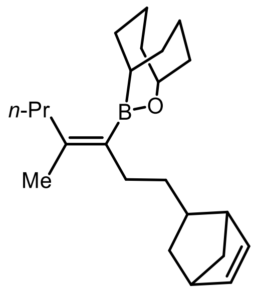

Prepared following **General Procedure A**. Purification by flash column chromatography (aluminum oxide, neutral for chromatography, 5-8% DCM in pentane) gave the title compound (85.0 mg, 50%, >20:1 *E*/*Z*).

R*f* = 0.65 (20:1 pentane: EtOAc, CAM)

^{1}H NMR (400 MHz, CD_2Cl_2) δ 6.10 (dd, *J* = 5.6, 3.2 Hz, 1H), 5.93 (dd, *J* = 5.6, 3.2 Hz, 1H), 4.62 (tt, *J* = 5.6, 3.2 Hz, 1H), 2.76 (d, *J* = 22.4 Hz, 2H), 2.15 – 2.03 (m, 4H), 1.89 – 1.81 (m, 5H), 1.72 – 1.62 (m, 5H), 1.63 (s, 3H), 1.52 – 1.35 (m, 8H), 1.22 – 1.20 (m, 1H), 1.13 – 1.02 (m, 2H), 0.87 (t, *J* = 7.2 Hz, 3H), 0.54 – 0.49 (m, 1H).

^{13}C NMR (101 MHz, CD_2Cl_2) δ 140.6, 137.0, 132.6, 49.7, 45.6, 42.6, 41.2, 39.4, 35.6, 32.6, 31.84, 31.81, 30.1, 26.54, 26.49, 22.6, 22.5, 17.4, 14.4.

HRMS (ESI) m/z calculated for $C_{23}H_{37}BONa$ $[M+Na]^+$, 363.2830, found: 363.2832.

(*S,E*)-10-(4,8,12-trimethyltrideca-4,11-dien-5-yl)-9-oxa-10-borabicyclo[3.3.2]decane (6o)

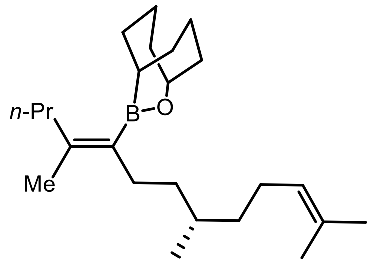

Prepared following **General Procedure A**. Purification by flash column chromatography (aluminum oxide, neutral for chromatography, 5-8% DCM in pentane) gave the title compound (94.9 mg, 53%, >20:1 *E*/*Z*).

R_f = 0.60 (20:1 pentane: EtOAc, CAM)

^{1}H NMR (400 MHz, CD_2Cl_2) δ 5.10 (t, *J* = 7.2 Hz, 1H), 4.62 (tt, *J* = 5.6, 3.2 Hz, 1H), 2.12 – 2.08 (m, 2H), 2.07 – 1.93 (m, 3H), 1.90 – 1.83 (m, 4H), 1.76 – 1.74 (m, 1H), 1.70 – 1.65 (m, 3H), 1.68 (s, 3H), 1.65 (s, 3H), 1.60 (s, 3H), 1.55 – 1.24 (m, 11H), 1.19 – 1.08 (m, 2H), 0.91 – 0.86 (m, 6H).

^{13}C NMR (101 MHz, CD_2Cl_2) δ 140.6, 131.1, 125.3, 73.5, 41.2, 37.5, 37.1, 33.2, 31.8, 28.4, 26.5, 25.9, 25.8, 22.6, 22.5, 19.8, 17.8, 17.4, 14.4.

Specific rotation $[\alpha]_D^{22}$ = 30.6 (c = 0.20, CH_2Cl_2)

HRMS (ESI) m/z calculated for $C_{24}H_{43}BONa$ $[M+Na]^+$, 381.3299, found: 381.3297.

10-((4*E*,6*E*)-4-methyldeca-4,6-dien-5-yl)-9-oxa-10-borabicyclo[3.3.2]decane (6p)

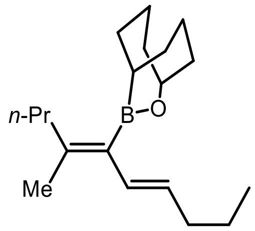

Prepared following **General Procedure A**. Purification by flash column chromatography (aluminum oxide, neutral for chromatography, 5-8% DCM in pentane) gave the title compound (93.7 mg, 65%, >20:1 *E*/*Z*).

R_f = 0.60 (20:1 pentane: EtOAc, UV)

^{1}H NMR (400 MHz, CD_2Cl_2) δ 6.29 (d, *J* = 16.0 Hz, 1H), 5.47 (dt, *J* = 16.0, 7.2 Hz, 1H), 4.62 (tt, *J* = 5.6, 3.2 Hz, 1H), 2.11 – 2.04 (m, 4H), 1.94 – 1.90 (m, 4H), 1.74 – 1.66 (m, 5H), 1.72 (s, 3H), 1.49 – 1.38 (m, 8H), 0.90 (t, *J* = 6.4 Hz, 2x3H).

^{13}C NMR (101 MHz, CD_2Cl_2) δ 141.1, 131.5, 130.9, 74.2, 41.7, 36.2, 32.0, 26.9, 23.4, 22.8, 17.8, 14.4, 13.9.

HRMS (ESI) m/z calculated for $C_{19}H_{33}BONa$ $[M+Na]^+$, 311.2517, found: 311.2518.

10-((1*E*,3*E*)-4-methyl-1-phenylhepta-1,3-dien-3-yl)-9-oxa-10-borabicyclo[3.3.2]decane (6q)

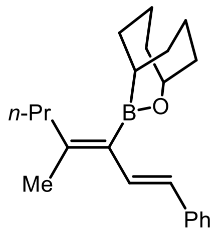

Prepared following **General Procedure A**. Purification by flash column chromatography (aluminum oxide, neutral for chromatography, 5-10% DCM in pentane) gave the title compound (109.5 mg, 68%, >20:1 *E*/*Z*).

R_f = 0.55 (20:1 pentane: EtOAc, UV)

^{1}H NMR (400 MHz, CD_2Cl_2) δ 7.40 (d, *J* = 7.2 Hz, 2H), 7.32 – 7.27 (m, 2H), 7.19 – 7.16 (m, 1H), 6.44 (d, *J* = 16.4 Hz, 1H), 4.71 (tt, *J* = 5.6, 3.2 Hz, 1H), 2.24 – 2.16 (m, 2H), 2.03 – 1.96 (m, 3H), 1.87 (s, 3H), 1.81 – 1.72 (m, 5H), 1.69 – 1.62 (m, 2H), 1.58 – 1.46 (m, 5H), 0.95 (t, *J* = 7.2 Hz, 3H).

^{13}C NMR (101 MHz, CD_2Cl_2) δ 145.1, 139.2, 130.4, 129.2, 128.9, 127.0, 126.2, 74.5, 42.1, 32.1, 26.9, 22.83, 22.78, 18.2, 14.4.

HRMS (ESI) m/z calculated for $C_{22}H_{31}BONa$ $[M+Na]^+$, 345.2360, found: 345.2361.

10-((1*E*,3*E*)-1-cyclopropyl-4-methylhepta-1,3-dien-3-yl)-9-oxa-10-borabicyclo[3.3.2]decane (6r)

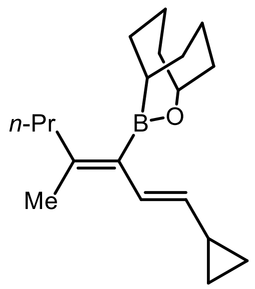

Prepared following **General Procedure A**. Purification by flash column chromatography (aluminum oxide, neutral for chromatography, 5-8% DCM in pentane) gave the title compound (117.3 mg, 82%, >20:1 *E*/*Z*).

R*f* = 0.63 (20:1 pentane: EtOAc, UV)

^{1}H NMR (400 MHz, CD_2Cl_2) δ 6.38 (d, *J* = 16.0 Hz, 1H), 5.00 (dd, *J* = 16.0, 8.8 Hz, 1H), 4.61 (tt, *J* = 5.6, 2.8 Hz, 1H), 2.10 (t, *J* = 8.0 Hz, 2H), 1.92 – 1.84 (m, 5H), 1.73 (s, 3H), 1.69 – 1.63 (m, 5H), 1.47 – 1.39 (m, 6H), 0.90 (t, *J* = 7.2 Hz, 3H), 0.71 (dt, *J* = 6.4, 3.2 Hz, 2H), 0.34 (dt, *J* = 6.4, 3.2 Hz, 2H).

^{13}C NMR (101 MHz, CD_2Cl_2) δ 140.6, 135.2 128.4, 74.2, 41.7, 32.0, 26.9, 22.8, 22.7, 17.9, 15.0, 14.4, 7.2.

HRMS (ESI) m/z calculated for $C_{19}H_{32}BO$ $[M+H]^+$, 287.2541, found: 287.2544.

10-((2*E*,4*E*)-1-methoxy-5-methylocta-2,4-dien-4-yl)-9-oxa-10-borabicyclo[3.3.2]decane(6s)

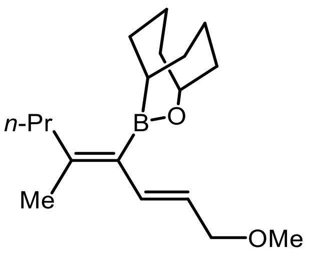

Prepared following **General Procedure A**. Purification by flash column chromatography (aluminum oxide, neutral for chromatography, 5-12% DCM in pentane) gave the title compound (111.7 mg, 77%, >20:1 *E*/*Z*).

R*f* = 0.48 (20:1 pentane: EtOAc, UV)

^{1}H NMR (400 MHz, CD_2Cl_2) δ 6.52 (d, *J* = 16.0 Hz, 1H), 5.56 (dt, *J* = 16.0, 6.4 Hz, 1H), 4.64 (tt, *J* = 5.4, 3.2 Hz, 1H), 3.94 (dd, *J* = 6.4, 1.2 Hz, 2H), 3.28 (s, 3H), 2.20 – 2.09 (m, 2H), 1.98

– 1.89 (m, 4H), 1.75 (s, 3H), 1.74 – 1.64 (m, 5H), 1.61 – 1.55 (m, 2H), 1.51 – 1.43 (m, 4H), 0.91 (t, *J* = 7.6 Hz, 3H).

^{13}C NMR (101 MHz, CD_2Cl_2) δ 144.3, 133.9, 126.5, 74.4, 74.3, 57.6, 41.8, 32.0, 26.8, 22.8, 22.7, 18.0, 14.4.

HRMS (ESI) m/z calculated for $C_{18}H_{31}BONa$ $[M+Na]^+$, 313.2309, found: 313.2308.

(*E*)-10-(1-(4-methoxyphenyl)-2-methylpent-1-en-1-yl)-9-oxa-10-borabicyclo[3.3.2]decane (6t)

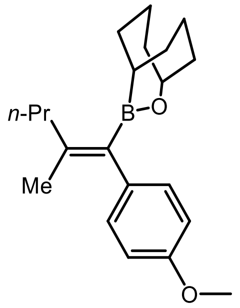

Prepared following **General Procedure C**. Purification by flash column chromatography (aluminum oxide, neutral for chromatography, 5-12% DCM in pentane) gave the title compound (128.8 mg, 79%, >20:1 *E*/*Z*).

R*f* = 0.46 (20:1 pentane: EtOAc, UV)

^{1}H NMR (400 MHz, CD_2Cl_2) δ 6.96 (d, *J* = 8.8 Hz, 2H), 6.83 (d, *J* = 8.8 Hz, 2H), 4.60 (tt, *J* = 5.6, 3.2 Hz, 1H), 3.78 (s, 3H), 2.27 (t, *J* = 8.8 Hz, 2H), 1.88 – 1.79 (m, 4H), 1.72 – 1.60 (m, 4H), 1.56 – 1.39 (m, 7H), 1.55 (s, 3H), 0.97 (t, *J* = 7.6 Hz, 3H).

^{13}C NMR (101 MHz, CD_2Cl_2) δ 157.8, 142.8, 136.4, 130.3, 113.6, 74.0, 55.4, 40.8, 32.1, 26.9, 22.8, 19.3, 14.3.

HRMS (ESI) m/z calculated for $C_{21}H_{31}BO_2Na$ $[M+Na]^+$, 349.2309, found: 349.2314.

(*E*)-10-(3-methylhex-2-en-2-yl)-9-oxa-10-borabicyclo[3.3.2]decane (6u)

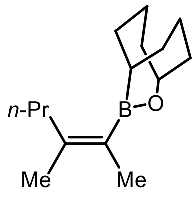

Prepared following **General Procedure C**. Purification by flash column chromatography (aluminum oxide, neutral for chromatography, 5-12% DCM in pentane) gave the title compound (62.1 mg, 53%, >20:1 *E*/*Z*).

R$_f$ = 0.67 (20:1 pentane: EtOAc, CAM)

^{1}H NMR (400 MHz, CD_2Cl_2) δ 4.60 – 4.53 (m, 1H), 2.14 – 2.07 (m, 2H), 1.90 – 1.81 (m, 4H), 1.76 – 1.64 (m, 5H), 1.62 (s, 3H), 1.60 (s, 3H), 1.49 – 1.38 (m, 6H), 0.87 (t, *J* = 7.2 Hz, 3H).

^{13}C NMR (101 MHz, CD_2Cl_2) δ 141.4, 73.7, 41.3, 32.2, 26.8, 22.9, 22.8, 17.8, 16.2, 14.4.

HRMS (ESI) m/z calculated for $C_{15}H_{27}BONa$ $[M+Na]^+$, 257.2047, found: 257.2044.

<u>(*E*)-10-(2,6-dimethylnona-1,5-dien-5-yl)-9-oxa-10-borabicyclo[3.3.2]decane (6v)</u>

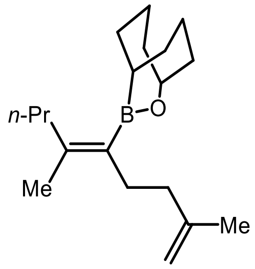

Prepared following **General Procedure C**. Purification by flash column chromatography (aluminum oxide, neutral for chromatography, 5-12% DCM in pentane) gave the title compound (75.0 mg, 52%, >20:1 *E*/*Z*).

R$_f$ = 0.65 (20:1 pentane: EtOAc, CAM)

^{1}H NMR (400 MHz, CD_2Cl_2) δ 4.68 (s, 2H), 4.58 (tt, *J* = 5.2, 3.2 Hz, 1H), 2.21 – 2.16 (m, 2H), 2.09 (t, *J* = 8.0 Hz, 2H), 2.00 – 1.95 (m, 2H), 1.92 – 1.82 (m, 4H), 1.75 (s, 3H), 1.72 – 1.67 (m, 4H), 1.65 (s, 3H), 1.53 – 1.39 (m, 7H), 0.88 (t, *J* = 7.2 Hz, 3H).

^{13}C NMR (101 MHz, CD_2Cl_2) δ 147.4, 141.5, 109.4, 73.7, 41.4, 38.9, 32.1, 30.11, 30.09, 26.8, 22.85, 22.83, 22.6, 17.4, 14.3.

HRMS (ESI) m/z calculated for $C_{19}H_{33}BONa$ $[M+Na]^+$, 311.2517, found: 311.2515.

<u>(*E*)-10-(4,9-dimethyldeca-4,8-dien-5-yl)-9-oxa-10-borabicyclo[3.3.2]decane (6w)</u>

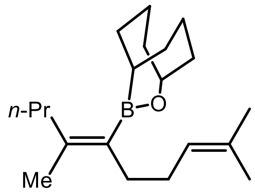

Prepared following **General Procedure C**. Purification by flash column chromatography (aluminum oxide, neutral for chromatography, 5-12% DCM in pentane) gave the title compound (87.6 mg, 58%, >20:1 *E*/*Z*).

R*f* = 0.65 (20:1 pentane: EtOAc, CAM)

^{1}H NMR (400 MHz, CD_2Cl_2) δ 5.16 (t, *J* = 7.2 Hz, 1H), 4.58 (tt, *J* = 5.2, 3.2 Hz, 1H), 2.13 – 2.03 (m, 4H), 1.99 – 1.94 (m, 1H), 1.90 – 1.81 (m, 4H), 1.71 – 1.65 (m, 4H), 1.68 (s, 3H), 1.66 (s, 3H), 1.61 (s, 3H), 1.52 – 1.43 (m, 4H), 1.40 – 1.27 (m, 4H), 0.91 (t, *J* = 7.2 Hz, 3H).

^{13}C NMR (101 MHz, CD_2Cl_2) 141.7, 131.4, 125.4, 73.7, 39.2, 32.14, 32.12, 31.5, 29.4, 26.8, 25.8, 23.4, 22.8, 17.7, 17.6, 14.4.

HRMS (ESI) m/z calculated for $C_{20}H_{35}BONa$ $[M+Na]^+$, 325.2673, found: 325.2678.

(*Z*)-1-methoxy-4-(5-methyl-1-phenyloct-4-en-4-yl)benzene ((*Z*)-7)

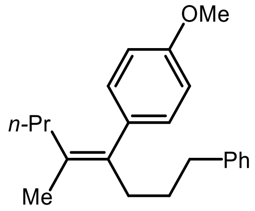

Prepared following procedure in **Section 2.6.1**. Purification by flash column chromatography (silica gel, 3-5% EtOAc in pentane) gave the title compound (49.9 mg, 81%, >20:1 *Z*/*E*).

R*f* = 0.48 (20:1 pentane: EtOAc, CAM)

^{1}H NMR (400 MHz, $CDCl_3$) δ 7.26 – 7.23 (m, 2H), 7.17 – 7.11 (m, 3H), 6.99 (d, *J* = 8.8 Hz, 2H), 6.84 (d, *J* = 8.8 Hz, 2H), 3.82 (s, 3H), 2.56 (t, *J* = 8.0 Hz, 2H), 2.35 (t, *J* = 8.0 Hz, 2H), 1.83 (t, *J* = 7.6 Hz, 2H), 1.73 (s, 3H), 1.63 – 1.55 (m, 2H), 1.39 – 1.29 (m, 2H), 0.75 (t, *J* = 7.2 Hz, 3H).

^{13}C NMR (101 MHz, $CDCl_3$) δ 157.8, 142.8, 136.5, 135.1, 131.9, 130.0, 128.5, 128.3, 125.7, 113.4, 55.3, 37.6, 35.9, 34.6, 30.0, 21.7, 17.5, 14.1.

HRMS (ESI) m/z calculated for $C_{22}H_{28}ONa$ $[M+Na]^+$, 331.2032, found: 331.2033.

(*E*)-(4-butyl-5-methyloct-4-en-1-yl)benzene ((*E*)-8)

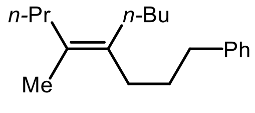

Prepared through sp^2-sp^3 coupling following procedure in **Section 2.6.1** or Zweifel olefination following procedure in **Section 2.6.2** and **Section 2.6.7**. Purification by flash column chromatography (silica gel, pentane) gave the title compound.

R*f* = 0.50 (pentane, $KMnO_4$)

^{1}H NMR (400 MHz, $CDCl_3$) δ 7.30 – 7.24 (m, 2H), 7.19 – 7.14 (m, 3H), 2.59 (t, *J* = 8.0 Hz, 2H), 2.07 – 2.02 (m, 2H), 1.99 – 1.89 (m, 4H), 1.72 – 1.65 (m, 2H), 1.62 (s, 3H), 1.34 – 1.28 (m, 6H), 0.91 – 0.83 (m, 6H).

^{13}C NMR (101 MHz, $CDCl_3$) δ 143.0, 133.2, 128.9, 128.5, 128.4, 125.7, 36.4, 36.3, 32.2, 31.9, 31.6, 30.7, 23.2, 21.9, 18.0, 14.2.

HRMS (ESI) m/z calculated for $C_{19}H_{30}ONa$ $[M+Na]^+$, 281.2240, found: 281.2247.

(*Z*)-(4-butyl-5-methyloct-4-en-1-yl)benzene ((*Z*)-8)

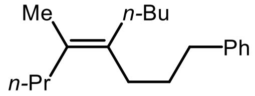

Prepared through Zweifel olefination following procedure in **Section 2.6.2**. Purification by flash column chromatography (silica gel, pentane) gave the title compound (32.0 mg, 62%, 18:1 *Z*/*E*).

R*f* = 0.50 (pentane, $KMnO_4$)

^{1}H NMR (400 MHz, $CDCl_3$) δ 7.29 – 7.25 (m, 2H), 7.19 – 7.15 (m, 3H), 2.60 (t, *J* = 8.0 Hz, 2H), 2.06 – 1.96 (m, 4H), 1.92 (t, *J* = 8.0 Hz, 2H), 1.70 – 1.63 (m, 2H), 1.60 (s, 3H), 1.37 – 1.27 (m, 6H), 0.89 (t, *J* = 7.2 Hz, 3H), 0.84 (t, *J* = 7.2 Hz, 3H).

^{13}C NMR (101 MHz, $CDCl_3$) δ 142.9, 133.2, 128.9, 128.5, 128.4, 125.7, 36.40, 36.38, 32.2, 31.8, 31.2, 31.1, 23.1, 21.9, 18.0, 14.2.

HRMS (ESI) m/z calculated for $C_{19}H_{30}ONa$ $[M+Na]^+$, 281.2240, found: 281.2246.

(*Z*)-(4-butyl-5-methyloct-4-en-1-yl)benzene ((*Z*)-9)

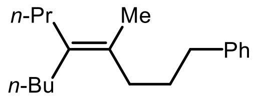

Prepared through Zweifel olefination following procedure in **Section 2.6.2**. Purification by flash column chromatography (silica gel, pentane) gave the title compound (37.7 mg, 73%, >20:1 *Z*/*E*).

R*f* = 0.50 (pentane, $KMnO_4$)

^{1}H NMR (400 MHz, $CDCl_3$) δ 7.29 – 7.24 (m, 2H), 7.19 – 7.14 (m, 3H), 2.59 (t, *J* = 8.0 Hz, 2H), 2.05 (t, *J* = 8.0 Hz, 2H), 1.99 – 1.89 (m, 4H), 1.72 – 1.65 (m, 2H), 1.62 (s, 3H), 1.38 – 1.32 (m, 2H), 1.31 – 1.24 (m, 4H), 0.91 – 0.83 (m, 6H).

^{13}C NMR (101 MHz, $CDCl_3$) δ 143.0, 133.8, 128.5, 128.4, 128.3, 125.7, 36.2, 34.6, 34.0, 31.9, 31.7, 30.7, 23.2, 22.0, 18.1, 14.4, 14.2.

HRMS (ESI) m/z calculated for $C_{19}H_{30}ONa$ $[M+Na]^+$, 281.2240, found: 281.2243.

<u>(*E*)-(4-butyl-5-methyloct-4-en-1-yl)benzene ((*E*)-9)</u>

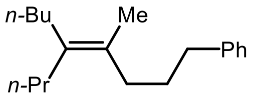

Prepared through Zweifel olefination following procedure in **Section 2.6.2**. Purification by flash column chromatography (silica gel, pentane) gave the title compound (27.9 mg, 54%, 19:1 *E*/*Z*).

R*f* = 0.50 (pentane, $KMnO_4$)

^{1}H NMR (400 MHz, $CDCl_3$) δ 7.29 – 7.24 (m, 2H), 7.19 – 7.14 (m, 3H), 2.59 (t, *J* = 8.0 Hz, 2H), 2.04 (m, *J* = 8.0 Hz, 2H), 1.99 – 1.89 (m, 4H), 1.72 – 1.65 (m, 2H), 1.62 (s, 3H), 1.34 – 1.28 (m, 6H), 0.91 – 0.83 (m, 6H).

^{13}C NMR (101 MHz, $CDCl_3$) δ 143.0, 133.9, 128.5, 128.4, 128.2, 125.7, 36.2, 34.4, 34.0, 32.2, 31.2, 30.7, 23.1, 22.5, 18.0, 14.6, 14.3.

HRMS (ESI) m/z calculated for $C_{19}H_{30}ONa$ $[M+Na]^+$, 281.2240, found: 281.2243.

<u>(*Z*)-(4-iodo-5-methyloct-4-en-1-yl)benzene ((*Z*)-10)</u>

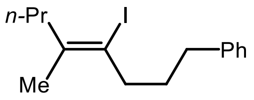

Prepared following procedure in **Section 2.6.3**. Purification by flash column chromatography (silica gel, pentane) gave the title compound (46.6 mg, 71%, >20:1 *Z*/*E*).

R$_f$ = 0.28 (pentane, $KMnO_4$)

^{1}H NMR (400 MHz, $CDCl_3$) δ 7.30 – 7.26 (m, 2H), 7.20 – 7.16 (m, 3H), 2.63 – 2.55 (m, 4H), 2.22 (t, *J* = 8.0 Hz, 2H), 1.88 – 1.82 (m, 2H), 1.74 (s, 3H), 1.49 – 1.39 (m, 2H), 0.94 (t, *J* = 7.2 Hz, 3H).

^{13}C NMR (101 MHz, $CDCl_3$) δ 142.2, 140.1, 128.54, 128.46, 125.9, 102.5, 46.9, 41.3, 34.7, 30.9, 20.8, 17.8, 14.0.

HRMS (ESI) m/z calculated for $C_{15}H_{21}INa$ $[M+Na]^+$, 351.0580, found: 351.0575.

(*Z*)-trimethyl(4-methyl-3-(3-phenylpropyl)oct-3-en-1-yn-1-yl)silane

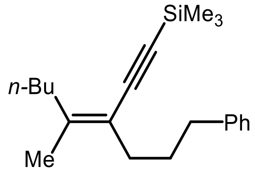

Prepared following procedure in **Section 2.6.4**. Purification by flash column chromatography (silica gel, pentane) gave the title compound (53.0 mg, 85%, >20:1 *Z*/*E*).

R$_f$ = 0.32 (pentane, $KMnO_4$)

^{1}H NMR (400 MHz, $CDCl_3$) δ 7.33 – 7.28 (m, 2H), 7.24 – 7.20 (m, 3H), 2.65 (t, *J* = 8.0 Hz, 2H), 2.39 (t, *J* = 7.6 Hz, 2H), 2.19 (t, *J* = 7.6 Hz, 2H), 1.91 – 1.83 (m, 2H), 1.71 (s, 3H), 1.49 – 1.40 (m, 2H), 1.39 – 1.31 (m, 2H), 0.96 (t, *J* = 7.2 Hz, 3H), 0.22 (s, 9H).

^{13}C NMR (101 MHz, $CDCl_3$) δ 146.3, 142.8, 128.6, 128.4, 125.7, 117.4, 106.5, 95.8, 37.3, 35.4, 31.3, 30.20, 30.16, 22.6, 17.9, 14.1, 0.4.

HRMS (ESI) m/z calculated for $C_{20}H_{33}Si$ $[M+H]^+$, 313.2346, found: 313.2349.

(*Z*)-3-methyl-2-(3-phenylpropyl)hex-2-en-1-ol ((*Z*)-12)

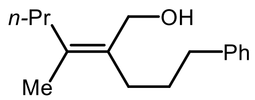

Prepared following procedure in **Section 2.6.5**. Purification by flash column chromatography (silica gel, 5-10% EtOAc in pentane) gave the title compound (28.3 mg, 61%, >20:1 *Z*/*E*).

R$_f$ = 0.42 (10:1 pentane: EtOAc, $KMnO_4$)

^{1}H NMR (400 MHz, $CDCl_3$) δ 7.33 – 7.29 (m, 2H), 7.22 – 7.19 (m, 3H), 3.63 (t, J = 7.2 Hz, 2H), 2.64 (t, J = 7.8 Hz, 2H), 2.37 (t, J = 7.2 Hz, 2H), 2.11 – 2.04 (m, 4H), 1.75 – 1.69 (m, 2H), 1.65 (s, 3H), 1.46 – 1.39 (m, 3H), 0.92 (t, J = 7.2 Hz, 3H).

^{13}C NMR (101 MHz, $CDCl_3$) δ 142.6, 133.0, 128.5, 128.4, 128.3, 125.8, 61.5, 36.5, 36.2, 35.2, 32.2, 30.7, 21.9, 18.2, 14.2.

HRMS (ESI) m/z calculated for $C_{16}H_{24}SiNa$ $[M+Na]^+$, 255.1719, found: 255.1709.

<u>(*Z*)-3-methyl-2-(3-phenylpropyl)hex-2-enoic acid ((*Z*)-13)</u>

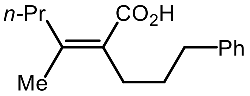

Prepared following procedure in **Section 2.6.6**. Purification by flash column chromatography (silica gel, 5-16% EtOAc in pentane) gave the title compound (51.7 mg, 42%, >20:1 *Z*/*E*).

R*f* = 0.40 (4:1 pentane: EtOAc, UV)

^{1}H NMR (400 MHz, $CDCl_3$) δ 7.33 – 7.29 (m, 2H), 7.24 – 7.19 (m, 3H), 2.68 (t, J = 7.6 Hz, 2H), 2.44 – 2.37 (m, 4H), 1.84 – 1.76 (m, 5H), 1.55 – 1.49 (m, 2H), 0.96 (t, J = 7.2 Hz, 3H).

^{13}C NMR (101 MHz, $CDCl_3$) δ 175.1, 150.4, 142.5, 128.5, 128.4, 127.1, 125.8, 38.6, 35.9, 30.7, 29.8, 21.9, 20.5, 14.2.

HRMS (ESI) m/z calculated for $C_{16}H_{22}O_2Na$ $[M+Na]^+$, 269.1512, found: 269.1510.

<u>(*Z*)-(4-allyl-5-methyloct-4-en-1-yl)benzene ((*Z*)-14)</u>

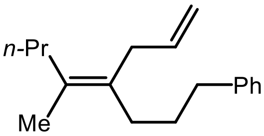

Prepared following procedure in **Section 2.6.8**. Purification by flash column chromatography (silica gel, pentane) gave the title compound (83.5 mg, 69%, >20:1 *Z*/*E*).

R*f* = 0.52 (pentane, $KMnO_4$)

^{1}H NMR (400 MHz, $CDCl_3$) δ 7.32 – 7.29 (m, 2H), 7.22 – 7.18 (m, 3H), 5.76 (ddt, J = 16.4, 10.0, 6.4 Hz, 1H), 5.02 – 4.96 (m, 2H), 2.81 (d, J = 6.4 Hz, 2H), 2.62 (t, J = 8.0 Hz, 2H), 2.10 – 2.06 (m, 2H), 2.04 – 2.00 (m, 2H), 1.74 – 1.68 (m, 2H), 1.65 (s, 3H), 1.46 – 1.37 (m, 2H), 0.91 (t, J = 7.2 Hz, 3H).

^{13}C NMR (101 MHz, $CDCl_3$) δ 142.9, 137.5, 130.7, 130.2, 128.5, 128.4, 125.7, 114.7, 36.7, 36.5, 36.2, 32.1, 30.4, 21.8, 18.2, 14.2.

HRMS (ESI) m/z calculated for $C_{16}H_{26}Na$ $[M+Na]^+$, 265.1927, found: 265.1926.

(*Z*)-3-methyl-1-phenyl-2-(3-phenylpropyl)hex-2-en-1-ol ((*Z*)-15)

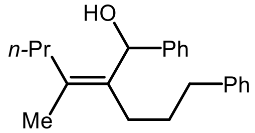

Prepared following procedure in **Section 2.6.9**. Purification by flash column chromatography (silica gel, 5-10% EtOAc in pentane) gave the title compound (73.9 mg, 48%, >20:1 *Z*/*E*).

R$_f$ = 0.55 (10:1 pentane:EtOAc, $KMnO_4$)

^{1}H NMR ^{1}H NMR (400 MHz, CD_2Cl_2) δ 7.35 – 7.28 (m, 4H), 7.25 – 7.19 (m, 3H), 7.14 – 7.11 (m, 1H), 7.03 (d, *J* = 7.2 Hz, 2H), 5.83 (d, *J* = 3.2 Hz, 1H), 2.49 – 2.35 (m, 2H), 2.28 – 2.19 (m, 2H), 2.05 – 1.97 (m, 1H), 1.91 – 1.83 (m, 1H), 1.68 (s, 3H), 1.62 – 1.45 (m, 3H), 1.17 – 1.08 (m, 1H), 0.95 (t, *J* = 7.2 Hz, 3H).

^{13}C NMR (101 MHz, CD_2Cl_2) δ 143.9, 143.0, 135.3, 134.7, 128.7, 128.5, 128.3, 127.0, 126.2, 125.9, 72.3, 36.8, 36.5, 32.3, 28.1, 22.7, 19.0, 14.4.

HRMS (ESI) m/z calculated for $C_{22}H_{28}Na$ $[M+Na]^+$, 331.2032, found: 331.2035.

***cis*-Tamoxifen**

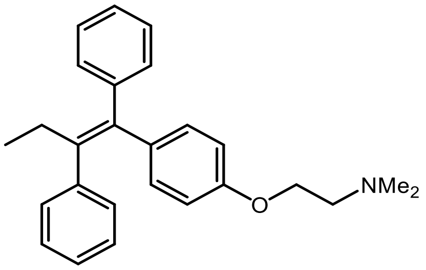

Prepared following procedure in **Section 2.6.10**. Purification by flash column chromatography (silica gel, 10-25% EtOAc in pentane) gave the title compound (790.1 mg, 71%, >20:1 *Z*/*E*).

R$_f$ = 0.30 (3:1 pentane:EtOAc, UV)

Melting Point: 102 – 104 °C.

^{1}H NMR (400 MHz, $CDCl_3$) δ 7.37 – 7.33 (m, 2H), 7.28 – 7.23 (m, 3H), 7.19 – 7.15 (m, 2H), 7.13 – 7.11 (m, 3H), 6.77 (d, J = 8.8 Hz, 2H), 6.56 (d, J = 8.8 Hz, 2H), 3.94 (t, J = 6.0 Hz, 2H), 2.66 (t, J = 6.0 Hz, 2H), 2.46 (q, J = 7.2 Hz, 2H), 2.30 (s, 6H), 0.93 (t, J = 7.2 Hz, 3H).

^{13}C NMR (101 MHz, $CDCl_3$) δ 156.8, 144.0, 142.5, 141.4, 138.4, 135.7, 132.0, 129.8, 129.6, 128.2, 128.0, 126.6, 126.1, 113.5, 65.7, 58.4, 46.0, 29.1, 13.7.

HRMS (ESI) m/z calculated for $C_{26}H_{27}NONa$ $[M+Na]^+$, 394.2141, found: 394.2146.

(*E*)-4,4'-(hex-3-ene-3,4-diyl)bis(methoxybenzene) (18)

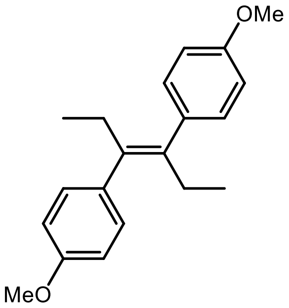

Prepared following procedure in **Section 2.6.11**. Purification by flash column chromatography (silica gel, 5-10% EtOAc in pentane) gave the title compound (242.7 mg, 82%, >20:1 *Z*/*E*).

R*f* = 0.42 (10:1 pentane:EtOAc, UV)

^{1}H NMR (400 MHz, $CDCl_3$) δ 7.12 (d, J = 8.8 Hz, 4H), 6.90 (d, J = 8.8 Hz, 4H), 3.84 (s, 6H), 2.13 (q, J = 7.2 Hz, 4H), 0.77 (t, J = 7.2 Hz, 6H).

^{13}C NMR (101 MHz, $CDCl_3$) δ 158.1, 138.9, 135.2, 129.9, 113.5, 55.4, 28.7, 13.6.

HRMS (ESI) m/z calculated for $C_{20}H_{24}O_2Na$ $[M+Na]^+$, 319.1669, found: 319.1674.

(*Z*)-5-allyl-2,6,10-trimethylundeca-1,5,9-triene ((*Z*)-24)

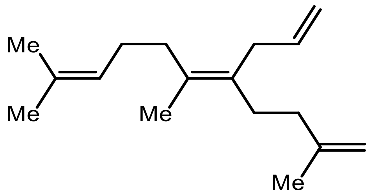

Prepared following procedure in **Section 2.6.12**. Purification by flash column chromatography (silica gel, pentane) gave the title compound (89.4 mg, 77%, 12:1 *Z*/*E*).

R*f* = 0.49 (pentane, $KMnO_4$)

^{1}H NMR (400 MHz, $CDCl_3$) δ 5.75 (ddt, *J* = 16.4, 10.0, 6.4 Hz, 1H), 5.14 – 5.10 (m, 1H), 5.03 – 4.95 (m, 2H), 4.69 (s, 2H), 2.79 (d, *J* = 6.4 Hz, 2H), 2.15 – 2.10 (m, 2H), 2.06 – 1.99 (m, 6H), 1.74 (s, 3H), 1.68 (s, 6H), 1.61 (s, 3H).

^{13}C NMR (101 MHz, $CDCl_3$) δ 146.6, 137.4, 131.6, 130.4, 130.3, 124.6, 114.8, 109.6, 36.70, 36.66, 34.6, 31.0, 27.2, 25.9, 22.7, 18.2, 17.8.

HRMS (ESI) m/z calculated for $C_{17}H_{28}Na$ $[M+Na]^+$, 255.2083, found: 255.2081.

<u>(*Z*)-γ-bisabolene</u>

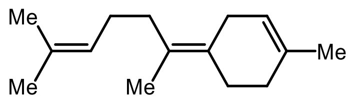

Prepared following procedure in **Section 2.6.12**. Purification by flash column chromatography (silica gel, pentane) gave the title compound (37.9 mg, 93%, 12:1 *Z*/*E*).

R*f* = 0.50 (pentane, $KMnO_4$)

^{1}H NMR (400 MHz, $CDCl_3$) δ 5.35 (m, 1H), 5.13 – 5.09 (m, 1H), 2.74 (brs, 2H), 2.31 (t, *J* = 6.4 Hz, 2H), 2.08 – 1.99 (m, 6H), 1.69 (s, 3H), 1.67 (s, 6H), 1.59 (s, 3H).

^{13}C NMR (101 MHz, $CDCl_3$) δ 134.3, 131.6, 128.6, 125.8, 124.6, 121.0, 34.6, 31.7, 29.5, 27.0, 26.9, 25.9, 23.5, 18.0, 17.8.

HRMS (ESI) m/z calculated for $C_{15}H_{24}Na$ $[M+Na]^+$, 227.1770, found: 227.1773.

<u>(*E*)-5-allyl-2,6,10-trimethylundeca-1,5,9-triene ((*E*)-24)</u>

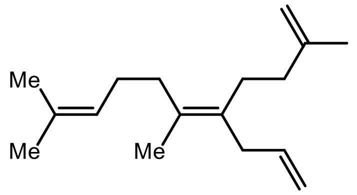

Prepared following procedure in **Section 2.6.12**. Purification by flash column chromatography (silica gel, pentane) gave the title compound (68.4 mg, 59%, 12:1 *E*/*Z*).

R*f* = 0.48 (pentane, $KMnO_4$)

^{1}H NMR (400 MHz, $CDCl_3$) δ 5.75 (ddt, *J* = 16.4, 10.0, 6.4 Hz, 1H), 5.14 – 5.10 (m, 1H), 5.03 – 4.95 (m, 2H), 4.69 (s, 2H), 2.80 – 2.76 (m, 2H), 2.15 – 2.10 (m, 2H), 2.06 – 1.99 (m, 6H), 1.74 (s, 3H), 1.68 (s, 3H), 1.65 (s, 3H), 1.61 (s, 3H).

^{13}C NMR (101 MHz, $CDCl_3$) δ 146.6, 136.7, 131.6, 130.6, 130.2, 124.6, 114.5, 109.5, 37.3, 36.9, 34.6, 30.9, 27.3, 25.9, 22.7, 18.2, 17.8.

HRMS (ESI) m/z calculated for $C_{17}H_{28}Na$ $[M+Na]^+$, 255.2083, found: 255.2080.

<u>(*E*)-γ-bisabolene</u>

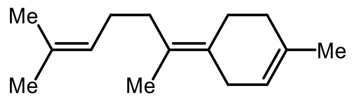

Prepared following procedure in **Section 2.6.12**. Purification by flash column chromatography (silica gel, pentane) gave the title compound (38.8 mg, 95%, 12:1 *E*/*Z*).

R*f* = 0.50 (pentane, $KMnO_4$)

^{1}H NMR (500 MHz, $CDCl_3$) δ 5.39 – 5.36 (m, 1H), 5.16 – 5.10 (m, 1H), 2.73 – 2.72 (m, 2H), 2.33 (t, *J* = 6.4 Hz, 2H), 2.09 – 1.99 (m, 6H), 1.68 (s, 3H), 1.66 (s, 3H), 1,65 (3, 3H), 1.60 (s, 3H).

^{13}C NMR (126 MHz, $CDCl_3$) δ 134.2, 131.4, 128.4, 125.9, 124.5, 120.8, 34.2, 31.8, 29.7, 27.3, 26.5, 25.7, 23.4, 18.4, 17.6.

HRMS (ESI) m/z calculated for $C_{15}H_{24}Na$ $[M+Na]^+$, 227.1770, found: 227.1776.

2.10. References

1 Li, H. *et al.* Synthesis of Multifluoromethylated γ-Sultines by a Photoinduced Radical Addition–Polar Cyclization. *Angew. Chem. Int. Ed.* **62**, e202300159 (2023).

2 Higuchi, K. *et al.* Preparation of Alkyl Di(p-tolyl)sulfonium Salts and Their Application in Metal-Free C(sp3)–C(sp3) and C(sp3)–C(sp2) Bond Formations. *Org. Lett.* **25**, 3766-3771 (2023).

3 Vasu, D., Yorimitsu, H. & Osuka, A. Base-Free Palladium-Catalyzed Cross-Coupling of Arylsulfonium Salts with Sodium Tetraarylborates. *Synthesis* **47**, 3286-3291 (2015).

4 Fang, G. Y. *et al.* Asymmetric Sulfur Ylide Reactions with Boranes: Scope and Limitations, Mechanism and Understanding. *J. Am. Soc. Chem.* **129**, 14632-14639 (2007).

5 Ishida, N., Shimamoto, Y. & Murakami, M. Stereoselective Synthesis of (E)-(Trisubstituted alkenyl)borinic Esters: Stereochemistry Reversed by Ligand in the Palladium-Catalyzed Reaction of Alkynylborates with Aryl Halides. *Org. Lett.* **11**, 5434-5437 (2009).

6 Nishihara, Y. *et al.* Highly Regio- and Stereoselective Synthesis of Multialkylated Olefins through Carbozirconation of Alkynylboronates and Sequential Negishi and Suzuki–Miyaura Coupling Reactions. *Angew. Chem. Int. Ed.* **50**, 8660-8664 (2011).

7 Armstrong, R. J., García-Ruiz, C., Myers, E. L. & Aggarwal, V. K. Stereodivergent Olefination of Enantioenriched Boronic Esters. *Angew. Chem. Int. Ed.* **56**, 786-790 (2017).

8 Brown, H. C. *et al.* Vinylic organoboranes. 13. A convenient stereospecific synthesis of (Z)-1-halo-1-alkenes from 1-alkynes via (E)-1-alkenylborane derivatives with halogens. *J. Org. Chem.* **54**, 6068-6075 (1989).

9. Canterbury, D. P. & Micalizio, G. C. Polyketide Assembly by Alkene−Alkyne Reductive Cross-Coupling: Spiroketals through the Union of Homoallylic Alcohols. *J. Am. Soc. Chem.* **132**, 7602-7604 (2010).

10 Zhang, Y.-D. *et al.* Highly Regioselective Cobalt-Catalyzed Hydroboration of Internal Alkynes. *Angew. Chem. Int. Ed.* **61**, e202208473 (2022).

11 Soderquist, J. A., Martinez, J., Oyola, Y. & Kock, I. Novel route to carboxylic acids via the DCME reaction. *Tetrahedron Lett.* **45**, 5541-5543 (2004)

12 Corey, E. J. & Seibel, W. L. A simple stereoselective synthesis of Z-γ-bisabolene. *Tetrahedron Lett.* **27**, 909-910 (1986).

13 Jacob, P., III & Brown, H. C. A Grignard-like addition of B-alkenyl-9-borabicyclo[3.3.1]nonanes to aldehydes. A novel synthesis of allylic alcohols with defined stereochemistry. *J. Org. Chem.* **42**, 579-580 (1977).

14 Negishi, E.-i., Zhang, Y. & Bagheri, V. Highly stereoselective synthesis of exocyclic alkenes via cyclialkylation. *Tetrahedron Lett.* **28**, 5793-5796 (1987).

15 Anastasia, L., Dumond, Yves R. & Negishi, E.-i. Stereoselective Synthesis of Exocyclic Alkenes by Cu-Catalyzed Allylmagnesiation, Pd-Catalyzed Alkylation, and Ru-Catalyzed Ring-Closing Metathesis: Highly Stereoselective Synthesis of (Z)- and (E)-γ-Bisabolenes. *Eur. J. Org. Chem.* **2001**, 3039-3043 (2001).

2.11 NMR Spectra

^{1}H NMR (500 MHz, CD_2Cl_2) of **5a**

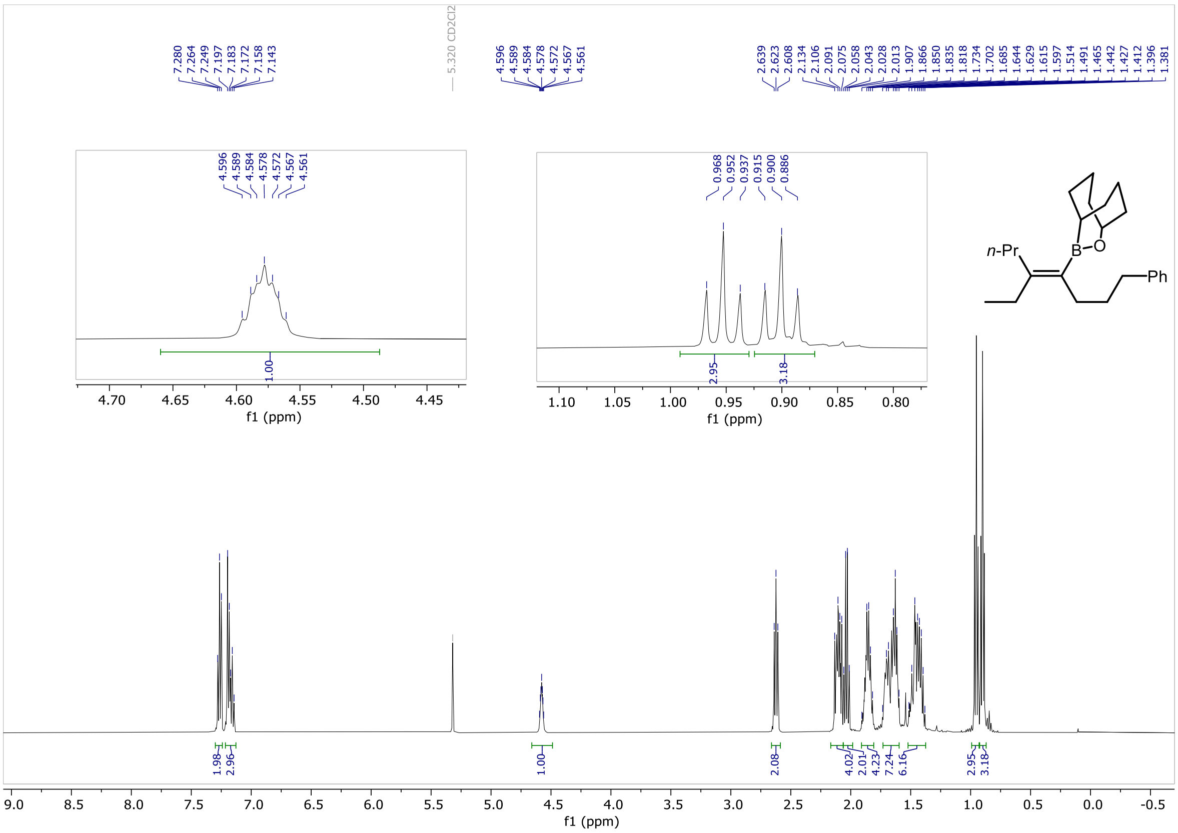

^{13}C NMR (500 MHz, CD_2Cl_2) of **5a**

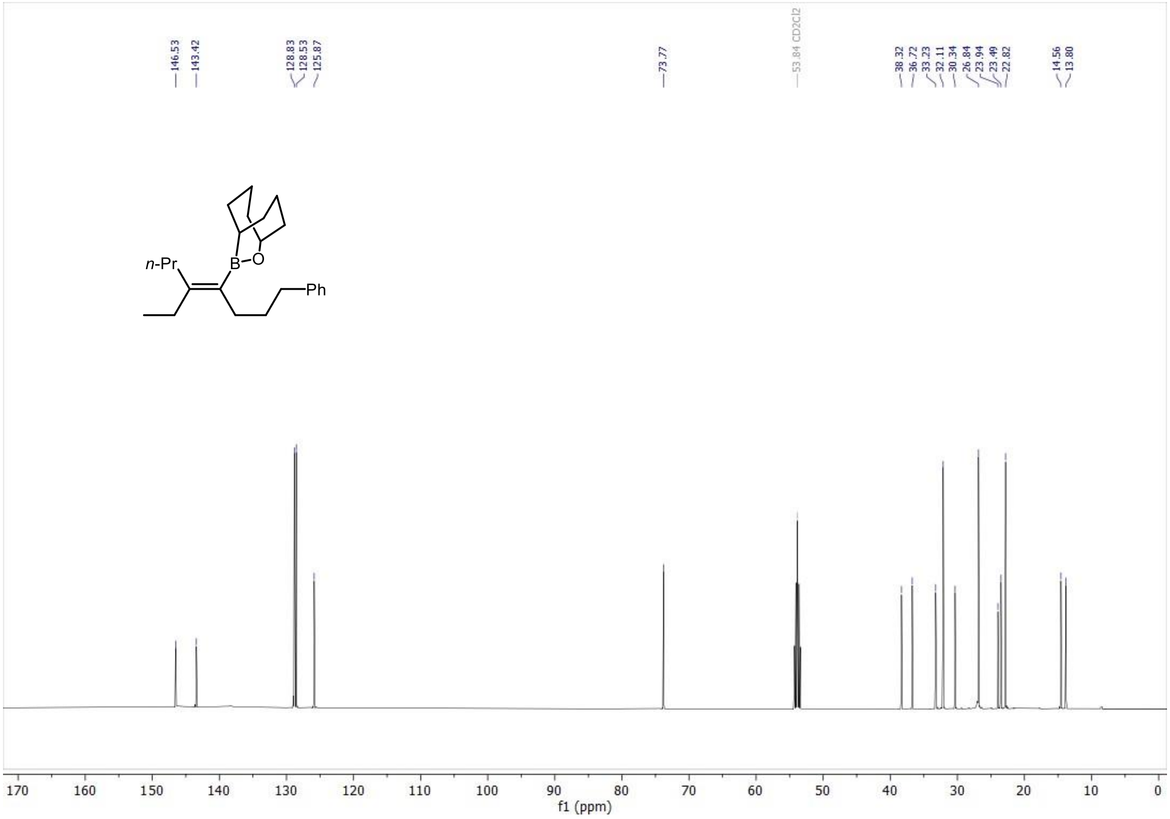

^{1}H NMR (400 MHz, CD_2Cl_2) of **5b**

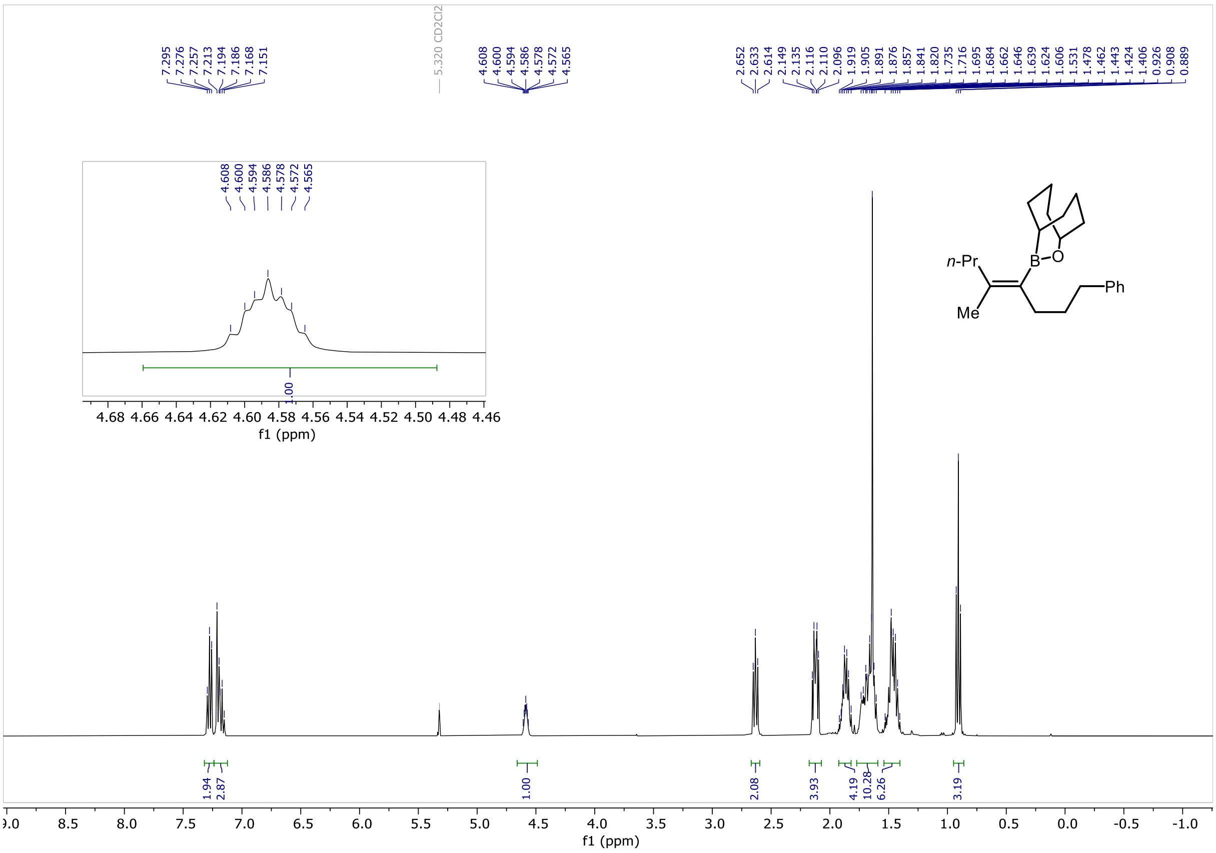

^{13}C NMR (400 MHz, CD_2Cl_2) of **5b**

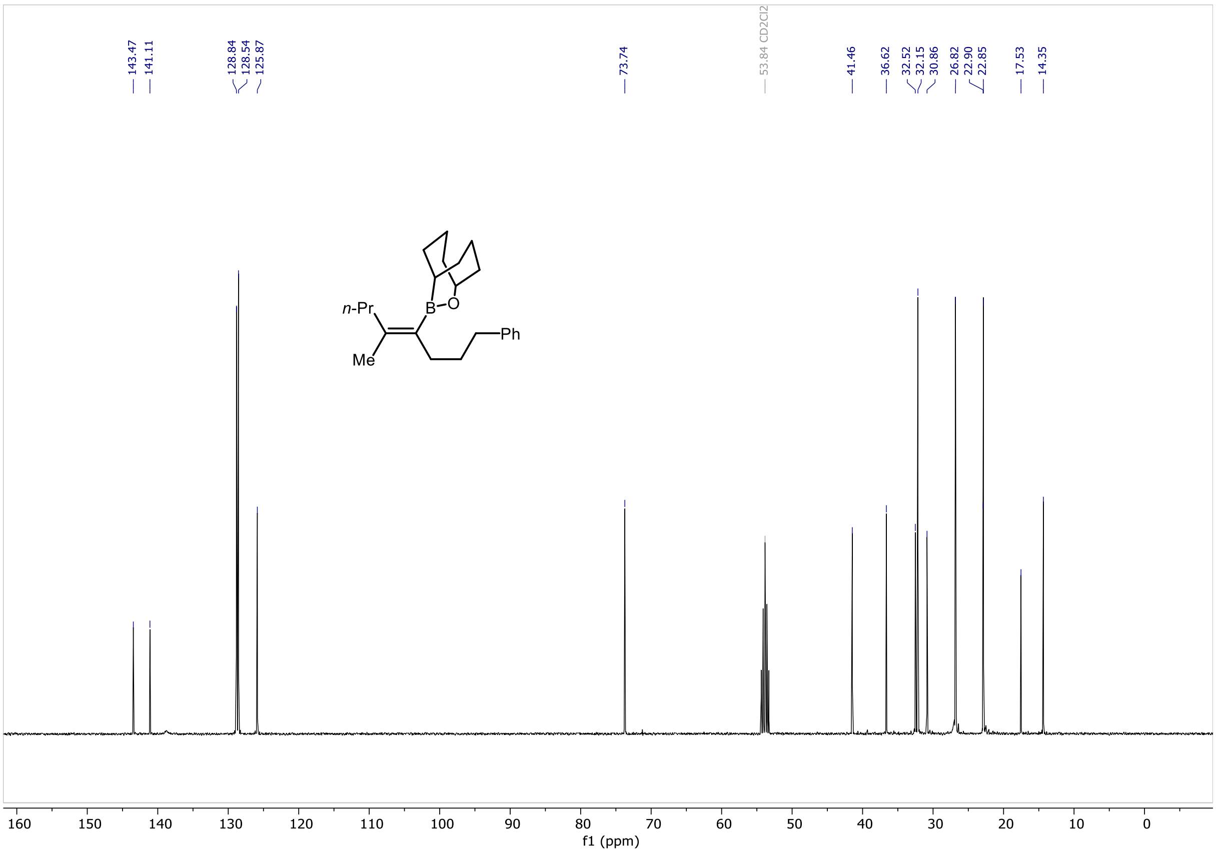

^{1}H NMR (400 MHz, CD_2Cl_2) of **5c**

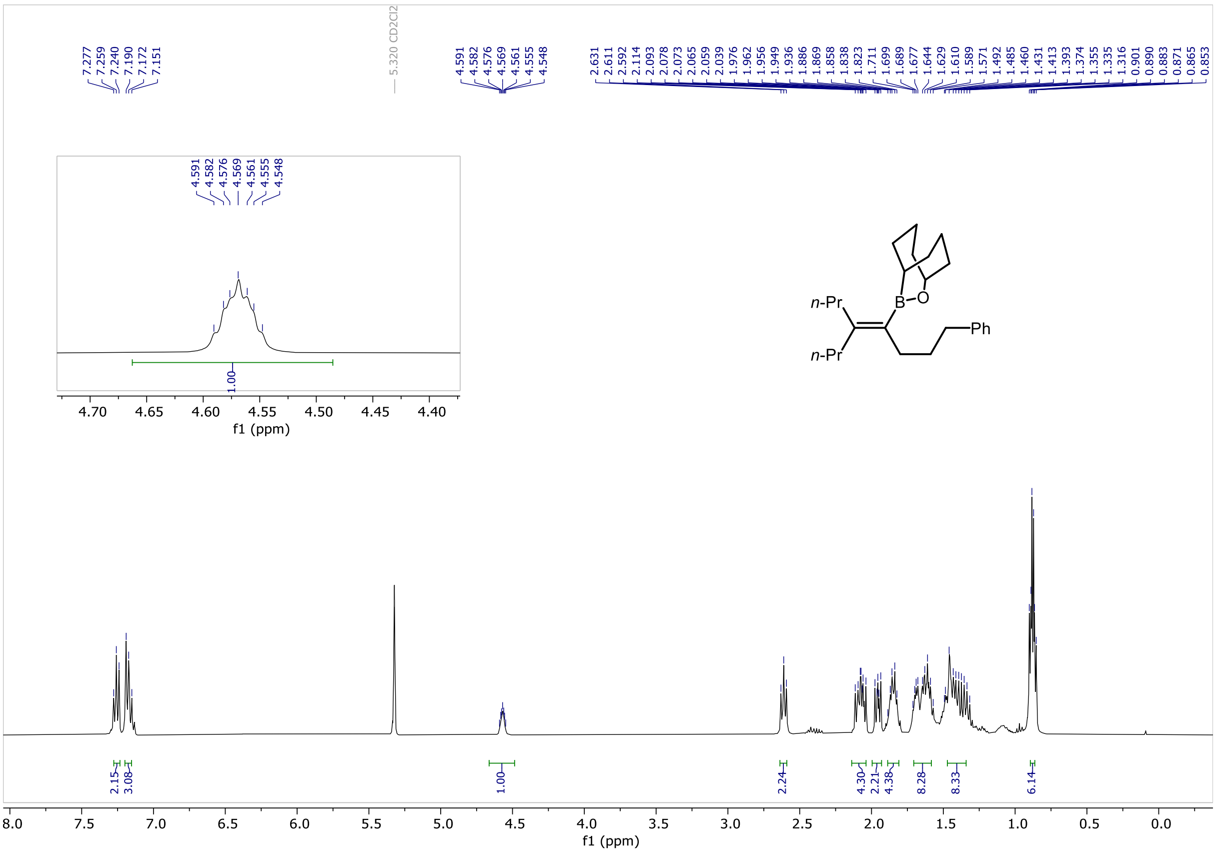

^{13}C NMR (400 MHz, CD_2Cl_2) of **5c**

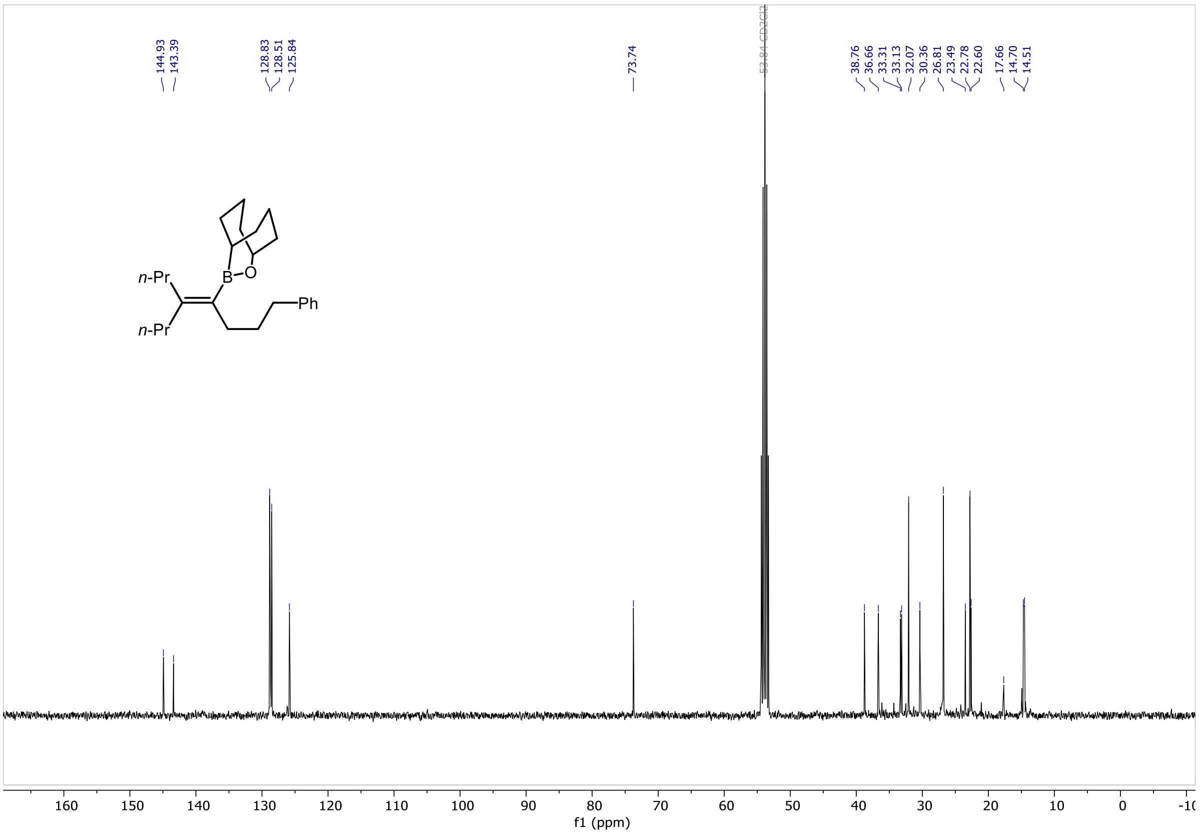

^{1}H NMR (400 MHz, CD_2Cl_2) of **5d**

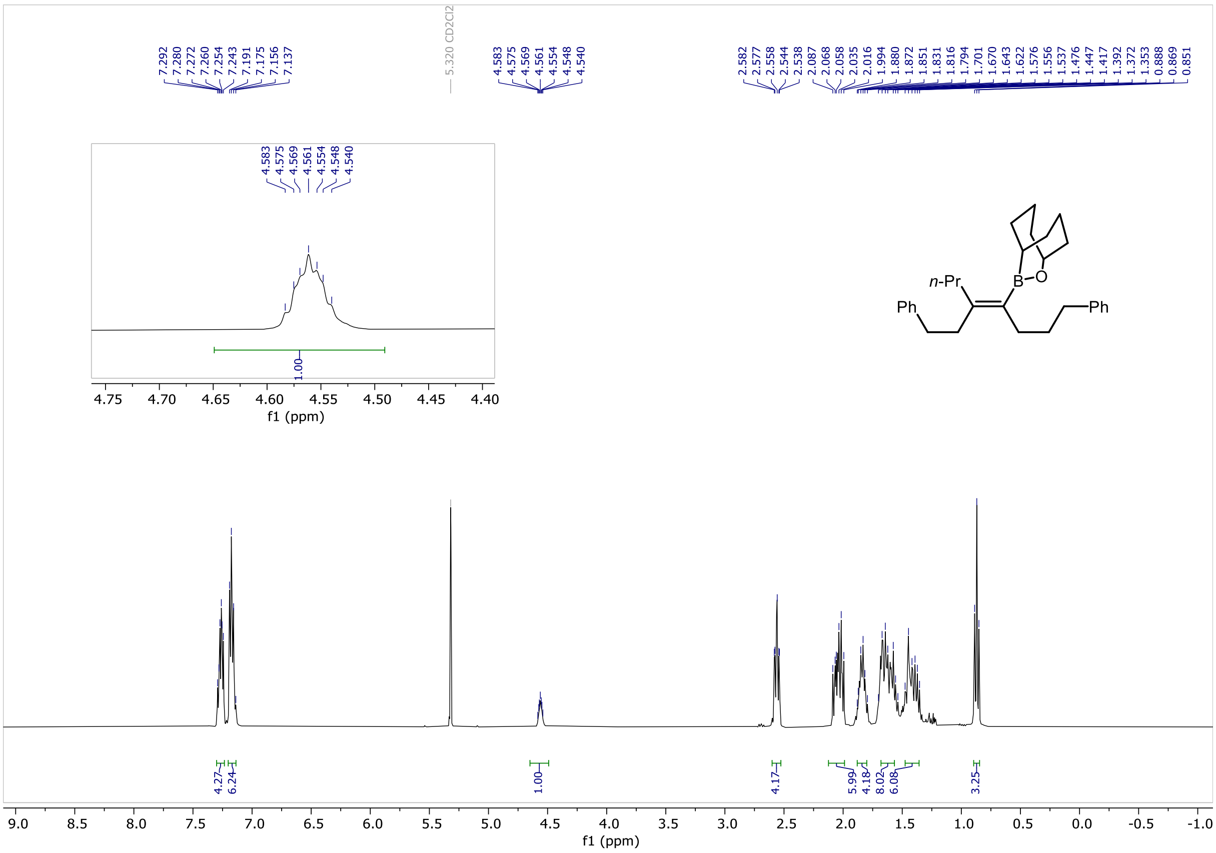

^{13}C NMR (400 MHz, CD_2Cl_2) of **5d**

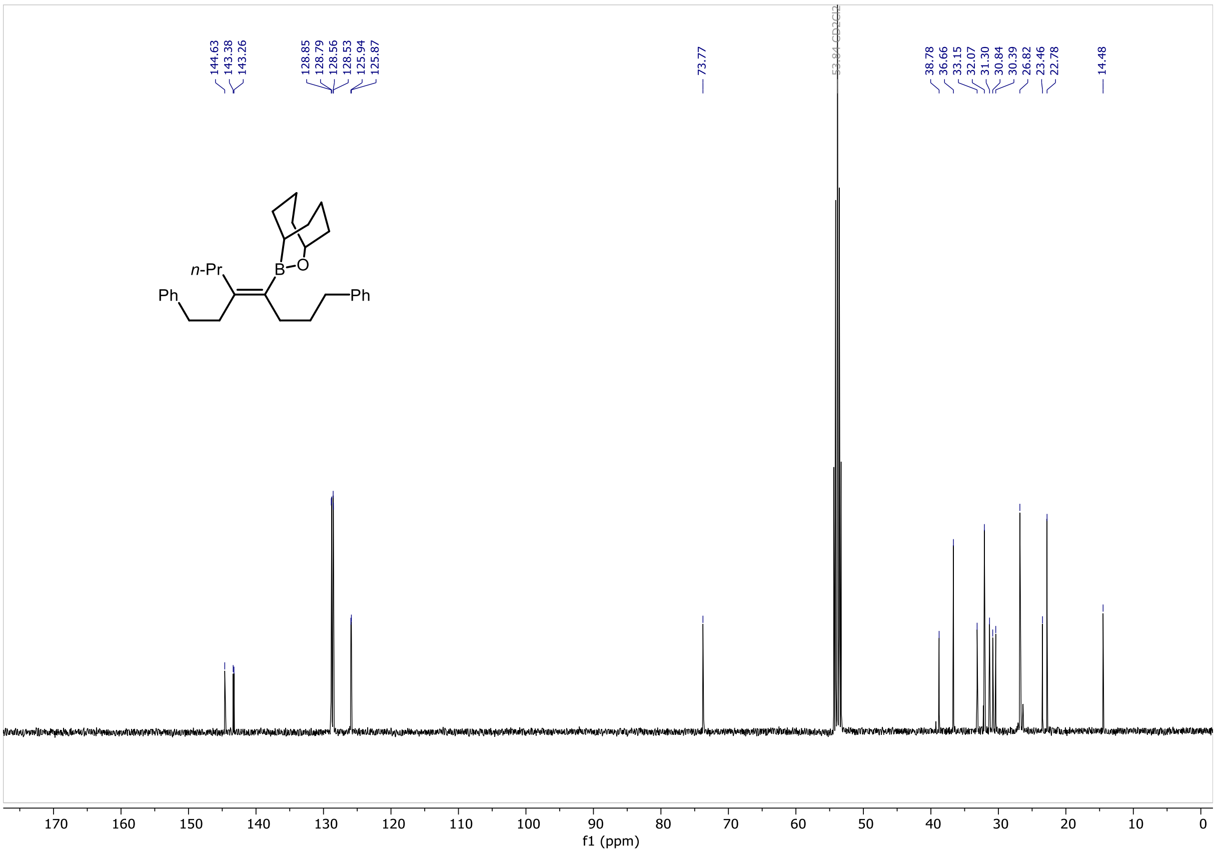

^{1}H NMR (400 MHz, CD_2Cl_2) of **5e**

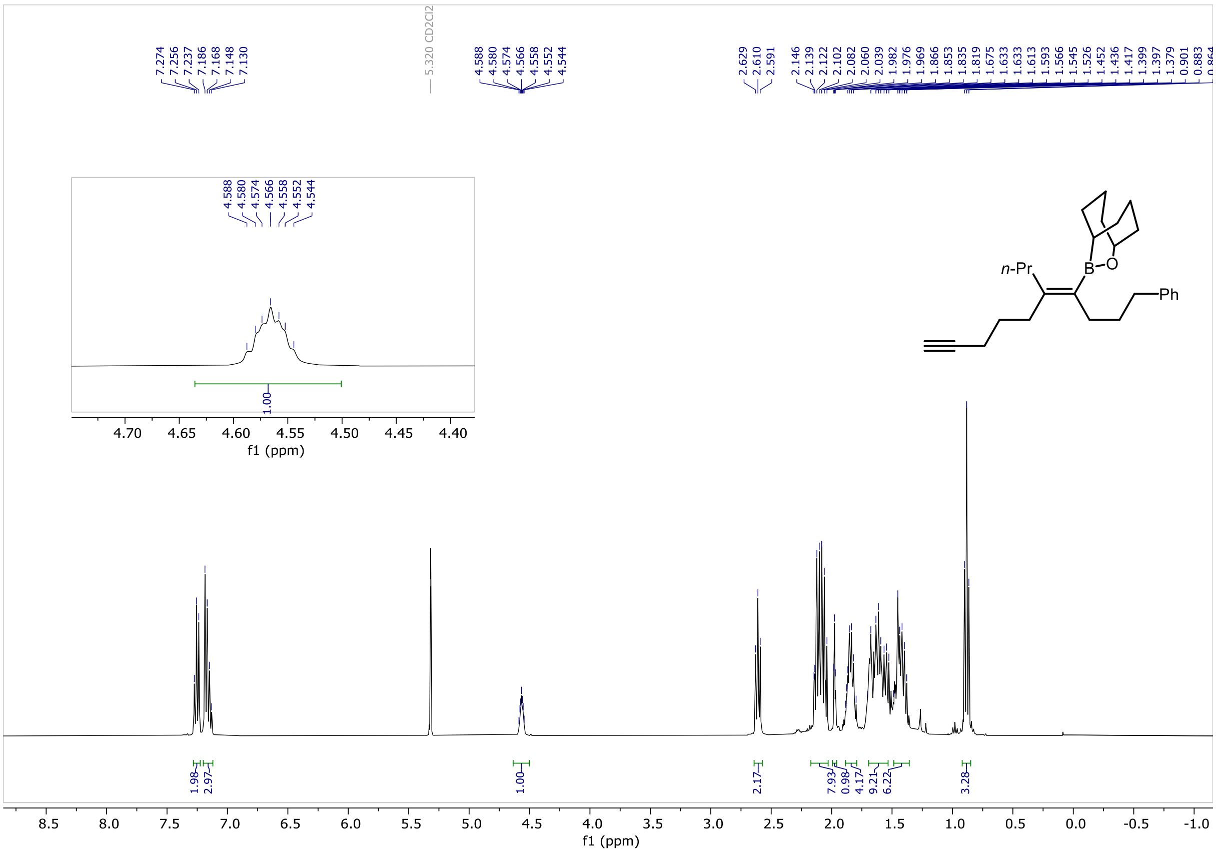

^{13}C NMR (400 MHz, CD_2Cl_2) of **5e**

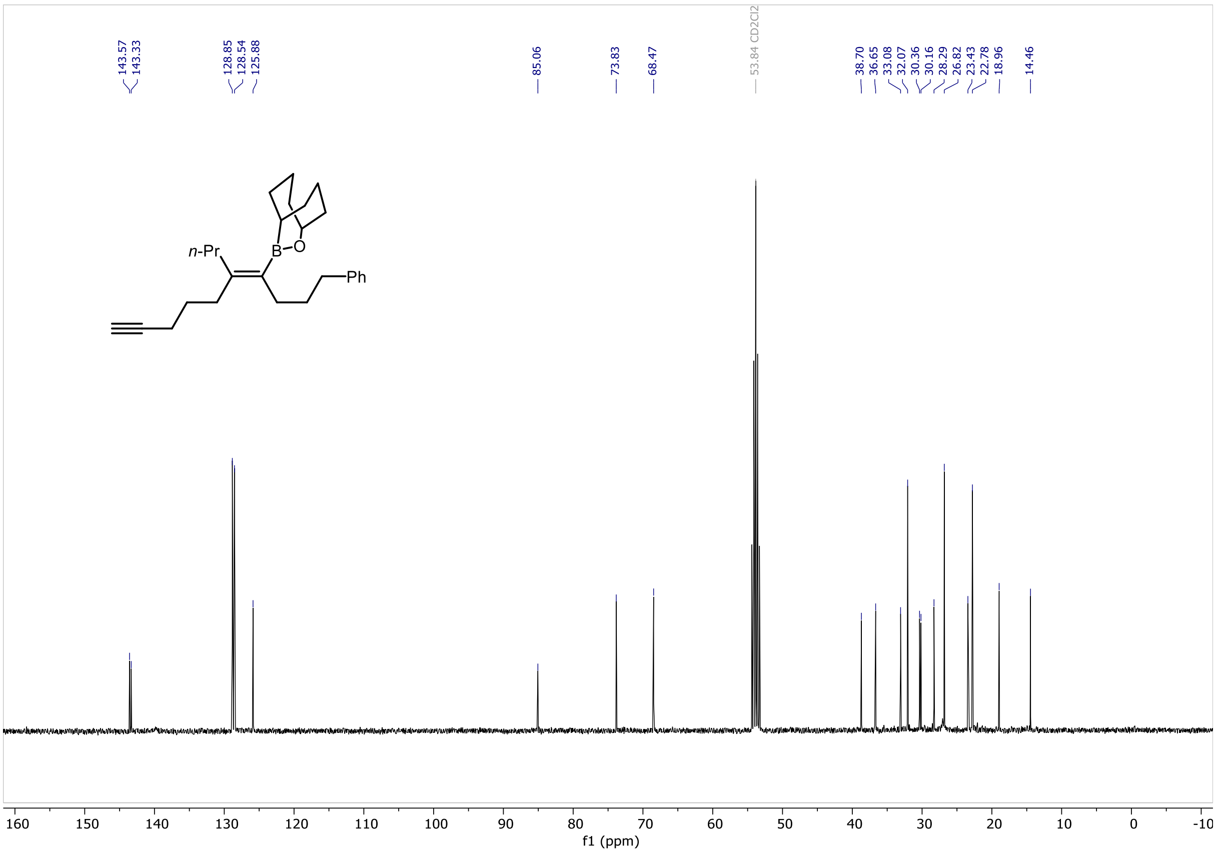

^{1}H NMR (400 MHz, CD_2Cl_2) of **5f**

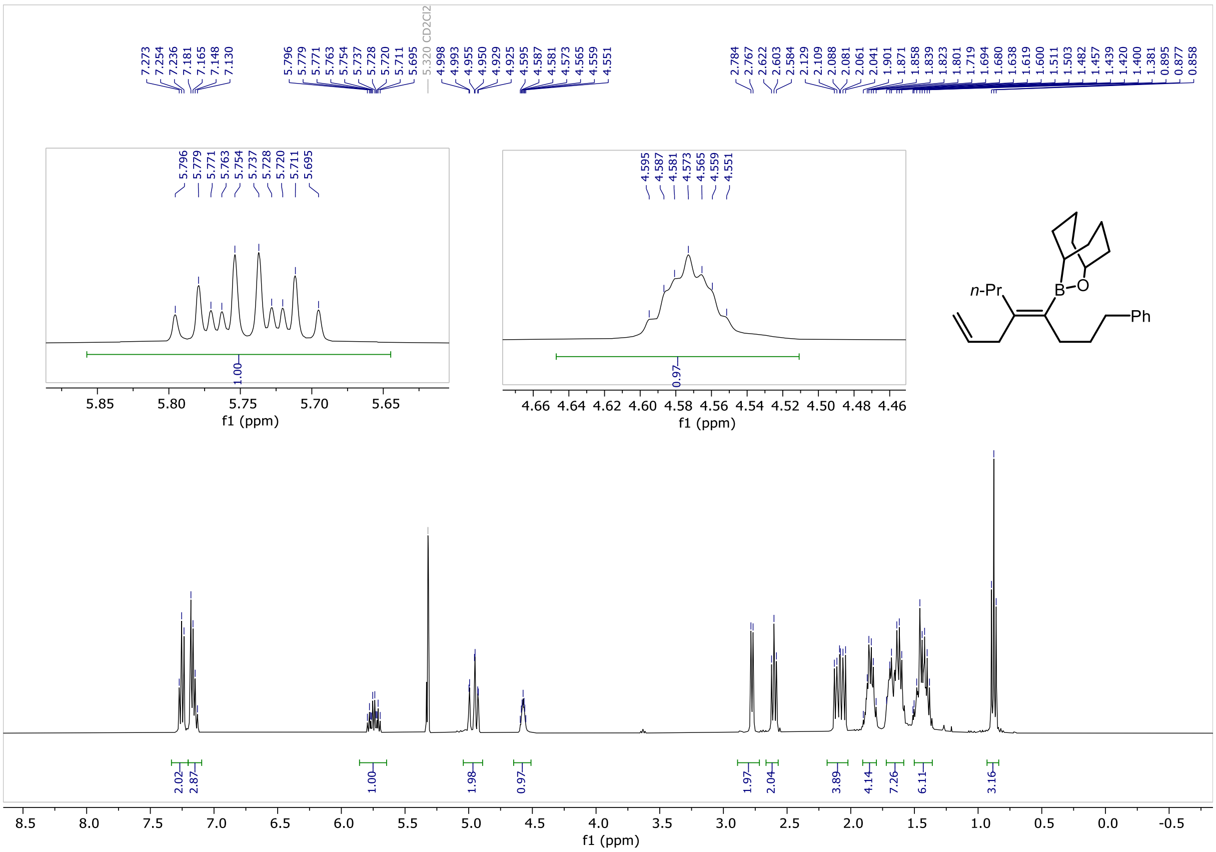

^{13}C NMR (400 MHz, CD_2Cl_2) of **5f**

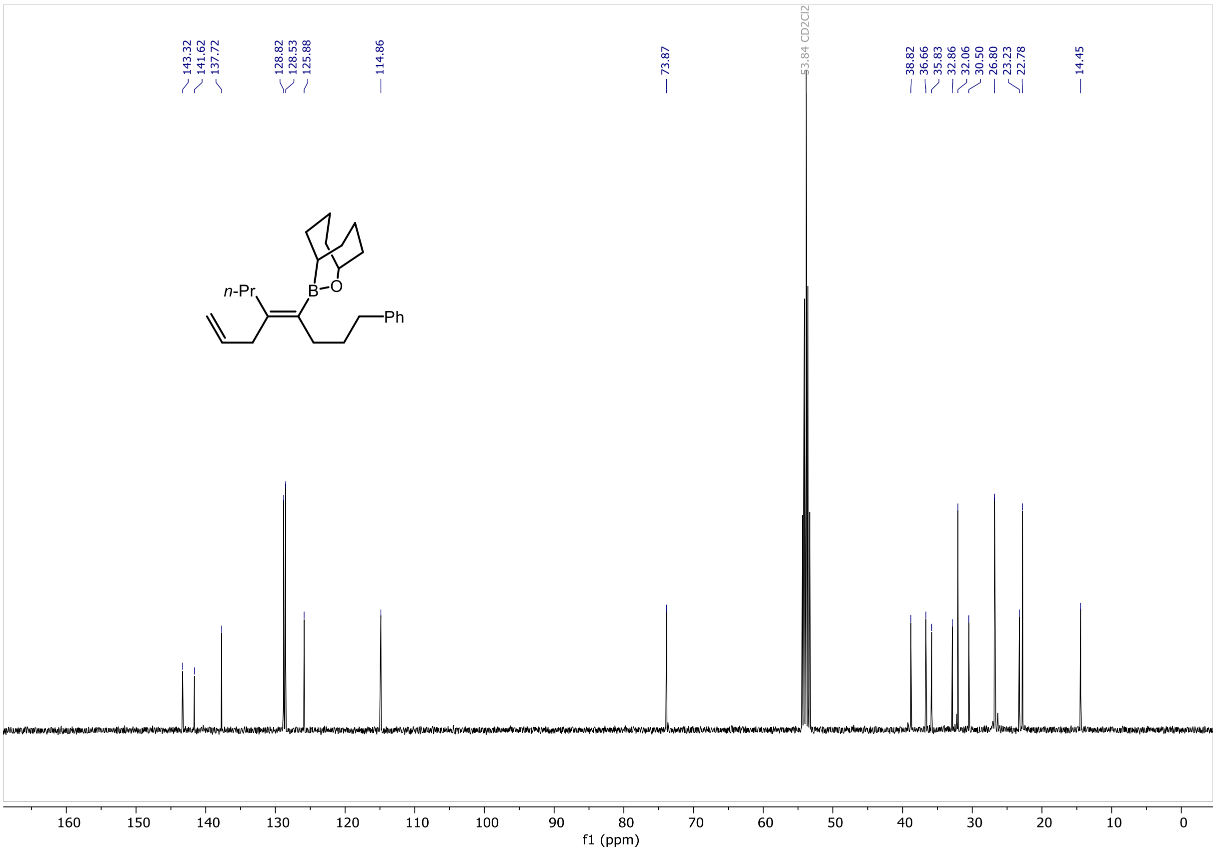

^{1}H NMR (400 MHz, CD_2Cl_2) of **5g**

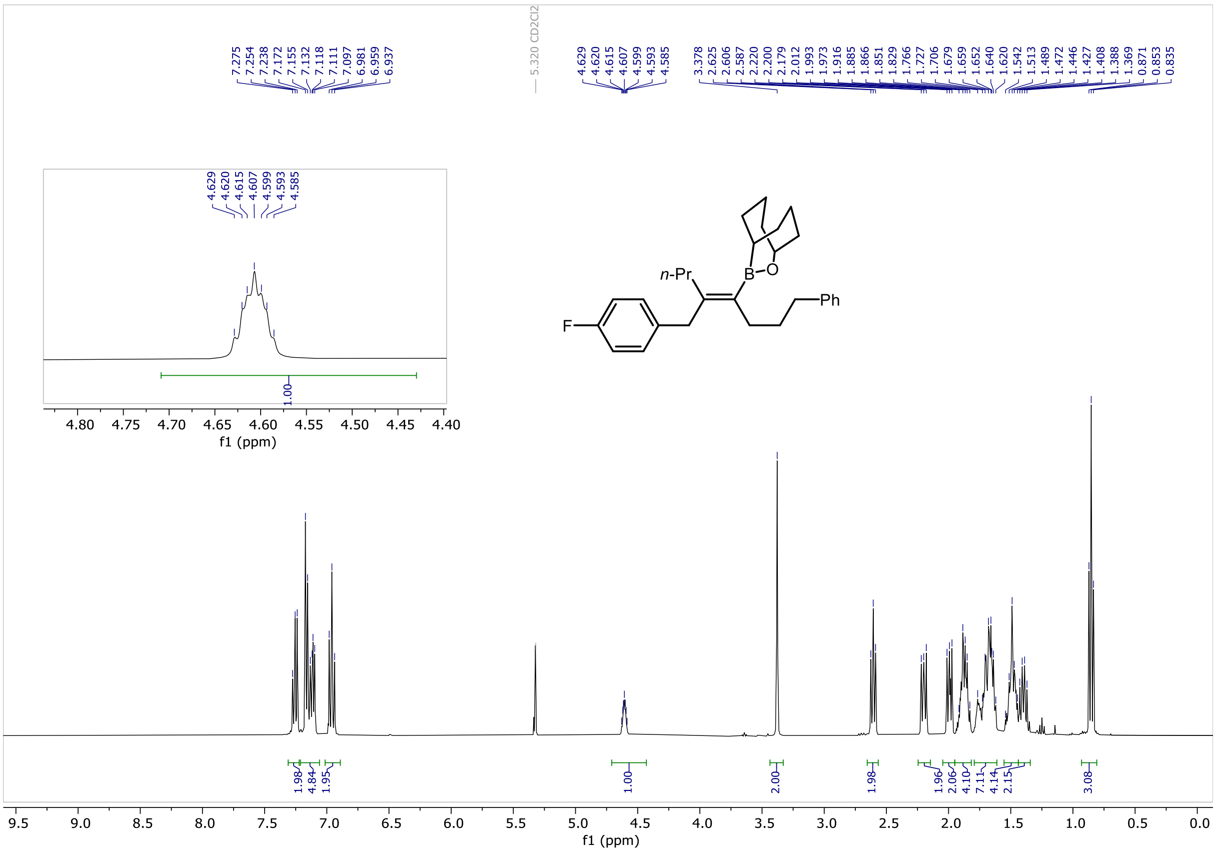

^{13}C NMR (400 MHz, CD_2Cl_2) of **5g**

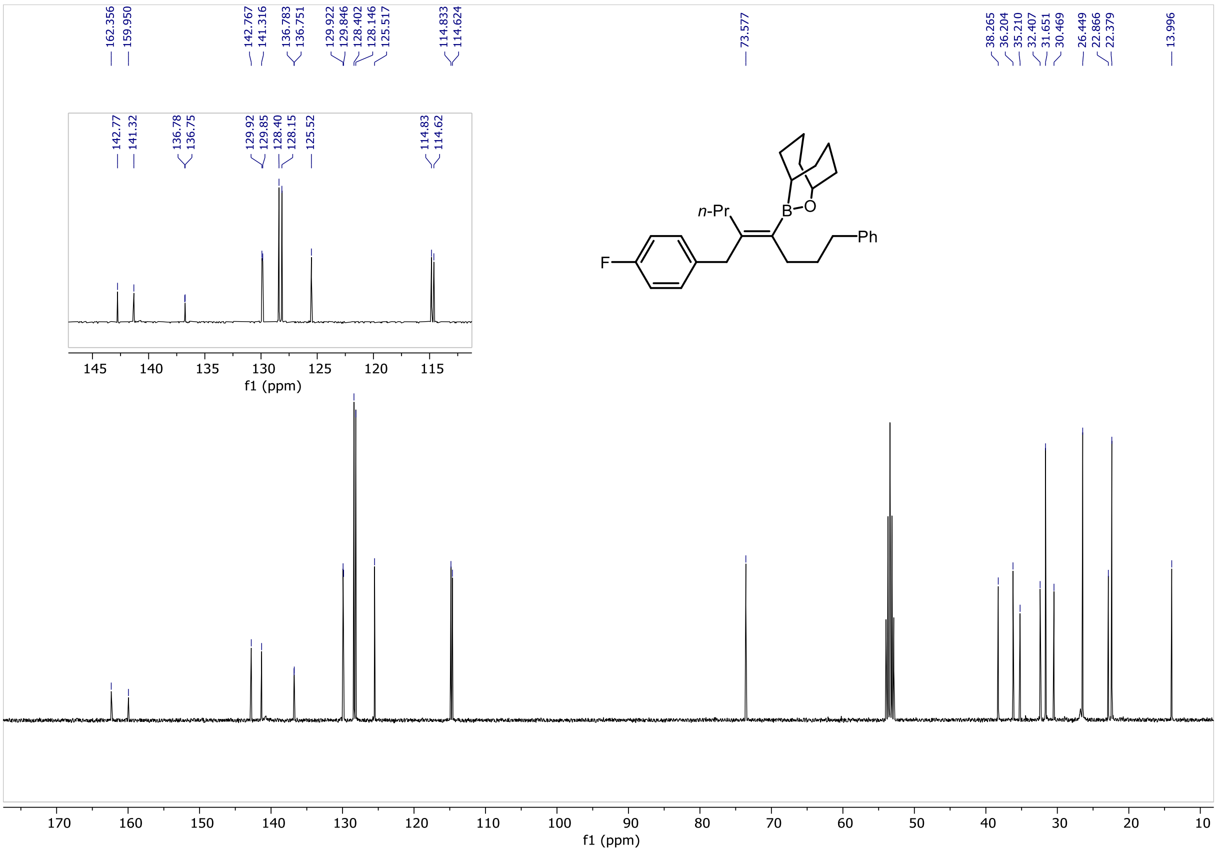

^{19}F NMR (377 MHz, CD_2Cl_2) of **5g**

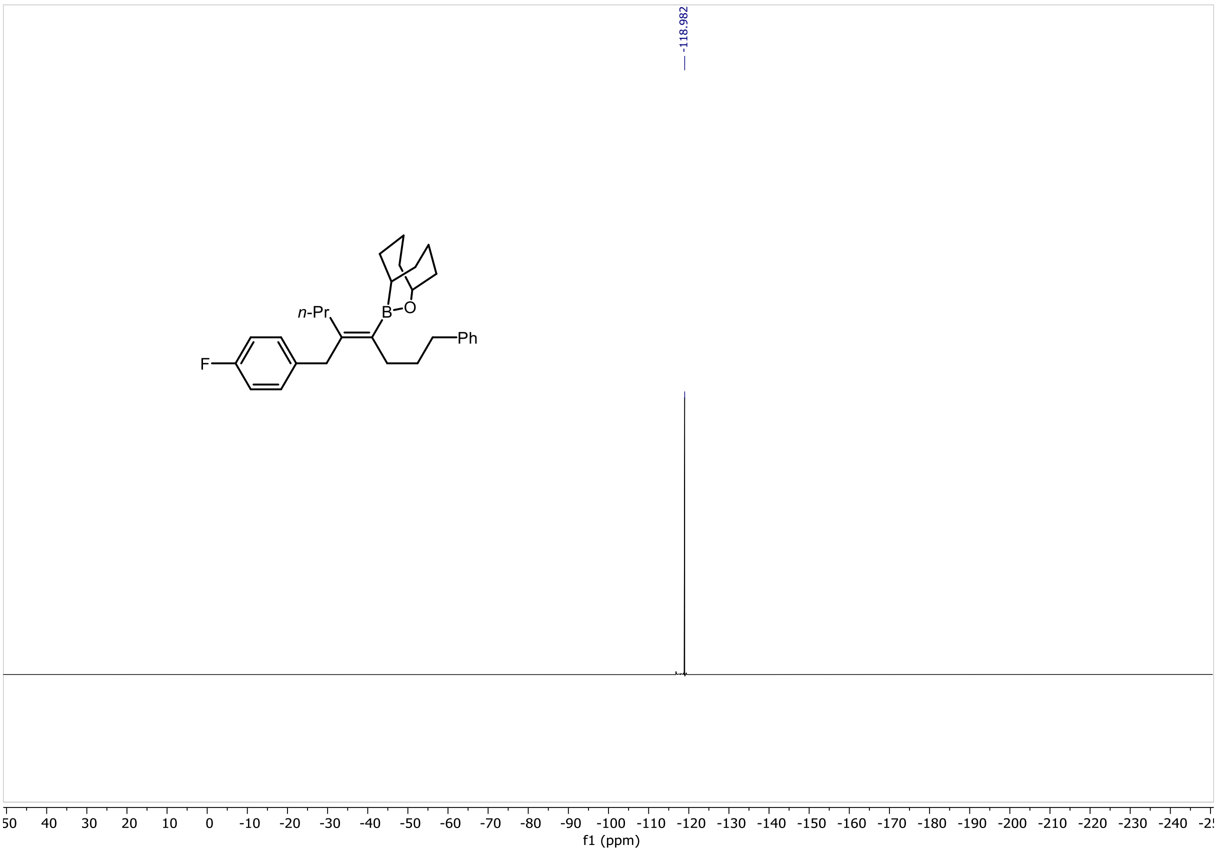

^{1}H NMR (400 MHz, CD_2Cl_2) of **5h**

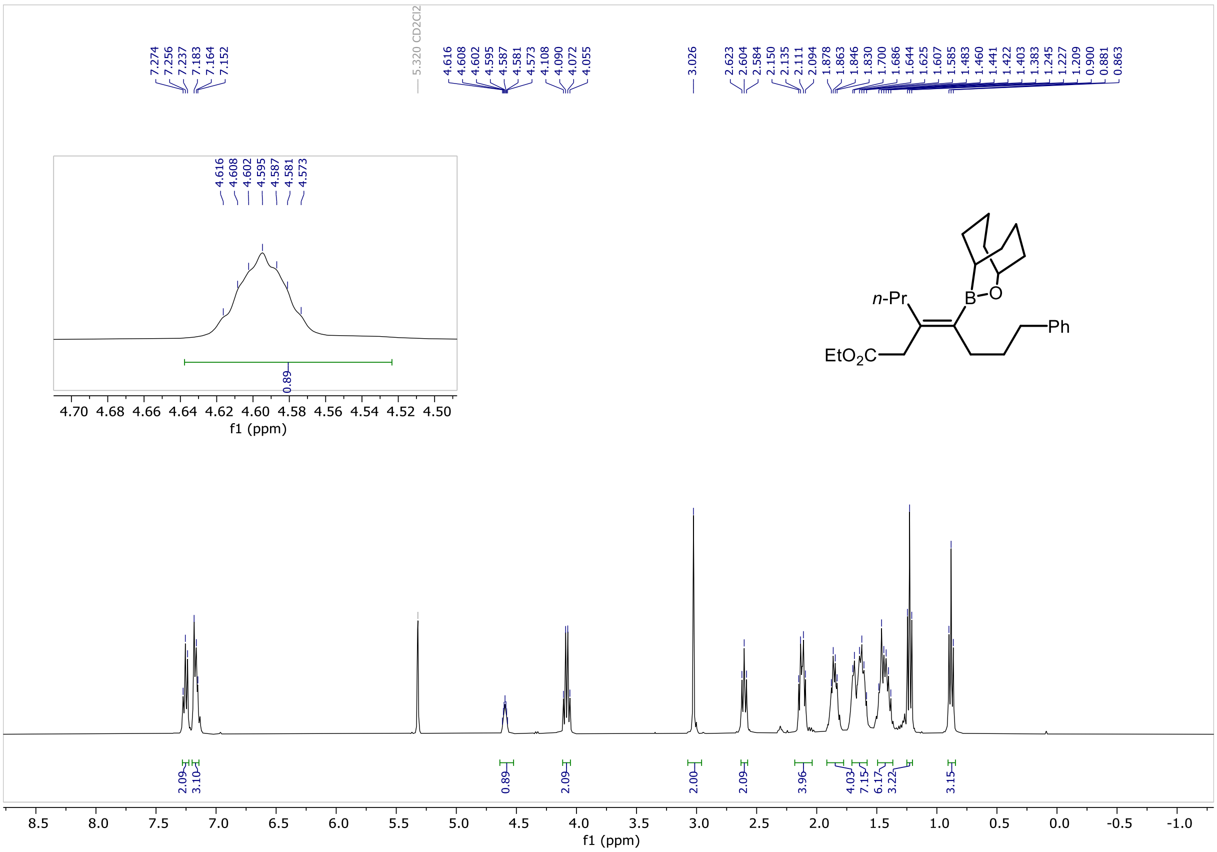

^{13}C NMR (400 MHz, CD_2Cl_2) of **5h**

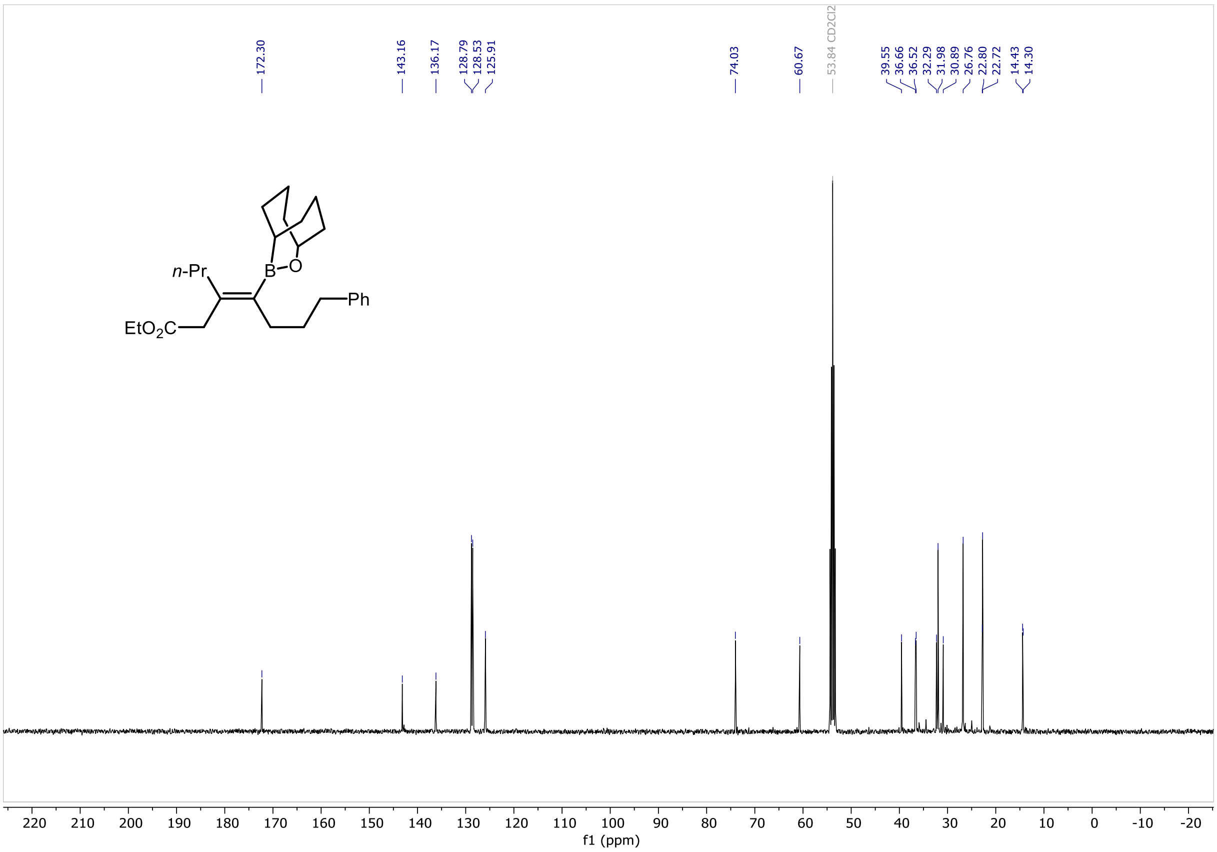

^{1}H NMR (400 MHz, CD_2Cl_2) of **5i**

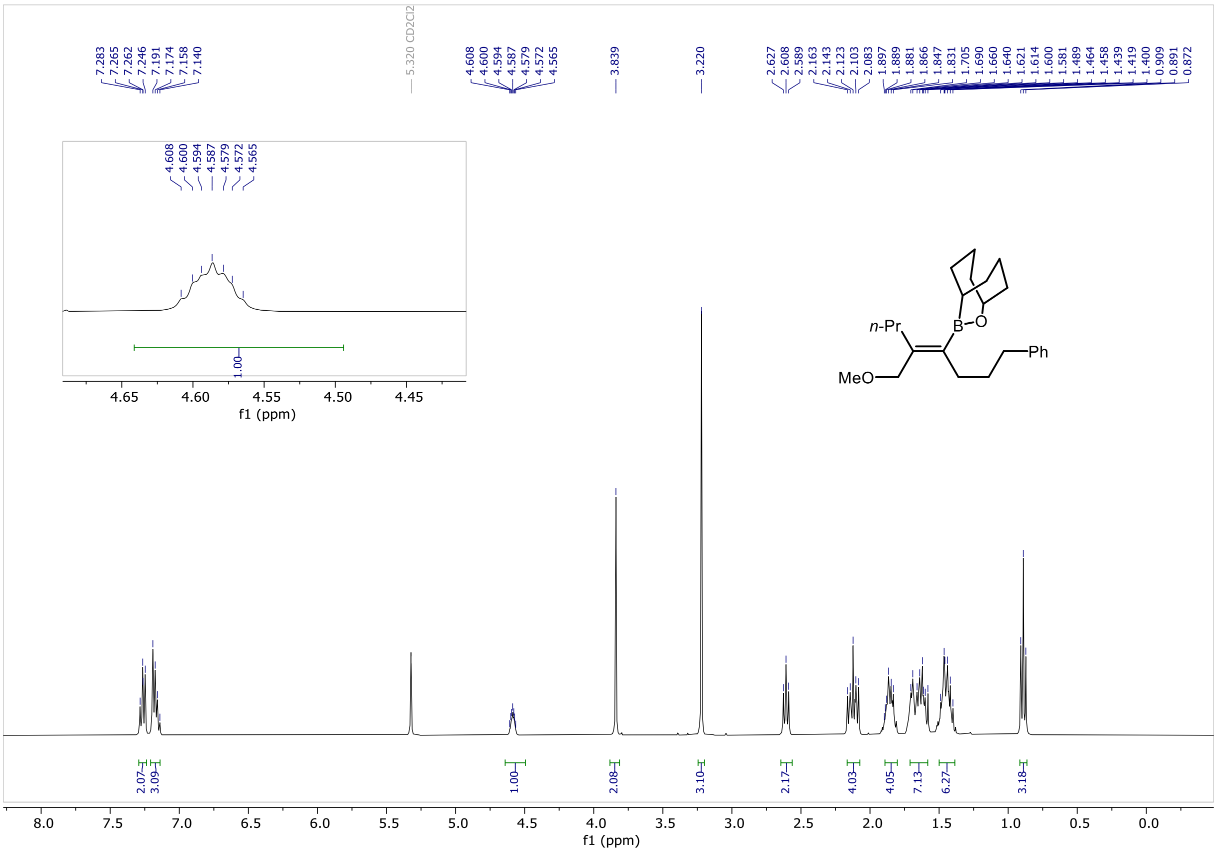

^{13}C NMR (400 MHz, CD_2Cl_2) of **5i**

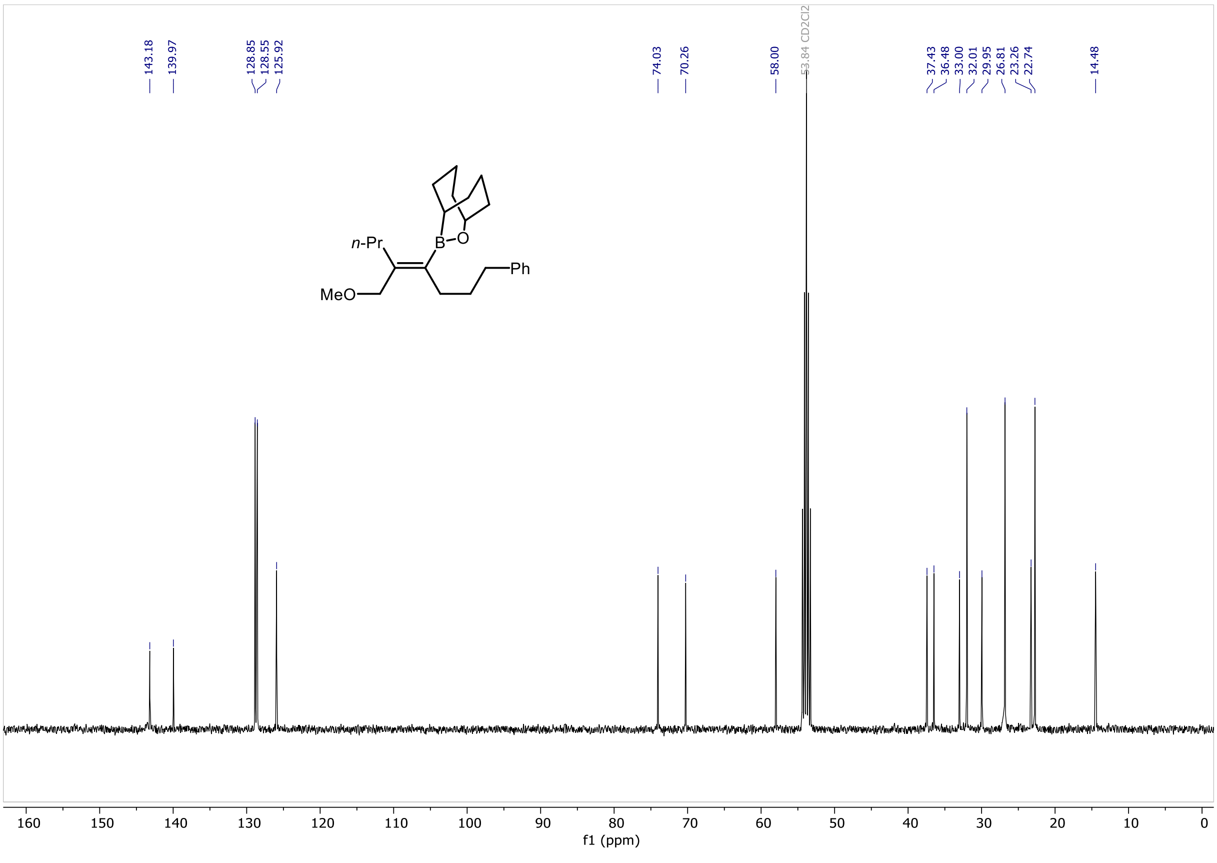

^{1}H NMR (400 MHz, CD_2Cl_2) of **5j**

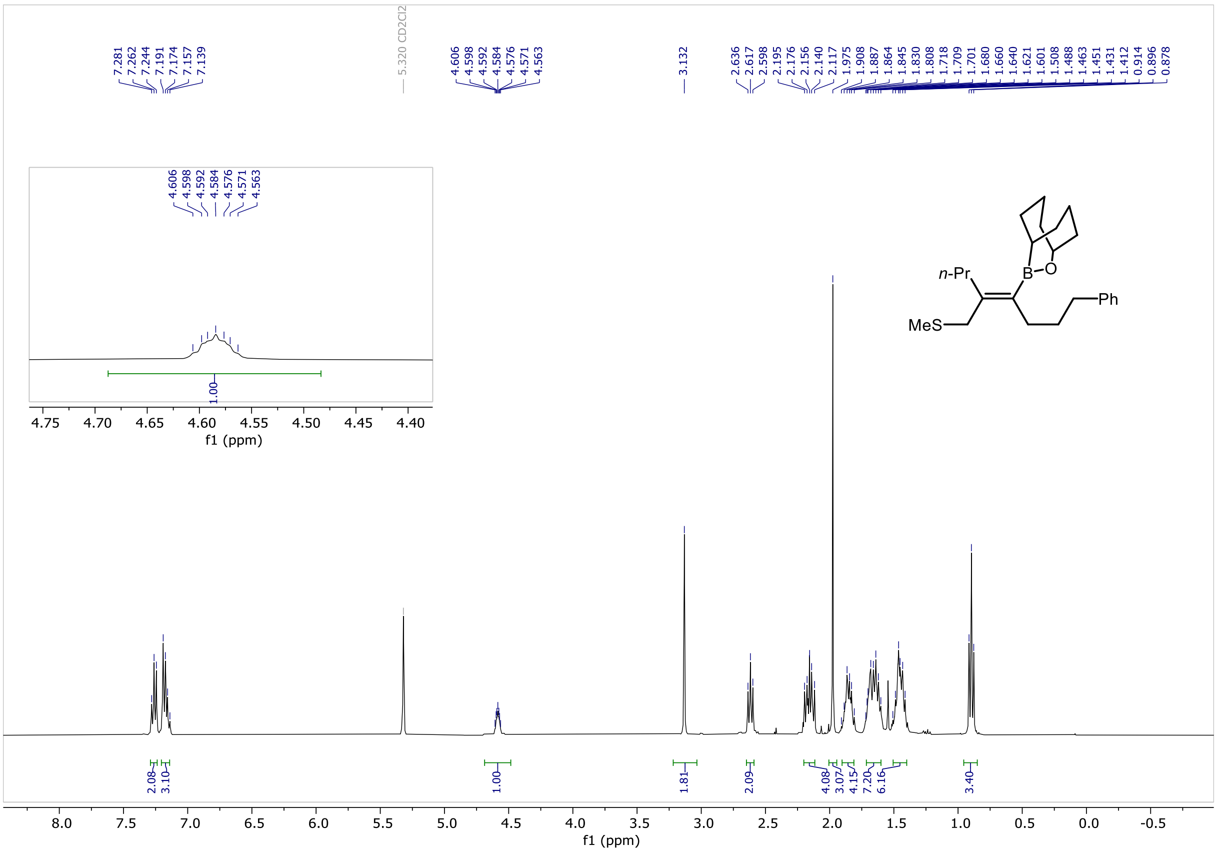

^{13}C NMR (400 MHz, CD_2Cl_2) of **5j**

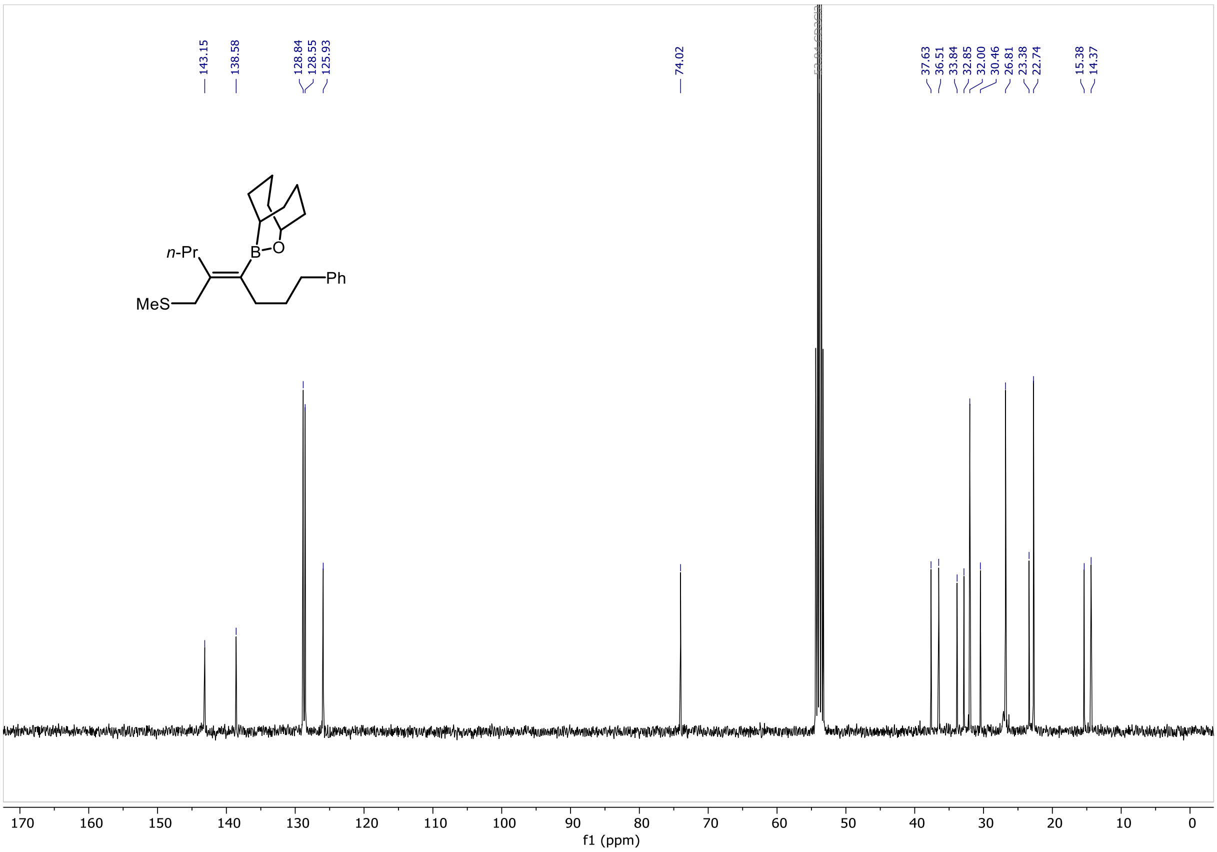

^{1}H NMR (400 MHz, C_6D_6) of **5k**

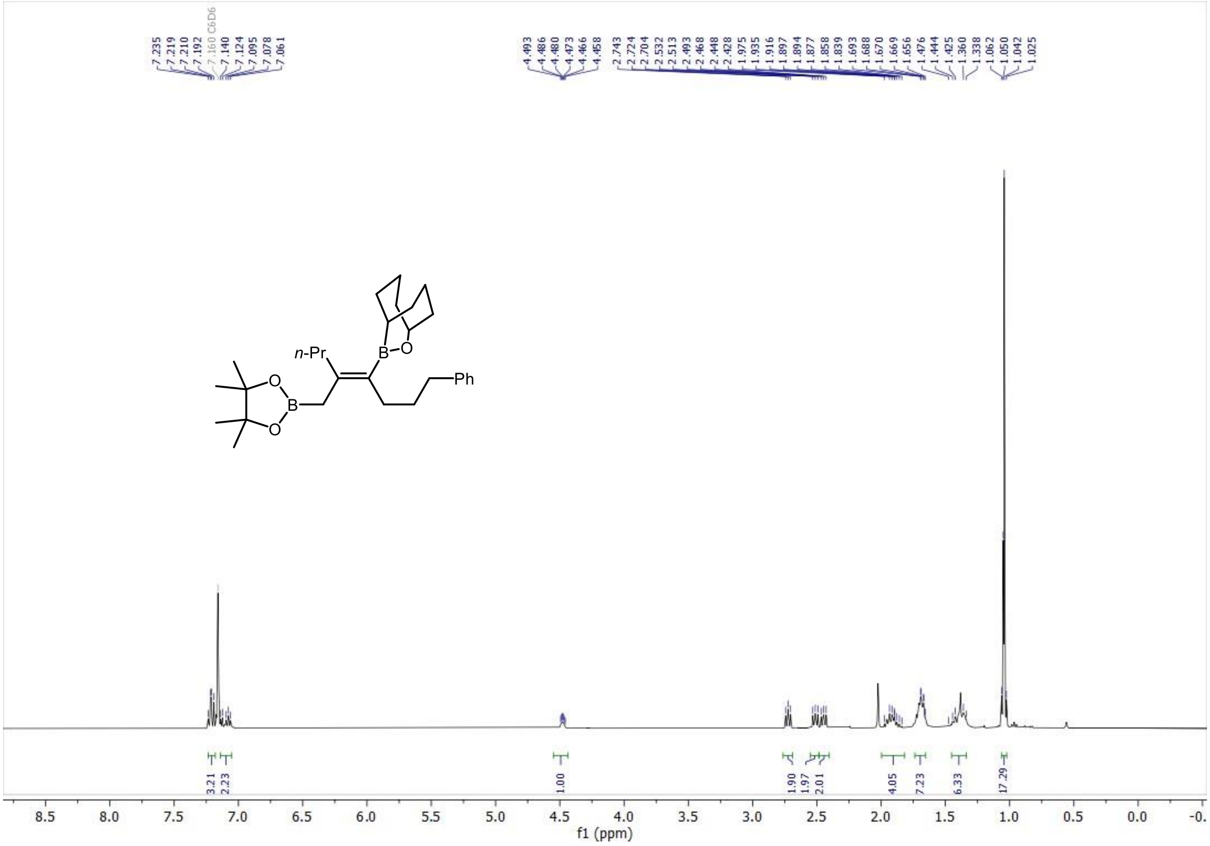

^{13}C NMR (400 MHz, C_6D_6) of **5k**

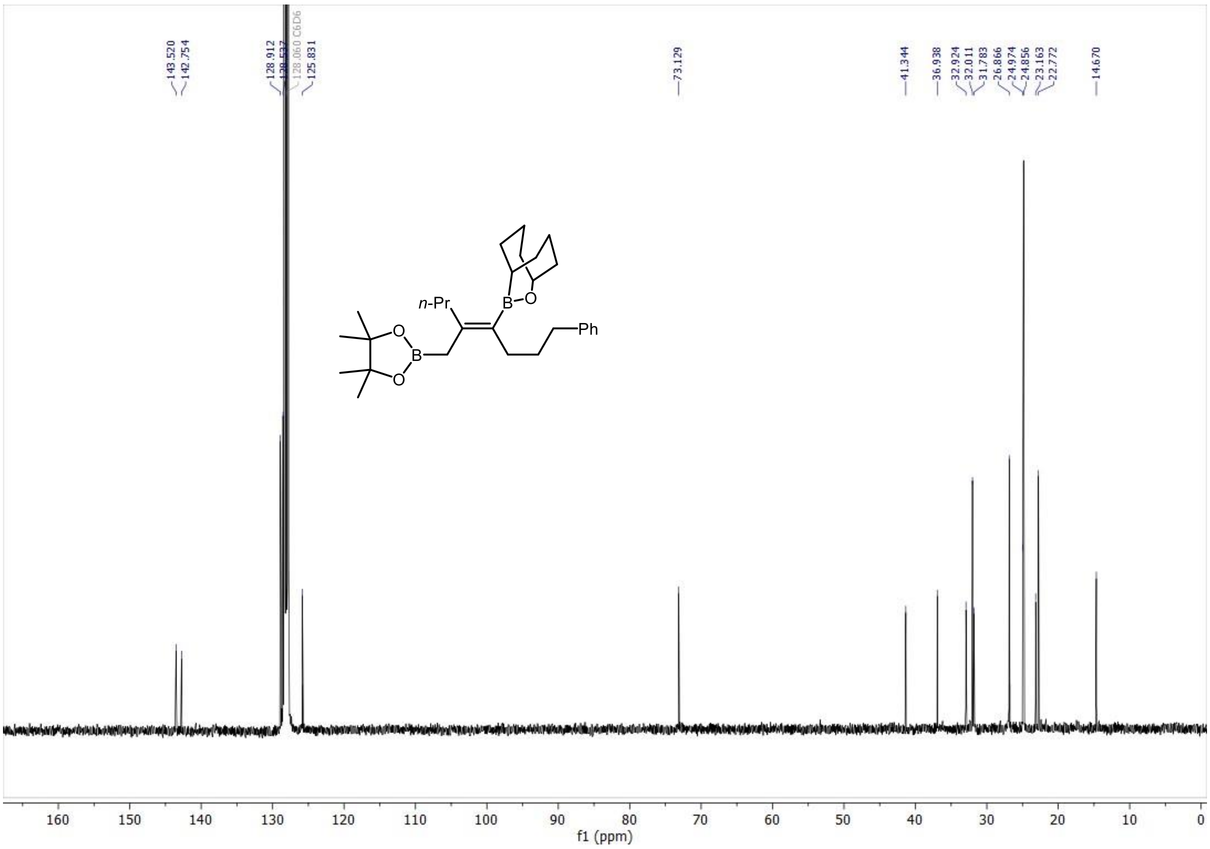

^{1}H NMR (400 MHz, CD_2Cl_2) of **5l**

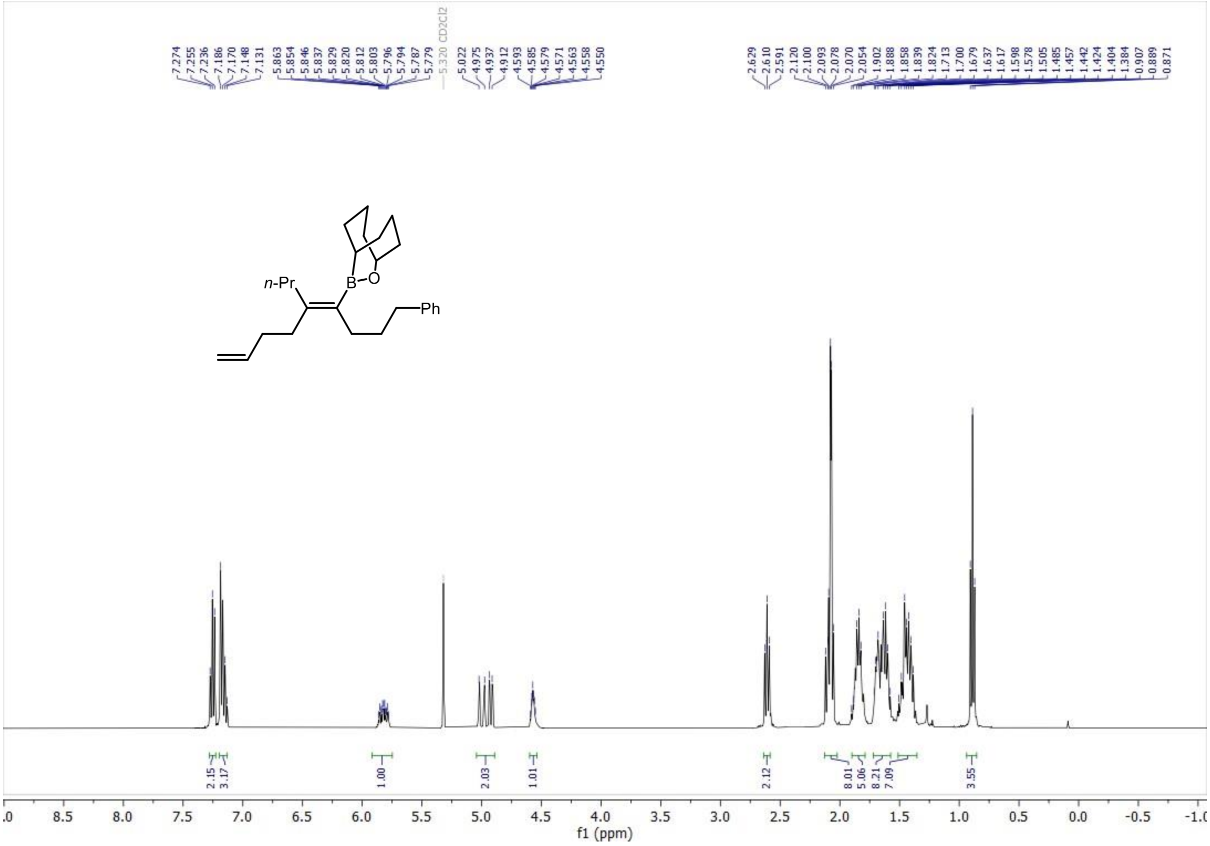

^{13}C NMR (400 MHz, CD_2Cl_2) of **5l**

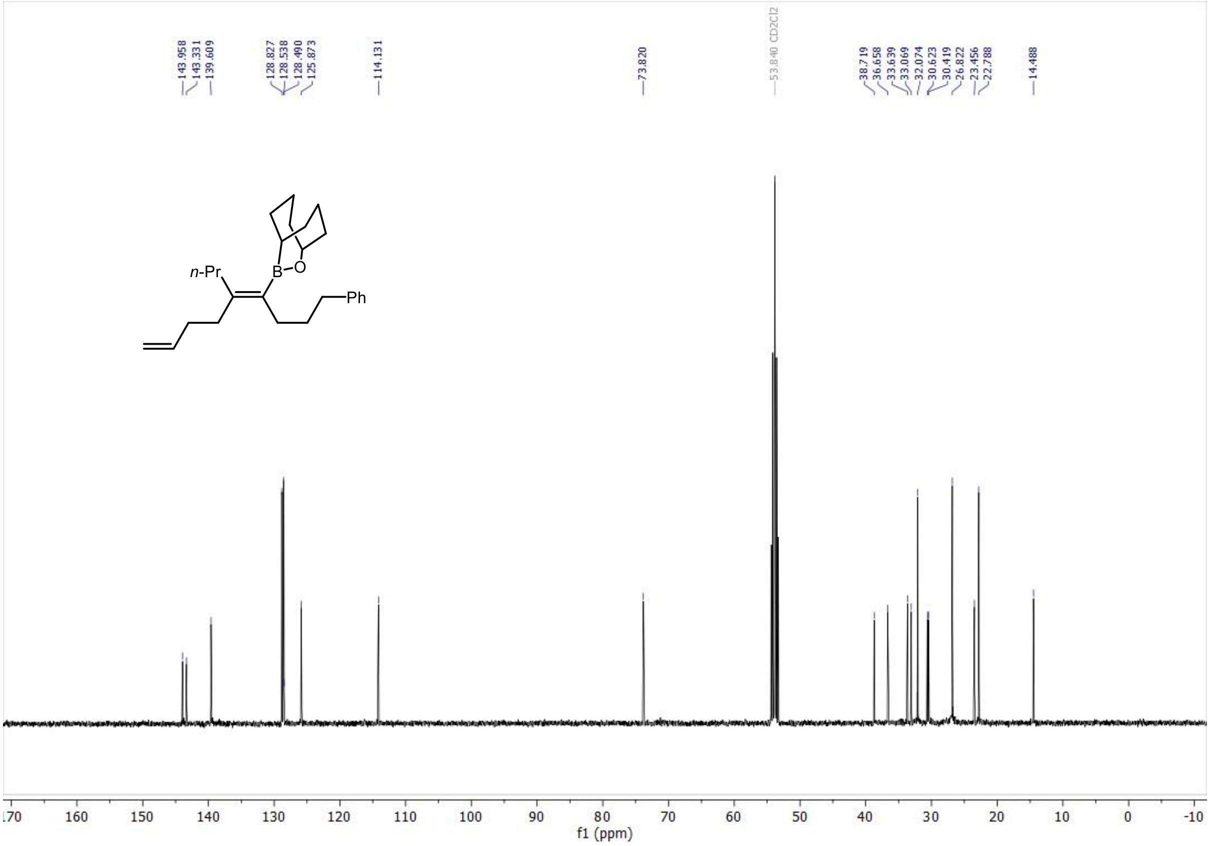

^{1}H NMR (400 MHz, CD_2Cl_2) of **5m**

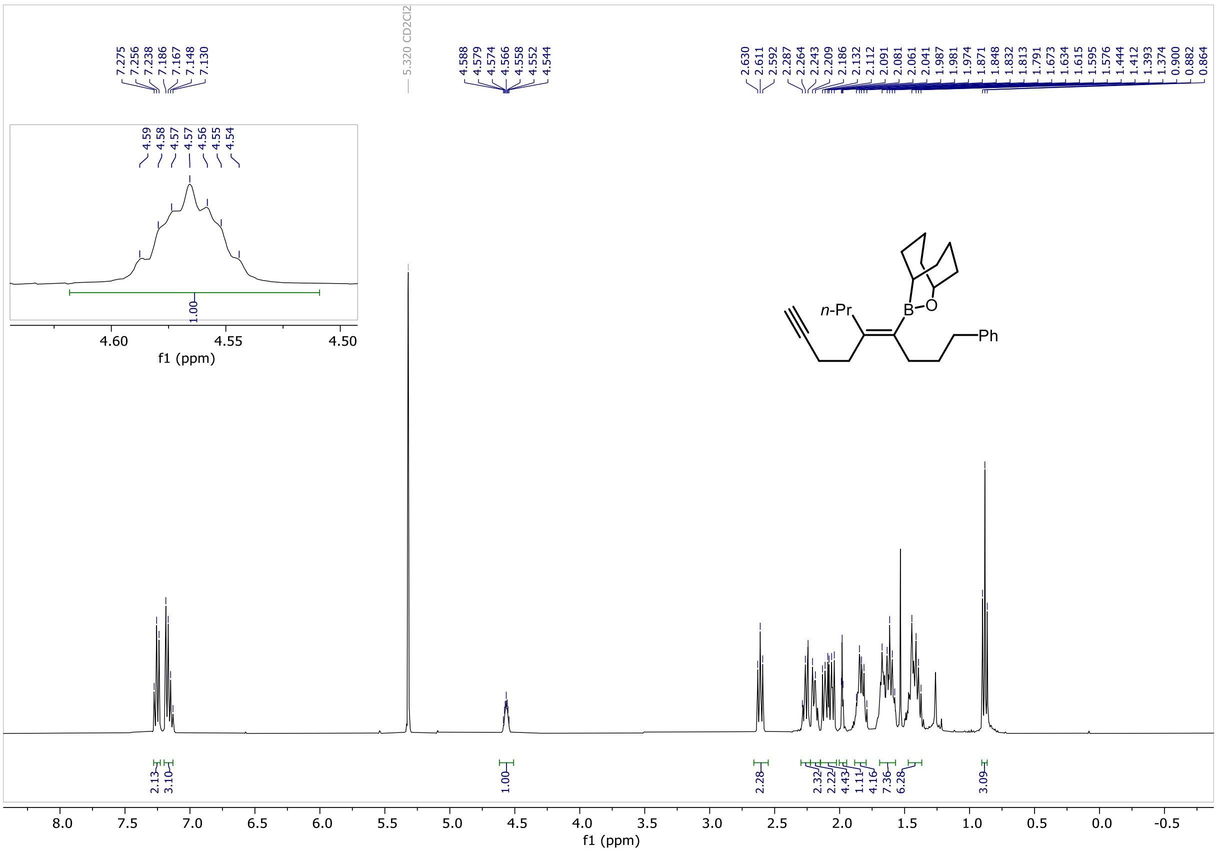

^{13}C NMR (400 MHz, CD_2Cl_2) of **5m**

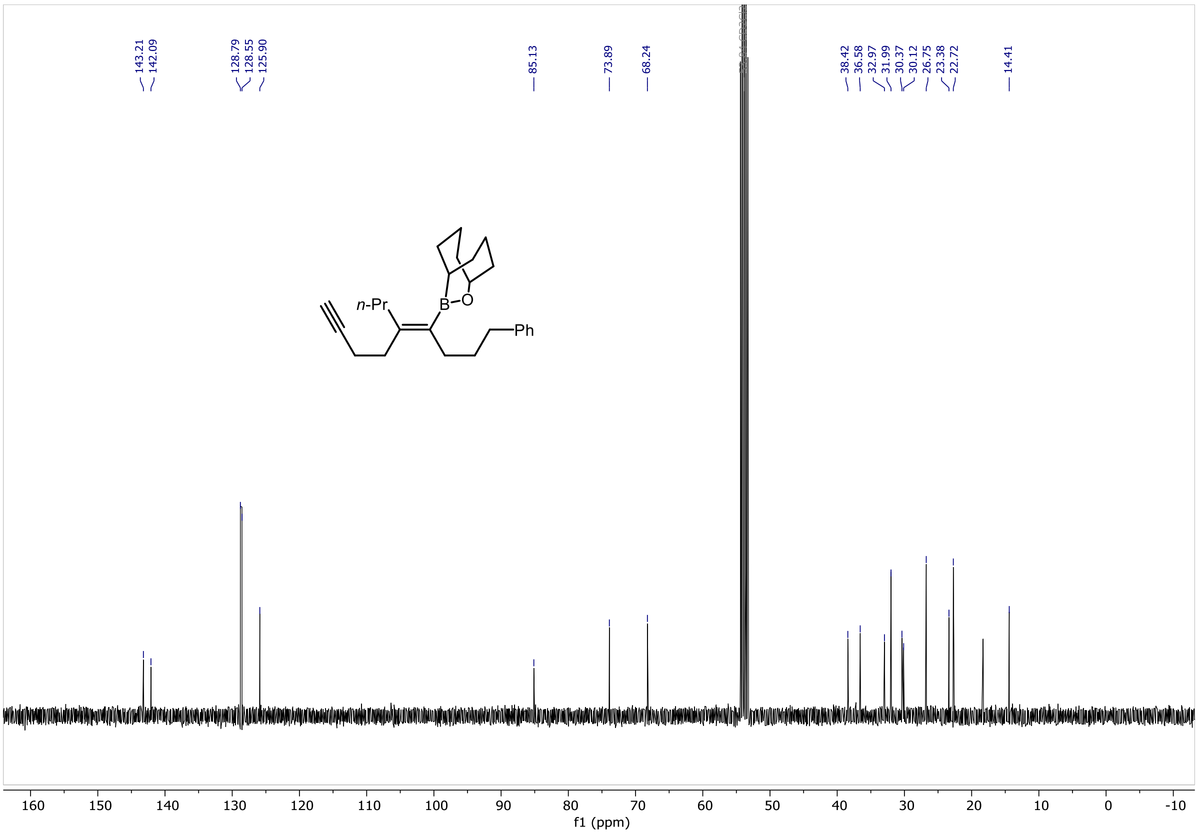

^{1}H NMR (400 MHz, CD_2Cl_2) of **5n**

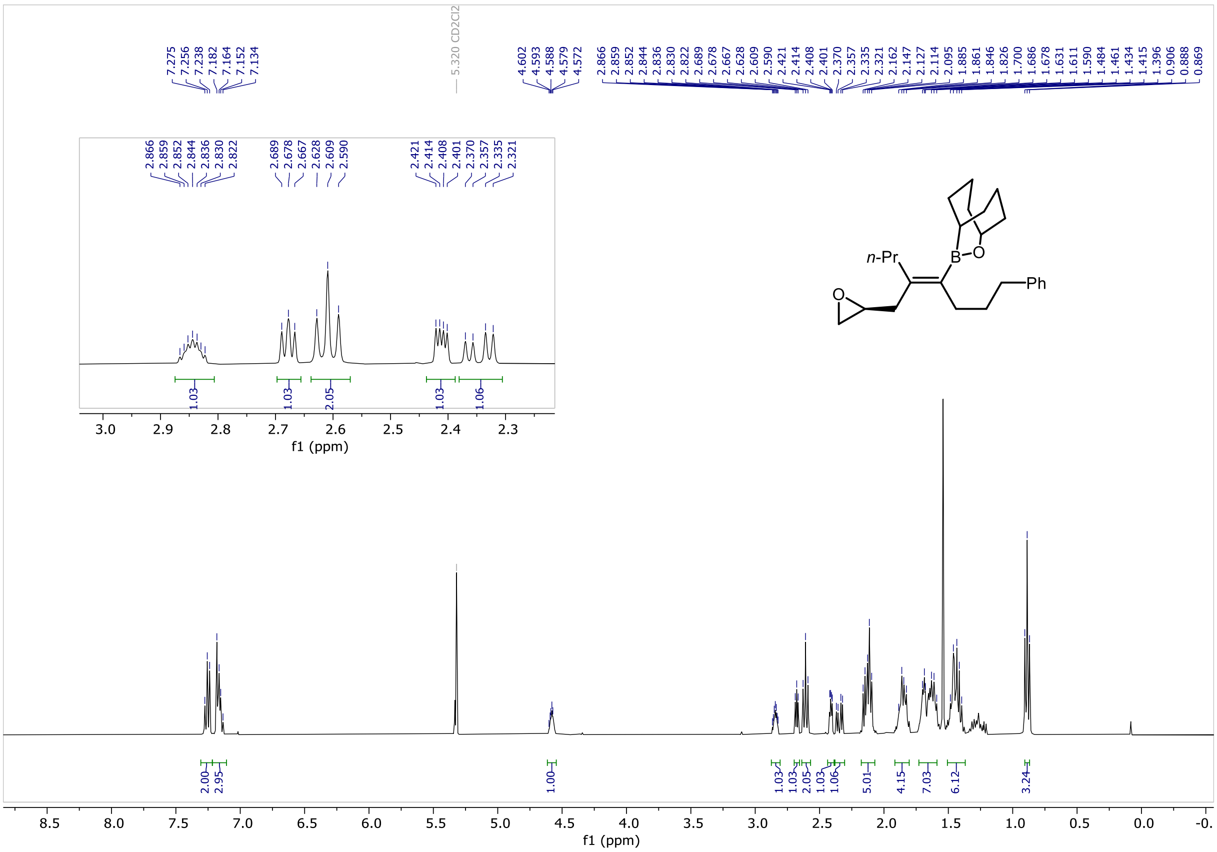

^{13}C NMR (400 MHz, CD_2Cl_2) of **5n**

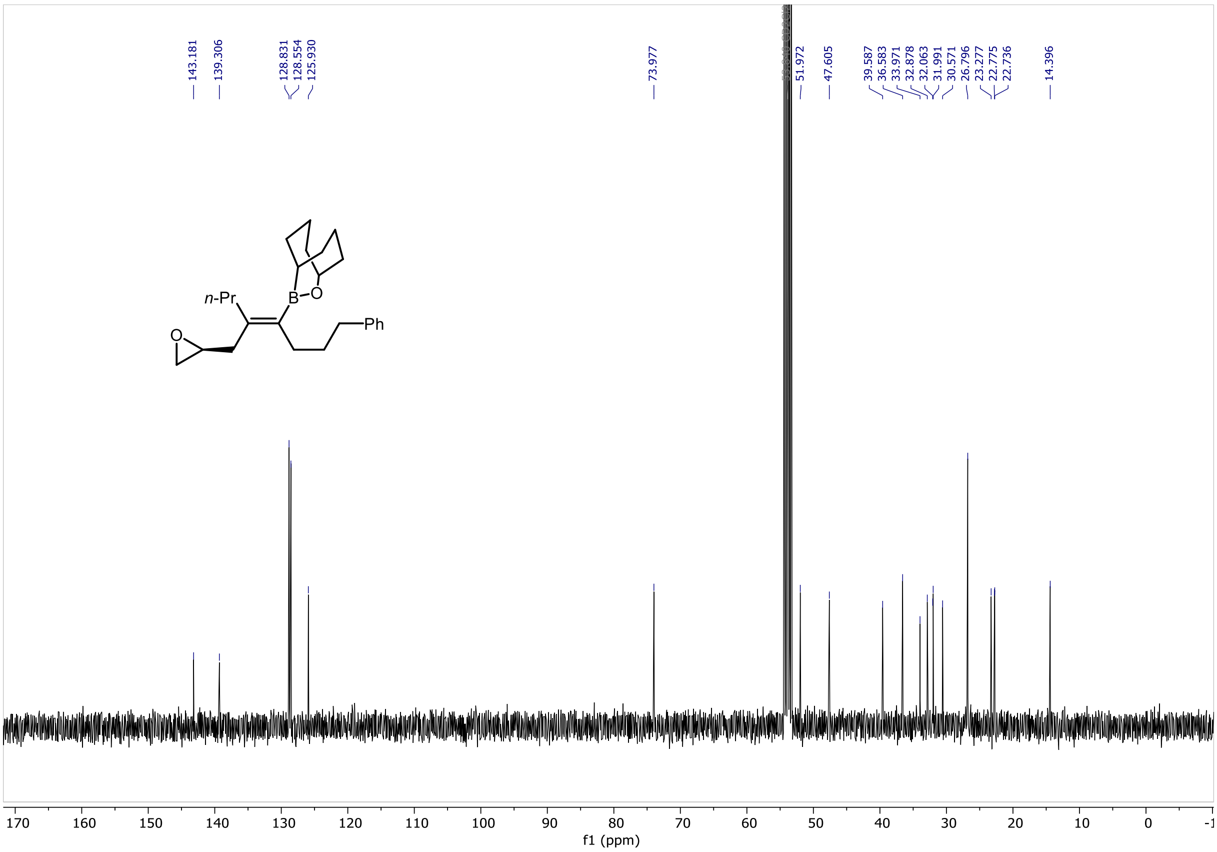

^1H NMR (400 MHz, CD_2Cl_2) of **5o**

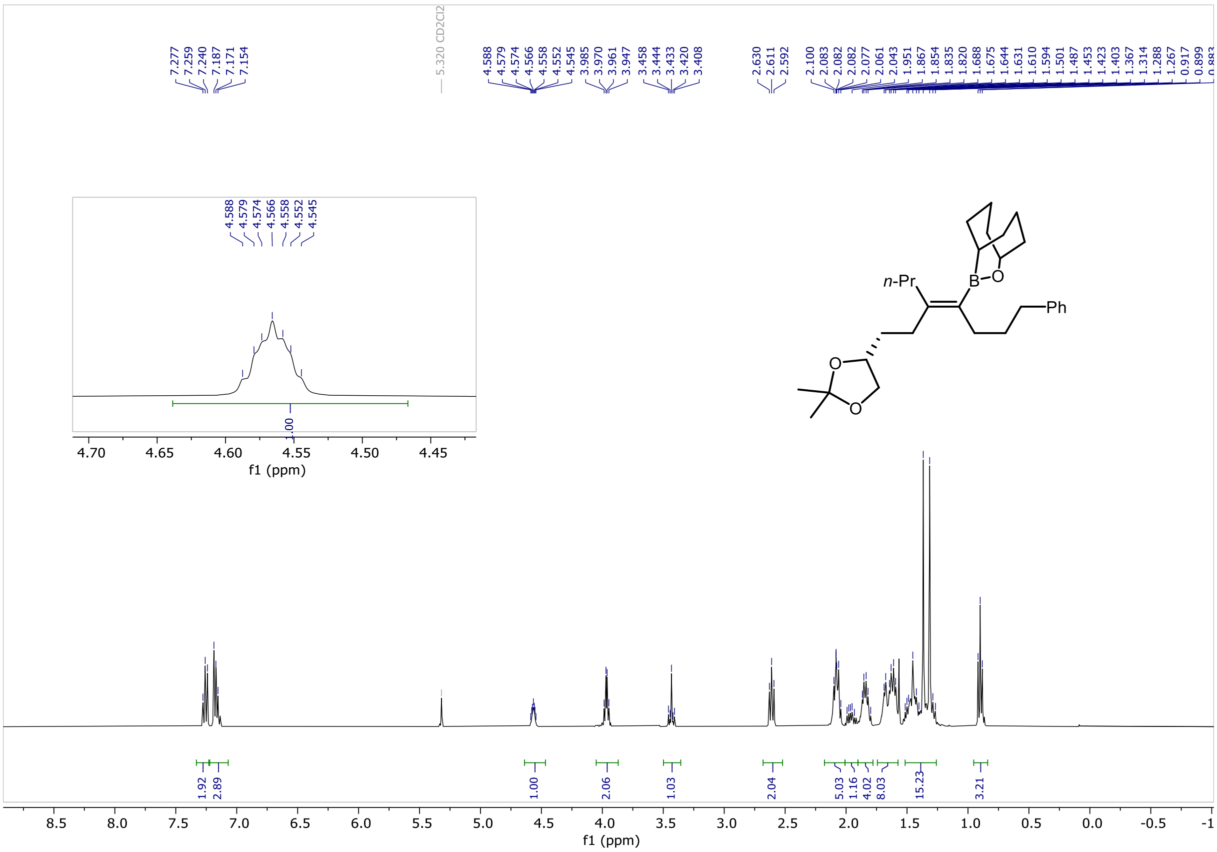

^{13}C NMR (400 MHz, CD_2Cl_2) of **5o**

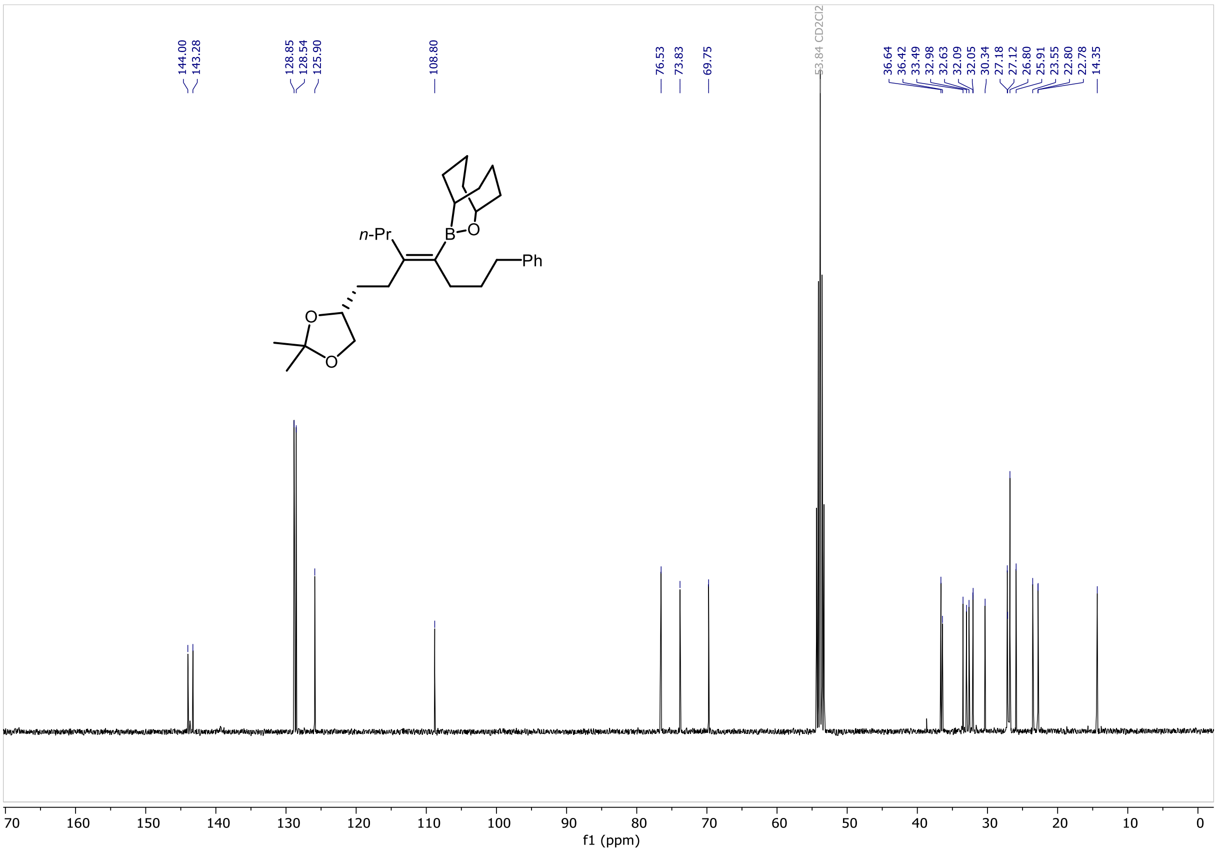

^{1}H NMR (400 MHz, CD_2Cl_2) of **5p**

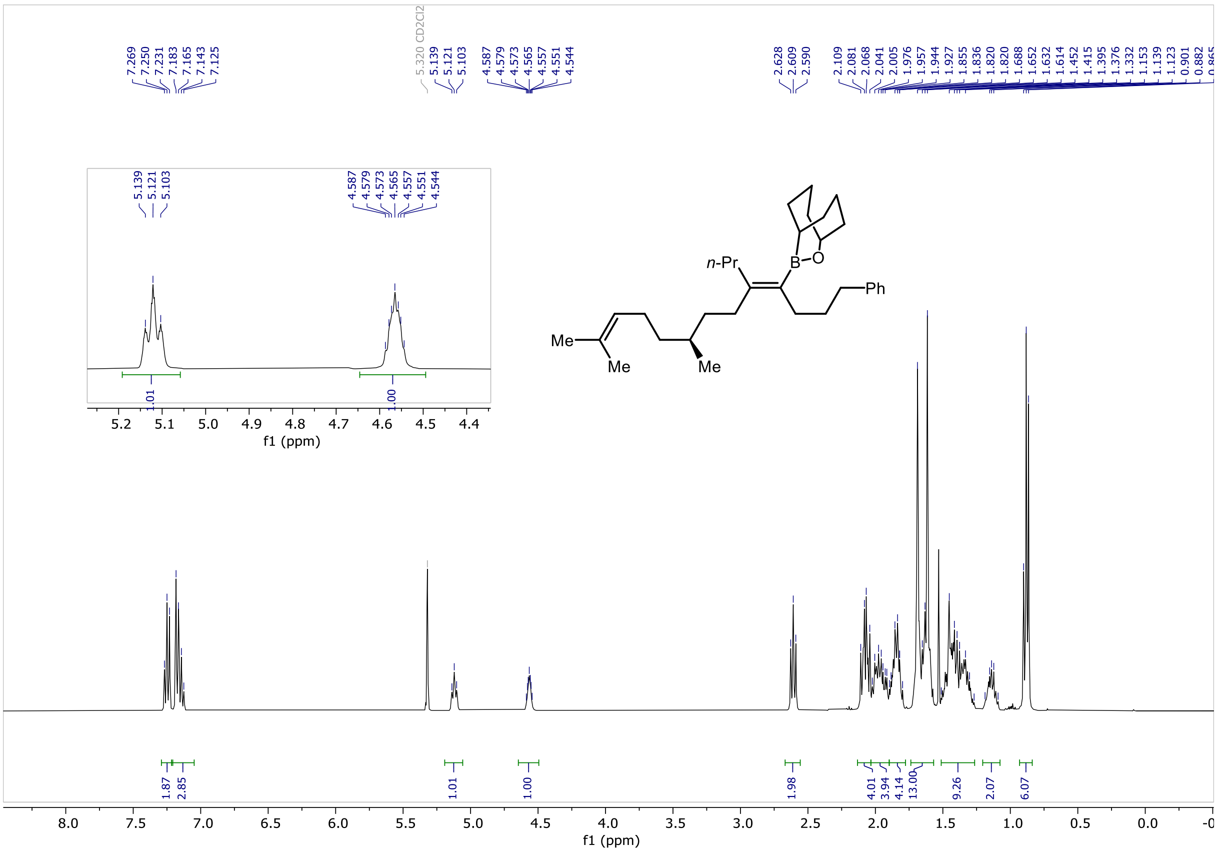

^{13}C NMR (400 MHz, CD_2Cl_2) of **5p**

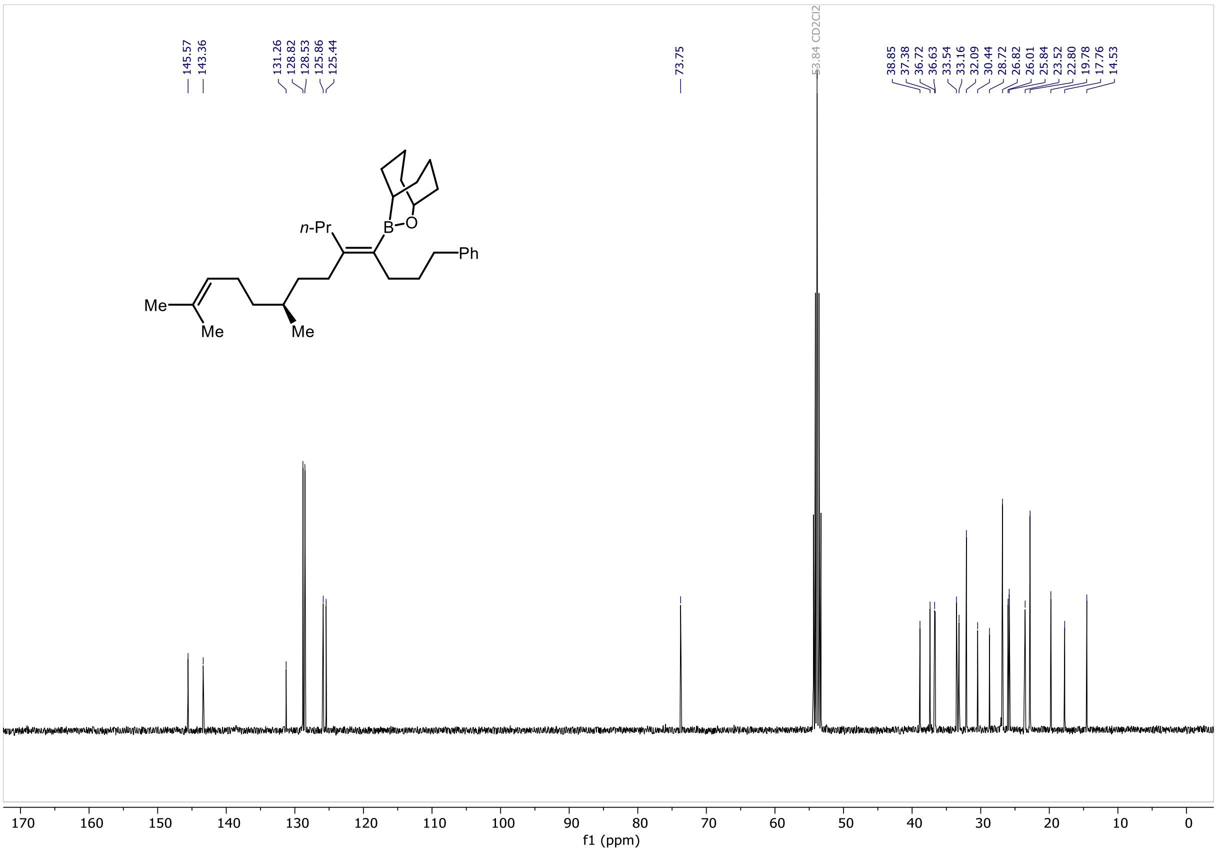

^{1}H NMR (400 MHz, CDCl3) of **5q**

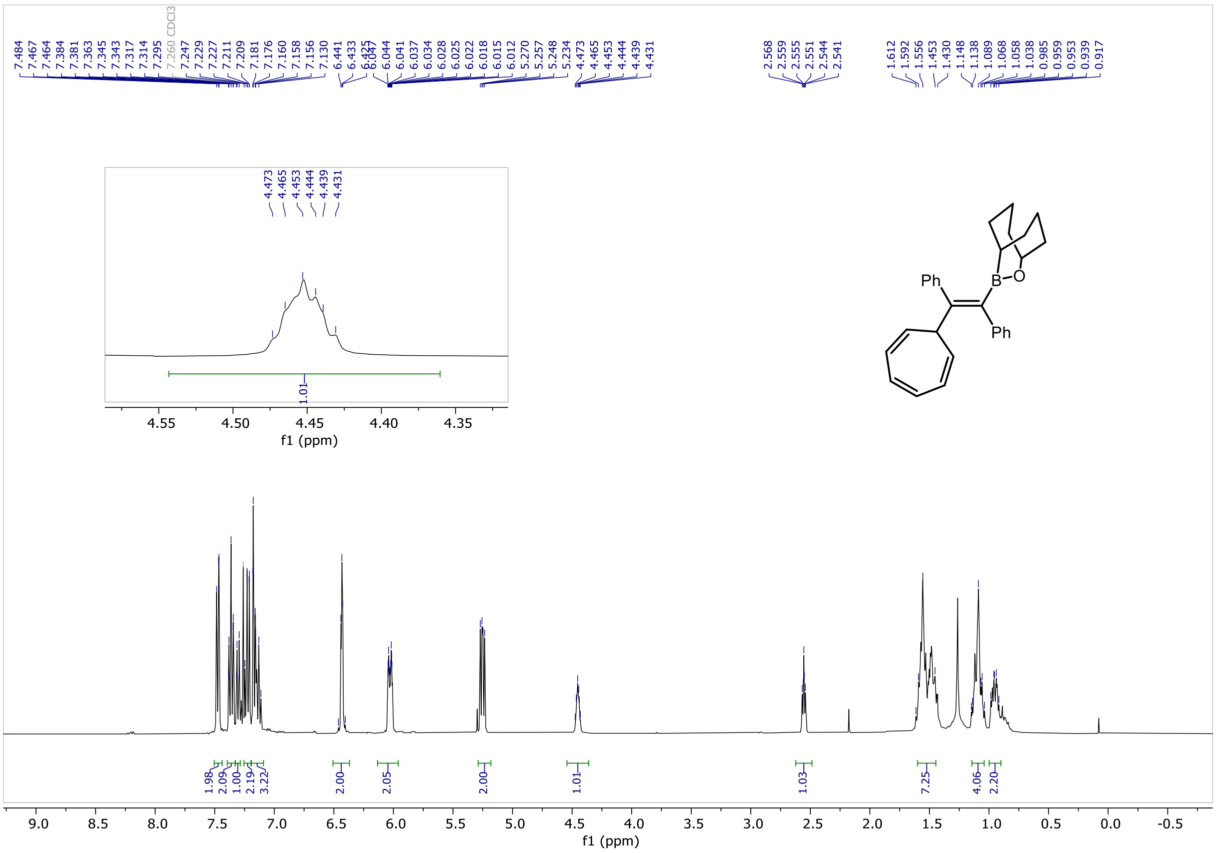

^{13}C NMR (400 MHz, CDCl3) of **5q**

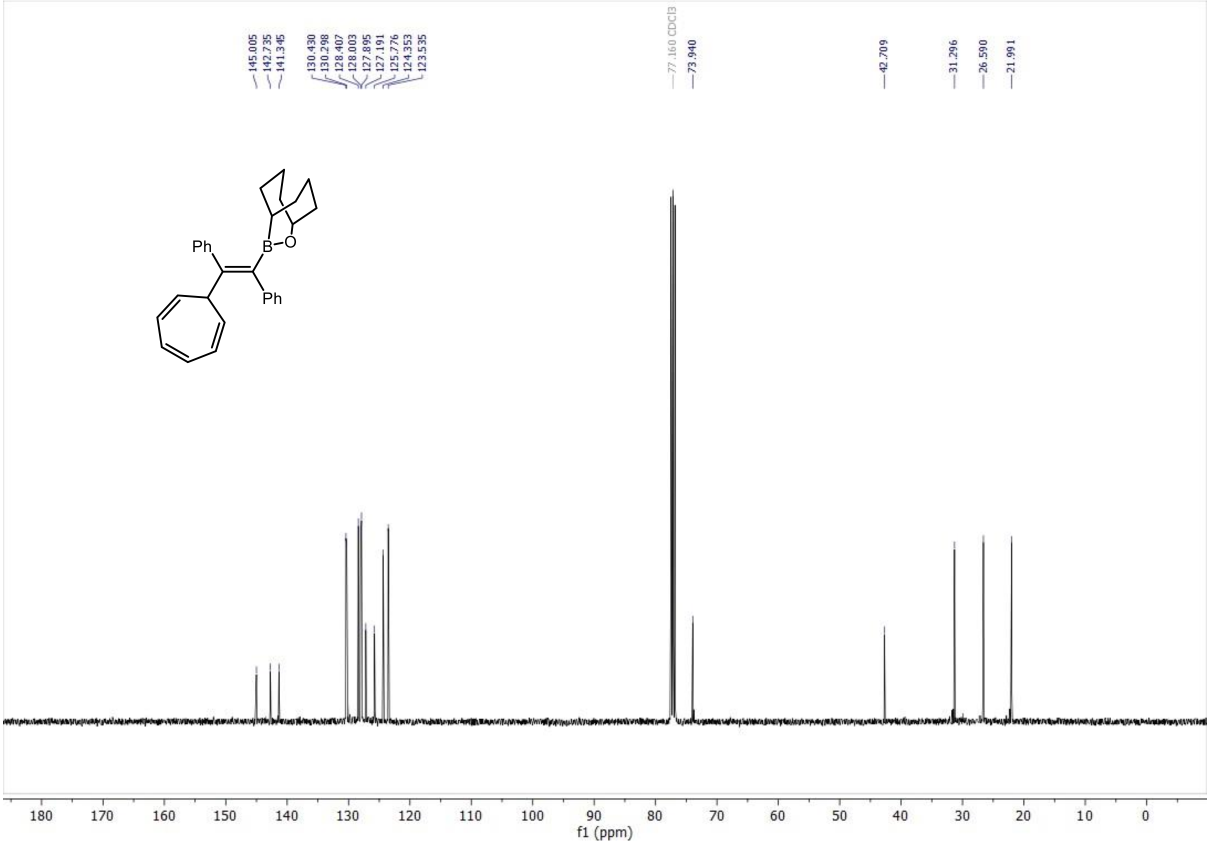

^{1}H NMR (500 MHz, CD_2Cl_2) of **5r**

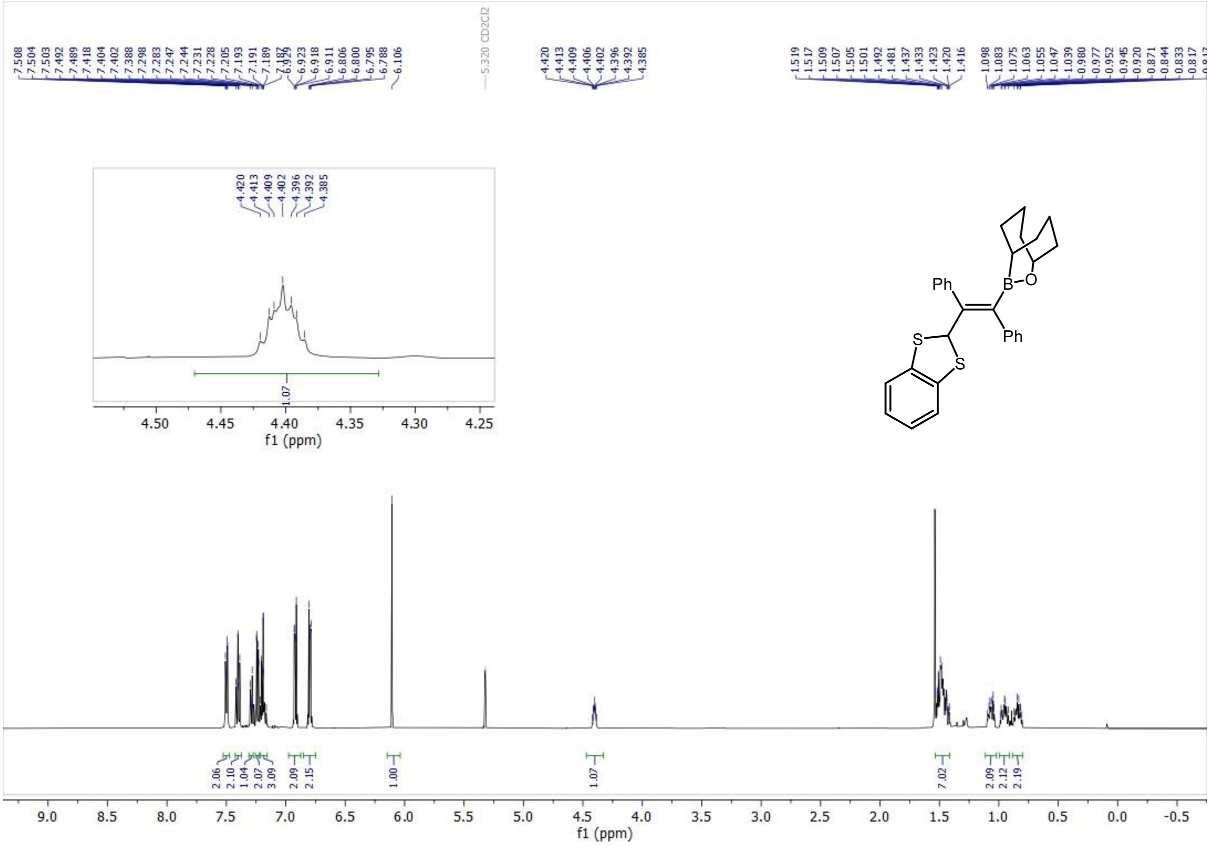

^{13}C NMR (500 MHz, CD_2Cl_2) of **5r**

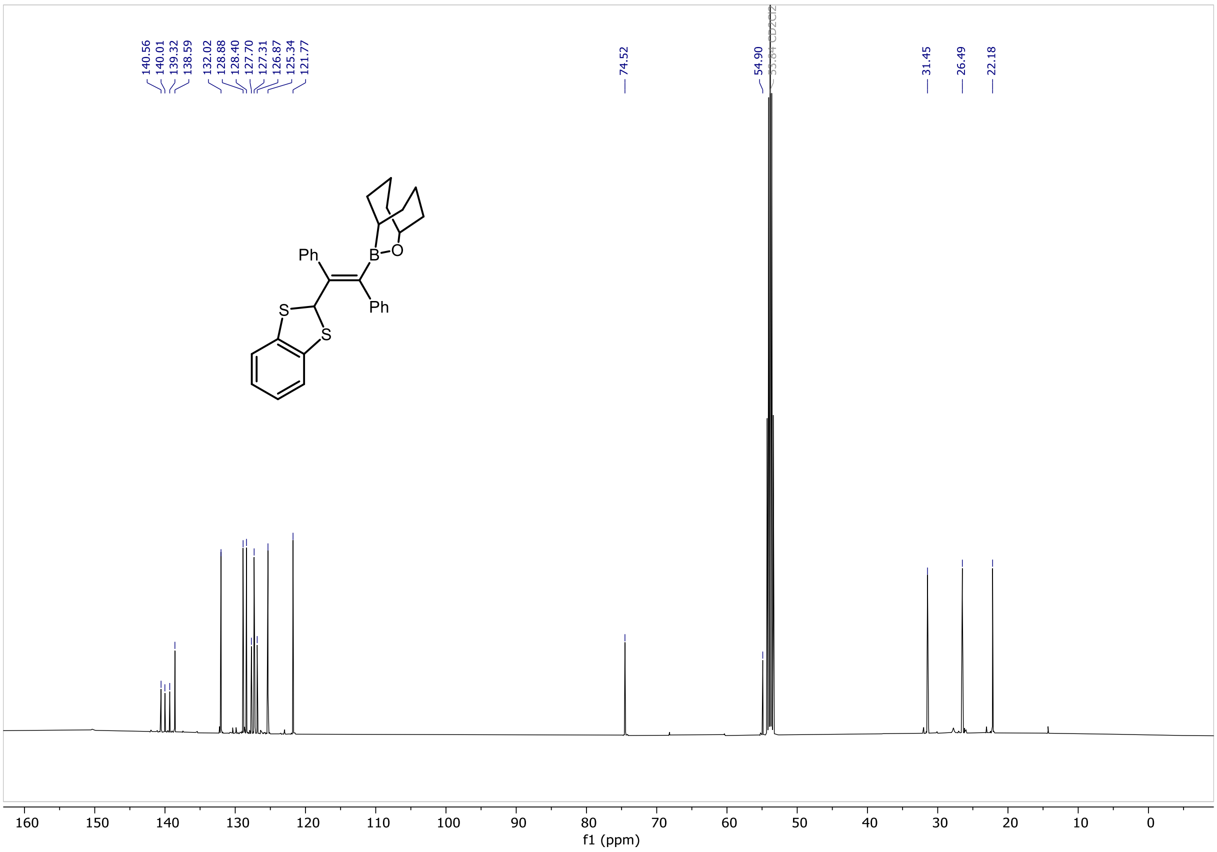

^{1}H NMR (400 MHz, $CDCl_3$) of **5s**

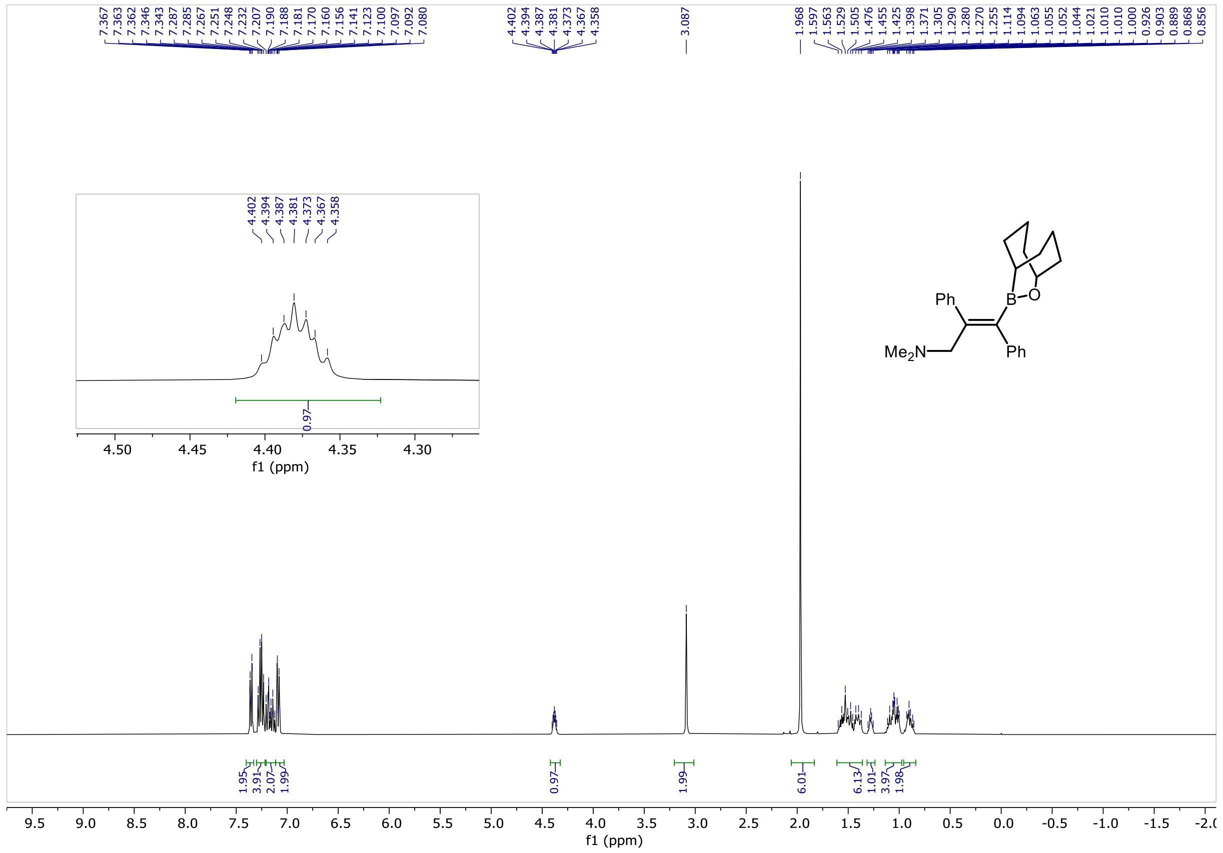

^{13}C NMR (400 MHz, $CDCl_3$) of **5s**

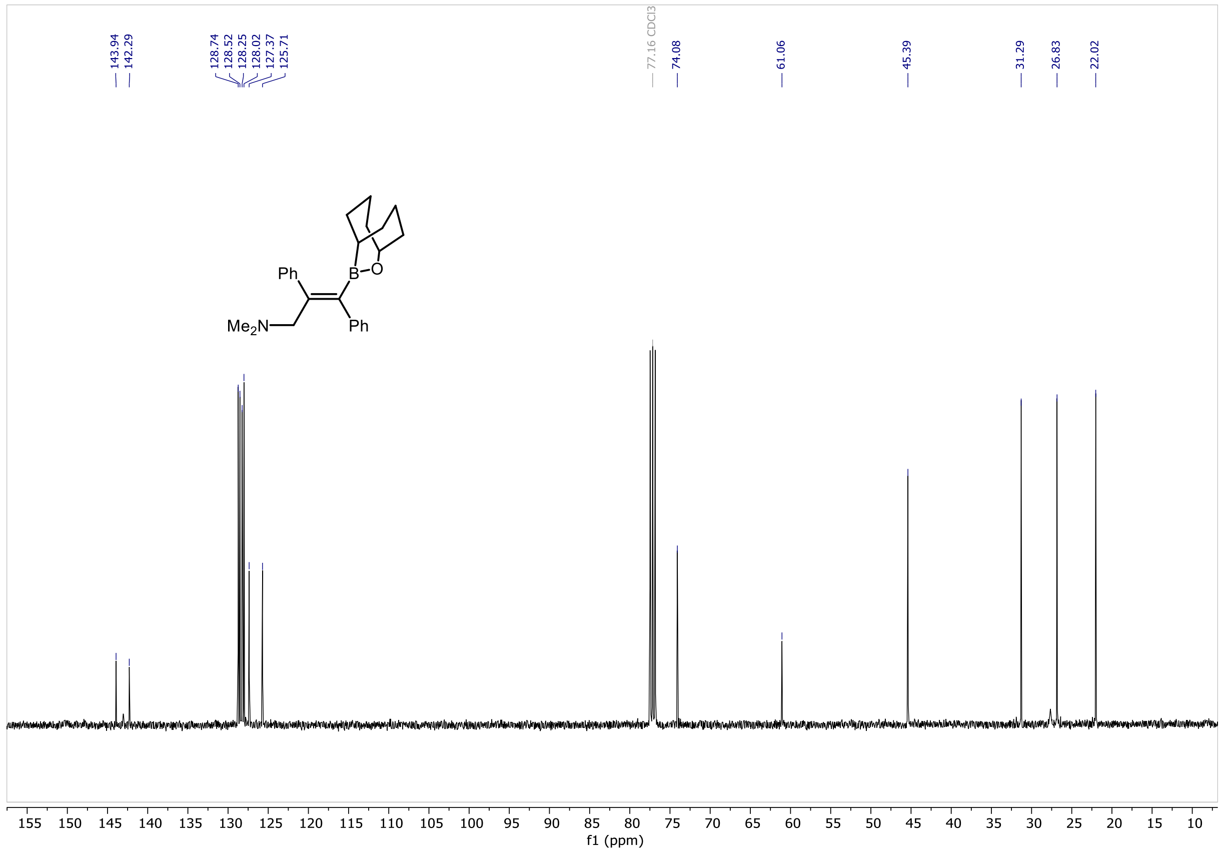

^{1}H NMR (400 MHz, $CDCl_3$) of **5t**

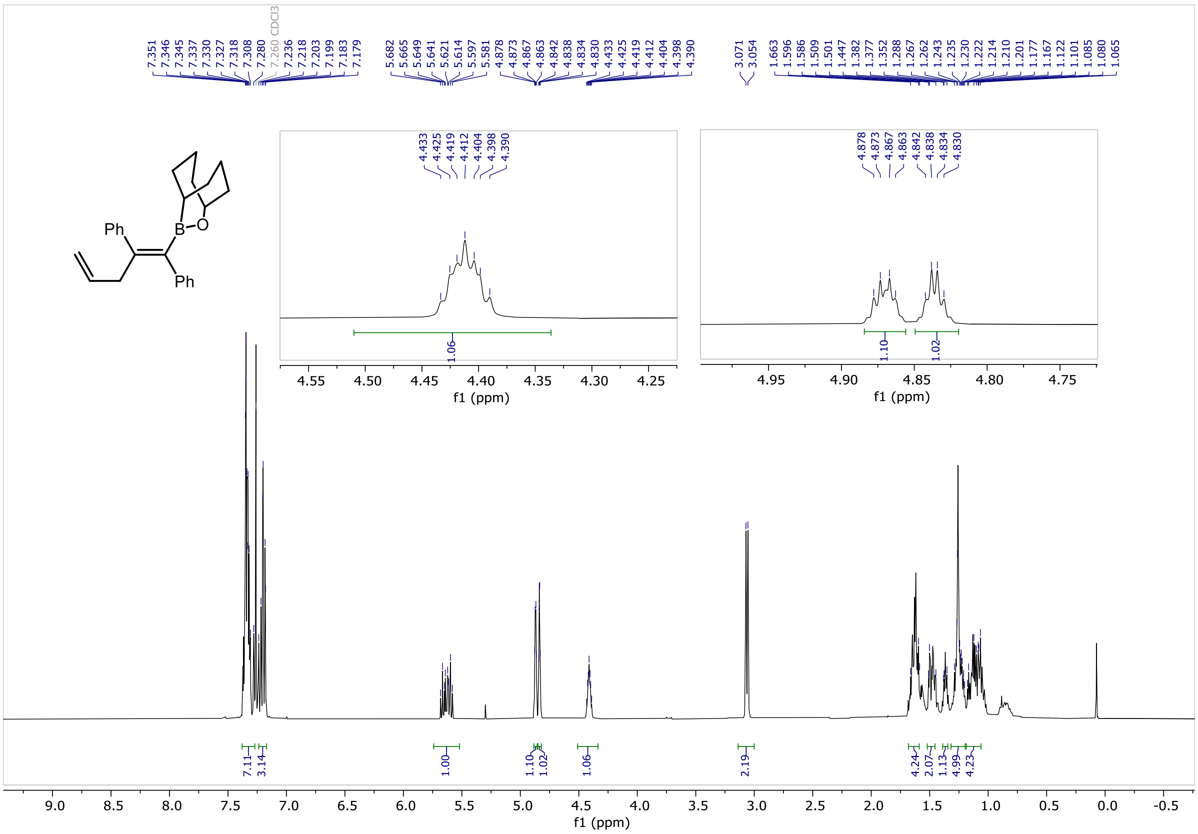

^{13}C NMR (400 MHz, $CDCl_3$) of **5t**

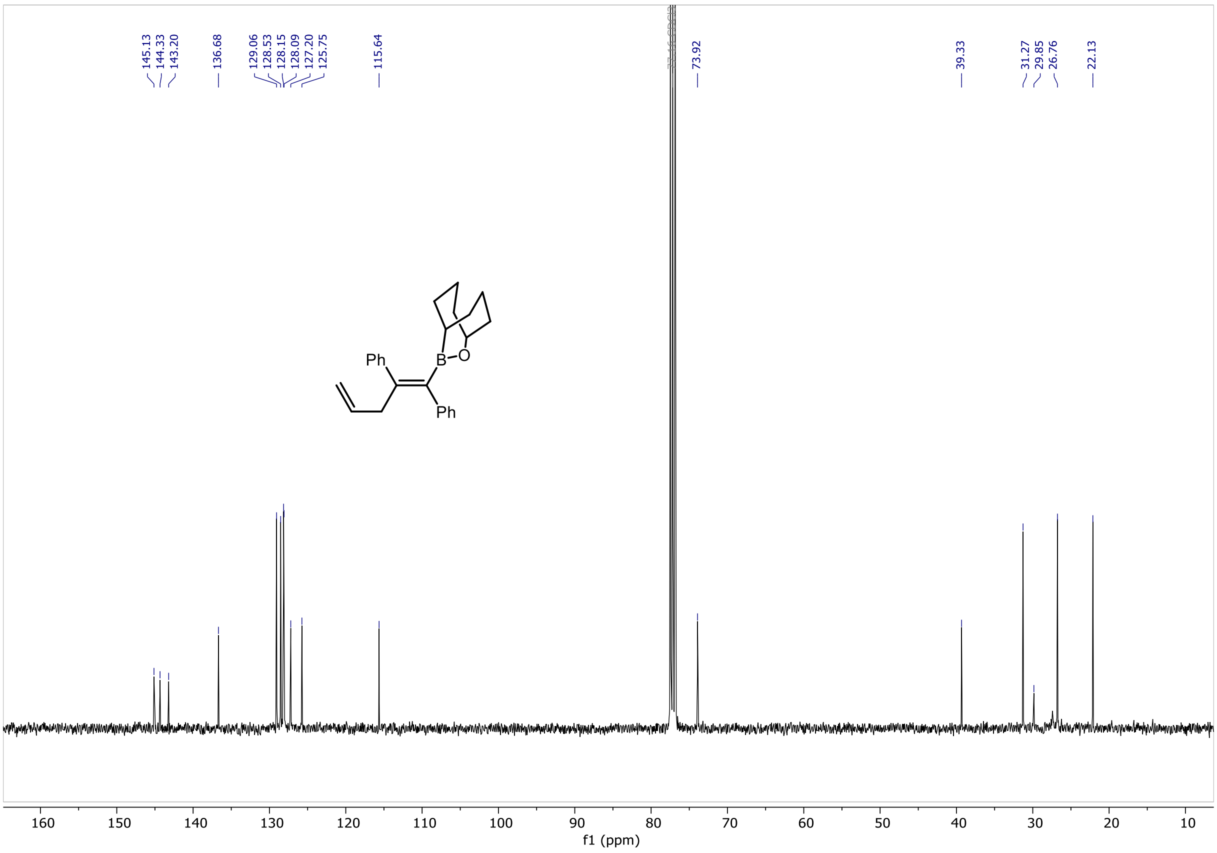

^{1}H NMR (500 MHz, CDCl$_3$) of **5u**

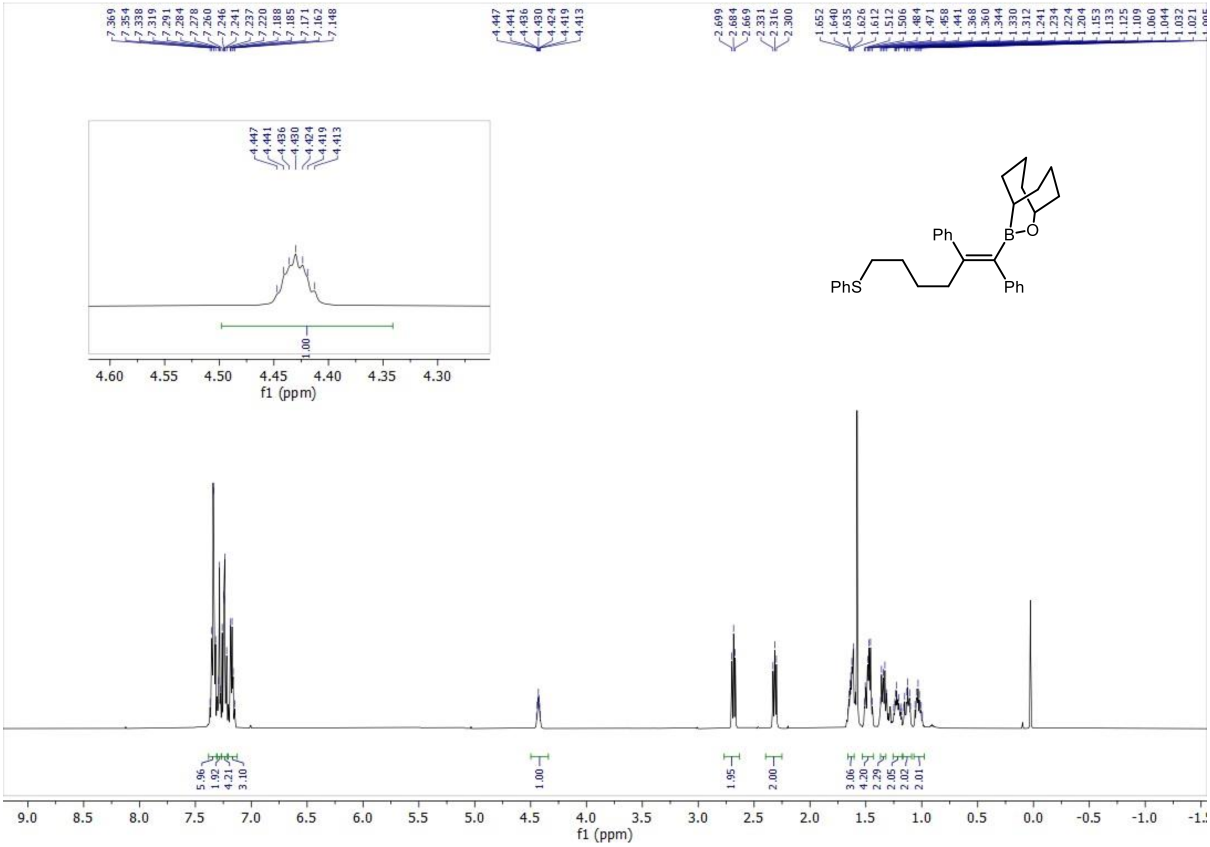

^{13}C NMR (500 MHz, CDCl$_3$) of **5u**

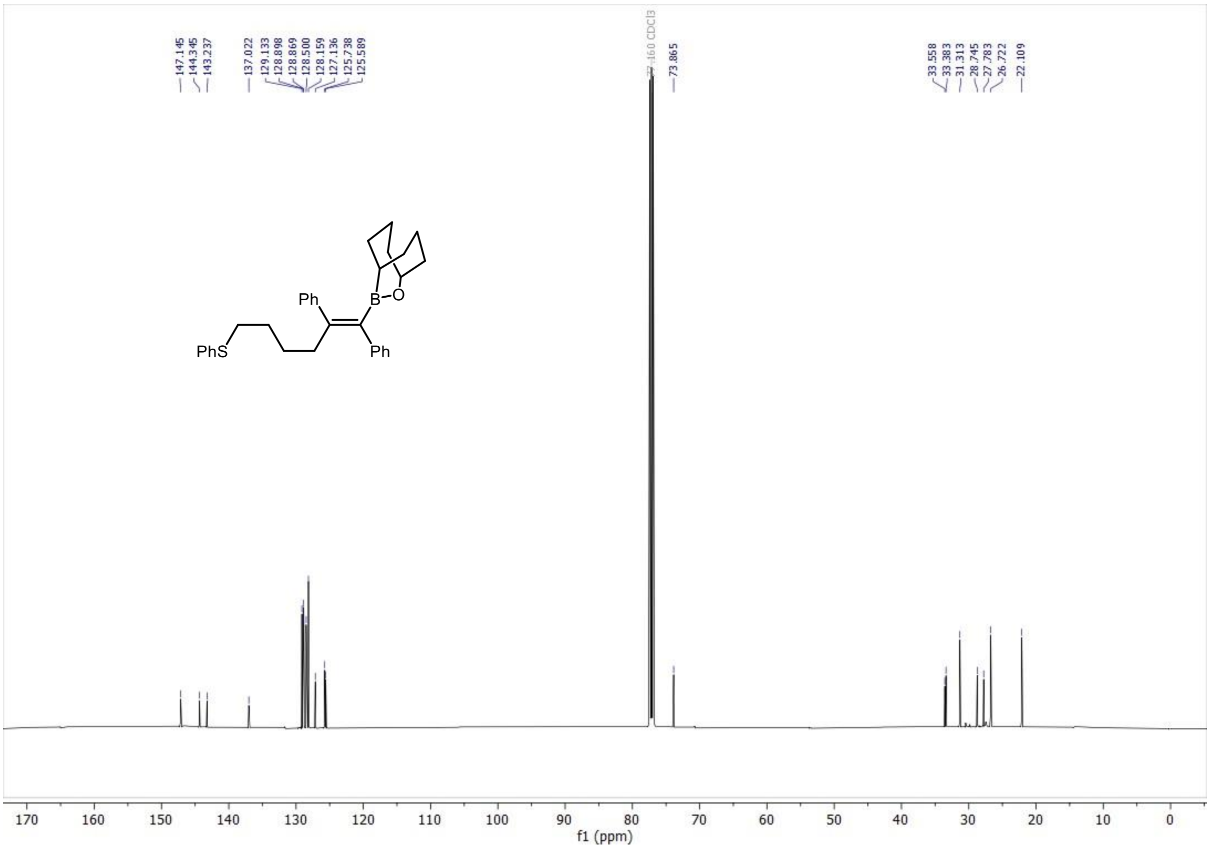

^{1}H NMR (400 MHz, CD_2Cl_2) of **5v**

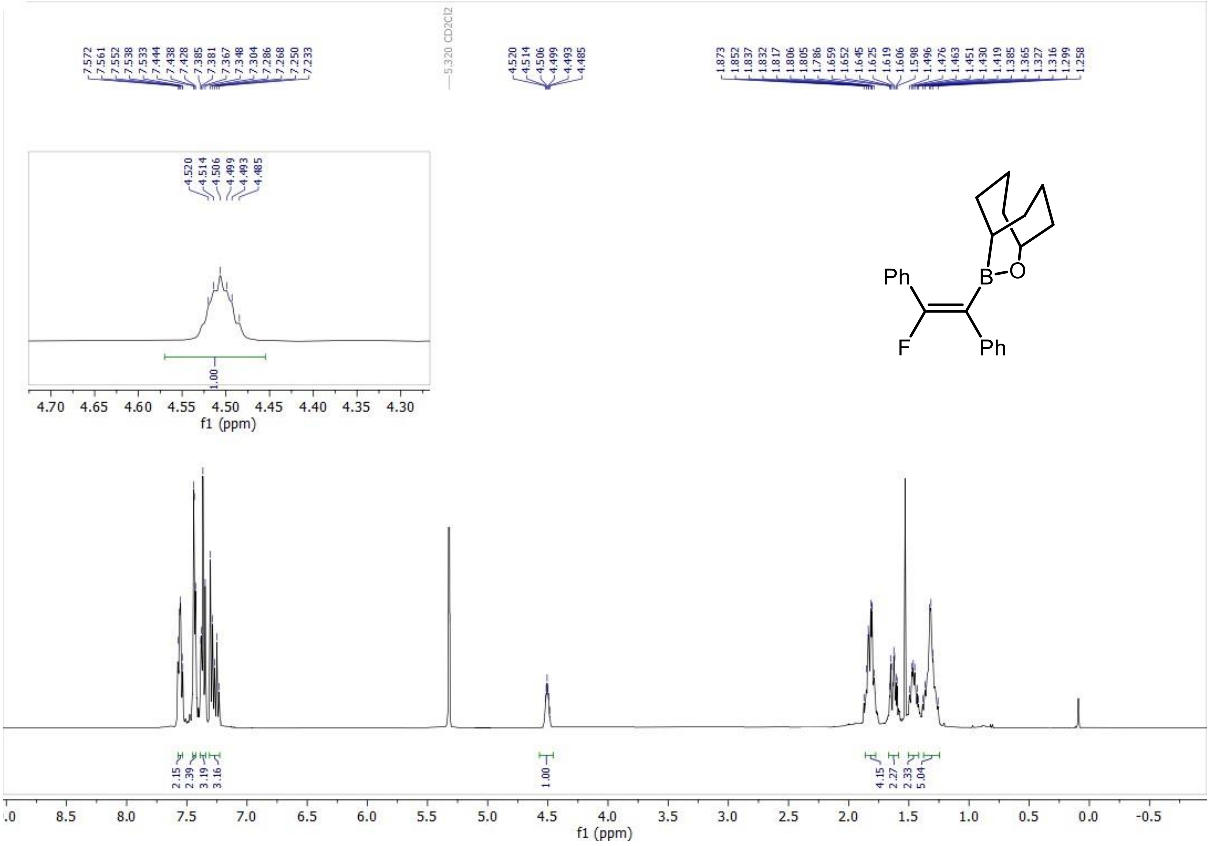

^{13}C NMR (101 MHz, CD_2Cl_2) of **5v**

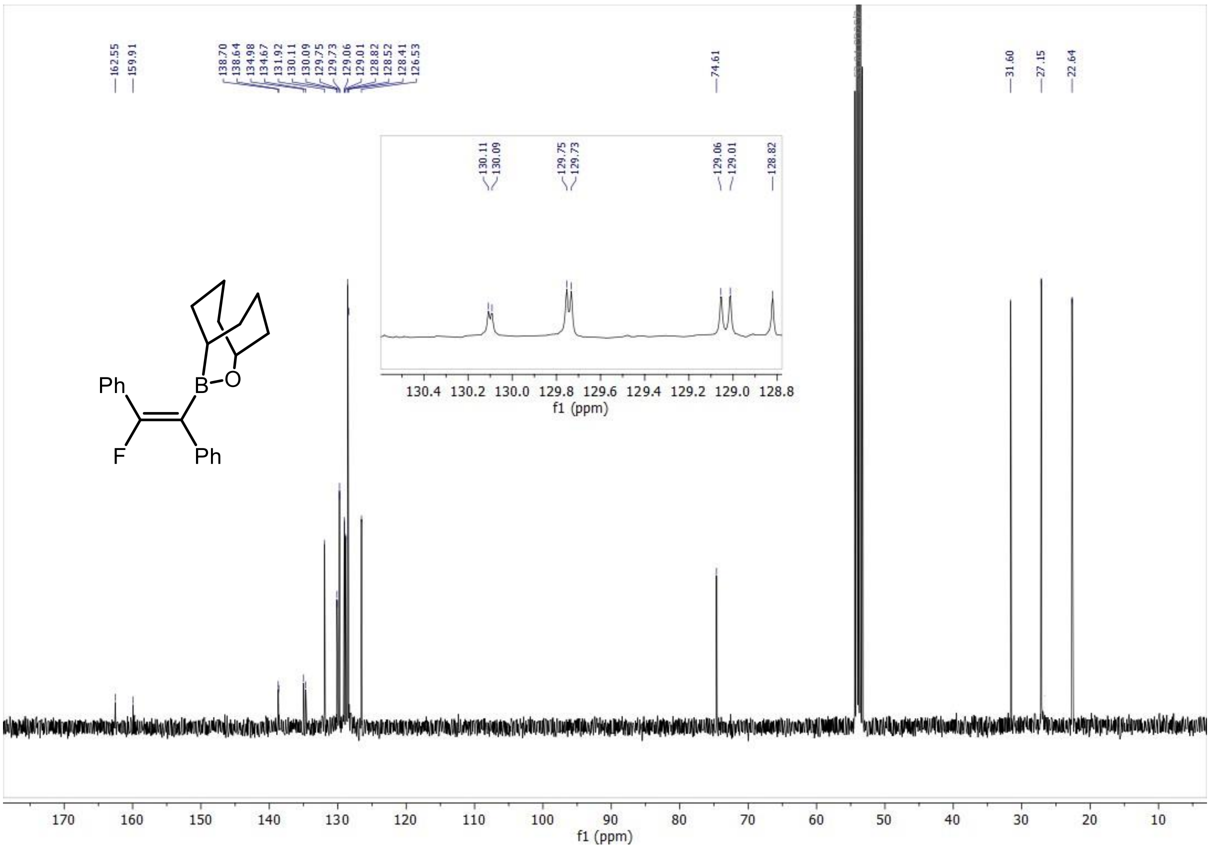

^{19}F NMR (101 MHz, CD_2Cl_2) of **5v**

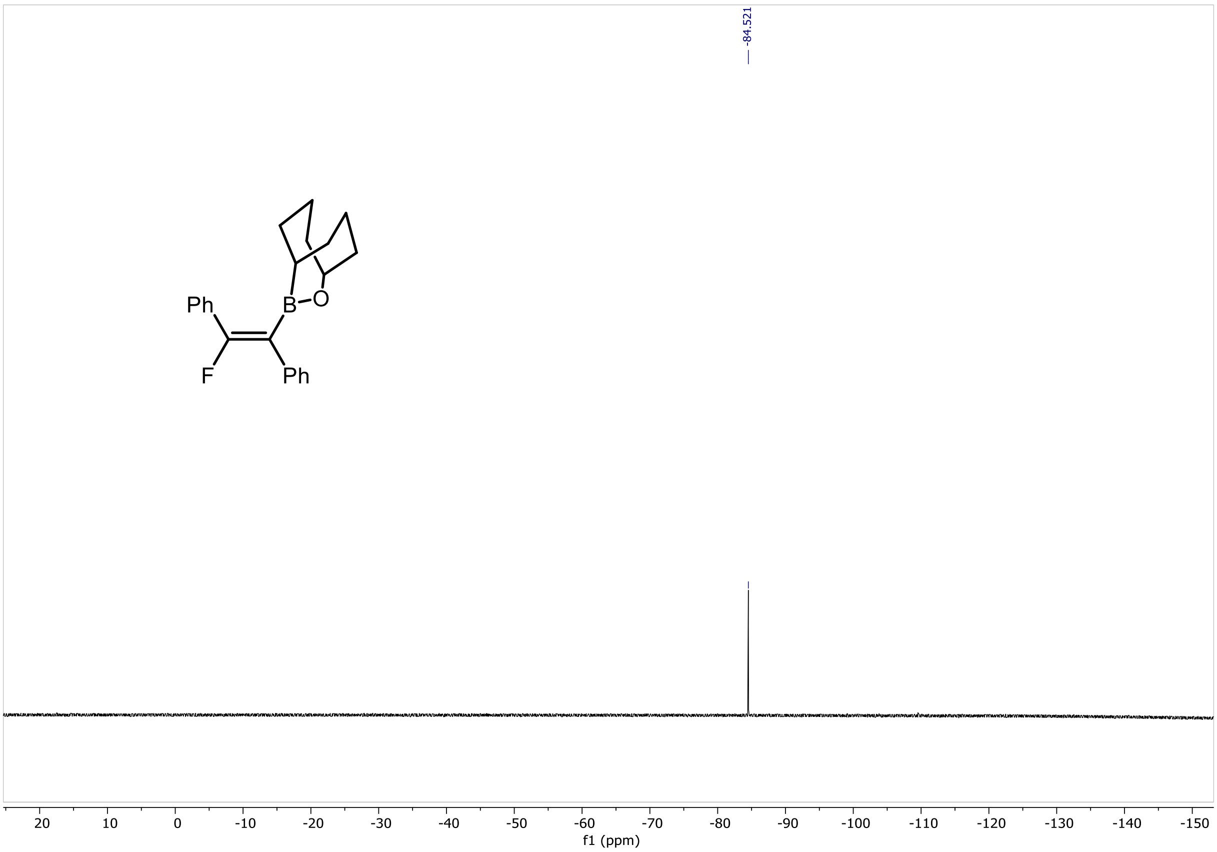

^{1}H NMR (400 MHz, $CDCl_3$) of **5w**

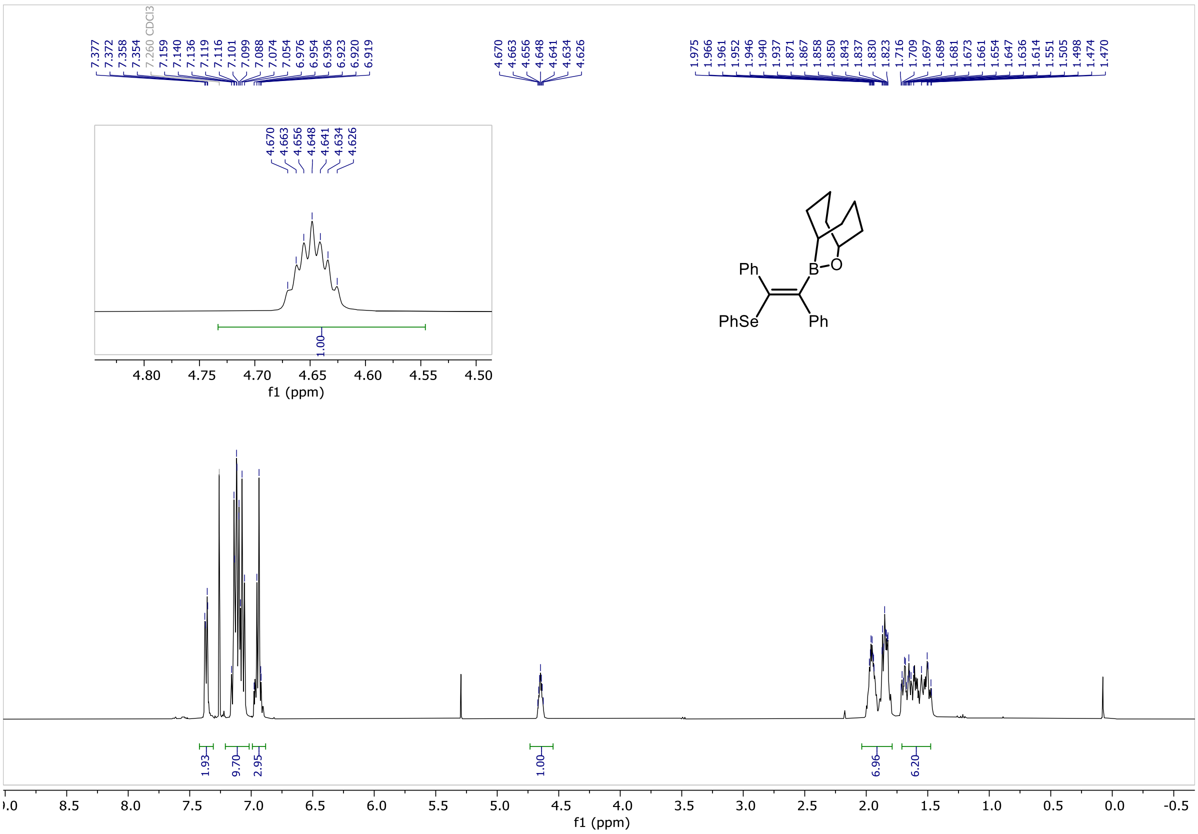

^{13}C NMR (400 MHz, $CDCl_3$) of **5w**

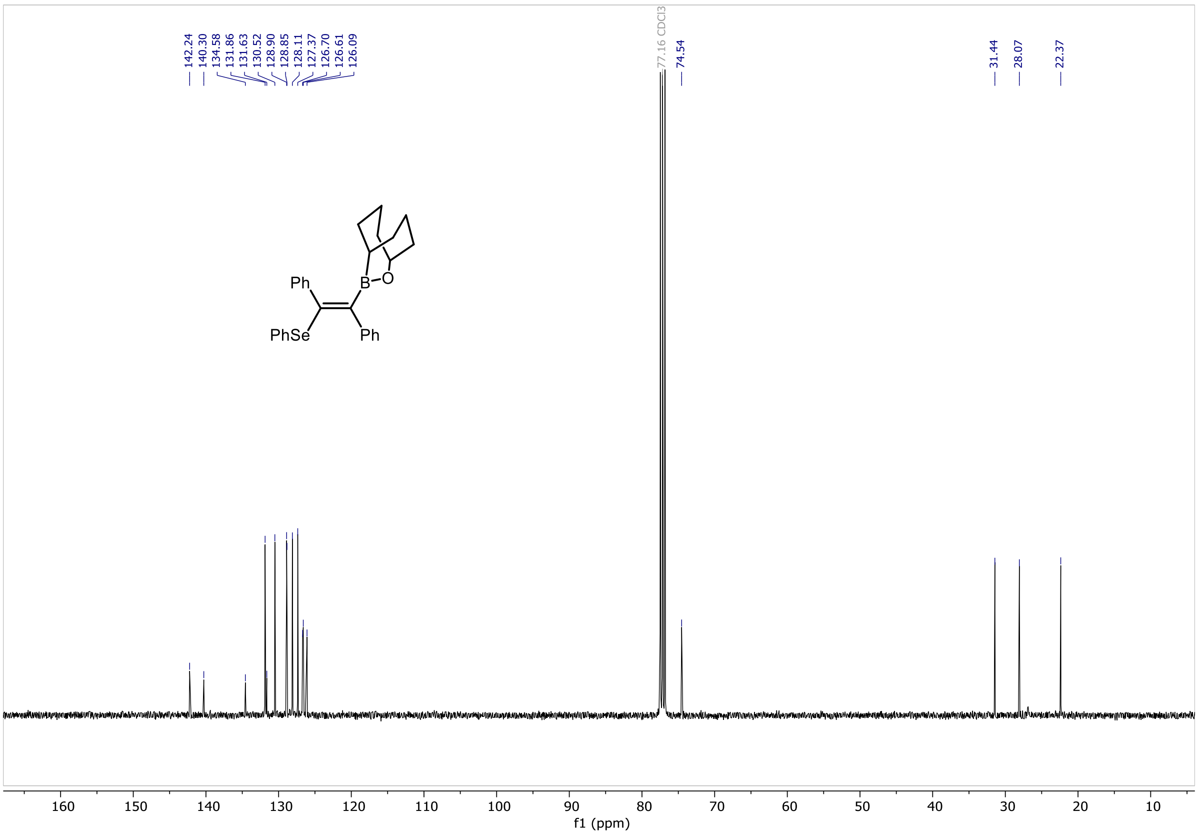

^{1}H NMR (500 MHz, $CDCl_3$) of **5x**

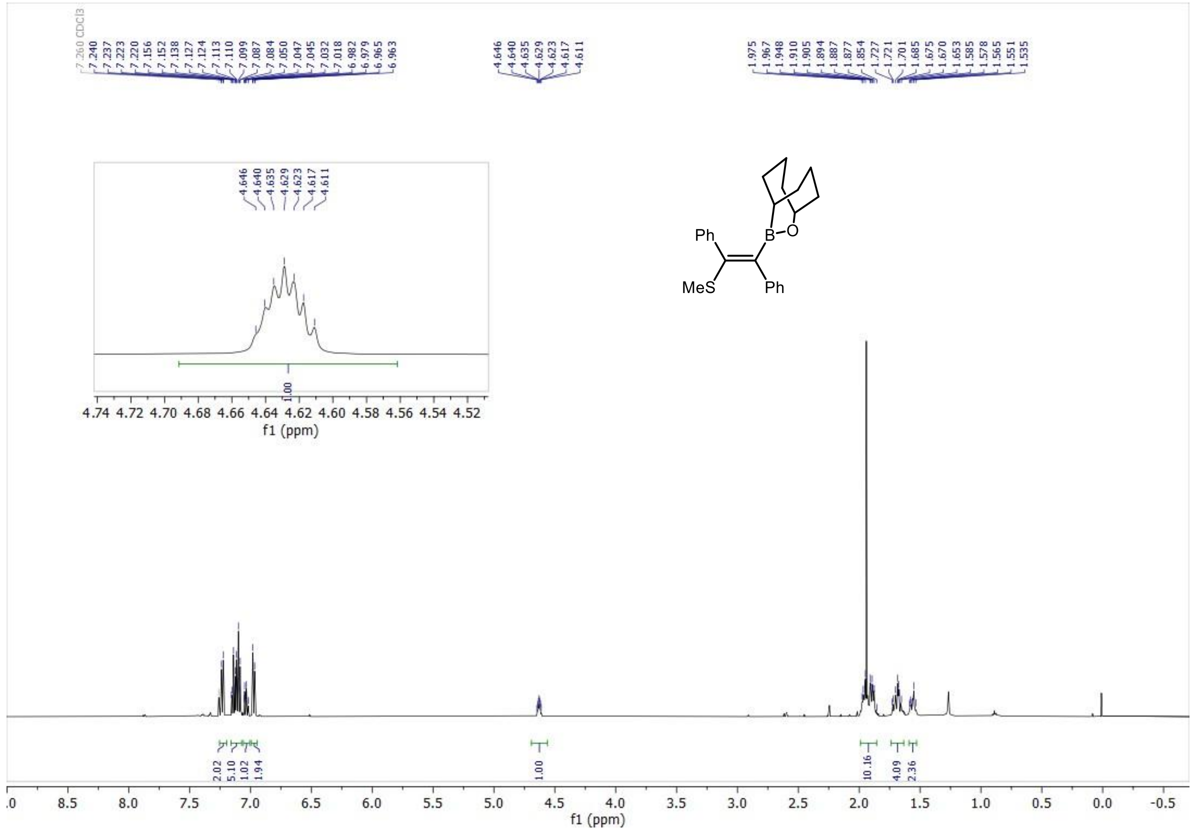

^{13}C NMR (500 MHz, $CDCl_3$) of **5x**

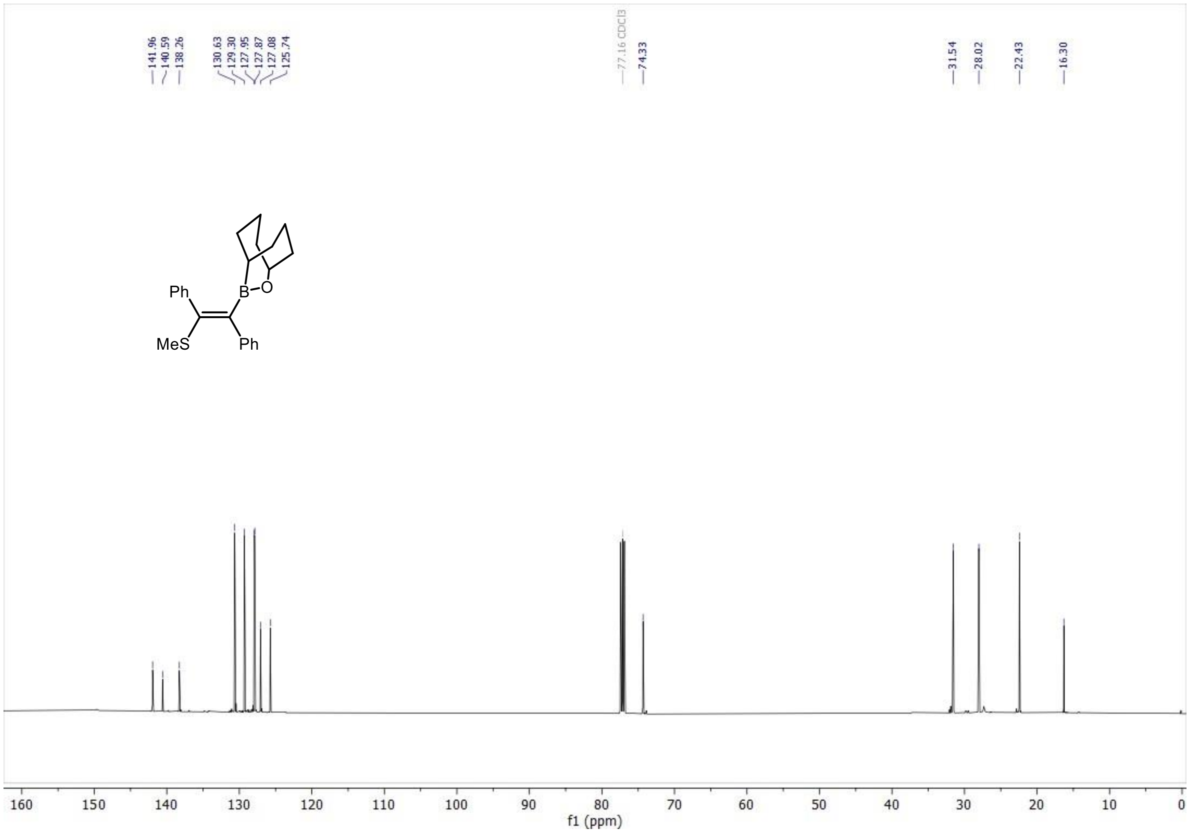

^{1}H NMR (400 MHz, CD_2Cl_2) of **6a**

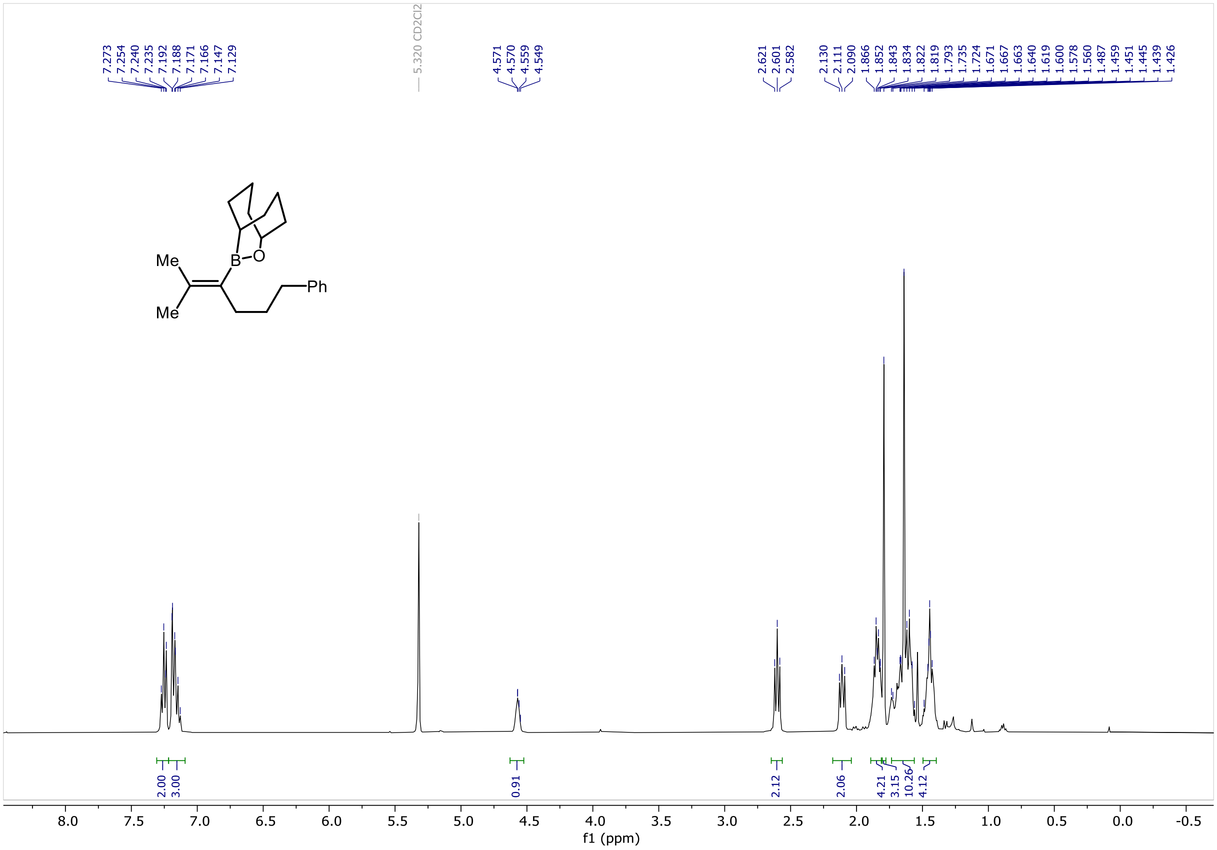

^{13}C NMR (400 MHz, CD_2Cl_2) of **6a**

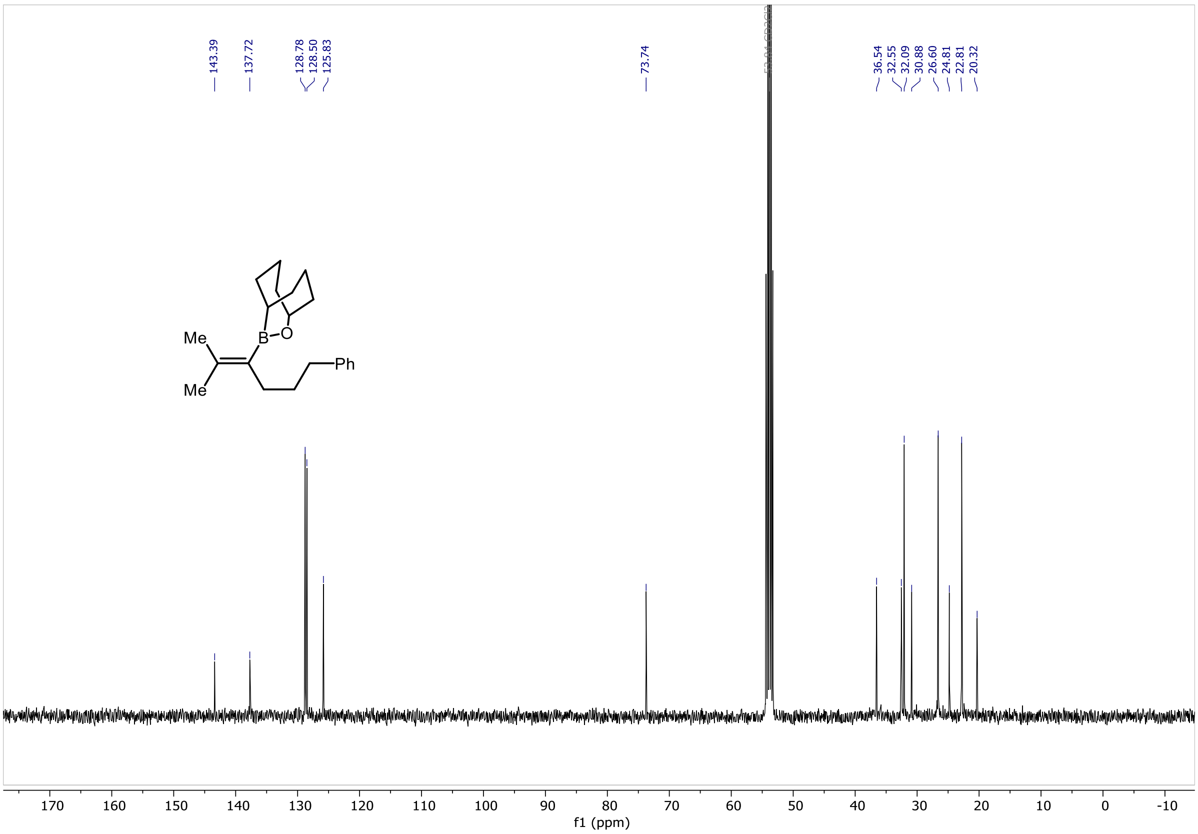

^{1}H NMR (400 MHz, CD_2Cl_2) of **6b**

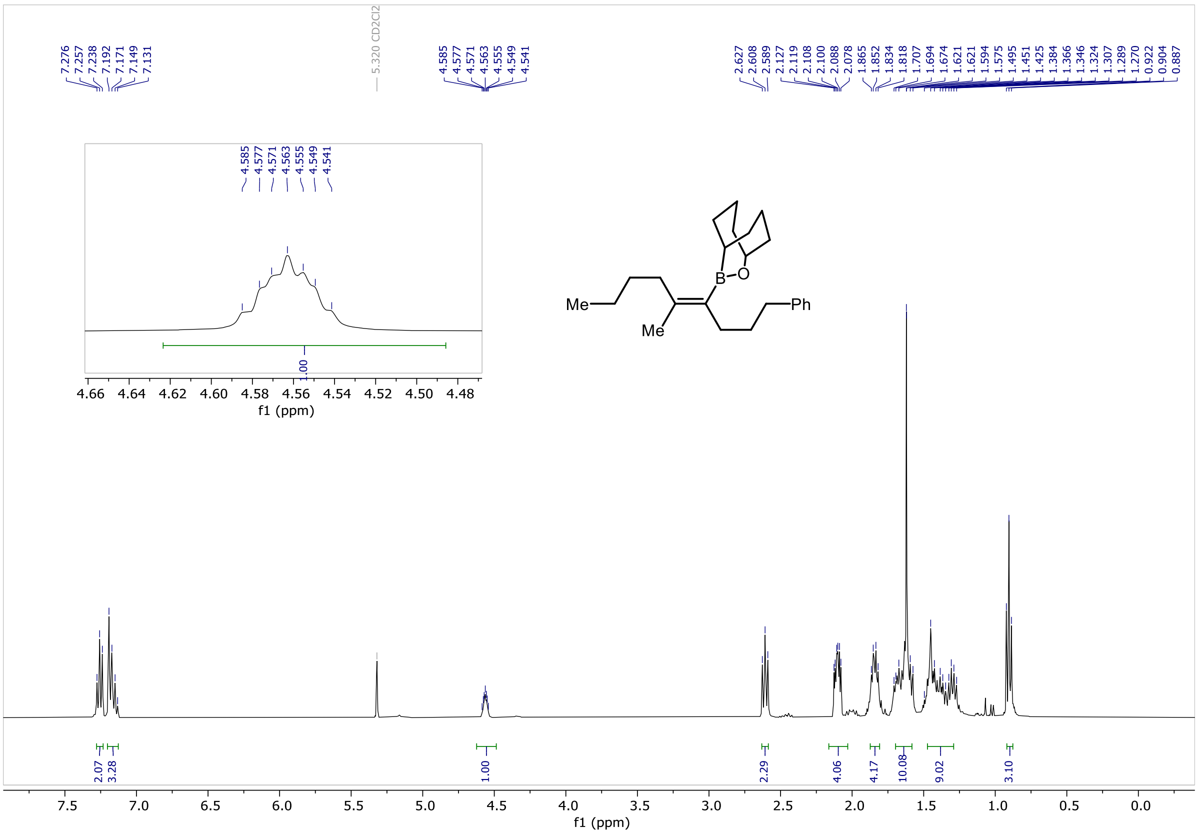

^{13}C NMR (400 MHz, CD_2Cl_2) of **6b**

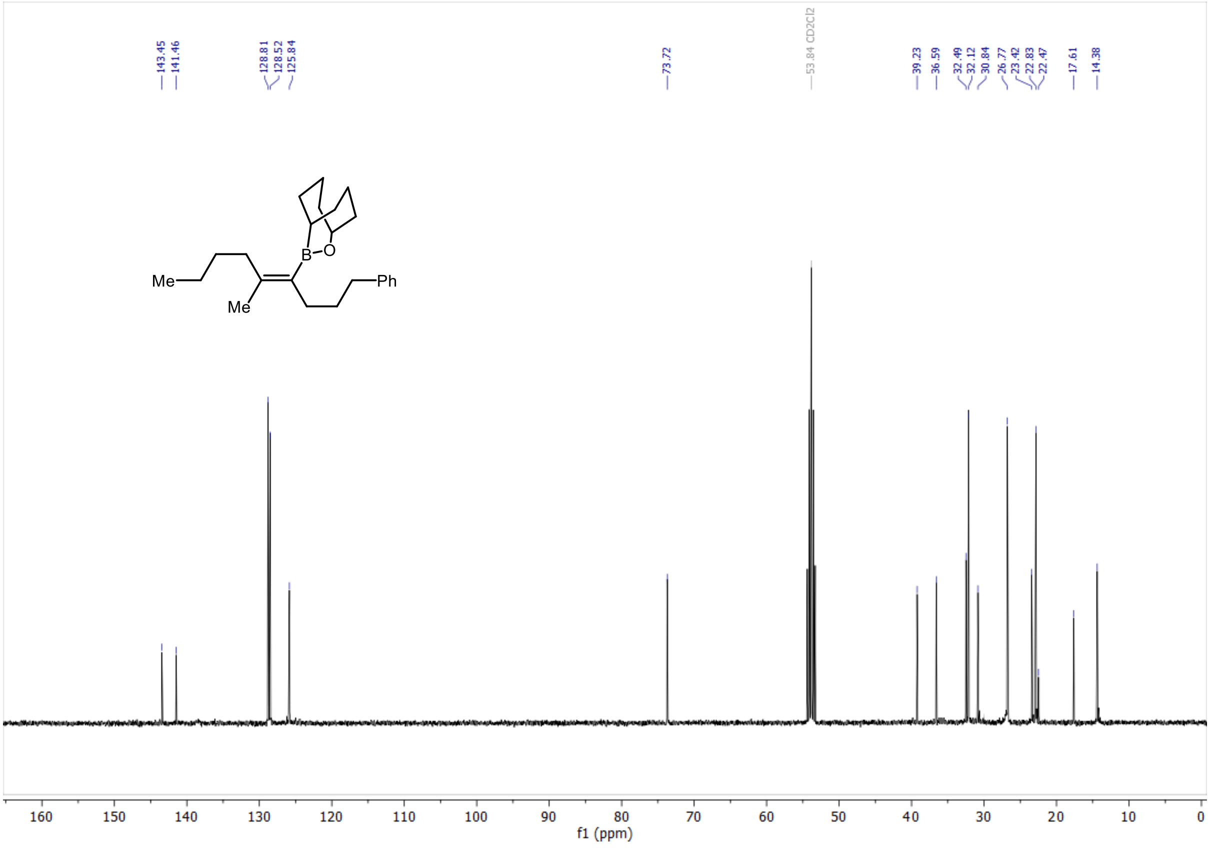

^{1}H NMR (400 MHz, CD_2Cl_2) of **6c**

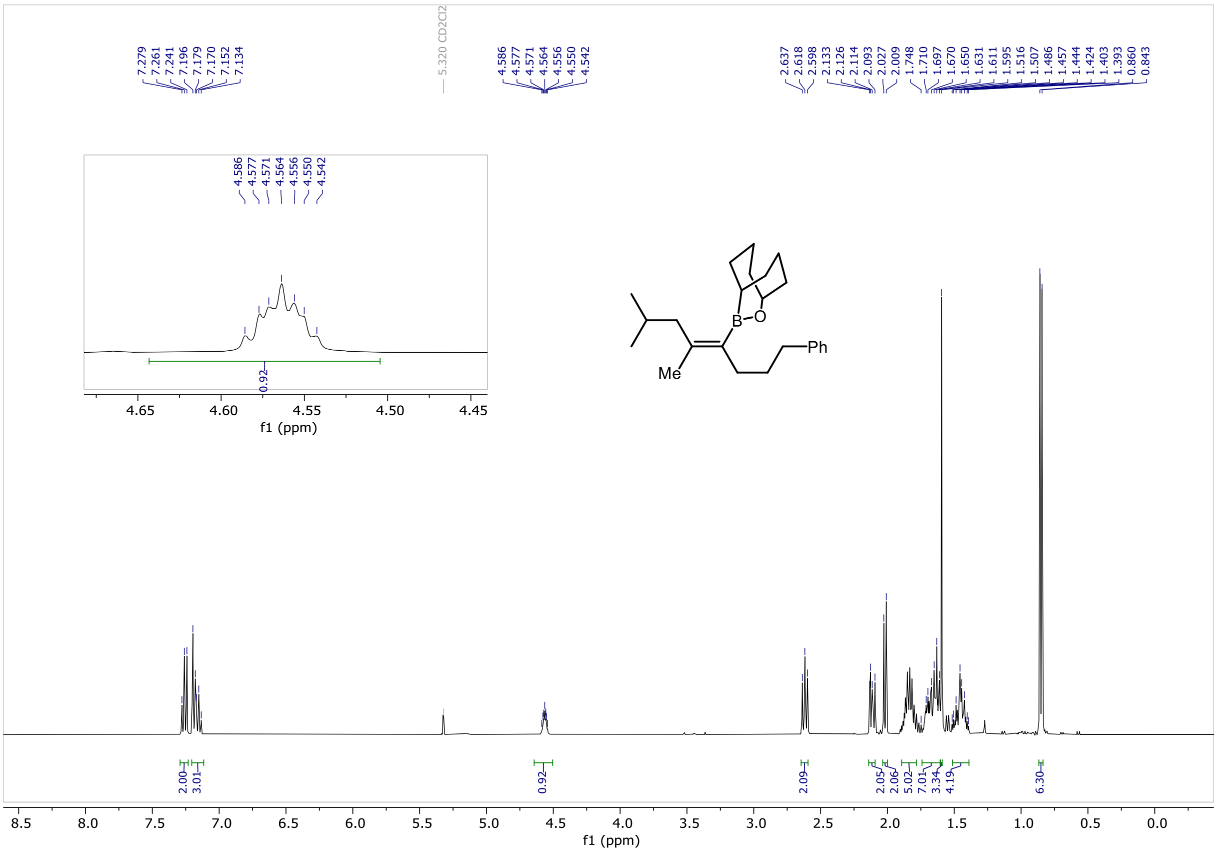

^{13}C NMR (400 MHz, CD_2Cl_2) of **6c**

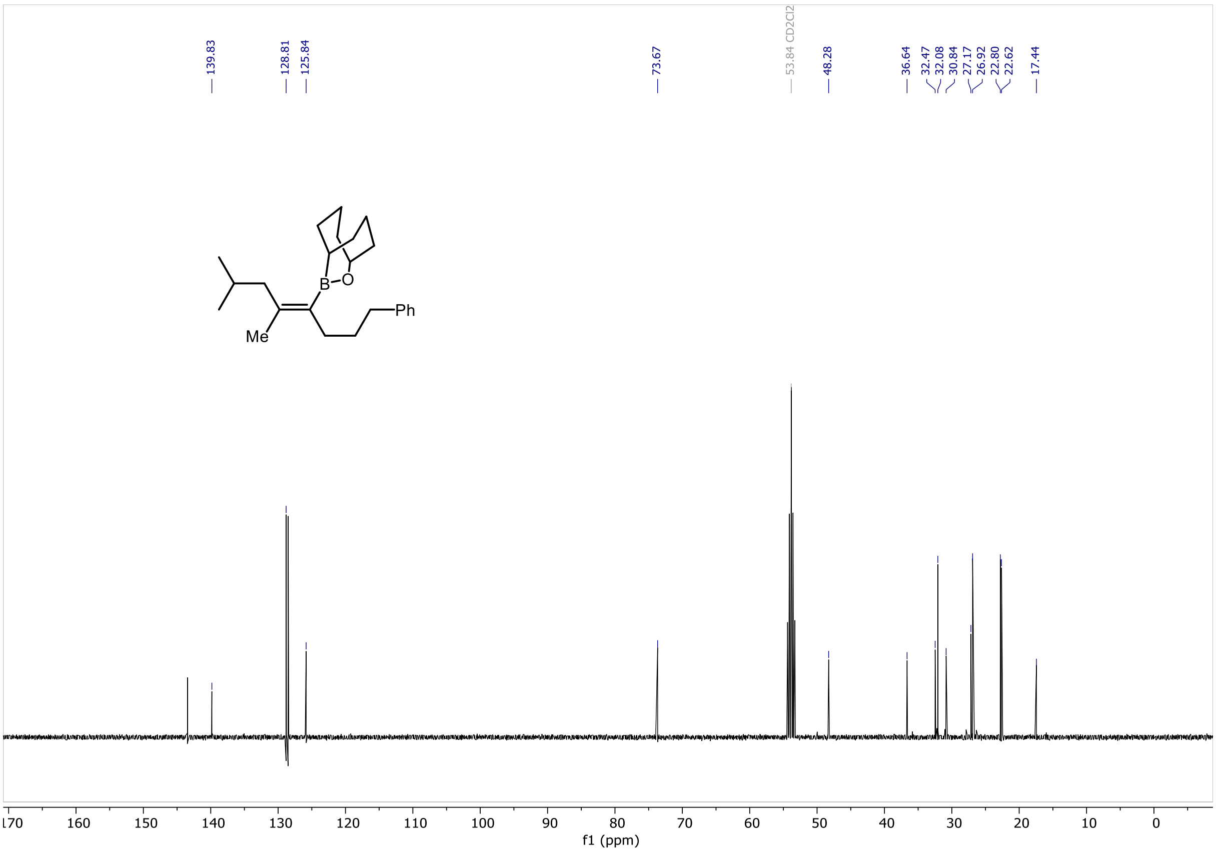

^{1}H NMR (400 MHz, CD_2Cl_2) of **6d**

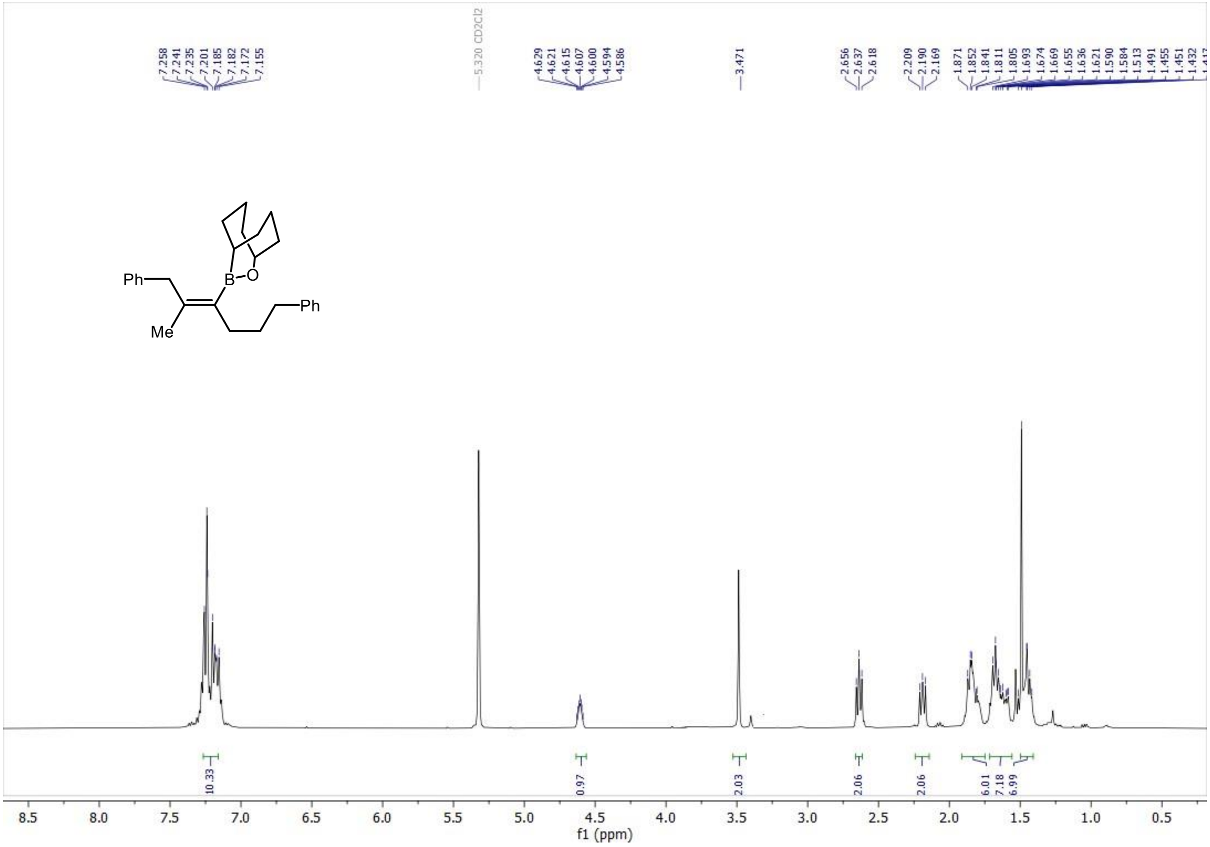

^{13}C NMR (400 MHz, CD_2Cl_2) of **6d**

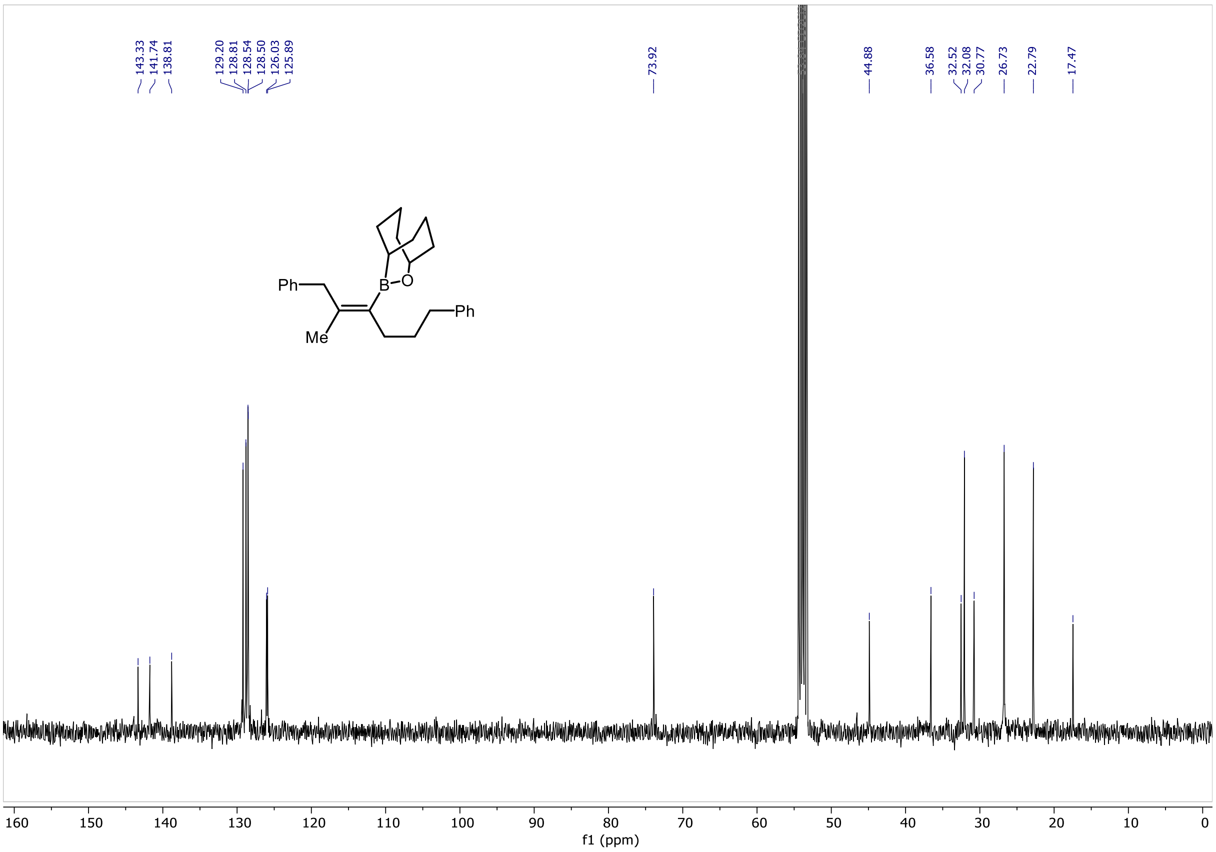

^{1}H NMR (400 MHz, CD_2Cl_2) of **6e**

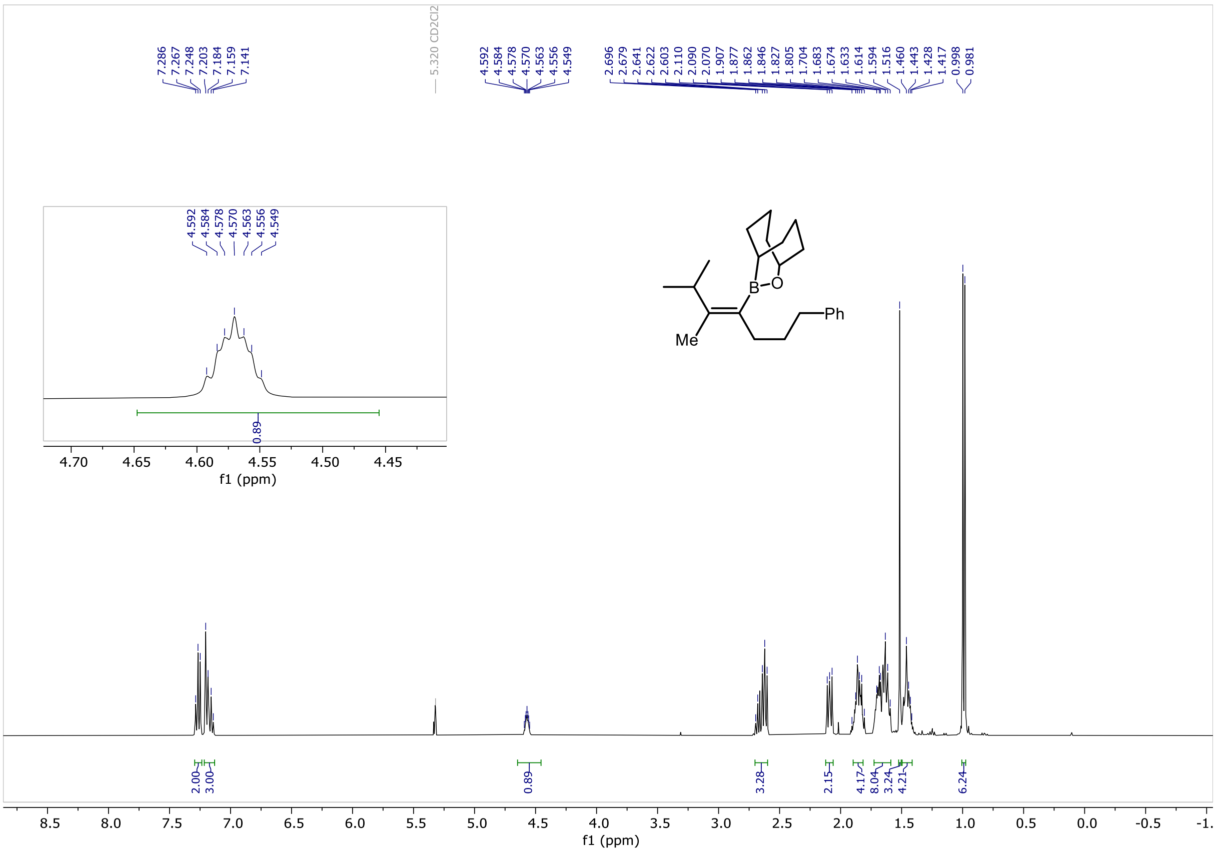

^{13}C NMR (400 MHz, CD_2Cl_2) of **6e**

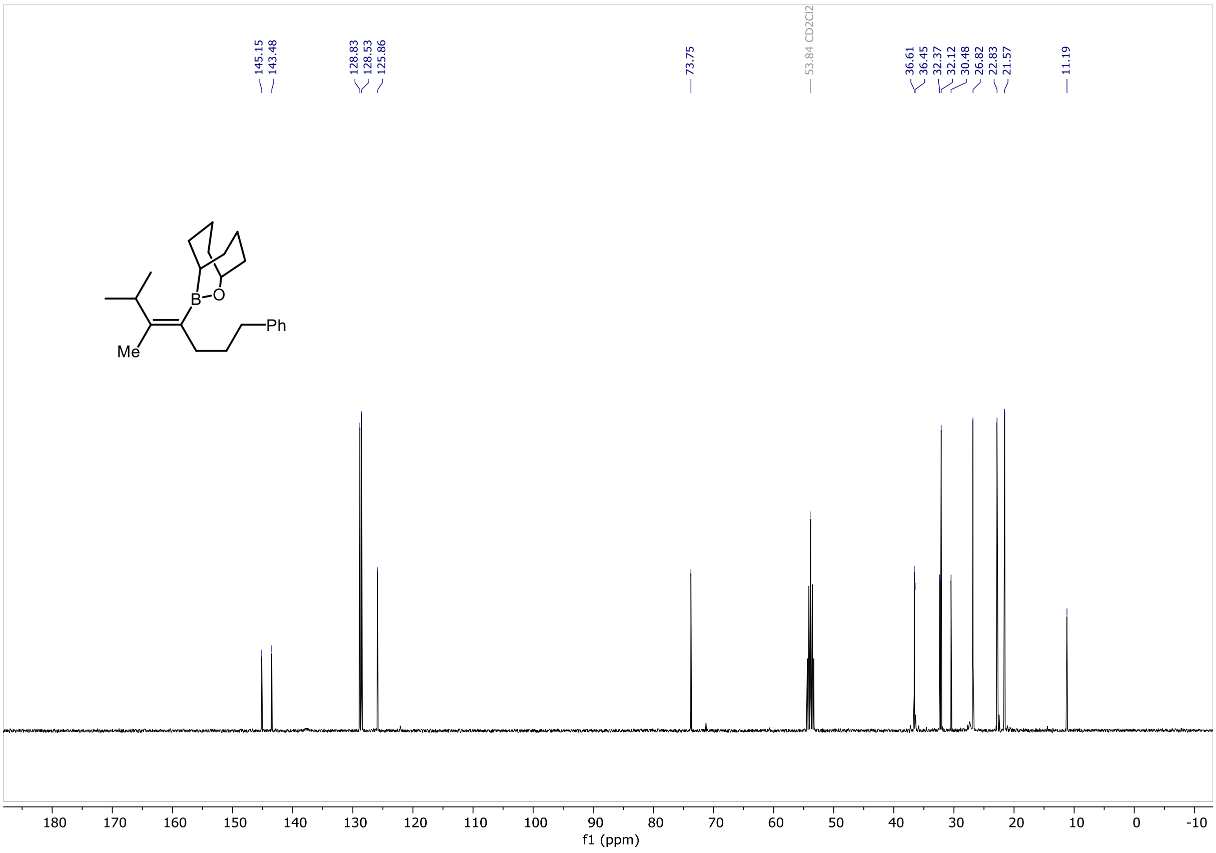

^{1}H NMR (400 MHz, CD_2Cl_2) of **6f**

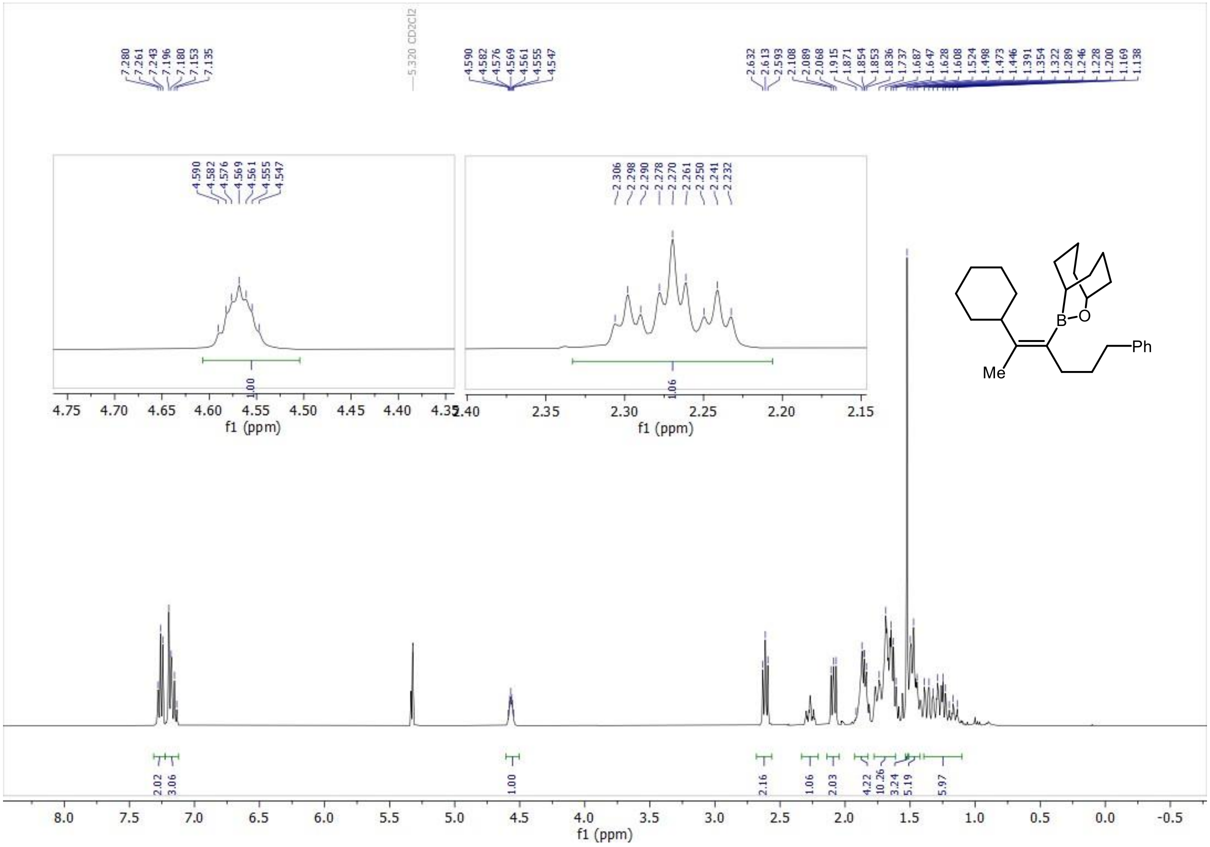

^{13}C NMR (400 MHz, CD_2Cl_2) of **6f**

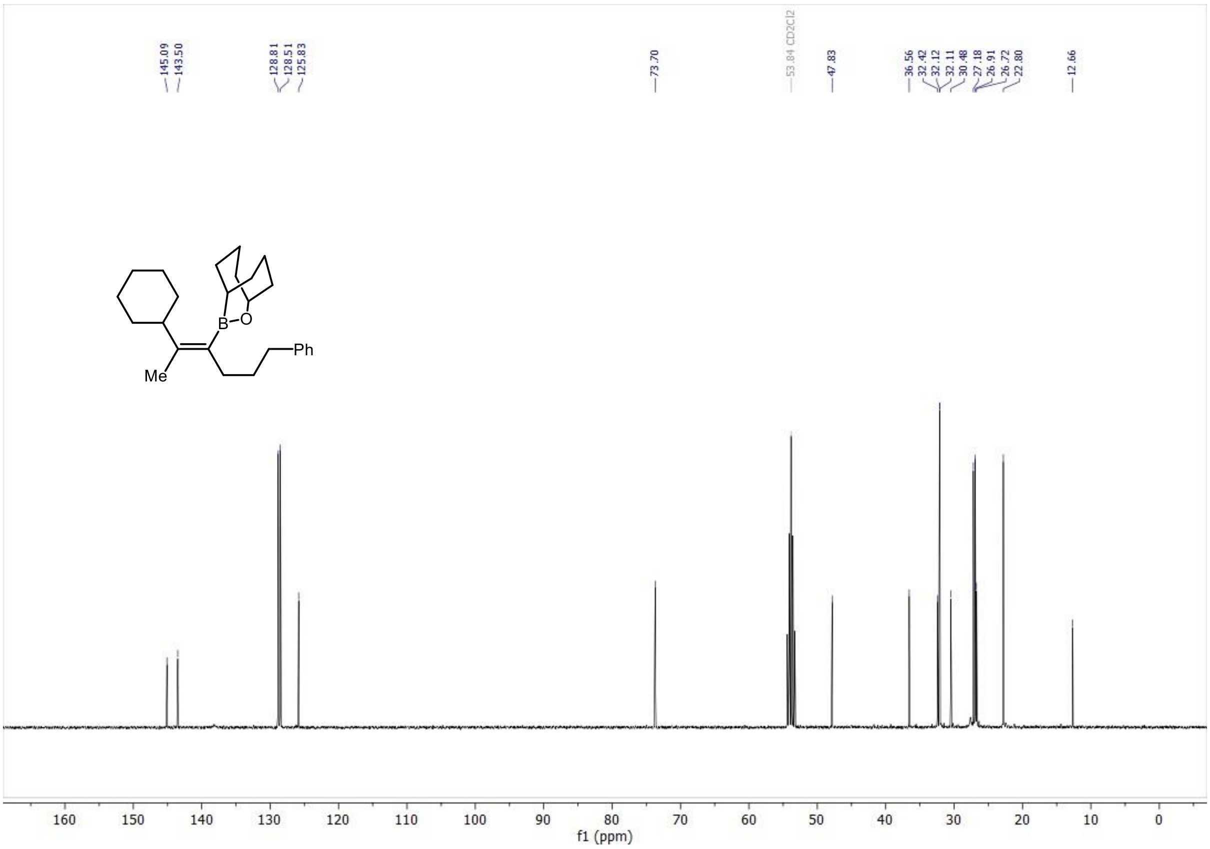

^{1}H NMR (400 MHz, $CDCl_3$) of **6g**

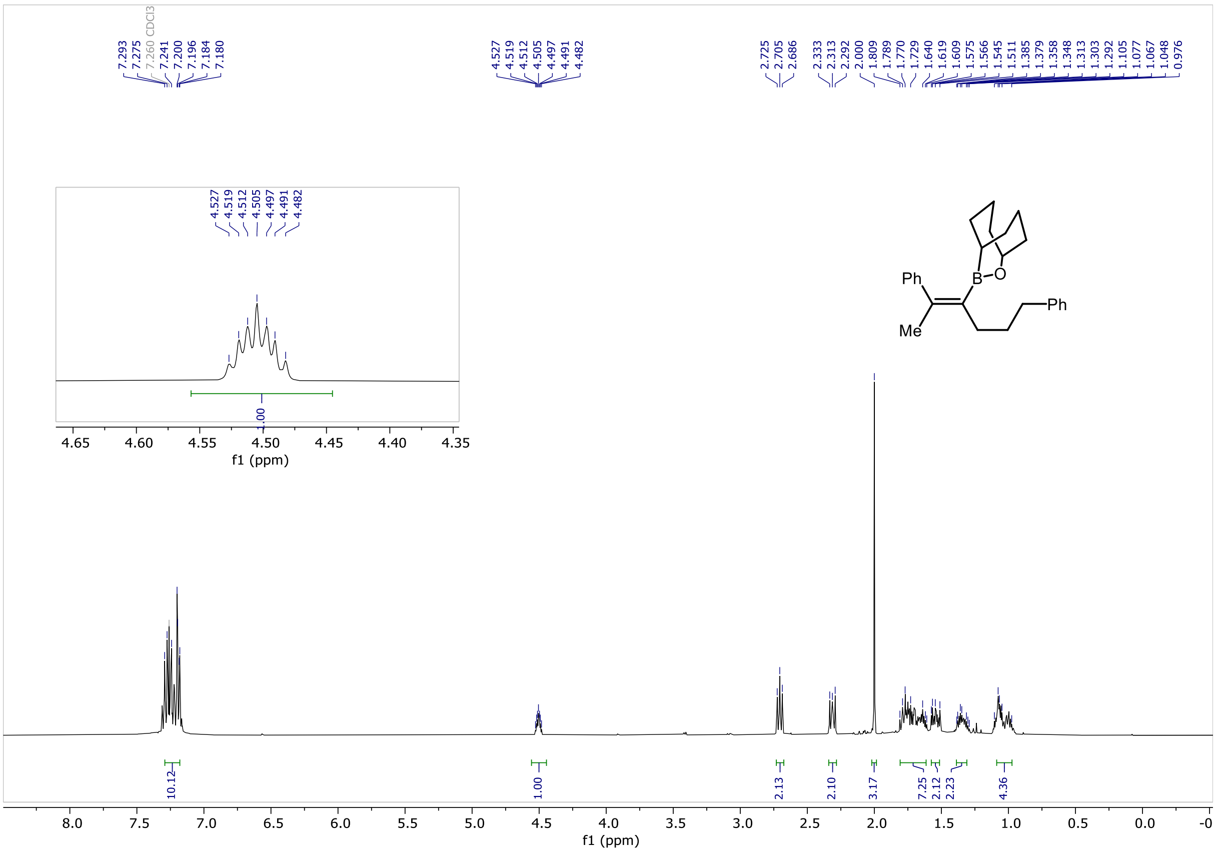

^{13}C NMR (400 MHz, $CDCl_3$) of **6g**

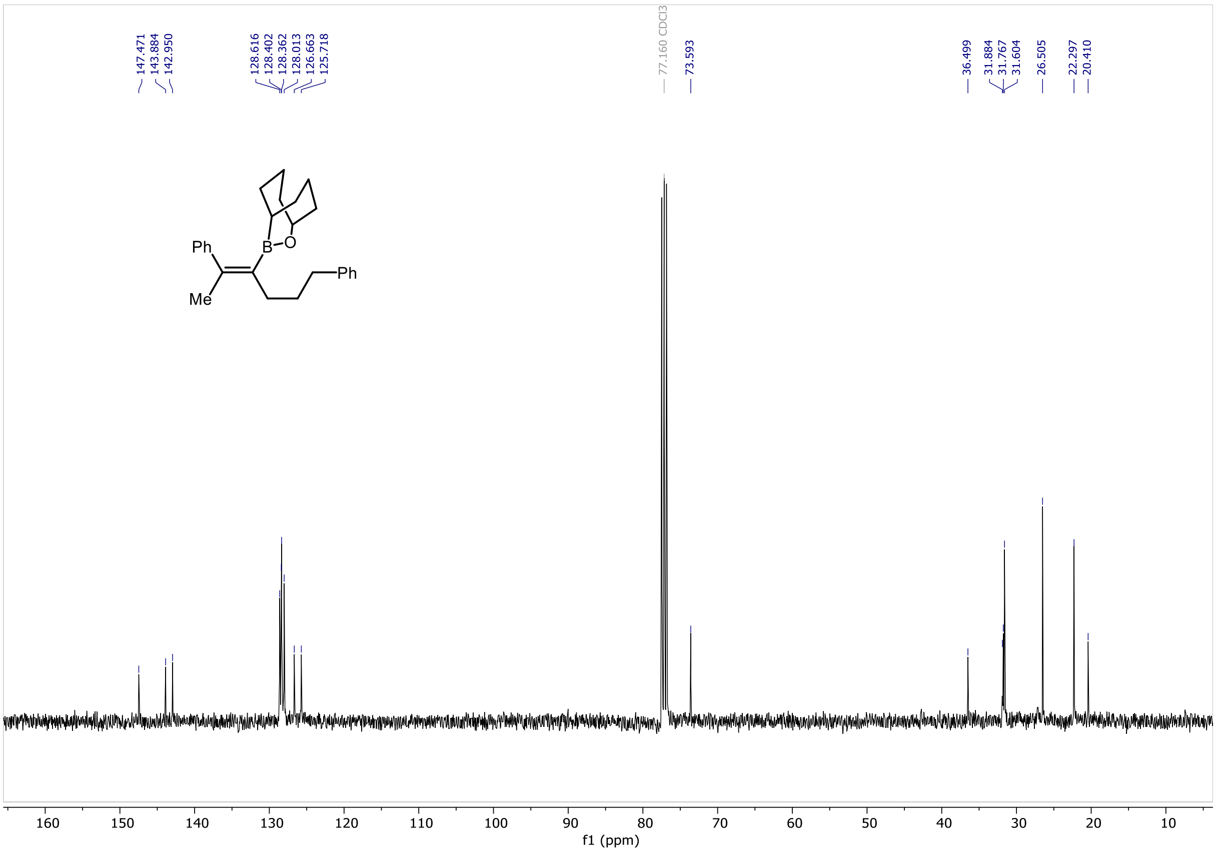

^{1}H NMR (400 MHz, CD_2Cl_2) of **6h**

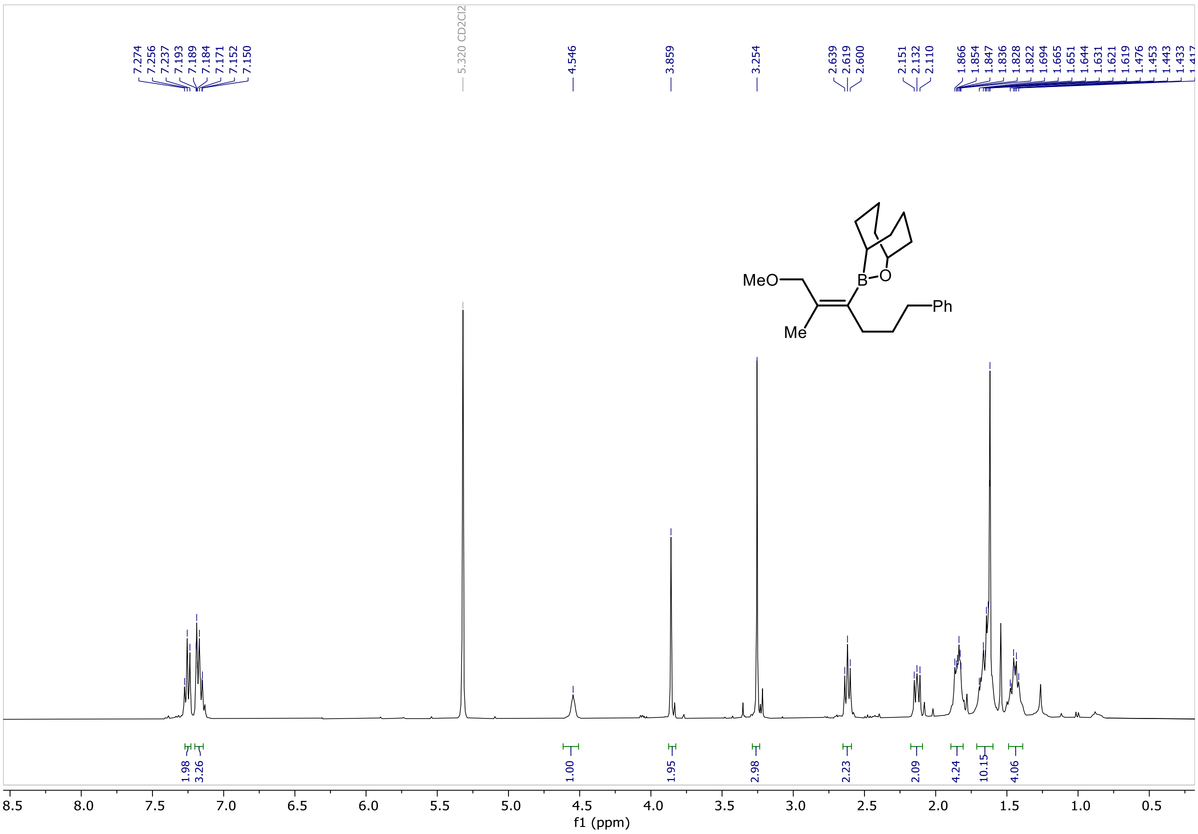

^{13}C NMR (400 MHz, CD_2Cl_2) of **6h**

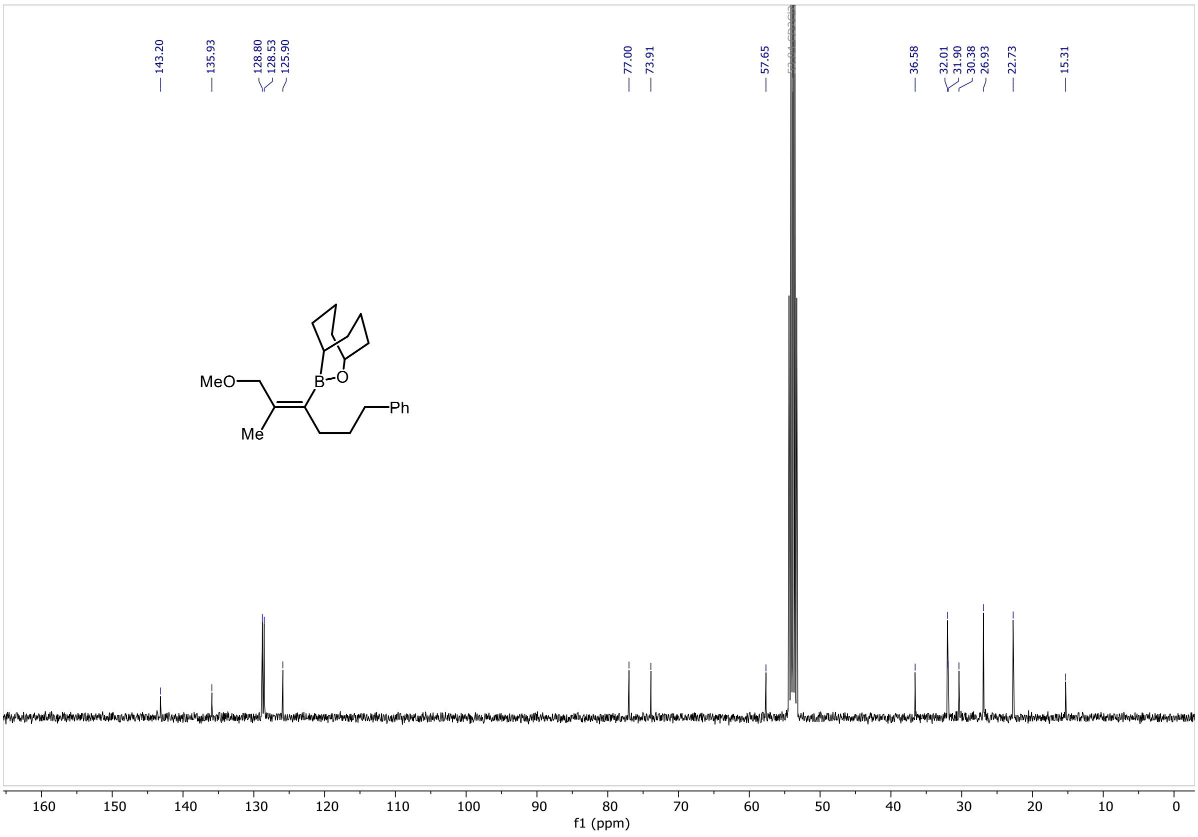

^{1}H NMR (400 MHz, CD_2Cl_2) of **6i**

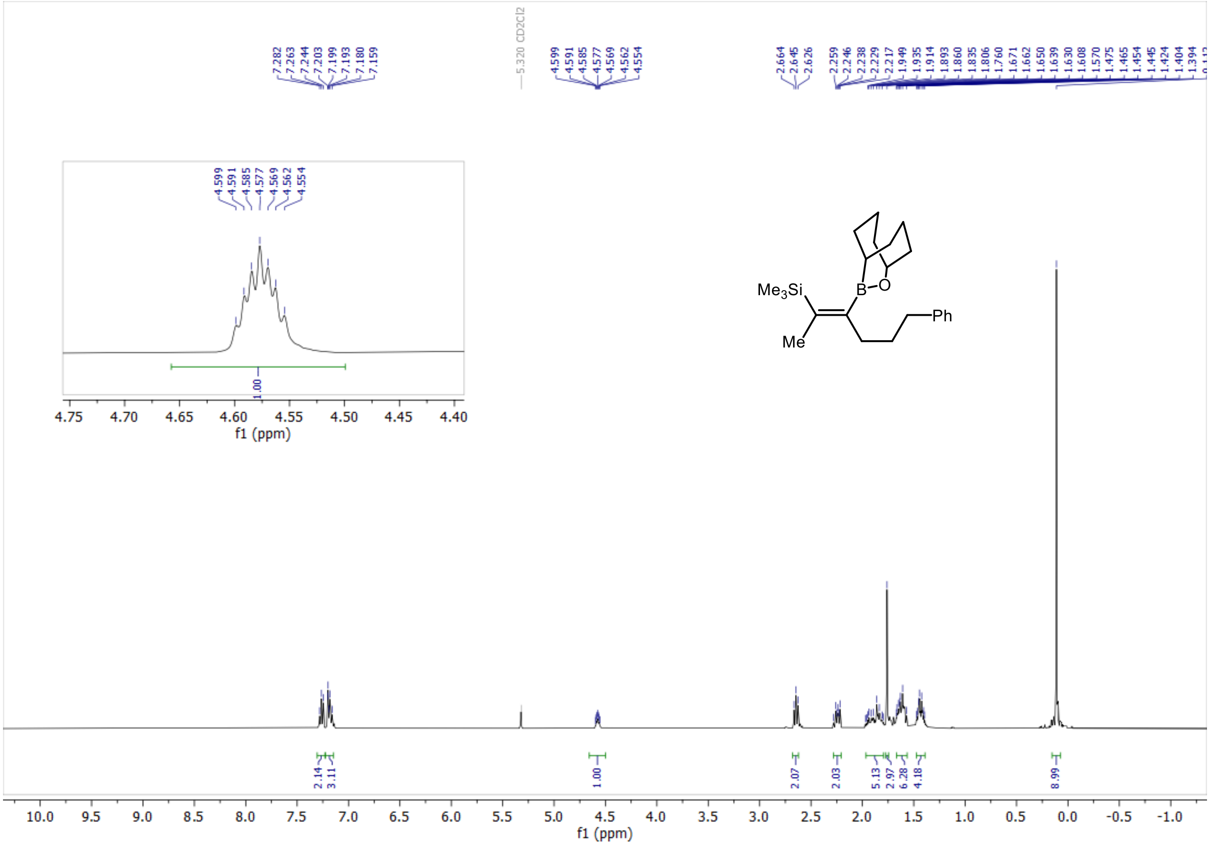

^{13}C NMR (400 MHz, CD_2Cl_2) of **6i**

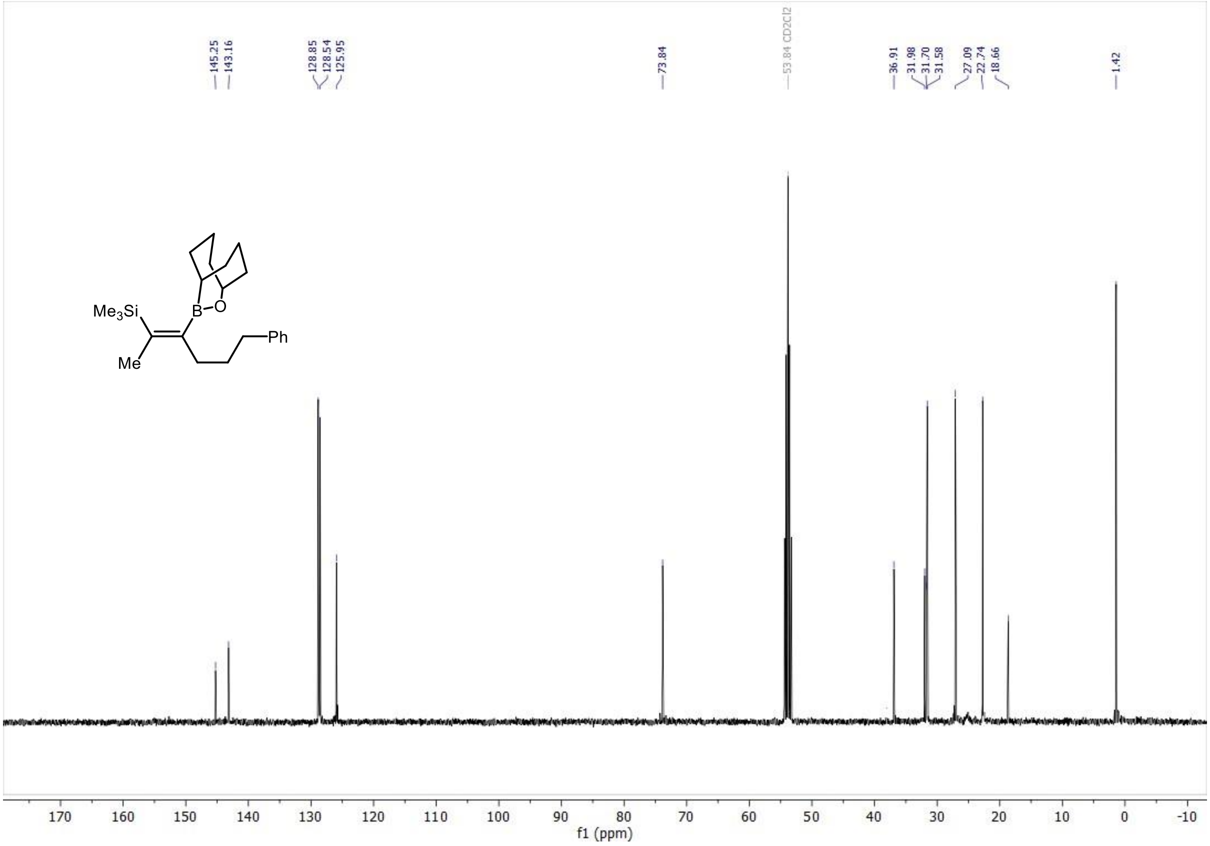

^{1}H NMR (400 MHz, CD_2Cl_2) of **6j**

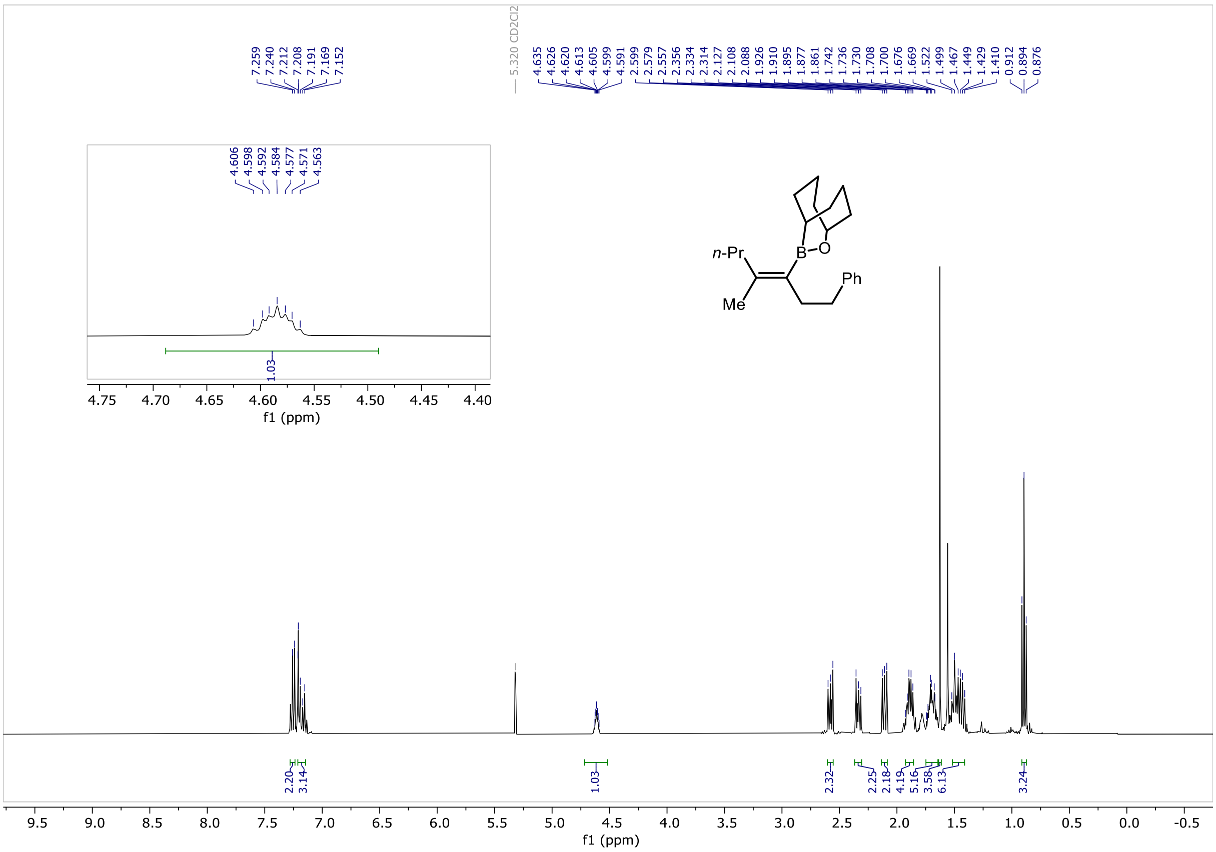

^{13}C NMR (400 MHz, CD_2Cl_2) of **6j**

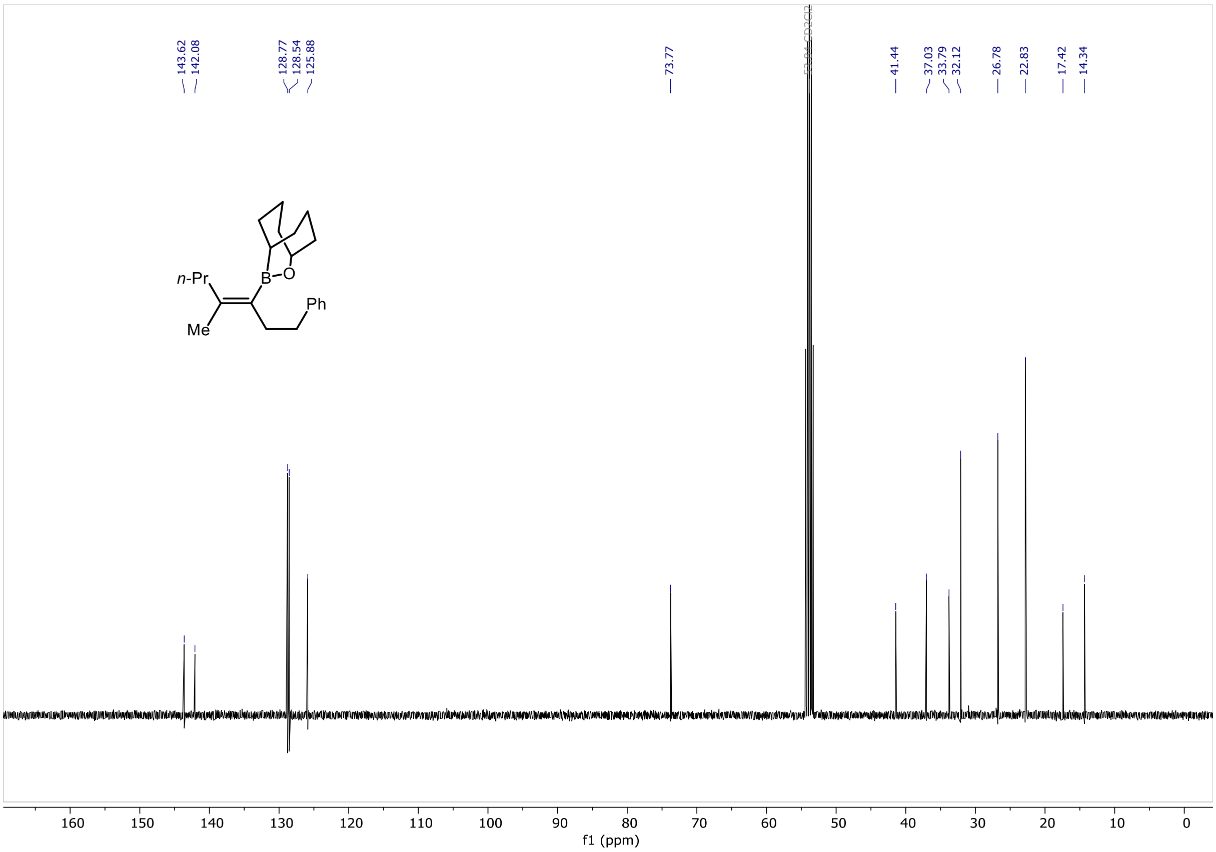

^{1}H NMR (400 MHz, CD_2Cl_2) of **6k**

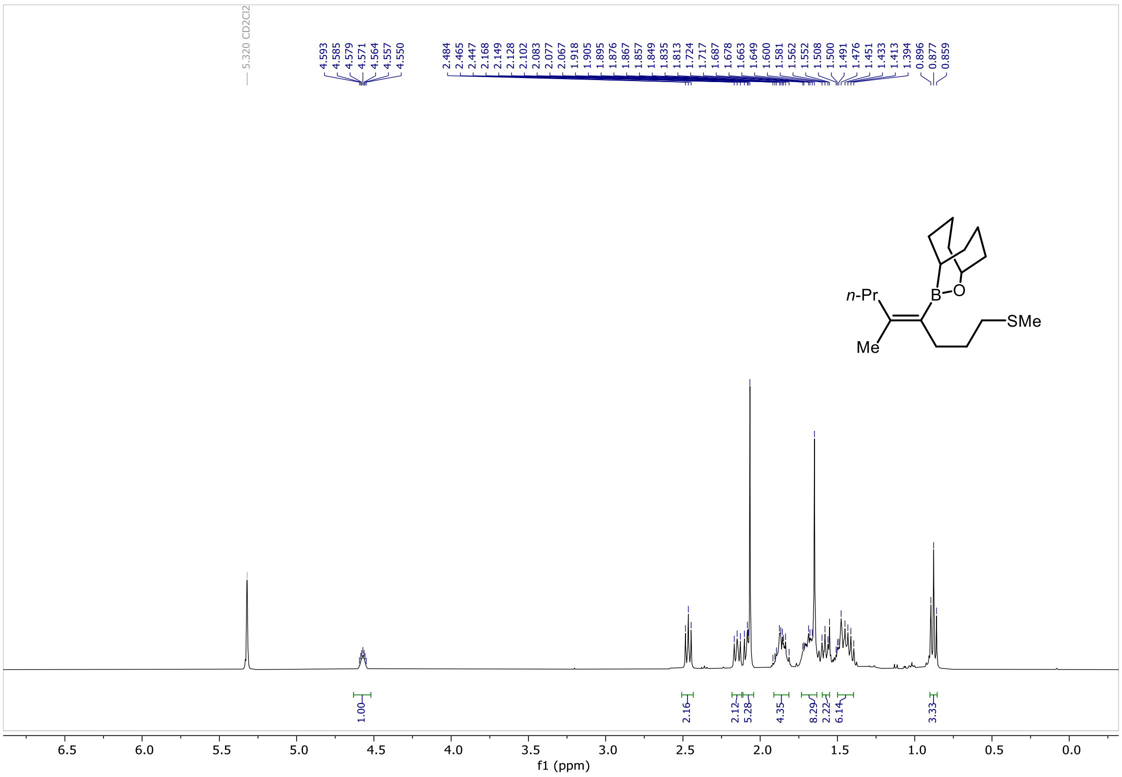

^{13}C NMR (400 MHz, CD_2Cl_2) of **6k**

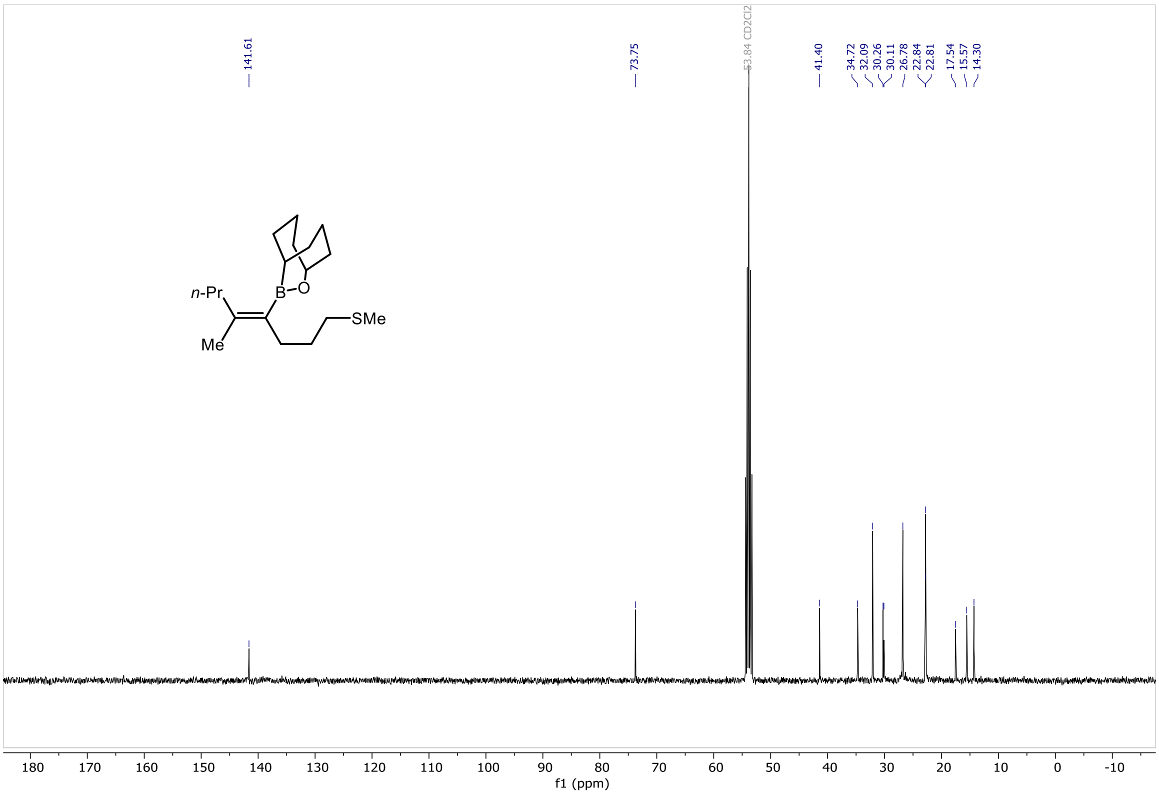

^{1}H NMR (400 MHz, CD_2Cl_2) of **6l**

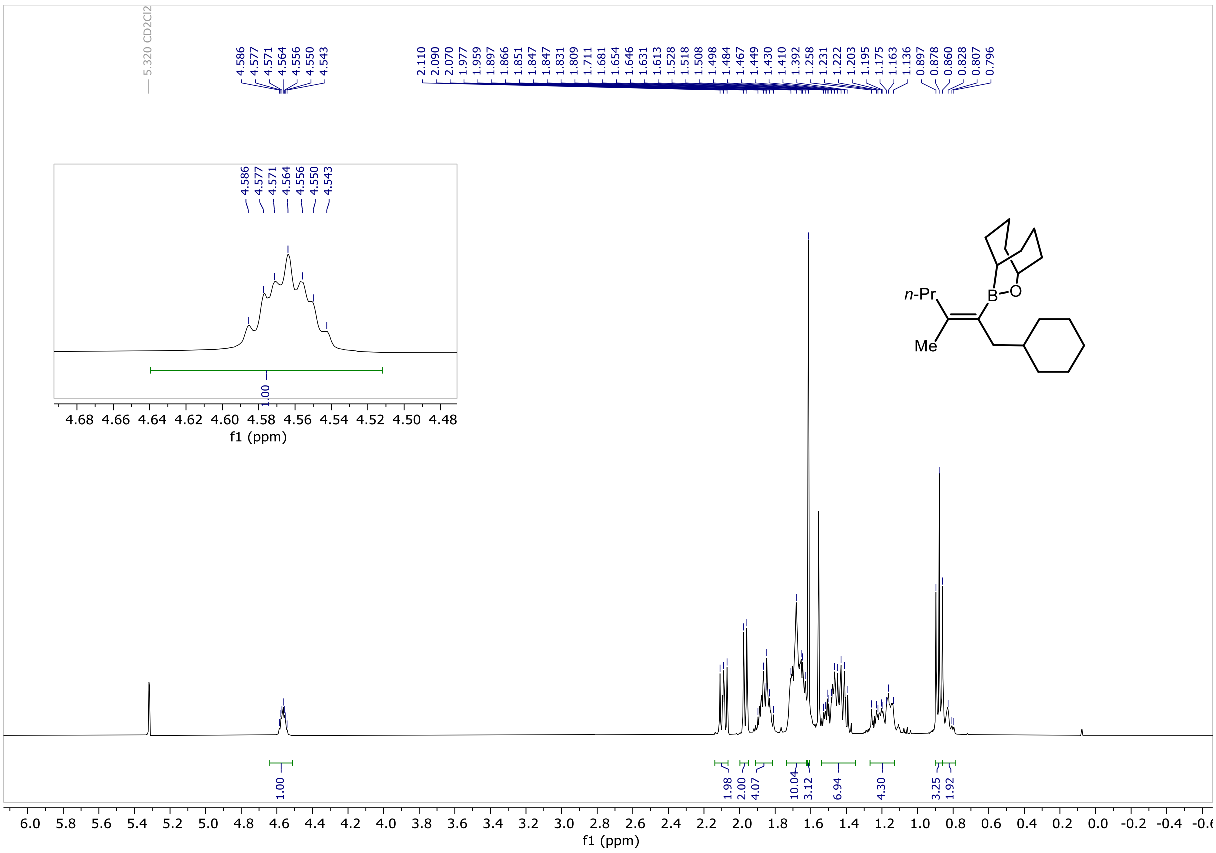

^{13}C NMR (400 MHz, CD_2Cl_2) of **6l**

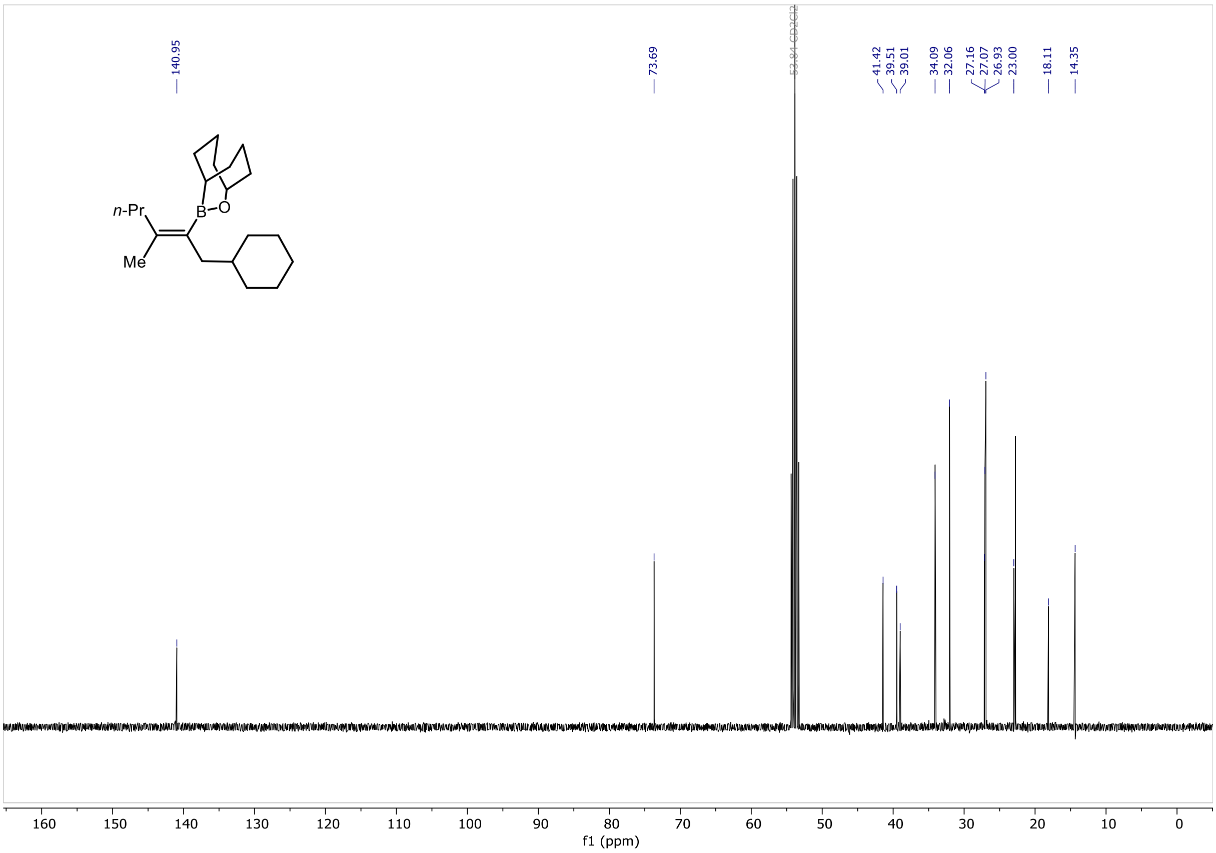

^{1}H NMR (400 MHz, CD_2Cl_2) of **6m**

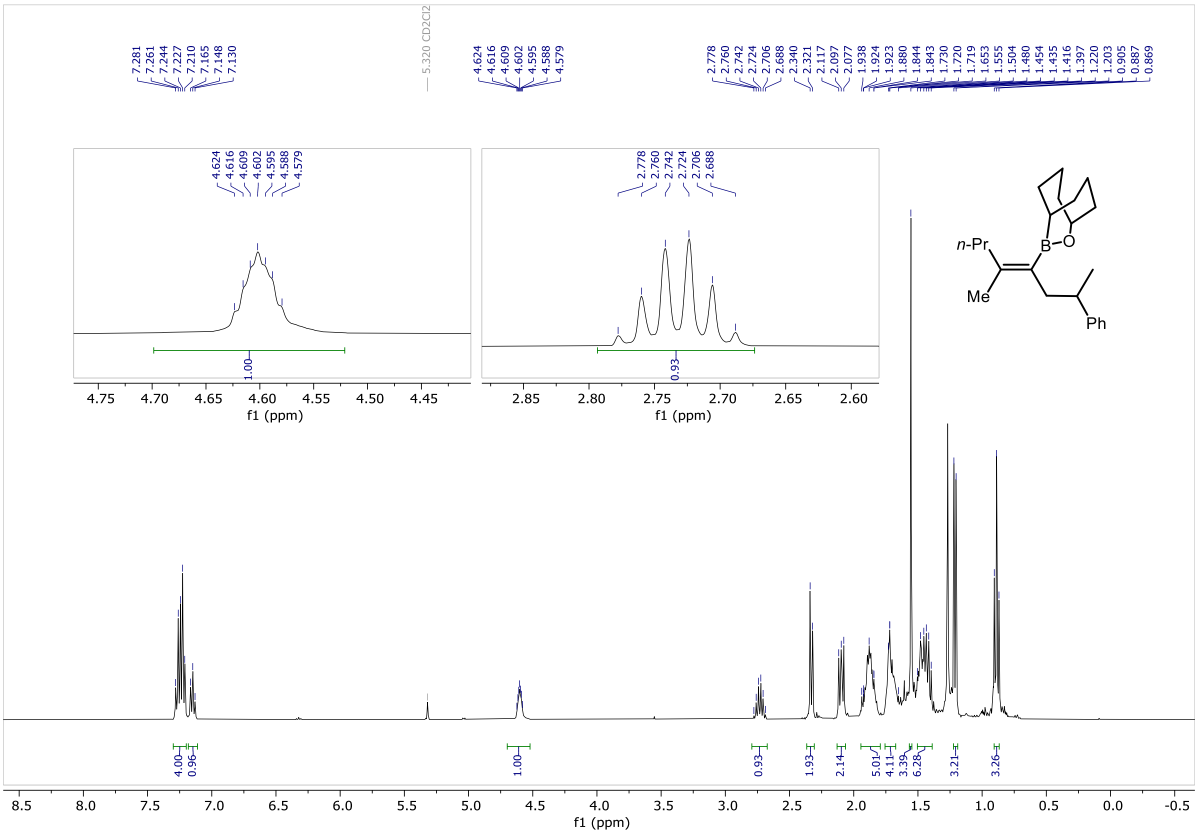

^{13}C NMR (400 MHz, CD_2Cl_2) of **6m**

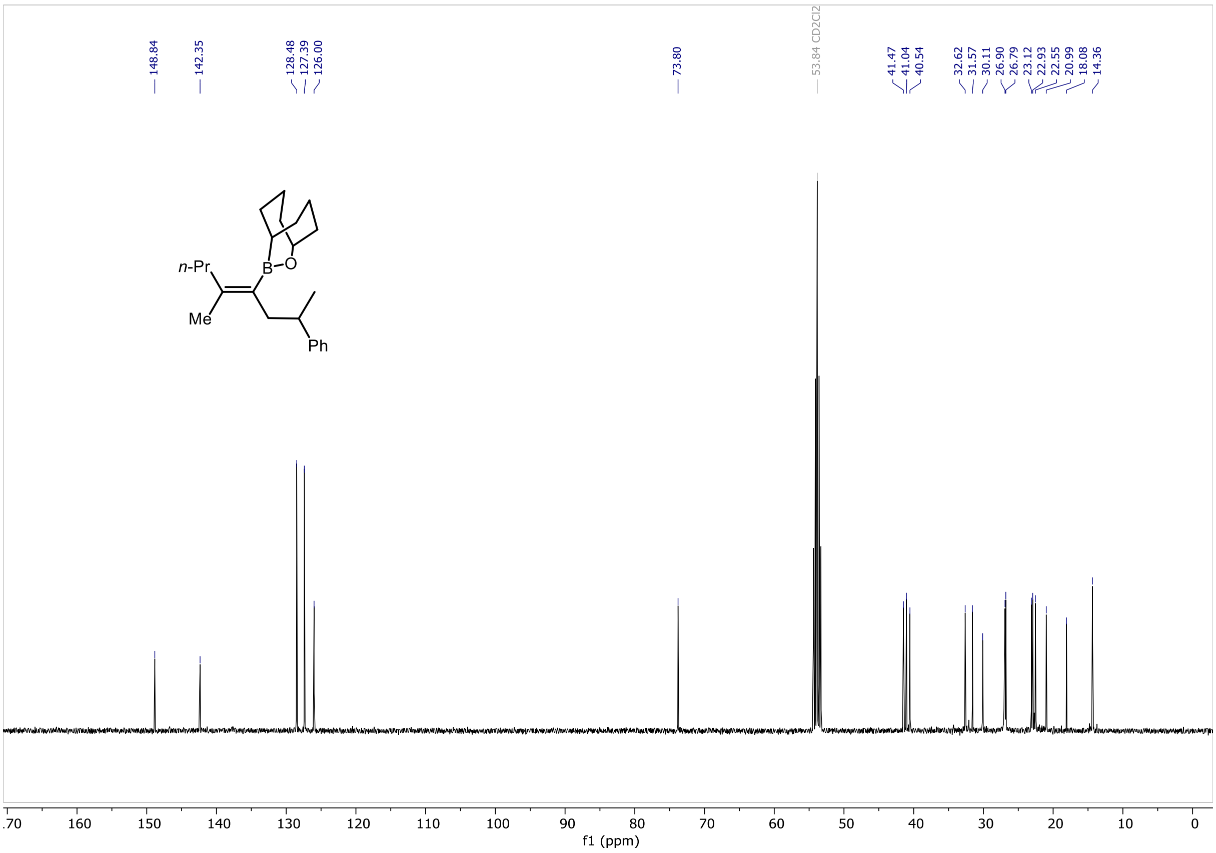

^{1}H NMR (400 MHz, CD_2Cl_2) of **6n**

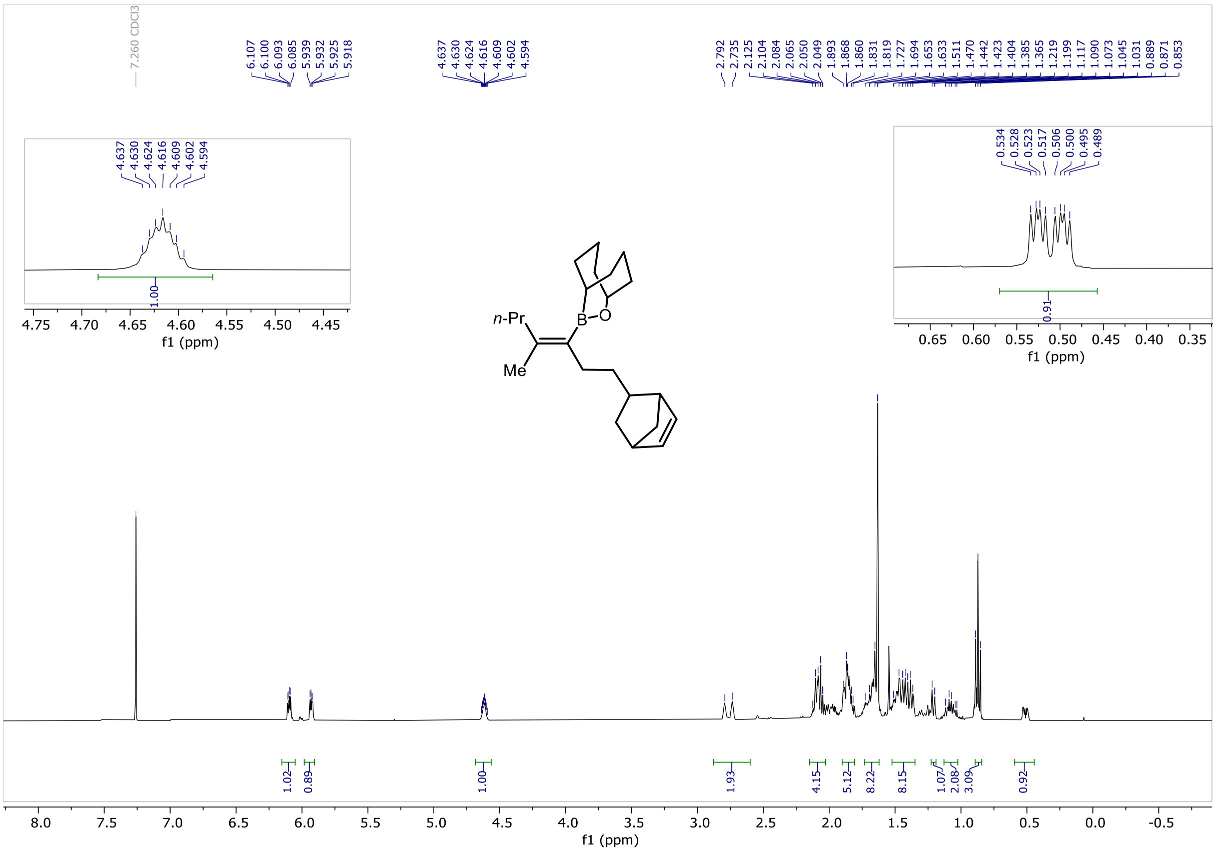

^{13}C NMR (400 MHz, CD_2Cl_2) of **6n**

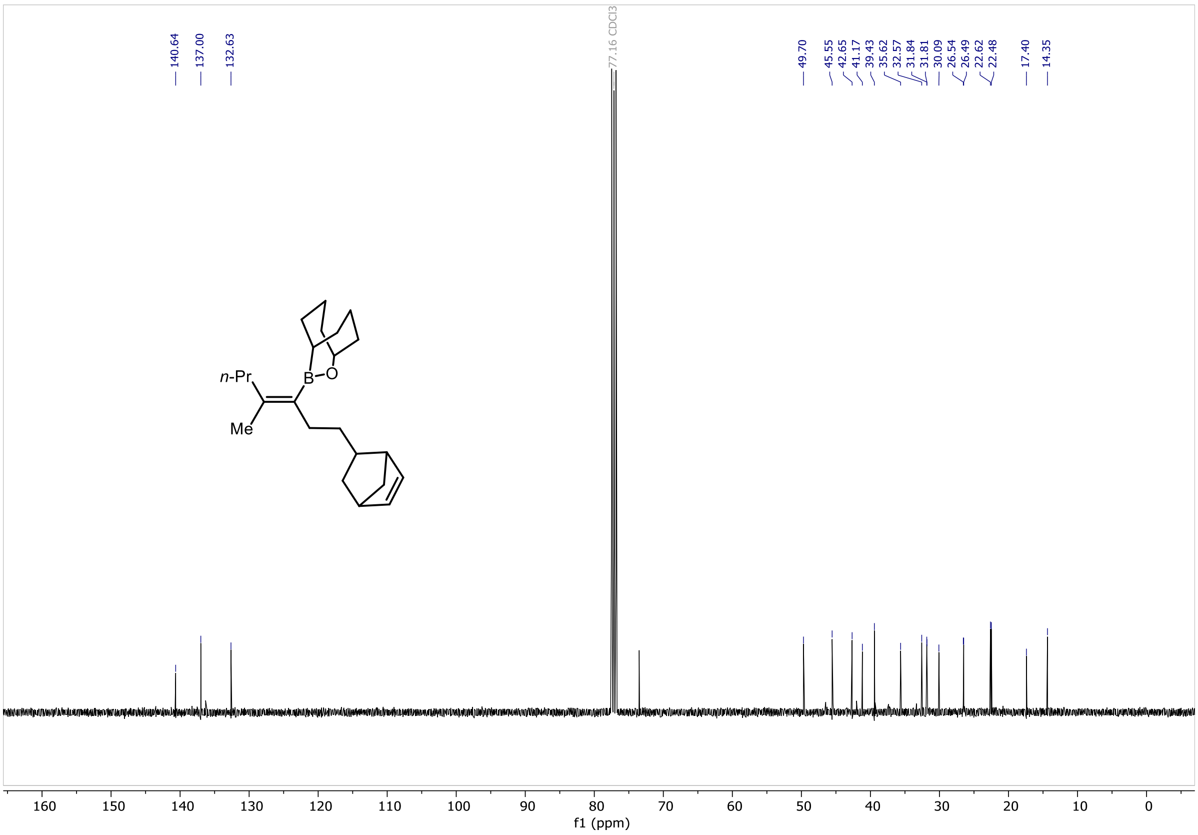

^{1}H NMR (400 MHz, CD_2Cl_2) of **6o**

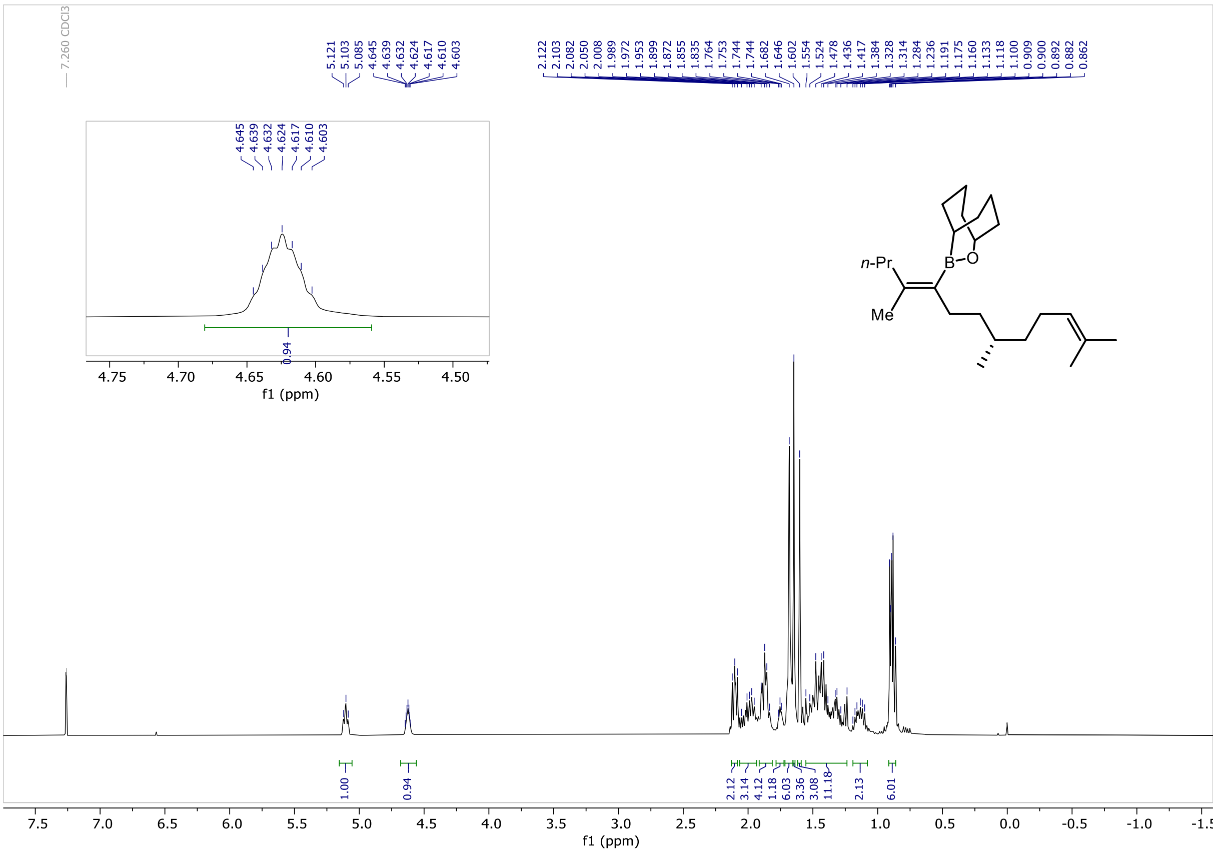

^{13}C NMR (400 MHz, CD_2Cl_2) of **6o**

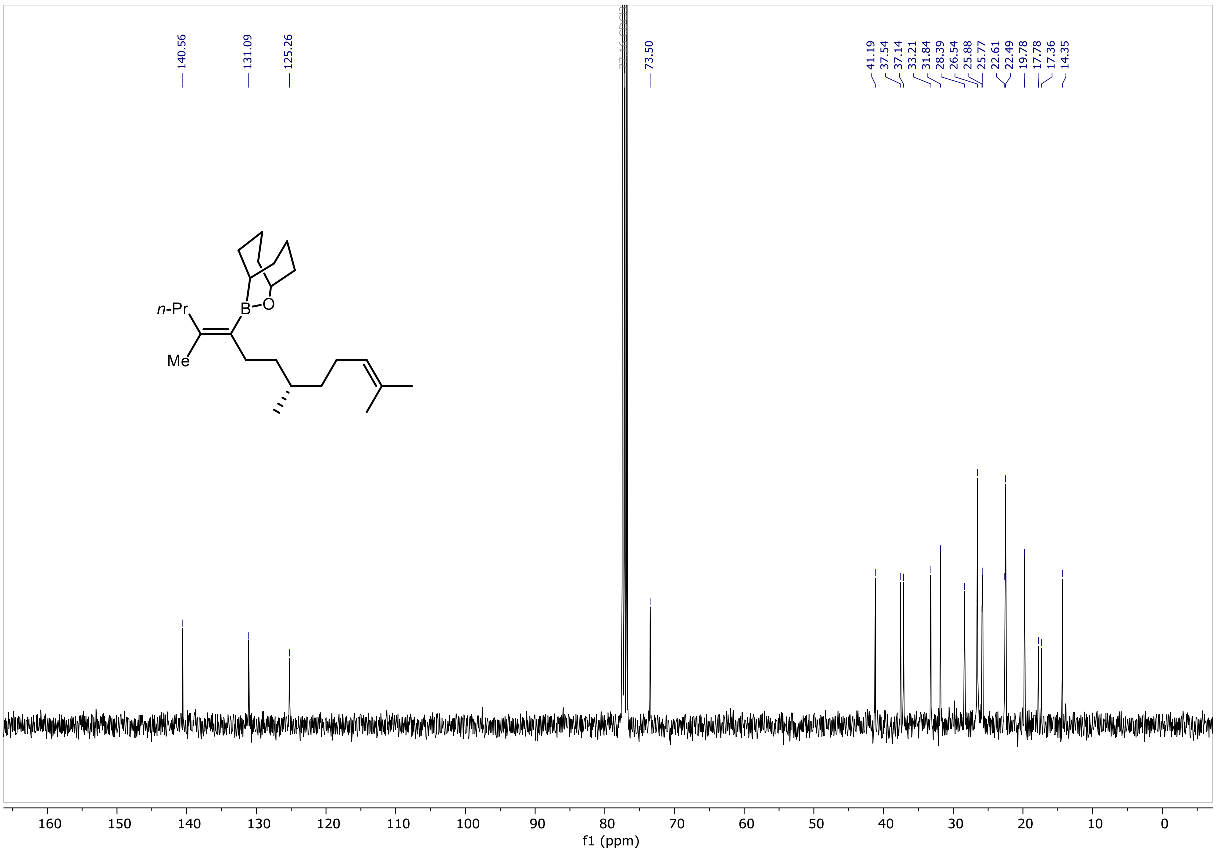

^{1}H NMR (400 MHz, CD_2Cl_2) of **6p**

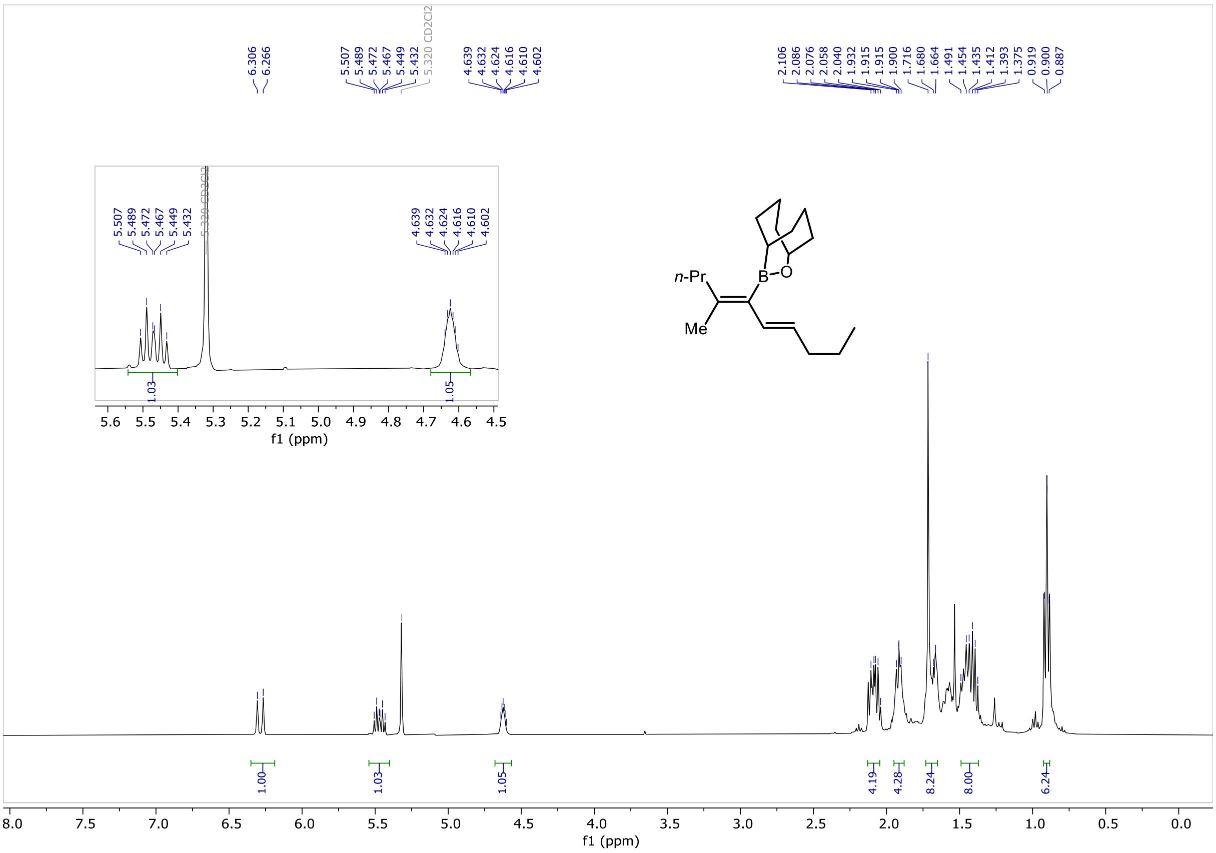

^{13}C NMR (400 MHz, CD_2Cl_2) of **6p**

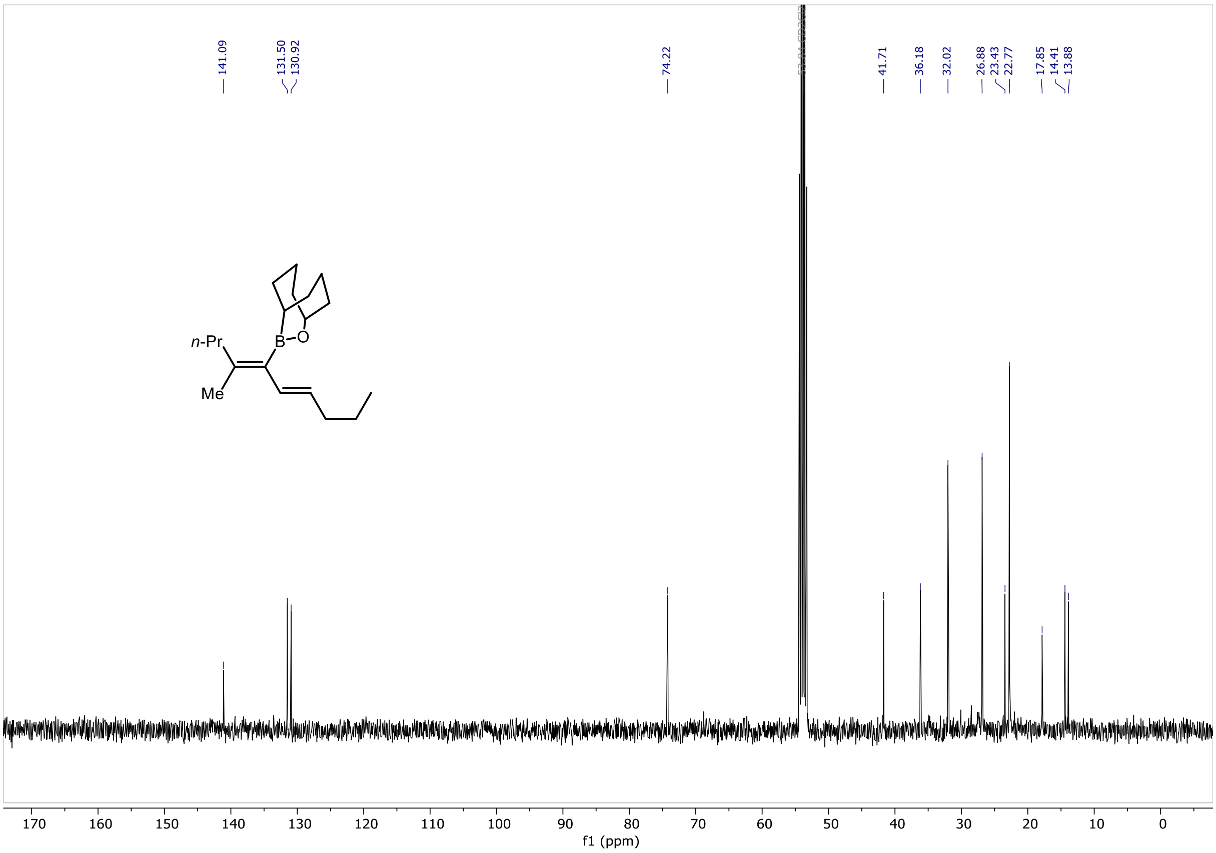

^{1}H NMR (400 MHz, CD_2Cl_2) of **6q**

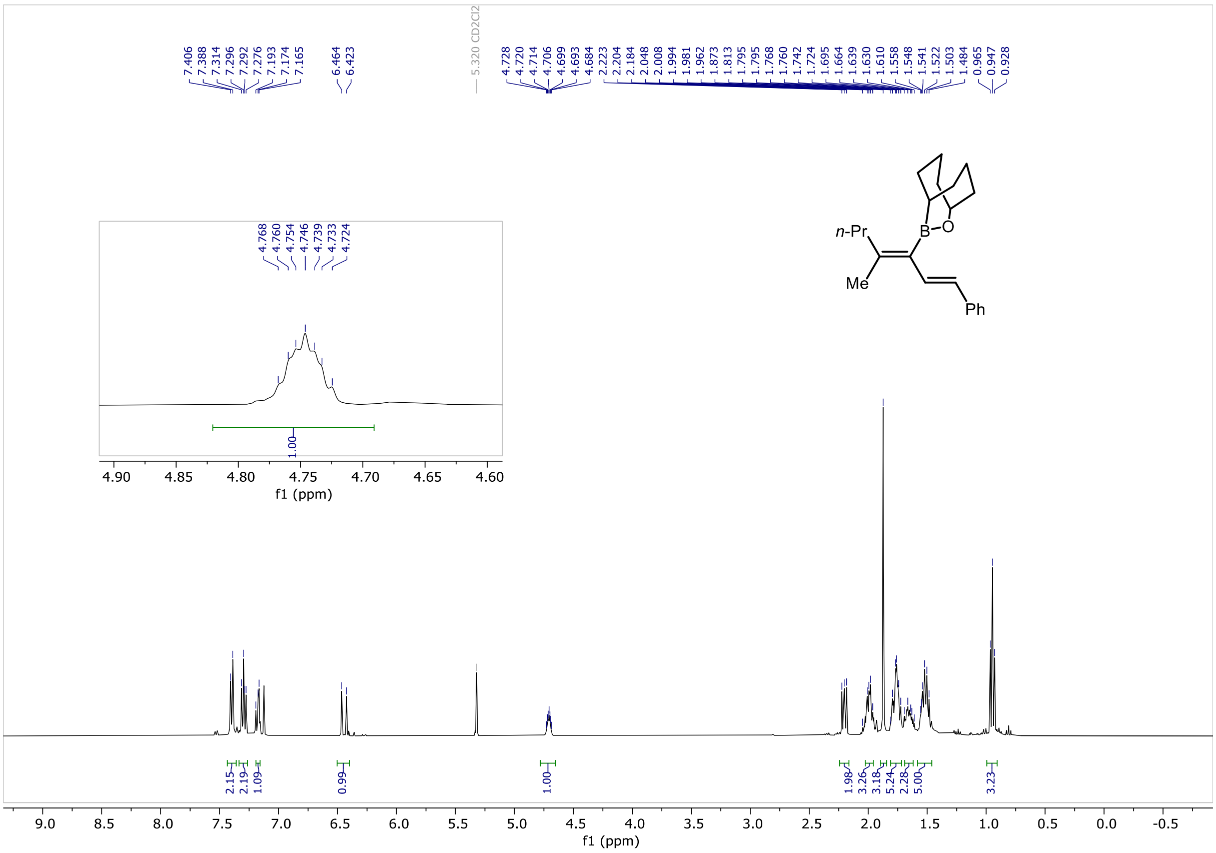

^{13}C NMR (400 MHz, CD_2Cl_2) of **6q**

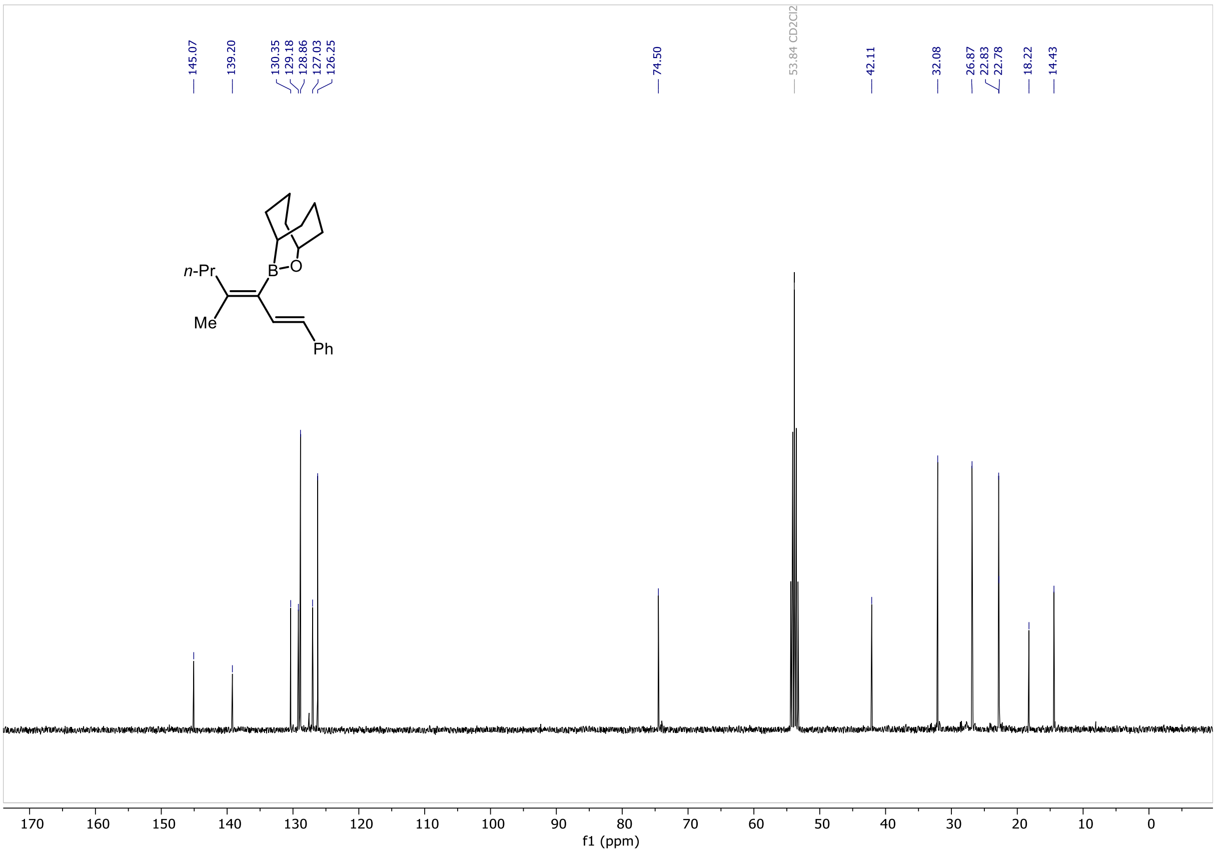

^{1}H NMR (400 MHz, CD_2Cl_2) of **6r**

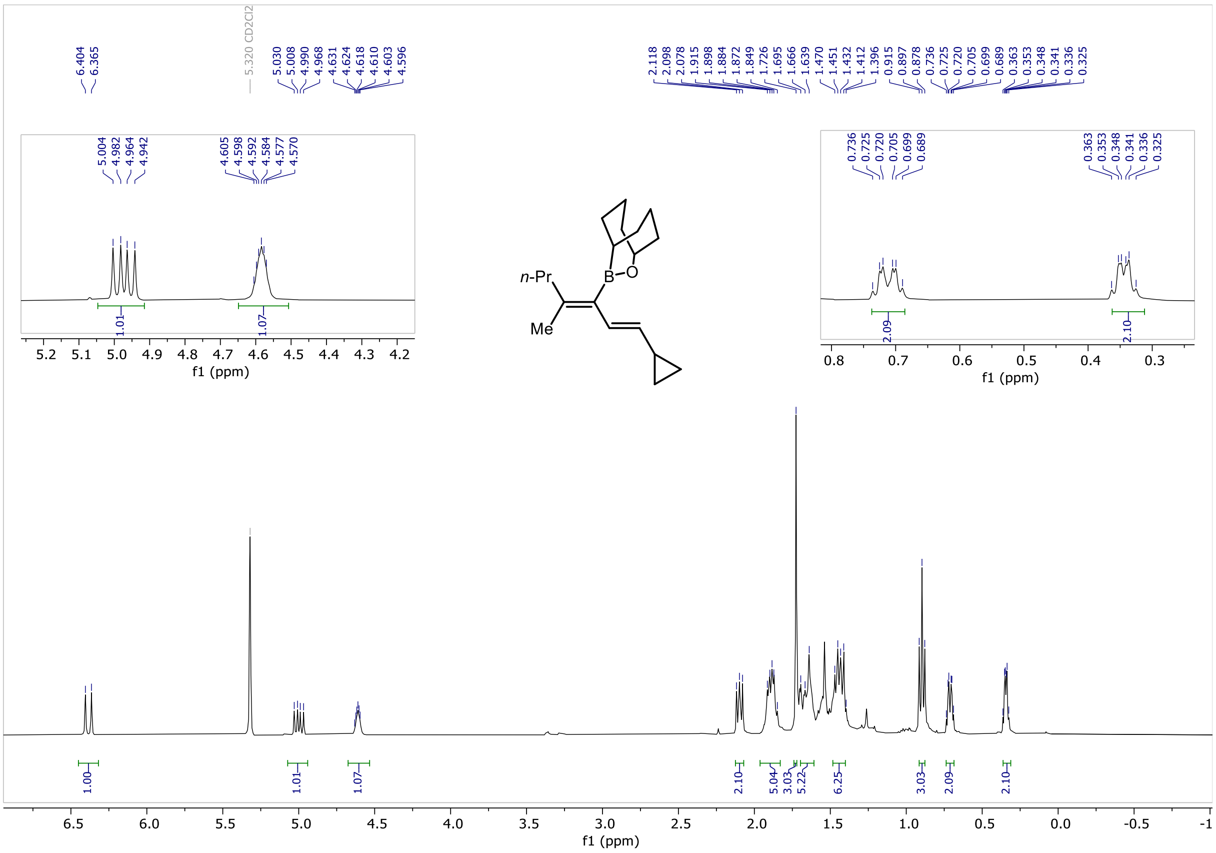

^{13}C NMR (400 MHz, CD_2Cl_2) of **6r**

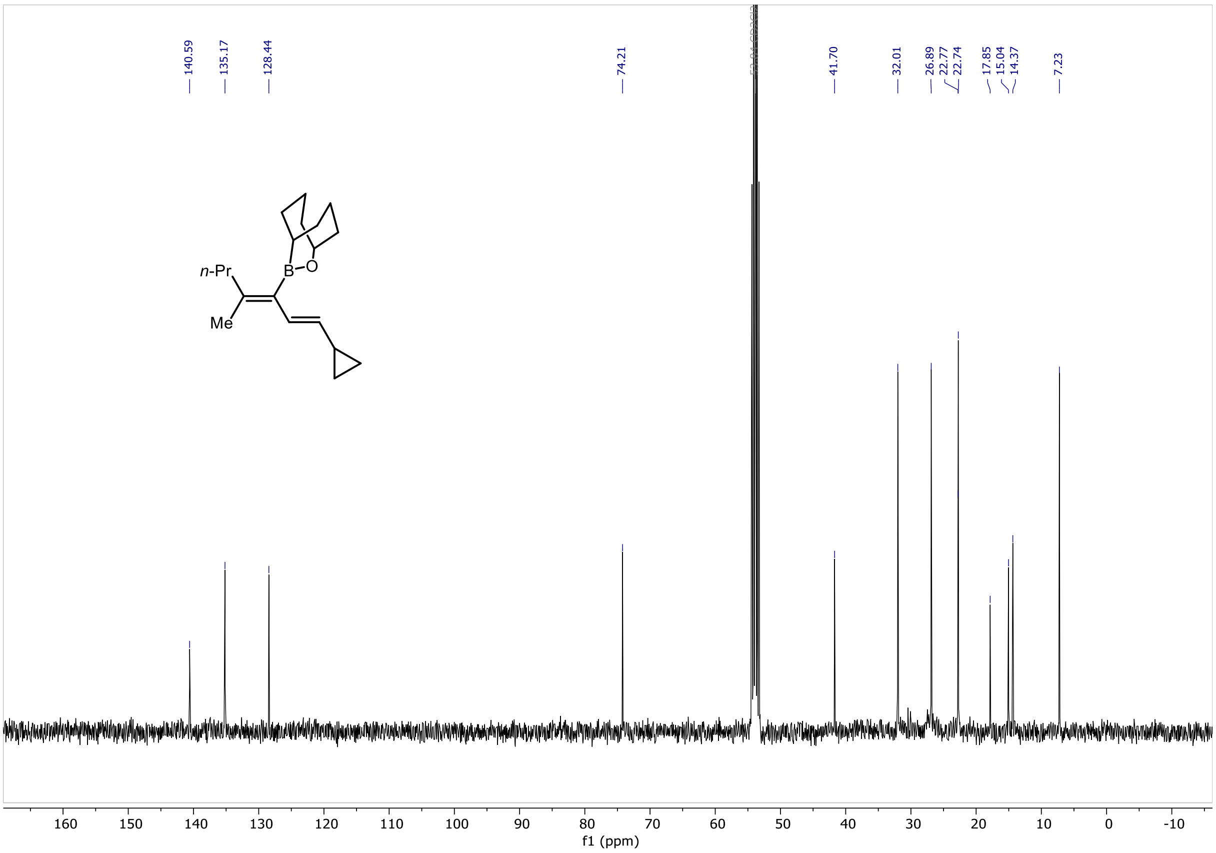

^{1}H NMR (400 MHz, CD_2Cl_2) of **6s**

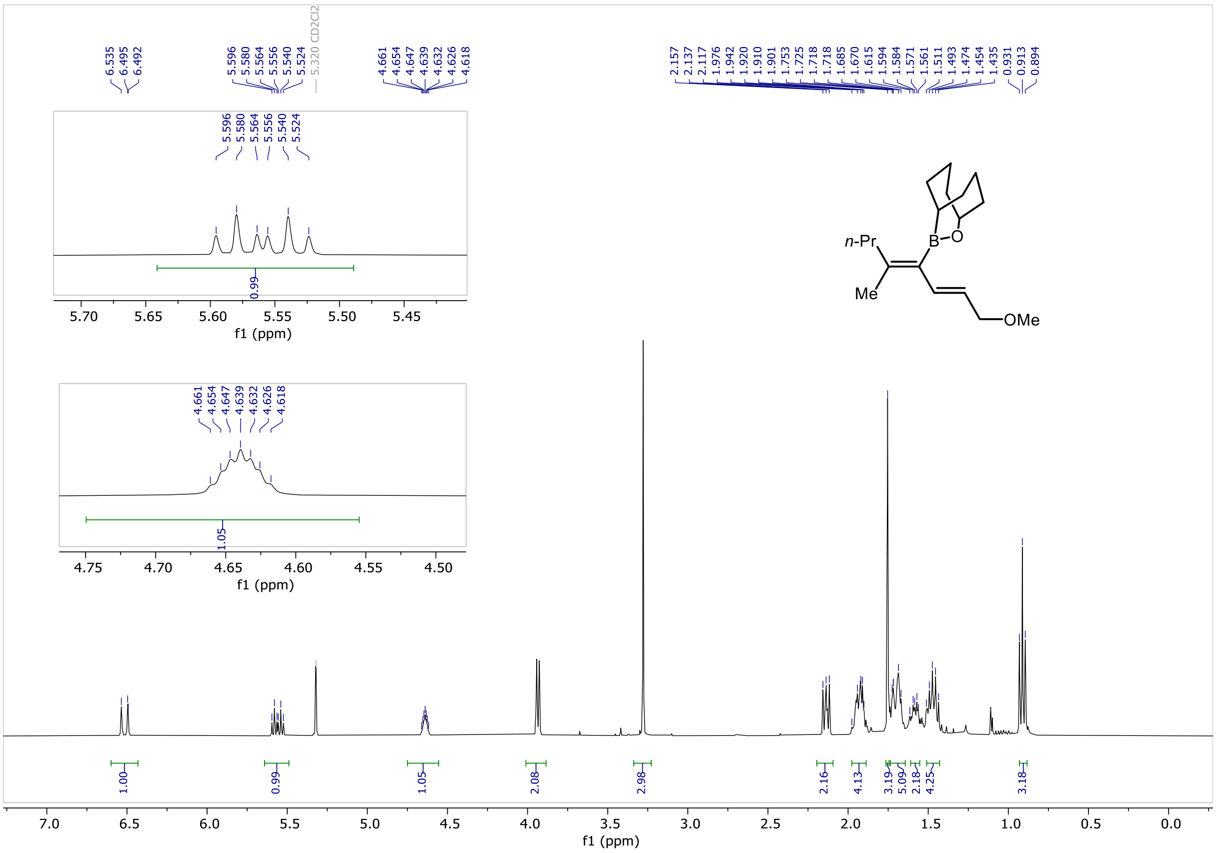

^{13}C NMR (400 MHz, CD_2Cl_2) of **6s**

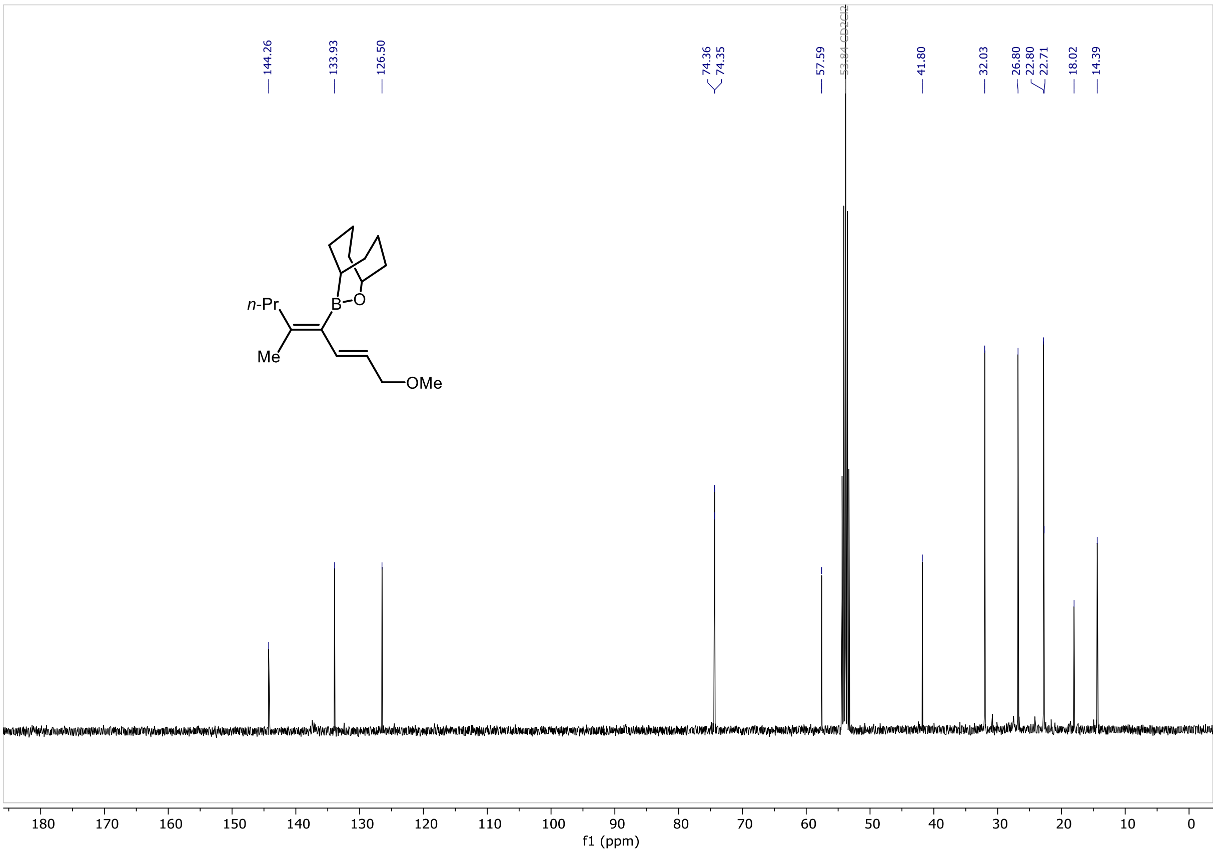

^{1}H NMR (400 MHz, CD_2Cl_2) of **6t**

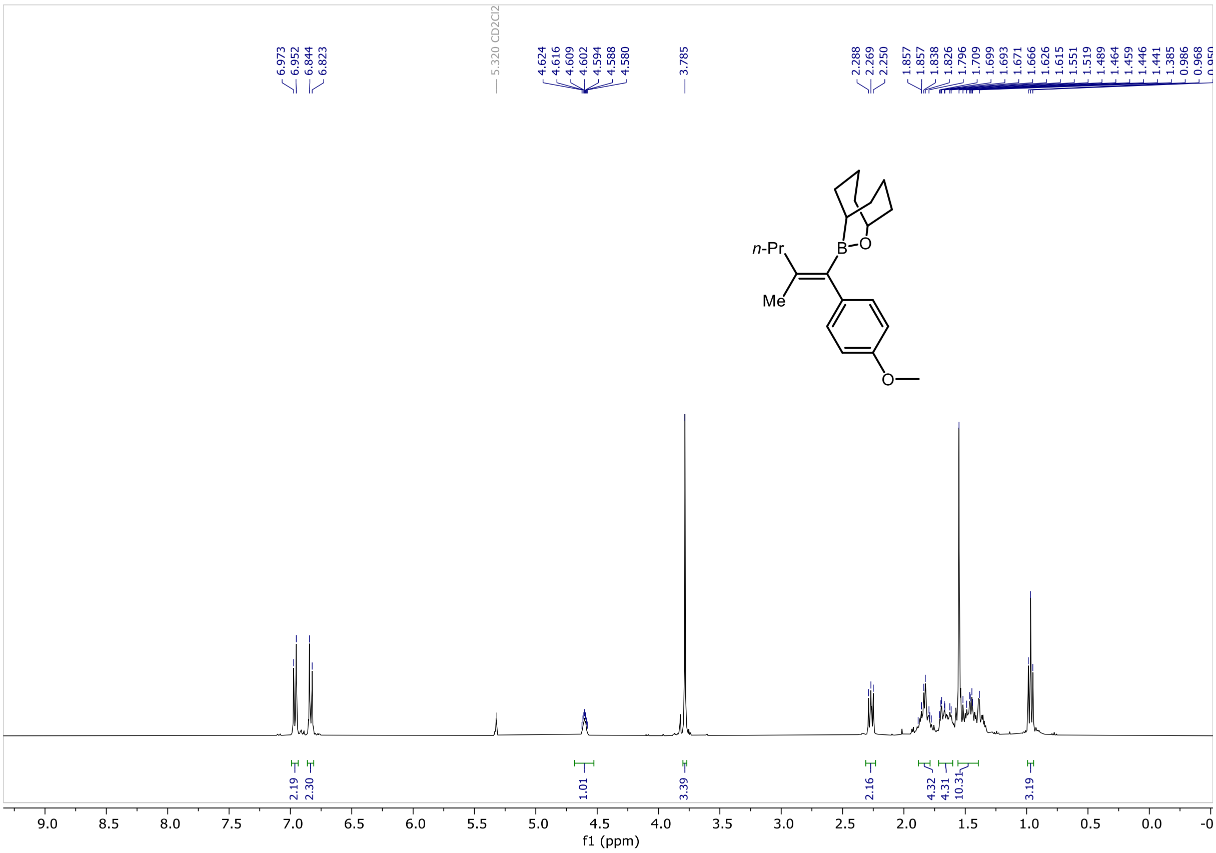

^{13}C NMR (400 MHz, CD_2Cl_2) of **6t**

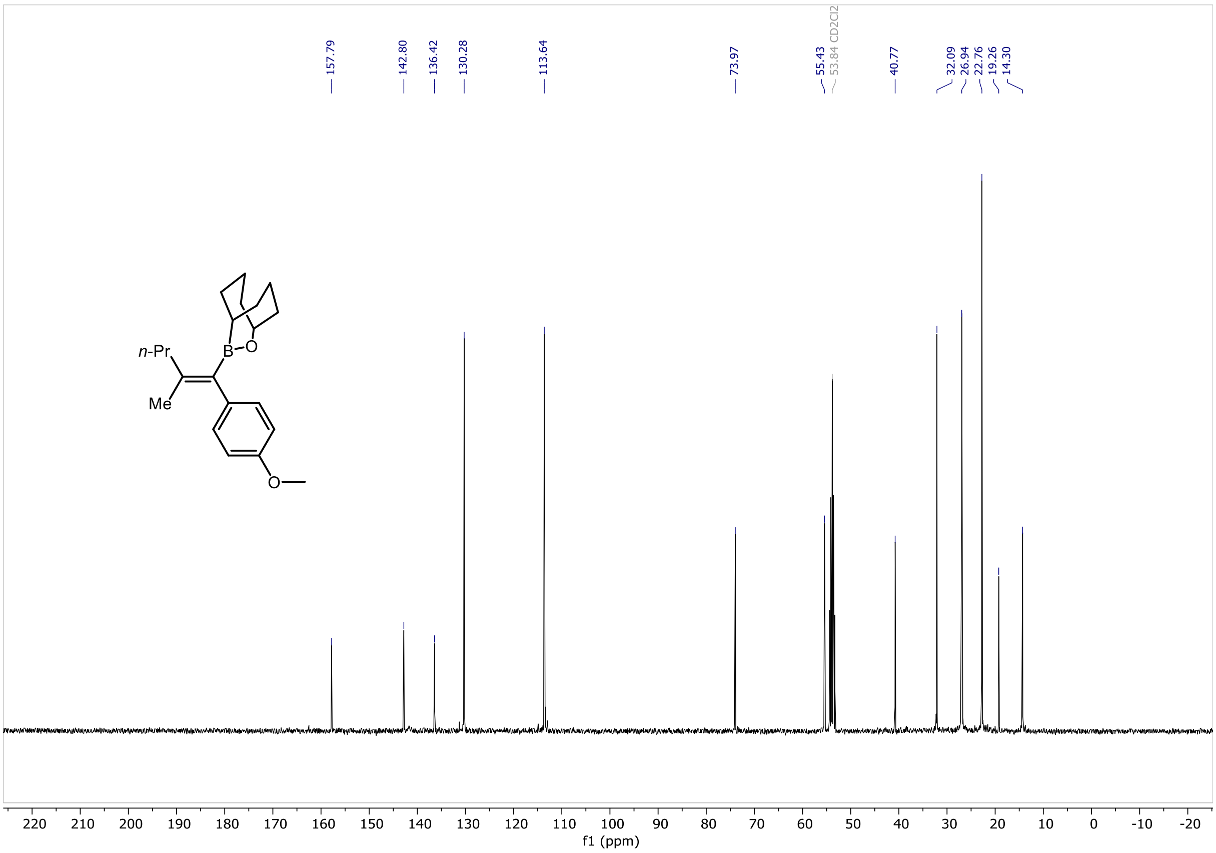

^{1}H NMR (400 MHz, CD_2Cl_2) of **6u**

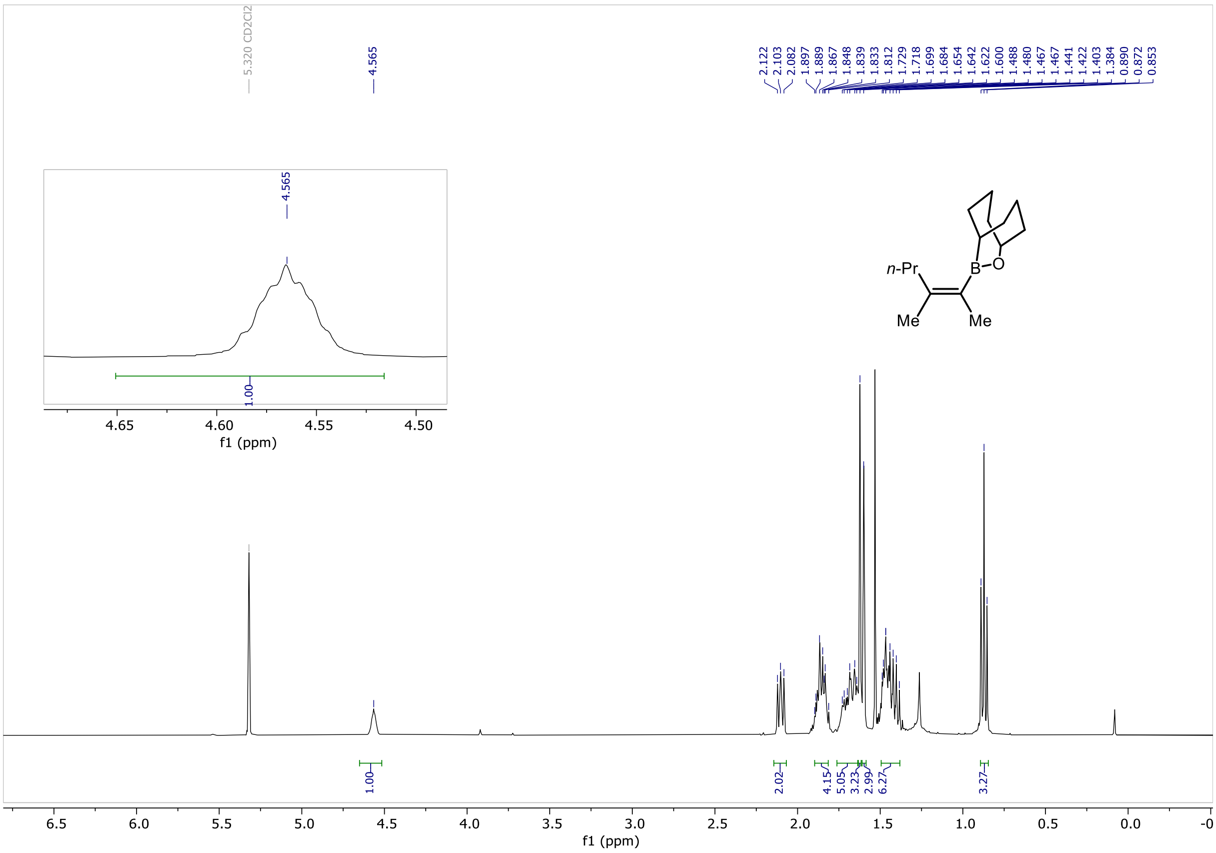

^{13}C NMR (400 MHz, CD_2Cl_2) of **6u**

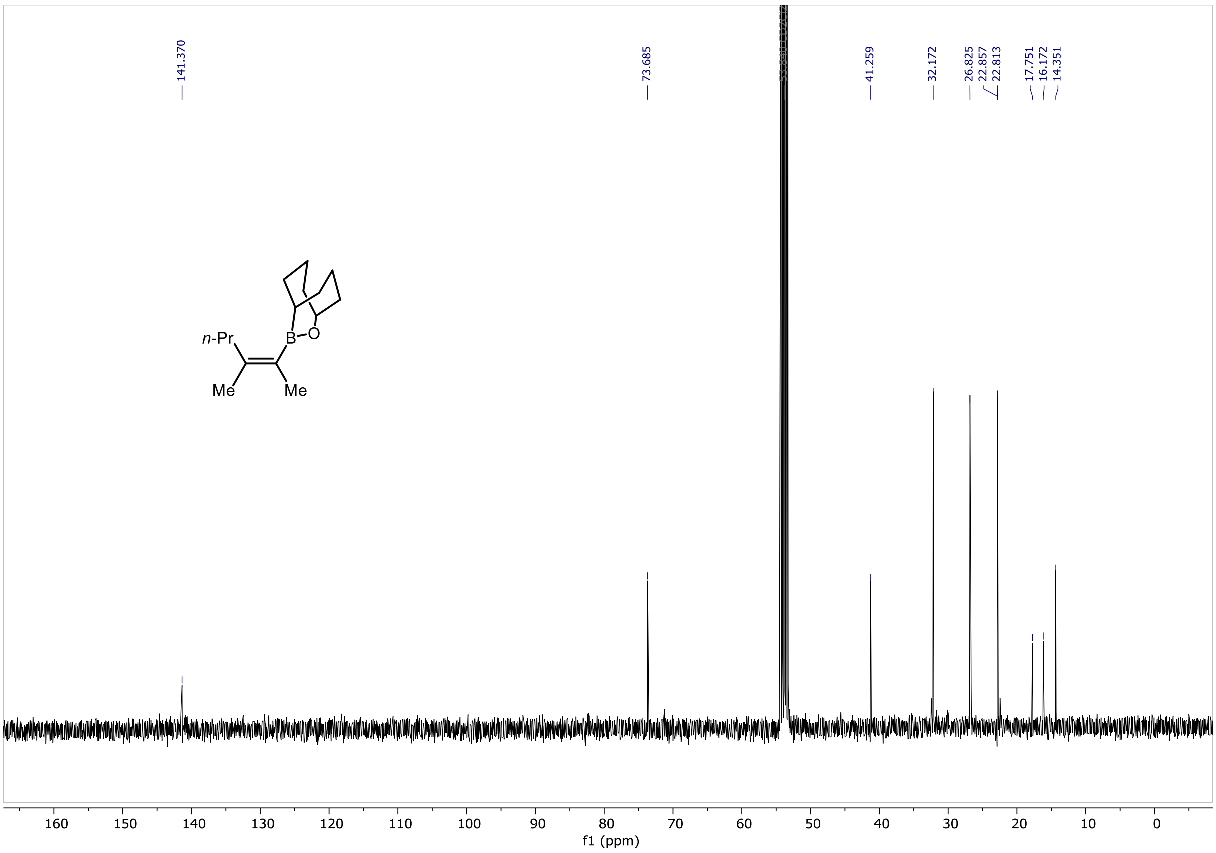

^{1}H NMR (400 MHz, CD_2Cl_2) of **6v**

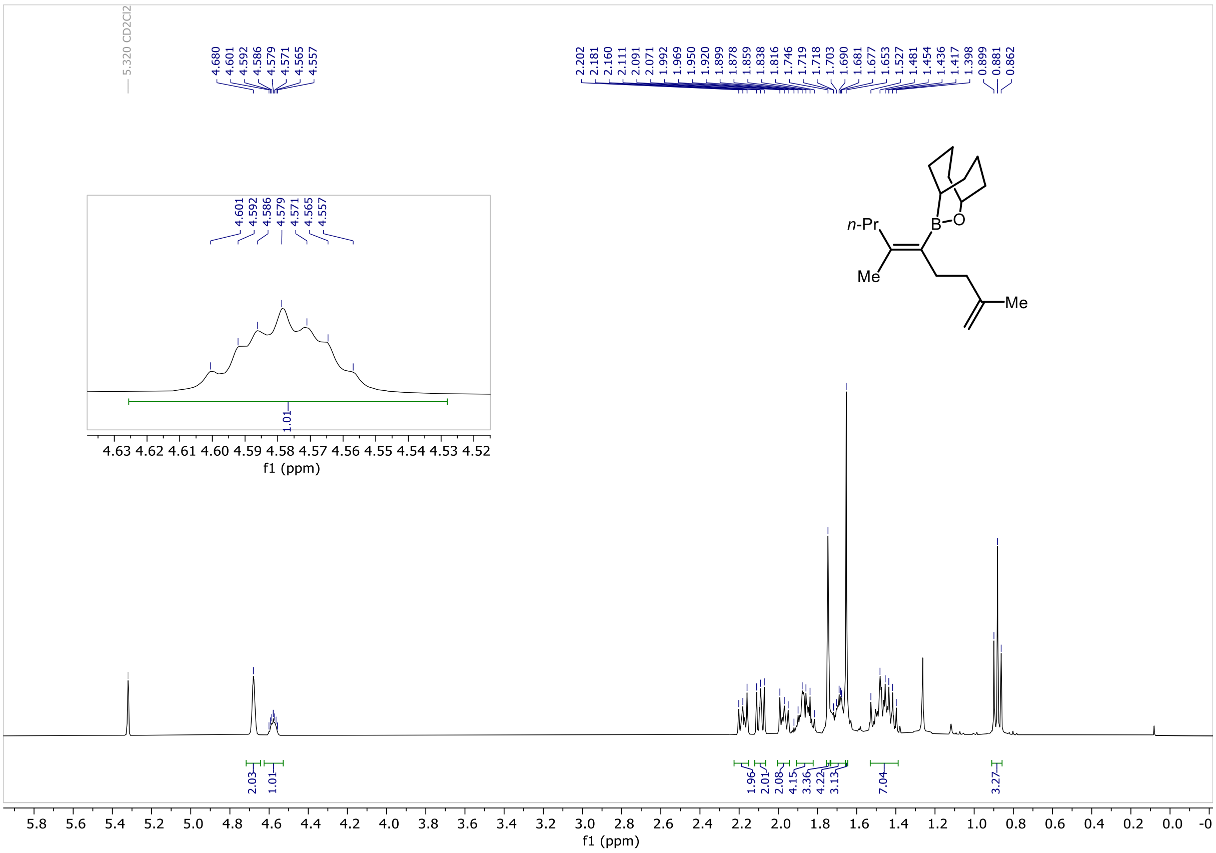

^{13}C NMR (400 MHz, CD_2Cl_2) of **6v**

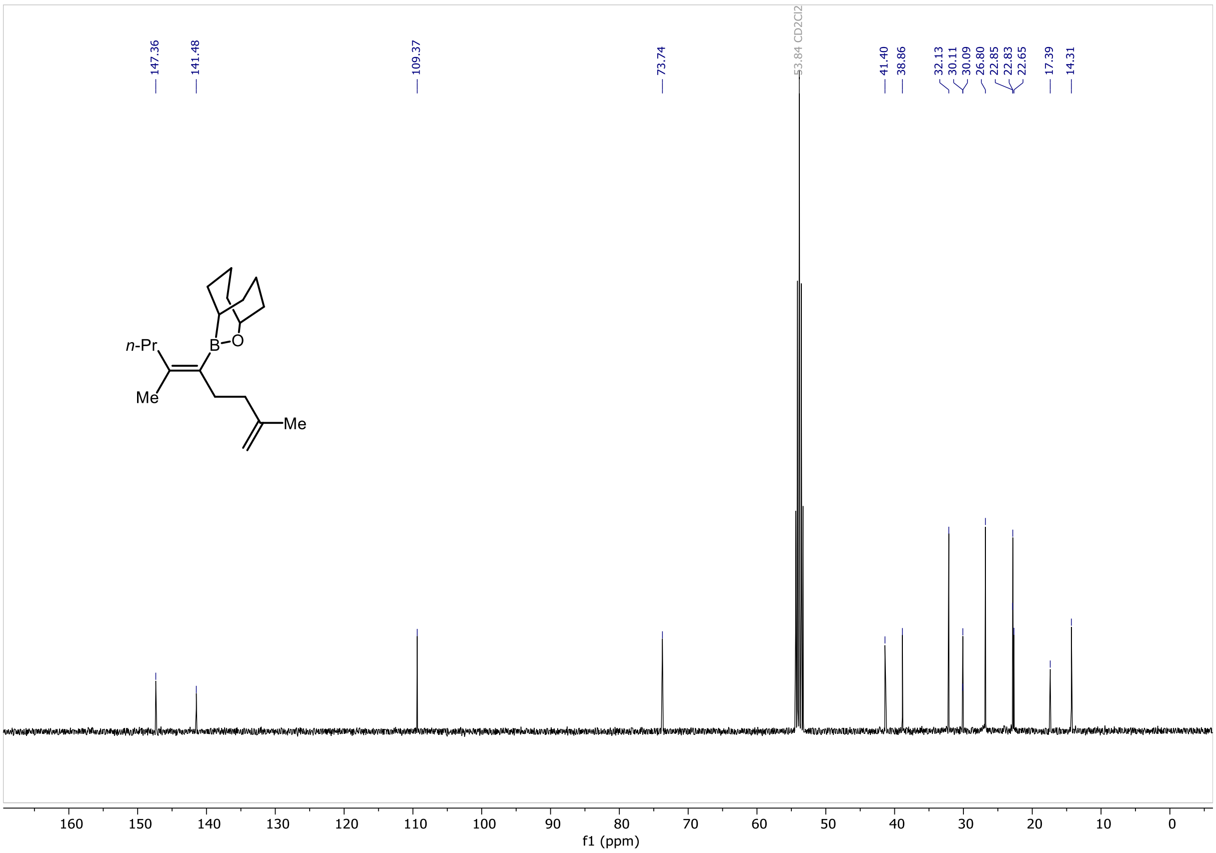

^{1}H NMR (400 MHz, CD_2Cl_2) of **6w**

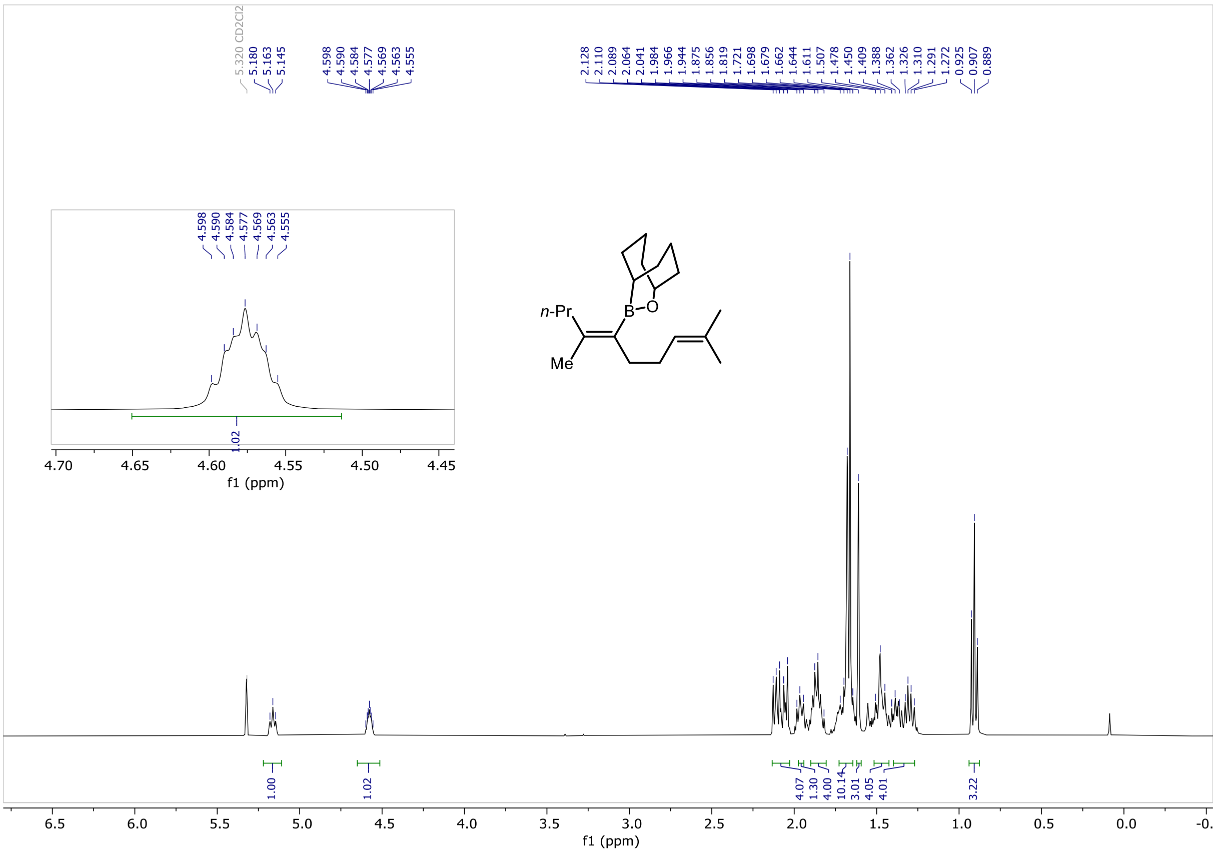

^{13}C NMR (400 MHz, CD_2Cl_2) of **6w**

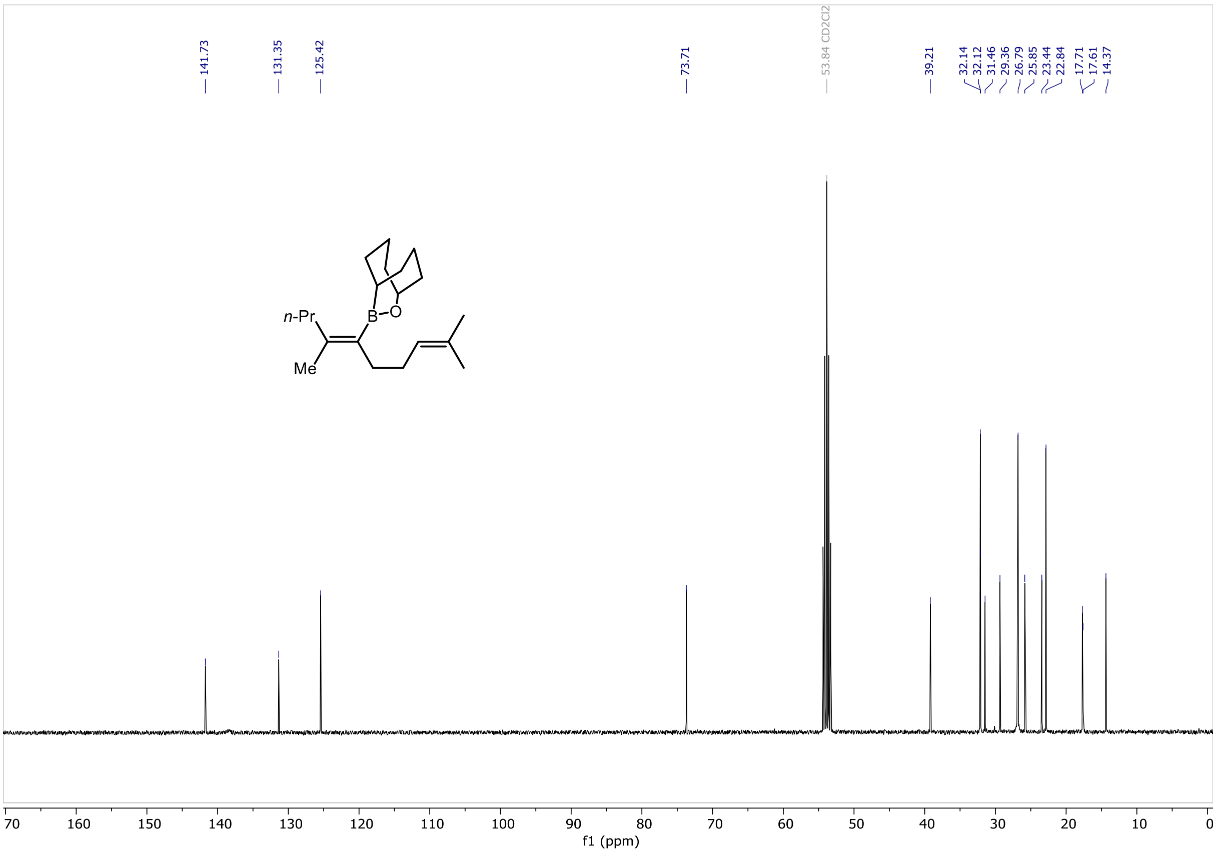

^{1}H NMR (400 MHz, $CDCl_3$) of (*Z*)-**7**

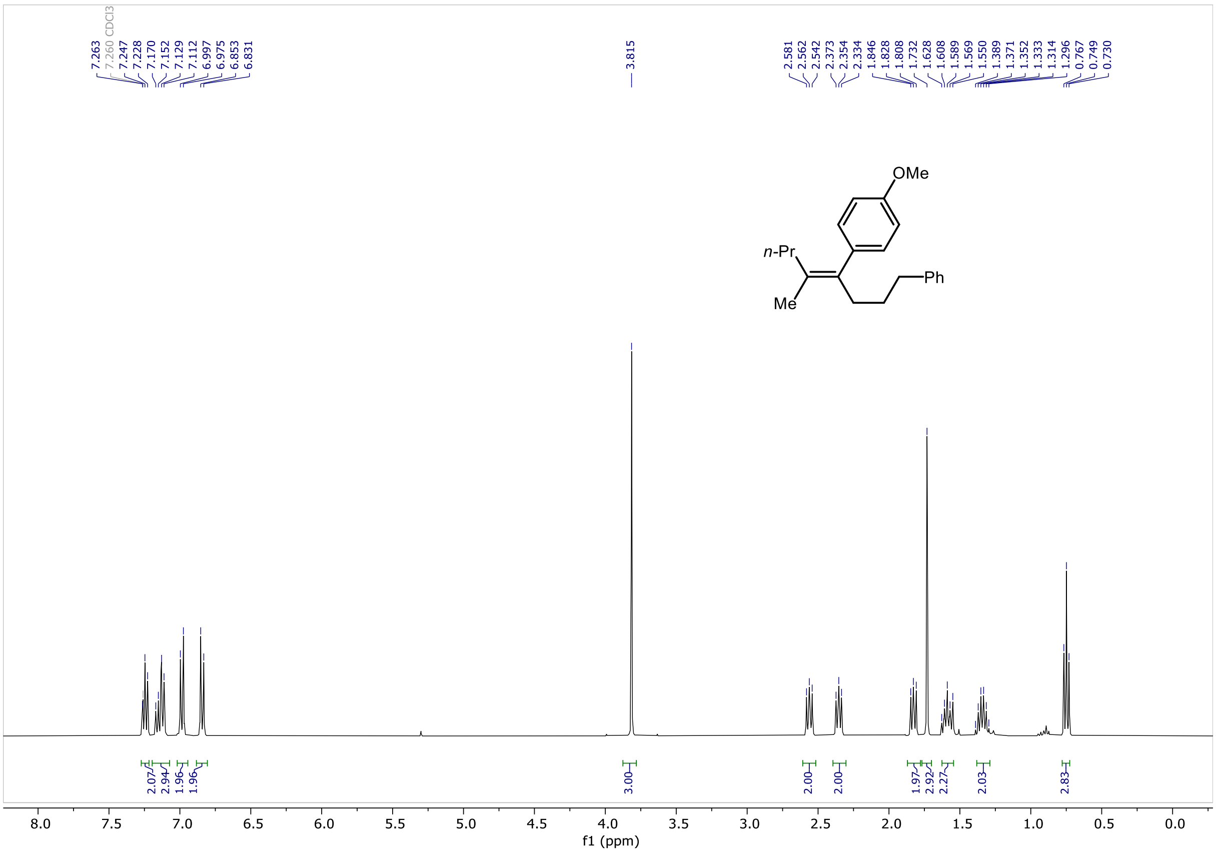

^{13}C NMR (400 MHz, $CDCl_3$) of (*Z*)-**7**

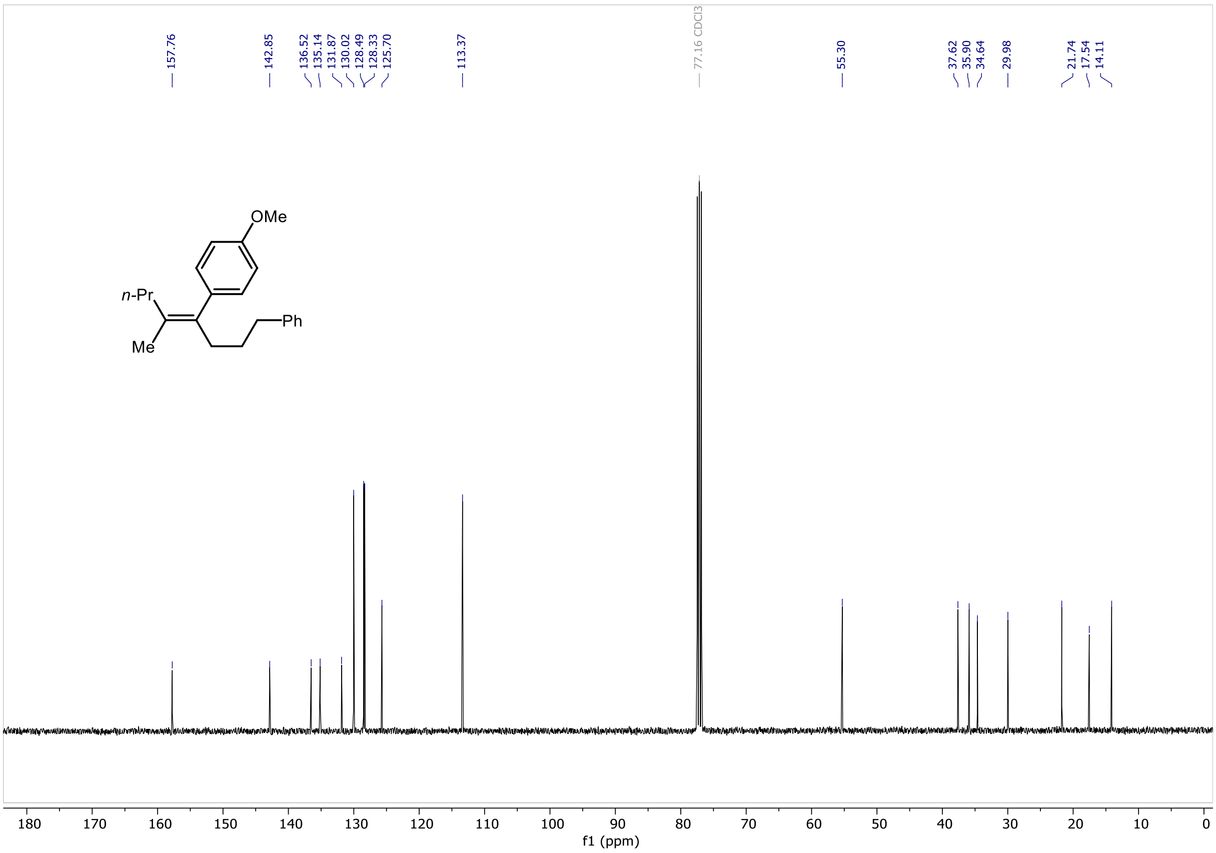

NOESY (500 MHz, $CDCl_3$) of (*Z*)-**7**

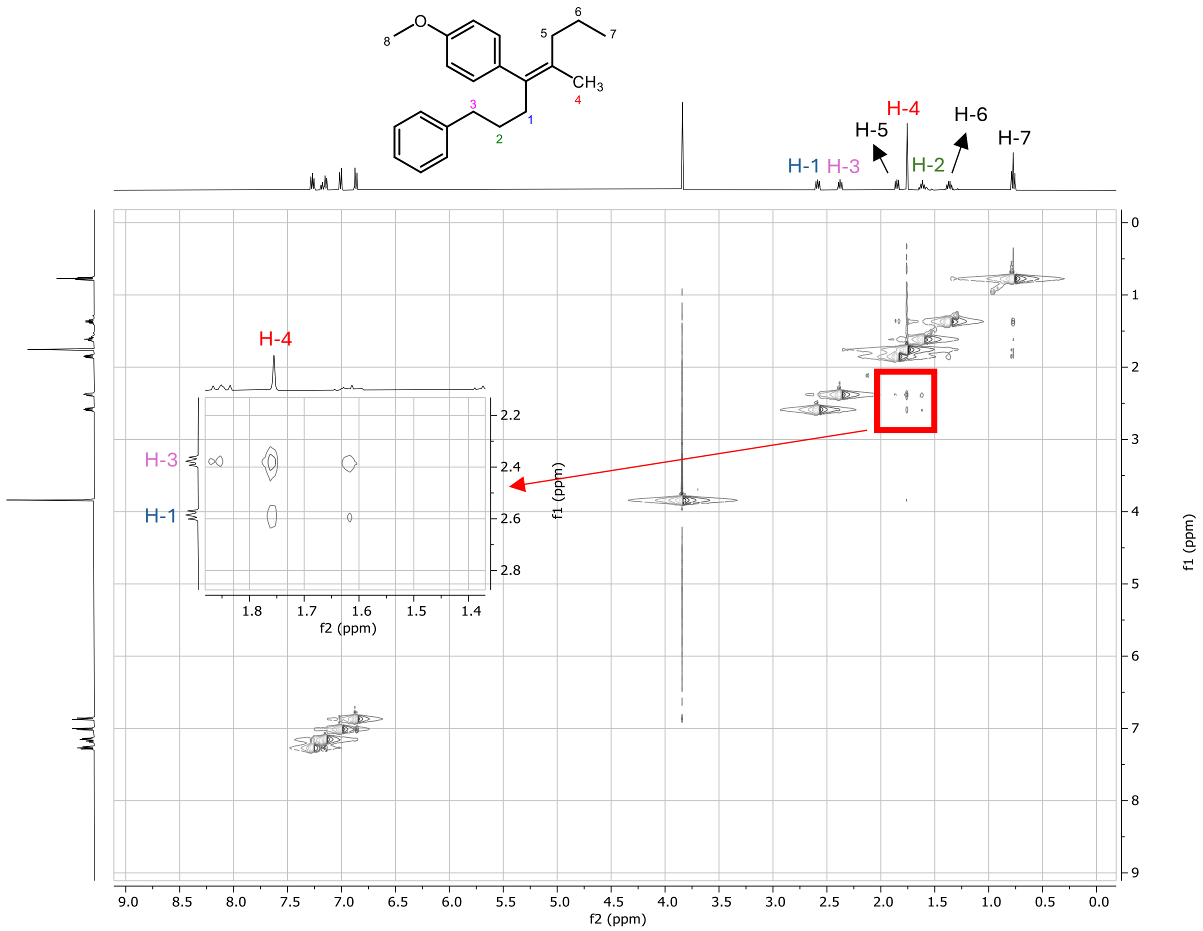

^{1}H NMR (400 MHz, $CDCl_3$) of (*E*)-**8**

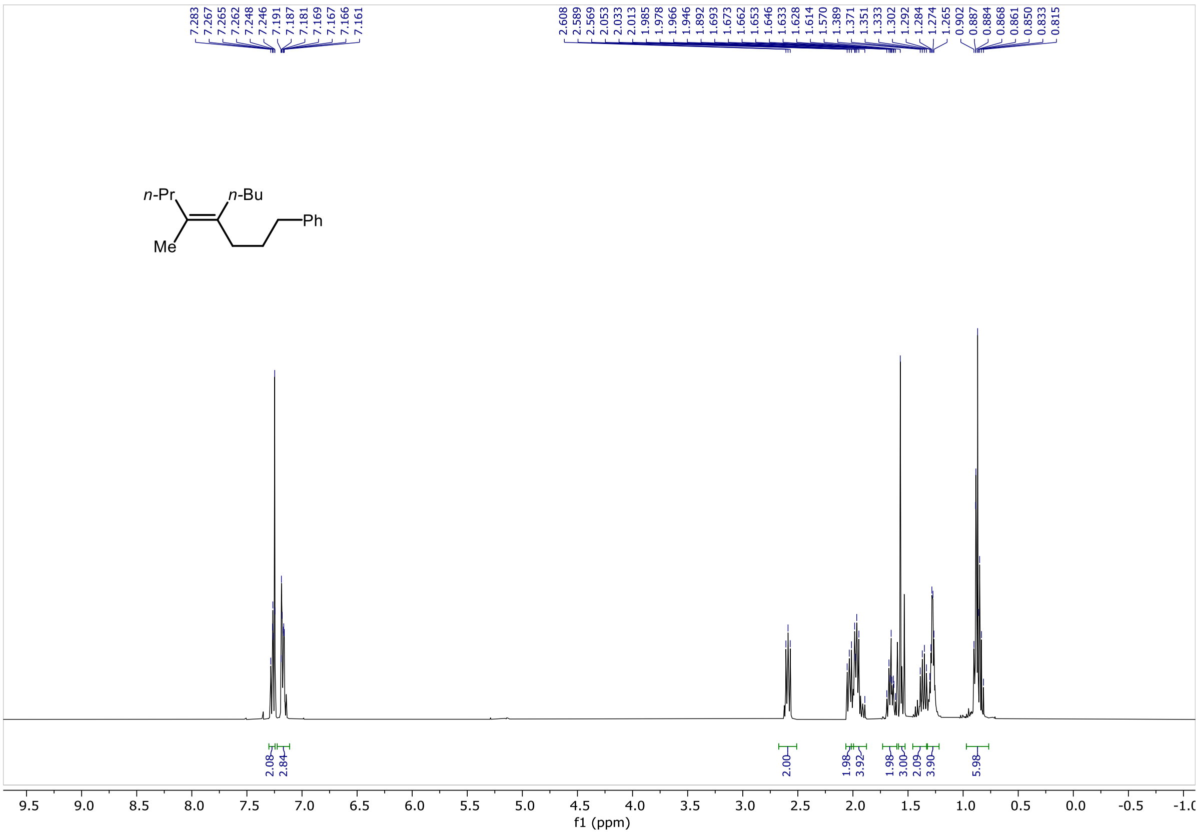

^{13}C NMR (400 MHz, $CDCl_3$) of (*E*)-**8**

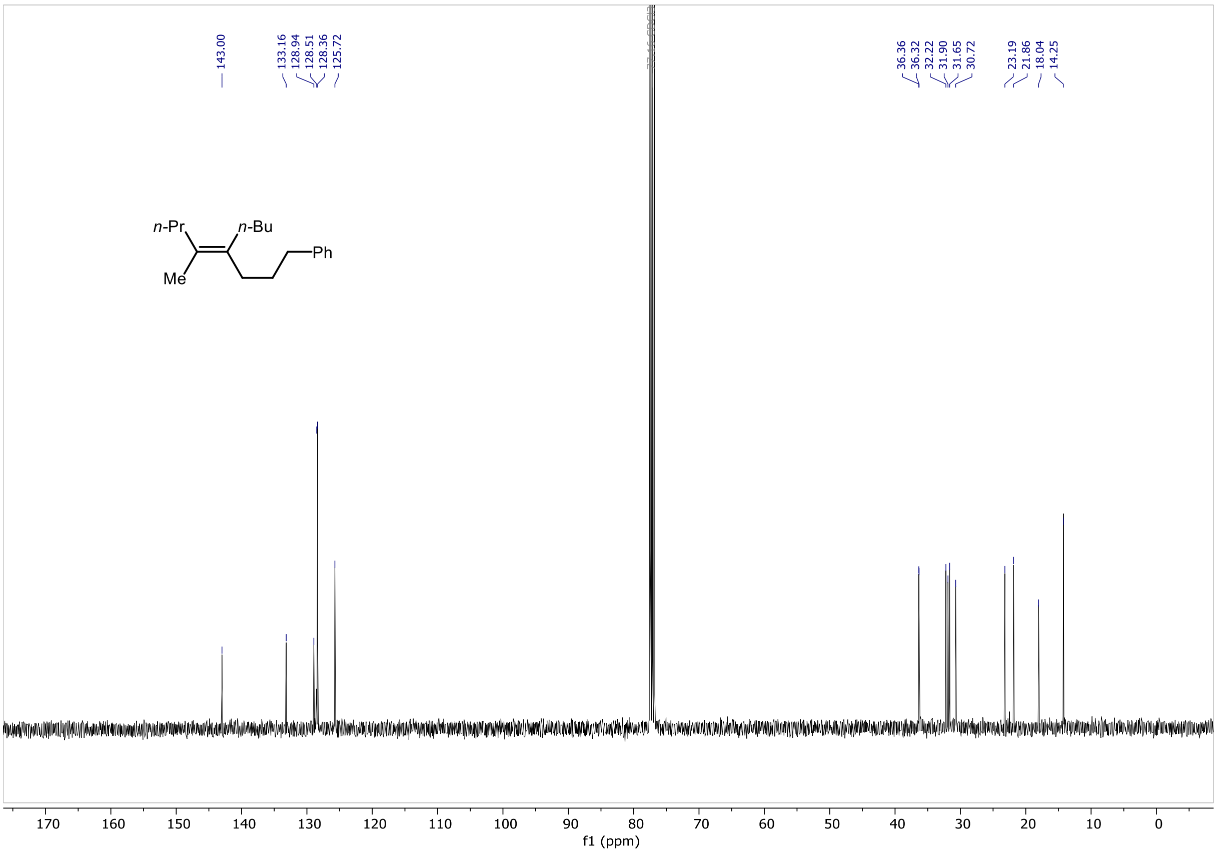

1D selective NOESY (500 MHz, $CDCl_3$) of (*E*)-**8**

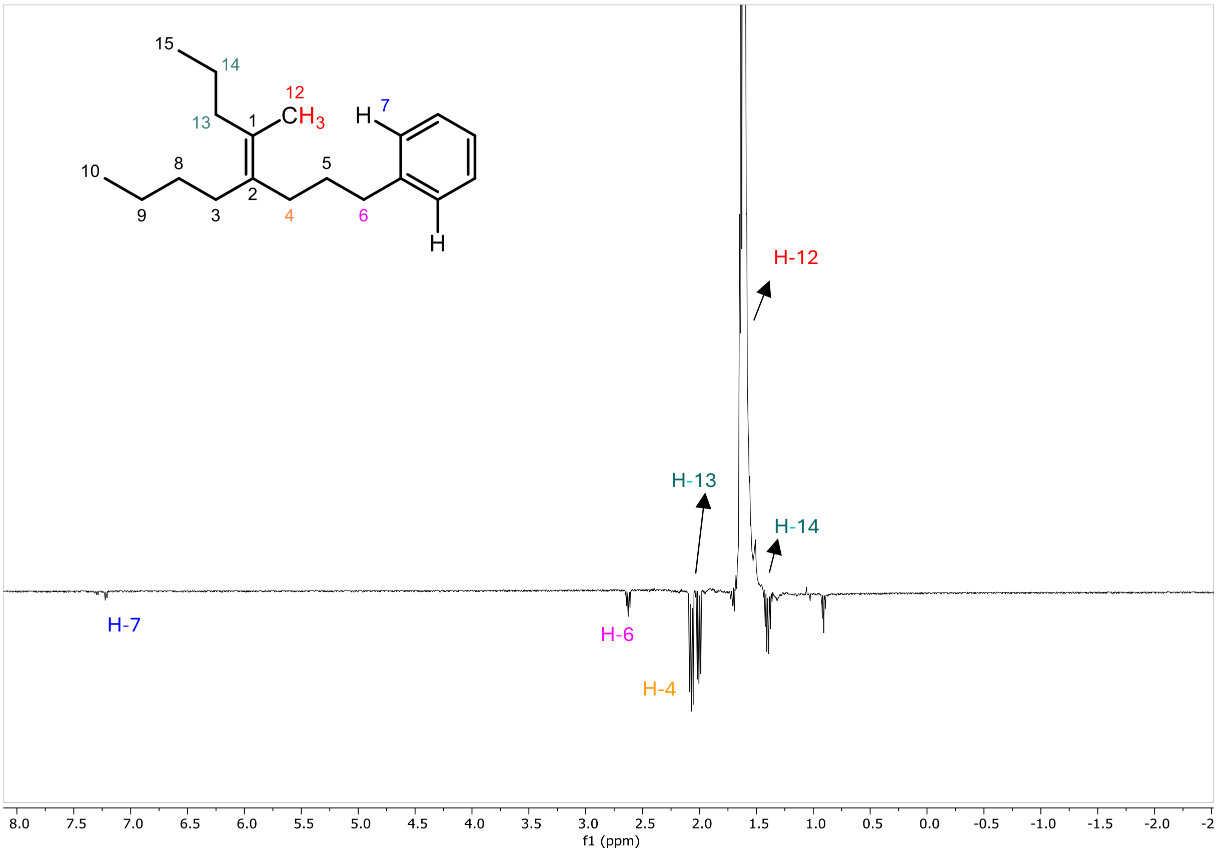

^{1}H NMR (400 MHz, CDCl$_3$) of (*Z*)-**8**

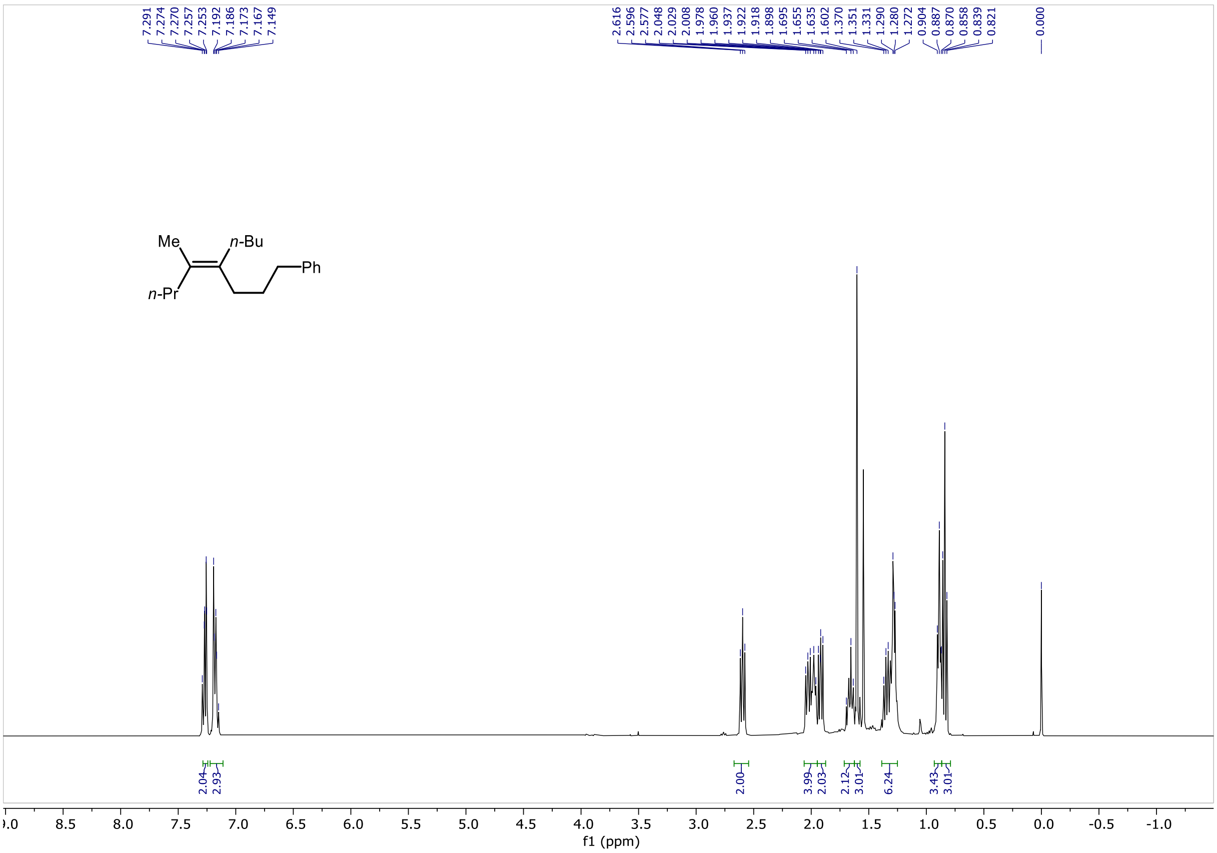

^{13}C NMR (400 MHz, CDCl$_3$) of (*Z*)-**8**

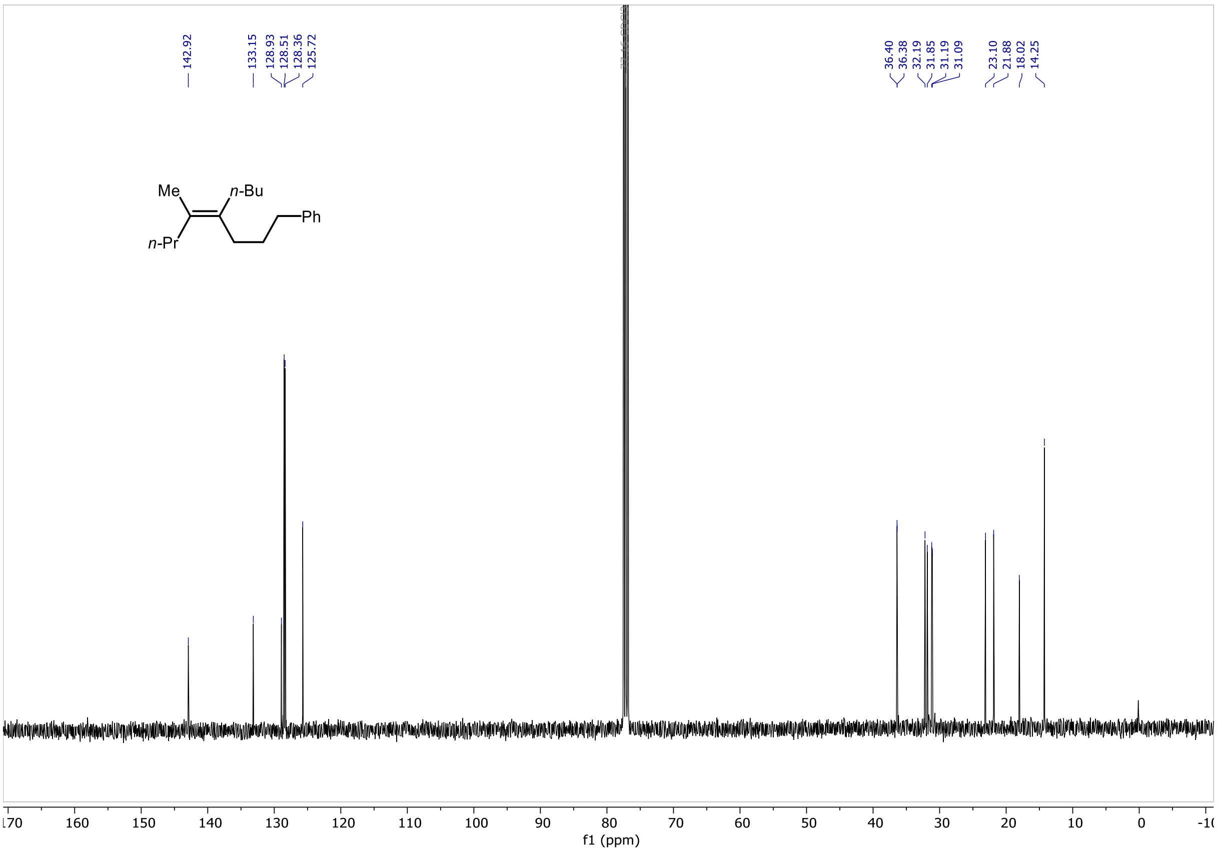

1D selective NOESY (500 MHz, $CDCl_3$) of (*Z*)-**8**

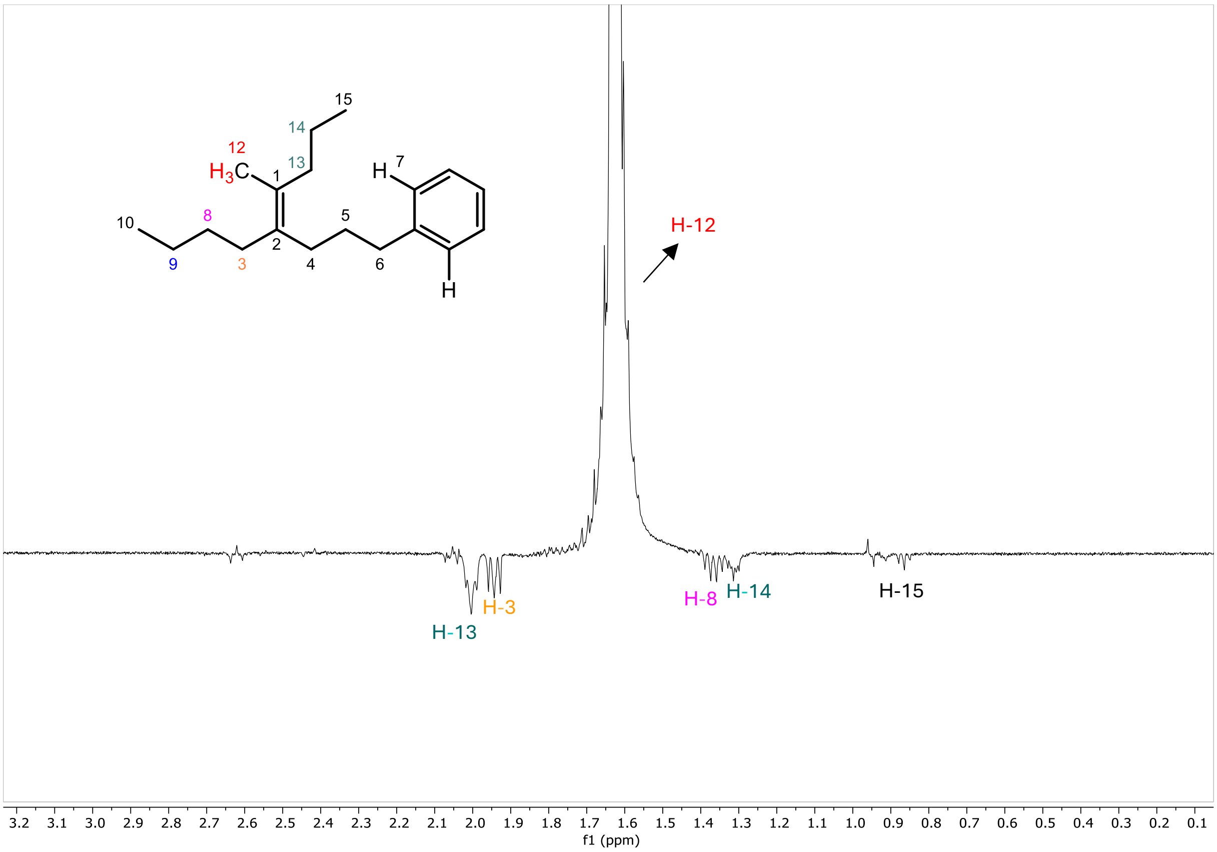

^{1}H NMR (400 MHz, CDCl$_3$) of (*Z*)-**9**

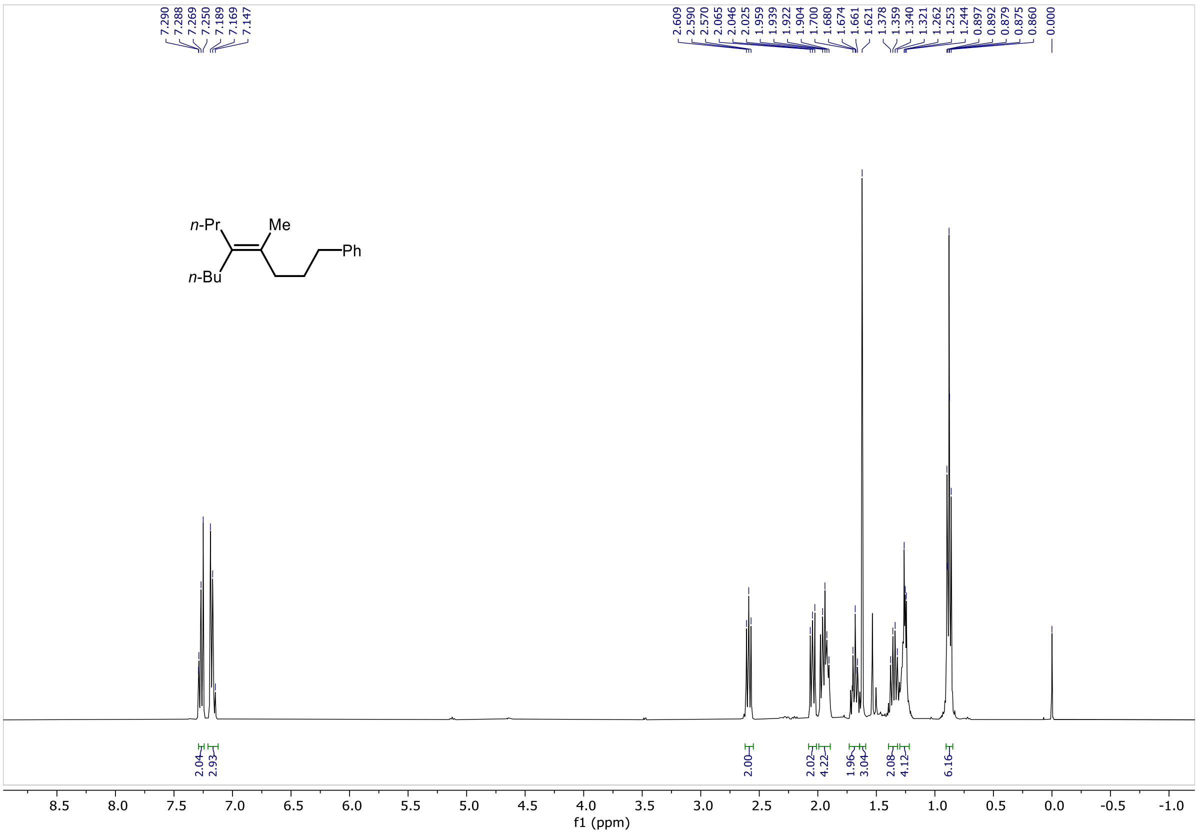

^{13}C NMR (400 MHz, CDCl$_3$) of (*Z*)-**9**

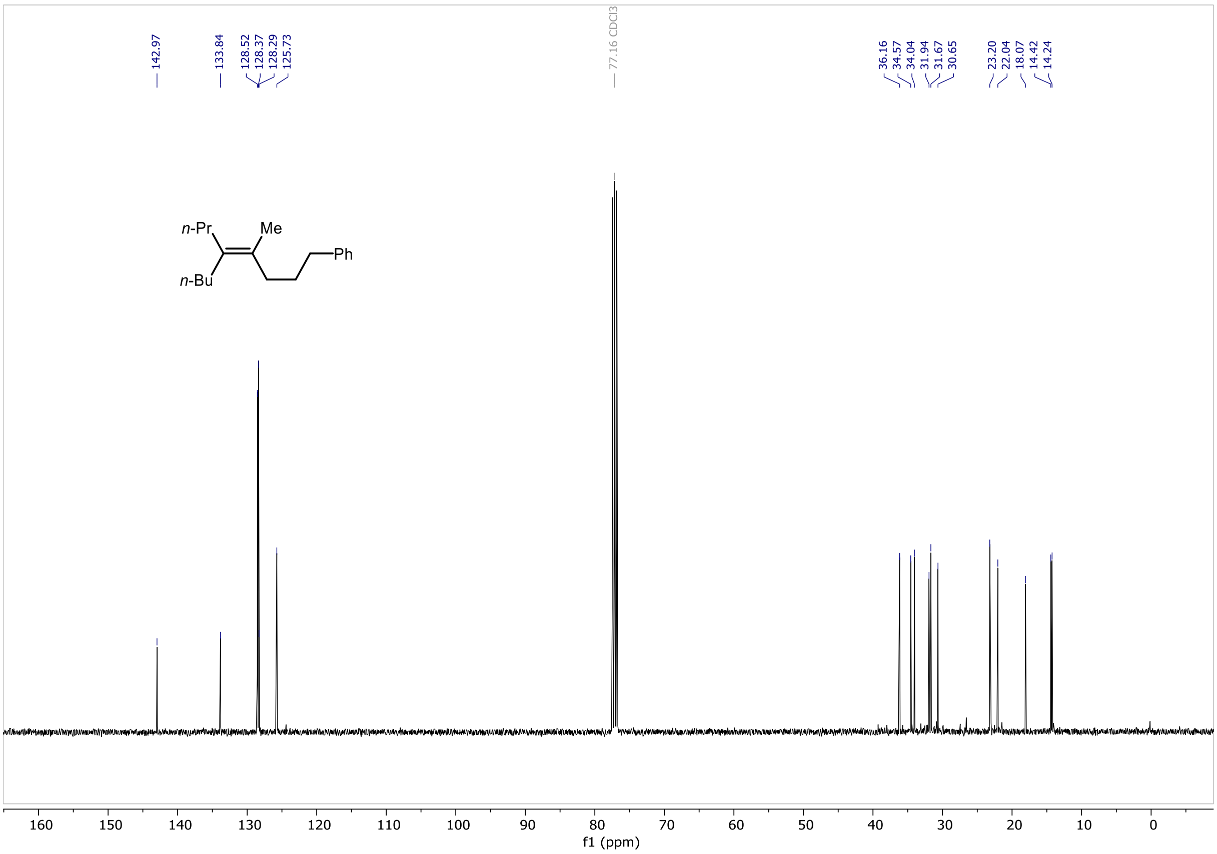

^{1}H NMR (400 MHz, CDCl$_3$) of (*E*)-**9**

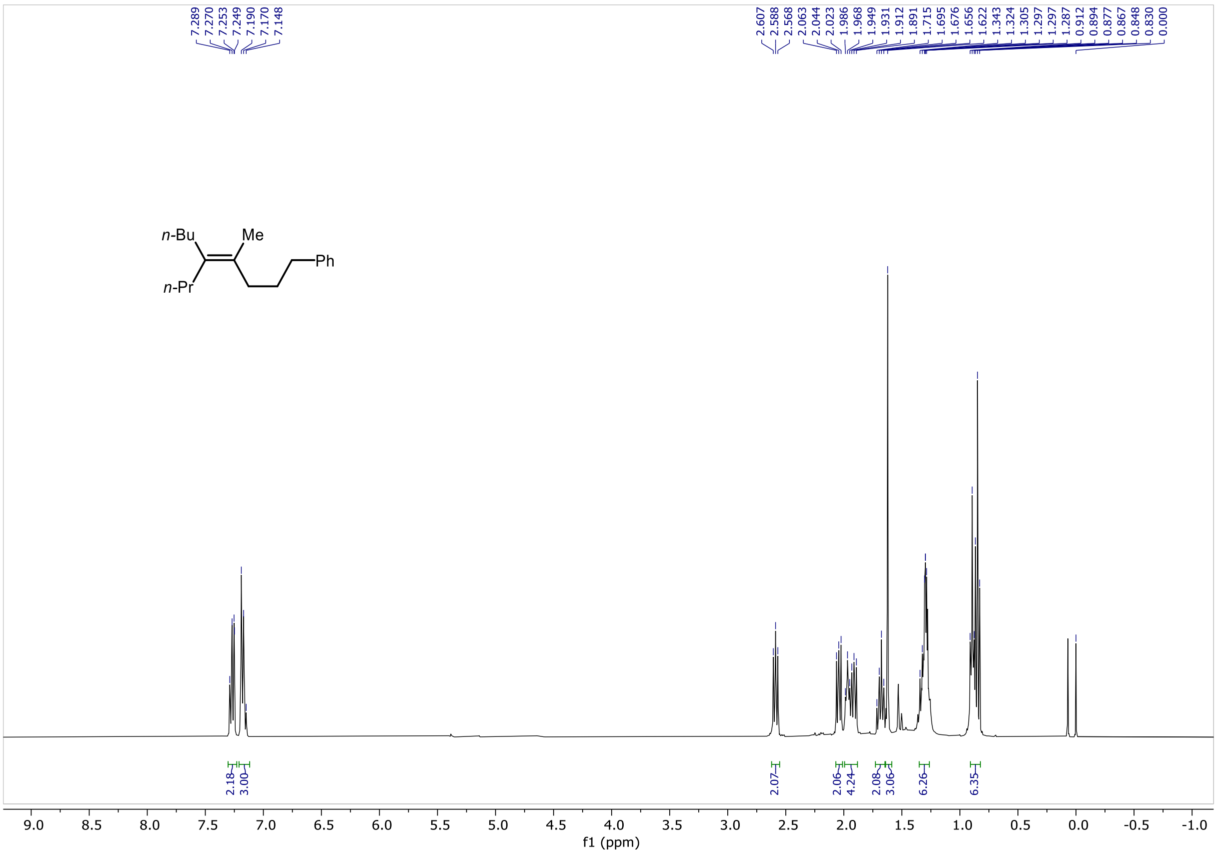

^{13}C NMR (400 MHz, CDCl$_3$) of (*E*)-**9**

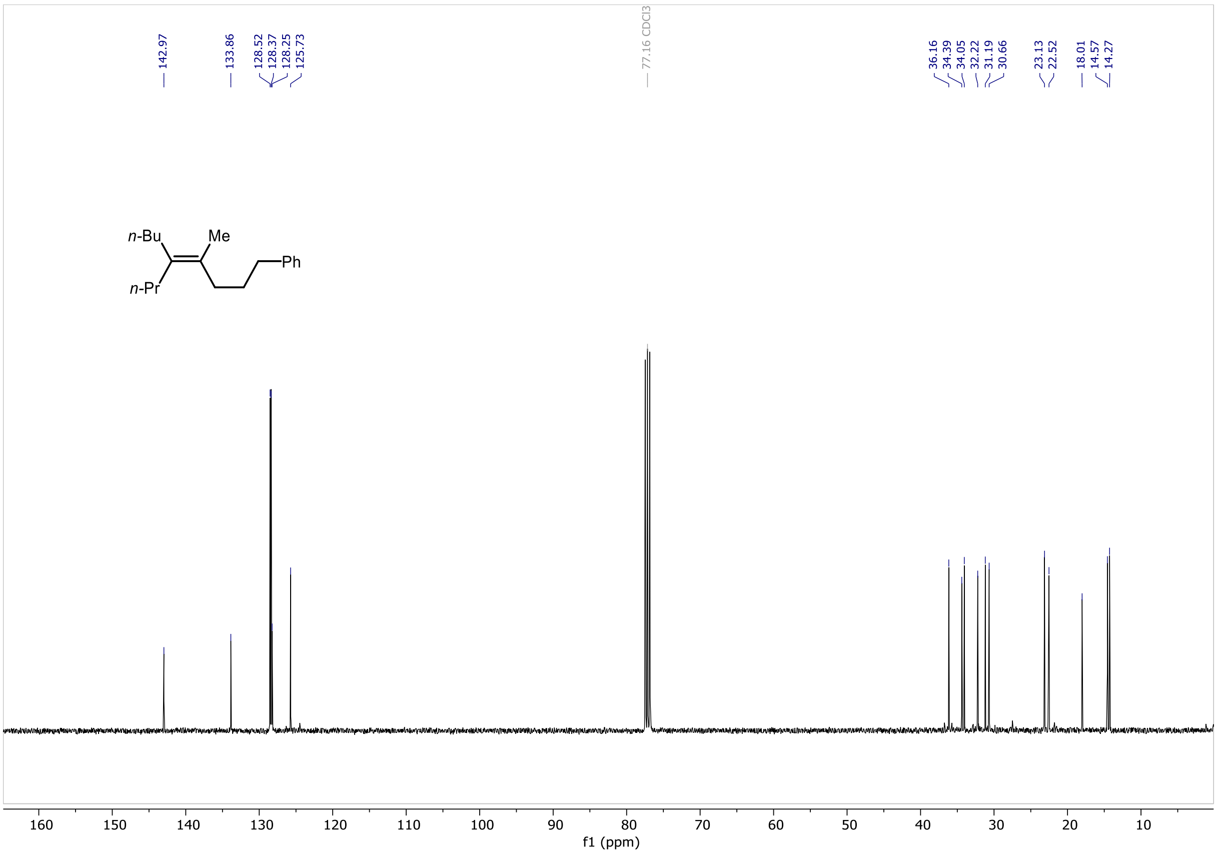

^{1}H NMR (400 MHz, $CDCl_3$) of (*Z*)-**10**

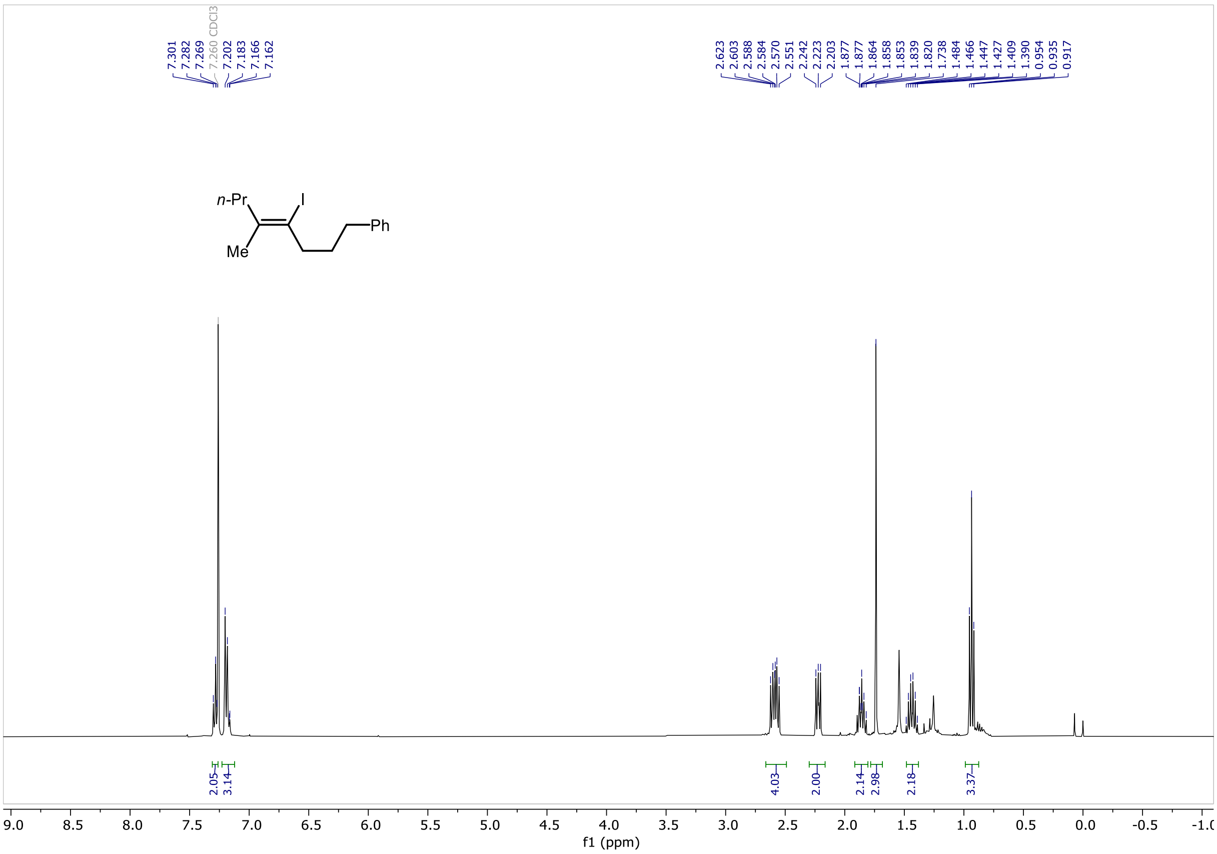

^{13}C NMR (400 MHz, $CDCl_3$) of (*Z*)-**10**

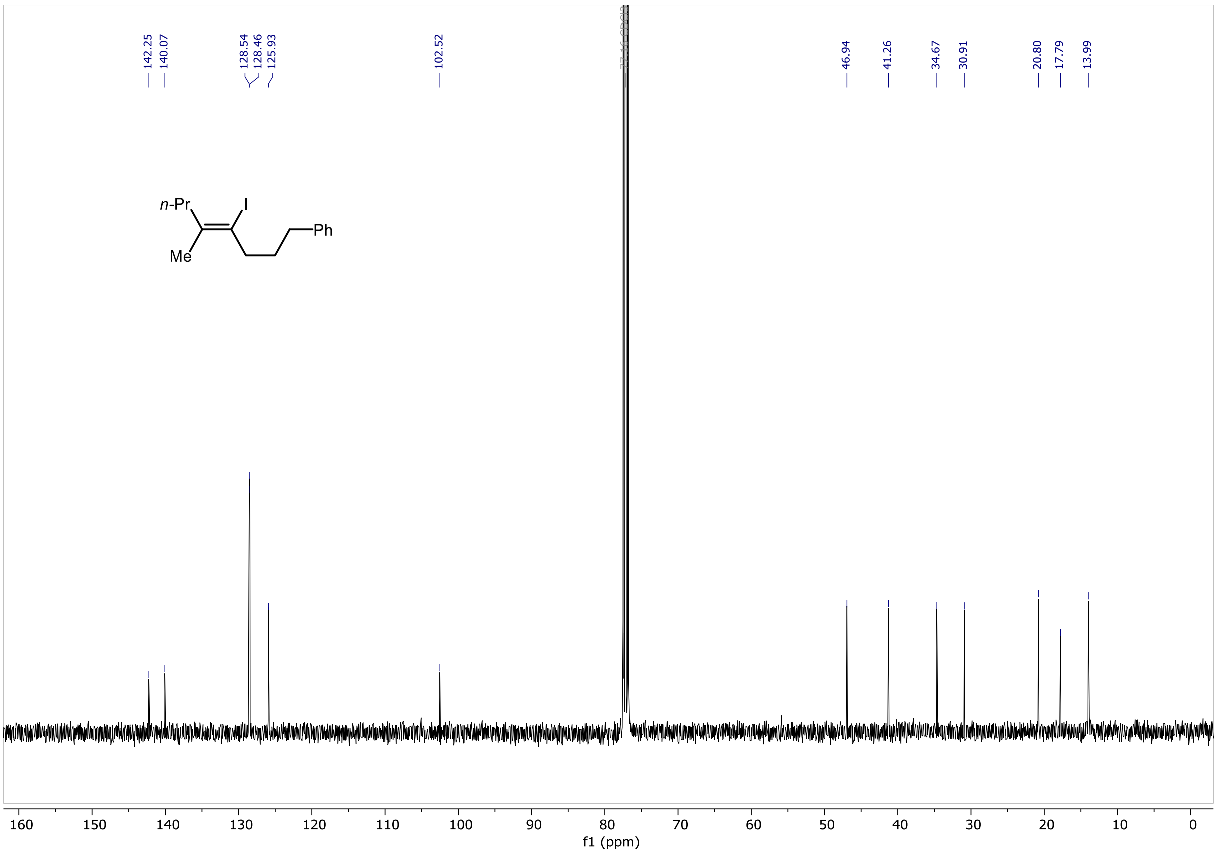

^{1}H NMR (400 MHz, $CDCl_3$) of (*Z*)-**11**

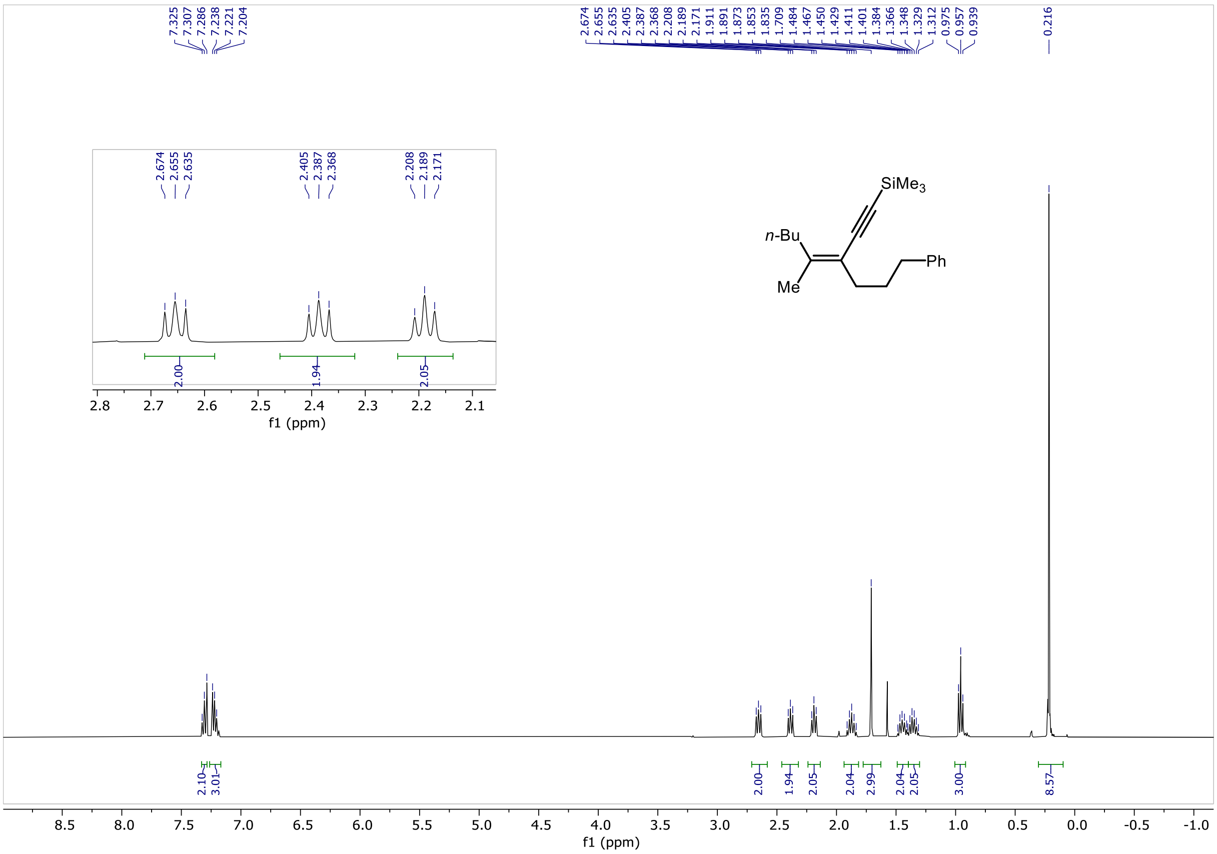

^{13}C NMR (400 MHz, $CDCl_3$) of (*Z*)-**11**

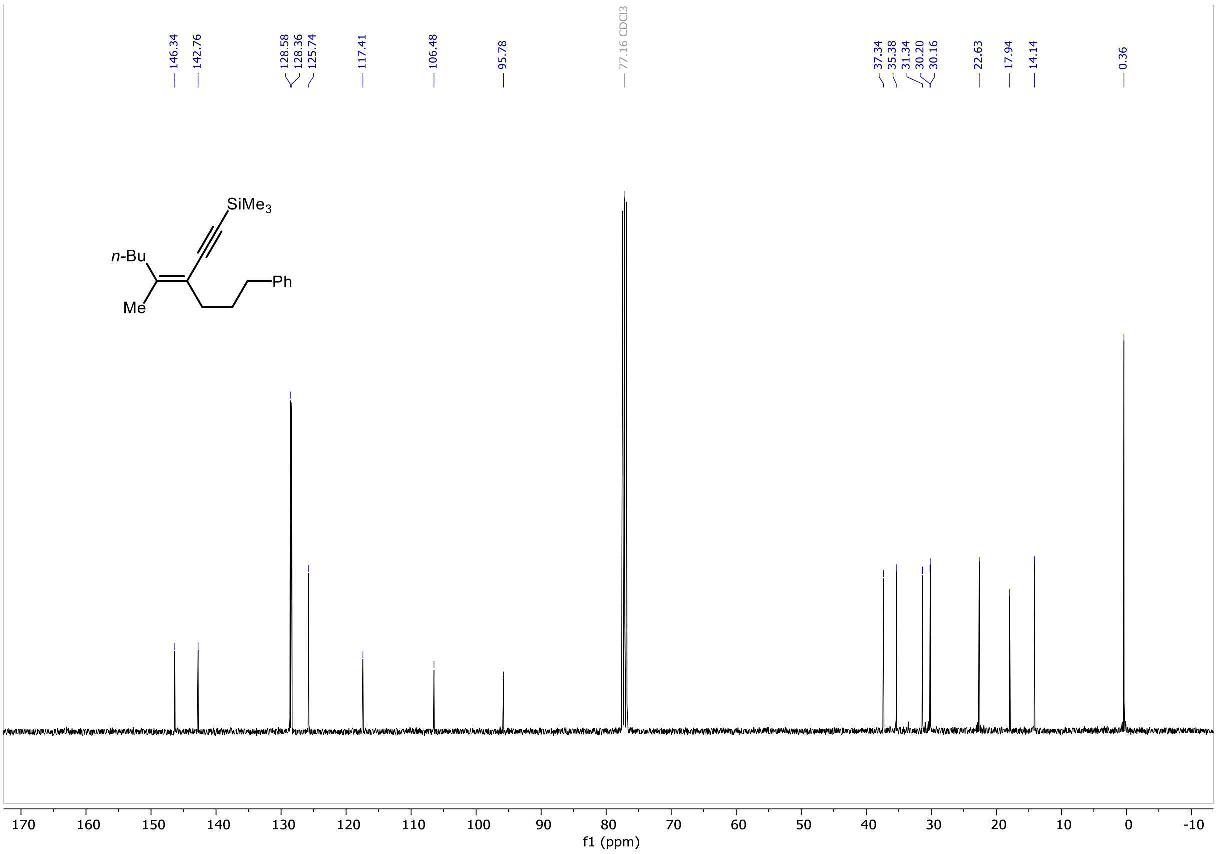

^{1}H NMR (400 MHz, $CDCl_3$) of (*Z*)-**12**

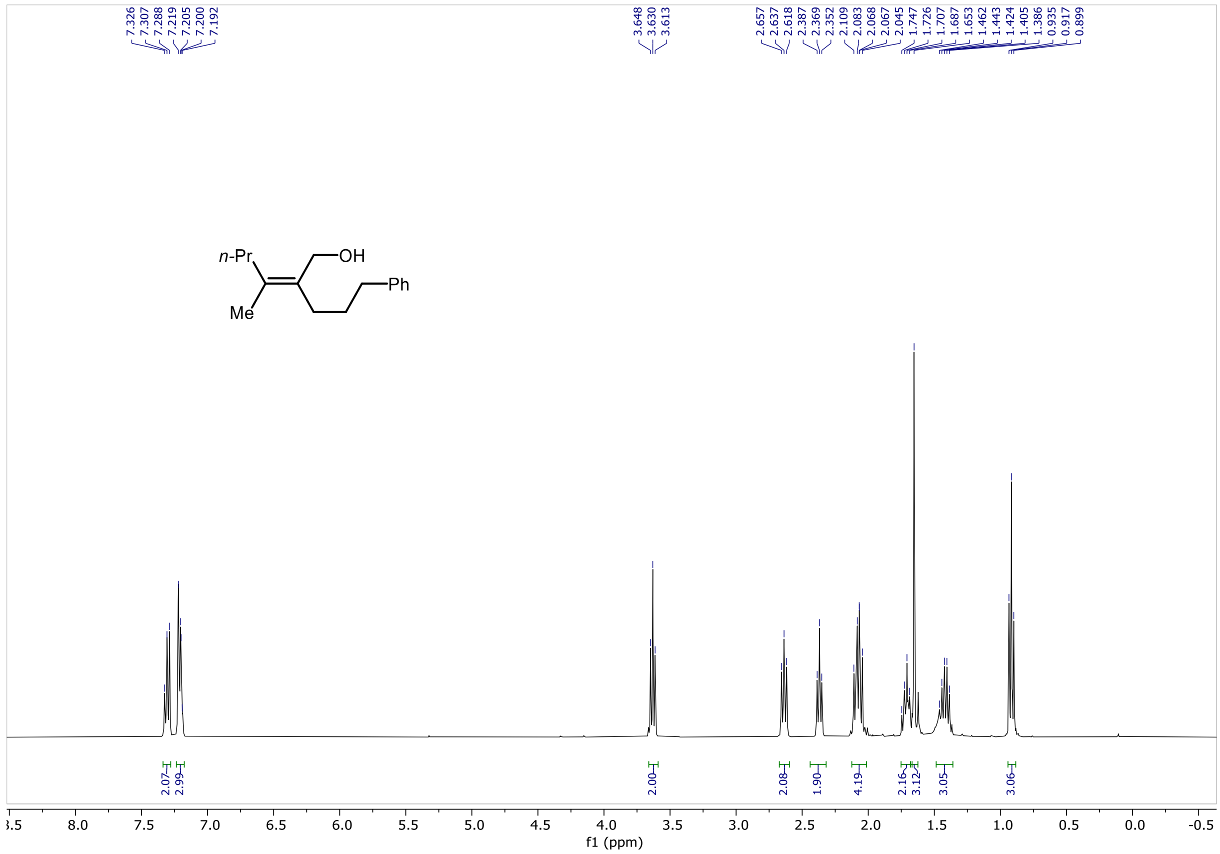

^{13}C NMR (400 MHz, $CDCl_3$) of (*Z*)-**12**

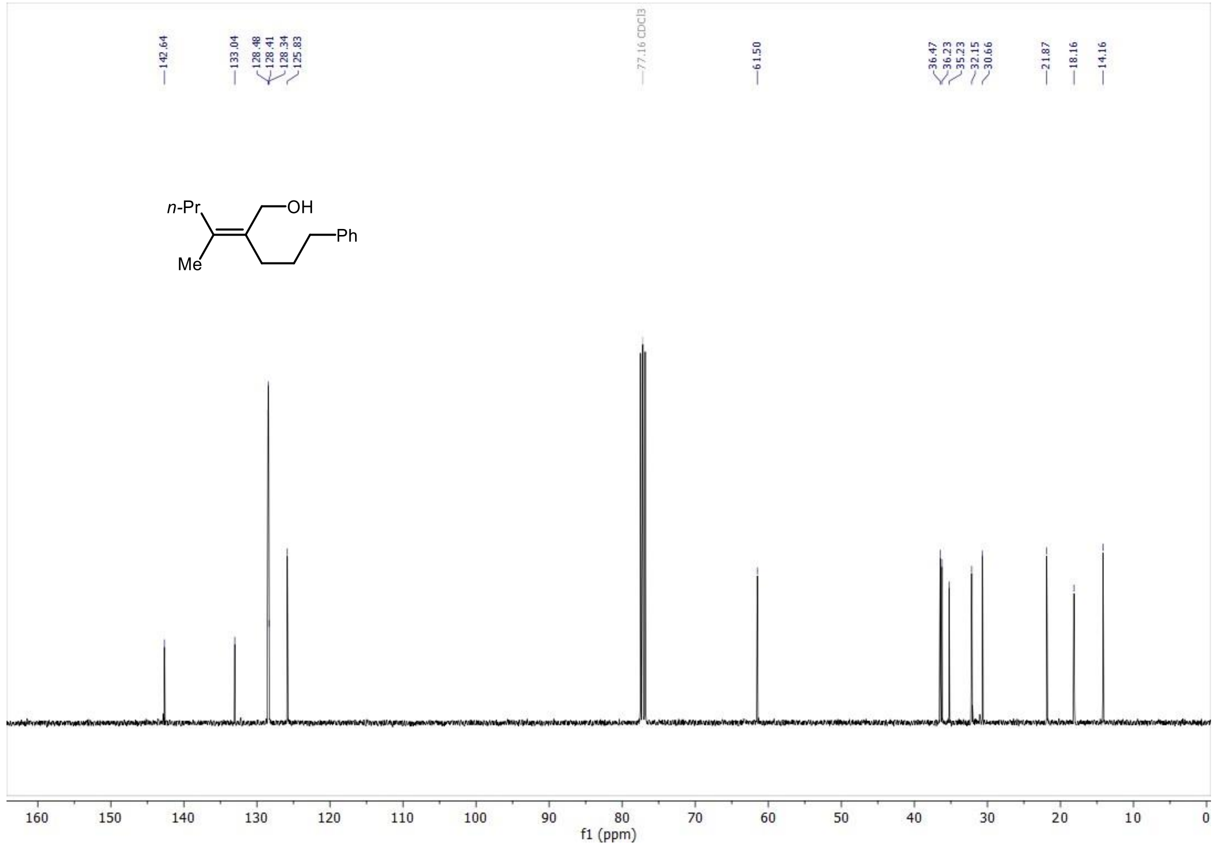

^{1}H NMR (400 MHz, $CDCl_3$) of (*Z*)-**13**

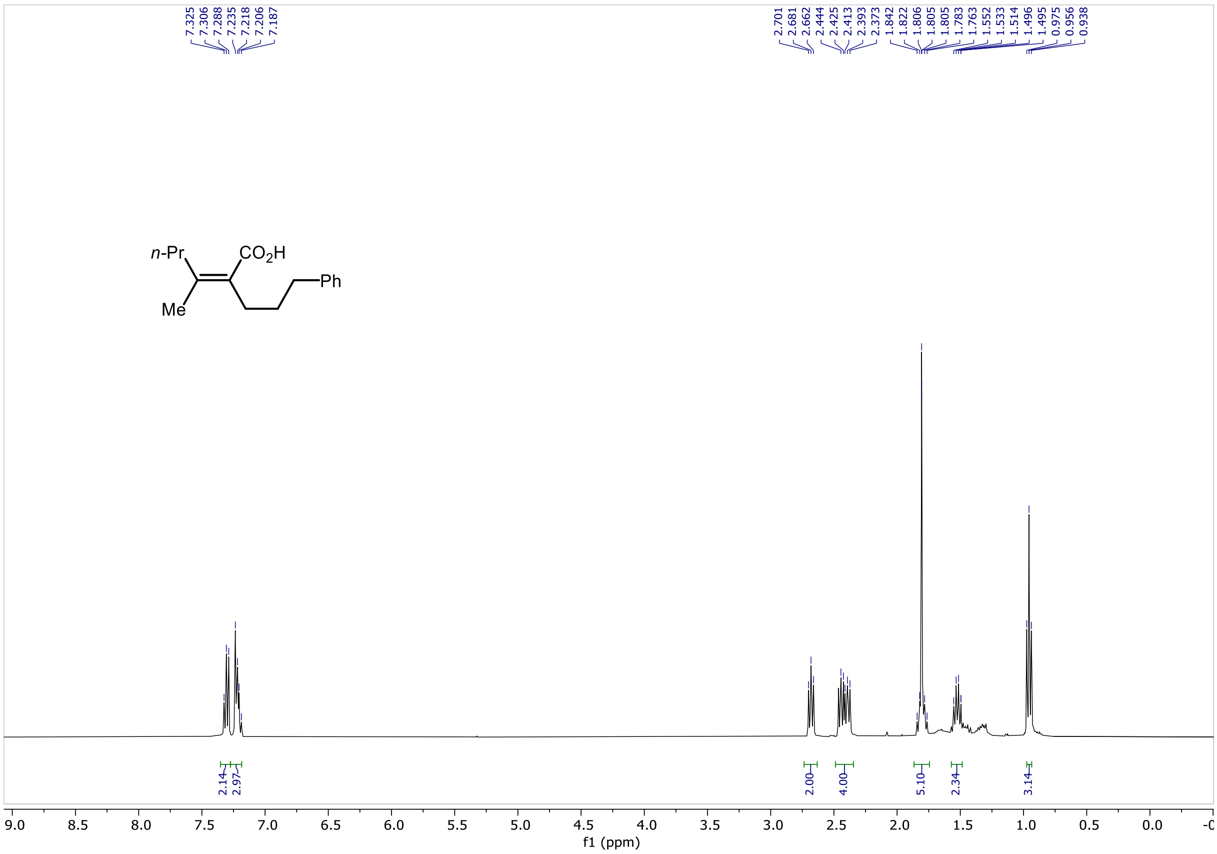

^{13}C NMR (400 MHz, $CDCl_3$) of (*Z*)-**13**

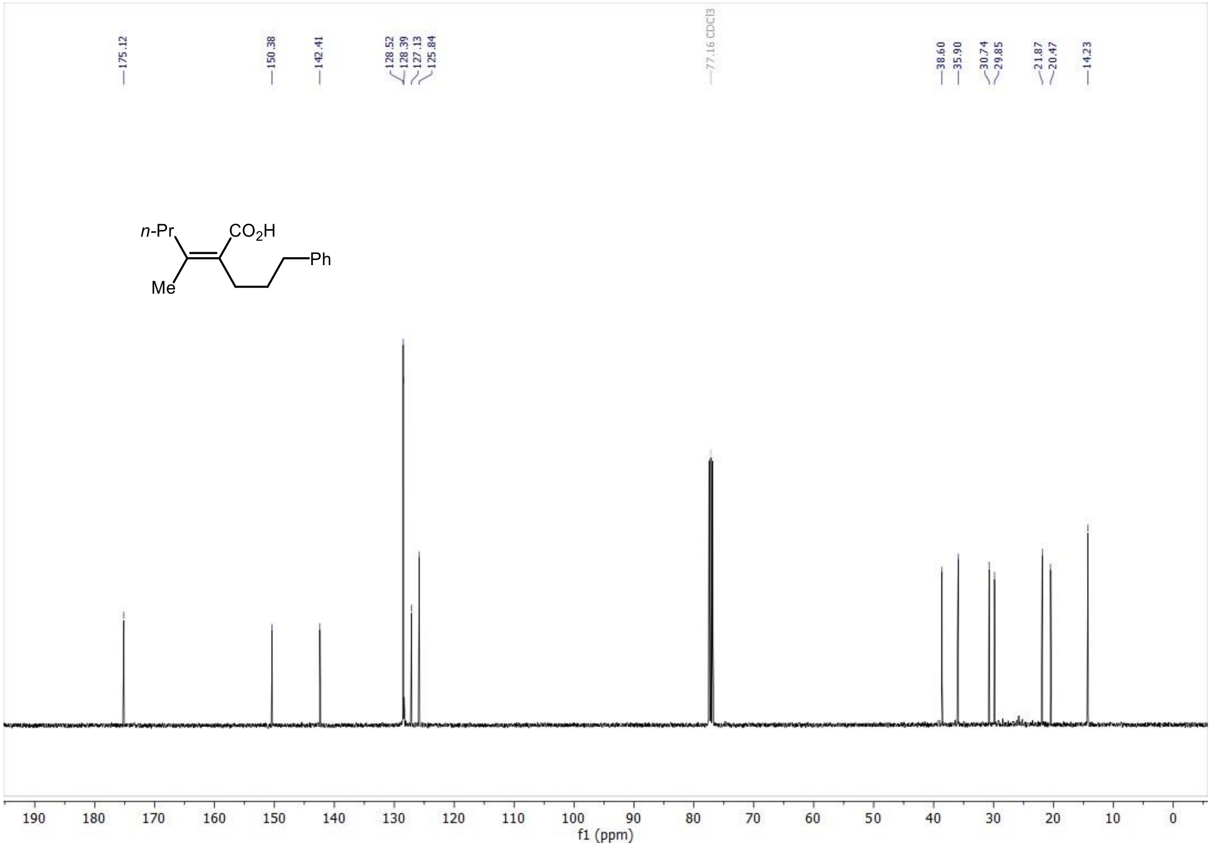

^{1}H NMR (400 MHz, $CDCl_3$) of (*Z*)-**14**

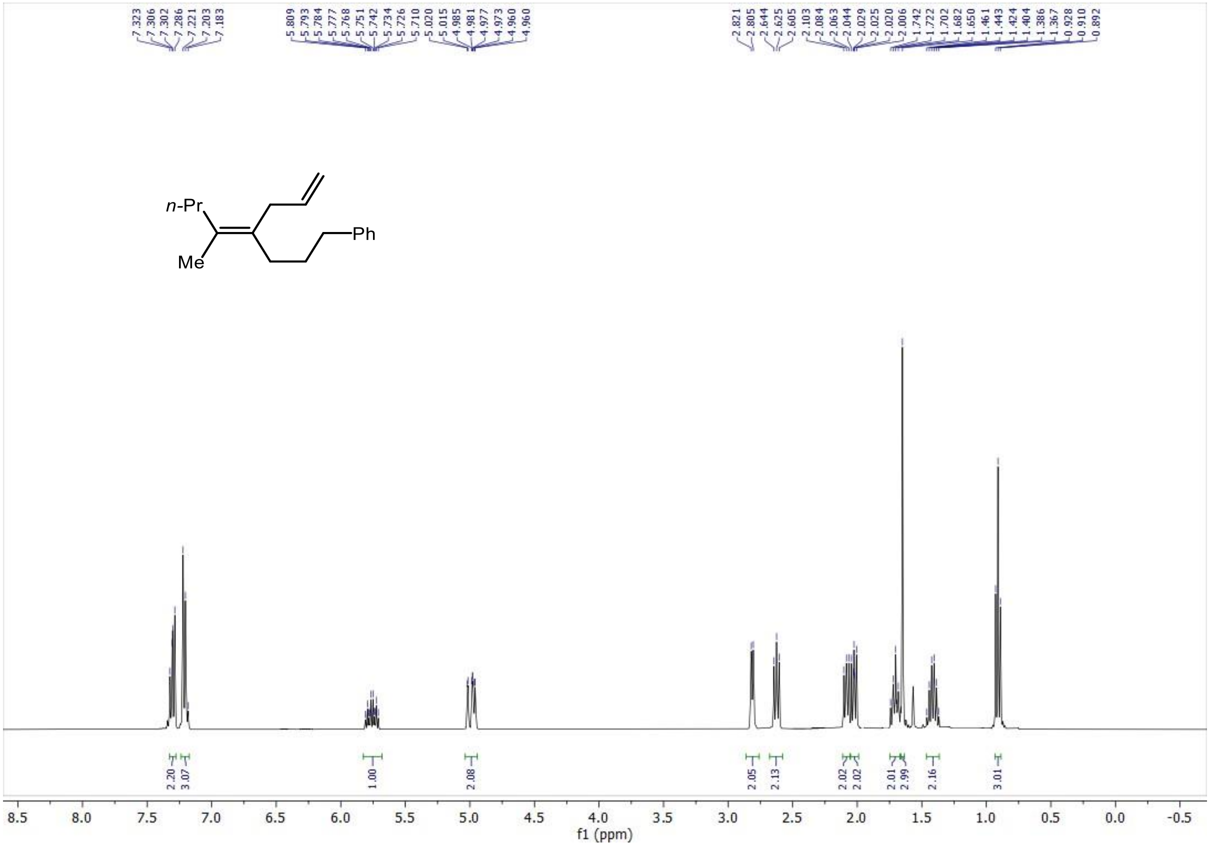

^{13}C NMR (400 MHz, $CDCl_3$) of (*Z*)-**14**

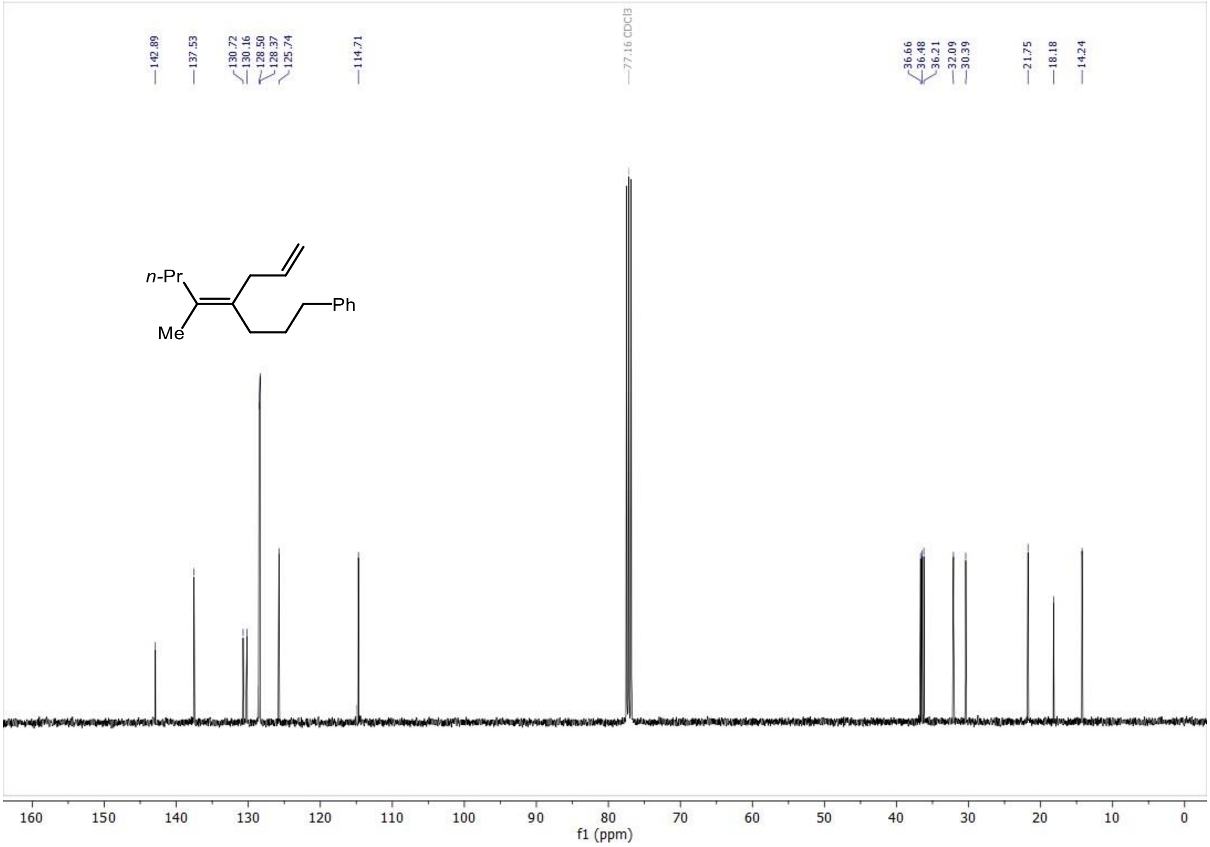

^{1}H NMR (400 MHz, CD_2Cl_2) of **(*Z*)-15**

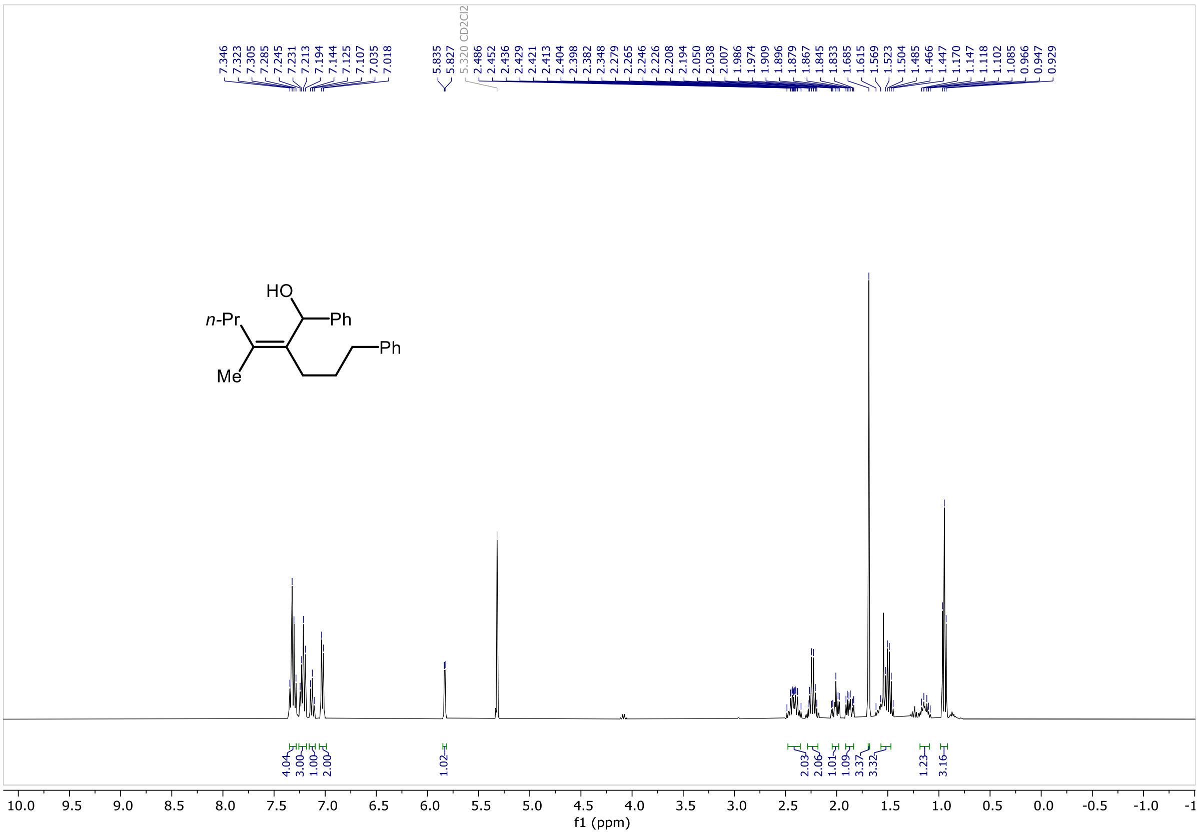

^{13}C NMR (400 MHz, CD_2Cl_2) of **(*Z*)-15**

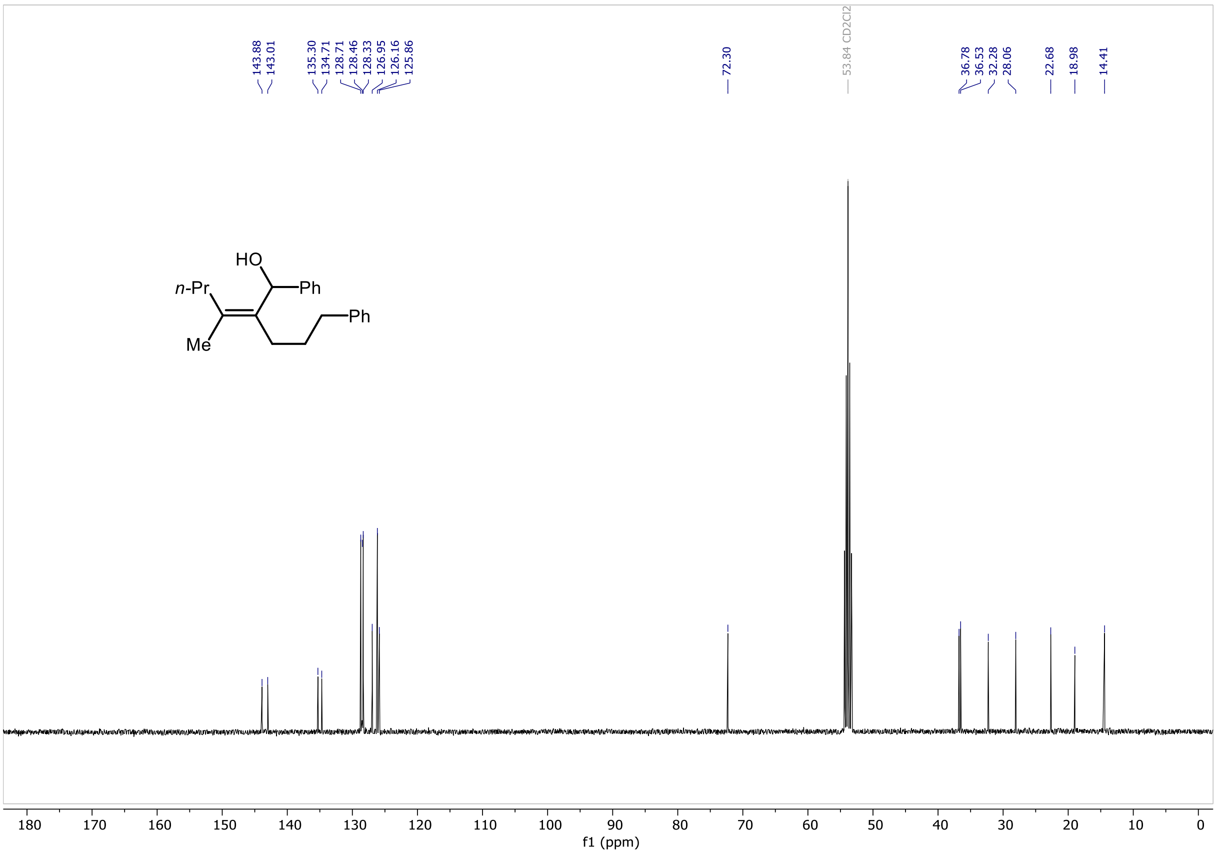

^{1}H NMR (400 MHz, $CDCl_3$) of *cis*-**tamoxifen**

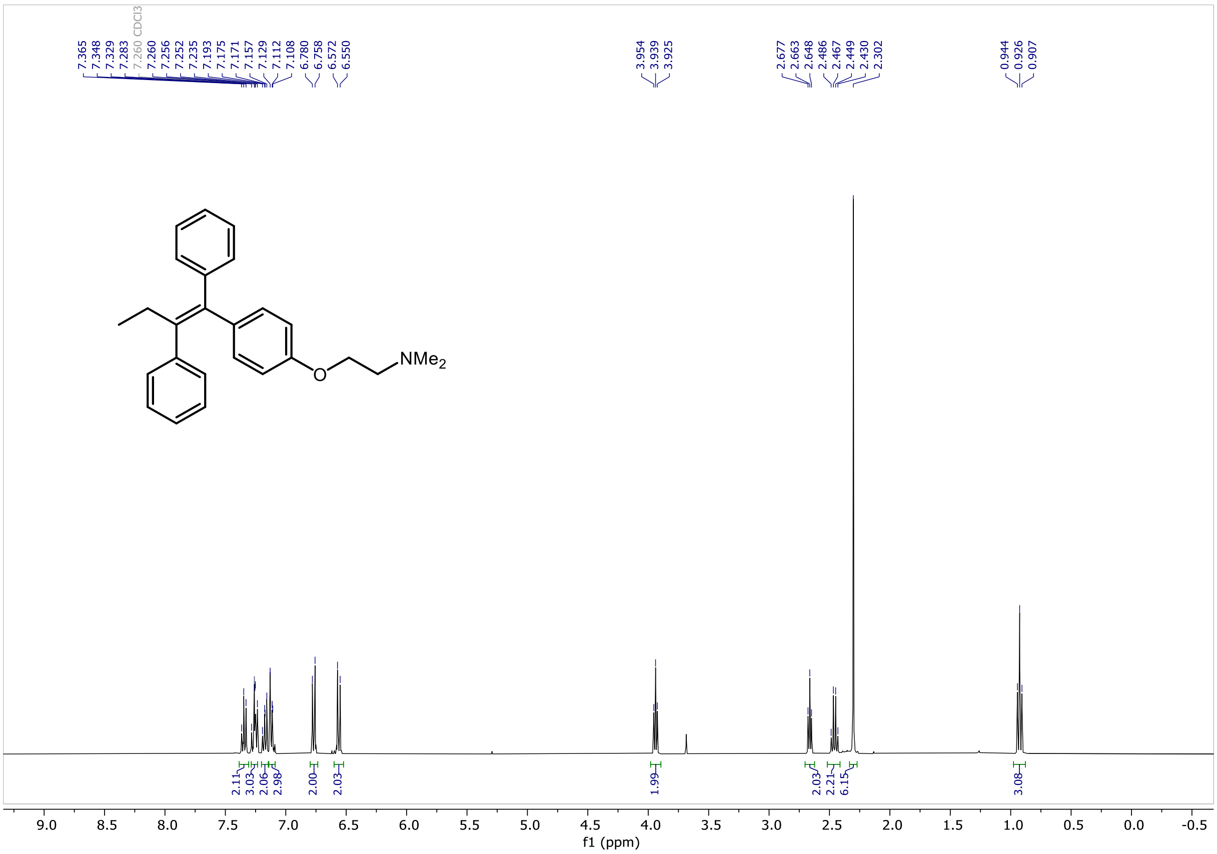

^{13}C NMR (400 MHz, $CDCl_3$) of *cis*-**tamoxifen**

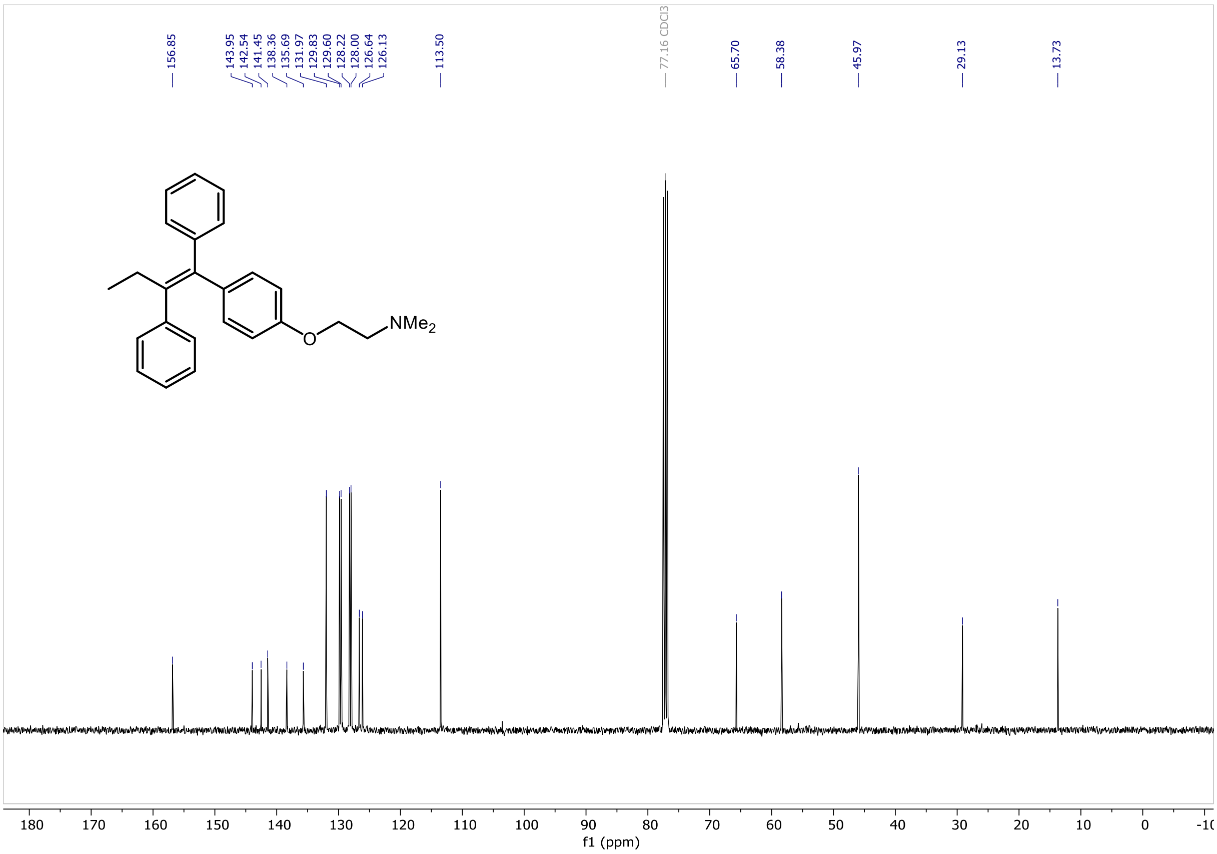

^{1}H NMR (400 MHz, CDCl3) of **18**

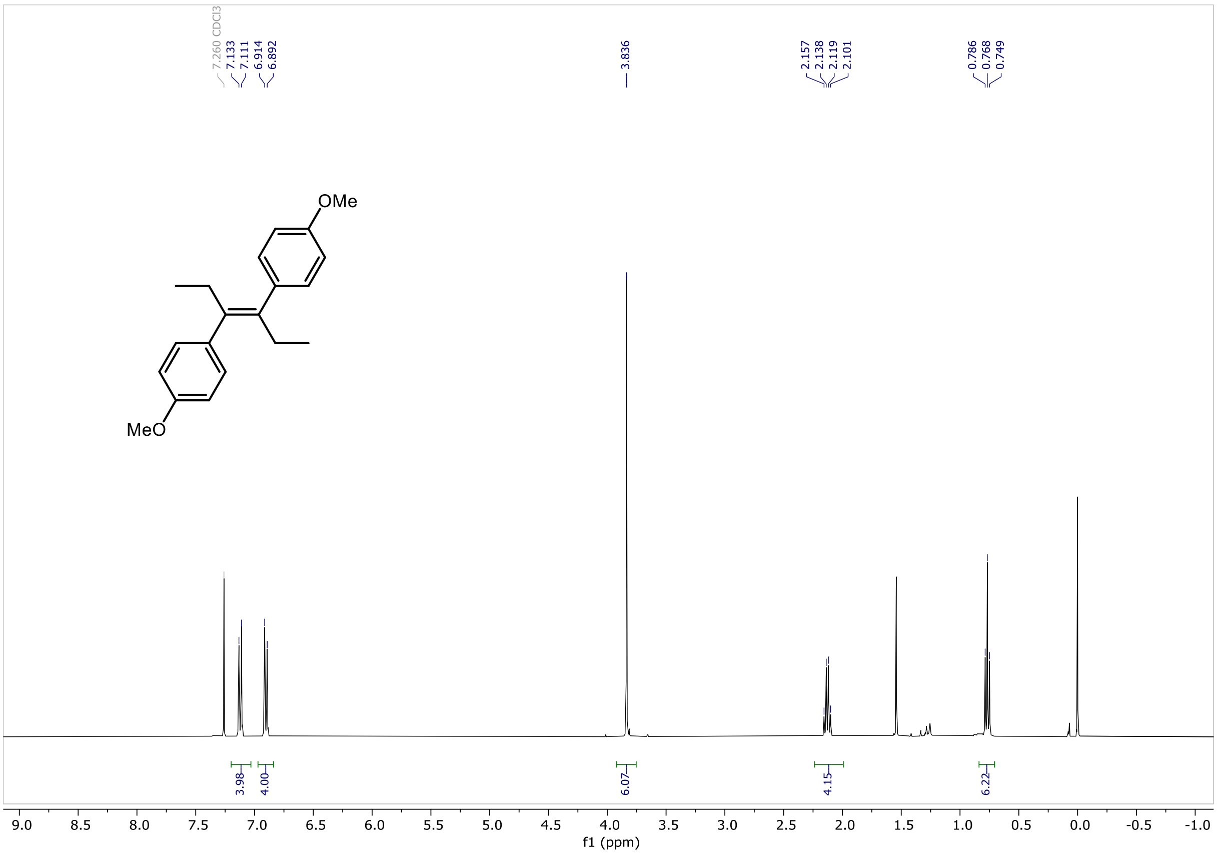

^{13}C NMR (400 MHz, CDCl3) of **18**

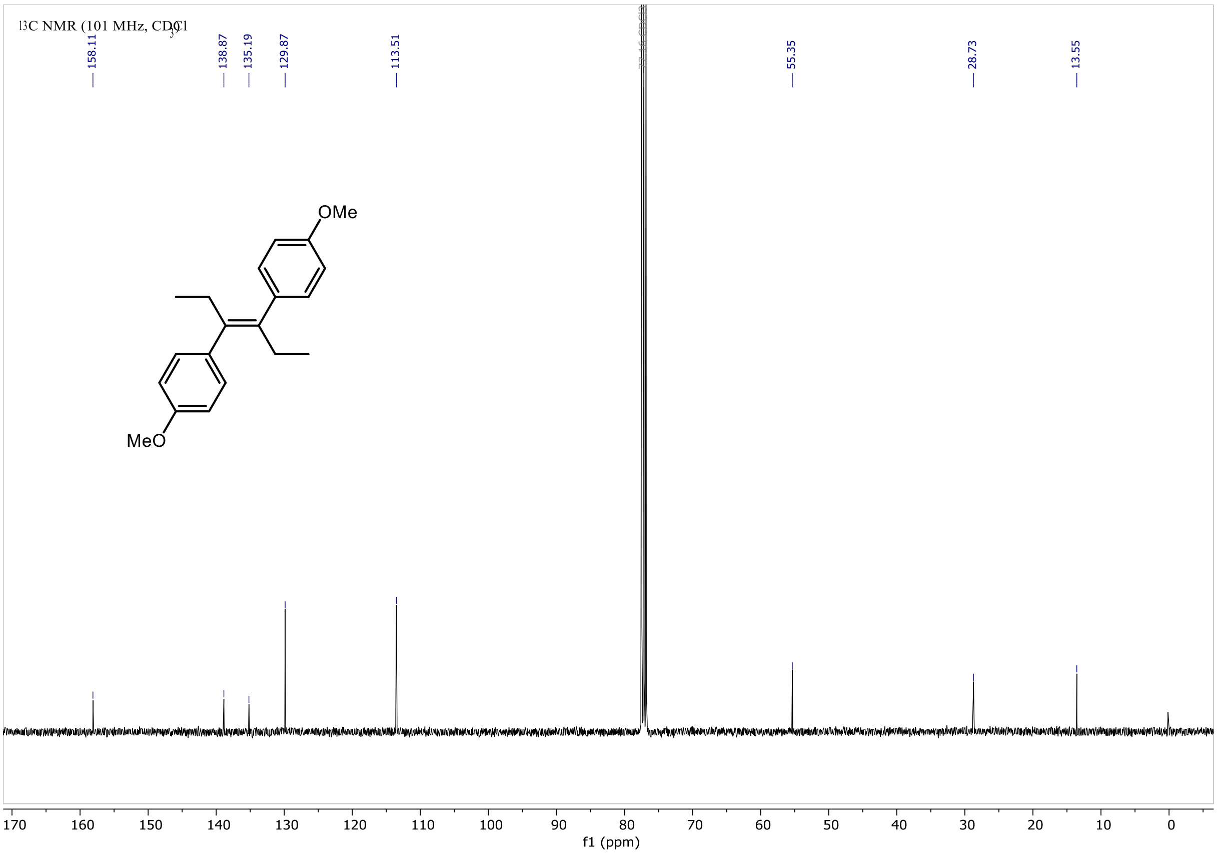

^{1}H NMR (400 MHz, CDCl$_3$) of (*Z*)-**24**

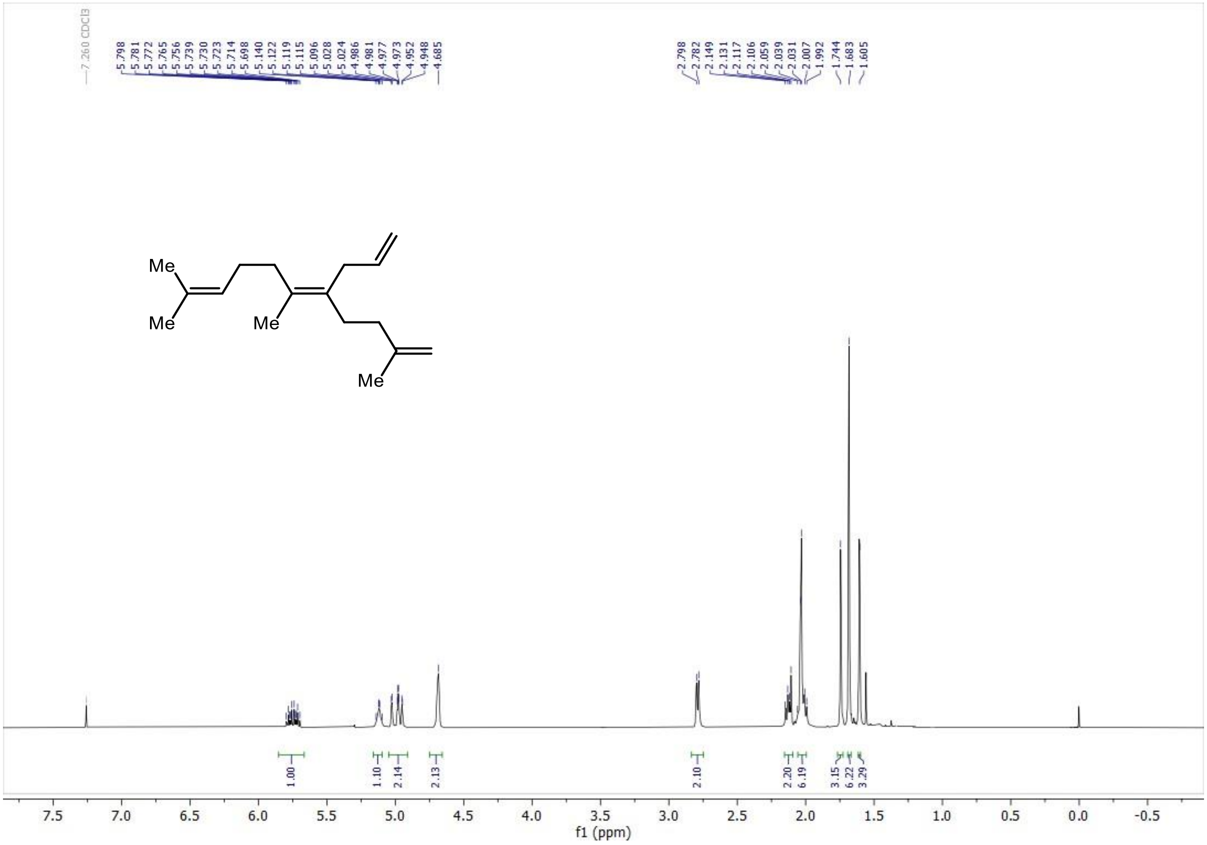

^{13}C NMR (400 MHz, CDCl$_3$) of (*Z*)-**24**

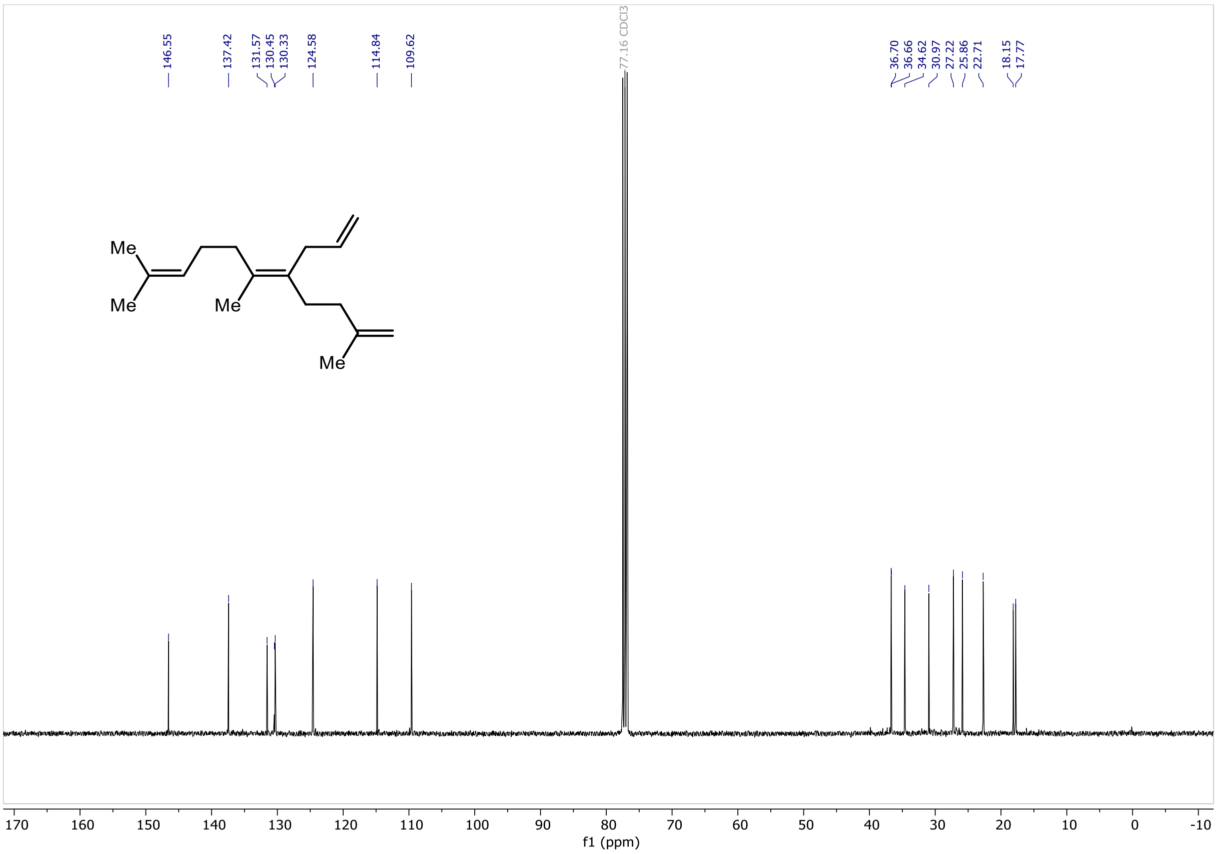

^{1}H NMR (400 MHz, CDCl$_3$) of (*Z*)-γ-**bisabolene**

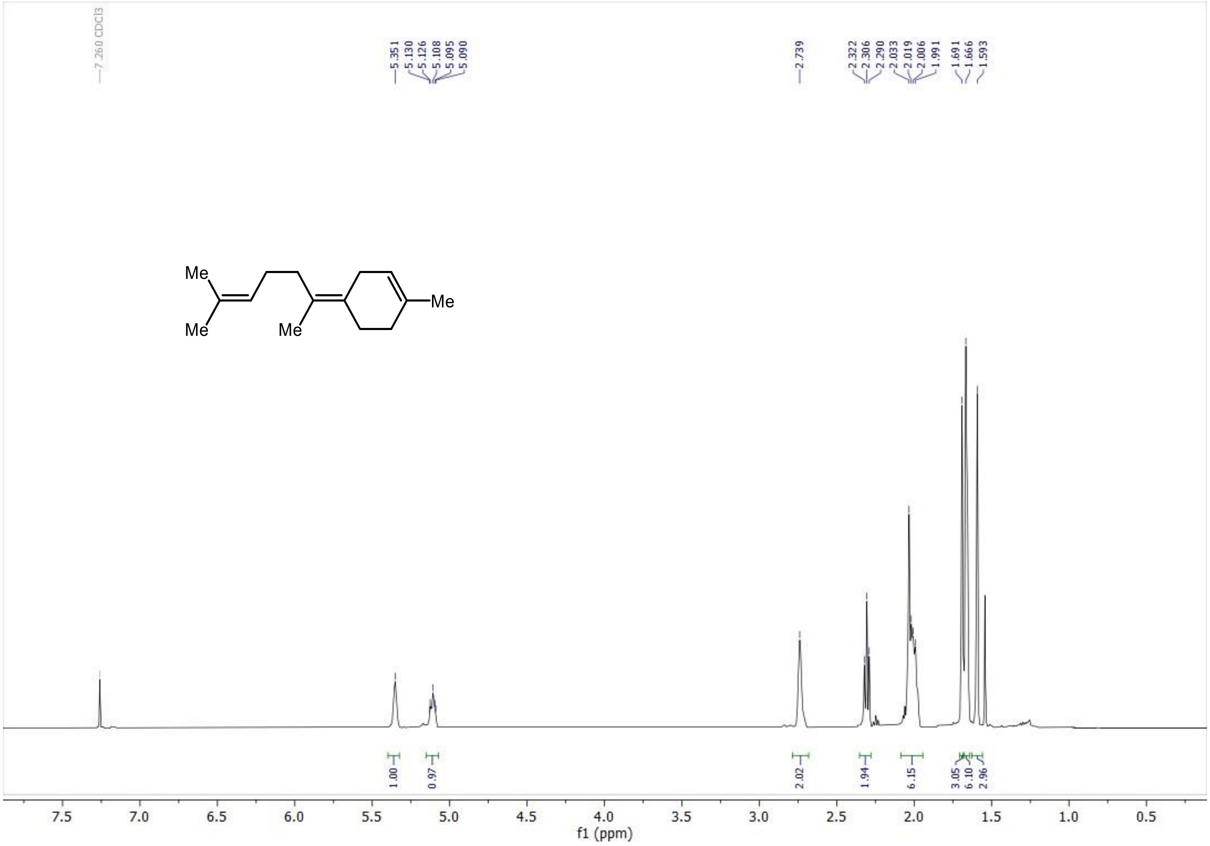

^{13}C NMR (400 MHz, CDCl$_3$) of (*Z*)-γ-**bisabolene**

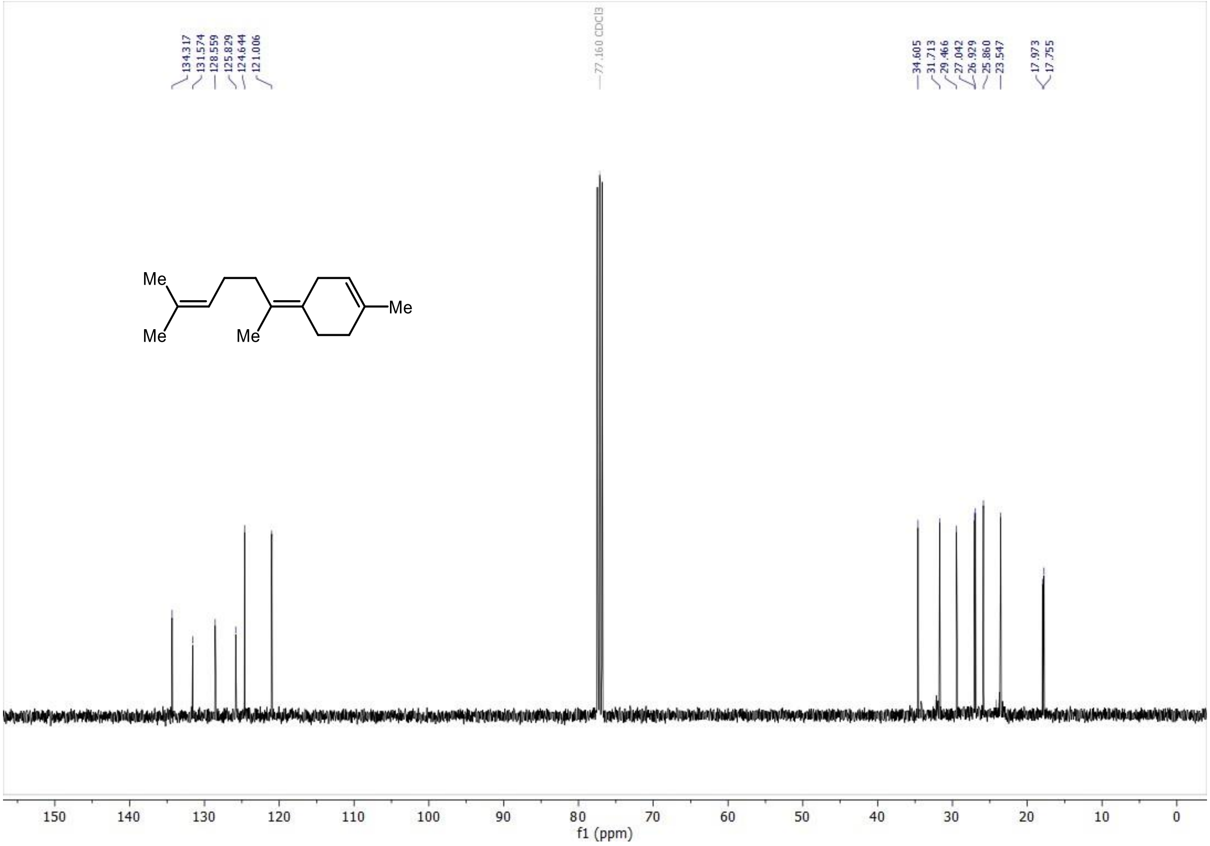

^{1}H NMR (400 MHz, CDCl$_3$) of ***(E)*-24**

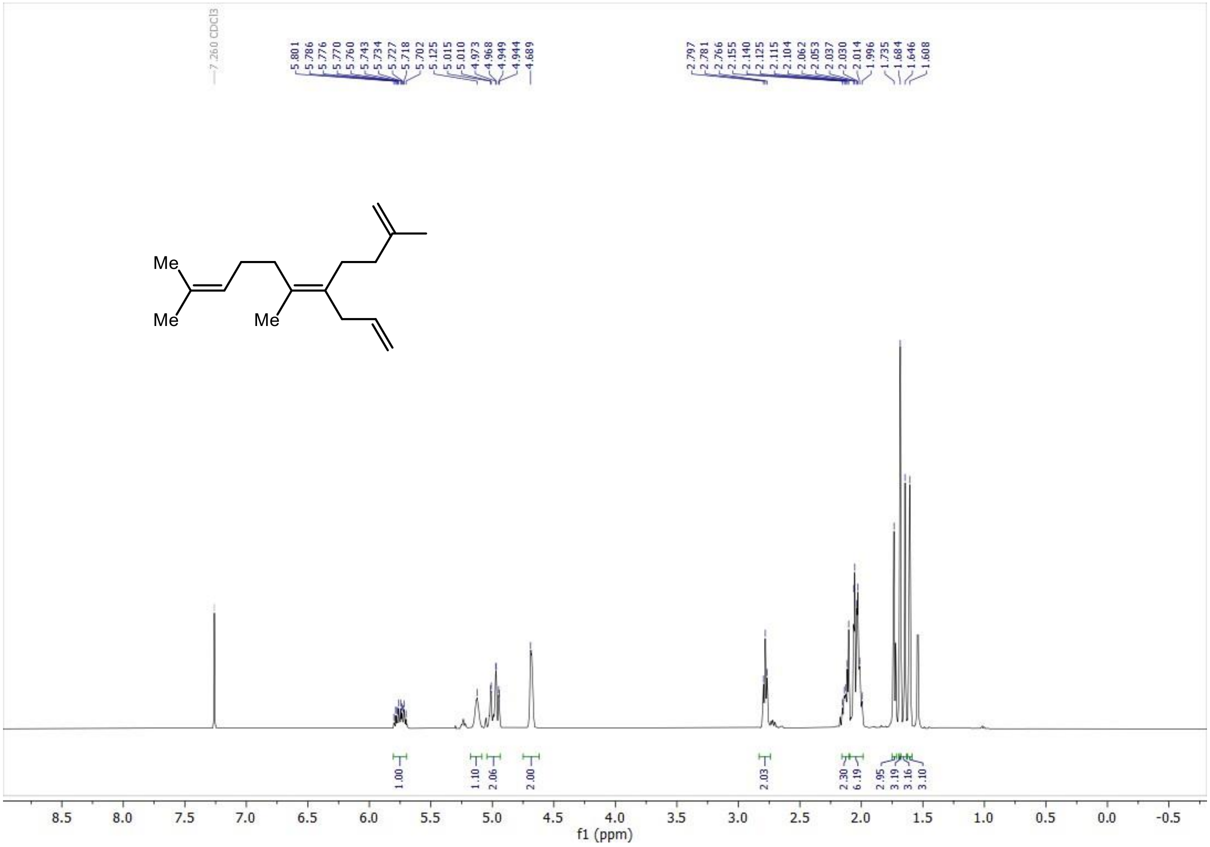

^{13}C NMR (400 MHz, CDCl$_3$) of ***(E)*-24**

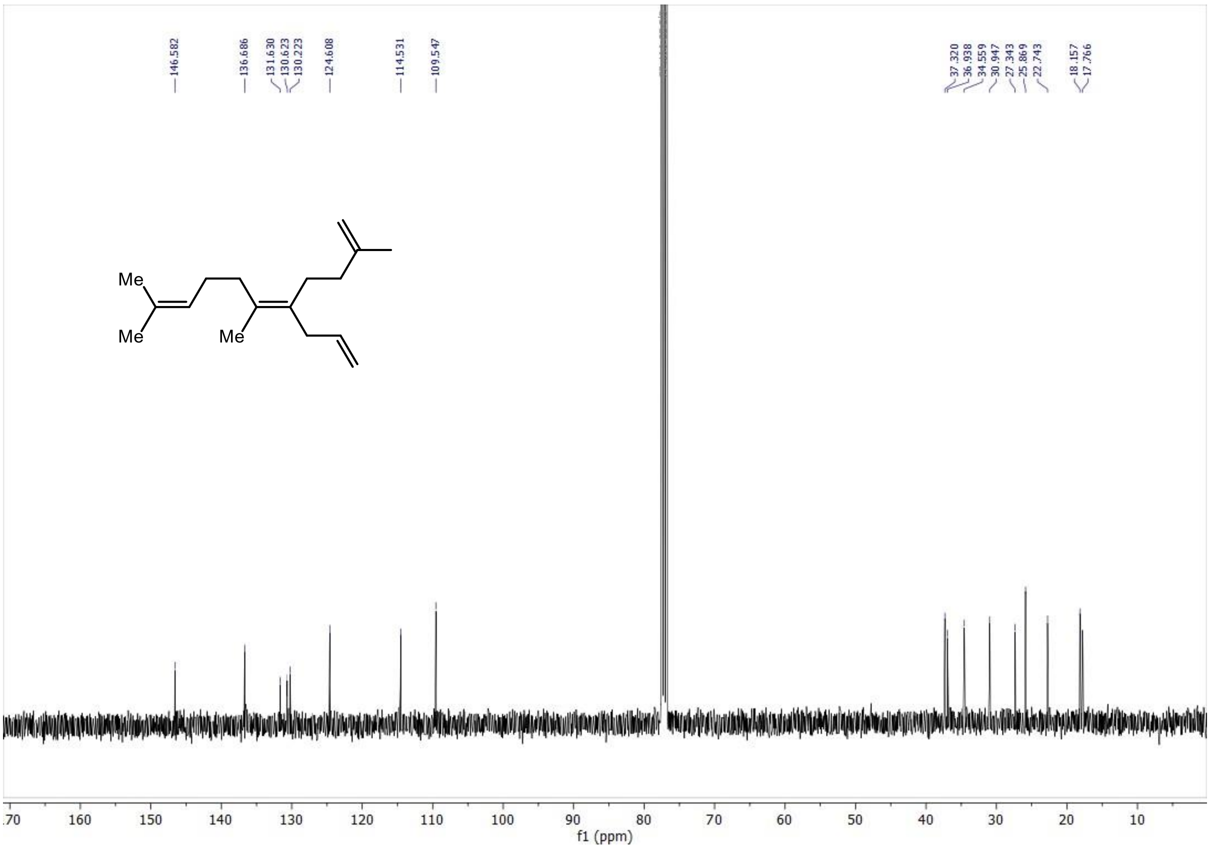

^{1}H NMR (500 MHz, $CDCl_3$) of **(*E*)-γ-bisabolene**

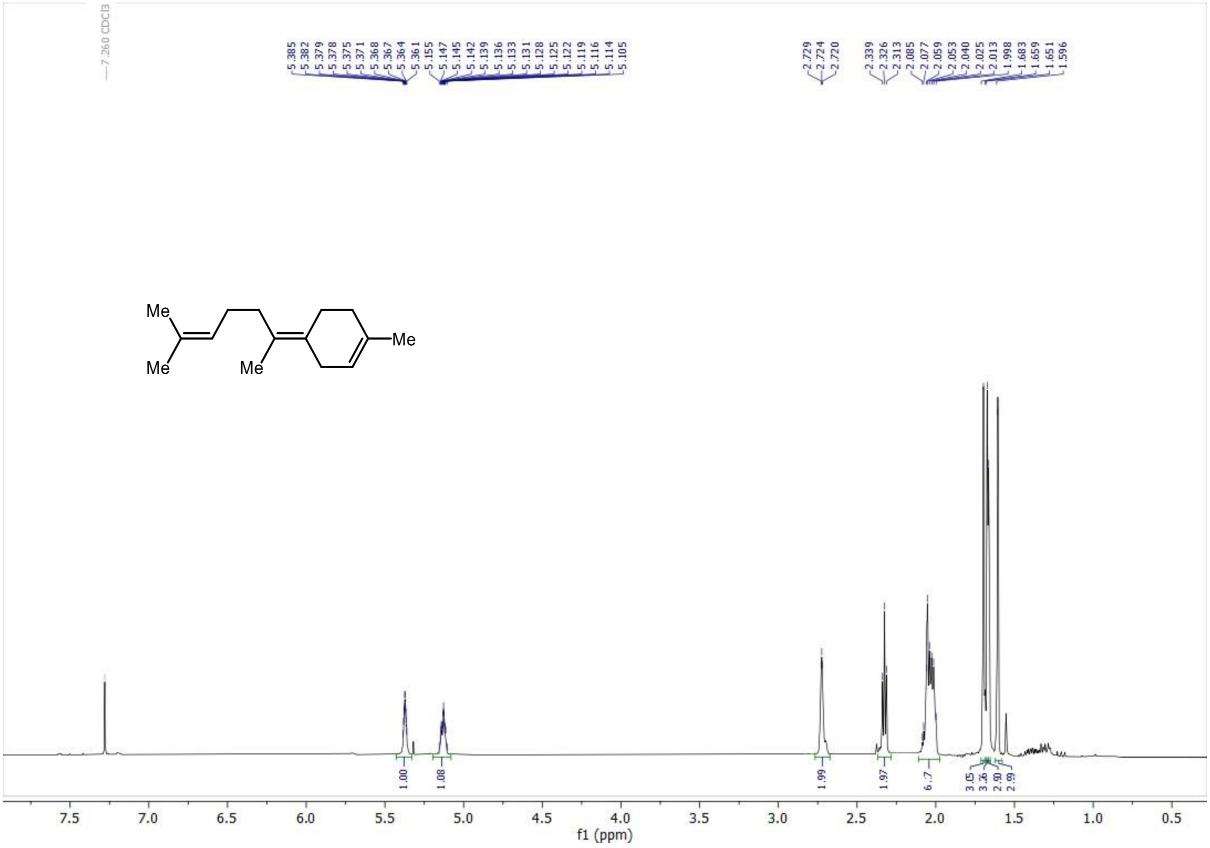

^{13}C NMR (126 MHz, $CDCl_3$) of **(*E*)-γ-bisabolene**

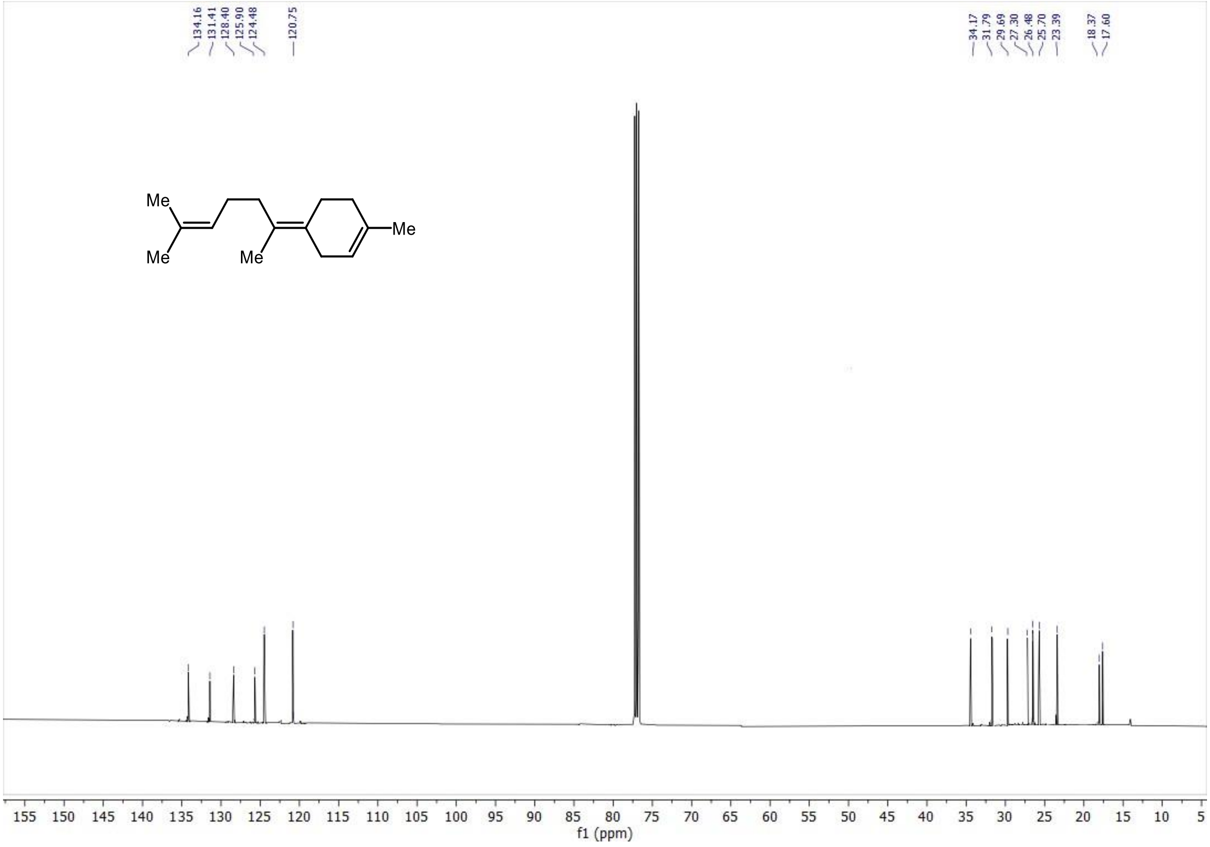

3. COMPUTATIONAL DATA

3.1. Methods

All density functional theory (DFT) geometry optimization calculations were performed using *Gaussian 16* version C.01,[1] and single point energy calculations were refined using ORCA 6.0.0.[2] Geometry optimization calculations were undertaken using the Minnesota hybrid meta-GGA M06-2X[3] density functional and in conjunction Grimme's zero-damped D3 correction.[4] The M06-2X functional has been shown to perform well in computation of main group thermochemistry, and has been recently shown to perform well in the modelling of boronate ion chemistry.[3,5] Subsequent choice for the final single point energy values was decided based on an internal benchmark performed against DLPNO-CCSD(T)/def2-TZVPD level of theory, which showed that the Brémond and Adamo's parameter free PBE0-DH[6] double hybrid functional in conjunction with the Becke-Johnson dampened D3 dispersion correction,[7] most accurate reproduced the coupled cluster results (see Benchmark section). ORCA 6 implements the resolution of identity (RI)[8] approximation for DFT calculations for double hybrid density functionals.

For geometry optimization, Ahlrichs's split valence double-ζ def2-SVPD augmented with additional diffuse functions was utilized for the light elements: H, B, C and O, while the equivalent triple-ζ def2-TZVPD basis set was employed for the remaining I atom.[9,10] Single point calculations were carried out using the triple-ζ def2-TZVPD basis set for all elements. Final results are displayed using both PBE0-DH-D3(BJ)/def2-TZVPD and M06-2X-D3/def2-TZVPD level of theory. All calculations included the integral equation formalism variant of the polarizable continuum model (IEF-PCM), with the SMD solvation model for tetrahydrofuran.[11] Similarly an "ultrafine" pruned (99,590) grid for numerical integration of the exchange-correlation functional and its derivatives was implemented for all *Gaussian 16* calculations, while the ORCA 6 calculations employed the "DEFGRID2" for numerical integration.

The key transitions state structures were further investigated with the aid of the second order perturbation theory analysis of the Fock matrix under the Natural Bonding Orbital (NBO) formalism. The NBO calculation were performed using ORCA 5.0.4 interfaced with the NBO7 package[12] at the PBE0-DH-D3(BJ) level of theory using additionally relaxed MP2 densities (key word: %mp2 density relaxed end"). The desired NBO's were visualized by generating the

corresponding cubes with the aid of the “orca_plot” auxiliary tool and visualized using PyMol utilizing an isovalue of 0.04.[13]

Conformational samplings were carried out using meta-dynamics *CREST* package,[14] with additional manual augmentation for each elementary step. Vibrational frequency calculations were performed to verify that stationary points were either minima or first-order saddle points on the potential energy surface, and for the calculations of Gibbs free energies (G). Grimme’s quasi-harmonic (QHA)[15] model was utilized to correct entropy calculations at 333.15 K (unless otherwise stated), with a frequency cut-off value of 100.0 cm^{-1} using the *GoodVibes* package.[16] *GoodVibes* additionally applies 1 M standard concentration corrections to all individual calculations to account for reactions carried out in solution.[17] The G values of all the energy profiles correspond to the Boltzmann weighted G of all the conformers found in each step, calculated by *GoodVibes*. Boltzmann weighted G (G_{av}) were calculated with *GoodVibes* as:

$$G_{av} = \sum_i G_i p_i$$

where G_i is the relative Gibbs free energy of the corresponding conformers of a certain reaction step and pi is the probability of each conformer calculated as:

$$p_i = \frac{e^{\frac{-G_i}{RT}}}{\sum_i \left(e^{\frac{-G_i}{RT}}\right)}$$

Intrinsic reaction coordinates (IRC) calculations were further undertaken to ensure that the transition state structures connected to the appropriate starting and final geometries.[18] *ORCA* single point calculations input files were generated automatically using the *AQME* python package.[19] Molecular graphics were generated using *PyMol*.

Gaussian 16 optimization (keywords list):

```
# opt freq genecp scrf=(smd,solvent=tetrahydrofuran) emp=gd3 m062x
```

Note: transitions state structures were localized using the “opt=(calcfc,ts,noeigen)” keyword instead

ORCA 6 single-point energy calculation (keyword line):

```
! PBE0-DH D3BJ SMD(tetrahydrofuran) def2-TZVPD def2-TZVPD/C RIJCOSX def2/J
```

AQME automated input file generation (terminal command):

```
python -m aqme –qprep --files "*.log" --qm_input "PBE0-DH D3BJ SMD(tetrahydrofuran) def2-TZVPD def2-TZVPD/C RIJCOSX def2/J" --suffix DH --program orca –mem 32GB –nprocs 16
```

GoodVibes data processing (terminal command):

```
python -m goodvibes *.log --spc DH -c 1 -t [333.15 or 195.15] --imag --dup --pes [name of .yaml file] --xyz --csv
```

3.2. Benchmark

Table S3. Benchmarking results in kcal/mol obtained using ORCA 6 utilizing the def2-TZVPD basis set and the SMD solvation model. (a) Calculation was performed using the CPCM solvation model and unitizing the default NormalPNO threshold in the DLPNO approximation. (b) Single point energy calculations were performed using Gaussian 16.

Method	$\Delta\Delta G_{(1\text{-}2)}^{\ddagger}$	$\Delta G_1^{\ddagger}$	$\Delta G_2^{\ddagger}$	$\Delta G_3^{\ddagger}$
DLPNO-CCSD(T) [(a)]	**8.0**	**16.5**	**24.6**	**11.7**
M06-2X-D3 [(b)]	6.1	7.3	13.4	11.2
ωB97x-D4	4.8	6.2	11.0	10.4
ωB97m-V	5.4	8.2	13.7	11.0
r2SCAN-D4	7.5	4.1	11.6	10.9
PBE0-D4	7.5	7.5	15.1	10.8
B3LYP-D4	8.1	5.8	12.9	10.9
TPSSh-D4	8.1	6.2	14.3	10.7
ωPR2SCAN50-D4	6.4	11.1	17.5	11.1
rev-DSD-PBEP86-D4/2021	7.2	11.2	18.6	11.1
B2PLYP-D4	7.4	8.1	15.5	11.1
ωB97x-2-D3(BJ)	6.3	11.1	17.5	11.2
PWPB95-D3(BJ)	6.3	9.7	16.0	11.7
PBE0-DH-D3(BJ)	**8.1**	**13.0**	**21.0**	**12.6**

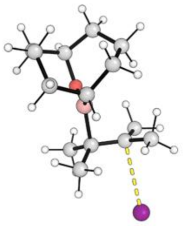

TS-I-Anti_Me ($\Delta G_1^{\ddagger}$)

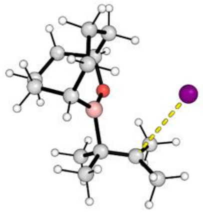

TS-I-Gauche_Me ($\Delta G_2^{\ddagger}$)

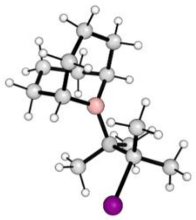

TS-II_Me ($\Delta G_3^{\ddagger}$)

To verify the choice of single point energy refinement was based on an internal benchmark by comparing the activation energy barriers for **TS-I-anti, TS-I-gauche**, and **TS-II**, using a small tetramethyl substituted system. With the exception of M06-2X-D3, all calculations were performed in ORCA 6 and for each functional the highest available level of dispersion correction (VV10, D4, D3(BJ) or D3) was implemented, together with the def2-TZVPD basis set and the SMD implicit solvation model. We initially screened a broad range of hybrid functionals: M06-2X-D3, ωB97x-D4, ωB97m-V, r2SCAN-D4, PBE0-D4, B3LYP-D4 and TPSSH-D4. Comparison of these functionals revealed consistent prediction for **TS-II** across the board, with an average standard deviation of 1.3–1.4 kcal/mol for the **TS-I** activation

energy barriers. Initially, M06-2X-D3 was chosen as it corresponded to the average prediction across all methods investigated and it has been previously successfully implemented in the study for boronate chemistry.

During the reviewing process, we investigated the performance of the chosen level of theory against the high-level DLPNO-CCSD(T)/def2-TZVPD reference, which revealed a chronic but systematic underprediction of both **TS-I-Anti** and **TS-I-Gauche** activation energy barriers, with up to 10 kcal/mol errors. To this extent, we expanded our search to include modern doubly hybrid functionals. While the majority of methods displayed only a marginal increase in performance, the parameter free PBE0-DH with the D3(BJ) dispersion correction was found very accurately predict both the magnitude of the activation energy barriers, as well as the relative energy difference. As such, the final energies are reported at the PBE0-DH-D3(BJ)/def2-TZVPD(SMD=THF) level of theory.

3.3. Computational results

Elimination under neutral conditions:

The results for the Zweifel elimination under neutral conditions reported in the main manuscript are summarized in **Figure S2** for (a) a tetramethyl and (b) tetraethyl substituted derivatives, which display qualitatively similar reaction profiles. Formation of the β-iodo borane **B** from the reaction of boronate **A** with molecular iodine was found to be barrierless on the electronic energy surface. A "downhill" IRC (**Figure S4**) starting from relaxed optimization calculations with a constrained C–I distance of 4.67 Å between one of the iodine atoms and the terminal carbon of the alkene support the barrierless formation of intermediate **B**. The overall process was found to be concerted asynchronous, where the initial C–I bond forms first, followed by the subsequent migration of the boronate alkyl substituent. Further investigation into the key **TS-I** elimination steps were performed for both β-iodo boranes and β-iodo borinic esters (**Figure S3**), which suggest that the borinic esters have lower activation energy barriers for the formation of the borenium cation **C**.

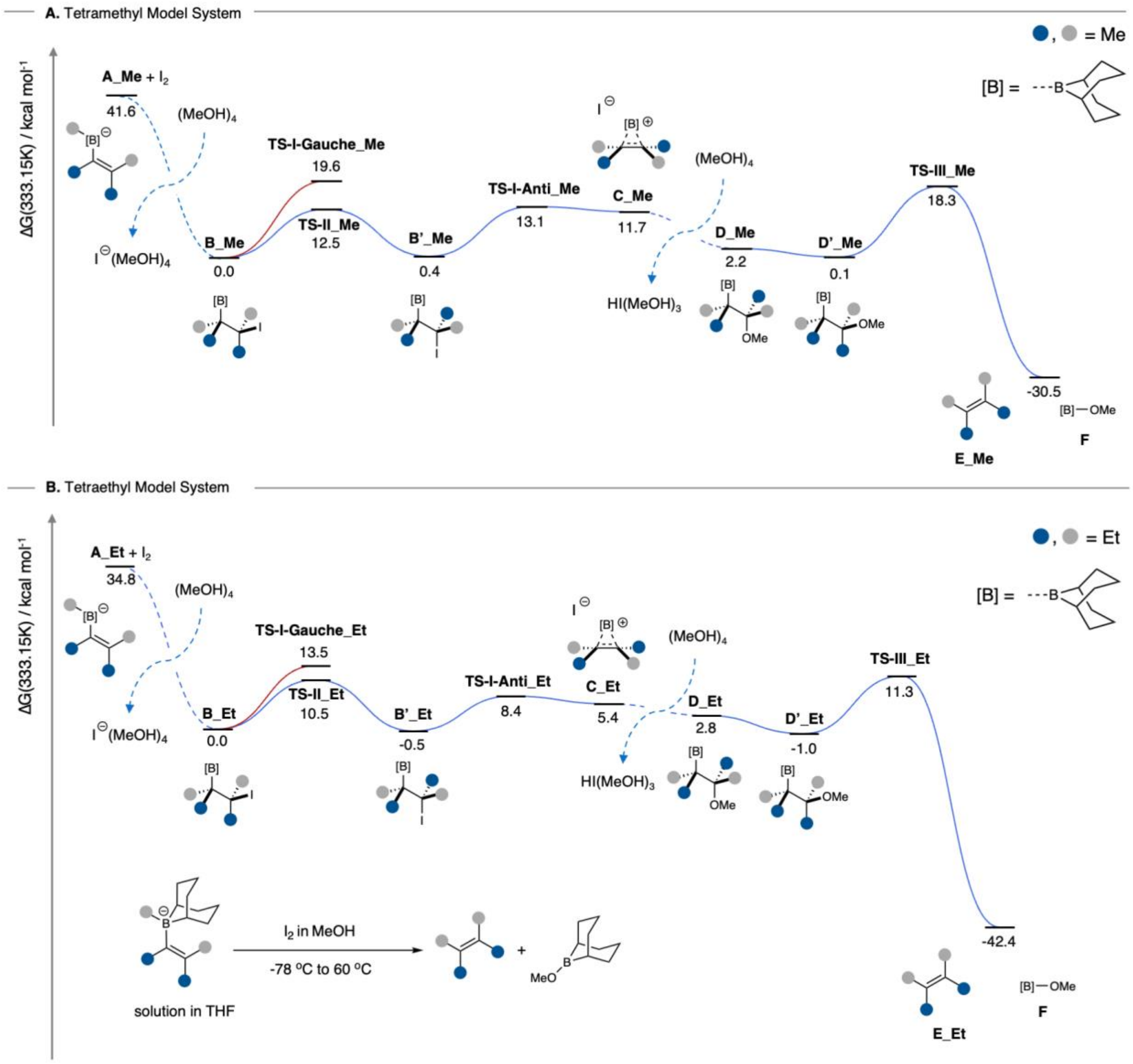

Figure S2. Potential energy surfaces computed for the stereoretentive elimination under neutral conditions utilizing for the A) tetramethyl substituted model system; B) tetraethyl substituted model system at the PBE0-DH-D3(BJ)/def2-TZVPD(SMD=THF)//M06-2X-D3/def2-SVPD;def2-TZVPD[I](SMD=THF) level of theory at 333.15K.

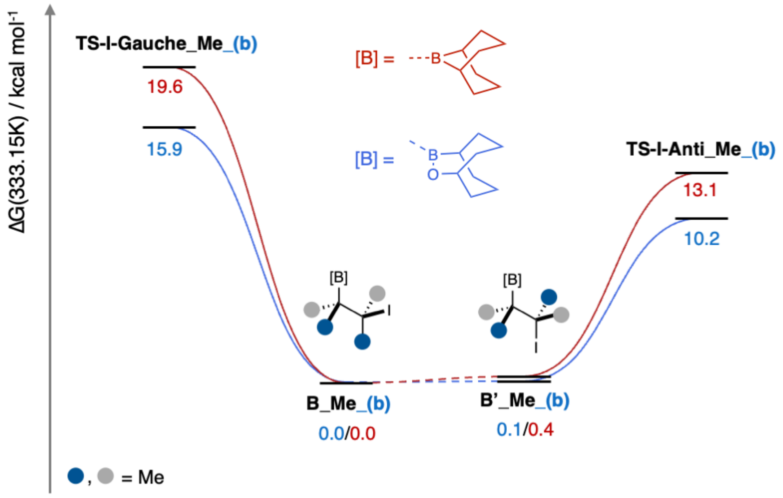

Figure S3. Comparison of β-iodo boranes (red) and β-iodo bornic ester (blue; also denoted with an addition "b" underscript) at the PBE0-DH-D3(BJ)/def2-TZVPD(SMD=THF)//M06-2X-D3/def2-SVPD;def2-TZVPD[I](SMD=THF) level of theory at 333.15K.

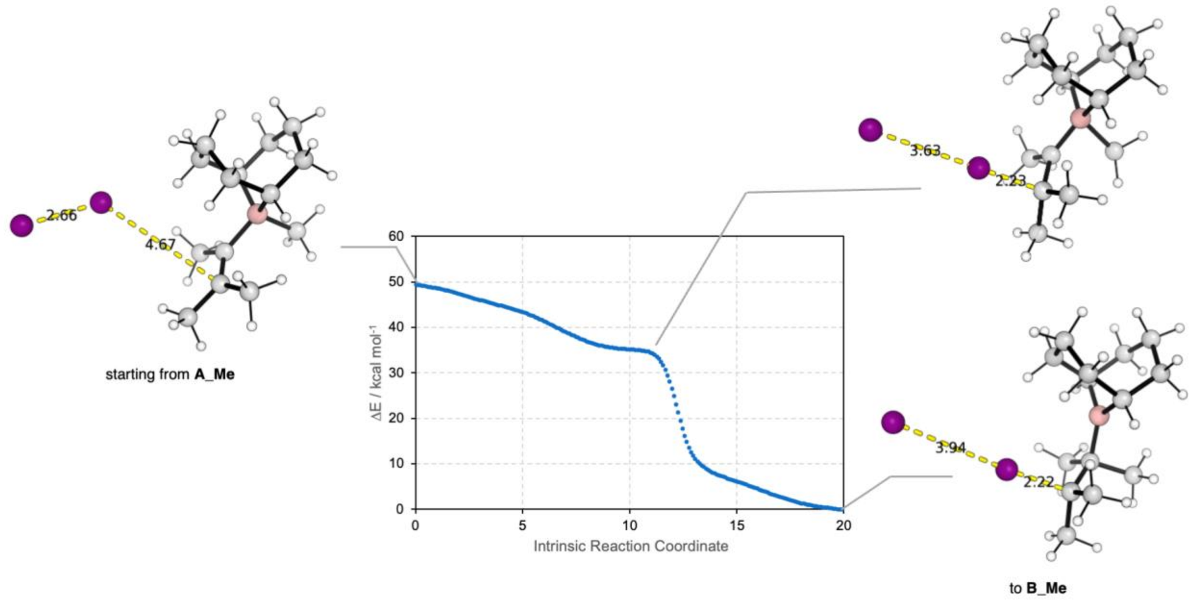

Figure S4. Downhill IRC performed at the M06-2X-D3/def2-SVPD;def2-TZVPD[I](SMD=THF) level of theory, starting from the encounter complex of **A_Me** and iodine. The starting structure was preoptimized with a constrained C–I bond distance.

An alternative syn elimination pathway from intermediate **B**, with formation of alkene with retention of configuration (**E_syn**) and the 9BBN-iodoborane (**I**) was also investigated (**Figure S5**). Extensive transition state search attempts did not reveal the presence of a saddle point. Subsequent relaxed energy surface scans, in which the B–I bond distance was progressively shortened, reveled a sharp increase in electronic energy. At a B–I distance of 1.99 Å, the energy rose to 35 kcal/mol, suggesting that this pathway is highly unfavorable and that a transition state may not exist. We hypothesize that the unfavorable reactivity originates from the very weak B–I bond, as supported by bond dissociation enthalpies (**Figure S6**).

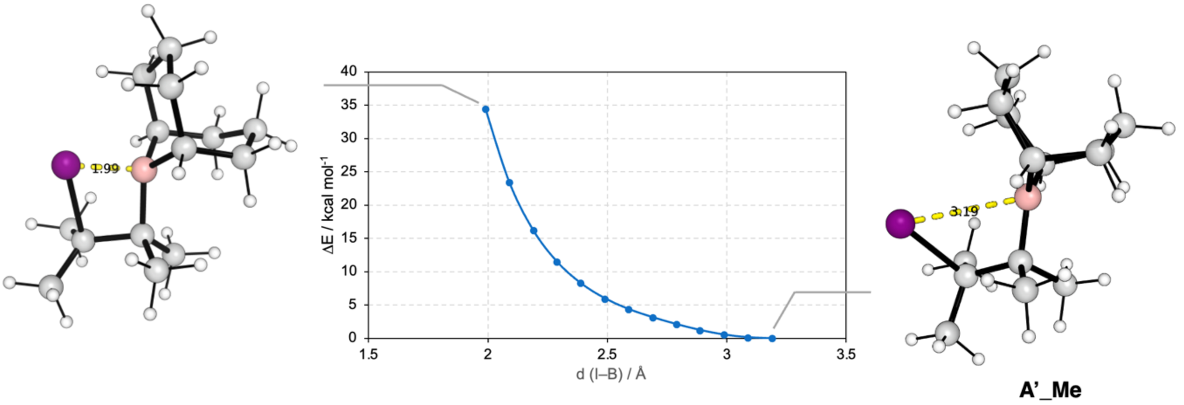

A'_Me

Figure S5. Relaxed I–B distance scan starting from intermediate **A_Me** at the M06-2X-D3/def2-SVPD;def2-TZVPD[I](SMD=THF) level of theory.

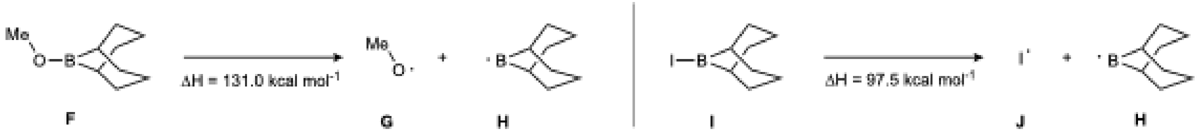

Figure S6. Bond dissociation enthalpies calculations calculated at the PBE0-DH-D3(BJ)/def2-TZVPD(SMD=THF)//M06-2X-D3/def2-SVPD;def2-TZVPD[I](SMD=THF) level of theory.

Table S4. Computed thermochemistry for the elimination under natural conditions for the tetramethyl model system at the PBE0-DH-D3(BJ)/def2-TZVPD(SMD=THF)//M06-2X-D3/def2-SVPD;def2-TZVPD[I](SMD=THF) level of theory at 333.15K.

Species	ΔE_{SPC}	ΔE	ΔZPE	ΔH_{SPC}	T.ΔS	T.qh-ΔS	$\Delta G(T)_{SPC}$	qh-$\Delta G(T)_{SPC}$
**A_Me + 2 $(MeOH)_4$**	56.0	53.9	-3.3	52.7	11.3	11.1	41.4	41.6
**B_Me + $(MeOH)_4$ + $I^-(MeOH)_4$**	0.0	0.0	0.0	0.0	0.0	0.0	0.0	0.0
**TS-I-Gauche_Me + $(MeOH)_4$ + $I^-(MeOH)_4$**	19.7	15.2	-0.5	18.9	-0.9	-0.8	19.8	19.6
**TS-II-Rotation_Me + $(MeOH)_4$ + $I^-(MeOH)_4$**	11.7	10.1	0.1	11.0	-1.9	-1.5	12.9	12.5
**B'_Me + $(MeOH)_4$ + $I^-(MeOH)_4$**	1.1	-0.6	-0.2	1.0	1.1	0.6	0.0	0.4
**TS-I-Anti_Me + $(MeOH)_4$ + $I^-(MeOH)_4$**	14.6	9.2	-1.0	13.8	1.1	0.8	12.7	13.1
**C_Me + $(MeOH)_4$ + $I^-(MeOH)_4$**	14.3	7.6	-1.2	14.1	3.4	2.5	10.7	11.7
**D_Me + $HI(MeOH)_4$ + $I^-(MeOH)_4$**	1.7	-9.9	0.1	0.7	-3.3	-1.5	4.0	2.2
**D'_Me + $HI(MeOH)_4$ + $I^-(MeOH)_4$**	-2.2	-14.2	0.8	-3.0	-5.5	-3.2	2.5	0.1
**TS-III_Me + $HI(MeOH)_4$ + $I^-(MeOH)_4$**	17.1	5.1	-0.5	14.8	-6.0	-3.4	20.8	18.3
**E_Me + F + $HI(MeOH)_4$ + $I^-(MeOH)_4$**	-13.8	-23.5	-2.6	-16.6	11.8	14.0	-28.3	-30.5

Table S5. Computed thermochemistry for the elimination under natural conditions for the tetraethyl model system at the PBE0-DH-D3(BJ)/def2-TZVPD(SMD=THF)//M06-2X-D3/def2-SVPD;def2-TZVPD[I](SMD=THF) level of theory at 333.15K.

Species	ΔE_{SPC}	ΔE	ΔZPE	ΔH_{SPC}	T.ΔS	T.qh-ΔS	$\Delta G(T)_{SPC}$	qh-$\Delta G(T)_{SPC}$

**A_Et + 2 $(MeOH)_4$**	49.0	47.0	-3.4	45.5	11.7	10.7	33.8	34.8
**B_Et + $(MeOH)_4$ + $I^-(MeOH)_4$**	0.0	0.0	0.0	0.0	0.0	0.0	0.0	0.0
**TS-I-Gauche_Et + $(MeOH)_4$ + $I^-(MeOH)_4$**	13.6	8.9	-1.0	12.6	-0.8	-1.0	13.3	13.5
**TS-II-Rotation_Et + $(MeOH)_4$ + $I^-(MeOH)_4$**	9.8	8.9	-0.3	9.1	-1.6	-1.3	10.7	10.5
**B'_Et + $(MeOH)_4$ + $I^-(MeOH)_4$**	-1.0	-2.2	0.1	-1.0	-0.6	-0.4	-0.3	-0.5
**TS-I-Anti_Et + $(MeOH)_4$ + $I^-(MeOH)_4$**	8.6	4.1	-0.5	7.9	-0.6	-0.6	8.5	8.4
**C_Et + $(MeOH)_4$ + $I^-(MeOH)_4$**	7.5	-0.3	-1.0	7.6	4.0	2.3	3.6	5.4
**D_Et + $HI(MeOH)_4$ + $I^-(MeOH)_4$**	1.2	-12.5	0.7	0.5	-4.4	-2.3	4.9	2.8
**D'_Et + $HI(MeOH)_4$ + $I^-(MeOH)_4$**	-3.3	-16.4	0.6	-4.1	-5.0	-3.0	1.0	-1.0
**TS-III_Et + $HI(MeOH)_4$ + $I^-(MeOH)_4$**	10.4	-1.6	-0.8	8.1	-5.5	-3.2	13.6	11.3
**E_Et + F + $HI(MeOH)_4$ + $I^-(MeOH)_4$**	-24.3	-33.6	-3.0	-27.6	13.3	14.8	-40.9	-42.4

Table S6. Comparison of computed thermochemistry for the elimination under natural conditions for the tetramethyl borinic and borane model systems at the PBE0-DH-D3(BJ)/def2-TZVPD(SMD=THF)//M06-2X-D3/def2-SVPD;def2-TZVPD[I](SMD=THF) level of theory at 333.15K.

Species	ΔE_{SPC}	ΔE	ΔZPE	ΔH_{SPC}	$T.\Delta S$	$T.qh\text{-}\Delta S$	$\Delta G(T)_{SPC}$	$qh\text{-}\Delta G(T)_{SPC}$
**TS-I-Gauche_b**	16.8	12.3	-0.8	15.9	0.1	0.0	15.8	15.9
**B_b**	0.0	0.0	0.0	0.0	0.0	0.0	0.0	0.0
**B'_b**	0.4	-0.8	-0.2	0.4	0.4	0.4	0.1	0.1
**TS-I-Anti_b**	11.4	6.6	-0.9	10.7	0.8	0.5	9.9	10.2
**TS-I-Gauche**	19.7	15.2	-0.5	18.9	-0.9	-0.8	19.8	19.6
**B**	0.0	0.0	0.0	0.0	0.0	0.0	0.0	0.0
**B'**	1.1	-0.6	-0.2	1.0	1.1	0.6	0.0	0.4
**TS-I-Anti**	14.6	9.2	-1.0	13.8	1.1	0.8	12.7	13.1

Elimination under basic conditions:

In the presence of base (**Figure 3B**, Method B), even lower barriers for the different steps were found for the β-iodoborinic esters. Methoxide addition to boron was found to be energetically barrierless (**Figure S7a, Figure S8**), as was elimination (forming the tetrasubstituted alkene) from the *anti*-conformer.

Due to the energetically barrierless nature of the methoxide addition in basic conditions, we envisage a non Curtin-Hammett scenario in operation that accounts for the observed stereoselectivity. For the gauche conformer **G_(b)**, following methoxide addition, *syn*-elimination via to the alkene has a lower barrier of 5.3 kcal/mol, via **TS-IV-Syn_(b)**, compared to internal C–C bond rotation ($\Delta G^{\ddagger}$ = 6.6 kcal/mol), leading to the *anti*-conformation, which then undergoes spontaneous elimination. Therefore, the resultant stereoselectivity reflects the initial thermodynamic ratio of the two anti and gauche conformers, **G_(a)** and **G_(b).** The observed 6:1 ratio of products corresponds to a $\Delta G \approx 0.7$ kcal/mol, similar to the calculated $\Delta G = 0.8$ kcal/mol between conformers **B_(b)** and **B'_(b)** at 195.15 K.

In contrast, and as hypothesised, the barriers to elimination are much greater with β-selenoborinic esters (**Figure S7b**). Addition of methoxide forms an intermediate, for which *anti*-conformer **H** eliminates directly over a small barrier of 3.0 kcal/mol, while the gauche conformer **H'** has a much higher barrier to elimination. Instead, it can undergo a lower-barrier bond rotation ($\Delta G^{\ddagger}$ = 10.9 kcal/mol) followed by elimination from the *anti*-conformation. Curtin-Hammett conditions are partially restored, and the selectivity is determined by the relative barriers to elimination. Since this difference is considerably greater than the ratio of *anti:gauche* conformers of the β-iodoborinic esters, this accounts for the higher selectivity observed when using PhSeCl versus I_2.

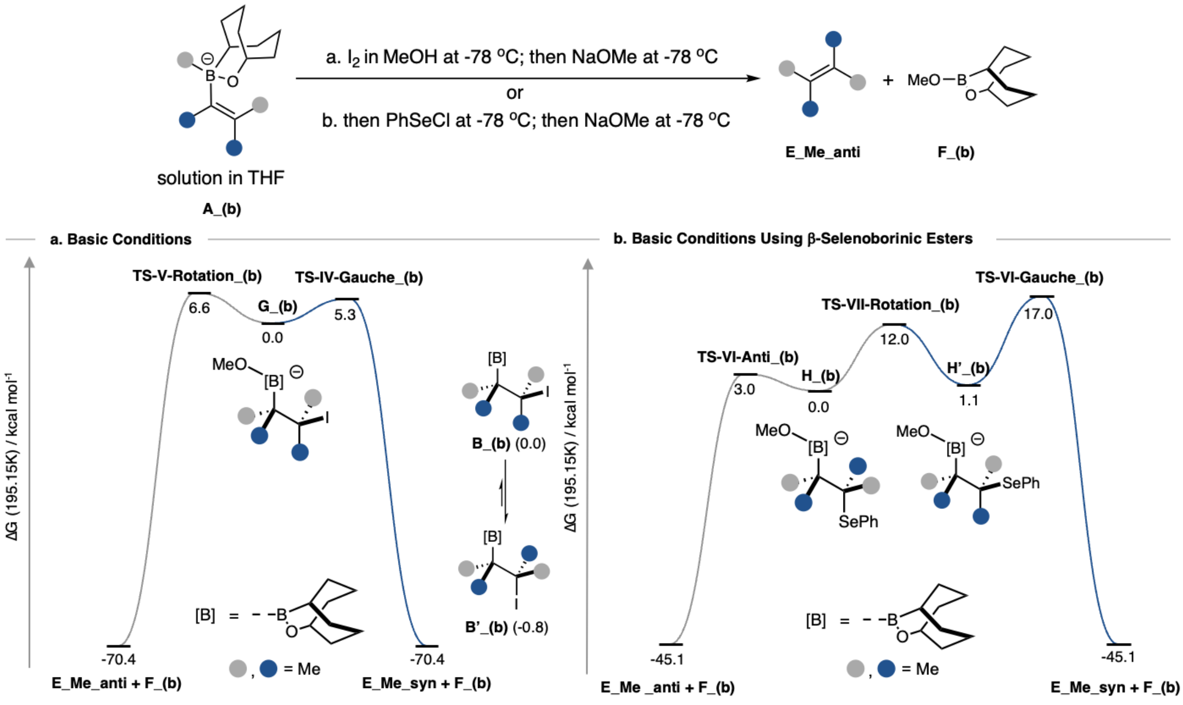

Figure S7. Potential energy surfaces computed for the elimination under basic conditions: a) for β-iodo borinic ester; b) β-selenoborinic ester computed at the M06-2X-D3/def2-TZVPD(SMD=THF)//M06-2X-D3/def2-SVPD;def2-TZVPD[I](SMD=THF) level of theory at 195.15K.

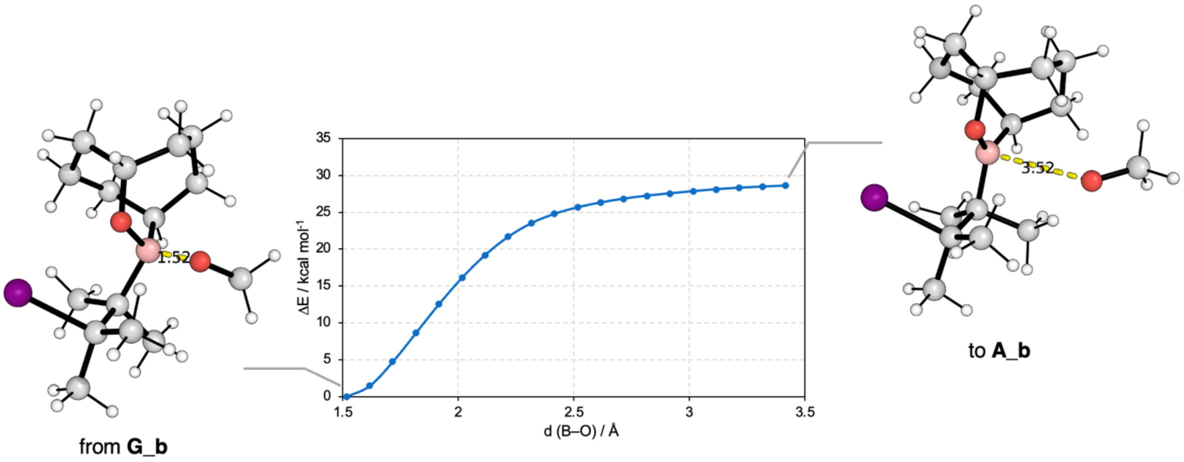

Figure S8. Relaxed MeO–B distance scan starting from intermediate **G_(b)** at the M06-2X-D3/def2-SVPD;def2-TZVPD[I](SMD=THF) level of theory.

Table S7. Computed thermochemistry for the elimination under basic conditions.

Species	ΔE_{SPC}	ΔE	ΔZPE	ΔH_{SPC}	$T.\Delta S$	$T.qh\text{-}\Delta S$	$\Delta G(T)_{SPC}$	$qh\text{-}\Delta G(T)_{SPC}$

**E_Me_anti + F_(b) + I^-**	-55.3	-47.7	-1.8	-55.4	14.8	15.0	-70.2	-70.4
**TS-V-Rotation_(b)**	6.7	6.8	-0.1	6.4	-0.4	-0.3	6.8	6.6
**G_(b)**	0.0	0.0	0.0	0.0	0.0	0.0	0.0	0.0
**TS-IV-Syn_(b)**	6.3	6.8	-0.7	5.6	0.5	0.3	5.1	5.3
**E_Me_syn + F_(b) + I^-**	-55.3	-47.7	-1.8	-55.4	14.8	15.0	-70.2	-70.4
**E_Me_anti + F_(b) + $PhSe^-$**	0.0	0.0	0.0	0.0	0.0	0.0	0.0	0.0
**TS-VI-Anti_(b)**	30.5	21.7	1.5	30.8	-16.9	-17.8	47.7	48.6
**H_(b)**	26.5	17.0	2.3	27.4	-17.0	-18.0	44.5	45.4
**TS-VII-Rotation_(b)**	37.8	28.4	2.5	38.6	-18.0	-18.7	56.6	57.3
**H'_(b)**	27.2	17.7	2.5	28.3	-17.6	-18.3	45.9	46.6
**TS-VI-Gauche_(b)**	44.4	35.9	1.3	44.6	-16.7	-17.6	61.4	62.3
**E_Me_Syn + F_(b) + $PhSe^-$**	0.0	0.0	0.0	0.0	0.0	0.0	0.0	0.0

3.4. Thermochemistry

Legend:

E_{SPC} = energy obtained using single-point energy correction

E = energy obtained at the geometry optimization

ZPE = zero-point energy

H_{SPC} = enthalpy corrected with E_{SPC}

T·S = temperature times entropy with no correction

T·qh-S = temperature times entropy with quasi-harmonic S correction

$G(T)_{SPC}$ = Gibbs free energy corrected only with E_{SPC}

qh-$G(T)_{SPC}$ = Gibbs free energy with E_{SPC} and quasi-harmonic S correction

ν_{imag} = imaginary frequencies

Table S8. Compiled thermochemical data for structures computed at the at the PBE0-DH-D3(BJ)/def2-TZVPD(SMD=THF)//M06-2X-D3/def2-SVPD;def2-TZVPD[I](SMD=THF) level of theory; Gibbs free energies are reported at 333.15 K

Structure	E_{SPC}	E	ZPE	H_{SPC}	T.S	T.qh-S	$G(T)_{SPC}$	qh-$G(T)_{SPC}$	ν_{imag} / cm^{-1}
A_Et_conf_1	-730.807825	-730.485802	0.489722	-730.291086	0.078840	0.076404	-730.369926	-730.367490	
A_Et_conf_10	-730.807656	-730.484902	0.488874	-730.291439	0.079777	0.077315	-730.371215	-730.368754	
A_Et_conf_11	-730.809661	-730.486333	0.487887	-730.293816	0.082655	0.079135	-730.376471	-730.372952	
A_Et_conf_12	-730.807099	-730.485202	0.489712	-730.290441	0.077714	0.076208	-730.368155	-730.366649	
A_Et_conf_13	-730.805565	-730.483965	0.490003	-730.288882	0.076873	0.075505	-730.365756	-730.364387	

A_Et_conf_14	-730.808781	-730.486474	0.488959	-730.292524	0.079526	0.077203	-730.372049	-730.369727
A_Et_conf_15	-730.807942	-730.485963	0.490222	-730.290877	0.077760	0.076022	-730.368636	-730.366898
A_Et_conf_16	-730.807509	-730.485592	0.489480	-730.290944	0.078636	0.076627	-730.369581	-730.367571
A_Et_conf_17	-730.801989	-730.480565	0.489440	-730.285499	0.078423	0.076447	-730.363922	-730.361945
A_Et_conf_18	-730.807410	-730.485392	0.489146	-730.290962	0.079278	0.077167	-730.370240	-730.368130
A_Et_conf_19	-730.800469	-730.479789	0.489493	-730.283930	0.078187	0.076444	-730.362117	-730.360374
A_Et_conf_2	-730.802589	-730.481806	0.489297	-730.286332	0.077718	0.076282	-730.364050	-730.362614
A_Et_conf_20	-730.800637	-730.479526	0.489718	-730.283921	0.077693	0.076379	-730.361614	-730.360299
A_Et_conf_21	-730.807645	-730.485625	0.489352	-730.291179	0.078604	0.076634	-730.369783	-730.367812
A_Et_conf_22	-730.801166	-730.479639	0.489306	-730.284698	0.079175	0.076856	-730.363873	-730.361555
A_Et_conf_23	-730.808451	-730.486292	0.489150	-730.291917	0.079599	0.077396	-730.371515	-730.369313
A_Et_conf_24	-730.801150	-730.479644	0.489123	-730.284829	0.080455	0.077559	-730.365284	-730.362387
A_Et_conf_25	-730.798880	-730.477169	0.489244	-730.282341	0.079373	0.077166	-730.361713	-730.359507
A_Et_conf_26	-730.799588	-730.478945	0.489234	-730.283210	0.078346	0.076691	-730.361556	-730.359901
A_Et_conf_27	-730.802951	-730.481022	0.489176	-730.286484	0.079302	0.077076	-730.365786	-730.363560
A_Et_conf_28	-730.802960	-730.481043	0.489108	-730.286638	0.079097	0.077012	-730.365735	-730.363650
A_Et_conf_29	-730.804084	-730.482507	0.489545	-730.287484	0.078371	0.076470	-730.365855	-730.363954
A_Et_conf_3	-730.805678	-730.483375	0.488471	-730.289609	0.081758	0.078095	-730.371367	-730.367704
A_Et_conf_30	-730.795439	-730.474246	0.489571	-730.278826	0.078447	0.076416	-730.357273	-730.355242
A_Et_conf_4	-730.807277	-730.485075	0.489472	-730.290628	0.078826	0.076830	-730.369454	-730.367458
A_Et_conf_5	-730.801946	-730.480255	0.489022	-730.285618	0.079542	0.077215	-730.365160	-730.362833
A_Et_conf_6	-730.803358	-730.481804	0.488813	-730.287226	0.079484	0.077143	-730.366710	-730.364369
A_Et_conf_7	-730.800108	-730.478858	0.489033	-730.283961	0.078639	0.076592	-730.362599	-730.360553
A_Et_conf_8	-730.808393	-730.486465	0.489187	-730.292094	0.078719	0.076687	-730.370813	-730.368781
A_Et_conf_9	-730.806402	-730.483993	0.489205	-730.289925	0.079263	0.076991	-730.369189	-730.366917
A_Me_conf_1	-573.676904	-573.426348	0.372452	-573.282635	0.068275	0.066521	-573.350910	-573.349157

A_Me_conf_2	-573.669809	-573.419820	0.372660	-573.275286	0.068797	0.066779	-573.344083	-573.342065
B_Et_conf_1	-1028.428869	-1028.042225	0.496066	-1027.904412	0.081873	0.080044	-1027.986284	-1027.984456
B_Et_conf_10	-1028.426231	-1028.039053	0.495374	-1027.902228	0.082938	0.080827	-1027.985166	-1027.983055
B_Et_conf_11	-1028.427799	-1028.039153	0.495562	-1027.903465	0.083668	0.081208	-1027.987133	-1027.984672
B_Et_conf_12	-1028.426931	-1028.039091	0.495289	-1027.902718	0.084434	0.081663	-1027.987152	-1027.984381
B_Et_conf_13	-1028.425585	-1028.037919	0.495507	-1027.901263	0.084531	0.081424	-1027.985794	-1027.982687
B_Et_conf_15	-1028.425046	-1028.037597	0.495233	-1027.900868	0.083953	0.081550	-1027.984821	-1027.982418
B_Et_conf_16	-1028.425477	-1028.038327	0.496054	-1027.900733	0.083402	0.080884	-1027.984135	-1027.981617
B_Et_conf_17	-1028.424748	-1028.039076	0.496544	-1027.899890	0.081494	0.079841	-1027.981384	-1027.979730
B_Et_conf_19	-1028.425827	-1028.039740	0.495840	-1027.901271	0.083090	0.080942	-1027.984362	-1027.982213
B_Et_conf_2	-1028.427725	-1028.041379	0.496159	-1027.903085	0.082436	0.080438	-1027.985521	-1027.983522
B_Et_conf_20	-1028.425280	-1028.039237	0.495997	-1027.900810	0.082303	0.080298	-1027.983113	-1027.981108
B_Et_conf_21	-1028.423346	-1028.036927	0.496198	-1027.898726	0.082029	0.080191	-1027.980755	-1027.978917
B_Et_conf_22	-1028.424436	-1028.037990	0.496289	-1027.899591	0.082761	0.080571	-1027.982352	-1027.980162
B_Et_conf_23	-1028.422039	-1028.037257	0.496331	-1027.897235	0.082131	0.080278	-1027.979366	-1027.977513
B_Et_conf_24	-1028.426891	-1028.040345	0.496267	-1027.902152	0.082004	0.080209	-1027.984156	-1027.982361
B_Et_conf_25	-1028.421894	-1028.035524	0.495965	-1027.897131	0.083738	0.081155	-1027.980869	-1027.978286
B_Et_conf_26	-1028.422589	-1028.035514	0.495958	-1027.898049	0.082845	0.080600	-1027.980893	-1027.978649
B_Et_conf_27	-1028.421000	-1028.035630	0.495974	-1027.896309	0.083067	0.080965	-1027.979376	-1027.977274
B_Et_conf_28	-1028.422591	-1028.036697	0.496275	-1027.897821	0.081813	0.080227	-1027.979634	-1027.978048
B_Et_conf_29	-1028.423529	-1028.037779	0.496185	-1027.898850	0.082019	0.080287	-1027.980870	-1027.979138
B_Et_conf_3	-1028.428675	-1028.041167	0.495643	-1027.904340	0.083120	0.080884	-1027.987460	-1027.985224
B_Et_conf_30	-1028.423729	-1028.036809	0.496920	-1027.898576	0.081755	0.079754	-1027.980331	-1027.978330
B_Et_conf_31	-1028.428848	-1028.040916	0.496338	-1027.904087	0.082453	0.080185	-1027.986540	-1027.984272
B_Et_conf_32	-1028.421786	-1028.036015	0.496617	-1027.896759	0.082236	0.080192	-1027.978995	-1027.976951
B_Et_conf_33	-1028.424105	-1028.036604	0.495786	-1027.899853	0.081861	0.080150	-1027.981714	-1027.980003

B_Et_conf_34	-1028.422713	-1028.036675	0.496159	-1027.898208	0.081775	0.079964	-1027.979983	-1027.978172
B_Et_conf_35	-1028.424549	-1028.037701	0.496148	-1027.899767	0.082438	0.080593	-1027.982205	-1027.980360
B_Et_conf_36	-1028.420713	-1028.034935	0.496792	-1027.895748	0.080975	0.079474	-1027.976723	-1027.975222
B_Et_conf_4	-1028.429811	-1028.042907	0.496147	-1027.905245	0.082139	0.080137	-1027.987384	-1027.985382
B_Et_conf_5	-1028.425186	-1028.037837	0.495659	-1027.900865	0.083488	0.080945	-1027.984352	-1027.981810
B_Et_conf_6	-1028.427272	-1028.039463	0.495682	-1027.903040	0.082474	0.080531	-1027.985514	-1027.983571
B_Et_conf_7	-1028.426264	-1028.039170	0.496050	-1027.901794	0.081725	0.080127	-1027.983519	-1027.981921
B_Et_conf_8	-1028.426784	-1028.040031	0.495807	-1027.902181	0.083337	0.081196	-1027.985518	-1027.983377
B_Et_conf_9	-1028.425415	-1028.038368	0.495633	-1027.901123	0.083160	0.080872	-1027.984283	-1027.981994
B_Me_conf_1	-871.304159	-870.989875	0.380092	-870.901658	0.070239	0.068385	-870.971896	-870.970043
B_Me_conf_2	-871.303933	-870.989756	0.379890	-870.901536	0.070159	0.068650	-870.971694	-870.970185
B_Me_conf_3	-871.303211	-870.989587	0.379840	-870.900828	0.070666	0.068817	-870.971494	-870.969645
B_Me_conf_4	-871.302687	-870.988364	0.379938	-870.900211	0.070243	0.068770	-870.970454	-870.968981
B_Me_conf_5	-871.307496	-870.992380	0.380089	-870.904897	0.070681	0.068727	-870.975579	-870.973624
B_Me_conf_6	-871.305566	-870.990985	0.379771	-870.903279	0.070628	0.068677	-870.973908	-870.971956
B'_Et_conf_1	-1028.430355	-1028.044313	0.495686	-1027.906009	0.083116	0.080867	-1027.989125	-1027.986876
B'_Et_conf_10	-1028.427753	-1028.042335	0.496137	-1027.903167	0.082014	0.080273	-1027.985181	-1027.983439
B'_Et_conf_11	-1028.427124	-1028.040404	0.495625	-1027.902619	0.083646	0.081503	-1027.986265	-1027.984123
B'_Et_conf_12	-1028.428904	-1028.044226	0.495975	-1027.904270	0.082649	0.080899	-1027.986920	-1027.985169
B'_Et_conf_13	-1028.428606	-1028.043435	0.496081	-1027.903795	0.082917	0.081077	-1027.986711	-1027.984871
B'_Et_conf_14	-1028.426864	-1028.041969	0.496560	-1027.901886	0.082041	0.080222	-1027.983927	-1027.982107
B'_Et_conf_15	-1028.425314	-1028.039848	0.495876	-1027.900793	0.082886	0.080818	-1027.983679	-1027.981611
B'_Et_conf_16	-1028.425310	-1028.041864	0.496911	-1027.900207	0.081070	0.079637	-1027.981278	-1027.979844
B'_Et_conf_17	-1028.426631	-1028.039989	0.496130	-1027.901996	0.082826	0.080701	-1027.984822	-1027.982697
B'_Et_conf_18	-1028.427935	-1028.043589	0.496099	-1027.903416	0.081728	0.080165	-1027.985144	-1027.983581
B'_Et_conf_2	-1028.429938	-1028.044534	0.496109	-1027.905440	0.081642	0.080120	-1027.987082	-1027.985560

B'_Et_conf_20	-1028.425540	-1028.041569	0.496906	-1027.900435	0.081130	0.079666	-1027.981566	-1027.980101
B'_Et_conf_21	-1028.426666	-1028.042374	0.496261	-1027.901957	0.081932	0.080285	-1027.983889	-1027.982242
B'_Et_conf_22	-1028.425041	-1028.040172	0.496461	-1027.900022	0.082938	0.080681	-1027.982960	-1027.980703
B'_Et_conf_23	-1028.424296	-1028.038415	0.495928	-1027.899497	0.084153	0.081488	-1027.983649	-1027.980985
B'_Et_conf_25	-1028.426204	-1028.040311	0.496119	-1027.901567	0.082113	0.080444	-1027.983680	-1027.982011
B'_Et_conf_27	-1028.422476	-1028.036199	0.495856	-1027.897705	0.083986	0.081557	-1027.981691	-1027.979261
B'_Et_conf_3	-1028.430943	-1028.046112	0.496408	-1027.906227	0.081294	0.079888	-1027.987521	-1027.986114
B'_Et_conf_4	-1028.427475	-1028.041236	0.496337	-1027.902802	0.081999	0.080047	-1027.984801	-1027.982849
B'_Et_conf_5	-1028.425336	-1028.041302	0.496651	-1027.900329	0.081787	0.080116	-1027.982116	-1027.980445
B'_Et_conf_6	-1028.426639	-1028.041140	0.496415	-1027.901683	0.082466	0.080565	-1027.984149	-1027.982248
B'_Et_conf_9	-1028.427152	-1028.041097	0.496064	-1027.902480	0.082699	0.080736	-1027.985179	-1027.983215
B'_Me_conf_1	-871.302958	-870.990160	0.379762	-870.900402	0.071339	0.069705	-870.971740	-870.970107
B'_Me_conf_2	-871.305258	-870.993058	0.379735	-870.902801	0.073032	0.069998	-870.975833	-870.972800
B'_Me_conf_3	-871.305280	-870.992872	0.379805	-870.902825	0.071168	0.069183	-870.973993	-870.972009
C_Et_conf_1	-1028.416687	-1028.041773	0.493999	-1027.892309	0.090772	0.085653	-1027.983082	-1027.977962
C_Et_conf_10	-1028.414224	-1028.039753	0.495168	-1027.888952	0.090302	0.085280	-1027.979254	-1027.974232
C_Et_conf_11	-1028.414198	-1028.040070	0.495138	-1027.889041	0.089470	0.084897	-1027.978511	-1027.973938
C_Et_conf_12	-1028.413658	-1028.039958	0.495430	-1027.888216	0.089481	0.084927	-1027.977697	-1027.973143
C_Et_conf_2	-1028.410218	-1028.035496	0.494611	-1027.885703	0.089421	0.084562	-1027.975124	-1027.970265
C_Et_conf_3	-1028.416679	-1028.041492	0.494755	-1027.892006	0.088796	0.084518	-1027.980802	-1027.976523
C_Et_conf_4	-1028.414003	-1028.039647	0.494776	-1027.888734	0.091649	0.086294	-1027.980383	-1027.975028
C_Et_conf_5	-1028.416697	-1028.041452	0.496959	-1027.890262	0.087563	0.083504	-1027.977825	-1027.973766
C_Et_conf_6	-1028.414546	-1028.039877	0.494597	-1027.889493	0.091343	0.086256	-1027.980836	-1027.975749
C_Et_conf_7	-1028.413056	-1028.038510	0.495169	-1027.887733	0.090287	0.085274	-1027.978020	-1027.973007
C_Et_conf_8	-1028.414000	-1028.039848	0.495025	-1027.888832	0.090078	0.085327	-1027.978909	-1027.974158
C_Et_conf_9	-1028.412617	-1028.038993	0.494388	-1027.887849	0.090700	0.085715	-1027.978549	-1027.973564

C_Me_conf_1	-871.284138	-870.979903	0.378025	-870.881949	0.076786	0.073302	-870.958735	-870.955251
C_Me_conf_2	-871.282243	-870.979627	0.378628	-870.879734	0.075839	0.072472	-870.955573	-870.952205
D_Et_conf_1	-845.773368	-845.402566	0.538938	-845.204888	0.082797	0.081031	-845.287686	-845.285920
D_Et_conf_10	-845.771516	-845.399292	0.538630	-845.202909	0.084935	0.082329	-845.287844	-845.285238
D_Et_conf_11	-845.774315	-845.403854	0.539818	-845.205137	0.081993	0.080632	-845.287130	-845.285770
D_Et_conf_12	-845.772510	-845.400985	0.538922	-845.203753	0.084049	0.081820	-845.287802	-845.285573
D_Et_conf_13	-845.774539	-845.404029	0.539393	-845.205586	0.082520	0.081194	-845.288107	-845.286780
D_Et_conf_14	-845.771988	-845.401527	0.539284	-845.203116	0.082928	0.081216	-845.286044	-845.284331
D_Et_conf_15	-845.772985	-845.402489	0.539710	-845.203963	0.081724	0.080465	-845.285687	-845.284428
D_Et_conf_16	-845.768666	-845.398913	0.539738	-845.199579	0.081975	0.080538	-845.281554	-845.280116
D_Et_conf_17	-845.771423	-845.400702	0.539101	-845.202616	0.083940	0.081507	-845.286556	-845.284124
D_Et_conf_18	-845.769082	-845.398420	0.539157	-845.200168	0.083658	0.081585	-845.283826	-845.281753
D_Et_conf_19	-845.769591	-845.398316	0.539247	-845.200627	0.083573	0.081518	-845.284200	-845.282145
D_Et_conf_2	-845.763481	-845.394629	0.540093	-845.193912	0.082860	0.080913	-845.276771	-845.274824
D_Et_conf_20	-845.772608	-845.401573	0.539035	-845.203765	0.083986	0.081871	-845.287751	-845.285636
D_Et_conf_3	-845.770571	-845.399264	0.538932	-845.202015	0.083316	0.081220	-845.285331	-845.283235
D_Et_conf_4	-845.772475	-845.400710	0.538861	-845.203833	0.084098	0.081769	-845.287931	-845.285602
D_Et_conf_5	-845.771762	-845.400281	0.538722	-845.203309	0.083726	0.081494	-845.287034	-845.284802
D_Et_conf_6	-845.765885	-845.396181	0.539637	-845.196904	0.081878	0.080441	-845.278782	-845.277345
D_Et_conf_7	-845.770340	-845.399302	0.538936	-845.201730	0.083256	0.081371	-845.284985	-845.283100
D_Et_conf_8	-845.771390	-845.400256	0.538952	-845.202531	0.084710	0.082117	-845.287242	-845.284648
D_Et_conf_9	-845.766543	-845.396112	0.540074	-845.197066	0.082025	0.080770	-845.279091	-845.277836
D_Me_conf_2	-688.651640	-688.349686	0.422470	-688.205236	0.072327	0.070264	-688.277563	-688.275500
D_Me_conf_3	-688.649857	-688.348337	0.422648	-688.203433	0.071859	0.069891	-688.275292	-688.273324
D_Me_conf_4	-688.649807	-688.348188	0.422116	-688.203443	0.073629	0.071181	-688.277072	-688.274624
D'_Et_conf_1	-845.771847	-845.401665	0.538947	-845.203436	0.082396	0.080719	-845.285832	-845.284155

D'_Et_conf_10	-845.769437	-845.399447	0.538846	-845.200713	0.084527	0.081972	-845.285240	-845.282685
D'_Et_conf_11	-845.776198	-845.404035	0.540874	-845.206542	0.081178	0.079333	-845.287719	-845.285875
D'_Et_conf_12	-845.780067	-845.408702	0.539586	-845.211272	0.081284	0.080123	-845.292556	-845.291395
D'_Et_conf_13	-845.770767	-845.400486	0.538410	-845.202396	0.084501	0.081979	-845.286897	-845.284374
D'_Et_conf_14	-845.768788	-845.398421	0.538796	-845.200129	0.084876	0.082201	-845.285005	-845.282330
D'_Et_conf_15	-845.780562	-845.408364	0.538819	-845.211898	0.084243	0.081857	-845.296141	-845.293755
D'_Et_conf_2	-845.778738	-845.406687	0.539203	-845.209982	0.083105	0.081132	-845.293087	-845.291114
D'_Et_conf_3	-845.779728	-845.408654	0.539578	-845.210941	0.081461	0.080180	-845.292401	-845.291121
D'_Et_conf_4	-845.770329	-845.400308	0.539008	-845.201724	0.082840	0.081078	-845.284564	-845.282803
D'_Et_conf_5	-845.766053	-845.397292	0.539486	-845.197045	0.082488	0.080994	-845.279533	-845.278038
D'_Et_conf_6	-845.770356	-845.400530	0.539743	-845.201268	0.082064	0.080455	-845.283332	-845.281723
D'_Et_conf_7	-845.779678	-845.408602	0.539793	-845.210763	0.081087	0.079897	-845.291850	-845.290660
D'_Et_conf_8	-845.780257	-845.408211	0.539115	-845.211614	0.082539	0.080997	-845.294153	-845.292611
D'_Et_conf_9	-845.773515	-845.402392	0.539382	-845.204451	0.083224	0.081330	-845.287675	-845.285781
D'_Me_conf_1	-688.654180	-688.352813	0.422672	-688.207713	0.072026	0.069945	-688.279740	-688.277659
D'_Me_conf_2	-688.658094	-688.356748	0.423751	-688.211312	0.068711	0.067767	-688.280023	-688.279078
E_Et_conf_1	-392.811474	-392.628615	0.278955	-392.515306	0.059392	0.057699	-392.574698	-392.573005
E_Et_conf_2	-392.809302	-392.626336	0.279160	-392.513003	0.058821	0.057465	-392.571824	-392.570469
E_Et_conf_3	-392.805558	-392.622922	0.279364	-392.509016	0.060165	0.057721	-392.569180	-392.566737
E_Et_conf_4	-392.809445	-392.626998	0.279019	-392.513295	0.059091	0.057454	-392.572386	-392.570750
E_Et_conf_5	-392.809372	-392.627218	0.279326	-392.513226	0.057316	0.056562	-392.570542	-392.569787
E_Et_conf_6	-392.808634	-392.626147	0.279306	-392.512375	0.058305	0.057013	-392.570680	-392.569388
E_Et_conf_7	-392.808626	-392.626147	0.279308	-392.512374	0.058271	0.056988	-392.570645	-392.569362
E_Et_conf_8	-392.809542	-392.626734	0.279198	-392.513316	0.058430	0.057179	-392.571746	-392.570494
E_Et_conf_9	-392.809542	-392.626734	0.279195	-392.513318	0.058438	0.057183	-392.571756	-392.570501
E_Me_conf_1	-235.672732	-235.562998	0.163905	-235.497562	0.044117	0.043825	-235.541679	-235.541387

F_conf_1	-453.003118	-452.807818	0.254114	-452.734629	0.053207	0.051834	-452.787836	-452.786463	
F_conf_2	-453.002785	-452.807960	0.254287	-452.734426	0.051644	0.051007	-452.786070	-452.785433	
G_conf_1	-114.974200	-114.916098	0.036567	-114.933094	0.027174	0.027174	-114.960268	-114.960268	
HI	-298.276945	-298.224903	0.005338	-298.267914	0.023106	0.023106	-298.291020	-298.291020	
HI_cluster_conf_1	-645.279600	-645.060344	0.170381	-645.092155	0.066049	0.061530	-645.158204	-645.153684	
HI_cluster_conf_2	-645.279708	-645.060600	0.170480	-645.092294	0.065004	0.060942	-645.157298	-645.153235	
HI_cluster_conf_3	-645.279710	-645.060601	0.170480	-645.092296	0.065062	0.060974	-645.157358	-645.153270	
HI_cluster_conf_4	-645.279549	-645.060769	0.170880	-645.092073	0.062751	0.059792	-645.154824	-645.151865	
HI_cluster_conf_5	-645.279713	-645.060600	0.170480	-645.092298	0.065076	0.060982	-645.157374	-645.153280	
HI_cluster_conf_6	-645.279363	-645.060360	0.170621	-645.091878	0.064316	0.060573	-645.156194	-645.152451	
HI_cluster_conf_7	-645.263563	-645.045439	0.170774	-645.075893	0.062745	0.060267	-645.138637	-645.136160	
HI_cluster_conf_8	-645.245381	-645.024004	0.168701	-645.058361	0.068002	0.063786	-645.126363	-645.122147	
HI_cluster_conf_9	-645.279709	-645.060601	0.170481	-645.092295	0.065014	0.060947	-645.157309	-645.153242	
H_conf_1	-337.811729	-337.665759	0.208651	-337.592313	0.044249	0.044248	-337.636562	-337.636561	
H_conf_2	-337.812224	-337.665738	0.207944	-337.592845	0.047233	0.046143	-337.640077	-337.638988	
I2	-595.388701	-595.280286	0.000522	-595.383869	0.029999	0.030000	-595.413868	-595.413869	
I_anion	-297.848431	-297.806502	0.000000	-297.845794	0.018266	0.018266	-297.864060	-297.864060	
I_anion_MeOH4	-760.474460	-760.202544	0.210822	-760.239729	0.083206	0.076483	-760.322935	-760.316212	
I_conf_1	-635.620796	-635.418997	0.211205	-635.396904	0.050748	0.050102	-635.447652	-635.447007	
I_conf_2	-635.620518	-635.418621	0.210901	-635.396525	0.053596	0.051535	-635.450120	-635.448059	
J_conf_1	-297.651037	-297.613749	0.000000	-297.648399	0.018997	0.018997	-297.667397	-297.667397	
MeOH	-115.646634	-115.590008	0.051267	-115.590435	0.027438	0.027440	-115.617872	-115.617874	
MeOH_cluster	-462.626492	-462.401934	0.212651	-462.393607	0.074348	0.067215	-462.467956	-462.460822	
TS-I-Anti_Et_conf_1	-1028.414140	-1028.032797	0.495188	-1027.890557	0.082654	0.080604	-1027.973211	-1027.971161	-105.91
TS-I-Anti_Et_conf_10	-1028.408567	-1028.028850	0.496080	-1027.884556	0.081478	0.079454	-1027.966034	-1027.964010	-85.91
TS-I-Anti_Et_conf_11	-1028.408888	-1028.029391	0.495058	-1027.885573	0.082587	0.080242	-1027.968160	-1027.965815	-96.71

TS-I-Anti_Et_conf_12	-1028.408498	-1028.029804	0.495340	-1027.885027	0.082147	0.079832	-1027.967174	-1027.964859	-90.38
TS-I-Anti_Et_conf_13	-1028.409444	-1028.028996	0.494222	-1027.886670	0.083726	0.080928	-1027.970396	-1027.967598	-115.07
TS-I-Anti_Et_conf_14	-1028.409149	-1028.029458	0.495566	-1027.885309	0.082746	0.080224	-1027.968055	-1027.965533	-97.77
TS-I-Anti_Et_conf_15	-1028.407651	-1028.029965	0.495981	-1027.883157	0.084174	0.081112	-1027.967331	-1027.964269	-58.96
TS-I-Anti_Et_conf_16	-1028.410460	-1028.029659	0.494961	-1027.887169	0.082616	0.080373	-1027.969785	-1027.967542	-118.39
TS-I-Anti_Et_conf_2	-1028.415276	-1028.035072	0.494878	-1027.891991	0.082619	0.080668	-1027.974611	-1027.972659	-97.70
TS-I-Anti_Et_conf_3	-1028.414487	-1028.035406	0.495578	-1027.890785	0.081672	0.079782	-1027.972458	-1027.970567	-95.51
TS-I-Anti_Et_conf_4	-1028.413703	-1028.033940	0.494537	-1027.890674	0.082967	0.080779	-1027.973642	-1027.971453	-90.18
TS-I-Anti_Et_conf_5	-1028.413105	-1028.032619	0.495097	-1027.889827	0.081886	0.079966	-1027.971713	-1027.969793	-93.26
TS-I-Anti_Et_conf_6	-1028.412557	-1028.031079	0.494490	-1027.889385	0.084197	0.081379	-1027.973582	-1027.970764	-95.51
TS-I-Anti_Et_conf_7	-1028.415456	-1028.035725	0.495473	-1027.892072	0.080908	0.079209	-1027.972980	-1027.971282	-96.31
TS-I-Anti_Et_conf_8	-1028.413156	-1028.032877	0.495387	-1027.889345	0.083509	0.080756	-1027.972854	-1027.970101	-90.74
TS-I-Anti_Et_conf_9	-1028.409994	-1028.029488	0.495849	-1027.885821	0.083037	0.080472	-1027.968858	-1027.966292	-109.26
TS-I-Anti_Me_conf_1	-871.283706	-870.977362	0.378312	-870.882424	0.073119	0.070516	-870.955543	-870.952939	-71.66
TS-I-Anti_Me_conf_2	-871.283089	-870.976718	0.378800	-870.881769	0.071184	0.069259	-870.952953	-870.951028	-95.76
TS-I-Gauche_Et_conf_1	-1028.400102	-1028.018385	0.493791	-1027.877889	0.082262	0.080331	-1027.960151	-1027.958220	-100.64
TS-I-Gauche_Et_conf_10	-1028.395512	-1028.018215	0.493548	-1027.872855	0.086107	0.082532	-1027.958962	-1027.955387	-33.32
TS-I-Gauche_Et_conf_11	-1028.399533	-1028.020435	0.493683	-1027.877164	0.084721	0.081508	-1027.961885	-1027.958672	-36.83
TS-I-Gauche_Et_conf_12	-1028.396424	-1028.019565	0.493658	-1027.873857	0.084935	0.081926	-1027.958792	-1027.955783	-56.84
TS-I-Gauche_Et_conf_13	-1028.401743	-1028.022636	0.494833	-1027.878596	0.082081	0.080273	-1027.960677	-1027.958869	-77.76
TS-I-Gauche_Et_conf_14	-1028.396627	-1028.020535	0.494272	-1027.873668	0.083936	0.081282	-1027.957604	-1027.954951	-28.61
TS-I-Gauche_Et_conf_15	-1028.400473	-1028.024440	0.494817	-1027.877180	0.083420	0.080800	-1027.960599	-1027.957979	-25.33
TS-I-Gauche_Et_conf_16	-1028.404327	-1028.023462	0.494691	-1027.881218	0.083104	0.080561	-1027.964323	-1027.961779	-62.34
TS-I-Gauche_Et_conf_17	-1028.397839	-1028.020807	0.493461	-1027.875196	0.086276	0.082632	-1027.961471	-1027.957828	-31.08
TS-I-Gauche_Et_conf_2	-1028.404232	-1028.024121	0.493980	-1027.881510	0.083759	0.081455	-1027.965269	-1027.962966	-65.62
TS-I-Gauche_Et_conf_3	-1028.391748	-1028.015805	0.493869	-1027.868988	0.084788	0.081828	-1027.953776	-1027.950816	-16.38

TS-I-Gauche_Et_conf_4	-1028.396998	-1028.016782	0.495514	-1027.873281	0.082184	0.079983	-1027.955465	-1027.953264	-89.52
TS-I-Gauche_Et_conf_5	-1028.392545	-1028.016184	0.493028	-1027.870234	0.087163	0.083118	-1027.957397	-1027.953352	-35.27
TS-I-Gauche_Et_conf_6	-1028.399297	-1028.021833	0.494675	-1027.876003	0.084289	0.081193	-1027.960292	-1027.957196	-30.87
TS-I-Gauche_Et_conf_7	-1028.407038	-1028.026960	0.494184	-1027.884284	0.083515	0.080945	-1027.967798	-1027.965229	-38.31
TS-I-Gauche_Et_conf_8	-1028.393602	-1028.016782	0.494434	-1027.870250	0.085088	0.082008	-1027.955338	-1027.952258	-48.80
TS-I-Gauche_Et_conf_9	-1028.404306	-1028.027706	0.494605	-1027.881256	0.083540	0.080749	-1027.964796	-1027.962004	-21.50
TS-I-Gauche_Me_conf_1	-871.270632	-870.966166	0.377919	-870.869945	0.072961	0.070142	-870.942906	-870.940086	-58.90
TS-I-Gauche_Me_conf_2	-871.276044	-870.967822	0.379317	-870.874713	0.069388	0.067836	-870.944102	-870.942549	-30.74
TS-III_Et_conf_1	-845.758106	-845.384064	0.536792	-845.191644	0.083285	0.081586	-845.274929	-845.273230	-459.65
TS-III_Et_conf_10	-845.754355	-845.381931	0.537835	-845.187432	0.081482	0.080054	-845.268914	-845.267487	-435.18
TS-III_Et_conf_11	-845.752630	-845.379059	0.536946	-845.186291	0.082300	0.080815	-845.268591	-845.267106	-434.32
TS-III_Et_conf_12	-845.753957	-845.381704	0.536982	-845.187518	0.082768	0.081004	-845.270286	-845.268521	-463.96
TS-III_Et_conf_13	-845.749593	-845.377660	0.537500	-845.183045	0.081130	0.079848	-845.264175	-845.262894	-431.97
TS-III_Et_conf_14	-845.751278	-845.378455	0.537687	-845.184654	0.080651	0.079541	-845.265305	-845.264194	-429.97
TS-III_Et_conf_15	-845.749540	-845.376512	0.537623	-845.182469	0.083414	0.081124	-845.265883	-845.263593	-439.04
TS-III_Et_conf_16	-845.755435	-845.382581	0.537580	-845.188687	0.081357	0.080253	-845.270043	-845.268940	-444.81
TS-III_Et_conf_17	-845.749328	-845.375906	0.537532	-845.182570	0.081970	0.080438	-845.264540	-845.263008	-441.94
TS-III_Et_conf_18	-845.751100	-845.377820	0.537708	-845.184310	0.081307	0.079979	-845.265618	-845.264289	-409.93
TS-III_Et_conf_19	-845.748240	-845.374792	0.537759	-845.181194	0.082277	0.080554	-845.263472	-845.261749	-461.32
TS-III_Et_conf_2	-845.758846	-845.385082	0.536670	-845.192720	0.082393	0.080967	-845.275113	-845.273688	-444.45
TS-III_Et_conf_20	-845.753550	-845.380416	0.537357	-845.186917	0.081838	0.080447	-845.268755	-845.267364	-438.42
TS-III_Et_conf_21	-845.750430	-845.377772	0.537858	-845.183340	0.082059	0.080403	-845.265399	-845.263743	-456.80
TS-III_Et_conf_22	-845.747429	-845.373623	0.537615	-845.180544	0.082312	0.080433	-845.262856	-845.260977	-404.85
TS-III_Et_conf_23	-845.754643	-845.381645	0.537119	-845.188151	0.081863	0.080721	-845.270014	-845.268872	-447.75
TS-III_Et_conf_24	-845.749334	-845.375934	0.537608	-845.182341	0.082350	0.080723	-845.264691	-845.263065	-455.27
TS-III_Et_conf_25	-845.751014	-845.378270	0.537250	-845.184252	0.083160	0.081163	-845.267413	-845.265415	-440.04

TS-III_Et_conf_26	-845.753460	-845.380663	0.537514	-845.186847	0.081246	0.080023	-845.268093	-845.266869	-424.38
TS-III_Et_conf_27	-845.748881	-845.376654	0.537581	-845.181992	0.082650	0.080606	-845.264642	-845.262598	-462.61
TS-III_Et_conf_28	-845.749842	-845.377068	0.537429	-845.183060	0.082978	0.080762	-845.266039	-845.263822	-432.43
TS-III_Et_conf_29	-845.750972	-845.378349	0.537572	-845.184248	0.081970	0.080249	-845.266219	-845.264497	-475.85
TS-III_Et_conf_3	-845.749664	-845.377398	0.537687	-845.182807	0.081085	0.080180	-845.263892	-845.262987	-453.31
TS-III_Et_conf_30	-845.749160	-845.376974	0.537995	-845.182284	0.080616	0.079453	-845.262900	-845.261736	-425.65
TS-III_Et_conf_4	-845.758538	-845.385325	0.536752	-845.192363	0.082397	0.080856	-845.274760	-845.273219	-451.32
TS-III_Et_conf_5	-845.749152	-845.376854	0.537394	-845.182546	0.082065	0.080292	-845.264611	-845.262838	-444.95
TS-III_Et_conf_6	-845.755862	-845.382866	0.537001	-845.189481	0.082043	0.080698	-845.271524	-845.270179	-452.67
TS-III_Et_conf_7	-845.748110	-845.375250	0.537873	-845.180932	0.082492	0.080529	-845.263424	-845.261461	-479.67
TS-III_Et_conf_8	-845.751981	-845.379556	0.537263	-845.185484	0.081685	0.080299	-845.267169	-845.265783	-428.32
TS-III_Et_conf_9	-845.754866	-845.380950	0.537448	-845.188107	0.082088	0.080596	-845.270194	-845.268703	-444.95
TS-III_Me_conf_1	-688.625345	-688.323264	0.421834	-688.180563	0.068199	0.067650	-688.248762	-688.248213	-456.72
TS-III_Me_conf_2	-688.626680	-688.325537	0.421264	-688.182302	0.068886	0.068025	-688.251188	-688.250327	-471.95
TS-II_Et_conf_1	-1028.411973	-1028.027935	0.496205	-1027.888142	0.080231	0.078790	-1027.968373	-1027.966932	-44.01
TS-II_Et_conf_10	-1028.409214	-1028.023685	0.496194	-1027.885457	0.080144	0.078564	-1027.965601	-1027.964022	-48.66
TS-II_Et_conf_11	-1028.408996	-1028.024507	0.495656	-1027.885448	0.081019	0.079429	-1027.966467	-1027.964877	-54.50
TS-II_Et_conf_12	-1028.408042	-1028.022085	0.496122	-1027.884342	0.080054	0.078629	-1027.964396	-1027.962971	-36.22
TS-II_Et_conf_13	-1028.408383	-1028.022585	0.495923	-1027.884889	0.079963	0.078528	-1027.964853	-1027.963417	-37.84
TS-II_Et_conf_14	-1028.412048	-1028.026371	0.496185	-1027.888292	0.079980	0.078601	-1027.968272	-1027.966893	-31.80
TS-II_Et_conf_15	-1028.402949	-1028.017394	0.496317	-1027.879030	0.080265	0.078731	-1027.959296	-1027.957762	-37.32
TS-II_Et_conf_16	-1028.409092	-1028.024405	0.495873	-1027.885415	0.080914	0.079199	-1027.966329	-1027.964614	-39.18
TS-II_Et_conf_17	-1028.407989	-1028.024083	0.496280	-1027.884045	0.080296	0.078872	-1027.964341	-1027.962917	-61.28
TS-II_Et_conf_18	-1028.405571	-1028.020129	0.496206	-1027.881752	0.080338	0.078719	-1027.962090	-1027.960471	-50.39
TS-II_Et_conf_19	-1028.406146	-1028.020112	0.495857	-1027.882617	0.080606	0.078821	-1027.963224	-1027.961438	-52.02
TS-II_Et_conf_2	-1028.412642	-1028.028302	0.496164	-1027.888993	0.079363	0.078269	-1027.968356	-1027.967262	-53.47

TS-II_Et_conf_20	-1028.409473	-1028.023159	0.495532	-1027.885957	0.081506	0.079515	-1027.967463	-1027.965472	-54.38
TS-II_Et_conf_21	-1028.408184	-1028.023458	0.496138	-1027.884242	0.081220	0.079231	-1027.965462	-1027.963473	-66.02
TS-II_Et_conf_22	-1028.404682	-1028.021659	0.496680	-1027.880630	0.079649	0.077996	-1027.960279	-1027.958626	-36.52
TS-II_Et_conf_3	-1028.413101	-1028.027471	0.495663	-1027.889580	0.080893	0.079312	-1027.970473	-1027.968891	-44.19
TS-II_Et_conf_4	-1028.412680	-1028.026195	0.495007	-1027.889519	0.082132	0.080023	-1027.971651	-1027.969542	-42.67
TS-II_Et_conf_5	-1028.402012	-1028.017119	0.496496	-1027.877740	0.081047	0.079132	-1027.958787	-1027.956872	-70.83
TS-II_Et_conf_6	-1028.404079	-1028.018395	0.496033	-1027.880312	0.080760	0.079132	-1027.961072	-1027.959444	-39.86
TS-II_Et_conf_7	-1028.404590	-1028.018688	0.495927	-1027.881107	0.079790	0.078517	-1027.960896	-1027.959624	-49.06
TS-II_Et_conf_8	-1028.411253	-1028.027436	0.496202	-1027.887387	0.080416	0.078834	-1027.967803	-1027.966221	-57.90
TS-II_Et_conf_9	-1028.412137	-1028.026663	0.495944	-1027.888618	0.079589	0.078482	-1027.968208	-1027.967101	-42.10
TS-II_Me_conf_1	-871.288913	-870.976351	0.380327	-870.887313	0.067518	0.066282	-870.954831	-870.953595	-59.74
TS-II_Me_conf_2	-871.287075	-870.974936	0.380653	-870.885350	0.066936	0.065797	-870.952285	-870.951147	-56.41
TS-II_Me_conf_3	-871.287270	-870.974975	0.379646	-870.886192	0.067884	0.066585	-870.954076	-870.952778	-75.57
B_b_Me_conf_1	-946.535464	-946.194392	0.386605	-946.125666	0.072463	0.070191	-946.198129	-946.195857	
B_b_Me_conf_2	-946.533163	-946.192619	0.386213	-946.123484	0.072912	0.070755	-946.196396	-946.194239	
B_b_Me_conf_3	-946.530426	-946.189609	0.386178	-946.120642	0.073447	0.071116	-946.194089	-946.191758	
B_b_Me_conf_4	-946.532565	-946.192312	0.386146	-946.122901	0.073557	0.071098	-946.196458	-946.193999	
B_b_Me_conf_5	-946.532376	-946.191156	0.386495	-946.122370	0.073054	0.070825	-946.195425	-946.193196	
B_b_Me_conf_6	-946.535860	-946.195009	0.386412	-946.126160	0.072367	0.070265	-946.198528	-946.196425	
B'_b_Me_conf_1	-946.534459	-946.195195	0.385963	-946.124738	0.074003	0.071698	-946.198742	-946.196436	
B'_b_Me_conf_2	-946.535350	-946.197009	0.386573	-946.125489	0.072251	0.070370	-946.197740	-946.195859	
B'_b_Me_conf_3	-946.531465	-946.192421	0.386580	-946.121391	0.073649	0.071123	-946.195040	-946.192513	
B'_b_Me_conf_4	-946.531811	-946.192504	0.386260	-946.121853	0.074346	0.071674	-946.196199	-946.193526	
TS-I-Anti_b_Me_conf_1	-946.513290	-946.179353	0.385107	-946.104559	0.074181	0.071720	-946.178740	-946.176279	-113.22
TS-I-Anti_b_Me_conf_2	-946.517334	-946.184225	0.384970	-946.108802	0.074171	0.071590	-946.182973	-946.180392	-126.41
TS-I-Anti_b_Me_conf_3	-946.516546	-946.183466	0.385066	-946.107868	0.074397	0.071841	-946.182266	-946.179710	-103.30

TS-I-Gauche_b_Me_conf_1	-946.508750	-946.173976	0.385381	-946.100204	0.072223	0.070245	-946.172426	-946.170449	-25.28
TS-I-Gauche_b_Me_conf_2	-946.504377	-946.173677	0.384583	-946.096196	0.074508	0.071713	-946.170704	-946.167909	-36.23
TS-I-Gauche_b_Me_conf_3	-946.509174	-946.174913	0.385262	-946.100840	0.072343	0.070082	-946.173183	-946.170922	-48.16
TS-I-Gauche_b_Me_conf_4	-946.507780	-946.175538	0.384913	-946.099459	0.073311	0.070977	-946.172770	-946.170436	-40.69

Table S9. Compiled thermochemical data for structures computed at the M06-2X-D3/def2-TZVPD,SMD(THF)//M06-2X-D3/def2-SVPD;def2-TZVPD[I;Se],SMD(THF) level of theory; Gibbs free energies are reported at 298.15 K.

Structure	E_{SPC}	E	ZPE	H_{SPC}	T.S	T.qh-S	$G(T)_{SPC}$	qh-$G(T)_{SPC}$	ν_{imag} / cm^{-1}
B_Me_b_conf_1	-946.876768	-946.194392	0.386605	-946.471358	0.060810	0.058956	-946.532168	-946.530314	
B_Me_b_conf_2	-946.874862	-946.192619	0.386213	-946.469586	0.061198	0.059455	-946.530784	-946.529040	
B_Me_b_conf_3	-946.877453	-946.195009	0.386412	-946.472149	0.060718	0.059018	-946.532867	-946.531167	
B_Me_b_conf_4	-946.874541	-946.192312	0.386146	-946.469280	0.061774	0.059762	-946.531054	-946.529042	
B_Me_b_conf_5	-946.873597	-946.191156	0.386495	-946.467992	0.061328	0.059536	-946.529319	-946.527527	
B_Me_b_conf_6	-946.871925	-946.189609	0.386178	-946.466546	0.061674	0.059796	-946.528220	-946.526342	
B’_Me_b_conf_1	-946.874380	-946.192504	0.386260	-946.468830	0.062477	0.060297	-946.531306	-946.529126	
B’_Me_b_conf_2	-946.878733	-946.197009	0.386573	-946.473267	0.060614	0.059108	-946.533881	-946.532375	
B’_Me_b_conf_3	-946.874390	-946.192421	0.386580	-946.468710	0.061865	0.059809	-946.530575	-946.528519	
B’_Me_b_conf_4	-946.877203	-946.195195	0.385963	-946.471900	0.062160	0.060306	-946.534061	-946.532206	
E_Me_conf_1	-235.818030	-235.562998	0.163905	-235.644705	0.037844	0.037643	-235.682549	-235.682348	
F_b_conf_1	-528.569603	-528.005206	0.260760	-528.296484	0.046207	0.045452	-528.342691	-528.341936	
F_b_conf_2	-528.572884	-528.008530	0.261024	-528.299796	0.045152	0.044680	-528.344948	-528.344476	
G_b_conf_1	-1062.094647	-1061.287106	0.428517	-1061.645035	0.064020	0.062872	-1061.709055	-1061.707908	
G_b_conf_2	-1062.109267	-1061.301943	0.427852	-1061.660340	0.063925	0.062669	-1061.724265	-1061.723009	
G_b_conf_3	-1062.096166	-1061.288408	0.428215	-1061.646975	0.063591	0.062498	-1061.710566	-1061.709473	
G_b_conf_4	-1062.097852	-1061.290122	0.427646	-1061.648837	0.065065	0.063493	-1061.713902	-1061.712330	

G_b_conf_5	-1062.097928	-1061.290537	0.427552	-1061.649001	0.064612	0.063381	-1061.713613	-1061.712382
G_b_conf_6	-1062.098554	-1061.290950	0.427735	-1061.649517	0.064722	0.063292	-1061.714239	-1061.712810
G_b_conf_7	-1062.101763	-1061.294148	0.427358	-1061.652985	0.065092	0.063569	-1061.718077	-1061.716554
H_b_conf_1	-3397.695916	-3396.645961	0.518824	-3397.150423	0.077076	0.073866	-3397.227499	-3397.224289
H_b_conf_10	-3397.694286	-3396.644529	0.518811	-3397.148875	0.076852	0.073796	-3397.225727	-3397.222671
H_b_conf_11	-3397.691345	-3396.641691	0.518710	-3397.145992	0.076963	0.073859	-3397.222955	-3397.219851
H_b_conf_12	-3397.692357	-3396.642638	0.519087	-3397.146728	0.077189	0.073692	-3397.223917	-3397.220420
H_b_conf_13	-3397.690734	-3396.641789	0.518619	-3397.145193	0.077850	0.074474	-3397.223043	-3397.219667
H_b_conf_14	-3397.693590	-3396.643884	0.518514	-3397.148251	0.077789	0.074314	-3397.226041	-3397.222566
H_b_conf_15	-3397.694399	-3396.644882	0.519349	-3397.148771	0.076284	0.072966	-3397.225055	-3397.221737
H_b_conf_16	-3397.691966	-3396.642272	0.518906	-3397.146572	0.076922	0.073451	-3397.223494	-3397.220023
H_b_conf_17	-3397.694407	-3396.644820	0.518810	-3397.148778	0.077639	0.074258	-3397.226418	-3397.223036
H_b_conf_2	-3397.692704	-3396.643053	0.519353	-3397.146955	0.076145	0.073169	-3397.223100	-3397.220124
H_b_conf_3	-3397.696430	-3396.646902	0.519444	-3397.150642	0.076532	0.073266	-3397.227174	-3397.223909
H_b_conf_4	-3397.695101	-3396.645374	0.519854	-3397.148955	0.076120	0.073008	-3397.225075	-3397.221963
H_b_conf_5	-3397.697328	-3396.647818	0.518909	-3397.151932	0.077701	0.073767	-3397.229633	-3397.225699
H_b_conf_6	-3397.692811	-3396.642965	0.518944	-3397.147288	0.076844	0.073661	-3397.224132	-3397.220949
H_b_conf_7	-3397.696181	-3396.646802	0.519162	-3397.150730	0.076049	0.072976	-3397.226780	-3397.223707
H_b_conf_8	-3397.695019	-3396.645783	0.519412	-3397.149330	0.075878	0.072998	-3397.225208	-3397.222328
H_b_conf_9	-3397.693374	-3396.644065	0.519094	-3397.147952	0.076453	0.073156	-3397.224406	-3397.221108
H'_b_conf_1	-3397.692902	-3396.643492	0.519945	-3397.146931	0.074971	0.072282	-3397.221902	-3397.219213
H'_b_conf_10	-3397.691813	-3396.642441	0.519127	-3397.146335	0.076699	0.073155	-3397.223034	-3397.219490
H'_b_conf_11	-3397.689649	-3396.640072	0.519043	-3397.144235	0.076302	0.073023	-3397.220537	-3397.217257
H'_b_conf_12	-3397.689440	-3396.639708	0.518685	-3397.144147	0.077082	0.073662	-3397.221229	-3397.217809
H'_b_conf_13	-3397.690258	-3396.640650	0.519516	-3397.144417	0.076152	0.072969	-3397.220569	-3397.217386
H'_b_conf_14	-3397.690700	-3396.642291	0.519557	-3397.144980	0.075593	0.072497	-3397.220573	-3397.217477

H'_b_conf_15	-3397.690450	-3396.640927	0.519657	-3397.144587	0.075427	0.072580	-3397.220015	-3397.217167	
H'_b_conf_16	-3397.693473	-3396.644300	0.519365	-3397.147837	0.076432	0.072896	-3397.224269	-3397.220733	
H'_b_conf_17	-3397.688461	-3396.640637	0.518790	-3397.143204	0.075419	0.072950	-3397.218624	-3397.216154	
H'_b_conf_18	-3397.692048	-3396.642740	0.519378	-3397.146423	0.076727	0.073032	-3397.223150	-3397.219455	
H'_b_conf_19	-3397.694168	-3396.644653	0.519131	-3397.148760	0.076156	0.072920	-3397.224916	-3397.221680	
H'_b_conf_2	-3397.696575	-3396.647147	0.519721	-3397.150707	0.075257	0.072491	-3397.225964	-3397.223199	
H'_b_conf_3	-3397.691534	-3396.641730	0.518690	-3397.146069	0.077616	0.074131	-3397.223685	-3397.220200	
H'_b_conf_4	-3397.695693	-3396.645832	0.518904	-3397.150237	0.076501	0.073456	-3397.226737	-3397.223692	
H'_b_conf_5	-3397.689317	-3396.639788	0.519098	-3397.143803	0.077705	0.073647	-3397.221508	-3397.217450	
H'_b_conf_6	-3397.694264	-3396.644794	0.519413	-3397.148319	0.076600	0.073474	-3397.224920	-3397.221793	
H'_b_conf_7	-3397.689659	-3396.640096	0.519072	-3397.144201	0.076311	0.073060	-3397.220512	-3397.217261	
H'_b_conf_8	-3397.692141	-3396.642708	0.519771	-3397.146308	0.075225	0.072239	-3397.221533	-3397.218547	
H'_b_conf_9	-3397.691561	-3396.641928	0.519239	-3397.145824	0.076529	0.073350	-3397.222353	-3397.219175	
PhSe_anion	-2633.348355	-2633.103034	0.090382	-2633.251325	0.034596	0.034588	-2633.285921	-2633.285913	
TS-IV-Syn_1	-1062.092389	-1061.284192	0.426792	-1061.644554	0.064011	0.062795	-1061.708564	-1061.707348	-169.99
TS-IV-Syn_2	-1062.099338	-1061.291217	0.426726	-1061.651574	0.064973	0.063050	-1061.716547	-1061.714624	-167.80
TS-IV-Syn_3	-1062.096765	-1061.288925	0.426392	-1061.649109	0.065021	0.063396	-1061.714130	-1061.712505	-161.58
TS-V-Rotation_b_conf_1	-1062.094593	-1061.286719	0.428172	-1061.646140	0.062313	0.061356	-1061.708453	-1061.707496	-70.77
TS-V-Rotation_b_conf_2	-1062.085392	-1061.277710	0.428223	-1061.636820	0.062303	0.061483	-1061.699123	-1061.698303	-60.16
TS-V-Rotation_b_conf_3	-1062.082954	-1061.275495	0.428803	-1061.633955	0.062319	0.061203	-1061.696274	-1061.695158	-68.09
TS-V-Rotation_b_conf_4	-1062.096683	-1061.288806	0.428092	-1061.648451	0.061710	0.061006	-1061.710161	-1061.709457	-47.33
TS-V-Rotation_b_conf_5	-1062.086681	-1061.278798	0.428013	-1061.638286	0.062814	0.061683	-1061.701101	-1061.699970	-60.01
TS-V-Rotation_b_conf_6	-1062.083202	-1061.275581	0.427746	-1061.634807	0.063414	0.062238	-1061.698220	-1061.697045	-81.59
TS-V-Rotation_b_conf_7	-1062.098738	-1061.291238	0.427725	-1061.650636	0.062496	0.061574	-1061.713132	-1061.712210	-59.24
TS-V-Rotation_b_conf_8	-1062.097560	-1061.289819	0.428162	-1061.649175	0.061949	0.061172	-1061.711124	-1061.710348	-51.90
TS-VI-Anti_b_conf_1	-3397.688801	-3396.638083	0.517869	-3397.144298	0.077622	0.074138	-3397.221921	-3397.218437	-237.40

TS-VI-Anti_b_conf_10	-3397.687850	-3396.637296	0.517428	-3397.143591	0.077824	0.074457	-3397.221415	-3397.218047	-245.17
TS-VI-Anti_b_conf_11	-3397.687193	-3396.636806	0.518380	-3397.142488	0.076268	0.073151	-3397.218757	-3397.215639	-225.89
TS-VI-Anti_b_conf_12	-3397.684904	-3396.634068	0.517905	-3397.140148	0.077999	0.074553	-3397.218147	-3397.214701	-226.88
TS-VI-Anti_b_conf_13	-3397.684878	-3396.634738	0.517361	-3397.140389	0.079061	0.075297	-3397.219449	-3397.215686	-258.99
TS-VI-Anti_b_conf_14	-3397.685233	-3396.634468	0.517735	-3397.140679	0.078285	0.074521	-3397.218964	-3397.215200	-208.73
TS-VI-Anti_b_conf_2	-3397.690062	-3396.639032	0.517578	-3397.145695	0.077393	0.074278	-3397.223087	-3397.219972	-254.25
TS-VI-Anti_b_conf_3	-3397.686266	-3396.635327	0.517961	-3397.141524	0.078835	0.074754	-3397.220358	-3397.216278	-213.46
TS-VI-Anti_b_conf_4	-3397.688066	-3396.637174	0.517185	-3397.143634	0.079885	0.075804	-3397.223518	-3397.219438	-248.77
TS-VI-Anti_b_conf_5	-3397.686011	-3396.635412	0.517846	-3397.141634	0.077466	0.073796	-3397.219100	-3397.215430	-217.73
TS-VI-Anti_b_conf_6	-3397.687494	-3396.636970	0.517888	-3397.143029	0.077492	0.074020	-3397.220522	-3397.217049	-218.63
TS-VI-Anti_b_conf_7	-3397.691093	-3396.640431	0.517729	-3397.146708	0.077862	0.074121	-3397.224571	-3397.220830	-234.35
TS-VI-Anti_b_conf_8	-3397.686316	-3396.635449	0.517548	-3397.141830	0.078383	0.074698	-3397.220212	-3397.216528	-216.46
TS-VI-Anti_b_conf_9	-3397.690239	-3396.639721	0.517720	-3397.145939	0.077486	0.073835	-3397.223425	-3397.219775	-236.65
TS-VI-Gauche_b_conf_1	-3397.656548	-3396.604602	0.517085	-3397.112384	0.079059	0.075052	-3397.191443	-3397.187436	-338.56
TS-VI-Gauche_b_conf_10	-3397.663338	-3396.612395	0.517760	-3397.118840	0.078410	0.074459	-3397.197250	-3397.193299	-357.29
TS-VI-Gauche_b_conf_2	-3397.664125	-3396.612994	0.517334	-3397.119849	0.078623	0.074682	-3397.198471	-3397.194531	-347.23
TS-VI-Gauche_b_conf_3	-3397.663315	-3396.612069	0.517687	-3397.118929	0.077869	0.074159	-3397.196798	-3397.193088	-363.22
TS-VI-Gauche_b_conf_4	-3397.659585	-3396.608477	0.517688	-3397.115053	0.078190	0.074383	-3397.193242	-3397.189436	-315.48
TS-VI-Gauche_b_conf_5	-3397.661332	-3396.610063	0.517912	-3397.116789	0.078016	0.073988	-3397.194805	-3397.190777	-303.47
TS-VI-Gauche_b_conf_6	-3397.667081	-3396.616022	0.517341	-3397.122624	0.078859	0.075068	-3397.201483	-3397.197692	-352.33
TS-VI-Gauche_b_conf_7	-3397.662626	-3396.611552	0.517132	-3397.118312	0.079297	0.075293	-3397.197609	-3397.193605	-337.88
TS-VI-Gauche_b_conf_8	-3397.668644	-3396.617561	0.517376	-3397.124378	0.078116	0.074470	-3397.202494	-3397.198848	-341.66
TS-VI-Gauche_b_conf_9	-3397.658282	-3396.606698	0.518071	-3397.113666	0.077390	0.073664	-3397.191056	-3397.187330	-357.78
TS-VII-Rotation_b_conf_1	-3397.679149	-3396.629522	0.519232	-3397.134415	0.074639	0.071546	-3397.209054	-3397.205961	-56.28
TS-VII-Rotation_b_conf_2	-3397.678168	-3396.628317	0.519379	-3397.133192	0.074933	0.071813	-3397.208124	-3397.205005	-58.24
TS-VII-Rotation_b_conf_3	-3397.673405	-3396.623581	0.519439	-3397.128536	0.073934	0.071205	-3397.202470	-3397.199742	-56.58

TS-VII-Rotation_b_conf_4	-3397.668994	-3396.619058	0.520083	-3397.123558	0.073932	0.071123	-3397.197489	-3397.194681	-54.48
TS-VII-Rotation_b_conf_5	-3397.676832	-3396.626924	0.520185	-3397.131495	0.073355	0.070658	-3397.204850	-3397.202154	-46.56
TS-VII-Rotation_b_conf_6	-3397.672689	-3396.622742	0.519673	-3397.127427	0.074721	0.071693	-3397.202148	-3397.199120	-60.44
TS-VII-Rotation_b_conf_7	-3397.675993	-3396.626091	0.519894	-3397.130999	0.073077	0.070464	-3397.204075	-3397.201463	-42.43
iodide	-297.806502	-297.806502	0.000000	-297.804142	0.016190	0.016190	-297.820332	-297.820332	

3.5 XYZ Coordinates:

53

A_Et_conf_1 Eopt -730.485802

C	-3.203683	0.675946	0.067898
C	-1.784039	0.885291	0.629339
C	-1.054989	-1.391382	-0.373322
C	-2.492445	-1.601720	-0.891558
H	-3.308121	1.198846	-0.894856
H	-2.761730	-2.672836	-0.845107
C	-1.714912	0.252977	2.053847
H	-2.721746	-0.070253	2.367476
C	-0.912668	-1.981641	1.057417
H	-0.033336	-2.643074	1.102249
H	-1.642295	1.975399	0.738220
H	-0.412177	-1.993974	-1.034050
C	-0.765519	-0.944883	2.179493
H	0.269496	-0.579735	2.171995
H	-0.911793	-1.432389	3.156711
C	-3.546828	-0.808433	-0.112706
H	-4.530272	-0.913184	-0.597024
H	-3.658045	-1.265540	0.883743
H	-1.421397	1.006566	2.800606
H	-1.776820	-2.632370	1.283622
H	-3.956949	1.125712	0.740502
H	-2.541792	-1.327475	-1.955537
B	-0.662152	0.235211	-0.430329
C	0.911028	0.552212	0.054148
C	2.025450	-0.025598	-0.463664
C	2.003269	-1.005250	-1.620451
H	0.999708	-1.056374	-2.041050
H	2.657988	-0.615316	-2.419875
C	3.433223	0.238489	0.065303
H	4.163265	0.001207	-0.724397
H	3.573271	1.305289	0.288611
C	1.134697	1.593277	1.155295
H	2.023976	1.361850	1.755099
H	0.296817	1.582047	1.858789
C	-0.900079	0.834551	-1.968746
H	-0.228505	0.327450	-2.682800
H	-1.911851	0.590905	-2.327029
C	2.471692	-2.419992	-1.266872
H	2.368286	-3.092032	-2.129938
H	3.526337	-2.436129	-0.960765
H	1.873040	-2.829600	-0.441206
C	3.790615	-0.580859	1.312611
H	3.743599	-1.656970	1.104719
H	4.804631	-0.346675	1.667482
H	3.088571	-0.379490	2.132710
C	1.271282	3.028055	0.634485
H	0.332753	3.374600	0.185694
H	1.525197	3.716599	1.452922
H	2.057468	3.104401	-0.130074
C	-0.704226	2.343059	-2.145611
H	0.338698	2.629068	-1.950541
H	-0.952111	2.688995	-3.161784
H	-1.334608	2.913828	-1.444916

53

A_Et_conf_10 Eopt -730.484902

C	3.008105	0.678618	-0.908713
C	1.728050	-0.145553	-1.125936
C	1.193105	-0.149066	1.398283
C	2.525567	0.581025	1.680488
H	2.806752	1.721133	-1.192671
H	3.025109	0.142675	2.564631
C	2.043305	-1.658186	-1.028374
H	2.898985	-1.907934	-1.683205
C	1.342738	-1.676649	1.424664
H	0.352019	-2.128138	1.241703
H	1.418352	0.035791	-2.173912
H	0.539587	0.104284	2.249072
C	2.325729	-2.205633	0.379040
H	2.279427	-3.305575	0.349383
H	3.349331	-1.965266	0.692161
C	3.563094	0.634339	0.531961
H	4.214777	1.508033	0.689639
H	4.231222	-0.231676	0.615686
H	1.197943	-2.238908	-1.421078
H	1.662290	-2.028605	2.422393
H	3.810395	0.337701	-1.588638
H	2.277525	1.611992	1.970834
B	0.555569	0.375850	-0.049135
C	-0.966328	-0.244809	-0.394606
C	-2.074381	-0.097166	0.376403
C	-2.132711	0.723533	1.650164
H	-2.653792	0.149073	2.436083
H	-1.128695	0.929409	2.022689
C	-3.416752	-0.751671	0.068011
H	-4.229540	-0.137709	0.483498
H	-3.610746	-0.813060	-1.009515
C	-1.185083	-0.972836	-1.724533
H	-1.878416	-1.818693	-1.609411
H	-0.251680	-1.406006	-2.087933
C	0.465089	2.040692	-0.029036
H	-0.156945	2.359584	0.825292
H	1.453219	2.480077	0.178785
C	-2.868537	2.054223	1.450818
H	-2.367138	2.653669	0.678703
H	-3.907884	1.901046	1.130565
H	-2.888586	2.640450	2.380303
C	-3.508468	-2.152734	0.682553
H	-4.496891	-2.605635	0.520208
H	-2.748897	-2.816322	0.246616
H	-3.327547	-2.112301	1.766020
C	-1.703473	-0.051418	-2.835203
H	-2.614500	0.480716	-2.527179
H	-0.948488	0.703761	-3.089551
H	-1.929387	-0.620962	-3.748004
C	-0.083254	2.706096	-1.293102
H	0.507889	2.424241	-2.179701
H	-1.118655	2.390562	-1.486560
H	-0.079657	3.806509	-1.238515

53

A_Et_conf_11 Eopt -730.486333

C	-3.157036	0.077129	1.105531
C	-1.699289	-0.402590	1.226468
C	-1.287605	0.185557	-1.246221
C	-2.746502	0.670575	-1.369043
H	-3.212609	1.087032	1.541100
H	-3.143670	0.430672	-2.372475
C	-1.523430	-1.896958	0.912810
H	-2.183389	-2.522649	1.542235
C	-1.131897	-1.309775	-1.579575
H	-0.056353	-1.534238	-1.638215
H	-1.458958	-0.272346	2.295430
H	-0.766029	0.745221	-2.038736
C	-1.758732	-2.277824	-0.559339
H	-1.357996	-3.288412	-0.735617
H	-2.836614	-2.361250	-0.744259
C	-3.728568	0.130222	-0.319464
H	-4.637692	0.752248	-0.321018
H	-4.065885	-0.870429	-0.616773
H	-0.490954	-2.177841	1.180516
H	-1.550169	-1.540428	-2.577142
H	-3.824247	-0.549432	1.726025
H	-2.742053	1.770299	-1.311499
B	-0.669098	0.496537	0.270592
C	0.918376	0.006233	0.546739
C	1.958453	-0.169291	-0.310584
C	1.949707	0.148603	-1.792503
H	2.303008	-0.733815	-2.356231
H	0.938514	0.355170	-2.136157
C	3.288080	-0.798063	0.103674
H	4.088195	-0.454221	-0.566948
H	3.598979	-0.502893	1.112096
C	1.262613	-0.222518	2.024602
H	1.901184	-1.107107	2.161546
H	0.357657	-0.427938	2.604052
C	-0.789391	2.103137	0.663818

H -1.817556 2.461639 0.492690
H -0.624995 2.239231 1.749421
C 2.841285 1.340544 -2.163181
H 2.495590 2.251712 -1.658966
H 3.889087 1.177166 -1.878650
H 2.817549 1.522692 -3.246648
C 3.217928 -2.327464 0.019815
H 4.185096 -2.793783 0.254976
H 2.466653 -2.720552 0.718618
H 2.926144 -2.646205 -0.991027
C 1.956629 0.990027 2.659242
H 2.886688 1.241065 2.130848
H 1.307543 1.873794 2.617342
H 2.202921 0.803290 3.714548
C 0.171398 3.015186 -0.100793
H 1.214661 2.761458 0.135395
H 0.052105 2.885750 -1.188938
H 0.030970 4.086033 0.117539
53
A_Et_conf_12 Eopt -730.485202
C 3.034480 -1.127612 -0.527063
C 1.714983 -1.170908 0.267147
C 1.292558 1.309965 -0.337125
C 2.620795 1.327374 -1.123023
H 2.867110 -1.528791 -1.538824
H 3.092241 2.324346 -1.049737
C 1.974421 -0.755279 1.744041
H 3.060681 -0.692280 1.934044
C 1.570978 1.705408 1.142303
H 0.940528 2.557580 1.437246
H 1.409529 -2.229310 0.270803
H 0.679892 2.110006 -0.778354
C 1.333523 0.577125 2.149857
H 0.249093 0.435726 2.238297
H 1.693968 0.878144 3.146885
C 3.624860 0.281416 -0.630725
H 4.513563 0.273500 -1.280934
H 3.987698 0.583194 0.364985
H 1.614475 -1.537606 2.429500
H 2.608649 2.067741 1.249701
H 3.787070 -1.788483 -0.059291
H 2.421844 1.167491 -2.193234
B 0.575143 -0.198994 -0.474947
C -0.965114 -0.381705 0.164386
C -2.026190 0.428036 -0.080895
C -1.951524 1.730928 -0.853851
H -1.086791 1.733970 -1.521626
H -2.842882 1.810989 -1.498361
C -3.460296 0.097529 0.314515
H -3.507702 -0.522172 1.216551
H -3.992811 1.030101 0.560761
C -1.287039 -1.696081 0.888255
H -0.526498 -2.446361 0.640294
H -2.232399 -2.107303 0.503989
C 0.392532 -0.644991 -2.072943
H -0.312408 0.048749 -2.566569
H 1.332106 -0.547018 -2.639498
C -1.902427 2.962848 0.053301
H -1.896633 3.894764 -0.529428
H -2.768852 2.994228 0.728943
H -0.998801 2.940456 0.674893
C -4.214946 -0.606371 -0.819411
H -3.744935 -1.570683 -1.057531
H -5.265044 -0.793488 -0.553721
H -4.201826 0.001538 -1.734704
C -1.399145 -1.620180 2.418457
H -2.046077 -0.790120 2.733544
H -1.821968 -2.551111 2.822113
H -0.422284 -1.472372 2.890688
C -0.129118 -2.071458 -2.279106
H 0.543782 -2.812584 -1.819342
H -1.118048 -2.200861 -1.814487
H -0.231972 -2.343271 -3.341580
53
A_Et_conf_13 Eopt -730.483965
C -3.157673 0.664705 -0.868077
C -1.784449 1.178013 -0.392332
C -1.234385 -1.166080 0.567336
C -2.598786 -1.676845 0.055169
H -3.113333 0.425727 -1.940129
H -3.003461 -2.438453 0.746476
C -1.908976 1.723436 1.061972
H -2.972354 1.759306 1.359669
C -1.424573 -0.590666 2.003926
H -0.791596 -1.124620 2.728463
H -1.536730 2.030762 -1.047121
H -0.587513 -2.054222 0.642533
C -1.146202 0.910797 2.113956
H -0.068887 1.083066 1.982545
H -1.404924 1.269516 3.123123
C -3.644414 -0.563229 -0.088872
H -4.559374 -0.963986 -0.552344
H -3.943992 -0.235823 0.919180
H -1.564699 2.767430 1.112289
H -2.456517 -0.773414 2.349053
H -3.919688 1.459591 -0.769807
H -2.466173 -2.187881 -0.909922
B -0.644279 -0.044869 -0.524764
C 0.911230 0.549735 -0.375413
C 1.982769 0.085875 0.318668
C 1.919772 -1.072391 1.304152
H 2.671259 -0.896184 2.092055
H 0.951695 -1.070648 1.808775
C 3.365435 0.727595 0.283194
H 3.402596 1.555487 -0.430023
H 3.562531 1.174000 1.274599
C 1.133644 1.783880 -1.255939
H 0.325933 1.857961 -1.992332
H 2.060487 1.702941 -1.843786
C -0.657189 -0.675402 -2.075146
H -1.678114 -0.934616 -2.392369
H -0.334391 0.098210 -2.794200
C 2.152874 -2.470073 0.720254
H 2.171895 -3.226741 1.517967
H 1.350069 -2.734557 0.024608
H 3.099539 -2.534821 0.169841
C 4.515415 -0.232153 -0.037429
H 4.351247 -0.729050 -1.004343
H 5.467799 0.312633 -0.092858
H 4.624962 -1.010044 0.729382
C 1.154824 3.087793 -0.453276
H 0.225496 3.197589 0.119855
H 1.985426 3.107721 0.264730
H 1.251785 3.964634 -1.109835
C 0.216341 -1.913810 -2.290135
H 1.261807 -1.716286 -2.009207
H -0.130572 -2.755850 -1.669854
H 0.215249 -2.262122 -3.335259
53
A_Et_conf_14 Eopt -730.486474
C 3.164887 -0.513397 0.818454
C 1.891797 0.334489 0.984570
C 1.068500 -0.385009 -1.351084
C 2.390911 -1.146366 -1.575290
H 3.022272 -1.475802 1.331709
H 2.733460 -1.033428 -2.620893
C 2.117998 1.772157 0.476297
H 3.068205 2.174003 0.875886
C 1.123432 1.080150 -1.815076
H 0.111445 1.501737 -1.696897
H 1.728312 0.421135 2.074076
H 0.338593 -0.873768 -2.015660
C 2.123547 1.961947 -1.053164
H 1.918635 3.020103 -1.282364
H 3.130686 1.775368 -1.446903
C 3.560552 -0.775710 -0.643713
H 4.313588 -1.578892 -0.677954
H 4.072470 0.112538 -1.035216
H 1.334771 2.413713 0.897731
H 1.358433 1.147959 -2.893112

H 4.020911 -0.028869 1.323207
H 2.179416 -2.219854 -1.450343
B 0.608858 -0.443049 0.246268
C -0.889726 0.264249 0.532538
C -2.059253 -0.158028 -0.016819
C -2.143152 -1.258774 -1.062301
H -2.412215 -0.819586 -2.039215
H -1.159884 -1.704701 -1.195423
C -3.429222 0.410294 0.327933
H -4.058010 -0.392395 0.748188
H -3.360004 1.168306 1.112204
C -0.977265 1.403246 1.551348
H -0.050730 1.447628 2.134678
H -1.772166 1.212587 2.288871
C 0.502924 -1.992537 0.857164
H 1.374167 -2.609037 0.576715
H -0.366577 -2.515221 0.426054
C -3.147660 -2.367700 -0.736000
H -2.960567 -2.778720 0.267038
H -4.186517 -2.012126 -0.762835
H -3.062631 -3.189834 -1.459094
C -4.161456 1.011074 -0.876498
H -3.539863 1.772306 -1.369797
H -4.403331 0.245939 -1.626674
H -5.104210 1.484443 -0.570057
C -1.225081 2.775545 0.910875
H -2.219397 2.823316 0.448546
H -1.153966 3.586720 1.650027
H -0.494234 2.976901 0.116246
C 0.356226 -2.036564 2.382682
H 0.179850 -3.051488 2.772786
H 1.256117 -1.650361 2.884832
H -0.489092 -1.409756 2.710821
53
A_Et_conf_15 Eopt -730.485963
C 3.076724 1.117744 0.586947
C 1.737694 1.242139 -0.156660
C 1.339018 -1.295406 0.024816
C 2.716433 -1.444453 0.710561
H 2.909935 1.406953 1.635960
H 3.210286 -2.370103 0.361574
C 1.884968 1.088055 -1.683930
H 2.638191 1.792982 -2.083087
C 1.427975 -1.442476 -1.501108
H 0.404505 -1.439374 -1.907763
H 1.406718 2.281161 0.020756
H 0.782345 -2.169815 0.395504
C 2.228108 -0.324950 -2.187082
H 2.051990 -0.366914 -3.273255
H 3.300685 -0.517019 -2.062253
C 3.711763 -0.281091 0.550321
H 4.475417 -0.352897 1.340866
H 4.264145 -0.398381 -0.390195
H 0.928422 1.375077 -2.148299
H 1.871655 -2.414654 -1.785388
H 3.812846 1.842592 0.192754
H 2.537138 -1.600576 1.785101
B 0.613756 0.157271 0.425938
C -0.883079 0.508486 -0.274261
C -1.894615 -0.330679 -0.627851
C -1.859057 -1.836255 -0.449187
H -1.909185 -2.315098 -1.444142
H -0.912737 -2.137296 -0.006222
C -3.216617 0.122010 -1.251692
H -3.041968 0.836926 -2.067712
H -3.691972 -0.748866 -1.727118
C -1.159410 2.002388 -0.471288
H -2.043783 2.166848 -1.096672
H -0.330505 2.472260 -1.016670
C 0.476855 0.268122 2.081863
H 0.268757 1.303713 2.399583
H 1.448302 0.028422 2.543628
C -3.001907 -2.401443 0.402158
H -3.985327 -2.212915 -0.048730
H -2.894053 -3.489031 0.513390
H -2.998522 -1.957473 1.406763
C -4.210830 0.732163 -0.259089
H -5.175307 0.948230 -0.741069
H -4.394617 0.051017 0.582453
H -3.824687 1.670469 0.159003
C -1.356489 2.791164 0.831604
H -0.404060 2.962063 1.345825
H -1.804252 3.773398 0.624780
H -2.018095 2.255091 1.527190
C -0.577918 -0.646057 2.711430
H -1.588801 -0.367805 2.378116
H -0.423343 -1.697027 2.417719
H -0.576590 -0.615087 3.812617
53
A_Et_conf_16 Eopt -730.485592
C 2.865044 -0.735017 1.284574
C 1.713156 0.274164 1.137032
C 1.284204 -0.514248 -1.283795
C 2.512885 -1.441718 -1.190160
H 2.496320 -1.632420 1.802088
H 3.072696 -1.444050 -2.144023
C 2.248068 1.625107 0.619132
H 3.165897 1.904414 1.170078
C 1.623201 0.893031 -1.799027
H 0.672010 1.436188 -1.918457
H 1.341589 0.466032 2.160650
H 0.636341 -0.963381 -2.054762
C 2.558365 1.706647 -0.890485
H 2.533219 2.763080 -1.203344
H 3.590664 1.381495 -1.071631
C 3.507544 -1.144754 -0.051062
H 4.144339 -2.029519 0.107139
H 4.195639 -0.349829 -0.366434
H 1.519867 2.405537 0.866845
H 2.078848 0.851215 -2.805350
H 3.659593 -0.321210 1.932361
H 2.142939 -2.471005 -1.064826
B 0.513647 -0.395824 0.182638
C -0.911643 0.497949 0.130440
C -2.039850 0.122282 -0.528227
C -2.111267 -1.055277 -1.482906
H -2.371005 -0.671120 -2.485996
H -1.128966 -1.517040 -1.575462
C -3.383345 0.831649 -0.372459
H -3.255432 1.919235 -0.318391
H -3.992769 0.648447 -1.271683
C -1.018549 1.775803 0.965356
H -0.263993 1.766201 1.761504
H -1.986590 1.839828 1.481079
C 0.100695 -1.882518 0.816053
H -0.685311 -2.352812 0.204945
H 0.944283 -2.591945 0.779417
C -3.137893 -2.130338 -1.110804
H -2.958842 -2.506596 -0.093345
H -4.166053 -1.746426 -1.152586
H -3.075158 -2.980029 -1.804125
C -4.183284 0.376085 0.855861
H -3.625539 0.567285 1.782038
H -5.143165 0.908256 0.923141
H -4.393784 -0.699417 0.819552
C -0.842544 3.053604 0.131139
H -0.839356 3.950486 0.767363
H 0.097043 3.038027 -0.433749
H -1.652869 3.162454 -0.601236
C -0.403297 -1.817022 2.261425
H -0.763551 -2.786359 2.640927
H 0.389062 -1.478087 2.946462
H -1.235148 -1.100382 2.354103
53
A_Et_conf_17 Eopt -730.480565
C 3.110471 0.344574 -0.799739
C 1.595568 0.410178 -1.144453
C 1.068383 -1.123346 0.866418
C 2.581956 -1.146921 1.234925
H 3.590661 1.308030 -1.029690

H	3.023846	-2.109258	0.921001
C	1.234846	-0.746002	-2.096381
H	1.814514	-0.682203	-3.035491
C	0.758388	-2.271662	-0.116095
H	-0.322144	-2.304621	-0.318680
H	1.446252	1.346535	-1.703412
H	0.532251	-1.336713	1.802085
C	1.477917	-2.116480	-1.458244
H	1.163125	-2.915584	-2.146913
H	2.560296	-2.258044	-1.310043
C	3.430065	-0.005490	0.657808
H	3.272893	0.890048	1.271402
H	4.498868	-0.254641	0.754009
H	0.171011	-0.671302	-2.377664
H	1.029339	-3.249523	0.322928
H	3.613013	-0.385901	-1.456802
H	2.697481	-1.125174	2.329444
B	0.595219	0.328803	0.196137
C	-1.013556	0.387647	-0.283175
C	-2.090527	-0.234837	0.262288
C	-2.052407	-1.149816	1.479881
H	-2.996249	-1.714262	1.529577
H	-1.262337	-1.901046	1.405790
C	-3.516546	-0.085474	-0.256652
H	-4.205406	-0.024411	0.602354
H	-3.666117	0.835481	-0.827258
C	-1.325708	1.392779	-1.398583
H	-2.073378	0.994066	-2.101512
H	-0.431664	1.578943	-2.003003
C	0.820299	1.626577	1.247176
H	-0.159211	1.939624	1.646075
H	1.390382	1.309541	2.139228
C	-1.885830	-0.372350	2.787599
H	-0.912464	0.133459	2.808961
H	-2.665134	0.397382	2.886550
H	-1.948912	-1.035138	3.662536
C	-3.918374	-1.279243	-1.129234
H	-3.783515	-2.226345	-0.587798
H	-4.968537	-1.217615	-1.447657
H	-3.286721	-1.319338	-2.028198
C	-1.823371	2.740813	-0.860861
H	-2.747964	2.623836	-0.280110
H	-1.079866	3.193036	-0.193951
H	-2.020563	3.450882	-1.677225
C	1.498750	2.874233	0.666450
H	1.487319	3.729259	1.361167
H	2.549696	2.681815	0.409370
H	1.011203	3.204085	-0.262528
53			
A_Et_conf_18		Eopt	-730.485392
C	-3.193370	-0.753435	0.439447
C	-1.814668	-1.115783	-0.139518
C	-1.085055	1.353246	-0.038340
C	-2.490472	1.748239	0.460901
H	-3.189897	-0.987722	1.514640
H	-2.809994	2.702887	0.003169
C	-1.809353	-1.060200	-1.678032
H	-2.647216	-1.650918	-2.093843
C	-0.961435	1.365404	-1.571238
H	0.086384	1.134608	-1.823474
H	-1.657378	-2.175479	0.128604
H	-0.421249	2.149778	0.330956
C	-1.854917	0.348774	-2.293889
H	-1.549447	0.287338	-3.350498
H	-2.887325	0.718882	-2.313733
C	-3.613029	0.714562	0.248501
H	-4.443843	0.943985	0.934322
H	-4.034018	0.834953	-0.757345
H	-0.894225	-1.551798	-2.034759
H	-1.166442	2.370473	-1.984338
H	-3.979269	-1.394080	-0.001575
H	-2.410389	1.954198	1.539231
B	-0.642350	-0.139011	0.537840
C	0.933498	-0.602523	0.160376
C	2.055499	0.095127	0.487044
C	3.465605	-0.479681	0.439356
H	3.859457	-0.466684	1.471474
H	3.453631	-1.531572	0.141325
C	2.026849	1.515016	1.033394
H	1.099547	1.677363	1.589348
H	2.849754	1.636124	1.758425
C	1.140773	-1.998369	-0.436682
H	0.189601	-2.539838	-0.453535
H	1.803715	-2.605893	0.199586
C	-0.738714	-0.249791	2.200008
H	0.040990	0.372725	2.670588
H	-1.692463	0.153447	2.580943
C	4.471054	0.266156	-0.445054
H	4.083995	0.396971	-1.465724
H	4.704187	1.260453	-0.043467
H	5.416023	-0.290942	-0.506735
C	2.152094	2.617813	-0.027188
H	1.381357	2.500440	-0.797897
H	2.028028	3.610808	0.428848
H	3.125835	2.599998	-0.529518
C	1.714235	-1.975733	-1.859045
H	1.115502	-1.326289	-2.512441
H	2.738076	-1.581118	-1.871409
H	1.731744	-2.981233	-2.303938
C	-0.581791	-1.678368	2.731857
H	0.366403	-2.123165	2.390456
H	-0.589026	-1.735622	3.831936
H	-1.391123	-2.330762	2.369796
53			
A_Et_conf_19		Eopt	-730.479789
C	-3.078530	-0.283110	-1.248690
C	-1.755932	0.536692	-1.155267
C	-1.238236	-0.548647	1.133131
C	-2.580506	-1.329403	1.098897
H	-3.204233	-0.670777	-2.271051
H	-3.302324	-0.860000	1.789717
C	-2.062342	1.873512	-0.459291
H	-2.879746	2.403733	-0.982107
C	-1.460499	0.828945	1.786993
H	-0.496780	1.359210	1.854391
H	-1.452614	0.773377	-2.191916
H	-0.564432	-1.103797	1.801062
C	-2.454669	1.696005	1.009562
H	-2.559974	2.679762	1.493242
H	-3.451951	1.228644	1.061373
C	-3.231307	-1.480506	-0.287537
H	-2.796778	-2.366682	-0.764792
H	-4.301737	-1.708122	-0.165441
H	-1.192283	2.536161	-0.515195
H	-1.827440	0.714405	2.823448
H	-3.933414	0.398840	-1.099256
H	-2.427232	-2.343521	1.498566
B	-0.562493	-0.333447	-0.375554
C	0.926383	0.440186	-0.282396
C	2.008408	-0.101508	0.329231
C	1.961850	-1.389143	1.127918
H	1.063922	-1.959138	0.874770
H	2.824411	-2.018110	0.848103
C	3.430338	0.430104	0.202138
H	3.457488	1.512842	0.046713
H	3.976828	0.246021	1.140429
C	1.170922	1.736754	-1.059266
H	0.364342	1.889002	-1.787823
H	2.093386	1.660637	-1.657044
C	-0.342438	-1.769193	-1.241692
H	-0.378115	-2.656879	-0.581332
H	-1.201267	-1.894204	-1.918797
C	2.015673	-1.150960	2.640825
H	2.943265	-0.638177	2.930465
H	1.175241	-0.520586	2.960973
H	1.969199	-2.096756	3.198748
C	4.192159	-0.248762	-0.943405
H	5.231169	0.104556	-0.998106
H	4.215434	-1.339622	-0.813172
H	3.707685	-0.039797	-1.907128

C 1.277746 2.990239 -0.177898
H 2.160563 2.950078 0.471745
H 1.349072 3.903297 -0.786724
H 0.407591 3.092556 0.482387
C 0.908387 -1.877899 -2.121446
H 1.015647 -0.987123 -2.762156
H 1.825411 -1.945475 -1.522132
H 0.877686 -2.756963 -2.785047
53
A_Et_conf_2 Eopt -730.481806
C -3.115684 -0.729893 0.482943
C -1.676028 -1.255188 0.324356
C -1.038643 0.970895 -0.822143
C -2.516105 1.409781 -0.766597
H -3.219321 -0.228728 1.459381
H -2.772203 1.982029 -1.677122
C -1.548800 -1.994390 -1.039792
H -2.541597 -2.104779 -1.505984
C -0.775520 0.212421 -2.155027
H 0.137581 0.604719 -2.629608
H -1.516161 -1.991620 1.132681
H -0.457551 1.905099 -0.871469
C -0.607365 -1.312408 -2.042542
H 0.427449 -1.518321 -1.740652
H -0.730463 -1.769867 -3.037117
C -3.502653 0.249060 -0.628918
H -4.518253 0.638162 -0.456183
H -3.551719 -0.294625 -1.585628
H -1.197807 -3.026960 -0.890930
H -1.589396 0.430123 -2.869935
H -3.841050 -1.564003 0.486447
H -2.664374 2.099644 0.071423
B -0.612732 0.021981 0.489684
C 0.983130 -0.474443 0.417979
C 2.044820 0.360682 0.313308
C 1.907263 1.870008 0.327388
H 0.885024 2.143641 0.599949
H 2.556957 2.262804 1.129831
C 3.489639 -0.116467 0.202641
H 4.165195 0.709827 0.473137
H 3.697523 -0.918397 0.925927
C 1.272307 -1.952416 0.656938
H 2.228626 -2.276570 0.224823
H 0.503757 -2.578212 0.190609
C -0.795865 0.715513 2.022933
H -1.288439 -0.028313 2.678170
H 0.211535 0.826173 2.456496
C 2.297837 2.560036 -0.982302
H 2.149546 3.646954 -0.918229
H 3.354036 2.383866 -1.228900
H 1.689708 2.178928 -1.814307
C 3.857697 -0.603775 -1.203525
H 3.702783 0.191808 -1.944517
H 4.907886 -0.924735 -1.259506
H 3.225183 -1.451090 -1.501231
C 1.283977 -2.255906 2.160223
H 0.338089 -1.945991 2.625208
H 1.425058 -3.328262 2.356638
H 2.093350 -1.706502 2.661978
C -1.505348 2.059877 2.246608
H -2.595146 1.985252 2.124528
H -1.325320 2.450573 3.260911
H -1.154778 2.827145 1.537927
53
A_Et_conf_20 Eopt -730.479526
C 3.047182 -0.972857 0.218942
C 1.602221 -0.923321 0.790626
C 1.142479 1.294936 -0.447674
C 2.575697 1.247470 -1.058901
H 3.324301 -2.009487 -0.025722
H 3.216123 1.992171 -0.555360
C 1.639389 -0.116352 2.111335
H 2.319209 -0.602293 2.834776
C 1.180244 2.042255 0.896175
H 0.160174 2.106262 1.308742
H 1.323104 -1.956049 1.061560
H 0.543493 1.910390 -1.132206
C 2.069875 1.336979 1.917897
H 2.042034 1.867522 2.882022
H 3.117927 1.376302 1.580897
C 3.285711 -0.119062 -1.035921
H 2.950832 -0.695558 -1.906845
H 4.367806 0.030687 -1.176856
H 0.648398 -0.097977 2.585098
H 1.531855 3.081616 0.760195
H 3.764952 -0.667194 0.999655
H 2.537765 1.573716 -2.109350
B 0.500971 -0.240406 -0.271671
C -1.046769 -0.279115 0.379165
C -2.083425 0.496080 -0.033025
C -2.003920 1.458789 -1.204620
H -1.141352 1.222899 -1.832697
H -2.896621 1.293258 -1.833770
C -3.499121 0.438449 0.535822
H -3.492405 0.347492 1.630348
H -3.998805 1.395207 0.322851
C -1.370592 -1.373175 1.393358
H -2.324424 -1.186935 1.903260
H -0.613751 -1.422092 2.185325
C 0.420459 -0.972185 -1.791436
H -0.624177 -0.933974 -2.145645
H 0.973982 -0.350865 -2.516928
C -1.971260 2.938147 -0.809936
H -1.981447 3.586810 -1.697232
H -2.839340 3.205876 -0.191516
H -1.067230 3.166950 -0.231722
C -4.348527 -0.688284 -0.059509
H -4.318952 -0.658365 -1.158281
H -3.972480 -1.669861 0.255990
H -5.399388 -0.610830 0.254518
C -1.433754 -2.767119 0.753900
H -1.856115 -3.502069 1.453646
H -2.050226 -2.764948 -0.155960
H -0.431300 -3.109662 0.474524
C 0.896876 -2.421570 -1.963013
H 0.931089 -2.728495 -3.020906
H 1.902755 -2.578153 -1.548868
H 0.230765 -3.130706 -1.454297
53
A_Et_conf_21 Eopt -730.485625
C 3.213842 0.870682 0.570258
C 1.733079 1.226775 0.338931
C 1.316281 -1.119662 -0.668025
C 2.803586 -1.450026 -0.416813
H 3.360660 0.540579 1.609334
H 3.174032 -2.135112 -1.200909
C 1.574479 1.838804 -1.086944
H 2.568713 2.010782 -1.535352
C 1.144995 -0.488593 -2.078100
H 0.386064 -1.039602 -2.654099
H 1.492703 2.017843 1.068456
H 0.817375 -2.100691 -0.674044
C 0.744198 0.992122 -2.059050
H -0.311029 1.059171 -1.761736
H 0.814734 1.413170 -3.074706
C 3.711862 -0.219266 -0.382463
H 4.739281 -0.514872 -0.118823
H 3.773982 0.200073 -1.399414
H 1.116713 2.837663 -1.027970
H 2.080019 -0.594670 -2.656398
H 3.849595 1.766034 0.443223
H 2.899149 -1.998370 0.533240
B 0.762871 -0.125783 0.556900
C -0.850430 0.323339 0.654145
C -1.967049 -0.204829 0.090049
C -1.938250 -1.335715 -0.922313
H -2.156693 -0.937158 -1.929772
H -0.930941 -1.742336 -0.969971
C -3.380665 0.298267 0.353233
H -3.987109 -0.515848 0.784471

H	-3.385817	1.096914	1.099417
C	-1.052168	1.484994	1.633587
H	-0.155548	1.596082	2.253743
H	-1.870399	1.277450	2.339512
C	1.025930	-0.863754	2.032613
H	2.099026	-0.963426	2.261345
H	0.623912	-0.237900	2.848571
C	-2.917381	-2.477589	-0.633398
H	-3.965476	-2.164598	-0.729743
H	-2.756664	-3.308735	-1.332954
H	-2.774916	-2.862903	0.386594
C	-4.084967	0.804418	-0.910488
H	-3.484128	1.579410	-1.408194
H	-4.247752	-0.003196	-1.636911
H	-5.065975	1.236007	-0.669541
C	-1.314752	2.823822	0.939552
H	-2.242508	2.798666	0.352322
H	-1.392809	3.646243	1.665376
H	-0.497375	3.062521	0.247809
C	0.394393	-2.255502	2.145758
H	0.475695	-2.686199	3.156296
H	-0.676202	-2.227781	1.888549
H	0.873441	-2.966027	1.454568

53

A_Et_conf_22 Eopt -730.479639

C	3.083630	-0.962341	-0.720527
C	1.666379	-1.300562	-0.179901
C	1.329117	1.152693	0.542036
C	2.774954	1.474702	0.054415
H	3.268759	-1.517324	-1.653112
H	3.462654	1.497332	0.916731
C	1.748450	-1.687662	1.312900
H	2.422401	-2.552289	1.455934
C	1.382098	0.726114	2.020081
H	0.359655	0.544999	2.389702
H	1.335415	-2.202091	-0.719803
H	0.798385	2.112102	0.502021
C	2.213695	-0.544272	2.215810
H	2.180198	-0.862604	3.269079
H	3.271430	-0.320709	2.004284
C	3.344065	0.524493	-1.003267
H	2.900028	0.784108	-1.972351
H	4.426845	0.696336	-1.111391
H	0.757143	-2.007664	1.668322
H	1.807529	1.530418	2.647945
H	3.849776	-1.327390	-0.013219
H	2.808626	2.494371	-0.358811
B	0.578213	-0.044624	-0.365529
C	-0.942784	-0.573703	0.140597
C	-1.974946	0.139034	0.665031
C	-1.918487	1.625379	0.949785
H	-2.117757	1.799299	2.022688
H	-0.918383	2.004266	0.750913
C	-3.345286	-0.445791	1.014053
H	-3.238179	-1.374922	1.591755
H	-3.858017	0.257660	1.687317
C	-1.227075	-2.047732	-0.166230
H	-2.157739	-2.385985	0.305135
H	-0.439218	-2.677464	0.266310
C	0.364141	0.419152	-1.969259
H	1.137174	-0.013616	-2.629105
H	-0.582516	0.002344	-2.343100
C	-2.929519	2.443868	0.138529
H	-2.787012	2.275915	-0.938550
H	-3.965964	2.177820	0.386291
H	-2.808303	3.517910	0.335343
C	-4.259003	-0.712271	-0.186174
H	-3.855029	-1.509762	-0.822053
H	-5.263723	-1.018223	0.139926
H	-4.361164	0.184466	-0.811336
C	-1.308465	-2.398611	-1.657405
H	-1.585660	-3.454628	-1.785165
H	-2.058682	-1.785820	-2.176174
H	-0.348042	-2.238599	-2.162041
C	0.302839	1.931499	-2.208621
H	1.250362	2.426095	-1.950250
H	0.072099	2.190920	-3.254180
H	-0.476990	2.391923	-1.582523

53

A_Et_conf_23 Eopt -730.486292

C	3.072764	0.744291	1.003652
C	1.726953	1.173281	0.391046
C	1.284017	-1.222171	-0.451723
C	2.613152	-1.689129	0.179063
H	2.937427	0.674568	2.092976
H	3.100091	-2.440041	-0.469986
C	1.859921	1.632389	-1.072805
H	2.557559	2.486139	-1.161980
C	1.466107	-0.748304	-1.904168
H	0.471857	-0.559592	-2.338665
H	1.413287	2.061384	0.968029
H	0.672449	-2.138510	-0.490674
C	2.286792	0.539035	-2.059274
H	2.172334	0.917560	-3.086943
H	3.356014	0.323994	-1.946482
C	3.648865	-0.591940	0.493279
H	4.361250	-0.980036	1.238207
H	4.253069	-0.404520	-0.402606
H	0.877276	2.000652	-1.405050
H	1.925114	-1.540925	-2.523831
H	3.838481	1.527869	0.852535
H	2.370014	-2.221246	1.111071
B	0.598599	-0.045792	0.507771
C	-0.926093	0.557124	0.165868
C	-1.972043	0.022383	-0.518331
C	-1.902238	-1.254122	-1.334609
H	-2.043509	-0.998084	-2.400845
H	-0.909015	-1.688344	-1.250295
C	-3.363791	0.652330	-0.534016
H	-3.299024	1.747111	-0.566822
H	-3.880058	0.357275	-1.461553
C	-1.221931	1.865689	0.915596
H	-0.545794	1.953041	1.775309
H	-2.232596	1.849718	1.342571
C	0.454261	-0.613663	2.071349
H	0.019480	0.164435	2.724234
H	1.443821	-0.825585	2.509187
C	-2.934762	-2.327482	-0.974410
H	-3.963583	-1.991797	-1.158722
H	-2.770160	-3.228768	-1.580529
H	-2.853147	-2.613610	0.083393
C	-4.245032	0.250700	0.657465
H	-5.223228	0.750595	0.612049
H	-4.419757	-0.831228	0.679924
H	-3.770038	0.521974	1.609279
C	-1.082896	3.147202	0.081395
H	-1.592616	3.055742	-0.887822
H	-1.518527	4.005543	0.612444
H	-0.031713	3.383092	-0.119208
C	-0.400707	-1.878398	2.197732
H	-0.526817	-2.215758	3.238783
H	-1.406633	-1.712573	1.782442
H	0.043656	-2.716407	1.638079

53

A_Et_conf_24 Eopt -730.479644

C	3.277747	0.432514	-0.396545
C	1.822121	0.527366	-0.933317
C	1.045701	-1.221236	0.799572
C	2.495703	-1.282067	1.367766
H	3.764241	1.418874	-0.443584
H	2.992060	-2.205982	1.021376
C	1.627855	-0.532436	-2.040071
H	2.344920	-0.368464	-2.865285
C	0.876371	-2.252433	-0.329890
H	-0.172275	-2.240495	-0.670311
H	1.729490	1.515084	-1.413705
H	0.390922	-1.554381	1.614386
C	1.786109	-1.964973	-1.525045
H	1.585336	-2.680212	-2.337677
H	2.833740	-2.134117	-1.228423

C	3.401697	-0.084960	1.040587
H	3.156528	0.738755	1.722489
H	4.450260	-0.349059	1.251338
H	0.623508	-0.441339	-2.480419
H	1.078503	-3.275620	0.036566
H	3.880058	-0.212842	-1.059265
H	2.457286	-1.375137	2.464073
B	0.652366	0.308936	0.246461
C	-0.881389	0.419633	-0.435895
C	-2.013224	-0.102231	0.103452
C	-2.037820	-0.753122	1.479125
H	-2.026295	-1.854791	1.401278
H	-1.124961	-0.474903	2.007620
C	-3.371852	-0.107449	-0.582867
H	-4.109119	0.453091	0.014714
H	-3.326915	0.398996	-1.550951
C	-1.055058	1.277944	-1.693972
H	-1.622077	0.752827	-2.478468
H	-0.082185	1.502779	-2.139646
C	0.709250	1.448757	1.478285
H	1.231355	1.055434	2.368556
H	-0.320804	1.644001	1.824978
C	-3.229457	-0.330568	2.344374
H	-3.292024	0.765894	2.404322
H	-4.187626	-0.700297	1.956249
H	-3.115728	-0.717201	3.365748
C	-3.897908	-1.529292	-0.810540
H	-4.024587	-2.075877	0.133597
H	-4.870166	-1.514809	-1.321963
H	-3.194115	-2.100674	-1.432404
C	-1.738873	2.614717	-1.385081
H	-2.756025	2.468906	-0.998658
H	-1.174704	3.159428	-0.615579
H	-1.798658	3.253758	-2.278098
C	1.334497	2.796821	1.105587
H	1.260877	3.546858	1.909228
H	2.400504	2.689534	0.856370
H	0.848024	3.225392	0.216096

53

A_Et_conf_25 Eopt -730.477169

C	-3.110376	0.074312	-1.202109
C	-1.750523	0.820809	-1.034270
C	-1.206922	-0.666500	1.006765
C	-2.589207	-1.365784	0.901414
H	-3.303248	-0.107643	-2.270300
H	-3.274026	-0.962500	1.667683
C	-1.955653	2.037500	-0.120558
H	-2.746928	2.700777	-0.516499
C	-1.338192	0.601397	1.879826
H	-0.349677	1.078717	1.974065
H	-1.482353	1.204871	-2.033924
H	-0.558665	-1.349494	1.570789
C	-2.317339	1.628499	1.308011
H	-2.358197	2.515237	1.959356
H	-3.333885	1.203075	1.315019
C	-3.260245	-1.276495	-0.475949
H	-2.831868	-2.065871	-1.106211
H	-4.330294	-1.520284	-0.383237
H	-1.037410	2.637222	-0.094762
H	-1.659930	0.333684	2.902687
H	-3.928672	0.739848	-0.876396
H	-2.484679	-2.433641	1.148315
B	-0.563214	-0.208264	-0.465539
C	0.977658	0.464841	-0.344544
C	2.055943	-0.100479	0.258121
C	2.012409	-1.290071	1.202106
H	1.130657	-1.905131	1.021699
H	2.884623	-1.934619	1.002254
C	3.497638	0.316172	-0.012976
H	3.591658	1.371637	-0.281772
H	4.099944	0.179229	0.899499
C	1.263083	1.698325	-1.210331
H	0.409228	1.887722	-1.872112
H	2.119661	1.520841	-1.880464
C	-0.368910	-1.367734	-1.663995
H	0.326606	-0.938401	-2.410815
H	-1.308210	-1.498286	-2.223416
C	2.049082	-0.873608	2.675374
H	2.914223	-0.228238	2.884358
H	1.143218	-0.310861	2.934926
H	2.111866	-1.747507	3.339057
C	4.107569	-0.530916	-1.136745
H	5.159799	-0.268979	-1.316691
H	4.064046	-1.601532	-0.893414
H	3.550677	-0.381050	-2.072461
C	1.541767	2.978438	-0.408135
H	0.755743	3.165191	0.333370
H	2.484401	2.914048	0.148863
H	1.601171	3.857037	-1.066548
C	0.154076	-2.755623	-1.280814
H	0.108130	-3.472097	-2.116355
H	1.203677	-2.712070	-0.957175
H	-0.426358	-3.188122	-0.448845

53

A_Et_conf_26 Eopt -730.478945

C	-2.560724	-1.717091	0.116059
C	-1.120539	-1.379600	-0.375190
C	-1.623564	1.140009	-0.093307
C	-3.081112	0.822333	0.343260
H	-2.526330	-2.593159	0.781525
H	-3.770439	1.004482	-0.498810
C	-1.139988	-1.194763	-1.904913
H	-1.499463	-2.113067	-2.404576
C	-1.581522	1.271575	-1.630534
H	-0.557807	1.508443	-1.955416
H	-0.512812	-2.272159	-0.166131
H	-1.367693	2.129751	0.321503
C	-2.004174	-0.013147	-2.342402
H	-1.937901	0.120256	-3.433031
H	-3.062828	-0.229103	-2.128580
C	-3.306086	-0.602450	0.871949
H	-2.990084	-0.629033	1.921774
H	-4.384608	-0.826221	0.879346
H	-0.119963	-1.016055	-2.278227
H	-2.224023	2.105137	-1.969775
H	-3.175080	-2.038390	-0.743244
H	-3.403824	1.524380	1.127016
B	-0.522913	-0.023418	0.390507
C	1.023794	0.471770	0.001774
C	2.086236	-0.256181	-0.430479
C	2.060460	-1.764238	-0.645551
H	1.198122	-2.063994	-1.248618
H	2.950437	-2.058492	-1.224304
C	3.478044	0.326133	-0.642943
H	3.842403	-0.001534	-1.631188
H	3.454352	1.419499	-0.683056
C	1.321247	1.920945	0.417908
H	2.304416	1.973527	0.908413
H	0.599882	2.219463	1.187684
C	-0.469976	-0.263552	2.064895
H	0.578626	-0.448295	2.356574
H	-0.992092	-1.203481	2.317869
C	2.043997	-2.566947	0.660108
H	2.906533	-2.321148	1.293438
H	1.138413	-2.345243	1.236861
H	2.064618	-3.648545	0.460844
C	4.499578	-0.112085	0.412201
H	4.649384	-1.200043	0.394367
H	5.476436	0.360323	0.238817
H	4.157854	0.164243	1.420230
C	1.282826	2.982492	-0.689900
H	1.726320	3.924941	-0.338182
H	1.840464	2.657846	-1.579395
H	0.255259	3.199389	-1.004139
C	-1.003620	0.836271	2.993956
H	-2.051782	1.085577	2.778449
H	-0.951097	0.550312	4.056966
H	-0.430379	1.768633	2.891107

53

A_Et_conf_27 Eopt -730.481022

C -3.234645 0.429973 -0.933371
C -1.809954 1.008774 -0.801409
C -1.186728 -0.841884 0.877363
C -2.583822 -1.471161 0.722564
H -3.307899 -0.086429 -1.900559
H -2.934305 -1.887868 1.684743
C -1.739131 2.072403 0.303246
H -2.481704 2.874349 0.133710
C -1.118409 0.238007 1.976347
H -0.065477 0.546523 2.068701
H -1.631171 1.530328 -1.758812
H -0.543095 -1.663709 1.226848
C -1.932379 1.513620 1.719249
H -1.637960 2.278349 2.455156
H -2.997244 1.328575 1.904918
C -3.682387 -0.541748 0.176845
H -4.514673 -1.155794 -0.201857
H -4.109908 0.030611 1.009022
H -0.753302 2.555934 0.257056
H -1.407255 -0.176111 2.960302
H -3.972886 1.251477 -0.987201
H -2.483480 -2.335589 0.046822
B -0.678550 -0.202228 -0.566161
C 0.917968 0.337584 -0.601836
C 2.022797 -0.348731 -0.198005
C 1.998882 -1.642066 0.608224
H 1.109095 -2.224838 0.363959
H 2.859543 -2.256596 0.293747
C 3.445733 0.018602 -0.607667
H 3.848924 -0.837566 -1.177294
H 3.456189 0.862809 -1.301831
C 1.168488 1.643267 -1.369493
H 0.222426 2.018744 -1.771403
H 1.803489 1.463596 -2.252246
C -0.816771 -1.230590 -1.874948
H -1.870623 -1.525282 -2.004174
H -0.580215 -0.634128 -2.778968
C 2.062649 -1.476385 2.131731
H 2.177915 -2.453861 2.622198
H 2.899283 -0.840719 2.444639
H 1.142955 -1.020671 2.512941
C 4.423375 0.309195 0.536266
H 4.616308 -0.586364 1.140957
H 5.389406 0.651880 0.140655
H 4.031890 1.088701 1.205422
C 1.801436 2.762799 -0.532065
H 2.834775 2.529330 -0.248753
H 1.810386 3.715094 -1.081998
H 1.244604 2.918220 0.401581
C 0.016728 -2.512446 -1.927126
H 1.093045 -2.294167 -1.875369
H -0.222925 -3.182502 -1.086113
H -0.156340 -3.086469 -2.851476
53
A_Et_conf_28 Eopt -730.481043
C -3.159146 -0.301823 -0.913699
C -1.828452 0.466822 -1.007609
C -1.101549 -0.463884 1.288484
C -2.480040 -1.135756 1.444595
H -3.078011 -1.244006 -1.474179
H -2.830885 -1.069827 2.491402
C -1.994079 1.879448 -0.413917
H -2.946154 2.323497 -0.760516
C -1.054651 0.968367 1.846888
H -0.007920 1.308043 1.788992
H -1.633106 0.612776 -2.085445
H -0.415230 -1.048372 1.924562
C -1.957139 1.985470 1.126914
H -1.641669 3.003369 1.407905
H -2.975779 1.886516 1.522606
C -3.597580 -0.604166 0.528123
H -4.425947 -1.330016 0.513568
H -4.023414 0.308879 0.964229
H -1.214087 2.523797 -0.833986
H -1.318259 0.984024 2.920357
H -3.968008 0.270398 -1.404062
H -2.351306 -2.210759 1.243984
B -0.620165 -0.450478 -0.299476
C 0.946139 0.128511 -0.527140
C 2.066377 -0.531788 -0.127945
C 2.046905 -1.711874 0.826805
H 1.022029 -1.993897 1.061822
H 2.515787 -2.586774 0.342052
C 3.487561 -0.234481 -0.615087
H 4.083950 -1.140032 -0.429165
H 3.483489 -0.111340 -1.708690
C 1.163520 1.392902 -1.349176
H 0.320941 1.547775 -2.037623
H 2.061463 1.311414 -1.981916
C -0.637742 -1.957065 -1.023431
H 0.170731 -2.589458 -0.627157
H -1.563695 -2.514385 -0.802488
C 2.792429 -1.421766 2.133568
H 2.747856 -2.284767 2.812098
H 3.852306 -1.191589 1.956783
H 2.340510 -0.560103 2.646274
C 4.244994 0.953033 -0.002255
H 5.328756 0.825211 -0.133309
H 3.969612 1.898810 -0.482779
H 4.042660 1.051516 1.072894
C 1.319892 2.647118 -0.473500
H 2.167388 2.540566 0.211546
H 1.482701 3.544702 -1.088110
H 0.436252 2.822323 0.148739
C -0.468825 -1.896919 -2.546386
H -1.329179 -1.409211 -3.028840
H 0.424809 -1.309895 -2.814698
H -0.359439 -2.889685 -3.010859
53
A_Et_conf_29 Eopt -730.482507
C 3.077188 1.221737 -0.298210
C 1.548912 1.275616 -0.482252
C 1.384519 -1.247669 0.062866
C 2.918080 -1.266032 0.241960
H 3.343806 1.547136 0.718514
H 3.319691 -2.248616 -0.065573
C 1.204336 0.952205 -1.968425
H 2.129064 0.922347 -2.570602
C 1.018484 -1.554756 -1.415284
H 0.285173 -2.374943 -1.461668
H 1.256393 2.322315 -0.295336
H 1.032154 -2.088687 0.677883
C 0.438175 -0.359696 -2.183184
H -0.597012 -0.211350 -1.850734
H 0.394215 -0.592678 -3.259284
C 3.646285 -0.176852 -0.546924
H 4.722773 -0.197418 -0.316193
H 3.566224 -0.407180 -1.621498
H 0.609473 1.766721 -2.408337
H 1.905946 -1.930041 -1.955344
H 3.573434 1.931813 -0.984795
H 3.160913 -1.161681 1.311070
B 0.787096 0.224880 0.582982
C -0.862949 0.539298 0.594338
C -1.934610 -0.295471 0.543674
C -1.808796 -1.806385 0.502829
H -0.757531 -2.077136 0.571032
H -2.287264 -2.213883 1.411629
C -3.379881 0.186422 0.559439
H -3.983339 -0.503126 1.173759
H -3.448544 1.164574 1.050231
C -1.137690 2.035459 0.794812
H -0.215692 2.514291 1.140920
H -1.866875 2.190931 1.605515
C 1.242256 0.476836 2.168879
H 2.332295 0.598006 2.275476
H 0.818549 1.424433 2.544766
C -2.431975 -2.513277 -0.708597
H -2.134174 -2.028492 -1.649242
H -2.102679 -3.560826 -0.748541

H	-3.527725	-2.519542	-0.662480
C	-4.045131	0.297659	-0.825916
H	-4.343451	-0.679200	-1.220270
H	-4.949448	0.920513	-0.773737
H	-3.363412	0.750532	-1.557380
C	-1.617122	2.796859	-0.444579
H	-0.996263	2.554811	-1.316851
H	-2.655217	2.552779	-0.697624
H	-1.561218	3.883100	-0.283802
C	0.800635	-0.647598	3.111443
H	0.993390	-0.424638	4.172756
H	-0.277484	-0.847326	3.009119
H	1.324055	-1.588065	2.880501

53

A_Et_conf_3 Eopt -730.483375

C	-3.259462	-0.250068	0.865938
C	-1.866207	-0.896243	0.740118
C	-1.216332	0.848401	-1.051753
C	-2.624530	1.462055	-0.914333
H	-3.274952	0.453917	1.710884
H	-2.957159	1.868244	-1.886891
C	-1.929867	-1.997491	-0.360304
H	-2.974924	-2.147501	-0.680514
C	-1.204299	-0.266815	-2.135239
H	-0.364209	-0.097807	-2.827409
H	-1.671029	-1.388874	1.709380
H	-0.597896	1.670209	-1.438174
C	-1.076266	-1.703698	-1.600639
H	-0.022738	-1.881956	-1.346947
H	-1.325550	-2.419184	-2.400504
C	-3.682074	0.478710	-0.411895
H	-4.640762	0.997839	-0.256697
H	-3.870465	-0.266631	-1.201542
H	-1.619525	-2.973047	0.044597
H	-2.113794	-0.197058	-2.759282
H	-4.017511	-1.019618	1.100579
H	-2.576111	2.323326	-0.229010
B	-0.691570	0.261262	0.422670
C	0.846549	-0.424000	0.426244
C	1.989249	0.067564	-0.121750
C	3.329865	-0.655372	-0.062093
H	4.144520	0.077233	0.015069
H	3.414378	-1.274663	0.837777
C	2.036098	1.339197	-0.953984
H	2.074768	1.079901	-2.027773
H	1.103598	1.877571	-0.807339
C	0.981101	-1.738840	1.197813
H	1.790321	-2.366587	0.801069
H	0.071688	-2.335549	1.078344
C	-0.747994	1.415427	1.624173
H	-1.765607	1.827983	1.706025
H	-0.570909	0.921515	2.597597
C	3.570081	-1.521273	-1.303304
H	2.799695	-2.301080	-1.385450
H	3.523557	-0.913983	-2.218459
H	4.553107	-2.012118	-1.274352
C	3.198690	2.290443	-0.650752
H	4.157292	1.918316	-1.035341
H	3.020203	3.269883	-1.114622
H	3.311064	2.445852	0.431913
C	1.213173	-1.519581	2.695848
H	1.326590	-2.472935	3.231499
H	2.119322	-0.920385	2.866927
H	0.370209	-0.978526	3.144880
C	0.213949	2.601885	1.521101
H	1.259198	2.263405	1.504370
H	0.041798	3.181575	0.600107
H	0.109338	3.302625	2.364829

53

A_Et_conf_30 Eopt -730.474246

C	-3.014443	0.435672	0.981466
C	-1.481045	0.579752	1.035601
C	-1.227160	-1.235354	-0.784945
C	-2.767891	-1.284703	-0.870534
H	-3.447330	1.225769	0.357830
H	-3.099732	-2.291963	-1.180832
C	-0.937382	-0.430792	2.093349
H	-1.777715	-0.821762	2.693105
C	-0.727585	-2.258909	0.269858
H	0.044364	-2.912359	-0.165237
H	-1.282800	1.595376	1.418461
H	-0.879694	-1.558554	-1.780281
C	-0.136815	-1.617232	1.533792
H	0.869116	-1.257266	1.289140
H	-0.013814	-2.381372	2.318014
C	-3.460357	-0.924533	0.443529
H	-4.553680	-0.942119	0.314286
H	-3.234192	-1.701379	1.191402
H	-0.292251	0.087636	2.818536
H	-1.546961	-2.939438	0.559125
H	-3.442447	0.575304	1.991286
H	-3.110905	-0.593623	-1.657804
B	-0.760825	0.331569	-0.461624
C	0.860091	0.747394	-0.346833
C	1.996216	0.103710	-0.721190
C	1.997731	-1.241576	-1.423021
H	0.969232	-1.557240	-1.588618
H	2.434654	-1.095679	-2.427215
C	3.391559	0.682927	-0.529055
H	4.016620	0.421597	-1.399831
H	3.350608	1.778898	-0.505749
C	0.984363	2.191940	0.158002
H	-0.001272	2.666646	0.071177
H	1.646715	2.776373	-0.500095
C	-1.275578	1.322501	-1.739823
H	-0.404174	1.908515	-2.076678
H	-1.494285	0.663082	-2.600388
C	2.770476	-2.380241	-0.745042
H	2.490226	-2.481896	0.312771
H	2.554850	-3.334794	-1.244879
H	3.855516	-2.226136	-0.794799
C	4.116582	0.203056	0.741448
H	4.466136	-0.830769	0.649780
H	4.994341	0.830880	0.951306
H	3.451223	0.244808	1.613520
C	1.465156	2.364845	1.601658
H	0.932864	1.684941	2.278689
H	2.537568	2.159059	1.698506
H	1.292003	3.392355	1.952574
C	-2.435511	2.321773	-1.615219
H	-2.456576	3.030220	-2.458748
H	-3.416147	1.825707	-1.595407
H	-2.358389	2.919961	-0.693073

53

A_Et_conf_4 Eopt -730.485075

C	-3.121019	0.841637	0.199172
C	-1.695005	0.867605	0.774002
C	-1.053464	-1.268629	-0.521856
C	-2.497752	-1.379272	-1.058920
H	-3.183147	1.596088	-0.598842
H	-2.834802	-2.432425	-1.037890
C	-1.601858	0.059912	2.087552
H	-2.360168	0.410805	2.812099
C	-0.866648	-2.019425	0.805177
H	0.194516	-1.949735	1.100349
H	-1.499361	1.923200	1.044657
H	-0.434160	-1.799274	-1.262542
C	-1.722044	-1.464813	1.947917
H	-1.418734	-1.934572	2.896630
H	-2.768521	-1.758221	1.798924
C	-3.577761	-0.523700	-0.356881
H	-4.409363	-0.360484	-1.060299
H	-4.015889	-1.104320	0.464074
H	-0.627414	0.253078	2.559042
H	-1.084506	-3.096881	0.686935
H	-3.855774	1.158735	0.962069
H	-2.475715	-1.105306	-2.123436
B	-0.612730	0.335239	-0.378698
C	0.976230	0.555233	0.117082
C	2.057935	-0.062195	-0.425408

C 1.998249 -0.935484 -1.663764
H 1.019494 -0.843017 -2.134793
H 2.729971 -0.541547 -2.390970
C 3.465257 0.042938 0.155996
H 4.199992 -0.189804 -0.630692
H 3.687524 1.068425 0.478690
C 1.276659 1.537128 1.257369
H 2.042695 1.133920 1.933776
H 0.391142 1.693844 1.878023
C -0.869016 1.113562 -1.828675
H -0.246664 0.654711 -2.617836
H -1.904960 0.951768 -2.166991
C 2.314277 -2.415763 -1.429069
H 1.646124 -2.839843 -0.666687
H 2.185744 -2.993257 -2.354947
H 3.349298 -2.560261 -1.091464
C 3.704751 -0.907719 1.336605
H 4.726882 -0.807432 1.728915
H 3.003695 -0.702367 2.156399
H 3.556648 -1.952994 1.038126
C 1.732116 2.925655 0.785189
H 2.531350 2.860406 0.034340
H 0.898633 3.477770 0.335405
H 2.105089 3.521032 1.630787
C -0.604127 2.621137 -1.844965
H 0.466503 2.830764 -1.713802
H -0.915152 3.101966 -2.786213
H -1.138003 3.132188 -1.027468
53
A_Et_conf_5 Eopt -730.480255
C -3.059728 0.526556 0.645951
C -1.561396 0.658011 0.993423
C -0.961183 -1.241100 -0.632184
C -2.418833 -1.275140 -1.131600
H -3.361643 1.417938 0.085954
H -2.714787 -2.304021 -1.407130
C -1.208662 -0.277693 2.171155
H -1.806716 -0.019038 3.064260
C -0.711747 -2.183296 0.558316
H 0.374587 -2.207527 0.745061
H -1.435851 1.692874 1.363020
H -0.369346 -1.649607 -1.466844
C -1.392405 -1.768202 1.868942
H -0.972591 -2.358662 2.698160
H -2.459567 -2.018797 1.843895
C -3.477802 -0.722884 -0.161133
H -4.390828 -0.487994 -0.730767
H -3.775608 -1.522402 0.528026
H -0.156328 -0.137217 2.456612
H -1.004841 -3.221000 0.312290
H -3.662467 0.548803 1.573200
H -2.460802 -0.696997 -2.067743
B -0.547202 0.340136 -0.298984
C 1.045751 0.596620 0.155829
C 2.147424 0.086926 -0.450313
C 2.096990 -0.758395 -1.708719
H 1.103970 -0.699617 -2.157469
H 2.787978 -0.302854 -2.439492
C 3.575657 0.339518 0.030314
H 4.277713 0.073816 -0.774564
H 3.747704 1.407235 0.230283
C 1.274184 1.677217 1.212450
H 2.278679 1.638019 1.651725
H 0.579625 1.570033 2.052328
C -0.762765 1.285963 -1.685479
H 0.136009 1.909975 -1.813217
H -0.742512 0.609920 -2.561051
C 2.503649 -2.222270 -1.521588
H 1.839195 -2.721958 -0.804405
H 2.455115 -2.771386 -2.472283
H 3.532060 -2.304874 -1.142756
C 3.941842 -0.474244 1.276036
H 4.978893 -0.289331 1.591387
H 3.277510 -0.226787 2.115086
H 3.830039 -1.549494 1.080495
C 1.059056 3.073504 0.615078
H 1.771297 3.265983 -0.200372
H 0.046306 3.163773 0.200238
H 1.189057 3.857102 1.374659
C -1.970240 2.217015 -1.857389
H -2.091224 2.888026 -0.992285
H -1.871002 2.856299 -2.749458
H -2.912381 1.660454 -1.966452
53
A_Et_conf_6 Eopt -730.481804
C 3.019120 0.965992 0.316261
C 1.595925 1.314572 -0.145203
C 1.010798 -1.179226 -0.394324
C 2.503223 -1.542840 -0.194171
H 3.036119 0.966006 1.416409
H 2.811500 -2.275058 -0.963858
C 1.516983 1.466061 -1.681649
H 2.322680 2.130429 -2.046990
C 0.704691 -0.957179 -1.885703
H -0.362547 -0.708206 -1.999143
H 1.367484 2.310318 0.282388
H 0.456324 -2.083142 -0.096233
C 1.543982 0.164603 -2.503719
H 1.180807 0.380034 -3.520749
H 2.573756 -0.190904 -2.632920
C 3.531066 -0.387368 -0.203403
H 4.409588 -0.690156 0.388089
H 3.913295 -0.241708 -1.221543
H 0.578070 1.976588 -1.937829
H 0.874203 -1.882870 -2.465741
H 3.740892 1.743564 0.005376
H 2.597227 -2.074882 0.758931
B 0.542981 0.177157 0.464498
C -1.056440 0.597899 0.202959
C -2.109147 -0.243549 0.351533
C -1.958563 -1.670681 0.839656
H -0.951806 -1.817902 1.238536
H -2.650988 -1.812867 1.688097
C -3.558015 0.153656 0.084541
H -4.226405 -0.559594 0.591298
H -3.786286 1.136344 0.522035
C -1.379543 2.072428 -0.020361
H -2.324169 2.223510 -0.559496
H -0.608849 2.561153 -0.623927
C 0.698022 0.084181 2.143599
H 1.075617 1.058756 2.507417
H -0.313597 0.010140 2.576281
C -2.266166 -2.742546 -0.209673
H -1.620994 -2.619716 -1.090618
H -2.105284 -3.751532 0.195023
H -3.310589 -2.683242 -0.546102
C -3.914553 0.174481 -1.406389
H -4.968047 0.445666 -1.566354
H -3.288735 0.895880 -1.948743
H -3.742927 -0.810395 -1.860926
C -1.455235 2.811428 1.321236
H -2.252115 2.394073 1.953418
H -0.510064 2.710437 1.870867
H -1.654516 3.883514 1.181581
C 1.534658 -1.012725 2.816701
H 2.606625 -0.922617 2.589789
H 1.435601 -0.987487 3.913970
H 1.218372 -2.015047 2.487838
53
A_Et_conf_7 Eopt -730.478858
C -3.038319 -0.570168 0.877375
C -1.580655 -1.062898 0.824548
C -1.161102 0.530730 -1.162787
C -2.660148 0.897736 -1.157538
H -3.111903 0.284784 1.569727
H -3.005035 1.055126 -2.195865
C -1.483922 -2.292526 -0.121718
H -2.493036 -2.640509 -0.398722
C -0.934762 -0.691250 -2.099262
H -0.076769 -0.498989 -2.762901

H	-1.327643	-1.390027	1.848596
H	-0.666039	1.401224	-1.620229
C	-0.670281	-2.040603	-1.400973
H	0.394218	-2.085054	-1.137526
H	-0.841642	-2.860687	-2.116156
C	-3.556858	-0.148491	-0.498129
H	-4.586690	0.233268	-0.419375
H	-3.615248	-1.035440	-1.148971
H	-1.028273	-3.147755	0.400745
H	-1.802515	-0.803594	-2.773690
H	-3.702152	-1.354761	1.283976
H	-2.804163	1.856001	-0.646273
B	-0.576433	0.189491	0.361433
C	1.037051	-0.282316	0.404704
C	2.089653	0.271631	-0.247310
C	1.978211	1.351372	-1.313928
H	2.897188	1.339404	-1.921008
H	1.160723	1.137575	-2.007831
C	3.544683	-0.099191	0.016127
H	4.157973	0.811650	-0.082053
H	3.699532	-0.450748	1.043153
C	1.363583	-1.376198	1.420336
H	2.308089	-1.884605	1.183110
H	0.594360	-2.156783	1.370856
C	-0.686640	1.435377	1.505029
H	-1.079806	1.003781	2.444368
H	0.339085	1.745138	1.763984
C	1.797178	2.755052	-0.733571
H	0.835656	2.829077	-0.212193
H	2.589595	2.983732	-0.005870
H	1.822110	3.524611	-1.518268
C	4.068537	-1.148365	-0.968018
H	3.516310	-2.091983	-0.853654
H	3.931939	-0.810169	-2.005218
H	5.137558	-1.355557	-0.816837
C	1.431271	-0.862338	2.861409
H	2.201451	-0.084022	2.962389
H	0.474183	-0.421027	3.165947
H	1.671246	-1.672476	3.564735
C	-1.477748	2.727177	1.244238
H	-2.563849	2.565964	1.289783
H	-1.241259	3.504145	1.988442
H	-1.261429	3.152864	0.252473
53			
A_Et_conf_8		Eopt	-730.486465
C	3.108581	0.498179	-0.876056
C	1.772121	-0.241736	-1.077709
C	1.235196	0.031860	1.442267
C	2.592440	0.740073	1.633423
H	2.995245	1.559215	-1.144436
H	3.016981	0.487838	2.622459
C	2.009974	-1.764725	-0.830848
H	3.088251	-1.956720	-0.700633
C	1.396693	-1.498252	1.654978
H	0.639569	-1.857021	2.369633
H	1.493200	-0.112297	-2.139114
H	0.595944	0.401642	2.258925
C	1.271148	-2.345231	0.381408
H	0.205021	-2.437744	0.137055
H	1.630057	-3.368167	0.578163
C	3.628257	0.380967	0.562735
H	4.530868	0.998711	0.690814
H	3.954280	-0.657723	0.732585
H	1.730914	-2.351289	-1.719539
H	2.370770	-1.712147	2.131314
H	3.881158	0.095028	-1.556159
H	2.439490	1.829174	1.644143
B	0.608950	0.411541	-0.063953
C	-0.912406	-0.231519	-0.360214
C	-2.033301	0.011798	0.364709
C	-2.098915	0.961186	1.544754
H	-2.635102	0.474121	2.378251
H	-1.095508	1.190865	1.906154
C	-3.375424	-0.661671	0.100603
H	-4.189913	-0.005681	0.441931
H	-3.551682	-0.823341	-0.970038
C	-1.095980	-1.121799	-1.593321
H	-1.833999	-1.915843	-1.410720
H	-0.163071	-1.639043	-1.831014
C	0.533369	2.067675	-0.228750
H	-0.083331	2.488278	0.584902
H	1.524836	2.519528	-0.075909
C	-2.817383	2.272307	1.204809
H	-2.300883	2.786826	0.382949
H	-3.854469	2.097281	0.888231
H	-2.842286	2.948609	2.070829
C	-3.488482	-1.996622	0.844812
H	-2.717523	-2.699667	0.500055
H	-3.340348	-1.850297	1.924132
H	-4.472270	-2.464717	0.698008
C	-1.513063	-0.340736	-2.844016
H	-2.433822	0.233917	-2.669563
H	-0.728483	0.369748	-3.134491
H	-1.686717	-1.017162	-3.693022
C	-0.006589	2.595896	-1.560234
H	-1.040356	2.262048	-1.731826
H	-0.003780	3.696163	-1.617859
H	0.594793	2.226259	-2.406551
53			
A_Et_conf_9		Eopt	-730.483993
C	2.915594	-1.225728	-0.299890
C	1.629610	-1.093911	0.534168
C	1.199202	1.281363	-0.366879
C	2.566621	1.251596	-1.088613
H	2.692039	-1.813363	-1.201739
H	3.090848	2.215622	-0.950410
C	1.945053	-0.480771	1.919604
H	2.790251	-1.020491	2.386583
C	1.308539	1.839181	1.059335
H	0.303706	1.837550	1.513334
H	1.288722	-2.125806	0.735619
H	0.597362	2.004394	-0.937975
C	2.248541	1.025957	1.951835
H	2.173322	1.382124	2.991212
H	3.285974	1.219715	1.652395
C	3.549063	0.118809	-0.708917
H	4.234497	-0.051017	-1.554084
H	4.193366	0.459023	0.111227
H	1.091832	-0.645120	2.591177
H	1.646174	2.891972	1.054035
H	3.679588	-1.804635	0.250995
H	2.367737	1.186341	-2.167580
B	0.499396	-0.232430	-0.352067
C	-1.038111	-0.260872	0.331160
C	-2.059712	0.569931	-0.008557
C	-1.967017	1.633929	-1.088086
H	-1.115147	1.445861	-1.742750
H	-2.869655	1.564441	-1.720571
C	-3.454953	0.542035	0.613531
H	-3.410824	0.440497	1.706426
H	-3.938238	1.512392	0.429287
C	-1.338528	-1.323701	1.387899
H	-2.246826	-1.082508	1.955851
H	-0.529340	-1.351210	2.126561
C	0.411869	-0.804314	-1.918634
H	1.373381	-0.641023	-2.431084
H	-0.311772	-0.194294	-2.486273
C	-1.878257	3.058962	-0.531214
H	-1.834714	3.801021	-1.340844
H	-2.746363	3.301542	0.096908
H	-0.978311	3.174035	0.086920
C	-4.357936	-0.553327	0.040057
H	-3.998719	-1.548411	0.329434
H	-5.392803	-0.449255	0.396505
H	-4.369332	-0.509527	-1.058564
C	-1.495144	-2.753046	0.853624
H	-0.565278	-3.117147	0.403909
H	-1.764872	-3.436827	1.671142
H	-2.278234	-2.815893	0.086753
C	0.027878	-2.272286	-2.127806

H 0.677170 -2.949537 -1.550342
H -1.004963 -2.461530 -1.805899
H 0.098562 -2.579634 -3.183530
41
A_Me_conf_1 Eopt -573.426348
C 2.082024 -1.439095 -0.745607
C 0.665831 -1.251407 -0.161720
C 1.092131 1.285467 0.029257
C 2.500106 1.118720 -0.568096
H 1.976074 -1.567160 -1.834016
H 3.215316 1.815599 -0.092671
C 0.633767 -1.365258 1.372037
H 1.039962 -2.337550 1.707911
C 1.077889 1.158732 1.564942
H 0.077131 1.447225 1.923032
H 0.092443 -2.108973 -0.544785
H 0.795949 2.325357 -0.198000
C 1.375361 -0.244663 2.115145
H 1.102080 -0.275328 3.181557
H 2.454919 -0.436022 2.088857
C 3.087408 -0.301923 -0.485946
H 3.917040 -0.388983 -1.205484
H 3.550348 -0.443957 0.498307
H -0.420960 -1.346589 1.692602
H 1.782690 1.874032 2.028798
H 2.529224 -2.382426 -0.380444
H 2.454372 1.419218 -1.626214
B 0.030991 0.202032 -0.658167
C -1.527661 0.550865 -0.180514
C -2.562356 -0.311166 -0.035451
C -2.476089 -1.785992 -0.375939
H -3.465076 -2.155328 -0.687107
H -1.776681 -1.978809 -1.193745
C -3.946461 0.060884 0.463655
H -4.294538 -0.701887 1.178597
H -4.687068 0.073048 -0.354521
H -3.987788 1.027197 0.975716
C -1.806966 2.039488 -0.033173
H -2.870481 2.302236 -0.084316
H -1.405621 2.456534 0.903498
H -1.303061 2.586297 -0.843894
H -2.167909 -2.402830 0.485810
C 0.015059 0.332923 -2.312729
H -0.636532 -0.434808 -2.768532
H -0.387979 1.308779 -2.640008
H 1.000925 0.229520 -2.795276
41
A_Me_conf_2 Eopt -573.419820
C -2.568543 1.116291 -0.319762
C -1.088429 1.283639 0.112740
C -0.670929 -1.258941 -0.119699
C -2.147829 -1.473470 -0.558118
H -2.829934 1.911959 -1.034044
H -2.693094 -1.995204 0.248029
C -0.981296 1.123907 1.642635
H -1.608056 1.875109 2.157439
C -0.575486 -1.363167 1.413573
H 0.478117 -1.274840 1.724567
H -0.808483 2.325006 -0.116805
H -0.112129 -2.112080 -0.526978
C -1.383359 -0.272531 2.117895
H -1.262093 -0.351235 3.209235
H -2.456942 -0.430426 1.920206
C -2.950638 -0.225105 -0.980863
H -2.844556 -0.108797 -2.065834
H -4.022726 -0.414801 -0.817443
H 0.055245 1.307927 1.967289
H -0.926140 -2.352666 1.760003
H -3.226151 1.288656 0.550560
H -2.170955 -2.169927 -1.410181
B -0.062231 0.202692 -0.637103
C 1.511745 0.551743 -0.202314
C 2.551485 -0.307541 -0.078615
C 2.457081 -1.787362 -0.395277
H 2.165855 -2.390547 0.481689
H 1.741012 -1.992060 -1.195506
C 3.949204 0.071902 0.375775
H 4.665872 0.073717 -0.463501
H 4.318358 -0.681439 1.090046
H 4.004587 1.044770 0.873856
C 1.793322 2.041990 -0.072918
H 2.853473 2.305929 -0.168265
H 1.255849 2.585684 -0.863654
H 1.429856 2.460619 0.878511
H 3.438787 -2.162577 -0.721475
C -0.065309 0.347072 -2.292694
H 0.390543 1.300750 -2.615265
H 0.542802 -0.450696 -2.756975
H -1.054060 0.303152 -2.774142
54
B_Et_conf_1 Eopt -1028.042225
C -1.841406 -2.311673 -0.413616
C -1.661335 -0.914187 -1.055502
C -2.551016 0.155833 1.143357
C -2.717797 -1.264956 1.740373
H -0.881597 -2.645566 0.011122
H -3.580546 -1.261508 2.423856
C -2.968709 -0.464050 -1.785240
H -3.666849 -1.313091 -1.843158
C -3.857814 0.569568 0.387398
H -4.235811 1.514093 0.800245
H -0.862791 -0.996219 -1.798275
H -2.390454 0.850487 1.977571
C -3.671928 0.728381 -1.126075
H -3.069148 1.632205 -1.313851
H -4.644213 0.904127 -1.606698
C -2.920681 -2.338414 0.669801
H -2.949395 -3.330642 1.140810
H -3.905396 -2.198667 0.199711
H -2.725905 -0.195570 -2.821758
H -4.642001 -0.177458 0.577662
H -2.093969 -3.036629 -1.202289
H -1.841737 -1.519297 2.354632
B -1.358323 0.147561 0.088586
C -0.079760 1.149617 0.090725
C 1.182227 0.382656 0.656207
C 2.318444 1.321717 1.116442
H 2.299639 2.233092 0.515738
H 2.031469 1.628527 2.134539
C 0.717620 -0.545925 1.790971
H 0.061903 -1.313657 1.361689
H 0.086518 0.070069 2.450270
C -0.346078 2.356902 1.039963
H 0.540423 2.995474 1.104362
H -0.523812 1.986657 2.057731
C 0.052977 1.670192 -1.363113
H 0.175235 0.817790 -2.039161
H -0.914324 2.116832 -1.634975
I 2.004078 -0.998831 -0.899713
C 1.766079 -1.236867 2.651678
H 2.374545 -0.515666 3.210135
H 1.262548 -1.880256 3.383483
H 2.432138 -1.868799 2.051283
C 3.763143 0.826383 1.145458
H 3.875474 -0.165701 1.591741
H 4.182091 0.786030 0.133293
H 4.366786 1.533804 1.729012
C -1.510960 3.258454 0.636897
H -1.696481 3.997334 1.426860
H -1.293511 3.813069 -0.284780
H -2.441764 2.702848 0.483520
C 1.151335 2.687300 -1.667237
H 2.148934 2.232916 -1.608485
H 1.027160 3.069169 -2.688483
H 1.124255 3.548875 -0.986421
54
B_Et_conf_10 Eopt -1028.039053
C 2.420828 -0.792383 -1.985146
C 1.612652 -1.136953 -0.696825
C 2.897606 0.732198 0.567297

C	3.685393	1.049504	-0.740796
H	1.785000	-0.958376	-2.865490
H	4.661847	0.542222	-0.720991
C	2.354931	-2.184754	0.173594
H	2.475485	-3.110102	-0.409863
C	3.619910	-0.361558	1.396945
H	3.087428	-0.513574	2.348762
H	0.662938	-1.589424	-0.991188
H	2.872554	1.637174	1.183995
C	3.717316	-1.697835	0.663519
H	4.166654	-2.451892	1.324354
H	4.400023	-1.597426	-0.193227
C	2.934359	0.650933	-2.014782
H	2.069757	1.325708	-2.134832
H	3.572620	0.808273	-2.895243
H	1.729610	-2.444798	1.042688
H	4.627339	-0.003651	1.659041
H	3.270513	-1.483751	-2.090499
H	3.903001	2.125192	-0.781109
B	1.452737	0.174700	0.187711
C	0.092603	0.778556	0.802665
C	-1.179629	0.697754	-0.126809
C	-2.308102	1.642120	0.339857
H	-2.268901	1.736233	1.426879
H	-2.023019	2.629767	-0.055801
C	-0.771927	0.960103	-1.586455
H	-0.124382	0.145857	-1.925392
H	-0.139720	1.857455	-1.560575
C	0.335419	2.249127	1.266041
H	1.119203	2.191923	2.030869
H	-0.539582	2.643932	1.792323
C	0.014130	-0.078458	2.108469
H	-0.007056	-1.144257	1.849676
H	0.956339	0.084387	2.652256
I	-2.032304	-1.389188	-0.139278
C	-1.865464	1.183907	-2.622589
H	-1.401943	1.323783	-3.606684
H	-2.542731	0.323301	-2.691761
H	-2.459126	2.080464	-2.407521
C	-3.765232	1.367630	-0.031082
H	-3.906474	1.079677	-1.076030
H	-4.181770	0.568818	0.592838
H	-4.353322	2.275956	0.154624
C	0.773482	3.274244	0.220456
H	1.567441	2.884953	-0.432298
H	-0.054903	3.605190	-0.418156
H	1.167171	4.167723	0.721768
C	-1.132071	0.203167	3.080989
H	-2.106343	-0.052377	2.647703
H	-1.000371	-0.411675	3.980230
H	-1.154089	1.252992	3.399720

54

B_Et_conf_11 Eopt -1028.039153

C	2.527637	-0.483917	-1.972512
C	1.544024	-0.894241	-0.848913
C	2.773224	0.695909	0.776332
C	3.712334	1.139322	-0.369889
H	2.017417	0.251147	-2.616160
H	4.706334	1.372505	0.042306
C	2.021344	-2.103650	-0.007067
H	2.164124	-2.972471	-0.667999
C	3.297849	-0.529368	1.568310
H	2.667782	-0.643117	2.463832
H	0.614030	-1.200716	-1.338660
H	2.721614	1.529308	1.489854
C	3.296715	-1.864627	0.809901
H	3.408734	-2.681158	1.537128
H	4.174005	-1.931512	0.158514
C	3.859720	0.127456	-1.513670
H	4.330102	0.629029	-2.371148
H	4.557036	-0.664171	-1.220909
H	1.205839	-2.376490	0.681836
H	4.313900	-0.315706	1.935689
H	2.726522	-1.355866	-2.614835
H	3.327911	2.085224	-0.782293
B	1.363086	0.316894	0.154101
C	0.000914	1.007504	0.657379
C	-1.257465	0.712237	-0.241743
C	-2.490244	1.559539	0.120031
H	-2.756940	1.414260	1.169376
H	-2.168521	2.606282	0.020961
C	-0.937643	0.902431	-1.749480
H	0.146128	0.954231	-1.881443
H	-1.309854	1.903715	-2.001500
C	0.218558	2.543102	0.803507
H	1.066224	2.666021	1.486904
H	-0.635892	2.994818	1.323074
C	-0.038098	0.444574	2.119842
H	0.073653	-0.648926	2.103796
H	0.866121	0.829038	2.609839
I	-1.901712	-1.411526	0.137462
C	-1.494886	-0.060963	-2.793722
H	-1.290870	0.344204	-3.793626
H	-1.025434	-1.048976	-2.728050
H	-2.577434	-0.197719	-2.696499
C	-3.733155	1.387204	-0.747585
H	-3.543096	1.654137	-1.794413
H	-4.117179	0.360357	-0.716037
H	-4.524211	2.051456	-0.378753
C	0.518546	3.348874	-0.459666
H	1.270339	2.853885	-1.090137
H	-0.372047	3.515294	-1.076088
H	0.912489	4.335141	-0.184595
C	-1.222790	0.787242	3.024381
H	-1.435731	1.863645	3.038914
H	-2.132916	0.253302	2.730584
H	-0.984403	0.484467	4.051952

54

B_Et_conf_12 Eopt -1028.039091

C	-2.274011	-0.843559	2.052909
C	-1.377397	-1.062787	0.810060
C	-2.775281	0.687931	-0.477042
C	-3.662564	0.891695	0.774398
H	-1.749202	-0.154817	2.735045
H	-4.694228	1.108340	0.456174
C	-1.891023	-2.166221	-0.148042
H	-1.989072	-3.112901	0.405801
C	-3.278756	-0.426359	-1.429412
H	-2.669233	-0.388130	-2.346470
H	-0.403479	-1.397935	1.181546
H	-2.817825	1.629224	-1.037559
C	-3.210165	-1.853002	-0.867198
H	-3.336474	-2.563783	-1.695917
H	-4.056323	-2.033609	-0.196571
C	-3.669083	-0.269969	1.775627
H	-4.100526	0.082677	2.723111
H	-4.339201	-1.062487	1.427557
H	-1.110311	-2.336644	-0.906346
H	-4.310985	-0.201854	-1.740556
H	-2.368701	-1.793470	2.601367
H	-3.310717	1.798156	1.291815
B	-1.308612	0.285151	-0.019976
C	-0.007396	1.095841	-0.489120
C	1.322031	0.706792	0.262591
C	2.498223	1.617517	-0.119999
H	2.640756	1.607576	-1.207696
H	2.179763	2.636075	0.144956
C	1.140530	0.686363	1.800668
H	0.071763	0.674776	2.031287
H	1.506589	1.660504	2.149654
C	-0.276975	2.623747	-0.364911
H	-1.191336	2.818251	-0.932239
H	0.508441	3.188682	-0.885478
C	0.086915	0.750982	-2.024596
H	1.142400	0.785695	-2.320191
H	-0.211080	-0.297273	-2.176576
I	1.968681	-1.343260	-0.445190
C	1.819090	-0.382063	2.653604
H	1.702889	-0.115989	3.712453
H	1.366975	-1.368907	2.502205

H	2.890058	-0.466857	2.439228
C	3.832957	1.371010	0.575176
H	4.569519	2.098559	0.212997
H	3.753143	1.498394	1.662121
H	4.225027	0.367347	0.372124
C	-0.485863	3.208495	1.031040
H	0.448082	3.316259	1.593424
H	-0.931511	4.207451	0.947040
H	-1.168099	2.590469	1.631957
C	-0.690868	1.614545	-3.016797
H	-0.314975	2.644073	-3.045511
H	-0.565817	1.192649	-4.022180
H	-1.765558	1.650778	-2.807344

54

B_Et_conf_13 Eopt -1028.037919

C	-2.230237	-0.490516	2.205319
C	-1.363443	-0.957164	1.004840
C	-2.816513	0.536592	-0.540582
C	-3.636347	0.973203	0.701466
H	-1.747894	0.365242	2.705551
H	-4.666516	1.197701	0.386023
C	-1.972143	-2.222027	0.327985
H	-2.788112	-2.621957	0.949205
C	-3.418823	-0.748824	-1.185495
H	-3.641093	-0.556036	-2.243603
H	-0.381483	-1.219468	1.406074
H	-2.882975	1.356985	-1.261630
C	-2.491383	-1.965169	-1.091003
H	-1.624340	-1.796049	-1.751509
H	-2.999589	-2.859878	-1.476292
C	-3.645251	-0.082339	1.804395
H	-4.185663	0.298279	2.682047
H	-4.201385	-0.967289	1.462502
H	-1.204828	-3.006335	0.283703
H	-4.383104	-0.987371	-0.712065
H	-2.266143	-1.299749	2.949898
H	-3.224048	1.912681	1.102848
B	-1.330284	0.229539	-0.053323
C	-0.051025	1.017637	-0.630278
C	1.293969	0.755077	0.153110
C	2.458052	1.625166	-0.349525
H	2.629764	1.452107	-1.417340
H	2.113542	2.665156	-0.250962
C	1.129541	0.931770	1.682918
H	0.065822	0.928586	1.928749
H	1.476520	1.950659	1.896086
C	-0.372997	2.542608	-0.669097
H	-1.323009	2.636839	-1.203548
H	0.365190	3.053325	-1.305077
C	0.094244	0.561585	-2.127218
H	1.100608	0.842429	-2.462194
H	0.072393	-0.532414	-2.172206
I	1.960487	-1.362044	-0.286336
C	1.841913	-0.000088	2.659727
H	1.731332	0.402260	3.675340
H	1.410503	-1.007373	2.647674
H	2.911918	-0.091119	2.444442
C	3.785401	1.512935	0.393726
H	4.189691	0.493914	0.357986
H	4.518825	2.180748	-0.074204
H	3.692592	1.812428	1.445026
C	-0.523713	3.307239	0.645194
H	0.437077	3.522830	1.125091
H	-1.012839	4.269892	0.451160
H	-1.143402	2.761276	1.369539
C	-0.894515	1.081511	-3.170323
H	-0.598165	0.686558	-4.150656
H	-1.920250	0.745811	-2.986234
H	-0.895682	2.174786	-3.249974

54

B_Et_conf_15 Eopt -1028.037597

C	-1.758966	-2.203981	-0.128355
C	-1.725591	-0.871944	-0.917863
C	-2.346797	0.351473	1.272044
C	-2.650771	-1.006461	1.947461
H	-0.755855	-2.395392	0.286752
H	-3.560795	-0.914038	2.561169
C	-3.101860	-0.493662	-1.528885
H	-3.519345	-1.373728	-2.043152
C	-3.575909	0.965392	0.548218
H	-3.288517	1.938852	0.118032
H	-1.030758	-1.000166	-1.755325
H	-2.059609	1.050890	2.067947
C	-4.154385	0.074789	-0.557211
H	-4.888400	0.655262	-1.133471
H	-4.724183	-0.737376	-0.093107
C	-2.778152	-2.219276	1.011546
H	-2.641598	-3.135822	1.602444
H	-3.789026	-2.286151	0.594685
H	-2.917272	0.251623	-2.315379
H	-4.359208	1.184483	1.289444
H	-1.965229	-3.033931	-0.821374
H	-1.832701	-1.213633	2.654809
B	-1.269537	0.236647	0.114672
C	0.027457	1.206410	-0.037615
C	1.311509	0.465730	0.493830
C	2.559213	1.360336	0.496285
H	2.754131	1.725388	-0.518533
H	2.292840	2.232759	1.103438
C	1.056694	-0.124232	1.902303
H	-0.021877	-0.241631	2.052503
H	1.359251	0.666518	2.605813
C	-0.244307	2.472263	0.847294
H	-0.156979	2.192960	1.903886
H	-1.305357	2.712229	0.710928
C	0.137673	1.634309	-1.518646
H	1.094893	2.136862	-1.712081
H	0.139958	0.738041	-2.147216
I	1.844005	-1.195000	-0.925312
C	1.720373	-1.430456	2.330219
H	1.264051	-2.288059	1.822136
H	2.794738	-1.451089	2.122640
H	1.577489	-1.564215	3.410377
C	3.842187	0.785416	1.087959
H	4.644696	1.526593	0.990089
H	3.730642	0.563506	2.156446
H	4.165142	-0.127638	0.573687
C	0.555639	3.766236	0.589393
H	1.114960	3.744983	-0.353076
H	-0.122007	4.627735	0.546467
H	1.275633	3.971236	1.389805
C	-1.000258	2.544060	-1.982607
H	-1.984134	2.119095	-1.740418
H	-0.951172	3.539008	-1.523877
H	-0.961478	2.678192	-3.070551

54

B_Et_conf_16 Eopt -1028.038327

C	-1.679858	-2.309748	-0.453585
C	-1.524764	-0.900107	-1.065031
C	-2.410489	0.143451	1.128483
C	-2.652356	-1.269665	1.707966
H	-0.690157	-2.633393	-0.096168
H	-3.591388	-1.263847	2.283458
C	-2.798731	-0.410387	-1.805767
H	-3.079000	-1.156749	-2.565167
C	-3.640926	0.689743	0.350215
H	-3.439888	1.727625	0.048352
H	-0.730067	-0.963749	-1.817206
H	-2.235853	0.807725	1.984523
C	-4.006768	-0.108189	-0.907658
H	-4.742635	0.466915	-1.487197
H	-4.513676	-1.037358	-0.629004
C	-2.695536	-2.424702	0.692886
H	-2.517432	-3.368264	1.227638
H	-3.704142	-2.515915	0.276943
H	-2.539096	0.503986	-2.361128
H	-4.506151	0.732403	1.029401
H	-1.955306	-3.020083	-1.248568
H	-1.857763	-1.471860	2.440634
B	-1.232856	0.168163	0.069976

C 0.038106 1.176139 0.061620
C 1.247847 0.364406 0.691325
C 2.459995 1.179217 1.176506
H 2.127091 1.736291 2.065488
H 3.193345 0.453662 1.545134
C 0.726586 -0.523740 1.837700
H 0.062876 -1.285703 1.409959
H 0.091247 0.115921 2.469386
C -0.129820 2.426298 0.988011
H 0.684627 3.120407 0.744236
H 0.040692 2.130933 2.030711
C 0.356988 1.625817 -1.389378
H 1.389492 1.985381 -1.444229
H 0.319464 0.746862 -2.040261
I 2.120752 -1.089272 -0.780583
C 1.745470 -1.221736 2.730764
H 2.303673 -0.510334 3.350024
H 1.222615 -1.907790 3.408073
H 2.462663 -1.810044 2.144544
C 3.190617 2.128770 0.232978
H 3.499965 1.618401 -0.688434
H 2.595727 3.006333 -0.042109
H 4.097623 2.490409 0.733230
C -1.442814 3.223231 0.979543
H -2.199131 2.776574 1.631145
H -1.244680 4.231112 1.367623
H -1.886195 3.341493 -0.010903
C -0.545860 2.697539 -1.995961
H -1.610693 2.438430 -1.919791
H -0.401110 3.676218 -1.521841
H -0.308473 2.810564 -3.061179
54
B_Et_conf_17 Eopt -1028.039076
C -1.560670 -2.319103 -0.464465
C -1.554489 -0.892035 -1.075846
C -2.390629 0.022458 1.209568
C -2.440452 -1.427347 1.762496
H -0.555809 -2.572271 -0.095837
H -3.294926 -1.503640 2.451883
C -2.957116 -0.577861 -1.692591
H -3.561378 -1.496567 -1.710397
C -3.774169 0.357207 0.559099
H -4.169597 1.280695 1.002310
H -0.802201 -0.875100 -1.872549
H -2.236807 0.693093 2.064000
C -3.731822 0.525515 -0.962728
H -3.257272 1.490963 -1.189327
H -4.755250 0.586759 -1.358049
C -2.584396 -2.479563 0.661381
H -2.500071 -3.484553 1.097318
H -3.597518 -2.419207 0.237854
H -2.833911 -0.282350 -2.742574
H -4.494042 -0.433140 0.817695
H -1.781433 -3.041886 -1.264603
H -1.548385 -1.639730 2.365297
B -1.251449 0.142027 0.093190
C 0.012475 1.173157 0.155962
C 1.267420 0.385993 0.712421
C 2.465614 1.227386 1.175888
H 2.155919 1.713627 2.115075
H 3.255077 0.523220 1.458915
C 0.838157 -0.567327 1.841223
H 0.209398 -1.349412 1.404933
H 0.190829 0.006945 2.519706
C -0.279694 2.294235 1.199248
H 0.602247 2.940228 1.290363
H -0.443115 1.854938 2.190189
C 0.314228 1.876503 -1.205774
H 0.760635 2.854124 -0.988406
H 1.081666 1.317065 -1.748604
I 2.117849 -0.994845 -0.849137
C 1.928539 -1.233695 2.672249
H 1.471956 -1.971341 3.343292
H 2.655922 -1.758805 2.040222
H 2.468197 -0.511738 3.295733
C 3.074329 2.265617 0.240881
H 4.036353 2.595047 0.652042
H 3.262658 1.846176 -0.755958
H 2.444506 3.153854 0.127408
C -1.468667 3.192585 0.862381
H -1.242855 3.859525 0.021880
H -2.368174 2.618554 0.606340
H -1.713759 3.825491 1.724627
C -0.836563 2.114960 -2.184115
H -1.244353 1.181373 -2.583006
H -1.657102 2.690405 -1.744597
H -0.458739 2.692222 -3.038549
54
B_Et_conf_19 Eopt -1028.039740
C -1.834088 -2.168048 0.215932
C -1.763126 -0.971255 -0.765222
C -2.264680 0.624389 1.209638
C -2.627590 -0.589020 2.098092
H -0.826723 -2.383792 0.603375
H -3.540852 -0.361320 2.670277
C -3.157552 -0.658659 -1.378421
H -3.628519 -1.607121 -1.681048
C -3.474957 1.179374 0.408830
H -3.143556 2.033608 -0.204664
H -1.103117 -1.255072 -1.594041
H -1.920257 1.425679 1.876198
C -4.139921 0.133883 -0.492669
H -4.872633 0.635461 -1.140100
H -4.723168 -0.548203 0.135964
C -2.803021 -1.941645 1.381410
H -2.674305 -2.748468 2.116400
H -3.828696 -2.045403 1.010207
H -2.998300 -0.099561 -2.309600
H -4.218896 1.583883 1.111417
H -2.140860 -3.067974 -0.338534
H -1.829010 -0.696341 2.846931
B -1.230359 0.269786 0.066952
C 0.111452 1.129475 -0.246216
C 1.332934 0.371843 0.405129
C 2.668665 1.147838 0.458821
H 3.493192 0.428013 0.470341
H 2.785116 1.710198 -0.474933
C 0.958786 -0.194476 1.781010
H 0.035929 -0.780305 1.699047
H 0.704991 0.675230 2.406561
C -0.060345 2.542592 0.405039
H 0.048790 2.460172 1.492346
H -1.108585 2.836171 0.256592
C 0.240968 1.326489 -1.772332
H 1.175911 1.855377 -2.011657
H 0.310015 0.348699 -2.258120
I 1.846956 -1.431950 -0.848198
C 1.993137 -1.054452 2.504329
H 1.740042 -1.118691 3.569697
H 2.007580 -2.073059 2.100997
H 3.009628 -0.652879 2.424147
C 2.875308 2.055643 1.680034
H 2.049287 2.743446 1.875795
H 3.033026 1.462790 2.588259
H 3.779119 2.657636 1.521187
C 0.789388 3.708940 -0.113318
H 0.622635 4.584198 0.528606
H 1.863972 3.512806 -0.123879
H 0.505136 3.998454 -1.130405
C -0.941520 2.068500 -2.397830
H -1.005126 3.118367 -2.091952
H -0.867204 2.047300 -3.492187
H -1.894449 1.596634 -2.127040
54
B_Et_conf_2 Eopt -1028.041379
C -2.128515 -2.015563 0.973164
C -1.539552 -1.311113 -0.274749
C -2.715340 0.889128 0.387491
C -2.990742 0.268393 1.783608
H -1.298030 -2.216970 1.665408

H -3.866462 0.760210 2.233306
C -2.481906 -1.398256 -1.504585
H -2.580983 -2.452566 -1.804154
C -3.841343 0.601663 -0.637091
H -3.732962 1.329302 -1.454156
H -0.616285 -1.835910 -0.541144
H -2.677695 1.977502 0.512338
C -3.871181 -0.808236 -1.253925
H -4.421250 -0.765963 -2.204491
H -4.438453 -1.496111 -0.616894
C -3.218181 -1.245341 1.742479
H -3.263523 -1.628627 2.771265
H -4.206485 -1.441489 1.312016
H -2.014026 -0.883008 -2.358526
H -4.816316 0.820617 -0.173355
H -2.516591 -3.004043 0.680802
H -2.141730 0.486381 2.453038
B -1.332860 0.241888 -0.036053
C 0.034746 1.055468 -0.327908
C 1.278838 0.537668 0.476998
C 2.480237 1.494749 0.551987
H 2.160017 2.352548 1.160316
H 3.255736 0.990509 1.139177
C 0.838064 0.095351 1.883231
H 0.186292 -0.778784 1.773725
H 0.201117 0.897794 2.276115
C -0.165043 2.576422 -0.042892
H -1.047414 2.874547 -0.620238
H 0.669519 3.145741 -0.473170
C 0.221898 0.883940 -1.870454
H 1.236733 1.184102 -2.151910
H 0.157589 -0.184914 -2.106219
I 2.183068 -1.336125 -0.420695
C 1.919992 -0.206359 2.913073
H 1.458677 -0.623796 3.815975
H 2.645051 -0.939150 2.536762
H 2.464741 0.697660 3.209983
C 3.133817 2.012210 -0.726293
H 3.452586 1.186622 -1.375378
H 2.484011 2.674641 -1.307952
H 4.028567 2.586388 -0.455221
C -0.399208 3.061830 1.390014
H -1.183680 2.490545 1.900256
H 0.498591 3.021937 2.017559
H -0.723017 4.110228 1.362049
C -0.773888 1.638245 -2.751492
H -0.689392 1.286803 -3.787229
H -1.813659 1.482850 -2.434046
H -0.585742 2.718380 -2.754823
54
B_Et_conf_20 Eopt -1028.039237
C -3.656188 0.684907 0.892177
C -2.299263 0.039789 1.329242
C -1.854284 -0.662167 -1.136694
C -3.241008 -0.058385 -1.535088
H -3.831955 1.593036 1.483704
H -3.994636 -0.858027 -1.572689
C -2.538028 -1.444637 1.702589
H -3.318337 -1.489930 2.477490
C -2.006573 -2.138218 -0.683800
H -1.016670 -2.542278 -0.417776
H -1.949149 0.554498 2.231447
H -1.190174 -0.645095 -2.009877
C -2.952900 -2.300426 0.506163
H -2.994674 -3.357061 0.804342
H -3.975216 -2.031862 0.202105
C -3.724961 1.052966 -0.593426
H -3.097968 1.944278 -0.750533
H -4.749543 1.348279 -0.858545
H -1.635350 -1.874286 2.158327
H -2.369572 -2.738150 -1.532088
H -4.479370 -0.002960 1.137009
H -3.182998 0.344483 -2.554671
B -1.289276 0.157317 0.095457
C 0.045468 1.091184 0.066530
C 1.261820 0.288926 0.659924
C 2.532584 1.064929 1.030313
H 2.255746 1.691118 1.894906
H 3.239861 0.326528 1.422417
C 0.807578 -0.557529 1.863584
H 0.108810 -1.314754 1.494188
H 0.227022 0.103816 2.522358
C -0.296905 2.308577 0.991558
H -0.283666 1.985972 2.039726
H -1.349481 2.562447 0.792080
C 0.326022 1.601643 -1.368801
H 1.256970 2.174143 -1.378883
H 0.500166 0.743132 -2.024035
I 1.936091 -1.268891 -0.826504
C 1.872591 -1.253057 2.703822
H 2.552043 -1.853033 2.085126
H 2.469906 -0.540904 3.284694
H 1.383776 -1.928202 3.416694
C 3.287668 1.898218 -0.000404
H 2.775025 2.824088 -0.274679
H 4.261642 2.177140 0.421047
H 3.475018 1.321514 -0.915396
C 0.505623 3.607341 0.875560
H -0.030485 4.401220 1.411945
H 1.500900 3.534991 1.324161
H 0.625851 3.939569 -0.162204
C -0.770131 2.451656 -2.009971
H -1.105114 3.278038 -1.370389
H -0.393790 2.890034 -2.943065
H -1.645708 1.848564 -2.271180
54
B_Et_conf_21 Eopt -1028.036927
C 3.728344 -0.278250 1.006853
C 2.860187 0.781868 0.260473
C 1.479835 -1.132820 -0.811273
C 2.336133 -2.173901 -0.026521
H 4.034905 0.122879 1.982063
H 3.100461 -2.599822 -0.694338
C 3.460618 1.079195 -1.141328
H 4.468701 1.501012 -1.012020
C 2.139732 -0.800462 -2.176593
H 1.493010 -0.116750 -2.748515
H 2.890098 1.720703 0.824813
H 0.510032 -1.586147 -1.027889
C 3.515872 -0.157261 -2.034672
H 3.902822 0.116946 -3.025644
H 4.226456 -0.886878 -1.620264
C 3.012425 -1.614699 1.230687
H 2.242486 -1.471154 2.004404
H 3.717245 -2.353734 1.636119
H 2.864392 1.852629 -1.650382
H 2.211405 -1.725862 -2.767377
H 4.660096 -0.452072 0.449004
H 1.690669 -3.011279 0.271249
B 1.387497 0.218030 0.026316
C 0.069210 0.881330 0.678668
C -1.280579 0.695733 -0.119600
C -2.386669 1.612502 0.437364
H -2.245193 1.708901 1.519597
H -2.170010 2.605163 0.014726
C -1.032244 0.860860 -1.627123
H -0.474248 -0.009497 -1.980325
H -0.350563 1.714289 -1.737192
C 0.300777 2.394323 0.981141
H 1.207922 2.445566 1.587926
H -0.495970 2.772790 1.634246
C -0.013761 0.157624 2.069053
H -1.037976 0.273227 2.450621
H 0.113437 -0.921795 1.913793
I -2.071031 -1.414744 0.085904
C -2.227475 1.081001 -2.544266
H -2.771379 2.001361 -2.298500
H -1.872708 1.171546 -3.578127
H -2.929247 0.238240 -2.505637
C -3.866125 1.302749 0.207839

H	-4.098384	1.004865	-0.817665
H	-4.206055	0.501922	0.873097
H	-4.452871	2.200956	0.441342
C	0.500897	3.364820	-0.180434
H	1.267328	3.004707	-0.879461
H	-0.414931	3.555249	-0.753057
H	0.843103	4.331358	0.211120
C	0.954026	0.614435	3.161211
H	0.914721	-0.095990	3.996596
H	1.993765	0.662919	2.815021
H	0.686434	1.600773	3.557291

54

B_Et_conf_22 Eopt -1028.037990

C	-2.206859	-2.141384	-0.680965
C	-1.939088	-0.678422	-1.130191
C	-2.168564	0.110705	1.335371
C	-2.376619	-1.376344	1.764335
H	-1.630105	-2.822007	-1.321202
H	-3.450205	-1.564584	1.914697
C	-3.268879	0.077323	-1.429371
H	-3.772680	-0.416277	-2.273665
C	-3.541126	0.758003	1.004708
H	-3.417298	1.837410	0.845063
H	-1.362882	-0.694421	-2.060849
H	-1.745757	0.667832	2.180210
C	-4.205622	0.132425	-0.221862
H	-5.112393	0.695983	-0.481301
H	-4.539871	-0.885786	0.024882
C	-1.819346	-2.407493	0.776174
H	-0.718870	-2.400380	0.834867
H	-2.135544	-3.416454	1.075404
H	-3.045334	1.104803	-1.761561
H	-4.198400	0.651144	1.880711
H	-3.265126	-2.400108	-0.836823
H	-1.904661	-1.539010	2.743361
B	-1.276764	0.188879	0.017899
C	0.031817	1.134173	-0.186286
C	1.278030	0.441928	0.493094
C	2.562046	1.310359	0.570254
H	3.438096	0.659551	0.479846
H	2.592975	1.978973	-0.290668
C	0.968051	-0.153856	1.873112
H	0.067820	-0.768092	1.831543
H	0.715644	0.702068	2.516481
C	-0.211074	2.498326	0.520731
H	0.713305	3.085445	0.515962
H	-0.464254	2.327658	1.574817
C	0.172964	1.408390	-1.709962
H	0.309498	0.461681	-2.237880
H	-0.809174	1.773084	-2.040850
I	1.822962	-1.325103	-0.791108
C	2.067783	-0.984913	2.535507
H	1.901720	-1.022798	3.619257
H	2.056546	-2.014342	2.160117
H	3.072686	-0.583835	2.362016
C	2.750296	2.136344	1.852282
H	3.614261	2.798384	1.713191
H	1.889298	2.762908	2.102956
H	2.960738	1.502084	2.720367
C	-1.292442	3.363520	-0.132561
H	-2.180336	2.787543	-0.419112
H	-1.614605	4.154813	0.555252
H	-0.917918	3.848706	-1.042373
C	1.233299	2.386908	-2.221316
H	1.305564	3.299369	-1.614258
H	2.227763	1.926506	-2.265206
H	0.978335	2.692282	-3.244337

54

B_Et_conf_23 Eopt -1028.037257

C	-3.741661	0.395546	0.449342
C	-2.333262	0.332096	1.128037
C	-1.522675	-1.280906	-0.739449
C	-2.936967	-1.224880	-1.399311
H	-4.142429	1.412633	0.549884
H	-3.498286	-2.132322	-1.130902
C	-2.315574	-0.844905	2.138153
H	-3.134179	-0.694957	2.858373
C	-1.471085	-2.412036	0.322998
H	-0.454003	-2.483304	0.737625
H	-2.185782	1.254238	1.698960
H	-0.786192	-1.525372	-1.512048
C	-2.471626	-2.205736	1.459860
H	-2.359394	-3.006888	2.203235
H	-3.494482	-2.298903	1.065492
C	-3.763172	0.016037	-1.036438
H	-3.378278	0.867553	-1.615226
H	-4.802425	-0.121779	-1.365460
H	-1.388706	-0.829775	2.727890
H	-1.668144	-3.373270	-0.175483
H	-4.432586	-0.257702	1.002129
H	-2.823438	-1.253424	-2.491043
B	-1.227920	0.091852	0.003518
C	-0.001968	1.087152	-0.373725
C	1.304223	0.633952	0.400645
C	2.435999	1.672585	0.338505
H	2.701481	1.860948	-0.707734
H	2.026748	2.614439	0.724915
C	1.076374	0.128357	1.836079
H	2.015681	-0.313844	2.185827
H	0.363307	-0.703080	1.809462
C	-0.300475	2.570951	0.033529
H	0.385085	3.206356	-0.543169
H	-0.036518	2.736040	1.080582
C	0.268949	1.074791	-1.905103
H	1.240893	1.553847	-2.089859
H	0.371936	0.040373	-2.242831
I	2.170089	-1.215286	-0.575202
C	0.629556	1.159645	2.874061
H	0.687758	0.701445	3.869157
H	1.280484	2.043639	2.877196
H	-0.402274	1.492050	2.722652
C	3.697679	1.368337	1.140564
H	3.499285	1.349089	2.219568
H	4.148290	0.409976	0.855481
H	4.439881	2.154727	0.957707
C	-1.737705	3.083199	-0.179008
H	-2.328748	2.441931	-0.838529
H	-2.281676	3.162860	0.768557
H	-1.727363	4.082633	-0.630818
C	-0.759513	1.748490	-2.808955
H	-1.750511	1.286782	-2.717953
H	-0.859380	2.822308	-2.610524
H	-0.443708	1.635612	-3.853723

54

B_Et_conf_24 Eopt -1028.040345

C	-2.323259	-1.987678	-0.755322
C	-1.948033	-0.527413	-1.134298
C	-2.067565	0.124247	1.380044
C	-2.433610	-1.347115	1.730940
H	-1.795042	-2.675358	-1.429576
H	-3.521619	-1.432340	1.872810
C	-3.220116	0.341688	-1.370304
H	-3.785368	-0.081901	-2.213752
C	-3.365584	0.935147	1.100098
H	-3.125648	2.000041	0.978245
H	-1.382056	-0.534090	-2.072579
H	-1.586168	0.585879	2.249334
C	-4.115950	0.431638	-0.133891
H	-4.970848	1.089510	-0.342624
H	-4.540070	-0.560611	0.078518
C	-1.971222	-2.366036	0.687609
H	-0.876974	-2.453564	0.759490
H	-2.378912	-3.358632	0.925584
H	-2.923720	1.357797	-1.677534
H	-4.015046	0.865908	1.985569
H	-3.397159	-2.157126	-0.928933
H	-1.983810	-1.610799	2.698154
B	-1.207628	0.224255	0.042626
C	0.122019	1.144041	-0.146386
C	1.353526	0.372423	0.444957

C	2.757846	0.977616	0.241320
H	3.472313	0.188714	0.498890
H	2.922050	1.183098	-0.818477
C	1.141020	-0.061664	1.903557
H	0.282382	-0.739097	1.959717
H	0.852594	0.840668	2.462564
C	-0.109883	2.461348	0.661865
H	0.833506	2.982588	0.839495
H	-0.503742	2.215988	1.653924
C	0.285218	1.498721	-1.648888
H	0.509783	0.583928	-2.206429
H	-0.706361	1.807888	-2.002282
I	1.541609	-1.569700	-0.720778
C	2.327462	-0.708346	2.610063
H	3.162077	-0.009955	2.743978
H	2.016600	-1.045499	3.606116
H	2.695341	-1.583321	2.059461
C	3.118529	2.214901	1.077073
H	4.208270	2.254455	1.196465
H	2.810350	3.150076	0.597821
H	2.676562	2.187811	2.081170
C	-1.066547	3.446264	-0.016479
H	-1.963574	2.953299	-0.409482
H	-1.393303	4.209678	0.700456
H	-0.588252	3.963532	-0.856256
C	1.294360	2.591694	-2.049101
H	1.567869	3.245587	-1.212734
H	2.220761	2.168210	-2.452858
H	0.870828	3.231634	-2.833318

54

B_Et_conf_25 Eopt -1028.035524

C	-1.597050	-2.351418	0.149080
C	-1.589394	-1.127794	-0.795812
C	-2.269305	0.386168	1.185504
C	-2.411362	-0.855448	2.098386
H	-0.561345	-2.539286	0.470212
H	-3.301533	-0.728765	2.734462
C	-2.965299	-0.864365	-1.464665
H	-3.295831	-1.782380	-1.975041
C	-3.594834	0.721119	0.443527
H	-3.464564	1.650846	-0.127372
H	-0.884353	-1.351019	-1.605417
H	-2.030295	1.234262	1.840352
C	-4.073118	-0.365913	-0.525738
H	-4.893252	0.041686	-1.133525
H	-4.508278	-1.201736	0.031003
C	-2.492550	-2.219433	1.389454
H	-2.215718	-3.004191	2.107413
H	-3.529559	-2.435068	1.113159
H	-2.819061	-0.113686	-2.256402
H	-4.378676	0.928056	1.188155
H	-1.900150	-3.244659	-0.418968
H	-1.555887	-0.866071	2.788523
B	-1.206044	0.169633	0.030057
C	0.043066	1.142679	-0.318543
C	1.321786	0.531033	0.391433
C	2.588701	1.421647	0.414438
H	3.434917	0.751586	0.593524
H	2.751955	1.841623	-0.585470
C	0.976536	-0.021726	1.780751
H	0.257826	-0.841547	1.660176
H	0.439215	0.773491	2.320634
C	-0.099539	2.586108	0.259473
H	0.663874	3.197576	-0.240584
H	0.167212	2.585852	1.321463
C	0.272074	1.242472	-1.848406
H	1.285759	1.629414	-2.029974
H	0.254454	0.235618	-2.275368
I	2.043892	-1.277677	-0.754751
C	2.130010	-0.519177	2.644734
H	2.847333	0.275048	2.879911
H	1.735329	-0.900893	3.594317
H	2.672102	-1.336469	2.154456
C	2.675061	2.540573	1.470016
H	3.728009	2.687638	1.739839
H	2.293132	3.497284	1.103380
H	2.133211	2.298671	2.392227
C	-1.443127	3.322582	0.151864
H	-2.107685	3.081918	0.987266
H	-1.260047	4.404383	0.192088
H	-1.987752	3.120814	-0.772532
C	-0.705748	2.101353	-2.647509
H	-0.601899	3.169829	-2.423090
H	-0.508773	1.970144	-3.718898
H	-1.751187	1.815981	-2.466347

54

B_Et_conf_26 Eopt -1028.035514

C	-2.280056	-2.207026	-0.481577
C	-2.129424	-0.769046	-1.062528
C	-2.075519	0.172056	1.354342
C	-2.265220	-1.270932	1.905808
H	-1.732160	-2.910159	-1.122749
H	-3.324982	-1.448038	2.142117
C	-3.525726	-0.109648	-1.255632
H	-4.115221	-0.728384	-1.948746
C	-3.445009	0.851137	1.076080
H	-3.281203	1.878288	0.708238
H	-1.667700	-0.838520	-2.054087
H	-1.578866	0.764886	2.123316
C	-4.282748	0.084530	0.055543
H	-5.222031	0.621030	-0.135856
H	-4.567398	-0.892941	0.470807
C	-1.760055	-2.355683	0.951620
H	-0.659987	-2.301999	0.923550
H	-2.012641	-3.351429	1.341905
H	-3.416651	0.873983	-1.735857
H	-3.993813	0.945049	2.025099
H	-3.336374	-2.515219	-0.521840
H	-1.725314	-1.365346	2.857960
B	-1.325480	0.159081	-0.052225
C	-0.054638	1.074990	-0.465515
C	1.253226	0.681074	0.318933
C	2.377039	1.717830	0.113432
H	2.174067	2.301152	-0.788855
H	2.266326	2.421601	0.952367
C	1.023886	0.383840	1.808650
H	0.360872	-0.481992	1.907476
H	0.471871	1.239793	2.212835
C	-0.443249	2.577701	-0.209234
H	-1.421143	2.717229	-0.692517
H	0.247837	3.200979	-0.795107
C	0.223357	0.984129	-1.997255
H	1.232334	1.364262	-2.209521
H	0.242797	-0.069657	-2.286355
I	1.916886	-1.335242	-0.482038
C	2.255742	0.165456	2.679457
H	2.892111	1.057585	2.723863
H	1.933378	-0.058951	3.703401
H	2.862185	-0.677815	2.327698
C	3.834656	1.269578	0.046034
H	4.042513	0.766410	-0.905420
H	4.483797	2.152618	0.109306
H	4.109407	0.585392	0.854569
C	-0.542164	3.196064	1.184843
H	-1.243996	2.673810	1.842576
H	0.426459	3.257994	1.693783
H	-0.908198	4.225162	1.073563
C	-0.749943	1.705387	-2.928316
H	-0.662327	2.796428	-2.865096
H	-0.535611	1.420787	-3.966334
H	-1.792813	1.439794	-2.725067

54

B_Et_conf_27 Eopt -1028.035630

C	-2.148804	-2.299901	0.444602
C	-1.572181	-1.279867	-0.580967
C	-2.708776	0.654688	0.723690
C	-3.322575	-0.376205	1.713677
H	-1.397150	-3.080036	0.630499
H	-4.371815	-0.565835	1.442695
C	-2.579860	-1.053172	-1.745082

H -2.760272 -2.016150 -2.245707
C -3.669596 0.846182 -0.492914
H -3.263736 1.590449 -1.194396
H -0.663967 -1.698557 -1.025717
H -2.619673 1.620956 1.233717
C -3.899095 -0.450470 -1.267707
H -4.552883 -0.254059 -2.128380
H -4.431817 -1.176769 -0.637065
C -2.566225 -1.706493 1.799233
H -1.666904 -1.545894 2.409876
H -3.171846 -2.441005 2.347979
H -2.134085 -0.386748 -2.499536
H -4.624909 1.249516 -0.125598
H -3.009286 -2.817912 -0.004926
H -3.350875 0.066445 2.718027
B -1.357448 0.158691 0.054649
C -0.027873 1.061667 -0.176153
C 1.204804 0.384317 0.552836
C 2.406322 1.292126 0.921198
H 3.259183 0.628940 1.090663
H 2.672057 1.907496 0.055247
C 0.712407 -0.408755 1.771361
H 0.095178 -1.236889 1.408765
H 0.038367 0.261189 2.332575
C -0.182504 2.484432 0.433631
H 0.779962 3.000305 0.387792
H -0.431587 2.394104 1.500060
C 0.093439 1.203858 -1.716697
H 0.153627 0.208156 -2.170948
H -0.854128 1.631954 -2.076759
I 2.173059 -1.171794 -0.765266
C 1.760510 -0.975569 2.721111
H 2.348987 -0.192472 3.211176
H 1.264727 -1.559110 3.506567
H 2.450860 -1.644664 2.192878
C 2.258784 2.178992 2.178352
H 1.415914 1.884944 2.813575
H 3.167229 2.107591 2.788023
H 2.118842 3.233915 1.923218
C -1.187842 3.422745 -0.234685
H -2.193987 3.004366 -0.310310
H -1.261997 4.346944 0.353043
H -0.867001 3.706201 -1.244722
C 1.240908 2.061882 -2.246571
H 2.214280 1.595679 -2.053547
H 1.144441 2.179979 -3.333459
H 1.248300 3.065825 -1.802924
54
B_Et_conf_28 Eopt -1028.036697
C -1.443451 -2.307356 0.615040
C -1.515071 -1.313980 -0.577021
C -2.328221 0.507751 1.086386
C -2.290784 -0.535167 2.234361
H -0.421914 -2.325001 1.022295
H -3.106780 -0.307573 2.936992
C -2.940065 -1.346809 -1.215602
H -3.510383 -2.189380 -0.797328
C -3.739105 0.472043 0.410636
H -4.167597 1.483019 0.415151
H -0.784560 -1.641886 -1.323551
H -2.189150 1.497007 1.537712
C -3.734874 -0.050527 -1.028936
H -3.292137 0.720436 -1.676634
H -4.767603 -0.189106 -1.377822
C -2.432471 -1.972316 1.732015
H -2.296858 -2.672043 2.568070
H -3.458513 -2.129641 1.368975
H -2.849901 -1.549856 -2.290747
H -4.416381 -0.144763 1.019445
H -1.641033 -3.322454 0.238205
H -1.360884 -0.436065 2.810794
B -1.217179 0.138002 -0.002433
C 0.012059 1.117827 -0.430085
C 1.320996 0.681628 0.344920
C 2.489314 1.654740 0.135310
H 2.694941 1.740133 -0.938882
H 2.141339 2.642719 0.460961
C 1.141407 0.308318 1.828134
H 2.084079 -0.134412 2.166216
H 0.404842 -0.498800 1.907792
C -0.339390 2.580197 -0.004008
H 0.499506 3.242720 -0.243464
H -0.483400 2.640149 1.075568
C 0.295752 1.173059 -1.967412
H 0.706828 2.168443 -2.192384
H 1.091141 0.467225 -2.223787
I 2.077932 -1.291275 -0.493158
C 0.776834 1.432387 2.802769
H 1.019583 1.105535 3.821164
H 1.341872 2.352242 2.605174
H -0.291506 1.673060 2.782998
C 3.779780 1.367199 0.895581
H 4.179043 0.370150 0.674626
H 4.539911 2.104101 0.609449
H 3.636275 1.449291 1.980579
C -1.571052 3.172181 -0.689534
H -2.440916 2.505869 -0.647617
H -1.851103 4.110892 -0.194899
H -1.373877 3.405783 -1.742223
C -0.856890 0.907894 -2.936556
H -1.211074 -0.126930 -2.877244
H -1.712469 1.571536 -2.778212
H -0.504412 1.070790 -3.963830
54
B_Et_conf_29 Eopt -1028.037779
C -2.129046 -2.176386 -0.326562
C -1.989212 -0.753482 -0.925504
C -1.990633 0.186209 1.500283
C -2.182399 -1.256438 2.031743
H -1.131743 -2.623415 -0.200841
H -2.777667 -1.217925 2.956705
C -3.409042 -0.131952 -1.145543
H -4.175876 -0.899525 -0.969021
C -3.383717 0.846983 1.232081
H -3.430829 1.811941 1.753702
H -1.478001 -0.843480 -1.892608
H -1.475660 0.764602 2.277314
C -3.692347 1.082149 -0.252556
H -3.074279 1.922861 -0.605659
H -4.737596 1.399692 -0.369700
C -2.851784 -2.183908 1.019852
H -2.877791 -3.206881 1.419842
H -3.901118 -1.885006 0.882427
H -3.523114 0.166362 -2.195484
H -4.173260 0.221090 1.674287
H -2.669999 -2.812615 -1.043624
H -1.212573 -1.694496 2.311698
B -1.208705 0.164905 0.110854
C 0.088142 1.090254 -0.213589
C 1.395534 0.406103 0.349484
C 2.709350 1.198644 0.159753
H 3.542138 0.492589 0.078447
H 2.681039 1.727232 -0.798173
C 1.262034 -0.064891 1.802543
H 0.340769 -0.636259 1.921525
H 1.134143 0.842906 2.408306
C -0.168038 2.454682 0.519794
H 0.055605 2.342751 1.586276
H -1.249735 2.654522 0.487502
C 0.207255 1.379484 -1.731227
H 1.078953 2.020831 -1.911637
H 0.406176 0.446674 -2.268191
I 1.693651 -1.494236 -0.830116
C 2.413183 -0.884739 2.385217
H 3.398707 -0.499614 2.099647
H 2.353862 -0.873334 3.480635
H 2.359144 -1.928986 2.058755
C 3.057583 2.168672 1.300251
H 2.211871 2.774188 1.638112
H 3.444061 1.629670 2.172710

H 3.844409 2.853519 0.959965
C 0.529858 3.707473 -0.025049
H 0.062058 4.062243 -0.949518
H 0.450285 4.517656 0.711515
H 1.591646 3.558528 -0.239339
C -1.006107 2.039401 -2.390204
H -0.733336 2.384020 -3.395955
H -1.838173 1.338928 -2.505956
H -1.377798 2.909182 -1.836407
54
B_Et_conf_3 Eopt -1028.041167
C -1.929933 -2.214315 -0.345060
C -1.649816 -0.851091 -1.017916
C -2.586798 0.379630 1.055555
C -2.762197 -0.975920 1.785348
H -0.959658 -2.624331 -0.025001
H -3.658039 -0.920049 2.422766
C -2.845204 -0.303349 -1.841292
H -3.061400 -0.998229 -2.667007
C -3.860434 0.759203 0.249477
H -3.789235 1.815218 -0.044900
H -0.826904 -1.003830 -1.723987
H -2.453814 1.150380 1.828330
C -4.115474 -0.060013 -1.024043
H -4.844206 0.477814 -1.646762
H -4.586046 -1.018494 -0.782060
C -2.887367 -2.199844 0.862486
H -2.713132 -3.107123 1.457697
H -3.922236 -2.279623 0.514065
H -2.539112 0.645143 -2.313771
H -4.734656 0.692079 0.915683
H -2.315287 -2.919994 -1.097812
H -1.917175 -1.121162 2.472225
B -1.350806 0.267050 0.062388
C -0.021541 1.202021 0.053912
C 1.191964 0.373339 0.646083
C 2.369127 1.253785 1.120557
H 2.415425 2.156036 0.507409
H 2.077567 1.591048 2.127549
C 0.655498 -0.515224 1.780473
H -0.041722 -1.245018 1.348583
H 0.055498 0.147231 2.423764
C -0.227697 2.441216 0.975604
H 0.702054 3.008631 1.078574
H -0.485327 2.100305 1.986703
C 0.152763 1.683661 -1.408750
H 0.210101 0.812580 -2.070547
H -0.775406 2.201307 -1.690388
I 1.979936 -1.062850 -0.878115
C 1.643221 -1.261998 2.665961
H 1.087147 -1.866947 3.392543
H 2.279959 -1.939056 2.083139
H 2.284399 -0.575041 3.230906
C 3.783487 0.680144 1.190153
H 3.830716 -0.312262 1.647048
H 4.225131 0.607526 0.189593
H 4.409954 1.359327 1.783139
C -1.293151 3.425980 0.499871
H -0.950591 3.998705 -0.371189
H -2.229654 2.931496 0.223246
H -1.520722 4.145391 1.296610
C 1.328789 2.607478 -1.719427
H 2.288251 2.080604 -1.635336
H 1.247007 2.975134 -2.750195
H 1.357133 3.482783 -1.056395
54
B_Et_conf_30 Eopt -1028.036809
C 3.449481 0.910066 1.116003
C 2.677421 1.057477 -0.225009
C 1.676186 -1.302093 0.102110
C 2.503385 -1.472485 1.412140
H 2.880227 1.430788 1.901915
H 2.848181 -2.516374 1.468447
C 3.479104 0.547765 -1.441358
H 4.456164 1.055210 -1.468016
C 2.473846 -1.809325 -1.121498
H 1.800505 -1.866459 -1.985576
H 2.534223 2.133340 -0.372377
H 0.793206 -1.943762 0.207027
C 3.701345 -0.970388 -1.508046
H 4.003752 -1.237937 -2.530332
H 4.551061 -1.244959 -0.874616
C 3.712384 -0.534458 1.564121
H 4.019854 -0.525731 2.619296
H 4.569781 -0.936477 1.015075
H 2.952996 0.870596 -2.353076
H 2.797636 -2.843435 -0.926654
H 4.407176 1.446722 1.034239
H 1.839267 -1.329416 2.273510
B 1.321303 0.249088 -0.006173
C -0.069501 0.963209 0.472585
C -1.379294 0.659273 -0.348728
C -2.568761 1.561675 0.038679
H -2.807187 1.433978 1.095431
H -2.219570 2.596104 -0.076003
C -1.223647 0.698763 -1.875755
H -1.175827 1.757409 -2.163562
H -2.146264 0.313614 -2.321529
C 0.163791 2.504060 0.534362
H 1.083696 2.642723 1.116679
H -0.622563 2.980445 1.131992
C -0.113562 0.476378 1.959860
H -0.121265 -0.618316 1.975909
H 0.846920 0.774028 2.406729
I -2.087128 -1.436605 0.083467
C -0.054064 -0.048455 -2.479544
H -0.085175 -1.108626 -2.204725
H -0.084244 0.019348 -3.574070
H 0.914589 0.371996 -2.175874
C -3.849062 1.426631 -0.783059
H -3.716886 1.775661 -1.813779
H -4.214490 0.392630 -0.811769
H -4.632535 2.044500 -0.327815
C 0.302691 3.268477 -0.784982
H 0.862180 4.198129 -0.620985
H 0.837105 2.688049 -1.548386
H -0.670801 3.551262 -1.202842
C -1.217036 0.959786 2.903707
H -0.932498 0.710311 3.933863
H -1.376078 2.044112 2.860697
H -2.170099 0.457019 2.704783
54
B_Et_conf_31 Eopt -1028.040916
C -3.266239 0.489287 1.531864
C -1.834235 -0.137041 1.488152
C -2.081507 -0.456273 -1.078191
C -3.515958 0.170672 -1.001044
H -3.286462 1.278209 2.295157
H -4.259193 -0.638988 -0.967451
C -1.886581 -1.676288 1.643854
H -2.322339 -1.924391 2.623859
C -2.201725 -1.986425 -0.862148
H -1.232512 -2.465451 -1.043105
H -1.284938 0.258397 2.347338
H -1.675869 -0.293100 -2.085063
C -2.692753 -2.356682 0.537558
H -2.651590 -3.447114 0.666217
H -3.752416 -2.079759 0.641799
C -3.731423 1.093574 0.201598
H -3.175252 2.028499 0.029668
H -4.790475 1.378091 0.270500
H -0.859637 -2.075892 1.645175
H -2.896858 -2.391040 -1.613731
H -3.989043 -0.272381 1.861064
H -3.724631 0.730490 -1.922073
B -1.189115 0.203617 0.071737
C 0.058946 1.233582 -0.144757
C 1.429242 0.568258 0.261783
C 2.650345 1.269079 -0.358472
H 2.619942 1.152226 -1.445608

H 2.518001 2.341123 -0.163533
C 1.563589 0.394679 1.783781
H 0.567456 0.302552 2.216204
H 1.957773 1.361024 2.139071
C -0.270400 2.446557 0.784669
H -0.347216 2.109468 1.823700
H -1.285122 2.771220 0.517070
C 0.112425 1.719479 -1.612766
H 0.991300 2.352758 -1.772084
H 0.246825 0.844653 -2.264543
I 1.459609 -1.479987 -0.681706
C 2.404278 -0.738247 2.372088
H 1.871197 -1.692759 2.294913
H 3.377791 -0.861873 1.891720
H 2.572947 -0.535758 3.437700
C 4.032984 0.862955 0.134446
H 4.787271 1.435046 -0.419325
H 4.172513 1.077926 1.201031
H 4.230427 -0.202807 -0.037634
C 0.632903 3.679967 0.740229
H 0.849156 4.004757 -0.286039
H 0.135265 4.513543 1.251932
H 1.587808 3.512040 1.253361
C -1.095964 2.526208 -2.083266
H -2.046955 2.019604 -1.892366
H -1.137818 3.508833 -1.595167
H -1.028618 2.702040 -3.164301
54
B_Et_conf_32 Eopt -1028.036015
C -1.884478 -2.067782 -1.054373
C -1.855298 -0.521715 -1.252565
C -2.189720 -0.170072 1.296414
C -2.195199 -1.721738 1.501162
H -1.158802 -2.527271 -1.738114
H -3.232374 -2.056931 1.643651
C -3.304151 0.005736 -1.459389
H -3.712866 -0.446460 -2.375376
C -3.647138 0.292861 1.019360
H -3.699686 1.386921 0.959932
H -1.296270 -0.299131 -2.168295
H -1.862562 0.309600 2.227647
C -4.214738 -0.290976 -0.271296
H -5.215072 0.123502 -0.456916
H -4.348318 -1.377416 -0.166252
C -1.548694 -2.534202 0.368262
H -0.455570 -2.485045 0.477889
H -1.812866 -3.594401 0.483693
H -3.295670 1.093461 -1.628864
H -4.272478 -0.003308 1.875085
H -2.869785 -2.454906 -1.353335
H -1.674848 -1.964350 2.437768
B -1.274698 0.196962 0.042293
C 0.029455 1.180960 0.039996
C 1.285138 0.452783 0.663236
C 2.517057 1.322483 0.984370
H 2.235441 1.924761 1.863179
H 3.287571 0.631714 1.342018
C 0.915545 -0.328275 1.939914
H 0.206105 -1.114788 1.683348
H 0.368882 0.370653 2.588741
C -0.203046 2.444503 0.931320
H 0.500918 3.215979 0.589880
H 0.074737 2.234492 1.969620
C 0.330992 1.651831 -1.408754
H 1.320829 2.108514 -1.445585
H 0.388584 0.776266 -2.060637
I 2.011221 -1.114872 -0.770159
C 2.045850 -0.949548 2.755325
H 1.614253 -1.591799 3.532678
H 2.701308 -1.572360 2.133050
H 2.661052 -0.195210 3.258671
C 3.178446 2.233116 -0.044614
H 2.581942 3.121500 -0.279644
H 4.133107 2.581788 0.369024
H 3.397304 1.699071 -0.978741
C -1.624794 3.023390 0.948864
H -2.188967 2.797128 0.036900
H -2.197382 2.631245 1.795959
H -1.595815 4.115304 1.051595
C -0.652803 2.639214 -2.030348
H -0.668539 3.599908 -1.499778
H -0.353608 2.841824 -3.066528
H -1.676345 2.247763 -2.056631
54
B_Et_conf_33 Eopt -1028.036604
C -2.580343 -1.165812 1.716211
C -1.592041 -1.212463 0.509700
C -2.765348 0.889302 -0.473308
C -3.738596 0.897170 0.744494
H -2.062319 -1.520679 2.618120
H -4.679413 0.391726 0.478261
C -2.161905 -2.031968 -0.674947
H -2.325120 -3.070179 -0.348003
C -3.308035 0.015930 -1.629808
H -2.624382 0.089144 -2.490984
H -0.675395 -1.711165 0.832097
H -2.671510 1.914739 -0.852157
C -3.458637 -1.453136 -1.237874
H -3.779126 -2.039893 -2.109808
H -4.260346 -1.553888 -0.491234
C -3.141765 0.234411 1.990806
H -2.321611 0.874080 2.363198
H -3.888459 0.192749 2.795645
H -1.407399 -2.072872 -1.477299
H -4.277165 0.416515 -1.964599
H -3.413019 -1.865008 1.546037
H -4.006961 1.933430 0.989399
B -1.374121 0.272854 -0.002249
C -0.023611 1.137284 -0.118991
C 1.226313 0.466581 0.545064
C 2.490149 1.343129 0.529055
H 2.765484 1.592653 -0.497073
H 2.198316 2.284163 1.016326
C 0.910058 0.058188 2.010036
H -0.174650 0.053134 2.158464
H 1.282276 0.887413 2.628733
C -0.308177 2.492703 0.627738
H 0.062795 2.417669 1.657486
H -1.394660 2.613776 0.731467
C 0.013861 1.338885 -1.669558
H -0.237472 0.380607 -2.152169
H -0.822479 2.007887 -1.907737
I 1.766380 -1.337366 -0.677672
C 1.471134 -1.238460 2.586427
H 1.289721 -1.255834 3.669061
H 0.985039 -2.117933 2.148476
H 2.548991 -1.337239 2.419716
C 3.718428 0.812148 1.262000
H 4.051716 -0.151507 0.856855
H 4.542838 1.526188 1.146155
H 3.537346 0.692810 2.337062
C 0.213447 3.793765 0.014181
H -0.096060 4.636486 0.645896
H 1.305725 3.825447 -0.066143
H -0.204323 3.964026 -0.986081
C 1.254573 1.872516 -2.391889
H 2.074243 1.146194 -2.388126
H 0.989948 2.056288 -3.441574
H 1.622903 2.814155 -1.973616
54
B_Et_conf_34 Eopt -1028.036675
C -2.272680 -2.251926 -0.248350
C -1.618944 -1.007799 -0.921272
C -2.757812 0.528292 0.833922
C -3.450659 -0.711544 1.469442
H -1.549960 -3.080062 -0.254599
H -4.493529 -0.763525 1.123228
C -2.567689 -0.432191 -2.011219
H -2.758221 -1.215532 -2.759993
C -3.651375 1.091984 -0.315959

H	-3.187892	1.982931	-0.765839
H	-0.700652	-1.321928	-1.429363
H	-2.666326	1.307814	1.599001
C	-3.886410	0.071688	-1.429096
H	-4.495348	0.526034	-2.222487
H	-4.469166	-0.777214	-1.043229
C	-2.752301	-2.049518	1.198200
H	-1.889150	-2.124723	1.873560
H	-3.420234	-2.875892	1.477839
H	-2.071421	0.393972	-2.541997
H	-4.609974	1.419727	0.112931
H	-3.117127	-2.592001	-0.866157
H	-3.506074	-0.570238	2.556812
B	-1.389601	0.181607	0.108395
C	-0.018468	1.055074	0.191733
C	1.144797	0.136129	0.765610
C	2.284058	0.791248	1.567660
H	1.817479	1.202819	2.476601
H	2.923127	-0.027051	1.915152
C	0.489157	-0.939838	1.655690
H	-0.097161	-1.597211	1.007461
H	-0.226028	-0.403214	2.304032
C	-0.165938	2.251131	1.177765
H	0.809625	2.715077	1.349079
H	-0.490191	1.863768	2.155001
C	0.132919	1.583733	-1.259044
H	0.198422	0.729418	-1.942986
H	-0.815344	2.083111	-1.511178
I	2.190239	-1.014630	-0.846341
C	1.372742	-1.814399	2.536935
H	0.763057	-2.609812	2.982602
H	2.171295	-2.291270	1.953725
H	1.830480	-1.250861	3.357350
C	3.204883	1.833403	0.947938
H	2.693217	2.755759	0.659642
H	3.975868	2.096417	1.683202
H	3.714762	1.432230	0.063065
C	-1.106897	3.380361	0.757748
H	-1.174886	4.114564	1.570994
H	-0.735049	3.909593	-0.128128
H	-2.122364	3.041147	0.540453
C	1.269013	2.550049	-1.577438
H	2.243947	2.055018	-1.519922
H	1.148508	2.928487	-2.600874
H	1.281247	3.414732	-0.902482

54

B_Et_conf_35 Eopt -1028.037701

C	-3.422667	0.000453	1.418935
C	-1.882416	-0.175342	1.414391
C	-1.933484	-0.453700	-1.147748
C	-3.396852	0.065900	-1.132748
H	-3.634998	1.053436	1.647678
H	-3.916562	-0.304123	-2.029165
C	-1.462136	-1.664648	1.543032
H	-1.778287	-2.045817	2.526064
C	-1.893530	-1.998629	-0.989238
H	-0.935236	-2.344470	-1.392037
H	-1.511192	0.360174	2.297798
H	-1.501183	-0.218062	-2.129755
C	-2.044792	-2.557977	0.441788
H	-1.552328	-3.539305	0.487326
H	-3.099133	-2.749533	0.669547
C	-4.165021	-0.352646	0.119949
H	-5.143176	0.148021	0.126431
H	-4.382955	-1.424733	0.068710
H	-0.364799	-1.739310	1.523760
H	-2.671803	-2.440281	-1.631869
H	-3.844184	-0.580681	2.254545
H	-3.415116	1.163579	-1.201824
B	-1.198218	0.277399	0.054542
C	0.040241	1.329329	-0.094705
C	1.409203	0.696655	0.389816
C	2.617461	1.585080	0.041546
H	2.730698	1.649185	-1.043577
H	2.355036	2.596312	0.392172
C	1.409674	0.350715	1.896115
H	0.380837	0.229203	2.235756
H	1.779923	1.257388	2.396928
C	-0.232207	2.593946	0.787704
H	0.315390	3.434853	0.340504
H	0.188783	2.467210	1.789806
C	0.181398	1.810922	-1.562774
H	1.066294	2.452059	-1.642071
H	0.366011	0.945337	-2.211070
I	1.737160	-1.184394	-0.789406
C	2.217187	-0.836058	2.418700
H	3.245564	-0.853135	2.046978
H	2.251605	-0.781475	3.514803
H	1.750075	-1.789168	2.146527
C	3.967639	1.205444	0.638068
H	3.968255	1.247729	1.733863
H	4.277475	0.199472	0.326968
H	4.725463	1.912688	0.279801
C	-1.709056	2.988022	0.952403
H	-1.816358	4.078338	1.009685
H	-2.337138	2.645494	0.120148
H	-2.130478	2.569466	1.872471
C	-0.975487	2.622979	-2.141547
H	-1.930033	2.088573	-2.114911
H	-1.104538	3.577923	-1.616089
H	-0.761244	2.856855	-3.192205

54

B_Et_conf_36 Eopt -1028.034935

C	-2.283222	-0.462601	2.044318
C	-1.571655	-1.059918	0.782838
C	-2.814923	0.665347	-0.704983
C	-3.523065	1.237062	0.568410
H	-1.601000	-0.506251	2.903852
H	-4.533268	0.809093	0.643684
C	-2.463238	-2.180827	0.185654
H	-2.636168	-2.934948	0.968408
C	-3.663865	-0.489770	-1.292971
H	-3.226088	-0.834221	-2.240760
H	-0.633457	-1.527798	1.098945
H	-2.779982	1.457492	-1.460555
C	-3.807633	-1.669111	-0.331167
H	-4.350934	-2.486898	-0.824407
H	-4.431536	-1.362987	0.521536
C	-2.765837	0.982669	1.876068
H	-1.889909	1.647357	1.905531
H	-3.393663	1.267585	2.731548
H	-1.932728	-2.697110	-0.623363
H	-4.661320	-0.098191	-1.544422
H	-3.139217	-1.100380	2.308824
H	-3.664081	2.319394	0.450102
B	-1.375062	0.115780	-0.279694
C	0.012942	0.711019	-0.890152
C	1.236583	0.754801	0.101082
C	2.402237	1.667513	-0.317837
H	2.023516	2.698570	-0.235278
H	3.173228	1.575954	0.454530
C	0.787529	1.115625	1.526353
H	0.159674	0.307621	1.908426
H	0.134581	1.990635	1.428300
C	-0.242609	2.161893	-1.425695
H	-1.018086	2.056493	-2.191648
H	0.644585	2.508986	-1.968299
C	0.335975	-0.126256	-2.184409
H	-0.013014	0.445974	-3.053672
H	1.420545	-0.198814	-2.290857
I	2.200772	-1.275565	0.327449
C	1.861567	1.438699	2.559638
H	2.614231	0.642625	2.625840
H	2.375154	2.382281	2.341467
H	1.394435	1.540307	3.546610
C	3.093000	1.493003	-1.666999
H	3.497085	0.478354	-1.778053
H	2.441479	1.697866	-2.523125
H	3.936710	2.192287	-1.719503
C	-0.693886	3.259153	-0.464119

H -1.510751 2.925315 0.187514
H 0.116629 3.629856 0.175315
H -1.063769 4.116330 -1.041336
C -0.242640 -1.534357 -2.301554
H 0.155305 -2.018288 -3.202889
H 0.016571 -2.163025 -1.443619
H -1.333916 -1.519097 -2.401811
54
B_Et_conf_4 Eopt -1028.042907
C -1.952514 -2.151113 -0.278206
C -1.715495 -0.797573 -0.987784
C -2.382570 0.432786 1.190434
C -2.810029 -0.914149 1.817623
H -0.994346 -2.516840 0.121301
H -3.739649 -0.770252 2.390646
C -3.003390 -0.274803 -1.684534
H -3.446999 -1.098764 -2.265131
C -3.526084 1.142354 0.414535
H -3.156676 2.113319 0.046531
H -0.973593 -0.965323 -1.777519
H -2.094247 1.097807 2.015006
C -4.080199 0.332937 -0.764998
H -4.730281 0.983465 -1.366789
H -4.734614 -0.455155 -0.377279
C -2.992612 -2.094271 0.846493
H -2.949964 -3.030988 1.419811
H -3.994830 -2.068408 0.404929
H -2.703391 0.480663 -2.423052
H -4.344001 1.377304 1.112257
H -2.269090 -2.896112 -1.024189
H -2.043300 -1.189481 2.557278
B -1.251451 0.261911 0.097768
C 0.114118 1.145485 0.038130
C 1.263744 0.259964 0.644898
C 2.573598 0.952951 1.038123
H 2.321856 1.610492 1.886013
H 3.227954 0.178864 1.454241
C 0.709329 -0.526686 1.850747
H -0.063254 -1.217116 1.489540
H 0.189903 0.200999 2.491734
C -0.103272 2.411424 0.932135
H -0.127211 2.116343 1.988381
H -1.122062 2.760596 0.717944
C 0.335791 1.586318 -1.427451
H 1.323348 2.042398 -1.559693
H 0.329837 0.697005 -2.066451
I 1.888843 -1.335367 -0.811883
C 1.689391 -1.311774 2.714691
H 1.129209 -1.923125 3.432448
H 2.311401 -1.985912 2.112286
H 2.348742 -0.652270 3.290506
C 3.383931 1.717684 -0.001864
H 4.329676 2.038940 0.452962
H 3.628458 1.077110 -0.858482
H 2.879332 2.611592 -0.375142
C 0.819182 3.628366 0.795006
H 1.773833 3.505426 1.315024
H 1.037308 3.882866 -0.248639
H 0.321287 4.497741 1.244034
C -0.734046 2.552574 -1.940340
H -0.651055 3.549211 -1.491276
H -0.651639 2.670955 -3.027740
H -1.746630 2.186702 -1.726365
54
B_Et_conf_5 Eopt -1028.037837
C -1.691494 -1.991085 1.019627
C -1.548017 -1.278805 -0.346616
C -2.623643 0.849715 0.653194
C -2.810166 0.120428 2.007859
H -0.716066 -1.936293 1.530322
H -3.760263 0.442094 2.462083
C -2.772615 -1.440122 -1.281526
H -2.969193 -2.512839 -1.434165
C -3.832180 0.679593 -0.300458
H -3.686677 1.351575 -1.160351
H -0.704141 -1.743642 -0.864285
H -2.556622 1.921121 0.873820
C -4.059675 -0.744428 -0.821243
H -4.763304 -0.705762 -1.665004
H -4.557165 -1.345773 -0.052875
C -2.773052 -1.415998 1.947329
H -2.610547 -1.803945 2.962742
H -3.753985 -1.796818 1.644801
H -2.491506 -1.041095 -2.269263
H -4.741538 1.035614 0.207876
H -1.886218 -3.062204 0.855083
H -2.020740 0.466994 2.692938
B -1.330640 0.261453 -0.057457
C -0.031809 1.110025 -0.517290
C 1.283852 0.677026 0.239725
C 2.449134 1.641576 -0.041982
H 2.676741 1.629478 -1.114396
H 2.081530 2.650121 0.182688
C 1.023913 0.504790 1.755025
H -0.043375 0.304599 1.912695
H 1.202028 1.493301 2.196931
C -0.306479 2.632756 -0.306884
H -1.298908 2.813447 -0.737422
H 0.387877 3.215783 -0.930864
C 0.088112 0.895992 -2.054139
H 1.052432 1.290618 -2.405795
H 0.104739 -0.179604 -2.260110
I 2.054939 -1.290363 -0.559273
C 1.802007 -0.517854 2.579259
H 1.486349 -1.540953 2.347513
H 2.882895 -0.456382 2.419736
H 1.603666 -0.335457 3.643588
C 3.734441 1.446696 0.754899
H 3.570074 1.601426 1.828752
H 4.166809 0.449523 0.608262
H 4.475948 2.185012 0.426518
C -0.314682 3.225272 1.104465
H -0.828383 4.194812 1.085309
H -0.836524 2.586814 1.826709
H 0.693940 3.406462 1.493773
C -1.028331 1.518813 -2.893428
H -2.024319 1.247277 -2.516732
H -0.969501 2.613102 -2.922210
H -0.960502 1.157238 -3.926819
54
B_Et_conf_6 Eopt -1028.039463
C 2.502354 -0.656279 -1.969960
C 1.510339 -0.987786 -0.816077
C 2.839346 0.604819 0.748535
C 3.810071 0.924298 -0.427541
H 1.955539 -0.658841 -2.923233
H 4.722735 0.316464 -0.331854
C 2.011800 -2.185796 0.033364
H 2.086332 -3.074410 -0.611498
C 3.328719 -0.636447 1.545323
H 2.672809 -0.800425 2.413958
H 0.556152 -1.286099 -1.255371
H 2.836072 1.451908 1.445113
C 3.357348 -1.910904 0.703062
H 3.640922 -2.764712 1.333692
H 4.139132 -1.826719 -0.066256
C 3.197642 0.700062 -1.814459
H 2.453294 1.491734 -1.999192
H 3.968002 0.820309 -2.588749
H 1.262389 -2.423360 0.805866
H 4.332293 -0.428355 1.946523
H 3.259893 -1.450098 -2.052858
H 4.135012 1.970777 -0.354507
B 1.397413 0.236962 0.188029
C 0.054923 0.919000 0.751434
C -1.193781 0.739887 -0.194722
C -2.387868 1.636143 0.178178
H -2.679666 1.465595 1.218171
H -2.009436 2.666839 0.123796
C -0.816982 0.993887 -1.681826

H 0.273280 1.007875 -1.781650
H -1.139466 2.022232 -1.891622
C 0.307878 2.429592 1.038394
H 1.102160 2.467710 1.792533
H -0.568606 2.873296 1.525142
C -0.027714 0.238957 2.159018
H 0.016225 -0.853402 2.054919
H 0.893590 0.527641 2.683169
I -1.977194 -1.358413 0.052928
C -1.378645 0.109454 -2.791213
H -1.117136 0.550269 -3.762193
H -0.961616 -0.903394 -2.755848
H -2.469734 0.023703 -2.739127
C -3.620632 1.565872 -0.717747
H -4.066922 0.564308 -0.727593
H -4.377551 2.265889 -0.343349
H -3.391976 1.853299 -1.751422
C 0.736844 3.311233 -0.130350
H -0.068729 3.479798 -0.854277
H 1.052755 4.294215 0.241236
H 1.588730 2.874111 -0.669345
C -1.200148 0.586607 3.077380
H -1.006794 0.174944 4.076137
H -1.333362 1.670128 3.187129
H -2.141084 0.152263 2.722476
54
B_Et_conf_7 Eopt -1028.039170
C -2.267460 -0.948986 2.064069
C -1.345092 -1.104702 0.819380
C -2.835403 0.592439 -0.462191
C -3.737505 0.723952 0.801052
H -1.658141 -1.037855 2.974223
H -4.623222 0.078511 0.697177
C -1.857485 -2.224600 -0.125040
H -1.860532 -3.179671 0.421759
C -3.314977 -0.573061 -1.372697
H -2.693766 -0.612654 -2.281373
H -0.354356 -1.407184 1.164002
H -2.921476 1.515995 -1.044813
C -3.250511 -1.933667 -0.680636
H -3.541146 -2.721900 -1.388535
H -3.986971 -1.968918 0.135453
C -3.016936 0.385104 2.109443
H -2.287429 1.183234 2.323468
H -3.730163 0.390730 2.945365
H -1.150988 -2.346273 -0.961930
H -4.343182 -0.366306 -1.706307
H -2.990884 -1.777516 2.101219
H -4.117826 1.751953 0.866699
B -1.343189 0.226108 -0.050491
C -0.063700 1.018823 -0.608765
C 1.262152 0.737303 0.198205
C 2.413172 1.664760 -0.219843
H 2.583450 1.581501 -1.300709
H 2.051562 2.686411 -0.037130
C 1.035209 0.820487 1.730151
H -0.040526 0.792078 1.936358
H 1.362446 1.827833 2.018432
C -0.358483 2.547805 -0.643171
H -1.266292 2.669562 -1.239346
H 0.427709 3.066149 -1.209160
C 0.056992 0.541196 -2.103466
H 1.109043 0.618750 -2.405853
H -0.175860 -0.531540 -2.157547
I 2.022436 -1.324741 -0.349744
C 1.721469 -0.162800 2.674010
H 1.312784 -1.174642 2.573269
H 2.801767 -0.218300 2.499778
H 1.559024 0.167286 3.708451
C 3.737309 1.521759 0.523301
H 4.452028 2.256562 0.132984
H 3.621407 1.715659 1.597165
H 4.177705 0.525732 0.397143
C -0.601882 3.271326 0.678103
H -1.012499 4.269132 0.478890
H -1.331130 2.734608 1.299917
H 0.309270 3.407791 1.270746
C -0.777433 1.265190 -3.160467
H -0.432640 2.293876 -3.317684
H -0.673805 0.735812 -4.116129
H -1.845123 1.300371 -2.915729
54
B_Et_conf_8 Eopt -1028.040031
C -1.945221 -2.243079 0.103057
C -1.621092 -1.056324 -0.832703
C -2.537327 0.604881 0.928910
C -2.757186 -0.562700 1.922804
H -0.989086 -2.612104 0.505593
H -3.659450 -0.354097 2.517965
C -2.795490 -0.659764 -1.766171
H -3.025919 -1.504623 -2.432849
C -3.792565 0.843016 0.044200
H -3.684774 1.810580 -0.465000
H -0.796856 -1.372488 -1.481314
H -2.385925 1.516392 1.524879
C -4.063295 -0.219067 -1.031485
H -4.774093 0.194067 -1.760972
H -4.560855 -1.093182 -0.598421
C -2.904150 -1.950602 1.274837
H -2.755358 -2.716609 2.048651
H -3.938907 -2.077166 0.940671
H -2.460531 0.162400 -2.420417
H -4.673270 0.943917 0.697366
H -2.354384 -3.074666 -0.491935
H -1.925701 -0.577650 2.640735
B -1.300819 0.255828 -0.006663
C 0.048573 1.135342 -0.200166
C 1.235665 0.397250 0.540784
C 2.484442 1.258918 0.869164
H 3.359889 0.602563 0.900270
H 2.661910 1.958312 0.046449
C 0.710062 -0.308414 1.795744
H -0.143429 -0.940502 1.523905
H 0.307337 0.490509 2.438523
C -0.109367 2.536632 0.450729
H 0.845660 3.071604 0.425336
H -0.366858 2.412554 1.510310
C 0.218021 1.324287 -1.730368
H 0.250903 0.344208 -2.217940
H -0.703704 1.798680 -2.096875
I 2.049204 -1.249427 -0.755886
C 1.682623 -1.172187 2.596325
H 1.802148 -2.156967 2.131195
H 2.678968 -0.723475 2.678722
H 1.295609 -1.324738 3.611358
C 2.470193 2.025301 2.200221
H 3.398558 2.606546 2.269548
H 1.635592 2.723200 2.303626
H 2.446618 1.347221 3.060647
C -1.145153 3.445801 -0.207098
H -1.342896 4.312995 0.435519
H -0.790629 3.827452 -1.172756
H -2.099611 2.939369 -0.382684
C 1.406407 2.152779 -2.214456
H 1.317494 2.331436 -3.293624
H 1.460464 3.131918 -1.719974
H 2.356520 1.630411 -2.047193
54
B_Et_conf_9 Eopt -1028.038368
C -3.898857 0.552424 -0.451354
C -2.678792 0.955310 0.413120
C -1.578832 -1.330476 -0.069492
C -2.681121 -1.601476 -1.125557
H -3.837620 1.125229 -1.388881
H -2.824092 -2.688792 -1.219151
C -2.809551 0.543097 1.902577
H -3.662931 1.073320 2.352171
C -1.963347 -1.829354 1.345215
H -1.038053 -1.894854 1.936982
H -2.629103 2.049350 0.382120

H	-0.698162	-1.897082	-0.384578
C	-2.976441	-0.965303	2.119673
H	-2.880480	-1.183373	3.192465
H	-4.000652	-1.249225	1.854131
C	-4.023061	-0.936887	-0.810882
H	-4.687006	-1.035960	-1.681300
H	-4.515282	-1.488337	-0.002071
H	-1.915774	0.890160	2.446866
H	-2.343776	-2.860614	1.274491
H	-4.822057	0.890956	0.045232
H	-2.323898	-1.256714	-2.109101
B	-1.353072	0.238470	-0.075447
C	-0.030518	0.992217	-0.621628
C	1.284237	0.661931	0.183476
C	2.420584	1.640558	-0.171518
H	2.319392	1.920666	-1.226026
H	2.205249	2.550278	0.405543
C	0.958182	0.591840	1.682789
H	0.283010	-0.258424	1.842969
H	0.369904	1.487993	1.910692
C	-0.263896	2.538234	-0.653673
H	-1.220089	2.677359	-1.171415
H	0.486720	3.005046	-1.307765
C	0.083593	0.537387	-2.108224
H	1.069772	0.828435	-2.499991
H	0.057834	-0.557911	-2.141889
I	2.065304	-1.398826	-0.303641
C	2.104523	0.519719	2.682195
H	1.693700	0.427065	3.694854
H	2.752430	-0.346877	2.502090
H	2.719009	1.427965	2.658104
C	3.885418	1.268265	0.059167
H	4.243291	0.577921	-0.711937
H	4.493178	2.180634	-0.001442
H	4.067943	0.804950	1.032712
C	-0.351102	3.351212	0.640157
H	-0.723180	4.355835	0.400992
H	-1.043223	2.911190	1.368255
H	0.615912	3.479632	1.139584
C	-0.995117	1.074580	-3.051054
H	-2.005017	0.959083	-2.633272
H	-0.851927	2.136552	-3.281613
H	-0.966180	0.524322	-3.999562

42

B_Me_conf_1 Eopt -870.989875

C	-1.682886	-2.051963	-0.650203
C	-1.506269	-0.607226	-1.166213
C	-1.845028	0.251944	1.274132
C	-1.973894	-1.217636	1.728510
H	-0.693726	-2.493638	-0.462040
H	-2.627755	-1.271617	2.612629
C	-2.894128	-0.001235	-1.567728
H	-3.650843	-0.799945	-1.564042
C	-3.229864	0.849608	0.847302
H	-3.402534	1.784529	1.396354
H	-0.862840	-0.638877	-2.054113
H	-1.466218	0.837748	2.119299
C	-3.357618	1.144630	-0.656883
H	-2.753662	2.035602	-0.892920
H	-4.395760	1.414515	-0.893911
C	-2.514176	-2.132146	0.629695
H	-2.540004	-3.169747	0.990312
H	-3.558163	-1.860972	0.410136
H	-2.846002	0.371819	-2.599055
H	-4.031138	0.160239	1.152913
H	-2.154853	-2.660274	-1.437232
H	-0.984207	-1.584759	2.047354
B	-0.949215	0.347261	-0.026685
C	0.182976	1.473063	-0.255993
C	1.523451	1.060935	0.420118
C	2.723728	1.874008	-0.044823
H	3.625573	1.559269	0.492660
H	2.908039	1.776729	-1.118625
C	1.468883	1.027858	1.941869
H	0.645012	0.413502	2.318805
H	1.344402	2.053898	2.316489
C	-0.292267	2.799921	0.382781
H	0.493733	3.566400	0.344298
H	-0.605608	2.683179	1.425732
H	2.407832	0.637313	2.348187
C	0.372244	1.731035	-1.758090
H	0.875245	0.892908	-2.257518
H	-0.605598	1.870024	-2.240111
H	0.958084	2.642047	-1.940097
I	1.989014	-1.043431	-0.172280
H	2.545680	2.935478	0.183527
H	-1.147247	3.193371	-0.181631

42

B_Me_conf_2 Eopt -870.989756

C	3.520850	1.012739	0.017518
C	2.433023	0.538296	-0.978734
C	1.242085	-0.686714	0.983245
C	2.359713	-0.174397	1.928601
H	3.260680	2.017930	0.387791
H	2.501928	-0.901733	2.742021
C	2.785949	-0.841443	-1.608137
H	3.807643	-1.138125	-1.324228
C	1.616440	-2.046002	0.318728
H	0.819951	-2.773062	0.525769
H	2.380602	1.289836	-1.775734
H	0.348358	-0.841793	1.592804
C	1.803554	-1.943975	-1.198837
H	0.820987	-1.721534	-1.653065
H	2.122745	-2.912183	-1.608726
C	3.684918	0.073058	1.210508
H	4.417475	0.490357	1.915087
H	4.101141	-0.886246	0.869152
H	2.784803	-0.754122	-2.702655
H	2.533301	-2.451208	0.774036
H	4.478424	1.116066	-0.515190
H	2.030998	0.764689	2.405215
B	1.075439	0.398555	-0.161836
C	-0.159382	1.361470	-0.497154
C	-1.472511	1.132887	0.298509
C	-1.320765	1.373331	1.795014
H	-1.264276	2.458962	1.962177
H	-2.191602	1.000999	2.344518
C	-2.665508	1.931960	-0.218988
H	-2.946928	1.660627	-1.240524
H	-2.416648	3.003340	-0.198521
C	-0.434333	1.362482	-2.011559
H	0.499192	1.428001	-2.580138
H	-1.048523	2.226491	-2.299178
H	-3.534110	1.776992	0.431456
C	0.403383	2.762921	-0.125769
H	0.792552	2.808862	0.900073
H	1.221774	3.019683	-0.809442
H	-0.366867	3.538575	-0.230929
I	-2.112055	-0.994840	0.028970
H	-0.416320	0.920805	2.212022
H	-0.960082	0.451849	-2.327716

42

B_Me_conf_3 Eopt -870.989587

C	-1.616616	-2.055410	0.308106
C	-1.327376	-0.979358	-0.758487
C	-2.313067	0.818083	0.831271
C	-2.713242	-0.286746	1.831236
H	-0.691614	-2.208793	0.887923
H	-3.687134	-0.041143	2.283624
C	-2.496025	-0.728613	-1.752650
H	-2.783723	-1.682077	-2.222288
C	-3.404219	1.129572	-0.233679
H	-3.060194	1.975377	-0.852111
H	-0.473657	-1.322659	-1.355942
H	-2.151442	1.743063	1.402092
C	-3.739163	-0.043806	-1.162852
H	-4.360877	0.327558	-1.989614
H	-4.361273	-0.770888	-0.631107
C	-2.760191	-1.719418	1.273914
H	-2.728964	-2.423926	2.117088

H -3.720501 -1.901830 0.780069
H -2.108954 -0.093449 -2.565879
H -4.317258 1.476613 0.273563
H -1.828790 -3.017848 -0.183101
H -1.984524 -0.261368 2.655993
B -1.077479 0.411969 -0.059431
C 0.166238 1.381652 -0.365722
C 1.358288 0.964198 0.556305
C 2.483207 1.990559 0.605551
H 3.343540 1.591045 1.153076
H 2.815142 2.293337 -0.393956
C 0.893472 0.618605 1.968106
H 0.348071 1.475201 2.390864
H 1.748515 0.403670 2.618913
C -0.195487 2.846371 -0.025529
H -1.152593 3.108815 -0.493680
H 0.560012 3.539192 -0.418930
H 0.229200 -0.255287 1.982419
C 0.512083 1.353093 -1.862674
H 1.447474 1.892130 -2.068995
H 0.614507 0.335074 -2.252835
H -0.283507 1.854825 -2.431143
I 2.321781 -0.900679 -0.186057
H 2.130112 2.881369 1.143497
H -0.295962 3.025445 1.051252
42
B_Me_conf_4 Eopt -870.988364
C 3.622253 0.840675 0.014587
C 2.450800 0.541782 -0.965177
C 1.242254 -0.770050 0.925890
C 2.429644 -0.438961 1.878620
H 4.048203 1.825296 -0.220165
H 3.130084 -1.287651 1.911935
C 2.655626 -0.795114 -1.723313
H 3.569092 -0.731990 -2.333898
C 1.508101 -2.078462 0.129944
H 0.625377 -2.315142 -0.483620
H 2.410392 1.338339 -1.717714
H 0.345361 -0.947330 1.525839
C 2.732134 -1.994518 -0.779876
H 2.822294 -2.921375 -1.362912
H 3.644944 -1.926463 -0.169953
C 3.191233 0.829920 1.484706
H 2.534146 1.698365 1.660812
H 4.064359 0.966255 2.137815
H 1.819063 -0.947356 -2.426352
H 1.623063 -2.907228 0.844791
H 4.435514 0.113189 -0.131747
H 2.048129 -0.318233 2.901457
B 1.088579 0.373432 -0.163968
C -0.158085 1.314230 -0.533604
C -1.472816 1.114680 0.269975
C -1.298599 1.355969 1.764430
H -2.172385 1.008373 2.325226
H -0.401692 0.876574 2.169614
C -2.653884 1.937050 -0.240987
H -2.937428 1.677748 -1.265263
H -2.395943 3.005262 -0.209566
C -0.418774 1.262097 -2.049775
H -0.913690 0.328221 -2.346383
H 0.519936 1.340197 -2.609444
H -3.525397 1.785539 0.406289
C 0.385367 2.736757 -0.211546
H 1.214248 2.974211 -0.888395
H -0.388361 3.500198 -0.363661
H 0.753780 2.830378 0.817743
I -2.176320 -0.990726 0.024588
H -1.209451 2.439395 1.930053
H -1.053064 2.100200 -2.369082
42
B_Me_conf_5 Eopt -870.992380
C 1.830182 -1.213599 1.642265
C 1.717353 0.277693 1.268818
C 1.568143 -0.381507 -1.234930
C 1.860128 -1.849476 -0.861348
H 0.820345 -1.581959 1.884998
H 2.534673 -2.294508 -1.610325
C 3.085321 0.967045 0.991973
H 3.754445 0.792916 1.849016
C 2.848056 0.459601 -1.510871
H 2.548561 1.477694 -1.811964
H 1.282049 0.809995 2.124188
H 0.975903 -0.383182 -2.160325
C 3.795258 0.563285 -0.310380
H 4.570737 1.309768 -0.532541
H 4.326942 -0.384981 -0.180915
C 2.433714 -2.099852 0.545148
H 2.263192 -3.153204 0.808679
H 3.522139 -1.974904 0.540281
H 2.907574 2.053293 0.960536
H 3.383136 0.033457 -2.372908
H 2.426320 -1.322801 2.561679
H 0.909686 -2.396196 -0.944738
B 0.889726 0.434262 -0.068998
C -0.287191 1.510443 -0.295380
C -1.597282 1.033720 0.399034
C -1.555722 1.039029 1.920810
H -2.482434 0.618049 2.325359
H -0.713357 0.471068 2.324506
C -2.850762 1.749038 -0.084389
H -2.738369 2.826159 0.113113
H -3.727369 1.392855 0.468583
C -0.528317 1.760014 -1.791346
H 0.422084 1.963407 -2.299747
H -1.175224 2.632773 -1.952572
H -3.034111 1.610044 -1.153315
C 0.157856 2.851306 0.331289
H -0.646401 3.599674 0.292791
H 0.481468 2.746179 1.372994
H 1.002160 3.256162 -0.243496
I -1.895298 -1.117855 -0.152288
H -1.480958 2.079104 2.269991
H -0.991467 0.893731 -2.281772
42
B_Me_conf_6 Eopt -870.990985
C -1.727001 -2.071715 -0.479447
C -1.473976 -0.680396 -1.122046
C -2.028857 0.435565 1.166153
C -2.293387 -0.970862 1.768778
H -1.034129 -2.800463 -0.921587
H -3.372416 -1.188916 1.762566
C -2.785909 -0.068013 -1.698572
H -3.175840 -0.734815 -2.482074
C -3.323741 1.022173 0.526720
H -3.127983 2.040760 0.155960
H -0.770621 -0.783846 -1.957158
H -1.726656 1.123753 1.964496
C -3.849774 0.166008 -0.625809
H -4.728269 0.649554 -1.074624
H -4.198107 -0.801635 -0.236736
C -1.539232 -2.087424 1.041918
H -0.464665 -1.966780 1.253947
H -1.830096 -3.066798 1.447201
H -2.560759 0.891975 -2.191418
H -4.091273 1.118569 1.309035
H -2.740138 -2.425091 -0.727328
H -1.992351 -0.974880 2.825225
B -1.023808 0.393064 -0.056848
C 0.138846 1.478324 -0.308129
C 1.420055 1.067077 0.480128
C 2.624249 1.960875 0.214216
H 3.496080 1.596149 0.768852
H 2.885320 2.009925 -0.847195
C 1.200709 0.914270 1.980566
H 0.919949 1.890291 2.401630
H 2.126940 0.593549 2.469519
C -0.342237 2.856801 0.205185
H -0.664939 2.840882 1.251334
H -1.196341 3.185159 -0.401895
H 0.413607 0.191855 2.222922

C 0.427977 1.644666 -1.808460
H 0.908899 0.759485 -2.240548
H -0.508724 1.816186 -2.354272
H 1.073755 2.512545 -1.997848
I 2.074550 -0.963138 -0.204299
H 2.403186 2.977532 0.569484
H 0.437130 3.621940 0.095406
54
B'_Et_conf_1 Eopt -1028.044313
C 3.585530 -1.184718 1.029791
C 2.458336 -0.151913 1.291396
C 2.393803 0.374473 -1.239417
C 3.626440 -0.541209 -1.462977
H 3.124227 -2.180433 0.929087
H 4.287997 -0.069441 -2.206105
C 2.968072 1.281917 1.571927
H 3.707636 1.254182 2.387379
C 2.777360 1.841966 -0.933247
H 1.855959 2.444855 -0.888949
H 1.931713 -0.490475 2.189425
H 1.842683 0.369267 -2.187598
C 3.561895 2.034055 0.370150
H 3.596642 3.106236 0.608870
H 4.604045 1.736347 0.213746
C 4.441129 -0.901279 -0.209881
H 5.051284 -1.788452 -0.430276
H 5.156403 -0.104044 0.017917
H 2.116280 1.864494 1.955467
H 3.361802 2.245938 -1.773874
H 4.233425 -1.242210 1.917556
H 3.267781 -1.470737 -1.928014
B 1.557453 -0.169065 -0.005802
C 0.047567 -0.771575 -0.081890
C -0.929359 0.433263 -0.225915
C -0.630642 1.503486 0.846899
H 0.081869 2.187635 0.356263
C -0.927613 1.052230 -1.632725
H -1.284296 0.314284 -2.358244
C -0.244990 -1.684015 1.146339
H 0.643498 -2.312839 1.299061
H 0.125191 1.247024 -1.875775
C 0.001969 -1.682418 -1.336262
H 0.343719 -1.118683 -2.211011
H -1.044503 -1.962123 -1.533144
I -3.057033 -0.338612 -0.027802
H -0.072507 1.044012 1.664679
H -1.044037 -2.376259 0.842046
C -1.695696 2.355047 -1.822792
H -1.625113 2.661711 -2.873296
H -2.758777 2.243965 -1.576715
H -1.280611 3.168576 -1.214631
C -1.760427 2.326761 1.455241
H -1.331639 3.150633 2.040122
H -2.427648 2.753804 0.700496
H -2.367871 1.713602 2.131257
C 0.841027 -2.957427 -1.228847
H 0.935992 -3.433783 -2.212266
H 1.856566 -2.750011 -0.863356
H 0.390700 -3.690371 -0.549616
C -0.643850 -1.118002 2.511780
H 0.144603 -0.527202 2.990368
H -1.547296 -0.499889 2.465020
H -0.863544 -1.961744 3.179208
54
B'_Et_conf_10 Eopt -1028.042335
C -3.225358 -1.351672 -1.452369
C -2.551272 0.045072 -1.339118
C -2.350345 -0.294481 1.226997
C -2.997835 -1.702566 1.068309
H -2.952380 -1.805268 -2.414407
H -4.070520 -1.647187 1.307577
C -3.597876 1.165141 -1.063245
H -4.296676 1.212525 -1.911400
C -3.440258 0.792264 1.444891
H -2.960339 1.756565 1.665987
H -2.073973 0.285214 -2.294177
H -1.725788 -0.294295 2.123948
C -4.367919 0.948604 0.239899
H -5.056151 1.788304 0.407540
H -4.998029 0.052258 0.146456
C -2.825830 -2.309486 -0.326992
H -1.766057 -2.582644 -0.465066
H -3.396794 -3.245083 -0.403286
H -3.090555 2.142930 -1.025544
H -4.025799 0.526373 2.337451
H -4.320272 -1.244451 -1.468402
H -2.558986 -2.385657 1.808626
B -1.578747 0.131206 -0.091520
C -0.057274 0.730384 -0.146970
C 0.861927 -0.528313 -0.057734
C 0.666121 -1.476525 1.140910
H 1.364736 -2.304219 0.987267
C 0.792121 -1.323465 -1.379583
H 1.261389 -0.728443 -2.169485
C 0.119881 1.713144 1.038359
H 1.190243 1.900547 1.200497
H -0.274203 -1.389196 -1.652381
C 0.069233 1.508877 -1.485750
H -0.787563 2.194872 -1.516580
H -0.096278 0.831877 -2.328298
I 3.067001 0.031026 0.121107
H -0.335924 -1.920586 1.034624
H -0.250071 1.253172 1.957847
C 1.380076 -2.728773 -1.401176
H 2.417504 -2.739376 -1.042543
H 0.796924 -3.430384 -0.792304
H 1.377793 -3.104664 -2.431091
C 0.862042 -1.003625 2.580482
H 1.779415 -0.412779 2.687272
H 0.030662 -0.412937 2.971454
H 0.960913 -1.885793 3.224946
C 1.335944 2.323524 -1.764422
H 1.717383 2.835048 -0.872259
H 2.147430 1.702131 -2.156030
H 1.113378 3.088583 -2.520467
C -0.585416 3.061089 0.867942
H -0.093608 3.693746 0.120028
H -1.632641 2.945037 0.559809
H -0.580237 3.608233 1.818717
54
B'_Et_conf_11 Eopt -1028.040404
C 3.796028 1.235141 -0.703179
C 2.603956 0.374841 -1.203825
C 2.295829 -0.564679 1.188470
C 3.440553 0.363642 1.672144
H 3.442457 2.262850 -0.536423
H 3.958636 -0.115716 2.516671
C 3.057055 -1.022219 -1.696421
H 3.840368 -0.892959 -2.458874
C 2.779288 -1.954352 0.711369
H 1.891809 -2.596722 0.595746
H 2.170132 0.894758 -2.068564
H 1.646215 -0.724062 2.056698
C 3.576694 -1.973573 -0.605100
H 3.571750 -2.998181 -1.002099
H 4.628074 -1.749632 -0.399796
C 4.457041 0.754263 0.595233
H 5.093686 1.560982 0.984686
H 5.132847 -0.083586 0.396122
H 2.212017 -1.495741 -2.217200
H 3.383936 -2.421984 1.503839
H 4.549166 1.301095 -1.503318
H 2.986615 1.283572 2.075646
B 1.583083 0.200120 -0.001880
C 0.073167 0.805244 0.076272
C -0.881908 -0.445527 0.119752
C -0.503747 -1.397138 -1.030214
H 0.570871 -1.604880 -0.898986
C -0.884805 -1.176888 1.477340
H -0.674243 -0.460296 2.274971

C -0.207698 1.696218 -1.159984
H -1.269925 1.956358 -1.194131
H -0.018158 -1.852461 1.435089
C 0.087883 1.679713 1.357969
H 0.861948 2.445933 1.208778
H 0.442623 1.096335 2.215429
I -3.031208 0.132593 -0.340705
H -0.598860 -0.870813 -1.986112
H -0.010877 1.120272 -2.072821
C -2.097429 -1.995051 1.922347
H -2.486654 -2.654864 1.142117
H -1.805149 -2.616208 2.779142
H -2.914811 -1.342378 2.247145
C -1.191642 -2.752610 -1.122563
H -0.829058 -3.276790 -2.015060
H -0.963270 -3.383065 -0.254702
H -2.280466 -2.656160 -1.209735
C -1.214067 2.379426 1.740244
H -1.027052 3.097954 2.548923
H -1.650071 2.927251 0.894707
H -1.966870 1.664844 2.094616
C 0.572446 3.007974 -1.226984
H 0.204087 3.732169 -0.489220
H 1.645530 2.876308 -1.055036
H 0.449462 3.461247 -2.218694
54
B'_Et_conf_12 Eopt -1028.044226
C -3.485842 1.032725 1.392995
C -2.628101 -0.238010 1.191257
C -2.218744 0.451507 -1.265459
C -2.927732 1.795104 -0.979688
H -2.883241 1.764334 1.949928
H -3.380994 2.169325 -1.909890
C -3.476211 -1.450304 0.717873
H -4.272689 -1.630080 1.455507
C -3.207661 -0.647017 -1.748430
H -2.614770 -1.431589 -2.236636
H -2.216715 -0.510534 2.171422
H -1.517707 0.613897 -2.092987
C -4.109960 -1.290771 -0.674473
H -4.424790 -2.281330 -1.031795
H -5.034426 -0.712709 -0.575533
C -3.993286 1.712917 0.114219
H -4.330925 2.728540 0.363996
H -4.877217 1.195972 -0.274518
H -2.845871 -2.350757 0.738259
H -3.843445 -0.219848 -2.539482
H -4.341003 0.787537 2.042062
H -2.177918 2.546663 -0.682813
B -1.525073 -0.070146 0.063232
C 0.022303 -0.621559 0.166254
C 1.023087 0.580835 0.146540
C 0.767812 1.572548 -0.999303
H -0.279481 1.881139 -0.922607
C 1.169149 1.292984 1.496019
H 1.948584 2.055548 1.400873
C 0.199796 -1.526588 -1.088903
H 1.263059 -1.764779 -1.204925
H 1.525604 0.586184 2.253612
C 0.111387 -1.474915 1.456940
H -0.742146 -2.164639 1.431140
H -0.076293 -0.836334 2.328147
I 3.169103 -0.110887 -0.255252
H 0.857213 1.046623 -1.956824
H -0.081019 -0.963278 -1.987687
C -0.126781 1.948375 1.972029
H -0.629054 2.504500 1.167524
H -0.828377 1.205612 2.364752
H 0.087121 2.659111 2.779453
C 1.597221 2.851812 -1.024317
H 1.348699 3.422538 -1.927285
H 1.375710 3.492791 -0.161448
H 2.674953 2.653647 -1.039526
C 1.368677 -2.303765 1.723410
H 1.152037 -3.045690 2.503791
H 1.710132 -2.845486 0.832302
H 2.204119 -1.690013 2.075555
C -0.558688 -2.854691 -1.073262
H -0.165134 -3.536380 -0.309417
H -1.632284 -2.738820 -0.889537
H -0.447381 -3.351074 -2.045400
54
B'_Et_conf_13 Eopt -1028.043435
C 3.073493 -0.385301 1.909035
C 2.135478 0.620427 1.189512
C 2.675095 -0.523343 -1.058607
C 3.502166 -1.596454 -0.297914
H 2.437881 -1.148484 2.384932
H 4.304123 -1.964330 -0.955747
C 2.856967 1.897714 0.697786
H 3.289652 2.423670 1.562349
C 3.538750 0.694638 -1.473453
H 2.978293 1.264259 -2.228211
H 1.405196 0.940662 1.937487
H 2.320876 -0.984088 -1.989046
C 3.949828 1.660102 -0.349625
H 4.224894 2.624581 -0.798775
H 4.857398 1.302538 0.146890
C 4.097985 -1.109206 1.025552
H 4.488967 -1.974866 1.577939
H 4.964070 -0.468808 0.832722
H 2.104350 2.581999 0.273921
H 4.442283 0.330457 -1.987255
H 3.591445 0.133758 2.730418
H 2.860422 -2.464100 -0.083017
B 1.498745 -0.109478 -0.070553
C -0.044707 -0.594355 -0.243398
C -1.075851 0.582044 -0.217706
C -0.890861 1.611354 0.903997
H 0.122905 2.008519 0.776479
C -1.207237 1.299550 -1.571284
H -2.025031 2.024211 -1.505448
C -0.194666 -1.645166 0.910935
H 0.700065 -2.286331 0.877611
H -1.496213 0.592255 -2.352529
C -0.229560 -1.375177 -1.579479
H 0.188818 -0.779470 -2.400763
H -1.305522 -1.463266 -1.783542
I -3.152831 -0.294960 0.080330
H -0.899351 1.116046 1.876465
H -1.025645 -2.305608 0.626463
C 0.075381 2.020445 -1.979328
H -0.045898 2.465498 -2.974478
H 0.337357 2.825351 -1.281044
H 0.923503 1.326742 -2.039331
C -1.840368 2.805483 0.929045
H -1.712458 3.452768 0.052981
H -2.891386 2.497925 0.980235
H -1.625919 3.413396 1.816354
C 0.349502 -2.787829 -1.672311
H 0.259354 -3.144139 -2.706463
H 1.407932 -2.840818 -1.400617
H -0.194957 -3.496967 -1.038355
C -0.423449 -1.234282 2.364164
H 0.329431 -0.540494 2.749742
H -1.412904 -0.787733 2.509993
H -0.385295 -2.138743 2.985374
54
B'_Et_conf_14 Eopt -1028.041969
C -3.550530 1.094254 0.941254
C -2.361566 0.141703 1.243284
C -2.291225 -0.492073 -1.254648
C -3.569566 0.349823 -1.505431
H -3.159860 2.106682 0.763627
H -4.197645 -0.169568 -2.245763
C -2.823603 -1.292553 1.601156
H -3.593611 -1.230873 2.385939
C -2.602674 -1.963153 -0.883586
H -1.655038 -2.522466 -0.838254
H -1.859232 0.536114 2.130932

H -1.752325 -0.509140 -2.209923
C -3.354928 -2.150861 0.440161
H -3.298435 -3.209026 0.730876
H -4.419458 -1.951468 0.281942
C -4.404598 0.685206 -0.261973
H -5.087947 1.510024 -0.507833
H -5.047857 -0.158407 0.007985
H -1.971329 -1.811465 2.063699
H -3.182427 -2.423390 -1.698121
H -4.183163 1.166051 1.838840
H -3.261535 1.293256 -1.979834
B -1.461871 0.154182 -0.062089
C 0.051775 0.749541 -0.221336
C 0.983847 -0.515097 -0.146394
C 0.721259 -1.473180 1.022233
H 1.467545 -2.271744 0.964859
C 1.005575 -1.315645 -1.463926
H 1.470019 -0.705157 -2.243282
C 0.456543 1.810989 0.847110
H 1.273921 2.390523 0.396034
H -0.040219 -1.450691 -1.766667
C 0.197075 1.406902 -1.619310
H -0.142446 0.716251 -2.396189
H 1.269366 1.578983 -1.804237
I 3.150500 0.129377 0.094938
H -0.240838 -1.961079 0.785552
H 0.910552 1.321461 1.711081
C 1.672168 -2.687520 -1.445308
H 2.689855 -2.643231 -1.036563
H 1.097917 -3.418941 -0.863863
H 1.740377 -3.066179 -2.472047
C 0.693119 -0.979182 2.462410
H -0.112209 -0.266355 2.659803
H 0.545095 -1.841755 3.123912
H 1.647206 -0.511008 2.736517
C -0.542279 2.728345 -1.816566
H -0.485421 3.029033 -2.870138
H -1.605921 2.647802 -1.551432
H -0.107074 3.538799 -1.220041
C -0.587434 2.805817 1.369040
H -1.275980 3.159292 0.596825
H -1.187447 2.392768 2.186143
H -0.069318 3.687138 1.769311
54
B'_Et_conf_15 Eopt -1028.039848
C 3.817521 1.089765 -0.894345
C 2.616595 0.157762 -1.221654
C 2.342355 -0.314575 1.317262
C 3.499775 0.689311 1.577962
H 3.485229 2.135884 -0.846171
H 4.010119 0.405621 2.510400
C 3.123945 -1.306663 -1.422301
H 4.220046 -1.298768 -1.514016
C 2.900461 -1.753399 1.107744
H 2.399031 -2.438804 1.804351
H 2.177464 0.511993 -2.162382
H 1.710154 -0.309703 2.210188
C 2.715882 -2.289648 -0.316330
H 1.653752 -2.547846 -0.452249
H 3.270260 -3.230417 -0.436640
C 4.500385 0.743032 0.425842
H 5.279990 1.485753 0.643565
H 5.016124 -0.224080 0.336793
H 2.744631 -1.688862 -2.379544
H 3.966106 -1.781089 1.376615
H 4.539513 1.033769 -1.722612
H 3.085785 1.697447 1.742617
B 1.611430 0.210337 0.007076
C 0.085579 0.806934 -0.022403
C -0.851455 -0.448111 -0.025989
C -0.658197 -1.231001 -1.343971
H 0.423519 -1.255347 -1.545479
C -0.675213 -1.318036 1.231438
H -0.422178 -0.669202 2.073642
C -0.140331 1.652398 -1.299027
H -1.210605 1.873686 -1.402643
H 0.222742 -1.923733 1.036080
C 0.027726 1.724715 1.231806
H 0.902688 2.387172 1.156048
H 0.206261 1.142449 2.141421
I -3.041134 0.151665 -0.118989
H -1.089270 -0.655150 -2.167925
H 0.131195 1.059494 -2.178728
C -1.786281 -2.252715 1.709470
H -1.368586 -2.937489 2.459356
H -2.592961 -1.687902 2.188386
H -2.227742 -2.854305 0.911337
C -1.183256 -2.657307 -1.414464
H -0.714545 -3.307152 -0.664594
H -2.271136 -2.696734 -1.279647
H -0.954911 -3.072131 -2.403680
C -1.199300 2.613648 1.458826
H -0.927841 3.434528 2.135965
H -1.577833 3.056032 0.529357
H -2.024224 2.064050 1.922089
C 0.627736 2.969638 -1.362770
H 0.500948 3.427145 -2.352008
H 0.273863 3.692999 -0.618139
H 1.701819 2.826304 -1.203266
54
B'_Et_conf_16 Eopt -1028.041864
C -3.693043 0.783488 1.092200
C -2.366962 -0.024678 1.269112
C -2.320790 -0.471791 -1.286618
C -3.659122 0.314440 -1.442851
H -3.689508 1.631492 1.789523
H -4.496064 -0.396232 -1.511826
C -2.708760 -1.518923 1.513745
H -3.367867 -1.582715 2.392436
C -2.601058 -1.970092 -0.983393
H -1.650571 -2.523854 -0.919272
H -1.862485 0.343108 2.166101
H -1.785080 -0.426529 -2.241176
C -3.386816 -2.174332 0.311447
H -3.519975 -3.248497 0.498882
H -4.398318 -1.759167 0.192403
C -3.932879 1.323331 -0.321581
H -3.279372 2.194609 -0.473241
H -4.961238 1.701101 -0.404266
H -1.800329 -2.078927 1.774265
H -3.154462 -2.404215 -1.829330
H -4.541514 0.149595 1.389350
H -3.644700 0.855888 -2.397804
B -1.489896 0.094119 -0.056076
C 0.022219 0.701621 -0.181400
C 1.002865 -0.524241 -0.167920
C 0.797246 -1.561820 0.940909
H 1.598324 -2.301718 0.846630
C 1.051240 -1.250933 -1.527341
H 1.489683 -0.584526 -2.274731
C 0.342182 1.722836 0.949872
H 1.157220 2.354012 0.572587
H 0.006868 -1.407348 -1.831818
C 0.174973 1.439322 -1.540880
H -0.121183 0.779210 -2.360876
H 1.246177 1.651362 -1.683007
I 3.137047 0.208377 0.117554
H -0.123766 -2.100893 0.660483
H 0.765390 1.207916 1.814320
C 1.771577 -2.594149 -1.584385
H 1.846719 -2.916011 -2.629825
H 2.790310 -2.527536 -1.180840
H 1.234026 -3.378057 -1.037609
C 0.725708 -1.154598 2.405851
H 1.643408 -0.638478 2.715905
H -0.128219 -0.511724 2.635483
H 0.633146 -2.061592 3.016252
C -0.593642 2.746139 -1.709772
H -1.676823 2.598980 -1.614093
H -0.285669 3.511974 -0.988226

H -0.407564 3.147542 -2.713862
C -0.799976 2.638505 1.429226
H -1.637471 2.681949 0.725269
H -1.204544 2.314160 2.394421
H -0.438345 3.665787 1.560167
54
B'_Et_conf_17 Eopt -1028.039989
C -2.932958 2.013853 0.141105
C -2.307716 0.852707 0.962496
C -2.800105 -0.736388 -1.025707
C -3.398596 0.471954 -1.796101
H -2.142058 2.557320 -0.398263
H -4.175710 0.104051 -2.482640
C -3.408914 0.128783 1.798799
H -4.329781 0.730881 1.785516
C -3.903495 -1.442953 -0.179343
H -3.914072 -2.512950 -0.425769
H -1.604141 1.314404 1.659765
H -2.433533 -1.443590 -1.776999
C -3.723240 -1.298471 1.336578
H -2.898205 -1.952840 1.653511
H -4.619652 -1.666527 1.854471
C -3.974200 1.536765 -0.867089
H -4.338328 2.388949 -1.456888
H -4.849737 1.134915 -0.336708
H -3.089451 0.088105 2.848715
H -4.892133 -1.056375 -0.468983
H -3.384043 2.732517 0.841250
H -2.624671 0.938029 -2.425313
B -1.656271 -0.166802 -0.076078
C -0.126528 -0.714343 -0.142803
C 0.888629 0.356966 0.364235
C 0.552741 1.742546 -0.237778
H -0.099129 2.215824 0.508872
C 0.990752 0.409317 1.896694
H 1.423904 -0.526435 2.264082
C 0.195089 -1.278867 -1.546062
H -0.547883 -2.057140 -1.758435
H -0.039191 0.430697 2.275621
C -0.291657 -1.944732 0.807610
H -1.132070 -2.536245 0.413246
H -0.605321 -1.612349 1.806293
I 3.016889 -0.197803 -0.248401
H -0.073397 1.598284 -1.124945
H 1.155828 -1.802034 -1.505720
C 1.744025 1.586678 2.503665
H 1.272312 2.546377 2.257674
H 1.744900 1.490286 3.596056
H 2.788184 1.617534 2.168912
C 1.650897 2.736182 -0.598939
H 2.205923 2.410589 -1.486188
H 1.190312 3.706724 -0.825273
H 2.370966 2.881972 0.212630
C 0.894135 -2.900329 0.961771
H 1.758103 -2.424215 1.436494
H 0.589847 -3.746711 1.590970
H 1.223450 -3.305950 -0.002182
C 0.235464 -0.307585 -2.720630
H -0.706732 0.244053 -2.831490
H 1.050812 0.420829 -2.622292
H 0.405013 -0.863902 -3.651375
54
B'_Et_conf_18 Eopt -1028.043589
C -3.458824 1.169261 1.244831
C -2.667015 -0.168657 1.165773
C -2.214375 0.278791 -1.352284
C -2.989467 1.625753 -1.238157
H -3.306038 1.617624 2.235915
H -4.010316 1.494884 -1.626596
C -3.607291 -1.337320 0.750242
H -4.399328 -1.433467 1.507557
C -3.199793 -0.864002 -1.724496
H -2.640601 -1.782153 -1.947192
H -2.280429 -0.412173 2.161554
H -1.496815 0.356127 -2.175505
C -4.234383 -1.133896 -0.630337
H -4.835905 -2.013804 -0.896808
H -4.937249 -0.289108 -0.584018
C -3.055165 2.194774 0.181504
H -2.061848 2.586154 0.443673
H -3.739636 3.053899 0.208895
H -3.050270 -2.286070 0.769104
H -3.713594 -0.585838 -2.656735
H -4.537625 0.968069 1.169165
H -2.512981 2.370618 -1.890295
B -1.551245 -0.129716 0.037461
C -0.010484 -0.669434 0.224591
C 0.984159 0.547674 0.180123
C 0.633509 1.566180 -0.912946
H -0.389058 1.905356 -0.693589
C 1.215173 1.228443 1.538826
H 2.041544 1.939244 1.434377
C 0.200708 -1.654695 -0.963998
H 1.264713 -1.895533 -1.048762
H 1.549700 0.485258 2.268817
C 0.058803 -1.451363 1.559678
H -0.781582 -2.158686 1.556154
H -0.155772 -0.772106 2.393424
I 3.104748 -0.122333 -0.348702
H 0.581305 1.053645 -1.880777
H -0.067795 -1.158907 -1.905403
C 0.002025 1.982389 2.079389
H -0.229157 2.858975 1.462327
H -0.892860 1.352115 2.135625
H 0.218325 2.337929 3.094247
C 1.496149 2.817145 -1.032009
H 2.534872 2.582653 -1.288440
H 1.091180 3.455542 -1.826857
H 1.493821 3.405380 -0.106022
C 1.334878 -2.232805 1.875869
H 1.148441 -2.912356 2.718177
H 1.673468 -2.838683 1.026030
H 2.165724 -1.577175 2.158435
C -0.543879 -2.988328 -0.851957
H -1.594922 -2.878910 -0.564921
H -0.518661 -3.510948 -1.816177
H -0.074448 -3.644760 -0.109336
54
B'_Et_conf_2 Eopt -1028.044534
C 2.821593 -1.854527 1.043975
C 2.421820 -0.375726 1.278012
C 2.489252 -0.006889 -1.295810
C 2.918012 -1.487139 -1.474315
H 1.914743 -2.480191 1.010834
H 3.586735 -1.556087 -2.345500
C 3.684692 0.531776 1.424456
H 4.584646 -0.096491 1.504254
C 3.756816 0.891555 -1.109901
H 3.757771 1.683616 -1.870046
H 1.867579 -0.330049 2.220714
H 1.966355 0.300959 -2.209895
C 3.860027 1.532996 0.278021
H 3.077237 2.304196 0.370364
H 4.820394 2.056870 0.379212
C 3.623322 -2.055359 -0.242227
H 3.823440 -3.125247 -0.391412
H 4.607116 -1.576090 -0.132138
H 3.615752 1.089336 2.367573
H 4.659521 0.292181 -1.297302
H 3.407799 -2.207410 1.905613
H 2.040832 -2.105991 -1.710356
B 1.594332 0.117651 0.012703
C 0.091397 0.758931 0.086892
C -0.894652 -0.455671 0.168534
C -0.510227 -1.504660 -0.895297
H 0.272425 -2.108178 -0.405832
C -0.954759 -1.054648 1.579700
H -1.392267 -0.316307 2.261308
C -0.175568 1.620896 -1.168661
H -1.198818 2.008307 -1.122316

H 0.089971 -1.185210 1.894671
C 0.120316 1.661293 1.349468
H 1.010722 2.302060 1.262236
H 0.305822 1.047789 2.236970
I -3.034184 0.180596 -0.242086
H -0.021635 -0.996300 -1.733386
H -0.140257 0.988171 -2.065076
C -1.661665 -2.392061 1.760411
H -1.648626 -2.661982 2.823289
H -2.709064 -2.350803 1.437632
H -1.157198 -3.196475 1.210612
C -1.544458 -2.458610 -1.487222
H -1.020564 -3.254930 -2.032330
H -2.183486 -2.925885 -0.733103
H -2.192342 -1.936448 -2.199351
C -1.084020 2.561718 1.621472
H -1.367343 3.155865 0.743318
H -1.963172 1.986026 1.931083
H -0.841283 3.260880 2.432597
C 0.755237 2.814907 -1.366163
H 0.503223 3.327314 -2.303287
H 0.651394 3.548012 -0.555627
H 1.809847 2.525948 -1.426346
54
B'_Et_conf_20 Eopt -1028.041569
C 2.673650 -1.848869 1.085405
C 2.296290 -0.344096 1.287993
C 2.507696 0.040994 -1.268523
C 2.900001 -1.456925 -1.466995
H 2.089383 -2.462798 1.783610
H 3.988430 -1.530577 -1.602571
C 3.601395 0.475329 1.490558
H 4.126180 0.076081 2.371334
C 3.782028 0.885455 -0.987116
H 3.517006 1.948218 -0.878354
H 1.707302 -0.250023 2.207211
H 2.073316 0.414202 -2.202529
C 4.518455 0.438408 0.271848
H 5.387953 1.087489 0.443350
H 4.917811 -0.576477 0.130879
C 2.455892 -2.392981 -0.334002
H 1.384595 -2.604480 -0.469224
H 2.963246 -3.361841 -0.435952
H 3.364125 1.522688 1.717107
H 4.446243 0.819698 -1.861731
H 3.725292 -1.992462 1.371056
H 2.457566 -1.821942 -2.403449
B 1.546257 0.192662 -0.010459
C 0.024795 0.804422 -0.087633
C -0.945866 -0.428011 -0.067905
C -0.875426 -1.210704 -1.390983
H 0.193368 -1.299901 -1.642596
C -0.731208 -1.316059 1.171822
H -0.331035 -0.702562 1.982869
C -0.160029 1.593042 -1.408810
H -1.228416 1.823007 -1.524130
H 0.079871 -2.000051 0.889158
C -0.280279 1.760291 1.101357
H -0.760031 1.210808 1.919343
H -1.044347 2.466597 0.749110
I -3.087472 0.332832 0.008517
H -1.312491 -0.604916 -2.190228
H 0.111943 0.963635 -2.261598
C -1.872728 -2.150326 1.750139
H -2.436612 -2.698850 0.990931
H -1.453225 -2.879585 2.455765
H -2.578631 -1.520535 2.301930
C -1.497543 -2.599134 -1.427925
H -1.032850 -3.280415 -0.703724
H -2.576006 -2.562823 -1.229154
H -1.356618 -3.028997 -2.426880
C 0.895875 2.550713 1.683856
H 1.636963 2.827002 0.923080
H 0.537505 3.479207 2.145940
H 1.414976 1.979624 2.461040
C 0.616016 2.902025 -1.522247
H 0.448799 3.343917 -2.512631
H 0.292512 3.638493 -0.776164
H 1.696533 2.753724 -1.406916
54
B'_Et_conf_21 Eopt -1028.042374
C 3.033260 -0.814540 1.820713
C 2.133312 0.358851 1.337853
C 2.698969 -0.292297 -1.107460
C 3.607238 -1.430948 -0.555213
H 2.424412 -1.720128 1.971336
H 4.431176 -1.599027 -1.264623
C 2.980117 1.655543 1.183472
H 3.974135 1.505879 1.629746
C 3.520011 1.024706 -1.251413
H 3.394944 1.413591 -2.271025
H 1.390838 0.535721 2.116519
H 2.355114 -0.601642 -2.101542
C 3.125869 2.128345 -0.263869
H 2.158772 2.542791 -0.579194
H 3.847781 2.954807 -0.320513
C 4.156765 -1.131266 0.836869
H 4.736038 -1.992102 1.197261
H 4.859686 -0.287390 0.789803
H 2.510211 2.461021 1.764038
H 4.592268 0.802820 -1.143772
H 3.451174 -0.553030 2.804179
H 3.045254 -2.374486 -0.499978
B 1.520081 -0.125324 -0.053935
C -0.012377 -0.624092 -0.306608
C -1.036098 0.561368 -0.218576
C -0.767384 1.544930 0.925409
H 0.244818 1.937145 0.740947
C -1.238780 1.321109 -1.542974
H -2.060774 2.031655 -1.407456
C -0.219386 -1.769034 0.743908
H 0.654281 -2.433291 0.663241
H -1.568401 0.633593 -2.325339
C -0.177528 -1.296752 -1.700163
H 0.192317 -0.614831 -2.475191
H -1.254085 -1.422930 -1.887474
I -3.119256 -0.287860 0.118387
H -0.709348 1.005123 1.874174
H -1.065028 -2.369833 0.380217
C -0.004151 2.086044 -2.011312
H 0.883258 1.442378 -2.079215
H -0.185485 2.498090 -3.011597
H 0.224897 2.924229 -1.342278
C -1.700369 2.742231 1.068243
H -1.395317 3.331952 1.941267
H -1.657665 3.404946 0.195461
H -2.742228 2.436254 1.218430
C 0.473148 -2.661171 -1.924878
H 0.346475 -2.948069 -2.976698
H 1.547038 -2.662014 -1.716558
H 0.009074 -3.446985 -1.318026
C -0.459841 -1.498561 2.227976
H 0.373819 -0.992510 2.722485
H -1.372882 -0.921010 2.404894
H -0.593022 -2.466313 2.729751
54
B'_Et_conf_22 Eopt -1028.040172
C -3.471531 0.194379 1.615095
C -2.187246 -0.555377 1.168020
C -2.548397 0.274755 -1.243894
C -3.694036 1.191251 -0.746983
H -3.163666 1.068107 2.203411
H -4.421984 1.327469 -1.561135
C -2.506970 -2.001041 0.689031
H -2.929882 -2.568080 1.531567
C -3.045033 -1.135703 -1.655785
H -2.236708 -1.621114 -2.223704
H -1.550678 -0.635337 2.056510
H -2.147525 0.730981 -2.155025
C -3.455655 -2.071157 -0.509640

H -3.474180 -3.103493 -0.885829
H -4.478972 -1.859638 -0.184814
C -4.417073 0.680191 0.502930
H -5.042875 1.488548 0.905717
H -5.115842 -0.112481 0.217791
H -1.576036 -2.518507 0.412833
H -3.882864 -1.025733 -2.361870
H -4.029588 -0.452047 2.310391
H -3.286092 2.192388 -0.538705
B -1.488821 0.166836 -0.061113
C 0.064755 0.697271 -0.173551
C 1.002791 -0.536996 0.002546
C 0.941323 -1.339749 1.312557
H 1.730468 -2.095867 1.246799
C 0.859989 -1.496755 -1.192630
H 1.197118 -0.990417 -2.101407
C 0.383288 1.803433 0.879711
H 1.025594 2.544608 0.388074
H -0.224469 -1.658847 -1.321288
C 0.259290 1.322971 -1.583379
H -0.101330 0.631836 -2.353015
H 1.334776 1.453807 -1.766138
I 3.171691 0.167578 -0.074037
H 0.002957 -1.905952 1.276765
H 1.000018 1.399557 1.680280
C 1.541435 -2.856488 -1.100253
H 1.459114 -3.369581 -2.065645
H 2.608982 -2.754986 -0.864497
H 1.080054 -3.501278 -0.342745
C 1.066677 -0.686165 2.684754
H 2.007204 -0.128736 2.781436
H 0.236857 -0.013205 2.926487
H 1.075745 -1.481343 3.440500
C -0.417001 2.677603 -1.800293
H -1.485366 2.659858 -1.557663
H 0.044745 3.470508 -1.199866
H -0.323073 2.972067 -2.853207
C -0.814075 2.511583 1.509132
H -1.580678 2.779130 0.768961
H -1.286837 1.879747 2.270810
H -0.495709 3.438199 2.003533
54
B'_Et_conf_23 Eopt -1028.038415
C -2.842324 1.619567 -1.303507
C -2.232593 1.324609 0.092005
C -2.724606 -1.191139 -0.269707
C -3.427279 -0.853904 -1.610507
H -2.024167 1.649887 -2.041014
H -4.284433 -1.531126 -1.748742
C -3.273745 1.350354 1.238977
H -3.827960 2.300746 1.195511
C -3.689756 -1.190407 0.936720
H -3.150379 -1.588135 1.808953
H -1.523405 2.132838 0.302526
H -2.321068 -2.208884 -0.368708
C -4.278256 0.184831 1.287616
H -4.707842 0.137940 2.298044
H -5.123084 0.396281 0.623998
C -3.887040 0.601848 -1.771580
H -4.110279 0.785461 -2.831876
H -4.829462 0.767814 -1.239760
H -2.717979 1.376018 2.188323
H -4.513350 -1.894986 0.743850
H -3.283995 2.627453 -1.296307
H -2.728065 -1.081237 -2.430042
B -1.584048 -0.114997 -0.019000
C -0.049892 -0.614307 0.040503
C 0.969849 0.551944 0.056654
C 0.781591 1.456884 -1.180308
H 0.105280 2.254133 -0.843894
C 0.988525 1.344912 1.368785
H 1.382386 0.706978 2.163502
C 0.157391 -1.468564 -1.274199
H 1.003795 -1.046910 -1.825651
H -0.061586 1.545653 1.627599
C -0.012384 -1.523937 1.304524
H 1.016168 -1.872055 1.469011
H -0.592213 -2.417845 1.056678
I 3.062165 -0.372775 -0.003926
H 0.227671 0.904113 -1.947165
H -0.701894 -1.349276 -1.947395
C 1.755534 2.661518 1.372889
H 1.688196 3.115778 2.368745
H 2.818217 2.508159 1.146107
H 1.347256 3.381069 0.652138
C 1.982597 2.112374 -1.855965
H 2.576458 1.374538 -2.406841
H 1.623877 2.861783 -2.573527
H 2.645618 2.613224 -1.143690
C -0.575234 -0.983378 2.631593
H -1.409297 -0.283639 2.494314
H -0.953318 -1.816687 3.236413
H 0.177347 -0.467546 3.233968
C 0.422352 -2.964928 -1.123521
H -0.444684 -3.509861 -0.730261
H 0.653377 -3.387665 -2.109462
H 1.279941 -3.161628 -0.468078
54
B'_Et_conf_25 Eopt -1028.040311
C 3.538505 0.578605 -1.552342
C 2.404930 -0.436882 -1.240859
C 2.560872 0.169494 1.280821
C 3.630102 1.236466 0.902198
H 3.104194 1.520614 -1.914092
H 4.307594 1.376759 1.757394
C 3.014313 -1.836380 -0.914479
H 4.088518 -1.831753 -1.152199
C 3.213243 -1.213649 1.562080
H 2.910937 -1.557189 2.560286
H 1.794745 -0.520497 -2.146599
H 2.068841 0.520987 2.193482
C 2.825895 -2.284572 0.538819
H 1.765786 -2.541094 0.697179
H 3.393931 -3.207163 0.721133
C 4.432040 0.856687 -0.343374
H 5.143195 1.658688 -0.584240
H 5.040078 -0.033863 -0.127245
H 2.562817 -2.588350 -1.576180
H 4.308427 -1.115935 1.595168
H 4.144329 0.181530 -2.380491
H 3.138599 2.208652 0.733978
B 1.608333 0.124571 0.015510
C 0.079184 0.714507 -0.006602
C -0.854643 -0.540726 0.054387
C -0.558262 -1.543148 -1.077672
H 0.424748 -1.968950 -0.835248
C -0.762701 -1.266757 1.418575
H 0.274872 -1.125731 1.758059
C -0.077878 1.528968 -1.314058
H -1.137895 1.759667 -1.480294
H -0.871646 -2.343023 1.243470
C -0.054033 1.660704 1.216999
H 0.833379 2.307865 1.202840
H 0.048159 1.079374 2.139665
I -3.012755 0.048312 -0.298049
H -0.437912 -1.008717 -2.025802
H 0.235028 0.921105 -2.170538
C -1.694940 -0.891756 2.568254
H -1.321142 -1.344540 3.495752
H -1.767764 0.189259 2.723813
H -2.706493 -1.273148 2.392688
C -1.517367 -2.711985 -1.288949
H -1.829030 -3.177976 -0.346037
H -2.421199 -2.403371 -1.823694
H -1.018482 -3.481615 -1.891382
C -1.282200 2.568925 1.339370
H -1.587349 2.998954 0.377712
H -2.151823 2.046479 1.748672
H -1.048017 3.400716 2.017351
C 0.705955 2.844156 -1.334646

H 1.738380 2.721241 -0.984188
H 0.748250 3.245877 -2.354369
H 0.240215 3.605812 -0.698100
54
B'_Et_conf_27 Eopt -1028.036199
C 3.051509 -1.037960 1.698060
C 2.558326 0.335311 1.171093
C 2.259148 -0.669641 -1.183048
C 2.541796 -2.078103 -0.582502
H 2.239521 -1.478733 2.296102
H 2.967154 -2.720340 -1.367845
C 3.716107 1.212767 0.631837
H 4.416044 1.425699 1.453981
C 3.572498 0.019436 -1.642919
H 3.303762 0.838671 -2.321152
H 2.132713 0.859855 2.033959
H 1.642930 -0.802963 -2.081842
C 4.481205 0.595115 -0.542168
H 5.125083 1.365191 -0.988987
H 5.165474 -0.171238 -0.164660
C 3.471136 -2.062617 0.633246
H 3.473632 -3.062899 1.088120
H 4.500984 -1.886355 0.308437
H 3.314708 2.188289 0.318538
H 4.147465 -0.695526 -2.252153
H 3.883541 -0.872475 2.400220
H 1.599185 -2.556391 -0.276418
B 1.531055 0.138100 -0.027006
C -0.020141 0.686099 -0.010455
C -0.989335 -0.545135 -0.026747
C -0.876702 -1.426169 -1.280802
H 0.087817 -1.932677 -1.186105
C -0.819735 -1.427923 1.236005
H 0.228577 -1.302734 1.546226
C -0.280135 1.622436 -1.227293
H -1.015750 2.372403 -0.908066
H -0.911321 -2.477751 0.934995
C -0.241382 1.500340 1.291677
H 0.064462 0.898236 2.155891
H -1.316125 1.685865 1.412093
I -3.121610 0.244681 -0.113577
H -0.811157 -0.809756 -2.182829
H -0.770856 1.072040 -2.035209
C -1.702969 -1.225834 2.464380
H -2.722670 -1.577923 2.273510
H -1.294208 -1.810859 3.298532
H -1.760946 -0.178323 2.777088
C -1.938321 -2.503358 -1.496959
H -2.862961 -2.083833 -1.906074
H -1.560856 -3.245237 -2.212003
H -2.193521 -3.033714 -0.570616
C 0.459640 2.856037 1.367848
H 1.544663 2.784853 1.236111
H 0.076065 3.553882 0.613090
H 0.276586 3.307721 2.351070
C 0.936824 2.334998 -1.822275
H 1.479428 1.677297 -2.509826
H 0.619790 3.215180 -2.395958
H 1.644030 2.681140 -1.057461
54
B'_Et_conf_3 Eopt -1028.046112
C -3.735304 1.003077 0.976673
C -2.488074 0.098992 1.246187
C -2.380771 -0.443751 -1.293483
C -3.617497 0.482154 -1.526183
H -3.734242 1.842320 1.684401
H -4.529673 -0.130427 -1.588660
C -2.937592 -1.363362 1.508803
H -3.606467 -1.374416 2.382422
C -2.826278 -1.893828 -0.978095
H -1.939998 -2.543924 -0.899760
H -1.984599 0.464389 2.148777
H -1.810002 -0.472815 -2.226387
C -3.645381 -1.994021 0.308845
H -3.869900 -3.047433 0.524813
H -4.617167 -1.502209 0.157201
C -3.798499 1.556998 -0.449915
H -3.000420 2.308053 -0.571312
H -4.747150 2.088926 -0.605150
H -2.068470 -1.980689 1.779328
H -3.415369 -2.278268 -1.824133
H -4.652674 0.433458 1.184752
H -3.513630 0.976983 -2.500563
B -1.561844 0.108931 -0.045542
C -0.042786 0.702550 -0.133963
C 0.921876 -0.528865 -0.196423
C 0.628843 -1.650136 0.812346
H 1.390394 -2.423265 0.669397
C 1.000000 -1.128611 -1.610890
H 1.442554 -0.388773 -2.285767
C 0.241464 1.633800 1.069842
H 1.276728 1.985976 1.009182
H -0.039286 -1.265215 -1.939884
C -0.054912 1.558090 -1.434204
H -0.920853 2.232670 -1.365488
H -0.267743 0.922613 -2.298920
I 3.076927 0.049548 0.264567
H -0.316491 -2.111284 0.475487
H 0.176964 1.069561 2.003449
C 1.722631 -2.460961 -1.782589
H 1.173076 -3.290529 -1.321989
H 1.814402 -2.683624 -2.852325
H 2.733746 -2.436703 -1.357417
C 0.544526 -1.361654 2.305941
H 1.442827 -0.841193 2.660463
H -0.332422 -0.770181 2.581875
H 0.478551 -2.315707 2.843710
C 1.179290 2.405364 -1.740976
H 0.967690 3.062605 -2.594702
H 1.469337 3.041950 -0.895580
H 2.045935 1.788365 -2.003784
C -0.656267 2.865467 1.179548
H -1.721516 2.610678 1.210533
H -0.423662 3.406272 2.105592
H -0.499994 3.563310 0.347316
54
B'_Et_conf_4 Eopt -1028.041236
C 3.590005 -1.255693 0.920638
C 2.555060 -0.149154 1.277351
C 2.404965 0.463132 -1.239813
C 3.458852 -0.644679 -1.539016
H 3.076755 -2.222236 0.804451
H 4.057676 -0.323206 -2.403903
C 3.272502 1.203337 1.550021
H 4.360290 1.044997 1.601008
C 3.103156 1.820002 -0.937197
H 2.683088 2.594264 -1.593734
H 2.056065 -0.478288 2.192151
H 1.816364 0.560346 -2.157068
C 2.963085 2.288464 0.515078
H 1.929827 2.628384 0.674190
H 3.603479 3.164684 0.687456
C 4.374270 -0.938641 -0.351833
H 5.042315 -1.775815 -0.596607
H 5.025571 -0.070811 -0.171697
H 2.975911 1.573275 2.540688
H 4.170594 1.751466 -1.197120
H 4.279656 -1.374675 1.769281
H 2.944801 -1.567458 -1.845336
B 1.595656 -0.081066 0.014247
C 0.093395 -0.718253 -0.035580
C -0.927206 0.441871 -0.269550
C -0.659737 1.621973 0.691002
H -0.042161 2.314430 0.097818
C -0.977857 0.939061 -1.722972
H -1.292308 0.124967 -2.383182
C -0.185182 -1.542168 1.252907
H 0.698523 -2.170380 1.431856
H 0.058229 1.181384 -1.991163
C 0.057641 -1.706909 -1.236571

H	0.484774	-1.218659	-2.121574
H	-0.995843	-1.914641	-1.478315
I	-3.029256	-0.387039	0.027030
H	-0.019047	1.283254	1.510216
H	-0.999867	-2.243432	1.022922
C	-1.828617	2.171870	-2.008893
H	-1.773509	2.402459	-3.079686
H	-2.883247	2.010557	-1.755300
H	-1.471166	3.055042	-1.465100
C	-1.816054	2.412290	1.292535
H	-1.419085	3.304194	1.794259
H	-2.539326	2.736684	0.537648
H	-2.354183	1.816018	2.038802
C	0.761986	-3.048243	-1.028532
H	0.823812	-3.586354	-1.982676
H	1.784353	-2.932195	-0.649333
H	0.218663	-3.689171	-0.324695
C	-0.541213	-0.853625	2.571822
H	-1.461609	-0.263496	2.499771
H	-0.711530	-1.627740	3.331533
H	0.250116	-0.197266	2.950239

54

B'_Et_conf_5 Eopt -1028.041302

C	3.683697	-0.635952	1.250547
C	2.352373	0.183280	1.308575
C	2.373704	0.378744	-1.281351
C	3.723750	-0.402406	-1.328095
H	3.654038	-1.424653	2.013381
H	4.553496	0.308491	-1.454051
C	2.699887	1.692075	1.416236
H	3.343393	1.836071	2.297043
C	2.634670	1.901171	-1.108525
H	1.677811	2.447857	-1.097638
H	1.808178	-0.089796	2.219972
H	1.859636	0.239390	-2.239330
C	3.403352	2.227224	0.170797
H	3.536360	3.314317	0.256895
H	4.415494	1.801314	0.108984
C	3.984958	-1.290438	-0.104273
H	3.364327	-2.193091	-0.197630
H	5.025715	-1.642060	-0.118527
H	1.791523	2.279628	1.602046
H	3.190397	2.266526	-1.984831
H	4.518702	0.023632	1.528878
H	3.736296	-1.039606	-2.221983
B	1.523251	-0.085885	-0.025104
C	0.033389	-0.753029	-0.116316
C	-0.980352	0.445086	-0.151256
C	-0.690406	1.440385	0.985604
H	0.045134	2.133158	0.546028
C	-1.021265	1.138357	-1.523051
H	-1.443916	0.452385	-2.263350
C	-0.248536	-1.670788	1.106597
H	-1.078552	-2.328604	0.819692
H	0.026567	1.295355	-1.817140
C	-0.104268	-1.579171	-1.419071
H	0.152854	-0.954224	-2.279918
H	-1.164492	-1.849451	-1.537503
I	-3.089301	-0.354089	0.097004
H	-0.168620	0.918088	1.793883
H	-0.637761	-1.067056	1.935918
C	-1.746773	2.474571	-1.616510
H	-1.703346	2.832927	-2.652022
H	-2.804077	2.383512	-1.338094
H	-1.283856	3.241120	-0.982323
C	-1.808523	2.262957	1.619361
H	-2.428967	1.637784	2.271083
H	-1.362285	3.052675	2.238188
H	-2.465368	2.735878	0.884012
C	0.726242	-2.855123	-1.513985
H	0.563866	-3.324350	-2.492437
H	1.801025	-2.650242	-1.424460
H	0.451471	-3.589974	-0.747775
C	0.906099	-2.542481	1.634943
H	0.549869	-3.554323	1.863864
H	1.723825	-2.648316	0.915778
H	1.335943	-2.134984	2.556315

54

B'_Et_conf_6 Eopt -1028.041140

C	-2.600274	-1.881683	-1.035076
C	-2.344654	-0.370237	-1.261937
C	-2.356465	-0.011062	1.294058
C	-2.808811	-1.477636	1.505901
H	-1.634821	-2.411208	-1.044146
H	-3.576389	-1.501589	2.295224
C	-3.656189	0.450001	-1.382747
H	-4.300481	-0.016484	-2.144146
C	-3.560107	0.957642	1.124146
H	-3.186876	1.986265	1.019458
H	-1.829330	-0.268959	-2.225819
H	-1.814766	0.283033	2.201134
C	-4.450044	0.644710	-0.082103
H	-5.157092	1.474027	-0.223740
H	-5.067540	-0.233817	0.129816
C	-3.337710	-2.220076	0.265901
H	-3.258906	-3.301540	0.444265
H	-4.406893	-2.026387	0.134835
H	-3.393742	1.443002	-1.776138
H	-4.162398	0.944121	2.045089
H	-3.168664	-2.281191	-1.888642
H	-1.951407	-2.034889	1.908017
B	-1.501799	0.163670	-0.028060
C	-0.005271	0.811591	-0.136641
C	0.959458	-0.432679	-0.143558
C	0.623668	-1.379863	1.023578
H	-0.159644	-2.041286	0.616443
C	0.976893	-1.159033	-1.498742
H	1.438850	-0.509913	-2.248975
C	0.356071	1.749599	1.052576
H	1.208345	2.353466	0.713512
H	-0.071445	-1.272089	-1.802928
C	0.138713	1.587915	-1.467022
H	-0.166037	0.946807	-2.299851
H	1.205410	1.811358	-1.623099
I	3.105908	0.271672	0.080779
H	0.144512	-0.804173	1.821896
H	0.746745	1.150832	1.885955
C	1.634581	-2.531727	-1.555849
H	1.578824	-2.911163	-2.583263
H	2.693323	-2.489889	-1.271535
H	1.127491	-3.257871	-0.907863
C	1.699355	-2.248019	1.670181
H	1.214975	-2.992357	2.315897
H	2.319819	-2.779634	0.943621
H	2.361389	-1.640950	2.297513
C	-0.646885	2.892781	-1.570061
H	-0.241093	3.670248	-0.912052
H	-0.598824	3.274697	-2.597454
H	-1.707491	2.753875	-1.316333
C	-0.694254	2.709451	1.627116
H	-1.341021	3.154450	0.866299
H	-1.337925	2.228422	2.369991
H	-0.179484	3.533780	2.137746

54

B'_Et_conf_9 Eopt -1028.041097

C	3.810373	1.039399	-0.865385
C	2.580147	0.157285	-1.263164
C	2.329704	-0.564525	1.225948
C	3.551853	0.340022	1.592037
H	3.843459	1.930132	-1.506105
H	4.456892	-0.281077	1.669789
C	3.059942	-1.275958	-1.607656
H	3.792542	-1.213407	-2.426221
C	2.789997	-1.987375	0.822968
H	1.907230	-2.620391	0.634438
H	2.118124	0.578820	-2.163979
H	1.697524	-0.659696	2.114483
C	3.688687	-1.995524	-0.413802
H	3.930550	-3.030841	-0.690140
H	4.647342	-1.516754	-0.166020

C 3.803285 1.484202 0.601208
H 3.011711 2.239881 0.732799
H 4.748677 1.988040 0.844583
H 2.221078 -1.871906 -1.996563
H 3.322141 -2.446297 1.669751
H 4.737010 0.484204 -1.071041
H 3.390960 0.772885 2.587907
B 1.596663 0.108284 -0.011612
C 0.104952 0.763406 0.064079
C -0.883565 -0.458818 0.118803
C -0.538471 -1.436554 -1.019228
H 0.527461 -1.678556 -0.885334
C -0.895598 -1.169223 1.486554
H -0.681942 -0.443024 2.274264
C -0.140749 1.651105 -1.180941
H -1.179032 1.998276 -1.185561
H -0.031107 -1.849812 1.456013
C 0.129443 1.654843 1.334464
H 0.919438 2.401783 1.181649
H 0.465769 1.077119 2.202281
I -3.011983 0.177926 -0.345305
H -0.615047 -0.919373 -1.981619
H -0.022844 1.046257 -2.088234
C -2.118441 -1.967089 1.940324
H -2.925991 -1.298282 2.257214
H -2.518215 -2.630307 1.168743
H -1.835411 -2.581834 2.804773
C -1.269167 -2.770546 -1.098110
H -2.355298 -2.642012 -1.176233
H -0.930498 -3.309388 -1.991266
H -1.051436 -3.403758 -0.229634
C -1.154432 2.394276 1.703682
H -0.948190 3.113091 2.507440
H -1.569360 2.950768 0.853248
H -1.930581 1.707274 2.061275
C 0.749919 2.888261 -1.292993
H 0.638184 3.337182 -2.287914
H 0.470314 3.653489 -0.557603
H 1.813068 2.662898 -1.150696
42
B'_Me_conf_1 Eopt -870.990160
C -3.511157 1.221475 1.109021
C -2.556891 0.008346 1.307545
C -2.134883 -0.039318 -1.258253
C -3.103507 1.170747 -1.408917
H -3.446003 1.880302 1.984950
H -4.111375 0.817915 -1.675551
C -3.303779 -1.344865 1.184161
H -4.082039 -1.400629 1.960052
C -2.889878 -1.396401 -1.312566
H -2.162839 -2.222883 -1.242767
H -2.135786 0.051664 2.318478
H -1.442343 -0.031379 -2.104225
C -3.921846 -1.552525 -0.197201
H -4.380158 -2.549212 -0.254359
H -4.738048 -0.830846 -0.347695
C -3.183974 2.034479 -0.147158
H -2.206063 2.526224 0.000381
H -3.918640 2.839884 -0.283270
H -2.599697 -2.168377 1.388660
H -3.375702 -1.492762 -2.294837
H -4.556275 0.878863 1.058616
H -2.767166 1.796886 -2.245955
B -1.446962 0.003032 0.170973
C 0.107400 -0.010751 0.600600
C 1.059149 -0.007702 -0.608277
C 0.980453 -1.270882 -1.456307
H 1.270121 -2.163129 -0.892988
H -0.047573 -1.415299 -1.812346
C 0.970085 1.265221 -1.442017
H 1.597559 1.187096 -2.336614
H 1.284155 2.148386 -0.877585
C 0.290119 -1.258268 1.486448
H 1.323962 -1.324442 1.851467
H 0.059507 -2.190436 0.955116
H -0.068259 1.424845 -1.763569
C 0.283005 1.253443 1.468590
H 1.332230 1.371339 1.769929
H -0.022410 2.169771 0.946486
H -0.317921 1.175596 2.381001
I 3.225886 -0.010191 0.088244
H 1.634900 -1.189357 -2.331006
H -0.367060 -1.203030 2.361795
42
B'_Me_conf_2 Eopt -870.993058
C 3.069939 1.331330 -1.279991
C 2.273436 -0.002991 -1.302718
C 2.273301 -0.003537 1.302797
C 3.069787 1.330821 1.280753
H 2.770641 1.944569 -2.140643
H 4.144570 1.132289 1.410486
C 3.226681 -1.235362 -1.268588
H 3.871153 -1.211193 -2.159768
C 3.226583 -1.235846 1.268487
H 2.634559 -2.162771 1.336294
H 1.695953 -0.066982 -2.232534
H 1.695550 -0.067827 2.232430
C 4.079245 -1.284579 -0.000028
H 4.689036 -2.198294 -0.000196
H 4.787823 -0.443802 0.000163
C 2.855683 2.144194 0.000530
H 1.820444 2.523483 0.000550
H 3.508385 3.028171 0.000731
H 2.634586 -2.162215 -1.336772
H 3.870984 -1.211928 2.159723
H 4.144740 1.132840 -1.409659
H 2.770357 1.943702 2.141614
B 1.403600 -0.200655 -0.000087
C -0.121480 -0.747146 -0.000259
C -0.997058 0.530573 -0.000479
C -0.830514 1.392309 1.247083
H -1.488058 2.266492 1.196209
H -1.048905 0.848713 2.170591
C -0.831224 1.391465 -1.248703
H -1.488525 2.265837 -1.197873
H -1.050465 0.847355 -2.171705
C -0.358973 -1.625123 1.237432
H 0.338091 -2.473628 1.231654
H -1.376358 -2.039212 1.225929
H 0.205771 1.756417 -1.302007
C -0.358644 -1.625263 -1.237912
H 0.337546 -2.474477 -1.231168
H -1.376473 -2.038305 -1.227296
H -0.217539 -1.091772 -2.183175
I -3.176216 -0.035389 0.000271
H 0.206429 1.757559 1.299397
H -0.219387 -1.091193 2.182674
42
B'_Me_conf_3 Eopt -870.992872
C 2.963182 1.114787 -1.553744
C 2.218984 -0.215793 -1.311980
C 2.239313 0.240249 1.241708
C 2.861071 1.626451 0.970400
H 2.223690 1.839477 -1.927761
H 3.501145 1.917842 1.817405
C 3.161241 -1.432087 -1.079929
H 3.788807 -1.578983 -1.971862
C 3.276161 -0.903059 1.429520
H 2.731146 -1.783961 1.804860
H 1.626546 -0.435715 -2.210461
H 1.675812 0.302760 2.182031
C 4.047271 -1.320195 0.167003
H 4.519842 -2.294983 0.351841
H 4.871434 -0.625901 -0.025591
C 3.665471 1.729851 -0.331531
H 3.865468 2.790349 -0.539423
H 4.649758 1.271656 -0.189048
H 2.544404 -2.341452 -0.988878
H 3.985094 -0.622332 2.223628
H 3.694989 0.985949 -2.366621

H 2.043252 2.365288 0.940727
B 1.360255 -0.195138 0.007646
C -0.157738 -0.742047 0.103810
C -1.033996 0.533117 0.176973
C -1.006584 1.243907 1.524064
H -1.650614 2.129545 1.495872
H -1.336456 0.601909 2.346198
C -0.713710 1.529346 -0.931805
H 0.283937 1.958485 -0.734629
H -1.427886 2.359162 -0.934308
C -0.304776 -1.624019 1.351994
H 0.262835 -2.554765 1.216994
H -1.358281 -1.902321 1.505222
H -0.702005 1.071658 -1.926484
C -0.476695 -1.609693 -1.123488
H -1.402398 -2.176810 -0.962946
H -0.595443 -1.022909 -2.041339
H 0.328601 -2.335905 -1.294819
I -3.196935 -0.007708 -0.105716
H 0.018350 1.582210 1.730691
H 0.059068 -1.140921 2.265423
54
C_Et_conf_1 Eopt -1028.041773
C -0.168677 -2.153723 -0.773733
C 0.331847 -1.434522 0.517188
C 2.617797 -1.052201 -0.690461
C 2.116361 -1.862243 -1.913716
H -0.734156 -1.425941 -1.374425
H 2.957057 -2.442734 -2.319832
C 1.016066 -2.380451 1.535933
H 0.330839 -3.211244 1.755725
C 3.298174 -1.922348 0.408231
H 3.730219 -1.243708 1.158768
H -0.547090 -0.992961 0.997256
H 3.365225 -0.337020 -1.050051
C 2.384356 -2.937050 1.108729
H 2.902958 -3.309289 2.002641
H 2.247202 -3.811329 0.465222
C 0.922088 -2.787791 -1.642166
H 0.476267 -3.071317 -2.605159
H 1.264477 -3.723676 -1.189951
H 1.131616 -1.825374 2.477892
H 4.146172 -2.448734 -0.052990
H -0.897987 -2.916108 -0.465394
H 1.834501 -1.141335 -2.695070
B 1.387019 -0.417836 0.011191
C 1.239456 1.302584 0.675742
C 1.082568 1.491604 -0.697282
C 2.204531 1.938156 -1.583749
H 2.174407 1.401686 -2.539492
C -0.290862 1.446041 -1.318492
H -0.732789 2.438442 -1.121142
C 2.606870 1.517348 1.334476
H 2.770626 2.605695 1.359709
H -0.935041 0.752767 -0.765546
C 0.050883 1.420830 1.611391
H -0.878513 1.114702 1.127132
H 0.188742 0.759442 2.474237
H 3.180612 1.768568 -1.128724
H 3.407602 1.113310 0.706748
C 2.026106 3.448653 -1.819439
H 2.055455 3.995846 -0.868389
H 2.843104 3.812434 -2.452039
H 1.076005 3.673264 -2.318055
C -0.361134 1.126361 -2.807108
H 0.123984 0.169137 -3.036009
H -1.414121 1.043192 -3.098080
H 0.101383 1.903658 -3.426306
C -0.082929 2.875676 2.070826
H -0.226358 3.542851 1.210064
H -0.954965 2.975622 2.726796
H 0.802352 3.213290 2.623905
C 2.738231 0.939251 2.740817
H 2.471611 -0.125485 2.763788
H 3.777028 1.031958 3.076844
H 2.103768 1.463532 3.464444
I -3.650334 -0.198510 0.179555
54
C_Et_conf_10 Eopt -1028.039753
C 0.202908 2.655120 0.275474
C 0.380417 1.427466 -0.675816
C 2.824203 1.149779 0.234717
C 2.608781 2.405747 1.136482
H -0.835849 2.662273 0.628208
H 3.018815 3.290224 0.630172
C 0.973123 1.849740 -2.052890
H 0.270748 2.547105 -2.528535
C 3.333534 1.570706 -1.183828
H 3.527230 0.675466 -1.792937
H -0.599299 0.976883 -0.857054
H 3.581310 0.496875 0.677774
C 2.358218 2.479468 -1.927511
H 2.755063 2.696322 -2.927968
H 2.281103 3.446632 -1.411871
C 1.138098 2.648461 1.490664
H 0.811754 1.851242 2.179369
H 1.036359 3.591887 2.042381
H 1.037854 0.967956 -2.709822
H 4.300935 2.074041 -1.047648
H 0.347504 3.581573 -0.298036
H 3.186563 2.285223 2.061132
B 1.439630 0.489665 -0.041701
C 1.058865 -1.488293 -0.600773
C 1.120586 -1.168983 0.755099
C 2.309965 -1.601504 1.601520
H 3.228188 -1.581177 1.007900
C -0.147300 -0.853217 1.541591
H -0.872194 -0.286570 0.951652
C 2.281739 -1.967272 -1.347044
H 3.160606 -1.382122 -1.049313
H 0.108679 -0.230700 2.405163
C -0.225986 -1.496074 -1.379688
H -1.057928 -1.107330 -0.791134
H -0.109736 -0.855108 -2.264214
H 2.128420 -2.660617 1.835549
H 2.122094 -1.800339 -2.419404
C 2.504294 -0.838939 2.908604
H 3.461228 -1.125848 3.358854
H 2.514422 0.247312 2.758635
H 1.715236 -1.072458 3.632911
C -0.801296 -2.153262 2.019963
H -1.716077 -1.918541 2.575409
H -1.071912 -2.784192 1.163610
H -0.131333 -2.721941 2.676994
C -0.582790 -2.912202 -1.857210
H 0.116898 -3.277643 -2.617064
H -0.606721 -3.624645 -1.023559
H -1.582718 -2.876207 -2.302960
C 2.591865 -3.452161 -1.097002
H 1.762624 -4.097637 -1.400145
H 3.474783 -3.730470 -1.683738
H 2.810061 -3.646891 -0.042179
I -3.678268 0.328559 -0.004976
54
C_Et_conf_11 Eopt -1028.040070
C -0.559296 -2.702082 0.699525
C -0.390135 -1.510734 -0.290947
C -2.943060 -0.976681 -0.010873
C -3.055009 -2.183721 0.972223
H 0.368000 -2.801244 1.277138
H -3.419877 -3.064915 0.426766
C -0.680131 -1.936744 -1.766100
H 0.054743 -2.703558 -2.045520
C -3.151640 -1.444901 -1.490998
H -3.135454 -0.575220 -2.162749
H 0.642687 -1.154799 -0.245032
H -3.713754 -0.232815 0.213961
C -2.104450 -2.455758 -1.953642
H -2.272384 -2.691627 -3.012703
H -2.230077 -3.399562 -1.405491

C -1.732350 -2.522474 1.667842
H -1.481990 -1.700042 2.359522
H -1.853131 -3.422441 2.284615
H -0.511971 -1.081289 -2.439613
H -4.159913 -1.876622 -1.558771
H -0.678766 -3.639209 0.137833
H -3.813368 -1.956220 1.731700
B -1.480494 -0.452818 0.000212
C -0.972437 1.474195 -0.750654
C -1.123229 1.264044 0.620845
C -2.347890 1.795089 1.360906
H -3.215584 1.853180 0.700313
C 0.089298 1.010489 1.510084
H 0.945741 0.688422 0.913378
C -2.128189 1.899113 -1.614552
H -3.088966 1.590833 -1.194333
H -0.129163 0.185670 2.204406
C 0.360521 1.434039 -1.451426
H 0.918741 0.523328 -1.207560
H 0.178257 1.424054 -2.531112
H -2.091080 2.835658 1.609588
H -2.021321 1.430122 -2.600517
C -2.727179 1.057978 2.641512
H -3.028819 0.023480 2.444487
H -1.909429 1.040604 3.370889
H -3.578041 1.565416 3.110506
C 0.461101 2.255252 2.325427
H 1.436624 2.092904 2.798001
H 0.531873 3.144137 1.686502
H -0.270097 2.462447 3.114610
C 1.253869 2.632402 -1.088834
H 0.712832 3.584150 -1.145809
H 1.679308 2.524991 -0.086396
H 2.088816 2.666106 -1.797713
C -2.109652 3.427572 -1.779856
H -2.122256 3.929342 -0.804011
H -1.221753 3.755119 -2.331865
H -2.996329 3.738442 -2.342845
I 3.659387 -0.445231 0.017432
54
C_Et_conf_12 Eopt -1028.039958
C 0.664601 2.464626 1.570561
C 0.186235 1.586395 0.371692
C 2.674238 1.415435 -0.450367
C 3.097635 2.289101 0.777538
H 0.020849 2.257963 2.434214
H 3.204233 3.334811 0.457327
C -0.114915 2.453431 -0.890533
H -0.928139 3.144885 -0.631567
C 2.289959 2.295408 -1.677797
H 2.041598 1.642547 -2.529295
H -0.739164 1.069721 0.637111
H 3.507762 0.771741 -0.742910
C 1.105976 3.219147 -1.395489
H 0.842581 3.763191 -2.311952
H 1.396870 3.982296 -0.659692
C 2.124711 2.210165 1.960345
H 2.195309 1.202611 2.406622
H 2.433823 2.914183 2.743641
H -0.500172 1.805905 -1.692957
H 3.172963 2.878826 -1.972649
H 0.526154 3.526295 1.323714
H 4.089762 1.960260 1.110997
B 1.368354 0.681292 -0.051294
C 1.192323 -1.158432 -0.730350
C 1.279934 -1.196911 0.655238
C 2.602951 -1.409071 1.367787
H 2.462892 -1.148363 2.423178
C 0.066033 -1.215153 1.551371
H -0.847559 -1.004008 0.991625
C 2.389819 -1.396686 -1.625187
H 3.321992 -1.355541 -1.057218
H 0.181989 -0.419254 2.302530
C -0.148628 -1.136593 -1.445354
H -0.815405 -0.372823 -1.027556
H 0.031177 -0.870691 -2.494255
H 3.374506 -0.736540 0.971595
H 2.428793 -0.597030 -2.376994
C 3.102400 -2.856576 1.259933
H 3.375238 -3.114031 0.231182
H 3.993031 -2.976700 1.887620
H 2.345129 -3.569960 1.600976
C -0.079101 -2.554690 2.283815
H 0.736083 -2.719150 2.997887
H -1.022071 -2.545219 2.841324
H -0.104498 -3.396920 1.581361
C -0.883531 -2.481375 -1.366026
H -1.197720 -2.701765 -0.339841
H -1.787509 -2.427405 -1.983568
H -0.264181 -3.309817 -1.725498
C 2.294569 -2.739843 -2.358402
H 2.158097 -3.573712 -1.658529
H 1.465108 -2.749344 -3.074582
H 3.223015 -2.905617 -2.916277
I -3.664822 0.115131 0.025590
54
C_Et_conf_2 Eopt -1028.035496
C -0.197342 -1.434173 -1.564052
C 0.270745 -1.512400 -0.069871
C 2.655286 -0.793186 -0.876284
C 2.103070 -0.733832 -2.338197
H -0.645748 -0.451728 -1.769242
H 2.931857 -0.966755 -3.021211
C 0.826225 -2.928843 0.255634
H 0.589544 -3.615200 -0.569567
C 3.170423 -2.224686 -0.534503
H 4.205893 -2.152946 -0.178302
H -0.607777 -1.303209 0.546651
H 3.479674 -0.075118 -0.804267
C 2.332981 -2.944391 0.529430
H 2.506415 -2.451650 1.499042
H 2.683458 -3.979057 0.640131
C 0.931952 -1.688610 -2.558165
H 0.550712 -1.569387 -3.580921
H 1.277001 -2.728394 -2.477460
H 0.302909 -3.319024 1.137234
H 3.205094 -2.831268 -1.450113
H -1.004187 -2.169105 -1.687815
H 1.776081 0.288501 -2.580828
B 1.411913 -0.453799 -0.002860
C 1.258086 0.999250 0.986691
C 0.982167 1.755456 -0.180333
C 2.045365 2.448847 -0.957663
H 1.799296 2.458408 -2.023186
C -0.427306 1.994605 -0.601400
H -0.784298 2.759801 0.117612
C 2.613741 1.239300 1.699790
H 2.342707 1.859424 2.566664
H -1.050367 1.125093 -0.359255
C 0.086684 0.813525 1.967793
H -0.083897 1.803477 2.418367
H -0.833145 0.560967 1.436890
H 3.023349 1.980754 -0.831477
H 3.266376 1.860136 1.082315
C 2.090018 3.903528 -0.438924
H 1.140420 4.418049 -0.626672
H 2.295062 3.935895 0.638018
H 2.887681 4.440180 -0.963248
C -0.683147 2.442448 -2.032087
H -0.239647 3.420347 -2.252272
H -0.290560 1.712717 -2.752760
H -1.764716 2.516165 -2.188154
C 0.301724 -0.210040 3.075859
H 1.076273 0.104026 3.785108
H -0.632534 -0.329961 3.636109
H 0.575668 -1.195270 2.673547
C 3.432281 0.047071 2.196776
H 2.841442 -0.665455 2.778748
H 3.912594 -0.493322 1.374443
H 4.231206 0.423305 2.847227

I -3.675116 -0.191408 0.180943
54
C_Et_conf_3 Eopt -1028.041492
C -1.967831 1.664264 2.119954
C -2.527008 1.267184 0.720420
C -0.118326 1.546330 -0.279057
C 0.369036 1.903082 1.165032
H -1.729862 0.754199 2.690186
H 1.227716 2.579809 1.056386
C -2.969489 2.523989 -0.095501
H -2.955709 3.406755 0.559597
C -0.605134 2.813718 -1.041753
H -0.054636 2.892167 -1.987306
H -3.392170 0.618000 0.883982
H 0.730895 1.094776 -0.798457
C -2.108381 2.793750 -1.334803
H -2.304067 2.000965 -2.077233
H -2.414791 3.738445 -1.802641
C -0.718857 2.539267 2.026927
H -0.324675 2.721015 3.035438
H -0.987676 3.524319 1.620634
H -4.011103 2.386832 -0.411409
H -0.352220 3.712378 -0.462382
H -2.763449 2.183578 2.671995
H 0.754505 0.999482 1.659788
B -1.329063 0.606608 -0.019726
C -1.339602 -1.029597 -0.802699
C -1.223376 -1.475935 0.520224
C 0.134355 -1.726374 1.112908
H 0.875588 -1.060762 0.655707
C -2.397461 -1.860367 1.364339
H -3.340972 -1.510516 0.945061
C -0.151727 -1.176833 -1.753296
H -0.106126 -2.246557 -2.008633
H -2.291679 -1.437058 2.370342
C -2.697163 -0.981181 -1.509794
H -3.507418 -0.749861 -0.816289
H -2.680921 -0.170529 -2.248960
H 0.417706 -2.731810 0.749970
H 0.788457 -0.957111 -1.242645
C 0.256522 -1.657005 2.631276
H -0.085638 -0.687652 3.015562
H -0.308746 -2.445225 3.140419
H 1.312038 -1.767755 2.903005
C -2.437532 -3.398479 1.463549
H -1.545064 -3.800777 1.954968
H -3.315727 -3.689324 2.050224
H -2.518218 -3.854859 0.470613
C -2.994775 -2.304775 -2.220516
H -2.233354 -2.542330 -2.972369
H -3.036553 -3.130721 -1.501014
H -3.964454 -2.239318 -2.727245
C -0.233527 -0.349648 -3.032567
H -1.024863 -0.700525 -3.705834
H -0.413984 0.712237 -2.820810
H 0.718609 -0.422195 -3.569936
I 3.657841 -0.127575 -0.141697
54
C_Et_conf_4 Eopt -1028.039647
C 0.858637 2.281081 1.744546
C 0.236114 1.548785 0.523849
C 2.614163 1.365333 -0.538552
C 3.171613 2.234690 0.626728
H 1.067808 1.534142 2.527268
H 3.943448 2.903484 0.218538
C -0.182313 2.491461 -0.642062
H -0.842110 3.268898 -0.230926
C 2.110716 2.182152 -1.761061
H 1.787826 1.472352 -2.539606
H -0.671974 1.038645 0.858384
H 3.428796 0.718254 -0.877307
C 0.954890 3.137516 -1.447072
H 0.540419 3.506309 -2.394763
H 1.339923 4.022227 -0.930766
C 2.145108 3.049675 1.427509
H 2.611918 3.358704 2.372441
H 1.898384 3.978028 0.903933
H -0.805459 1.896979 -1.326234
H 2.957493 2.741099 -2.183154
H 0.105825 2.962828 2.163058
H 3.688193 1.553703 1.319599
B 1.360014 0.628145 -0.005905
C 1.204734 -1.215891 -0.704687
C 1.251042 -1.210959 0.684133
C 2.562269 -1.369973 1.437569
H 2.391006 -1.066210 2.477463
C 0.005910 -1.289851 1.535821
H -0.888359 -1.061118 0.952075
C 2.419433 -1.490717 -1.558623
H 3.339542 -1.413297 -0.975597
H 0.074486 -0.539286 2.333947
C -0.111463 -1.157728 -1.458511
H -0.763866 -0.370269 -1.056860
H 0.107686 -0.898501 -2.501389
H 3.329907 -0.699410 1.029788
H 2.467601 -0.738416 -2.356468
C 3.108853 -2.804528 1.404037
H 3.417934 -3.093798 0.394068
H 3.986398 -2.868184 2.057924
H 2.368110 -3.529684 1.754294
C -0.141776 -2.672212 2.183255
H -1.119225 -2.724023 2.675532
H -0.088786 -3.476592 1.438552
H 0.629165 -2.847731 2.942498
C -0.898217 -2.475654 -1.407055
H -1.774822 -2.386303 -2.058777
H -0.296426 -3.324834 -1.746723
H -1.259882 -2.686797 -0.395385
C 2.335099 -2.878814 -2.206560
H 2.159281 -3.664140 -1.460580
H 1.535139 -2.924690 -2.953997
H 3.282505 -3.090981 -2.714549
I -3.634280 0.131353 0.023654
54
C_Et_conf_5 Eopt -1028.041452
C 0.165696 2.471830 0.190738
C 0.520627 1.256618 -0.707617
C 2.724291 0.995574 0.664874
C 2.411776 2.310239 1.415629
H -0.463644 2.114538 1.020604
H 3.350072 2.861113 1.576060
C 1.323820 1.634015 -1.979306
H 0.801808 2.454989 -2.490982
C 3.508535 1.186415 -0.673570
H 3.746979 0.191768 -1.080733
H -0.425710 0.815109 -1.035213
H 3.349179 0.373612 1.312188
C 2.795820 2.017483 -1.746947
H 3.341722 1.900010 -2.692999
H 2.873963 3.079066 -1.493516
C 1.371829 3.226472 0.754031
H 1.017533 3.947401 1.502785
H 1.835058 3.825783 -0.036451
H 1.279979 0.774298 -2.663064
H 4.474366 1.649685 -0.425795
H -0.464915 3.155372 -0.394778
H 2.042737 2.033864 2.414710
B 1.410046 0.325284 0.166130
C 1.015484 -1.648861 -0.450487
C 1.032231 -1.269400 0.895140
C 2.117916 -1.811500 1.822485
H 3.101409 -1.723714 1.353469
C -0.251145 -0.811307 1.596823
H -0.902884 -0.231260 0.937277
C 2.243610 -2.251080 -1.082816
H 2.152262 -3.334058 -0.884831
H 0.017999 -0.154425 2.432787
C -0.231864 -1.671052 -1.278537
H -1.062837 -1.173675 -0.776026
H -0.061714 -1.161953 -2.236039

H 1.926307 -2.891682 1.909306
H 3.142831 -1.937525 -0.545485
C 2.168091 -1.194025 3.216000
H 2.247533 -0.100658 3.177896
H 1.281616 -1.447130 3.808795
H 3.045609 -1.575682 3.750116
C -1.043533 -2.015067 2.117030
H -0.438055 -2.643920 2.781197
H -1.917513 -1.658653 2.673565
H -1.398853 -2.628537 1.280351
C -0.622140 -3.137989 -1.538583
H -0.693825 -3.700151 -0.599372
H -1.604407 -3.154857 -2.022833
H 0.095767 -3.647055 -2.191165
C 2.432963 -2.010387 -2.577930
H 1.606704 -2.405858 -3.178057
H 2.537562 -0.941133 -2.799357
H 3.352700 -2.507908 -2.904964
I -3.629087 0.463188 -0.115695
54
C_Et_conf_6 Eopt -1028.039877
C 3.263323 1.617721 -1.238113
C 2.901625 1.025898 0.159652
C 0.378203 1.507557 -0.312076
C 0.766879 1.811548 -1.796014
H 3.540938 0.774325 -1.885755
H 0.004495 2.483679 -2.212405
C 2.860744 2.091156 1.288108
H 3.866627 2.520076 1.399120
C 0.446049 2.750292 0.612261
H -0.141383 2.509454 1.508333
H 3.669192 0.287343 0.412877
H -0.655164 1.145730 -0.305451
C 1.849608 3.217887 1.044756
H 1.750428 3.804002 1.968108
H 2.265804 3.908379 0.303692
C 2.160827 2.424570 -1.943661
H 2.407528 2.488537 -3.011899
H 2.140233 3.457232 -1.581585
H 2.636795 1.586823 2.240310
H -0.082256 3.579863 0.120993
H 4.167265 2.234115 -1.126103
H 0.711275 0.882435 -2.386414
B 1.455058 0.475554 0.071126
C 1.104477 -1.245157 0.698958
C 1.009558 -1.451849 -0.677770
C -0.309914 -1.492960 -1.397478
H -0.123913 -1.442273 -2.476062
C 2.213013 -1.751999 -1.527100
H 3.140324 -1.423505 -1.051999
C -0.150250 -1.059956 1.545495
H -0.493903 -2.066023 1.823643
H 2.105861 -1.207842 -2.475084
C 2.331448 -1.694949 1.482428
H 3.200544 -1.799772 0.830953
H 2.587623 -0.943553 2.239944
H -0.937280 -0.634192 -1.130470
H -0.956463 -0.621256 0.950905
C -1.097921 -2.769578 -1.063928
H -1.426874 -2.779921 -0.020021
H -1.994174 -2.798533 -1.693842
H -0.508642 -3.673503 -1.256784
C 2.294817 -3.259097 -1.818158
H 3.208387 -3.464365 -2.386337
H 2.326359 -3.835478 -0.885101
H 1.439067 -3.597045 -2.412956
C 2.054041 -3.040227 2.161571
H 1.295340 -2.952164 2.947754
H 1.706766 -3.783026 1.430988
H 2.975781 -3.414575 2.621427
C 0.065442 -0.241428 2.817348
H 0.554268 0.720814 2.614564
H -0.904611 -0.025778 3.278468
H 0.676812 -0.778530 3.552250
I -3.703399 0.298947 -0.047535
54
C_Et_conf_7 Eopt -1028.038510
C 2.246340 2.470151 -1.356619
C 2.681191 1.318720 -0.404544
C 0.306585 1.416964 0.705414
C -0.056947 2.540866 -0.311266
H 1.875455 2.049953 -2.302377
H -0.806219 3.188855 0.163557
C 3.276822 1.891801 0.928717
H 3.442835 2.970987 0.804163
C 0.939229 2.011525 2.000807
H 0.378076 1.646052 2.869544
H 3.444173 0.722398 -0.912249
H -0.622976 0.900213 0.955343
C 2.417405 1.647567 2.175822
H 2.478849 0.580448 2.444144
H 2.841218 2.197949 3.025811
C 1.154747 3.354697 -0.757468
H 0.842707 4.099661 -1.500854
H 1.557369 3.921657 0.093664
H 4.263624 1.439831 1.087596
H 0.821384 3.103560 1.994241
H 3.135725 3.066625 -1.602801
H -0.546240 2.092869 -1.189973
B 1.380188 0.550206 -0.016764
C 1.123104 -1.465331 0.582695
C 1.166497 -1.104805 -0.765593
C -0.116809 -0.817710 -1.544300
H 0.113995 -0.151609 -2.384843
C 2.357419 -1.540636 -1.614802
H 2.238854 -2.624985 -1.747161
C -0.138043 -1.526753 1.389179
H -1.001969 -1.170925 0.825422
H 3.289026 -1.407698 -1.055456
C 2.375462 -1.889704 1.310760
H 3.206763 -1.224145 1.036823
H 2.198294 -1.767971 2.385697
H -0.867757 -0.309858 -0.933715
H -0.014315 -0.882178 2.271990
C -0.714598 -2.129076 -2.063312
H -0.017333 -2.656473 -2.725646
H -0.964148 -2.787755 -1.220889
H -1.634586 -1.918797 -2.619915
C 2.476915 -0.901037 -2.992629
H 2.502540 0.192331 -2.946741
H 3.406368 -1.237569 -3.465952
H 1.647949 -1.194516 -3.647444
C 2.794927 -3.342559 1.027442
H 1.952518 -4.035216 1.119522
H 3.225919 -3.456557 0.028810
H 3.556850 -3.634128 1.759072
C -0.413813 -2.960659 1.867493
H -0.466268 -3.659863 1.024077
H 0.346348 -3.312420 2.574031
H -1.383742 -2.967908 2.375860
I -3.671714 0.219472 0.008183
54
C_Et_conf_8 Eopt -1028.039848
C 0.632473 2.744686 1.098687
C 0.205801 1.582474 0.149940
C 2.728728 1.251993 -0.489690
C 3.093291 2.421019 0.475470
H -0.051117 2.771133 1.956066
H 3.197347 3.354624 -0.094989
C -0.043689 2.114850 -1.302821
H -0.852135 2.855640 -1.238913
C 2.391098 1.766734 -1.927002
H 2.168038 0.909489 -2.582535
H -0.731422 1.134487 0.494076
H 3.581970 0.572744 -0.569382
C 1.202515 2.727661 -1.938991
H 0.977436 3.014441 -2.974558
H 1.471064 3.655695 -1.415658
C 2.073304 2.614268 1.601588
H 2.130746 1.738742 2.271568

H 2.342193 3.489885 2.206574
H -0.418339 1.300632 -1.937886
H 3.288035 2.252525 -2.334847
H 0.510394 3.704912 0.579019
H 4.076153 2.215447 0.917124
B 1.397602 0.607905 -0.023288
C 1.125979 -1.361174 -0.754729
C 1.249004 -1.145670 0.617742
C 2.584066 -1.330559 1.329375
H 2.755614 -0.477450 2.002296
C 0.029289 -1.228079 1.529449
H -0.051363 -2.296322 1.781970
C 2.282409 -1.774122 -1.632067
H 3.158644 -1.139474 -1.468673
H -0.887997 -0.983026 0.988870
C -0.216021 -1.340448 -1.431120
H -0.925197 -0.690180 -0.911579
H -0.093249 -0.959850 -2.452306
H 3.406776 -1.331065 0.610510
H 1.981314 -1.648822 -2.677275
C 2.610207 -2.623454 2.154307
H 2.294261 -3.485855 1.554507
H 3.630162 -2.805511 2.511771
H 1.952057 -2.561489 3.027533
C 0.091711 -0.415947 2.818650
H -0.815298 -0.608738 3.402698
H 0.953724 -0.680676 3.442454
H 0.127993 0.660551 2.620813
C -0.808207 -2.758091 -1.487263
H -0.847604 -3.208833 -0.487689
H -1.830646 -2.694719 -1.875293
H -0.226121 -3.410528 -2.147761
C 2.692050 -3.235794 -1.385929
H 3.380960 -3.547376 -2.179318
H 3.206278 -3.351660 -0.426463
H 1.828733 -3.910169 -1.399749
I -3.683424 0.057721 0.044715
54
C_Et_conf_9 Eopt -1028.038993
C -0.426230 2.354337 0.568738
C 0.050326 1.436350 -0.602449
C 2.475211 1.583573 0.395737
C 1.935129 2.518961 1.516593
H -0.823106 1.731064 1.382894
H 2.731491 3.227099 1.783941
C 0.520982 2.278568 -1.828147
H 0.261793 3.334015 -1.666417
C 2.897043 2.401750 -0.872316
H 3.936340 2.148686 -1.116252
H -0.798774 0.809304 -0.885148
H 3.344715 1.050178 0.788631
C 2.021961 2.151025 -2.106089
H 2.220488 1.132713 -2.480761
H 2.315189 2.836023 -2.912192
C 0.673759 3.275010 1.096474
H 0.290887 3.847674 1.951355
H 0.932386 4.015019 0.325670
H -0.037966 1.953251 -2.713912
H 2.883969 3.472820 -0.626822
H -1.273804 2.945082 0.195337
H 1.729124 1.925021 2.418931
B 1.274402 0.689318 -0.018248
C 1.369453 -1.242815 -0.629380
C 1.265666 -1.074242 0.747108
C 2.467622 -1.232489 1.650333
H 2.427270 -0.491555 2.458246
C -0.093056 -1.141080 1.437184
H -0.369144 -2.207598 1.454805
C 2.711303 -1.339559 -1.330895
H 3.382641 -0.538760 -0.992014
H -0.863628 -0.658280 0.827736
C 0.178208 -1.498127 -1.513928
H -0.764545 -1.325910 -0.992274
H 0.219921 -0.808514 -2.369248
H 3.395470 -1.058472 1.100469
H 2.535023 -1.177508 -2.400334
C 2.476071 -2.646984 2.245430
H 1.626639 -2.809424 2.918007
H 2.429110 -3.407135 1.455723
H 3.397781 -2.799480 2.818664
C -0.154175 -0.567494 2.848594
H 0.443830 -1.140473 3.565639
H 0.186522 0.474129 2.878311
H -1.195459 -0.582492 3.190005
C 0.197920 -2.941888 -2.035483
H 1.043899 -3.122836 -2.708232
H 0.242261 -3.662024 -1.208216
H -0.726777 -3.121677 -2.594190
C 3.426811 -2.686342 -1.135553
H 4.238337 -2.759758 -1.868591
H 3.868037 -2.773691 -0.138069
H 2.751729 -3.534299 -1.289167
I -3.704107 -0.191323 -0.097603
42
C_Me_conf_1 Eopt -870.979903
C 3.227719 0.690588 -1.624603
C 2.666084 -0.688890 -1.157942
C 2.379214 0.306353 1.236321
C 2.997720 1.651512 0.753996
H 2.406436 1.254391 -2.095833
H 3.591381 2.082916 1.572661
C 3.725409 -1.668509 -0.612920
H 4.497155 -1.827458 -1.380836
C 3.403071 -0.729774 1.753090
H 2.833034 -1.576563 2.166783
H 2.194608 -1.153194 -2.029566
H 1.708306 0.537292 2.069755
C 4.389279 -1.265840 0.709966
H 4.896358 -2.145857 1.128291
H 5.180531 -0.530778 0.530119
C 3.851910 1.555390 -0.520169
H 4.003875 2.568386 -0.916061
H 4.850570 1.181488 -0.276550
H 3.228013 -2.640610 -0.473278
H 3.959389 -0.294066 2.595907
H 3.965726 0.509857 -2.419424
H 2.170900 2.357552 0.568992
B 1.672094 -0.247278 -0.037161
C 0.017369 -0.413811 -0.357861
C -0.332173 0.877602 0.086748
C -0.567697 1.265968 1.493731
H 0.130209 2.068702 1.774678
H -1.570404 1.719662 1.505055
C -0.492251 1.973881 -0.905425
H -0.481231 2.956789 -0.426957
H -1.479761 1.825394 -1.375115
C -0.123541 -1.625291 0.582734
H 0.545108 -2.437080 0.263870
H -1.156848 -1.984648 0.473017
H 0.252176 1.926821 -1.706897
C -0.286881 -0.763066 -1.806115
H -1.375681 -0.753579 -1.944603
H 0.162727 -0.065876 -2.520723
H 0.079588 -1.769279 -2.031769
H -0.539152 0.449073 2.214191
H 0.061017 -1.404685 1.637349
I -3.803885 -0.162546 0.065914
42
C_Me_conf_2 Eopt -870.979627
C 3.504085 1.058796 -1.346308
C 2.606671 -0.217371 -1.291448
C 2.430479 0.102441 1.307342
C 3.328821 1.370850 1.187526
H 3.320110 1.575694 -2.296416
H 4.373087 1.107851 1.409074
C 3.440762 -1.509610 -1.039364
H 4.140168 -1.643053 -1.876250
C 3.282331 -1.194043 1.479507
H 2.611015 -2.054009 1.626108
H 2.077418 -0.336880 -2.241513

H	1.780227	0.192172	2.183335
C	4.200026	-1.468328	0.287679
H	4.719119	-2.423606	0.440590
H	4.983592	-0.698935	0.239310
C	3.248597	2.033716	-0.191470
H	2.238712	2.465068	-0.312854
H	3.952125	2.874581	-0.247387
H	2.768396	-2.382685	-1.047261
H	3.873272	-1.094852	2.400600
H	4.563807	0.766068	-1.351140
H	3.022904	2.095986	1.952066
B	1.687344	-0.123266	-0.040453
C	-0.011359	-0.603273	-0.188831
C	-0.295384	0.756443	0.008798
C	-0.469877	1.388200	1.339984
H	0.339968	2.117005	1.503727
H	-1.400365	1.971037	1.288423
C	-0.376140	1.689589	-1.152859
H	-0.297260	2.731927	-0.831224
H	-1.363932	1.540270	-1.617357
C	-0.179754	-1.614071	0.940680
H	0.381828	-2.527654	0.712611
H	-1.247585	-1.873945	0.977693
H	0.377225	1.469369	-1.919540
C	-0.256173	-1.228900	-1.549435
H	-1.288818	-1.604897	-1.545472
H	-0.151995	-0.532121	-2.383539
H	0.417322	-2.078937	-1.709650
H	-0.530002	0.686418	2.171942
H	0.122913	-1.249345	1.924434
I	-3.753556	-0.072884	0.037757

58

D_Et_conf_1 Eopt -845.402566

C	2.210456	-1.792250	1.064091
C	1.682009	-0.335683	1.213096
C	1.847589	-0.057708	-1.366397
C	2.391842	-1.510111	-1.466544
H	1.750055	-2.425295	1.835063
H	3.491846	-1.504789	-1.503998
C	2.853513	0.670686	1.380884
H	3.437310	0.391860	2.271065
C	2.988853	0.974544	-1.138988
H	2.563705	1.991486	-1.112172
H	1.071679	-0.269452	2.120103
H	1.361336	0.207406	-2.311365
C	3.765585	0.723836	0.154499
H	4.523589	1.507718	0.289268
H	4.318381	-0.223105	0.069622
C	1.928981	-2.402766	-0.311706
H	0.841192	-2.559099	-0.410328
H	2.393387	-3.395886	-0.387588
H	2.449043	1.675050	1.577843
H	3.672722	0.948954	-2.000554
H	3.293648	-1.821637	1.257482
H	2.059542	-1.956612	-2.413284
B	0.914891	0.126667	-0.098276
C	-0.542087	0.812789	-0.117830
C	-1.619193	-0.331496	0.053647
C	-1.607800	-1.197604	-1.242797
H	-2.202575	-0.657591	-1.991064
C	-1.443587	-1.251531	1.285298
H	-0.462177	-1.740371	1.242310
H	-2.164962	-2.066440	1.164960
C	-0.736608	1.554170	-1.469771
H	0.114079	2.241345	-1.580817
C	-0.628817	1.844564	1.038763
H	-1.681806	2.074056	1.242316
H	-0.228870	1.399443	1.953918
H	-0.582280	-1.209483	-1.637096
H	-0.626634	0.850304	-2.301044
O	-2.864001	0.358979	0.190101
C	-4.051920	-0.378654	0.115516
H	-4.089060	-1.203716	0.845167
H	-4.869636	0.314935	0.344952
H	-4.225032	-0.790223	-0.891642
C	-2.081105	-2.649320	-1.173137
H	-1.410518	-3.266714	-0.561997
H	-3.093548	-2.754483	-0.766651
H	-2.084828	-3.074046	-2.185014
C	-1.696080	-0.647471	2.666871
H	-0.828698	-0.123573	3.080006
H	-2.533823	0.060442	2.632207
H	-1.958183	-1.444141	3.374383
C	0.125428	3.153115	0.788816
H	-0.373119	3.782456	0.041967
H	0.192886	3.734126	1.717439
H	1.150608	2.976394	0.435129
C	-2.028416	2.347574	-1.681637
H	-2.292968	2.952503	-0.805444
H	-1.911085	3.025125	-2.538622
H	-2.879513	1.687788	-1.881985

58

D_Et_conf_10 Eopt -845.399292

C	-2.520288	-1.269042	-1.406772
C	-2.029966	0.181281	-1.167465
C	-1.491538	-0.411666	1.290527
C	-1.926226	-1.872420	1.023553
H	-1.723502	-1.816844	-1.932853
H	-2.364242	-2.301606	1.937993
C	-3.160156	1.126132	-0.680819
H	-3.974318	1.128612	-1.422029
C	-2.640195	0.519764	1.752342
H	-2.187734	1.478679	2.052613
H	-1.681217	0.565032	-2.136008
H	-0.766222	-0.426514	2.114326
C	-3.726661	0.806168	0.708015
H	-4.325051	1.662650	1.049107
H	-4.426295	-0.033296	0.648017
C	-2.900820	-2.061638	-0.146956
H	-2.939443	-3.129761	-0.402753
H	-3.916039	-1.802096	0.168764
H	-2.770387	2.154948	-0.657327
H	-3.101784	0.104498	2.661847
H	-3.376659	-1.251126	-2.098864
H	-1.021026	-2.466354	0.814660
B	-0.896822	0.177712	-0.056425
C	0.594459	0.770950	-0.209690
C	1.661611	-0.390850	-0.114886
C	1.730623	-1.160784	1.222786
H	0.736599	-1.540255	1.473553
C	1.354014	-1.386398	-1.280523
H	1.835768	-0.979830	-2.178914
H	0.277488	-1.350563	-1.494197
C	0.675923	1.835099	0.917860
H	-0.156760	2.537840	0.755348
C	0.782920	1.465846	-1.586626
H	1.850231	1.654574	-1.748034
H	0.473748	0.773211	-2.379198
H	2.331827	-2.057826	1.041346
H	0.456851	1.368968	1.883595
O	2.932510	0.234970	-0.332987
C	4.048134	-0.590777	-0.525477
H	4.187252	-1.319861	0.288854
H	4.001467	-1.136738	-1.481188
H	4.927207	0.064134	-0.549513
C	2.365979	-0.461960	2.423463
H	3.334355	-0.024889	2.150337
H	1.747644	0.329908	2.855738
H	2.546050	-1.200867	3.214854
C	1.731485	-2.859140	-1.128980
H	1.144882	-3.349828	-0.341488
H	1.518494	-3.382706	-2.069736
H	2.792987	-3.009257	-0.899775
C	0.025426	2.777430	-1.785884
H	0.104765	3.099383	-2.832372
H	-1.041247	2.684043	-1.552268
H	0.430336	3.586070	-1.164745
C	1.962743	2.652056	1.029006
H	2.802812	2.033254	1.356289
H	2.244218	3.098383	0.066863

H 1.826166 3.467396 1.752342
58
D_Et_conf_11 Eopt -845.403854
C 2.227510 -1.951581 0.770202
C 1.601298 -0.600828 1.173885
C 1.884531 0.222875 -1.259933
C 2.647116 -1.077316 -1.600974
H 1.417731 -2.652040 0.509223
H 3.468855 -0.842462 -2.296031
C 2.654859 0.440081 1.648538
H 3.302569 -0.032515 2.403716
C 2.830090 1.355539 -0.778402
H 2.249771 2.286244 -0.701303
H 0.946821 -0.782718 2.033878
H 1.417537 0.580628 -2.187510
C 3.538543 1.076749 0.557265
H 3.944402 2.022167 0.944544
H 4.411416 0.442360 0.370636
C 3.203333 -1.865483 -0.406146
H 3.454410 -2.882388 -0.739633
H 4.147715 -1.427928 -0.064574
H 2.113971 1.237562 2.176665
H 3.587376 1.541200 -1.555423
H 2.741042 -2.391966 1.638818
H 1.963079 -1.736382 -2.154605
B 0.850552 0.045222 -0.066464
C -0.636725 0.686944 -0.011869
C -1.785695 -0.403708 0.083986
C -2.004413 -1.119883 -1.260969
H -2.786347 -1.875580 -1.126890
C -1.499860 -1.425973 1.202689
H -1.234316 -0.871506 2.109659
H -0.618723 -2.020843 0.934968
C -0.839483 1.594054 -1.250603
H 0.015054 2.283245 -1.292726
C -0.683170 1.560197 1.282689
H -1.730164 1.785123 1.510890
H -0.305875 0.975429 2.130698
H -2.398847 -0.400337 -1.988137
H -0.752907 0.992198 -2.164152
O -2.979295 0.305779 0.466963
C -4.231964 -0.042308 -0.060024
H -4.483786 -1.103164 0.079226
H -4.971894 0.558283 0.482281
H -4.324646 0.192289 -1.132098
C -0.768477 -1.788277 -1.850113
H -0.091105 -1.051414 -2.292926
H -0.206691 -2.365651 -1.102759
H -1.054919 -2.485735 -2.646912
C -2.651078 -2.366321 1.547360
H -2.973369 -2.971664 0.690438
H -2.335137 -3.061132 2.335616
H -3.516373 -1.809927 1.925947
C 0.086744 2.880567 1.232787
H -0.356775 3.588841 0.522210
H 0.070249 3.355326 2.222404
H 1.138920 2.748862 0.953738
C -2.123545 2.421500 -1.330347
H -2.995566 1.803451 -1.568000
H -2.341013 2.937670 -0.387163
H -2.028550 3.181143 -2.118216
58
D_Et_conf_12 Eopt -845.400985
C 2.201562 -1.307894 1.610546
C 1.694922 0.121381 1.308617
C 1.715022 -0.395939 -1.225053
C 1.955252 -1.889414 -0.897349
H 1.364062 -1.859317 2.064134
H 2.504852 -2.359973 -1.726981
C 2.833284 1.148389 1.063992
H 3.412028 1.275915 1.991127
C 3.035384 0.403732 -1.382122
H 2.793934 1.337281 -1.909982
H 1.143074 0.467119 2.190683
H 1.209131 -0.347091 -2.198019
C 3.772835 0.765414 -0.081471
H 4.452876 1.604950 -0.283237
H 4.414193 -0.061387 0.243589
C 2.723581 -2.120346 0.409499
H 2.683430 -3.188851 0.662923
H 3.782909 -1.902228 0.236172
H 2.384571 2.132849 0.848245
H 3.714162 -0.151247 -2.048937
H 2.983888 -1.264971 2.384982
H 0.982496 -2.405060 -0.849380
B 0.850732 0.186204 -0.027200
C -0.628150 0.819203 -0.143713
C -1.665917 -0.370800 -0.138842
C -1.663557 -1.079408 -1.524490
H -2.271095 -0.458569 -2.196026
C -1.402981 -1.431293 0.954642
H -0.413536 -1.874908 0.769826
H -2.106931 -2.251688 0.782052
C -0.735809 1.621951 -1.464328
H -0.459756 0.986032 -2.311465
C -0.827577 1.808677 1.043380
H -0.848305 1.259618 1.985425
H 0.087113 2.417663 1.096765
H -0.646338 -1.047410 -1.924780
H -1.780861 1.917952 -1.632146
O -2.928553 0.254982 0.091749
C -4.093081 -0.520383 0.010668
H -4.918299 0.113823 0.356494
H -4.312538 -0.834729 -1.021628
H -4.057090 -1.414012 0.654008
C -2.126123 -2.535532 -1.615223
H -3.115892 -2.710215 -1.178883
H -2.173478 -2.828714 -2.671840
H -1.419185 -3.214925 -1.122026
C -1.539043 -1.024603 2.420181
H -0.643692 -0.530248 2.811182
H -2.395670 -0.352056 2.560882
H -1.709633 -1.917749 3.034161
C -2.022001 2.769182 1.030037
H -2.948160 2.263465 1.315435
H -1.841197 3.584038 1.745031
H -2.188149 3.227500 0.047645
C 0.158749 2.861519 -1.503445
H 1.191081 2.619072 -1.211460
H 0.193923 3.282126 -2.516308
H -0.191265 3.652060 -0.828807
58
D_Et_conf_13 Eopt -845.404029
C -2.499782 0.570562 1.662126
C -1.531289 -0.542335 1.169472
C -1.883013 0.235806 -1.270923
C -2.755699 1.421798 -0.780193
H -1.889137 1.356783 2.127762
H -3.541637 1.617747 -1.525579
C -2.248051 -1.866499 0.834052
H -2.749105 -2.251694 1.735510
C -2.716351 -1.036566 -1.544527
H -2.084616 -1.743174 -2.101652
H -0.845193 -0.736415 2.002889
H -1.440429 0.548999 -2.226134
C -3.264026 -1.761606 -0.306642
H -3.578854 -2.773037 -0.600187
H -4.171084 -1.266599 0.056622
C -3.408546 1.214655 0.596489
H -3.751529 2.188620 0.973592
H -4.316980 0.615883 0.473578
H -1.489814 -2.619278 0.563531
H -3.551696 -0.783440 -2.216472
H -3.127394 0.161449 2.469466
H -2.133048 2.327601 -0.760163
B -0.808961 0.037778 -0.117901
C 0.707220 0.606344 -0.132767
C 1.794458 -0.541437 -0.047510
C 1.555218 -1.613341 1.050514
H 0.532050 -1.992950 0.976652

C	1.936308	-1.283045	-1.393830
H	2.681623	-2.076520	-1.257103
H	2.353604	-0.593652	-2.138521
C	0.838320	1.524952	1.124660
H	0.436739	1.001069	1.997536
C	0.911565	1.462347	-1.410333
H	0.754216	0.839574	-2.299524
H	0.093208	2.194969	-1.439999
H	2.192334	-2.470943	0.794841
H	1.902065	1.686190	1.328776
O	3.028092	0.129036	0.233816
C	4.217274	-0.611768	0.180724
H	4.533321	-0.821443	-0.853373
H	4.992910	-0.000969	0.658155
H	4.151258	-1.566691	0.725431
C	1.869065	-1.222457	2.493914
H	1.997704	-2.127552	3.101007
H	2.797704	-0.641382	2.552203
H	1.076715	-0.629728	2.961847
C	0.652784	-1.907173	-1.927158
H	0.875352	-2.641798	-2.710945
H	0.092989	-2.433484	-1.140734
H	-0.006391	-1.152520	-2.367640
C	2.232458	2.217872	-1.570953
H	2.510238	2.758877	-0.657763
H	3.062990	1.543428	-1.805322
H	2.144997	2.949765	-2.385641
C	0.154697	2.890691	1.040541
H	0.619850	3.542506	0.291462
H	-0.912325	2.816535	0.798514
H	0.231764	3.399291	2.010421

58

D_Et_conf_14 Eopt -845.401527

C	-2.099179	-1.924204	-0.881142
C	-1.575219	-0.513079	-1.226443
C	-1.870333	0.168516	1.245828
C	-2.605755	-1.164749	1.517884
H	-1.244309	-2.576835	-0.637892
H	-3.460634	-0.975098	2.186449
C	-2.712142	0.464067	-1.635440
H	-3.358319	-0.036886	-2.373614
C	-2.841160	1.301054	0.814851
H	-2.276896	2.241457	0.728845
H	-0.931617	-0.604645	-2.109697
H	-1.416386	0.489045	2.192613
C	-3.587701	1.025211	-0.497748
H	-4.055459	1.958186	-0.842655
H	-4.418727	0.341602	-0.293438
C	-3.091663	-1.942640	0.283900
H	-3.284649	-2.984332	0.576621
H	-4.055108	-1.552198	-0.062159
H	-2.245634	1.302955	-2.169446
H	-3.573916	1.465902	1.619334
H	-2.573263	-2.365817	-1.771013
H	-1.922681	-1.815545	2.081364
B	-0.829790	0.078833	0.049010
C	0.657193	0.739633	0.042558
C	1.840769	-0.315442	-0.011076
C	2.032458	-1.044593	1.327703
H	2.867973	-1.744744	1.215160
C	1.646845	-1.324434	-1.172581
H	1.944011	-0.813424	-2.098612
H	0.582448	-1.534776	-1.280965
C	0.780218	1.650550	1.292303
H	-0.107017	2.298650	1.307810
C	0.731820	1.606522	-1.250820
H	1.776808	1.884437	-1.424137
H	0.434236	0.993157	-2.111301
H	2.354154	-0.309212	2.074864
H	0.687254	1.035813	2.196653
O	3.016991	0.473906	-0.237578
C	4.222354	-0.154570	-0.573139
H	4.529943	-0.914972	0.162068
H	4.189061	-0.618169	-1.571509
H	4.988758	0.629787	-0.589488
C	0.803037	-1.797463	1.823035
H	0.111708	-1.125229	2.341070
H	0.254289	-2.272761	0.996302
H	1.079192	-2.592292	2.526699
C	2.351082	-2.680166	-1.074009
H	3.432655	-2.606874	-0.923023
H	1.941640	-3.282673	-0.252675
H	2.184319	-3.241199	-2.002451
C	-0.106762	2.885469	-1.242708
H	-0.090057	3.351061	-2.236561
H	-1.157443	2.705253	-0.985877
H	0.282631	3.621767	-0.528637
C	2.015613	2.542261	1.432950
H	1.842457	3.291566	2.217563
H	2.906122	1.964747	1.699890
H	2.246628	3.075218	0.501986

58

D_Et_conf_15 Eopt -845.402489

C	2.387885	-1.721539	1.025120
C	1.602886	-0.395631	1.232986
C	1.886651	0.146946	-1.290506
C	2.670619	-1.185302	-1.466058
H	1.978479	-2.492112	1.692947
H	3.753229	-0.987278	-1.454976
C	2.584829	0.761196	1.574501
H	3.150307	0.483161	2.476655
C	2.845571	1.308459	-0.901246
H	2.282336	2.252852	-0.854088
H	0.939423	-0.510432	2.096194
H	1.429590	0.421932	-2.248287
C	3.555475	1.069583	0.432713
H	4.165370	1.946499	0.690396
H	4.259755	0.232185	0.320528
C	2.338365	-2.245410	-0.411266
H	1.321380	-2.615991	-0.603009
H	3.009057	-3.109038	-0.522202
H	2.018937	1.666407	1.834197
H	3.589346	1.435679	-1.702099
H	3.436506	-1.585988	1.330678
H	2.452636	-1.602091	-2.458996
B	0.850142	0.078523	-0.088511
C	-0.660880	0.661946	-0.124743
C	-1.757945	-0.486156	-0.011140
C	-2.038955	-1.160527	-1.372330
H	-2.865602	-1.865351	-1.217797
C	-1.421103	-1.609105	1.004211
H	-0.387335	-1.940369	0.846389
H	-2.039567	-2.481869	0.748902
C	-0.865023	1.476728	-1.428162
H	-0.045733	2.206252	-1.491863
C	-0.777112	1.635570	1.094093
H	-1.835345	1.800527	1.317024
H	-0.351396	1.160371	1.983457
H	-2.408081	-0.409717	-2.078125
H	-0.719160	0.823341	-2.297422
O	-2.955844	0.179265	0.407774
C	-4.157710	-0.543830	0.419707
H	-4.855700	0.008247	1.060712
H	-4.604121	-0.624810	-0.583979
H	-4.043518	-1.558742	0.831055
C	-0.886695	-1.940200	-1.990549
H	-1.174281	-2.309060	-2.983115
H	0.012986	-1.326988	-2.119028
H	-0.625608	-2.813366	-1.380864
C	-1.656464	-1.292702	2.478204
H	-1.001611	-0.503974	2.859189
H	-2.690500	-0.970100	2.649576
H	-1.479262	-2.191458	3.082950
C	-0.106919	3.002233	0.921069
H	-0.076041	3.523365	1.886731
H	0.924575	2.929715	0.556507
H	-0.653639	3.644165	0.220701
C	-2.189202	2.227358	-1.588224
H	-2.131589	2.909009	-2.447747
H	-3.032344	1.547214	-1.750543

H -2.433091 2.823377 -0.700561
58
D_Et_conf_16 Eopt -845.398913
C -2.031050 -1.899172 -1.156879
C -1.484272 -0.464959 -1.356271
C -2.015013 0.016353 1.135938
C -2.604168 -1.414500 1.264382
H -1.201922 -2.578790 -0.897862
H -3.436529 -1.383824 1.983912
C -2.626070 0.516174 -1.777527
H -3.521284 -0.062167 -2.051444
C -3.166186 0.981811 0.693904
H -3.243923 1.811878 1.408034
H -0.760976 -0.500892 -2.176854
H -1.664128 0.323946 2.129758
C -2.998482 1.558694 -0.714717
H -2.210280 2.326065 -0.682440
H -3.916829 2.082175 -1.014613
C -3.098591 -1.977441 -0.067333
H -3.418182 -3.019929 0.068062
H -3.992545 -1.426519 -0.394645
H -2.318517 1.048182 -2.687533
H -4.124728 0.446606 0.752725
H -2.443173 -2.264011 -2.109928
H -1.860378 -2.102124 1.684622
B -0.864537 0.044108 0.023453
C 0.619975 0.703245 0.156620
C 1.833233 -0.312183 0.156728
C 1.945955 -1.113967 1.462644
H 2.810233 -1.782329 1.373206
C 1.775507 -1.253407 -1.072694
H 2.131673 -0.682353 -1.940643
H 0.728641 -1.485324 -1.280233
C 0.714932 1.567817 1.437820
H 0.470252 0.941954 2.306509
C 0.691374 1.617396 -1.111945
H 0.705658 0.982848 -2.005452
H -0.258901 2.164915 -1.174701
H 2.189469 -0.415272 2.271283
H 1.754908 1.889293 1.574546
O 2.990664 0.532139 0.097903
C 4.238579 -0.018482 -0.214517
H 4.538839 -0.821385 0.477895
H 4.281272 -0.404574 -1.245193
H 4.969659 0.794514 -0.127223
C 0.706862 -1.919387 1.820029
H -0.044400 -1.279990 2.295148
H 0.249221 -2.376058 0.930037
H 0.940158 -2.730888 2.520298
C 2.517664 -2.589466 -0.994324
H 3.582081 -2.486868 -0.759912
H 2.073165 -3.250827 -0.239390
H 2.440103 -3.102428 -1.961468
C 1.816683 2.653584 -1.233047
H 1.509319 3.444653 -1.930971
H 2.055954 3.126182 -0.273281
H 2.741546 2.207287 -1.609412
C -0.186664 2.799944 1.472731
H -1.244302 2.539884 1.353645
H -0.084159 3.314616 2.436961
H 0.068783 3.521595 0.686812
58
D_Et_conf_17 Eopt -845.400702
C -2.367881 -1.629405 -1.229414
C -1.878833 -0.158910 -1.237874
C -1.670280 -0.246232 1.354437
C -2.126904 -1.724865 1.307246
H -1.528813 -2.304275 -1.450894
H -2.671357 -1.965190 2.233127
C -3.101971 0.805199 -1.080446
H -4.034817 0.225914 -1.143435
C -2.892643 0.719616 1.469035
H -2.754373 1.370905 2.341885
H -1.401037 0.029006 -2.208030
H -1.055541 -0.116271 2.251280
C -3.094337 1.599249 0.230467
H -2.273502 2.334728 0.192624
H -4.023371 2.178264 0.325055
C -3.010930 -2.034959 0.098118
H -3.252121 -3.106755 0.080089
H -3.971606 -1.511140 0.210760
H -3.126790 1.507926 -1.923519
H -3.808457 0.139883 1.660362
H -3.094476 -1.766131 -2.044858
H -1.242114 -2.381940 1.290764
B -0.905158 0.089064 -0.001406
C 0.588703 0.695656 -0.074477
C 1.622665 -0.505089 0.061427
C 1.708411 -1.056723 1.508693
H 0.708721 -1.013498 1.954431
C 1.137782 -1.666479 -0.835519
H 0.794977 -1.289833 -1.807744
H 0.244156 -2.069277 -0.337145
C 0.710699 1.720678 1.083082
H -0.173763 2.376891 1.063874
C 0.826068 1.432927 -1.412982
H 1.855862 1.804221 -1.433034
H 0.736214 0.730235 -2.251153
H 1.955841 -2.122680 1.441712
H 0.642221 1.192534 2.039478
O 2.963396 -0.117152 -0.251765
C 3.443762 -0.048221 -1.573243
H 4.322473 -0.701928 -1.668471
H 2.704277 -0.349149 -2.324080
H 3.761211 0.979401 -1.800600
C 2.730499 -0.428650 2.454209
H 3.739465 -0.500223 2.035009
H 2.532398 0.624411 2.675789
H 2.719351 -0.973827 3.407256
C 2.111539 -2.821232 -1.057313
H 1.613010 -3.617866 -1.624169
H 2.995324 -2.516503 -1.626497
H 2.458125 -3.260297 -0.114830
C -0.096584 2.619853 -1.686611
H 0.164360 3.081935 -2.647668
H -1.149035 2.323382 -1.745125
H -0.008647 3.396977 -0.916957
C 1.960580 2.603458 1.068430
H 2.869180 2.006416 0.930771
H 1.921567 3.343274 0.258709
H 2.047641 3.157146 2.012789
58
D_Et_conf_18 Eopt -845.398420
C -2.571029 0.364560 1.859120
C -1.470357 -0.564646 1.281278
C -2.004602 0.311118 -1.083856
C -2.969913 1.351947 -0.450017
H -2.066479 1.232349 2.311026
H -3.751470 1.609776 -1.180819
C -2.003995 -1.961570 0.894056
H -2.436298 -2.445931 1.783152
C -2.727782 -1.020550 -1.412985
H -2.101805 -1.574937 -2.124019
H -0.753822 -0.702778 2.098783
H -1.650604 0.728672 -2.037225
C -3.050904 -1.953146 -0.228522
H -3.165541 -2.976362 -0.612802
H -4.025479 -1.696177 0.199209
C -3.609240 0.887472 0.858868
H -4.144133 1.732821 1.314136
H -4.372754 0.129921 0.655364
H -1.153026 -2.593518 0.594672
H -3.661401 -0.794215 -1.952265
H -3.078955 -0.157092 2.685583
H -2.423805 2.283800 -0.242712
B -0.840568 0.109341 -0.022823
C 0.659575 0.723172 -0.124208
C 1.839193 -0.333006 -0.142955
C 1.751862 -1.313389 1.051136
H 2.119641 -0.781289 1.939471

C	1.915924	-1.093093	-1.478759
H	2.727444	-1.826540	-1.409579
H	2.219322	-0.383238	-2.256804
C	0.698998	1.589717	1.176000
H	-0.225123	2.189255	1.188345
C	0.841947	1.626913	-1.373874
H	1.908753	1.859502	-1.470599
H	0.574356	1.050044	-2.269652
H	0.700985	-1.526185	1.245052
H	0.617170	0.929596	2.048365
O	3.026676	0.466208	-0.050110
C	4.256045	-0.145495	0.219302
H	4.514462	-0.928312	-0.511847
H	5.017305	0.641603	0.156244
H	4.297410	-0.577719	1.231563
C	2.450943	-2.669461	0.926393
H	1.971198	-3.293457	0.160884
H	3.514675	-2.598483	0.678092
H	2.368723	-3.206013	1.880293
C	0.626639	-1.790571	-1.885803
H	-0.093602	-1.066505	-2.281795
H	0.805210	-2.538050	-2.668651
H	0.156692	-2.309728	-1.038302
C	0.068048	2.943708	-1.423247
H	-1.011440	2.793008	-1.517309
H	0.245871	3.570702	-0.540920
H	0.387824	3.520661	-2.301159
C	1.872762	2.550464	1.399716
H	2.175298	3.060457	0.477650
H	1.583560	3.316014	2.132761
H	2.754585	2.027111	1.780947

58

D_Et_conf_19 Eopt -845.398316

C	-2.103807	0.083015	-2.085972
C	-1.406088	0.870212	-0.947370
C	-2.189039	-0.912989	0.752913
C	-2.741690	-1.762132	-0.422804
H	-1.320666	-0.455355	-2.643611
H	-3.595488	-2.359771	-0.068485
C	-2.319734	1.900589	-0.242142
H	-2.649154	2.655215	-0.972633
C	-3.233781	0.072891	1.325389
H	-2.863815	0.420377	2.300851
H	-0.587866	1.434804	-1.409886
H	-1.925581	-1.610581	1.560421
C	-3.545930	1.305045	0.458689
H	-3.999365	2.077734	1.095323
H	-4.307065	1.061471	-0.289838
C	-3.160732	-0.943623	-1.648979
H	-3.349548	-1.630528	-2.485907
H	-4.118136	-0.449773	-1.453449
H	-1.716313	2.445362	0.502564
H	-4.169011	-0.469634	1.536366
H	-2.549553	0.792205	-2.800893
H	-1.970454	-2.485218	-0.731633
B	-0.923964	-0.164875	0.152410
C	0.567788	-0.564308	0.593132
C	1.680909	0.300158	-0.095308
C	1.756941	0.044453	-1.627908
H	0.739509	-0.103211	-2.007707
C	1.435366	1.806204	0.243123
H	1.982277	1.997672	1.173525
H	0.382542	1.954263	0.495377
C	0.657740	-2.076525	0.220064
H	-0.209116	-2.571168	0.677761
C	0.638797	-0.484160	2.144925
H	0.075761	-1.348373	2.522862
H	1.677516	-0.638164	2.459684
H	2.121245	0.950051	-2.122931
H	0.521735	-2.200529	-0.863043
O	2.926400	-0.091623	0.500757
C	4.052535	0.682207	0.185402
H	4.050971	1.652265	0.707837
H	4.934355	0.118571	0.513119
H	4.146706	0.874676	-0.894819
C	2.637643	-1.109103	-2.117723
H	3.648245	-1.060499	-1.697957
H	2.232099	-2.097083	-1.883713
H	2.727825	-1.045544	-3.210036
C	1.817964	2.876573	-0.777428
H	1.180226	2.839786	-1.670511
H	1.688716	3.868580	-0.325735
H	2.860525	2.797279	-1.108735
C	0.083295	0.767461	2.838762
H	0.806705	1.588171	2.872606
H	-0.821115	1.153266	2.346983
H	-0.188408	0.532986	3.876025
C	1.897435	-2.849105	0.675687
H	1.996014	-2.826614	1.768588
H	1.802700	-3.901816	0.376178
H	2.819921	-2.444697	0.252630

58

D_Et_conf_2 Eopt -845.394629

C	3.031452	0.325584	-1.273083
C	1.594814	-0.290169	-1.340295
C	1.634842	-0.622594	1.237243
C	3.084347	-0.047446	1.286584
H	3.107449	1.146093	-1.998448
H	3.807414	-0.874408	1.346867
C	1.712045	-1.824637	-1.529991
H	2.311343	-2.020690	-2.432176
C	1.666111	-2.154223	0.986968
H	0.638612	-2.553286	0.981208
H	1.078309	0.109775	-2.221496
H	1.162953	-0.455296	2.212542
C	2.349174	-2.523300	-0.329720
H	2.316046	-3.612176	-0.472768
H	3.415206	-2.257447	-0.275272
C	3.438428	0.864633	0.104990
H	2.936723	1.830518	0.260400
H	4.515617	1.081535	0.112418
H	0.721878	-2.258898	-1.720895
H	2.183600	-2.643458	1.825945
H	3.760725	-0.432420	-1.595708
H	3.209683	0.528629	2.212843
B	0.840906	0.035364	0.027120
C	-0.505228	0.911598	0.178250
C	-1.760705	-0.078271	0.210211
C	-1.641797	-1.109304	-0.935178
H	-1.168924	-0.642219	-1.805956
C	-1.833552	-0.801531	1.570371
H	-2.120014	-0.054779	2.317979
H	-0.828855	-1.149433	1.838340
C	-0.686565	1.902777	-1.003034
H	-1.107290	1.377691	-1.866828
C	-0.519083	1.714210	1.505241
H	-1.544680	2.085430	1.651838
H	-0.310760	1.039725	2.342065
H	-0.919285	-1.851512	-0.572438
H	-1.451160	2.629575	-0.699533
O	-2.988100	0.656120	0.169811
C	-3.579608	1.073240	-1.037726
H	-3.546913	2.168208	-1.123386
H	-3.099814	0.650399	-1.927002
H	-4.633320	0.760115	-1.036633
C	-2.928389	-1.830209	-1.381332
H	-2.831870	-2.917060	-1.280131
H	-3.798989	-1.520786	-0.795254
H	-3.151600	-1.620700	-2.433877
C	-2.807133	-1.970161	1.674032
H	-2.817823	-2.342873	2.705966
H	-3.829349	-1.665118	1.419998
H	-2.522881	-2.808425	1.025802
C	0.440018	2.895291	1.627044
H	0.232486	3.681495	0.891224
H	0.340684	3.344599	2.623635
H	1.487841	2.586845	1.512127
C	0.561058	2.666713	-1.490927
H	0.926675	2.273870	-2.446391
H	0.335197	3.729261	-1.644917

H 1.393979 2.618238 -0.784058
58
D_Et_conf_20 Eopt -845.401573
C -2.843740 1.000172 1.273578
C -1.664861 -0.009751 1.321332
C -1.781746 -0.281166 -1.249562
C -2.819021 0.873291 -1.298321
H -2.429523 2.004116 1.442967
H -3.481390 0.726363 -2.164870
C -2.138711 -1.471146 1.488036
H -2.718062 -1.560213 2.419963
C -2.409660 -1.681528 -1.071597
H -1.627749 -2.421545 -1.302007
H -1.079399 0.249864 2.212508
H -1.263740 -0.272296 -2.216501
C -2.982280 -1.999677 0.320843
H -3.082202 -3.089705 0.419203
H -4.000453 -1.605549 0.408406
C -3.663848 1.021828 -0.027868
H -4.218595 1.969326 -0.079078
H -4.426693 0.236144 -0.008979
H -1.254738 -2.114767 1.621570
H -3.195889 -1.831269 -1.828512
H -3.513978 0.804005 2.125210
H -2.281828 1.817269 -1.485936
B -0.846295 0.108277 -0.035333
C 0.664678 0.658166 -0.175872
C 1.623413 -0.523642 0.234499
C 1.699958 -0.742932 1.776300
H 0.683803 -0.692900 2.181720
C 1.178804 -1.887754 -0.354926
H 0.177131 -2.118141 0.038761
H 1.828207 -2.651382 0.091769
C 0.835234 1.868519 0.773092
H 0.500487 1.586514 1.776895
C 0.893894 1.141536 -1.637233
H 0.856367 0.291669 -2.320970
H 0.018497 1.752576 -1.902105
H 2.019906 -1.779742 1.936360
H 1.898748 2.126685 0.864116
O 2.913364 -0.180986 -0.263655
C 3.938522 -1.127450 -0.104467
H 3.819828 -1.987340 -0.782381
H 4.879432 -0.621946 -0.352030
H 4.012039 -1.506554 0.926698
C 2.642664 0.166062 2.576466
H 3.086048 -0.395728 3.408134
H 3.462483 0.540777 1.952123
H 2.132337 1.033078 3.006824
C 1.209592 -2.076523 -1.869724
H 1.237547 -3.147793 -2.105246
H 0.329944 -1.656793 -2.368053
H 2.103126 -1.612137 -2.306704
C 2.149268 1.959334 -1.963878
H 1.994462 2.509632 -2.902471
H 2.389425 2.696785 -1.188457
H 3.025901 1.317926 -2.085115
C 0.052566 3.111480 0.345375
H 0.043382 3.856544 1.150926
H 0.484075 3.589504 -0.542142
H -0.992725 2.870603 0.106861
58
D_Et_conf_3 Eopt -845.399264
C -2.389848 -1.453402 -1.483313
C -2.034226 0.027352 -1.196978
C -1.539438 -0.610001 1.278417
C -1.868589 -2.081072 0.925380
H -1.520670 -1.970697 -1.917609
H -2.276239 -2.586127 1.814294
C -3.306655 0.803676 -0.725560
H -4.194250 0.166703 -0.855867
C -2.818996 0.173485 1.713999
H -2.642995 0.631778 2.696033
H -1.679736 0.469090 -2.136066
H -0.837734 -0.617458 2.118542
C -3.232508 1.276288 0.730614
H -2.494711 2.093313 0.792854
H -4.195048 1.709920 1.035209
C -2.854319 -2.206356 -0.236519
H -3.010362 -3.266359 -0.479498
H -3.835381 -1.820170 0.076933
H -3.462125 1.676997 -1.372682
H -3.656575 -0.527727 1.849176
H -3.178375 -1.487315 -2.250429
H -0.937565 -2.612472 0.666175
B -0.960238 0.098631 -0.021175
C 0.458831 0.855703 -0.073734
C 1.629849 -0.216118 -0.011784
C 1.735478 -0.894525 1.374416
H 1.447848 -0.180844 2.151972
C 1.344316 -1.272273 -1.108442
H 1.462606 -0.809408 -2.094504
H 0.276185 -1.512964 -1.029457
C 0.456569 1.835296 1.130896
H -0.380595 2.531644 0.986521
C 0.603345 1.658437 -1.391362
H 1.640134 1.998776 -1.498980
H 0.413425 0.989201 -2.239923
H 1.002662 -1.711636 1.421562
H 0.209442 1.298722 2.055046
O 2.838126 0.519996 -0.236470
C 3.859791 0.028792 -1.059460
H 3.504441 -0.241712 -2.065313
H 4.585891 0.843932 -1.168208
H 4.381374 -0.837433 -0.624122
C 3.123035 -1.418457 1.740864
H 3.490207 -2.186249 1.052248
H 3.850266 -0.598021 1.754718
H 3.095039 -1.860428 2.744985
C 2.075653 -2.613685 -1.084173
H 3.159982 -2.521179 -1.199408
H 1.876310 -3.165236 -0.157024
H 1.708908 -3.230663 -1.914645
C -0.324772 2.866278 -1.517909
H -0.313259 3.250396 -2.545976
H -1.365435 2.617628 -1.269327
H -0.014739 3.687587 -0.859067
C 1.723226 2.658343 1.360668
H 2.041799 3.175848 0.446416
H 1.542429 3.416230 2.134606
H 2.558563 2.026642 1.682314
58
D_Et_conf_4 Eopt -845.400710
C 2.167137 -1.865545 0.968468
C 1.624931 -0.435637 1.183492
C 1.832415 -0.009846 -1.361585
C 2.416011 -1.427229 -1.537977
H 1.310652 -2.554854 0.892576
H 3.158523 -1.423983 -2.351406
C 2.739666 0.611268 1.456950
H 3.367112 0.257362 2.289823
C 2.896199 1.087951 -1.083825
H 2.390768 2.065771 -1.119611
H 0.988288 -0.451932 2.077726
H 1.343495 0.261007 -2.305945
C 3.638143 0.961285 0.256943
H 4.146729 1.912929 0.466466
H 4.437659 0.219382 0.162688
C 3.035301 -2.048722 -0.280587
H 3.184774 -3.123854 -0.453784
H 4.035538 -1.638114 -0.106848
H 2.253338 1.530258 1.815687
H 3.628375 1.098784 -1.905929
H 2.731241 -2.182132 1.859441
H 1.598075 -2.083914 -1.877348
B 0.868482 0.075240 -0.110972
C -0.593286 0.754593 -0.117629
C -1.677839 -0.380474 0.041894
C -1.634815 -1.256568 -1.246677
H -2.207115 -0.721453 -2.015848

C	-1.537961	-1.292092	1.282878
H	-0.561387	-1.788125	1.262520
H	-2.263844	-2.101176	1.151000
C	-0.786507	1.515806	-1.458891
H	0.071957	2.193853	-1.563197
C	-0.666326	1.770152	1.054610
H	-1.717240	2.004953	1.264407
H	-0.267095	1.305742	1.960851
H	-0.597875	-1.272786	-1.611520
H	-0.685792	0.820558	-2.299038
O	-2.922330	0.316109	0.143620
C	-4.111155	-0.416730	0.043140
H	-4.174875	-1.231411	0.782680
H	-4.931868	0.283863	0.238101
H	-4.255794	-0.841272	-0.963136
C	-2.110199	-2.707755	-1.179819
H	-2.099994	-3.135285	-2.190422
H	-1.446060	-3.322566	-0.559142
H	-3.128134	-2.811940	-0.786860
C	-1.812115	-0.678757	2.656435
H	-2.629599	0.051016	2.599735
H	-2.113493	-1.467103	3.357658
H	-0.940785	-0.179137	3.091304
C	0.098361	3.076243	0.825798
H	0.151352	3.650389	1.759710
H	1.129603	2.896321	0.491574
H	-0.383410	3.713012	0.074229
C	-2.067335	2.330241	-1.659118
H	-1.934066	3.029678	-2.496019
H	-2.925080	1.687799	-1.884570
H	-2.331489	2.914183	-0.768652

58

D_Et_conf_5 Eopt -845.400281

C	-2.779980	0.565586	1.652480
C	-1.534582	-0.330979	1.365959
C	-1.996143	-0.112339	-1.182995
C	-3.239761	0.778688	-0.863811
H	-2.580629	1.182852	2.538301
H	-4.150806	0.162826	-0.890751
C	-1.926206	-1.826854	1.265713
H	-2.345292	-2.156836	2.228209
C	-2.407430	-1.607683	-1.209755
H	-1.552370	-2.231091	-1.514432
H	-0.838744	-0.226112	2.205907
H	-1.622924	0.145745	-2.181866
C	-2.920349	-2.105470	0.140082
H	-3.125547	-3.183305	0.084405
H	-3.881422	-1.623681	0.371271
C	-3.152018	1.493553	0.489217
H	-2.385061	2.282455	0.416505
H	-4.099129	2.006743	0.705674
H	-1.022178	-2.435199	1.095238
H	-3.180379	-1.749289	-1.980200
H	-3.643715	-0.067014	1.907763
H	-3.360957	1.531882	-1.653480
B	-0.914037	0.103698	-0.032839
C	0.553939	0.757145	-0.215406
C	1.655918	-0.368649	-0.131740
C	1.770554	-1.066180	1.245318
H	0.768828	-1.341244	1.590755
C	1.344849	-1.408436	-1.257378
H	1.838559	-1.040701	-2.165997
H	0.274578	-1.369329	-1.487392
C	0.647926	1.830335	0.900444
H	-0.230989	2.485226	0.799072
C	0.674113	1.446522	-1.599043
H	1.713179	1.758439	-1.754916
H	0.456292	0.714536	-2.386354
H	2.289224	-2.018240	1.094192
H	0.521217	1.353046	1.876226
O	2.904693	0.273165	-0.419082
C	4.021327	-0.553155	-0.609533
H	3.963822	-1.118627	-1.553281
H	4.899378	0.101798	-0.656577
H	4.168757	-1.267920	0.215787
C	2.535261	-0.336934	2.351877
H	2.006688	0.526898	2.763935
H	2.714376	-1.033845	3.180788
H	3.511399	0.010188	1.992563
C	1.709532	-2.875990	-1.044580
H	1.117107	-3.329467	-0.238903
H	1.495183	-3.436896	-1.963194
H	2.769575	-3.023850	-0.805770
C	-0.239031	2.653894	-1.805443
H	-1.289600	2.420337	-1.590771
H	0.048827	3.501511	-1.170657
H	-0.185613	2.994115	-2.847654
C	1.890708	2.718710	0.924666
H	1.785503	3.486421	1.703229
H	2.795597	2.138471	1.125714
H	2.038159	3.234760	-0.032920

58

D_Et_conf_6 Eopt -845.396181

C	2.655899	-1.747797	0.799841
C	2.094392	-0.341039	1.152504
C	1.498503	0.012101	-1.343337
C	2.021669	-1.423429	-1.627702
H	1.893233	-2.517458	0.984643
H	2.398985	-1.465595	-2.660339
C	3.222524	0.724498	1.027511
H	4.191771	0.224021	0.882624
C	2.645775	1.060001	-1.442947
H	2.348661	1.843076	-2.153945
H	1.756255	-0.382012	2.196267
H	0.772666	0.243728	-2.123291
C	2.998348	1.724521	-0.109344
H	2.171667	2.394205	0.176803
H	3.883633	2.364171	-0.230305
C	3.114126	-1.858282	-0.652788
H	3.415946	-2.892422	-0.868412
H	4.010265	-1.240232	-0.808206
H	3.309764	1.278307	1.972058
H	3.546319	0.592796	-1.868307
H	3.494167	-1.972403	1.476669
H	1.183958	-2.139037	-1.572264
B	0.896014	-0.049915	0.131422
C	-0.628628	-0.090412	0.662507
C	-1.781305	0.317674	-0.326807
C	-1.778492	1.824176	-0.668492
H	-2.662956	2.010787	-1.290274
C	-1.800421	-0.528347	-1.631693
H	-0.774185	-0.786432	-1.903074
H	-2.172668	0.106116	-2.449462
C	-0.747346	0.725640	1.986981
H	-1.810702	0.914910	2.173560
C	-0.725970	-1.609031	1.051608
H	-0.535835	-2.237597	0.169059
H	0.109857	-1.802202	1.733285
H	-1.947962	2.374461	0.266108
H	-0.413093	0.082292	2.812756
O	-2.998258	0.076014	0.399401
C	-4.208690	0.532888	-0.142709
H	-4.403917	1.587034	0.109672
H	-4.256366	0.427550	-1.237452
H	-5.008869	-0.077978	0.294675
C	-0.586103	2.410696	-1.409384
H	0.349216	2.314554	-0.849455
H	-0.444756	1.937116	-2.388895
H	-0.756340	3.481326	-1.581391
C	-2.620641	-1.820195	-1.623207
H	-3.661513	-1.655056	-1.325690
H	-2.626998	-2.244902	-2.635650
H	-2.202273	-2.579429	-0.955315
C	-1.978512	-2.114834	1.773564
H	-2.232347	-1.471181	2.625147
H	-2.854628	-2.158240	1.123550
H	-1.779568	-3.122605	2.163411
C	0.026636	2.042105	2.098960
H	-0.250864	2.557537	3.028286
H	1.109422	1.873882	2.137855

H	-0.174545	2.732780	1.272583

58

D_Et_conf_7 Eopt -845.399302

C	2.068025	-1.878769	1.139379
C	1.641245	-0.394503	1.329711
C	1.977381	0.014988	-1.213576
C	2.359371	-1.488175	-1.386108
H	1.509040	-2.508094	1.845140
H	3.454153	-1.584593	-1.442530
C	2.876108	0.511576	1.591220
H	3.357080	0.197724	2.529884
C	3.240924	0.858236	-0.884987
H	2.981498	1.926083	-0.847757
H	0.999691	-0.322141	2.213337
H	1.575042	0.391478	-2.162446
C	3.884863	0.473566	0.444886
H	4.720815	1.153091	0.660703
H	4.320832	-0.533174	0.369355
C	1.827365	-2.407196	-0.278643
H	0.741581	-2.529356	-0.419235
H	2.262949	-3.410242	-0.386526
H	2.544482	1.552092	1.743718
H	3.965929	0.745030	-1.705164
H	3.128826	-2.002294	1.402839
H	1.975025	-1.849376	-2.349657
B	0.972443	0.182555	0.006471
C	-0.469265	0.907985	-0.044818
C	-1.597055	-0.208053	-0.025653
C	-1.456140	-1.046481	-1.325710
H	-1.884071	-0.470737	-2.153199
C	-1.492059	-1.084403	1.242472
H	-1.157274	-0.453857	2.071484
H	-0.700645	-1.831936	1.089612
C	-0.657156	1.737963	-1.339615
H	-0.416663	1.113302	-2.207033
C	-0.508202	1.859865	1.183790
H	-0.337343	1.295889	2.107492
H	0.370268	2.517242	1.097043
H	-0.386179	-1.112358	-1.560206
H	-1.719145	2.000589	-1.437342
O	-2.837638	0.507672	0.021111
C	-3.977741	0.021670	-0.635256
H	-3.855736	-0.010985	-1.729055
H	-4.285631	-0.974425	-0.287679
H	-4.787026	0.726536	-0.408897
C	-2.016734	-2.466376	-1.363281
H	-1.754869	-2.927974	-2.324077
H	-1.591313	-3.095843	-0.571359
H	-3.107503	-2.497723	-1.272316
C	-2.773319	-1.774051	1.707733
H	-3.553509	-1.032596	1.917303
H	-3.168932	-2.494722	0.985269
H	-2.573112	-2.318107	2.639698
C	-1.739554	2.747325	1.380774
H	-2.594133	2.169713	1.745754
H	-1.513626	3.533622	2.113915
H	-2.051492	3.234749	0.448585
C	0.176736	3.012701	-1.438200
H	1.243370	2.815385	-1.277190
H	0.072744	3.456283	-2.436865
H	-0.134987	3.770360	-0.708557

58

D_Et_conf_8 Eopt -845.400256

C	2.910287	1.468396	-0.381564
C	1.999039	0.434335	-1.101071
C	1.525705	-0.672941	1.184525
C	2.572140	0.266581	1.841944
H	2.315968	2.352332	-0.105919
H	3.115451	-0.293115	2.619593
C	2.788939	-0.819946	-1.551084
H	3.704358	-0.497696	-2.072329
C	2.138962	-1.994870	0.673139
H	1.323311	-2.645045	0.323683
H	1.613614	0.912759	-2.011773
H	0.811634	-0.923951	1.976708
C	3.172129	-1.831028	-0.451633
H	3.340940	-2.810263	-0.921128
H	4.135202	-1.554500	-0.010058
C	3.573820	0.932745	0.889056
H	4.060433	1.766523	1.414443
H	4.379889	0.240497	0.625155
H	2.188636	-1.340479	-2.310190
H	2.610228	-2.527243	1.513827
H	3.678571	1.822302	-1.085774
H	2.021707	1.061750	2.367520
B	0.862390	0.091231	-0.044652
C	-0.644988	0.667275	-0.070769
C	-1.791816	-0.424165	-0.019296
C	-1.878932	-1.218416	-1.336450
H	-2.764103	-1.862290	-1.296189
C	-1.647104	-1.389193	1.170098
H	-1.570182	-0.789571	2.085593
H	-0.711432	-1.950778	1.078311
C	-0.918060	1.548412	-1.318822
H	-0.618433	0.989896	-2.216255
C	-0.623753	1.557536	1.214169
H	-0.407254	0.932478	2.089042
H	0.250062	2.221959	1.122503
H	-2.046395	-0.530245	-2.170113
H	-2.002498	1.704319	-1.402696
O	-3.015823	0.297374	0.214709
C	-4.139397	0.151465	-0.609639
H	-4.560491	-0.866321	-0.589209
H	-4.897737	0.839848	-0.217266
H	-3.941843	0.425909	-1.657450
C	-0.667326	-2.086532	-1.636938
H	-0.759657	-2.538872	-2.631986
H	0.261285	-1.502868	-1.643510
H	-0.554165	-2.899527	-0.909946
C	-2.808418	-2.366739	1.322839
H	-3.764540	-1.833204	1.389478
H	-2.864562	-3.077051	0.487616
H	-2.689425	-2.954086	2.241818
C	-1.841230	2.433897	1.531596
H	-1.542335	3.243406	2.211611
H	-2.275343	2.885701	0.632052
H	-2.634891	1.855683	2.012732
C	-0.246299	2.919550	-1.382096
H	-0.522431	3.419323	-2.319974
H	-0.556805	3.575553	-0.560098
H	0.845687	2.853538	-1.362259

58

D_Et_conf_9 Eopt -845.396112

C	2.307457	-1.898179	0.582704
C	1.468953	-0.710712	1.109809
C	1.902086	0.569540	-1.090252
C	2.753991	-0.612822	-1.606298
H	1.630385	-2.679177	0.210652
H	3.591259	-0.219117	-2.203867
C	2.355622	0.317846	1.871848
H	2.901696	-0.214468	2.666016
C	2.749143	1.613496	-0.308096
H	2.115889	2.481318	-0.074468
H	0.763804	-1.104639	1.851163
H	1.497490	1.078651	-1.975765
C	3.351081	1.106611	1.009218
H	3.706355	1.971278	1.587193
H	4.241967	0.503864	0.809861
C	3.306900	-1.562789	-0.533642
H	3.615144	-2.498753	-1.020430
H	4.222980	-1.148516	-0.101889
H	1.698129	1.033707	2.383836
H	3.551446	1.989060	-0.961917
H	2.849059	-2.355816	1.425336
H	2.136871	-1.197879	-2.302942
B	0.771910	0.095151	-0.077418
C	-0.752122	0.649086	-0.080487
C	-1.872058	-0.463338	-0.016212
C	-1.908354	-1.287449	-1.321311
H	-2.586310	-2.138212	-1.178673

C -1.824693 -1.360819 1.255171
H -2.862256 -1.450447 1.596683
H -1.311559 -0.821469 2.057619
C -1.055285 1.526520 -1.330964
H -0.735389 0.985058 -2.231534
C -0.739328 1.544636 1.199168
H -0.462163 0.939946 2.070794
H 0.090026 2.258287 1.080765
H -2.368211 -0.660841 -2.095592
H -2.141748 1.639422 -1.415866
O -3.110760 0.271202 0.073259
C -4.307360 -0.404117 -0.201718
H -4.461166 -0.562273 -1.280599
H -4.376212 -1.382512 0.301265
H -5.123294 0.226202 0.171997
C -0.565517 -1.785500 -1.839025
H 0.046811 -2.242930 -1.054310
H -0.707956 -2.544070 -2.619007
H 0.008226 -0.966790 -2.288464
C -1.268509 -2.780687 1.166334
H -0.195786 -2.812963 0.961795
H -1.428695 -3.287113 2.126874
H -1.778208 -3.374188 0.397100
C -1.999728 2.340494 1.552587
H -2.463008 2.799378 0.671718
H -2.757129 1.699775 2.013994
H -1.741174 3.137954 2.262443
C -0.444799 2.927225 -1.385000
H -0.713020 3.403683 -2.337294
H -0.820050 3.573630 -0.582452
H 0.647443 2.921338 -1.324018
46
D_Me_conf_2 Eopt -688.349686
C 2.062147 1.500659 -1.218631
C 1.367190 0.113355 -1.286291
C 1.438393 -0.000103 1.314502
C 2.146563 1.380790 1.332163
H 1.698705 2.128548 -2.043538
H 3.237604 1.252085 1.404654
C 2.404390 -1.047010 -1.338604
H 3.028482 -0.930100 -2.237206
C 2.457175 -1.173891 1.196173
H 1.915828 -2.134216 1.223233
H 0.767919 0.050555 -2.202416
H 0.892526 -0.142599 2.254647
C 3.284409 -1.108071 -0.089008
H 3.947947 -1.981820 -0.146129
H 3.941474 -0.226799 -0.058295
C 1.819012 2.234345 0.103750
H 0.754162 2.517363 0.154974
H 2.391855 3.171981 0.127943
H 1.874424 -2.006690 -1.449751
H 3.118657 -1.165195 2.075371
H 3.146292 1.391299 -1.377046
H 1.847130 1.930236 2.234817
B 0.546755 -0.211135 0.026807
C -0.910145 -0.879429 0.035634
C -1.975282 0.269059 -0.052226
C -1.937660 1.153588 1.200666
H -2.548895 2.053795 1.060882
H -2.311899 0.617020 2.079474
C -1.771006 1.151563 -1.288236
H -0.833570 1.718293 -1.218301
H -2.584162 1.882203 -1.369794
C -1.144960 -1.704782 1.312129
H -1.013444 -1.120768 2.229173
H -0.438622 -2.545160 1.355144
H -1.752703 0.556060 -2.207113
C -1.064144 -1.842915 -1.156273
H -0.865295 -1.370538 -2.124618
H -0.363591 -2.682519 -1.050422
H -2.078923 -2.258901 -1.181706
H -0.911934 1.481756 1.411659
H -2.160951 -2.120917 1.315755
O -3.237857 -0.396409 -0.158027
C -4.398253 0.367704 0.036927
H -5.247976 -0.270225 -0.232711
H -4.519612 0.678139 1.086328
H -4.429326 1.264893 -0.600878
46
D_Me_conf_3 Eopt -688.348337
C 2.468388 0.907039 -1.358685
C 1.346508 -0.183171 -1.295838
C 1.424433 -0.063741 1.310445
C 2.539973 1.031059 1.212328
H 2.305101 1.542714 -2.238654
H 3.525858 0.561305 1.348719
C 1.932905 -1.608641 -1.204324
H 2.528303 -1.820262 -2.105798
C 2.040080 -1.479423 1.329537
H 1.242645 -2.222092 1.488260
H 0.750240 -0.118651 -2.213477
H 0.872623 0.080262 2.247191
C 2.791237 -1.819728 0.042678
H 3.142145 -2.860215 0.080890
H 3.695795 -1.197115 -0.028432
C 2.524371 1.806410 -0.113450
H 1.639370 2.465694 -0.119467
H 3.397460 2.470729 -0.170082
H 1.106653 -2.339412 -1.199120
H 2.720524 -1.568388 2.190459
H 3.445495 0.421951 -1.503947
H 2.422821 1.745942 2.037046
B 0.529101 0.150529 0.018538
C -0.926806 0.818025 0.037659
C -1.997103 -0.321656 -0.035883
C -2.104908 -0.911670 -1.444990
H -2.731460 -1.812326 -1.439493
H -2.540689 -0.192134 -2.147378
C -1.649982 -1.451965 0.939333
H -0.763288 -1.996503 0.582407
H -2.466286 -2.181100 1.001270
C -1.093237 1.788459 -1.144353
H -0.827784 1.339361 -2.108212
H -0.454108 2.670966 -1.003238
H -1.445476 -1.074014 1.947465
C -1.112855 1.635743 1.327863
H -1.192373 1.007037 2.222047
H -0.256360 2.310769 1.473302
H -2.020405 2.248264 1.270293
H -1.113393 -1.201469 -1.811652
H -2.132251 2.141935 -1.203609
O -3.230919 0.290799 0.347548
C -4.414565 -0.427198 0.118665
H -5.220827 0.121372 0.619219
H -4.657315 -0.495883 -0.952979
H -4.381810 -1.445016 0.537974
46
D_Me_conf_4 Eopt -688.348188
C 2.691462 1.249517 -0.939215
C 1.797658 0.010036 -1.234231
C 1.108695 -0.051257 1.274834
C 2.018408 1.188503 1.523770
H 2.702121 1.907710 -1.818128
H 3.002237 0.867391 1.898792
C 2.562377 -1.321753 -1.028061
H 3.418692 -1.362139 -1.718391
C 1.894747 -1.382174 1.414961
H 1.204498 -2.230982 1.275171
H 1.479506 0.042563 -2.283284
H 0.321752 -0.062720 2.035509
C 3.039602 -1.511066 0.411104
H 3.517074 -2.494663 0.518920
H 3.816657 -0.767552 0.642031
C 2.209702 2.053064 0.273462
H 1.239981 2.517943 0.021014
H 2.902991 2.878933 0.484490
H 1.903576 -2.163511 -1.299450
H 2.281558 -1.463658 2.441905
H 3.734884 0.937219 -0.776722

H 1.576138 1.806468 2.316732
B 0.573442 -0.013839 -0.221135
C -0.918551 -0.037139 -0.783809
C -2.041869 -0.021121 0.291868
C -1.998575 -1.288175 1.157674
H -2.583523 -1.168230 2.077562
H -2.414031 -2.133634 0.595895
C -1.965421 1.258771 1.139492
H -0.931881 1.507508 1.403116
H -2.532218 1.162439 2.073264
C -1.042363 -1.301221 -1.661299
H -0.829490 -2.223007 -1.103190
H -0.341770 -1.254528 -2.504172
H -2.380002 2.101568 0.573156
C -1.061609 1.198256 -1.700448
H -0.842974 2.138418 -1.175954
H -0.374634 1.127460 -2.551737
H -2.084123 1.260316 -2.092477
H -0.975549 -1.547085 1.446068
H -2.058636 -1.379868 -2.068329
O -3.278009 -0.012957 -0.440112
C -4.460345 -0.011241 0.312706
H -5.293860 0.027733 -0.398563
H -4.571442 -0.922752 0.921057
H -4.534174 0.865509 0.975454
58
D'_Et_conf_1 Eopt -845.401665
C 3.194778 0.422606 -1.057279
C 1.804524 -0.267100 -1.235963
C 1.569000 -0.342679 1.354421
C 2.951288 0.364429 1.495356
H 3.365605 1.118328 -1.889620
H 3.736884 -0.381325 1.693151
C 1.949304 -1.810935 -1.231844
H 2.579551 -2.117338 -2.080760
C 1.740521 -1.880822 1.293227
H 0.745005 -2.346367 1.269407
H 1.383885 0.029552 -2.205842
H 0.954408 -0.117628 2.232955
C 2.537753 -2.344213 0.074462
H 2.565888 -3.442450 0.044564
H 3.583530 -2.016694 0.168715
C 3.333343 1.191622 0.262879
H 2.670687 2.072212 0.224011
H 4.356551 1.579118 0.366345
H 0.963141 -2.276544 -1.389819
H 2.236913 -2.224451 2.214224
H 3.995016 -0.329200 -1.124928
H 2.934906 1.028247 2.370269
B 0.883142 0.129350 -0.001930
C -0.504755 0.953622 -0.087945
C -1.644587 -0.144380 -0.124242
C -3.028986 0.477981 0.134519
H -3.074517 0.800890 1.181717
C -1.620198 -0.895708 -1.480221
H -0.607901 -0.848698 -1.895253
C -0.567877 1.854097 -1.339752
H -0.572590 1.238659 -2.246965
H -2.257432 -0.352783 -2.192311
C -0.588882 1.814702 1.208011
H -0.906394 1.152400 2.024882
H 0.431029 2.129288 1.480485
H -3.073831 1.392271 -0.468361
H -1.518540 2.406423 -1.346332
O -1.237754 -1.042124 0.917308
C -2.158215 -1.750193 1.702313
H -2.723420 -2.502852 1.134323
H -1.569733 -2.269857 2.469113
H -2.868554 -1.086208 2.216792
C 0.577677 2.859410 -1.466165
H 0.727729 3.439777 -0.546875
H 1.523695 2.358292 -1.701079
H 0.375019 3.570595 -2.277275
C -4.277592 -0.337939 -0.204874
H -4.314855 -0.592003 -1.271424
H -4.360451 -1.267129 0.368153
H -5.168069 0.264684 0.015754
C -2.025459 -2.366542 -1.434838
H -1.378814 -2.922901 -0.744334
H -3.064659 -2.515764 -1.123098
H -1.911351 -2.809305 -2.432535
C -1.452605 3.080244 1.212024
H -1.110958 3.814555 0.472360
H -2.511754 2.885599 1.018438
H -1.383482 3.559634 2.197797
58
D'_Et_conf_10 Eopt -845.399447
C -2.073160 -1.923801 -1.057246
C -1.739770 -0.428508 -1.279449
C -1.718844 -0.109765 1.303034
C -2.040105 -1.617802 1.457849
H -1.139872 -2.502718 -1.067907
H -2.620881 -1.772479 2.380243
C -3.049046 0.411626 -1.377540
H -3.919641 -0.258843 -1.446187
C -3.036452 0.723917 1.174733
H -3.043106 1.512135 1.939298
H -1.191473 -0.353026 -2.224949
H -1.181309 0.216765 2.203508
C -3.236013 1.375581 -0.201153
H -2.502195 2.189383 -0.313911
H -4.227734 1.846884 -0.248838
C -2.802850 -2.183479 0.260027
H -2.952903 -3.263767 0.395302
H -3.809081 -1.740833 0.220138
H -3.042105 0.995558 -2.307774
H -3.900907 0.081585 1.399161
H -2.686567 -2.284393 -1.897788
H -1.104297 -2.186337 1.577733
B -0.883895 0.083344 -0.035910
C 0.563094 0.805224 -0.134552
C 1.607172 -0.368370 0.007364
C 2.992478 0.005821 -0.553020
H 2.901145 0.067346 -1.645184
C 1.719647 -0.923860 1.446968
H 2.135316 -1.933315 1.343043
C 0.766442 1.852741 0.976515
H 0.704693 1.361989 1.952198
H 0.703908 -1.059307 1.834162
C 0.621595 1.477142 -1.537948
H 0.763509 0.685534 -2.285766
H -0.378656 1.887561 -1.747395
H 3.218750 1.021749 -0.208817
H 1.783500 2.266109 0.908265
O 0.974570 -1.394814 -0.780594
C 1.689704 -2.518680 -1.215307
H 2.462684 -2.254023 -1.952309
H 2.155470 -3.078750 -0.389914
H 0.965287 -3.178815 -1.708101
C -0.236263 3.006297 0.984932
H -1.222614 2.667623 1.322347
H 0.091824 3.788841 1.681199
H -0.359832 3.470936 -0.001354
C 4.188072 -0.879945 -0.189667
H 4.383237 -0.883811 0.888050
H 4.073658 -1.919658 -0.509879
H 5.087589 -0.484691 -0.679437
C 2.572822 -0.151755 2.473012
H 3.301969 -0.815480 2.952400
H 3.131729 0.675964 2.021223
H 1.954475 0.274634 3.270387
C 1.622643 2.608072 -1.800266
H 1.565645 3.400209 -1.043352
H 2.660941 2.266558 -1.845921
H 1.393758 3.071933 -2.769288
58
D'_Et_conf_11 Eopt -845.404035
C -2.380840 0.793363 1.557916
C -1.389284 -0.365181 1.309927
C -1.740894 -0.034210 -1.233643

C	-2.948013	0.888985	-0.940688
H	-1.836496	1.749080	1.571362
H	-3.769989	0.640570	-1.633073
C	-2.132198	-1.723956	1.239926
H	-2.904281	-1.750332	2.026868
C	-2.203728	-1.494064	-1.387040
H	-1.356876	-2.097127	-1.741113
H	-0.717830	-0.409019	2.181922
H	-1.328531	0.274559	-2.207644
C	-2.809469	-2.098420	-0.104679
H	-2.820840	-3.194512	-0.198789
H	-3.865601	-1.807460	-0.066674
C	-3.485406	0.864935	0.501179
H	-4.093528	1.765585	0.669472
H	-4.168248	0.018677	0.644145
H	-1.427892	-2.518146	1.511736
H	-2.961790	-1.562987	-2.184014
H	-2.841685	0.687207	2.553072
H	-2.672844	1.922849	-1.178295
B	-0.620987	0.006343	-0.061192
C	0.673525	1.056714	-0.044023
C	1.656354	-0.183045	-0.069717
C	2.741943	-0.185684	-1.154747
H	2.427946	0.442641	-1.989321
C	2.227060	-0.520367	1.318540
H	1.394629	-0.752553	1.993209
C	0.828940	1.969477	1.184043
H	-0.026106	2.656154	1.211622
H	2.659139	0.408845	1.697831
C	0.774400	1.924650	-1.314750
H	1.805439	2.281471	-1.456522
H	0.544884	1.311942	-2.195864
H	3.624490	0.305165	-0.717654
H	0.750489	1.384054	2.107856
O	0.579084	-1.146429	-0.443593
C	0.649969	-2.503099	-0.012193
H	-0.244434	-3.012733	-0.372166
H	1.529940	-2.980187	-0.447775
H	0.688722	-2.568985	1.079353
C	2.104486	2.821420	1.205197
H	2.057601	3.626381	0.461245
H	2.244501	3.292370	2.186949
H	3.008024	2.237415	0.983549
C	3.137994	-1.531466	-1.764167
H	2.283637	-1.982090	-2.285231
H	3.923867	-1.364212	-2.511035
H	3.520984	-2.254290	-1.037494
C	3.322515	-1.580573	1.451671
H	3.014186	-2.583603	1.140910
H	4.217574	-1.305318	0.880573
H	3.618712	-1.641400	2.506391
C	-0.140195	3.150209	-1.309636
H	0.275498	3.962921	-0.701007
H	-0.275356	3.539071	-2.326894
H	-1.128634	2.919597	-0.901493

58

D'_Et_conf_12 Eopt -845.408702

C	-2.439697	0.774695	1.690714
C	-1.394105	-0.337175	1.377703
C	-1.907317	-0.054392	-1.158111
C	-2.993138	1.003479	-0.796858
H	-2.095666	1.373238	2.546024
H	-3.978566	0.515833	-0.741490
C	-2.052665	-1.730501	1.342732
H	-2.567136	-1.925779	2.297939
C	-2.554671	-1.454974	-1.177814
H	-1.840196	-2.186947	-1.575818
H	-0.672306	-0.346334	2.203888
H	-1.536722	0.150967	-2.173500
C	-3.065208	-1.895066	0.201752
H	-3.409886	-2.938516	0.156706
H	-3.957493	-1.299351	0.447159
C	-2.731293	1.727515	0.525045
H	-1.869911	2.396610	0.390936
H	-3.585464	2.373067	0.776892
H	-1.266802	-2.496673	1.265794
H	-3.403195	-1.459865	-1.881351
H	-3.385232	0.309123	2.013941
H	-3.084547	1.747390	-1.600113
B	-0.712395	-0.052851	-0.062704
C	0.666163	0.860971	-0.178979
C	1.556506	-0.433930	-0.098192
C	2.679974	-0.563097	-1.135652
H	2.224135	-0.572402	-2.132379
C	2.035260	-0.866806	1.294362
H	2.298163	-1.928715	1.221885
C	0.900077	1.877241	0.946158
H	0.029728	2.544601	1.005236
H	1.196283	-0.810909	1.993544
C	0.810089	1.537726	-1.555638
H	1.849017	1.862414	-1.713463
H	0.602134	0.802015	-2.344161
H	3.265877	0.364181	-1.081099
H	0.919097	1.355719	1.910934
O	0.417613	-1.311142	-0.455744
C	0.543653	-2.714995	-0.629137
H	-0.452509	-3.156651	-0.596390
H	0.995981	-2.926746	-1.605419
H	1.146778	-3.160834	0.169054
C	2.151753	2.747379	0.788934
H	2.009807	3.513307	0.016120
H	2.390431	3.267267	1.726045
H	3.029392	2.155198	0.500692
C	3.643780	-1.745790	-1.009794
H	4.221860	-1.713224	-0.079194
H	3.138826	-2.716673	-1.054705
H	4.360320	-1.712608	-1.840031
C	3.243856	-0.132025	1.881622
H	3.707371	-0.756977	2.654832
H	4.012019	0.082680	1.128858
H	2.964961	0.815371	2.351631
C	-0.099083	2.742807	-1.782686
H	-1.156199	2.465400	-1.734750
H	0.072406	3.537575	-1.045321
H	0.084712	3.172268	-2.776417

58

D'_Et_conf_13 Eopt -845.400486

C	3.048098	0.991089	-1.084885
C	1.805695	0.077578	-1.282597
C	1.667968	-0.170241	1.300386
C	2.885832	0.777857	1.425854
H	2.746507	2.047707	-1.071821
H	3.446976	0.535566	2.341600
C	2.259924	-1.410545	-1.399184
H	3.351347	-1.448266	-1.534559
C	2.124675	-1.654447	1.169896
H	1.598809	-2.254267	1.923986
H	1.319923	0.378956	-2.220609
H	1.061546	-0.067249	2.207112
C	1.856421	-2.285208	-0.204486
H	0.777265	-2.482853	-0.271053
H	2.362301	-3.258870	-0.272354
C	3.806489	0.701566	0.209102
H	4.633796	1.416592	0.317940
H	4.268520	-0.295294	0.157520
H	1.829901	-1.850345	-2.309556
H	3.195933	-1.733992	1.410286
H	3.716528	0.869517	-1.951026
H	2.534308	1.817457	1.541707
B	0.873591	0.255109	-0.004426
C	-0.632587	0.850056	0.001897
C	-1.571095	-0.426192	-0.035805
C	-3.034556	-0.207003	0.396075
H	-3.468629	-1.198438	0.568607
C	-1.509464	-1.103168	-1.420149
H	-0.460247	-1.144691	-1.742089
C	-0.877466	1.788354	-1.199618
H	-0.801791	1.224675	-2.136318
H	-2.024123	-0.471833	-2.153374
C	-0.820701	1.617532	1.341641

H -1.011573 0.873707 2.127308
H 0.133377 2.086264 1.625135
H -3.045743 0.289953 1.373150
H -1.900910 2.174283 -1.162207
O -0.925078 -1.308608 0.906337
C -1.625725 -2.144618 1.787065
H -0.864430 -2.731337 2.316164
H -2.200747 -1.578073 2.535752
H -2.304769 -2.846739 1.281087
C 0.085746 2.977584 -1.284852
H -0.365351 3.803014 -1.849940
H 0.357961 3.367276 -0.294591
H 1.011384 2.704181 -1.800492
C -3.975195 0.507827 -0.571625
H -4.986146 0.521618 -0.144510
H -3.693964 1.545904 -0.771577
H -4.038115 -0.015692 -1.533898
C -2.093495 -2.510515 -1.465822
H -3.154365 -2.528156 -1.183243
H -2.017615 -2.916374 -2.482181
H -1.546731 -3.186451 -0.796483
C -1.890341 2.710991 1.394769
H -2.885029 2.354870 1.106369
H -1.964482 3.106424 2.416469
H -1.640285 3.553288 0.737156
58
D'_Et_conf_14 Eopt -845.398421
C 1.436880 -2.010627 1.028393
C 1.477910 -0.474510 1.245555
C 1.717281 -0.151568 -1.307032
C 1.822346 -1.686639 -1.495524
H 0.384424 -2.318631 0.980850
H 2.561888 -1.906999 -2.281689
C 2.925739 0.036048 1.456732
H 3.410410 -0.570060 2.238951
C 3.092316 0.522275 -1.060094
H 2.947104 1.611453 -0.995167
H 0.904744 -0.259623 2.157713
H 1.319803 0.263774 -2.244091
C 3.820447 0.058670 0.205737
H 4.666607 0.735404 0.390991
H 4.268411 -0.924599 0.032428
C 2.157497 -2.500629 -0.234100
H 1.878861 -3.548991 -0.413153
H 3.238676 -2.513897 -0.063955
H 2.876099 1.057653 1.855518
H 3.737998 0.357636 -1.936632
H 1.873022 -2.508186 1.908949
H 0.856875 -2.046515 -1.882670
B 0.816284 0.178268 -0.042636
C -0.554279 1.047514 -0.072349
C -1.760549 0.015623 -0.047960
C -3.097155 0.736308 0.235696
H -3.112779 1.033775 1.291008
C -1.803692 -0.760386 -1.388433
H -0.792868 -0.783039 -1.811096
C -0.667800 1.915819 -1.349463
H -0.544827 1.283694 -2.236263
H -2.408492 -0.186802 -2.104673
C -0.646483 1.942600 1.188725
H -1.410709 2.712738 1.010253
H -1.019454 1.327410 2.016886
H -3.094381 1.671520 -0.334152
H -1.682664 2.331831 -1.416620
O -1.403060 -0.875821 1.012295
C -2.361896 -1.532970 1.795701
H -2.978039 -2.241516 1.224996
H -1.801747 -2.098262 2.551469
H -3.025517 -0.832254 2.324028
C 0.302149 3.088955 -1.470475
H 0.150021 3.596681 -2.431560
H 0.147042 3.832247 -0.678374
H 1.348973 2.765249 -1.431800
C -4.399590 0.011548 -0.107495
H -4.458485 -0.229285 -1.176329
H -4.541118 -0.915944 0.457339
H -5.246833 0.670012 0.123946
C -2.300774 -2.201121 -1.320967
H -2.224980 -2.661391 -2.314290
H -1.683273 -2.790332 -0.630643
H -3.344653 -2.278425 -0.998206
C 0.661270 2.621907 1.638377
H 1.079996 2.129712 2.523562
H 1.436958 2.601564 0.862169
H 0.496280 3.674736 1.898974
58
D'_Et_conf_15 Eopt -845.408364
C -2.504372 -1.332892 -1.416466
C -1.739215 0.001318 -1.305347
C -1.489965 -0.343864 1.250760
C -2.200006 -1.706798 1.150120
H -1.821096 -2.072249 -1.859476
H -2.828086 -1.870220 2.041516
C -2.660531 1.210090 -1.047620
H -3.416364 1.287939 -1.846395
C -2.469461 0.823086 1.497557
H -1.880390 1.706454 1.781639
H -1.258500 0.168190 -2.281092
H -0.851017 -0.388882 2.144423
C -3.375559 1.193954 0.311633
H -3.815550 2.185239 0.495913
H -4.225486 0.502779 0.265979
C -3.078526 -1.897344 -0.100935
H -3.280615 -2.970739 -0.234224
H -4.058673 -1.443971 0.085684
H -2.055505 2.127276 -1.116606
H -3.103797 0.594485 2.370040
H -3.330462 -1.233787 -2.140508
H -1.433394 -2.494898 1.185644
B -0.662003 -0.064468 -0.110446
C 0.722528 0.859606 -0.120431
C 1.581320 -0.437943 0.052890
C 2.793515 -0.598705 -0.869766
H 2.435374 -0.600767 -1.906340
C 1.926606 -0.846727 1.490607
H 2.188448 -1.911265 1.463759
C 0.890551 1.890099 1.003865
H 0.556232 1.447795 1.952737
H 1.031498 -0.769256 2.112781
C 0.904001 1.526968 -1.502106
H 0.829795 0.763213 -2.286096
H 0.024142 2.164784 -1.654038
H 3.394536 0.310509 -0.754703
H 1.952436 2.138908 1.144391
O 0.465822 -1.301680 -0.405570
C 0.534747 -2.715023 -0.512970
H 1.137566 -2.986484 -1.387097
H 0.955604 -3.166406 0.392820
H -0.480812 -3.088711 -0.654925
C 0.137591 3.201321 0.771984
H 0.143684 3.814889 1.682038
H 0.597335 3.794887 -0.028465
H -0.908384 3.028969 0.491060
C 3.709084 -1.802360 -0.634023
H 3.180671 -2.760928 -0.677040
H 4.486575 -1.821439 -1.407647
H 4.215970 -1.744765 0.336722
C 3.085365 -0.089256 2.151007
H 2.743584 0.816945 2.659034
H 3.564053 -0.726233 2.904622
H 3.859603 0.205167 1.431797
C 2.161150 2.380052 -1.746693
H 2.562288 2.810849 -0.819825
H 2.967715 1.806914 -2.216707
H 1.931520 3.217094 -2.419004
58
D'_Et_conf_2 Eopt -845.406687
C 2.481538 -1.048589 1.549837
C 1.788975 0.278094 1.175862
C 1.325555 -0.576696 -1.223649

C	1.898809	-1.956556	-0.836790
H	1.820858	-1.589884	2.240688
H	2.421102	-2.406019	-1.697530
C	2.779623	1.296807	0.561377
H	3.566265	1.541333	1.293663
C	2.385183	0.391304	-1.782436
H	1.866614	1.284297	-2.166879
H	1.414192	0.718362	2.113950
H	0.602489	-0.757068	-2.033923
C	3.425228	0.856835	-0.756257
H	3.989451	1.703152	-1.174441
H	4.166167	0.070478	-0.575202
C	2.855111	-1.976958	0.372678
H	2.922599	-3.009099	0.747357
H	3.867010	-1.735491	0.029934
H	2.248311	2.236939	0.355063
H	2.898260	-0.060961	-2.647308
H	3.395522	-0.840997	2.131135
H	1.048623	-2.622848	-0.627617
B	0.620894	0.052633	0.091051
C	-0.696018	1.053205	-0.015866
C	-1.637616	-0.215916	-0.021101
C	-2.854360	-0.157598	0.911732
H	-2.518090	-0.012777	1.943805
C	-2.045765	-0.726293	-1.415757
H	-1.405644	-0.283676	-2.180562
C	-0.766466	1.962385	-1.246126
H	0.032926	2.711776	-1.154690
H	-3.060130	-0.357416	-1.617654
C	-0.947356	1.865200	1.276230
H	-2.004162	2.159763	1.329574
H	-0.777195	1.212380	2.144548
H	-3.374611	0.768749	0.632819
H	-0.513618	1.398685	-2.152192
O	-0.564753	-1.093738	0.505530
C	-0.755223	-2.141174	1.438344
H	0.206976	-2.641759	1.567310
H	-1.099238	-1.755958	2.407246
H	-1.472244	-2.867860	1.043901
C	-2.099825	2.682114	-1.447978
H	-2.329138	3.347977	-0.604955
H	-2.084375	3.294998	-2.358650
H	-2.928676	1.967064	-1.543948
C	-3.884062	-1.287804	0.875563
H	-4.709256	-1.029014	1.550827
H	-4.313798	-1.424451	-0.123821
H	-3.481448	-2.249677	1.211032
C	-1.998671	-2.241953	-1.597665
H	-0.976460	-2.614635	-1.458199
H	-2.654377	-2.777939	-0.903161
H	-2.311938	-2.499481	-2.616822
C	-0.130498	3.139243	1.476367
H	-0.452153	3.638801	2.399993
H	0.940370	2.940382	1.573097
H	-0.272594	3.854698	0.655942

58

D'_Et_conf_3 Eopt -845.408654

C	2.394782	0.434991	-1.754426
C	1.314687	-0.578289	-1.277387
C	1.812504	0.051301	1.196669
C	2.913815	1.040301	0.696029
H	2.067209	0.916132	-2.686862
H	3.897165	0.551244	0.765908
C	1.927019	-1.966419	-0.999938
H	2.367257	-2.378020	-1.922780
C	2.472998	-1.331016	1.404316
H	1.772779	-2.033666	1.871789
H	0.589439	-0.708328	-2.091238
H	1.455959	0.400998	2.177925
C	2.990209	-1.941910	0.099231
H	3.359812	-2.960253	0.286813
H	3.859406	-1.367262	-0.254028
C	2.714563	1.541605	-0.738112
H	1.887056	2.263120	-0.739742
H	3.605551	2.098897	-1.062565
H	1.122757	-2.659380	-0.703144
H	3.311837	-1.229702	2.112110
H	3.323055	-0.101328	-2.008661
H	2.977577	1.908890	1.365154
B	0.629085	-0.034299	0.085475
C	-0.657004	1.013069	0.009518
C	-1.617649	-0.226654	-0.019032
C	-2.850553	-0.133896	0.889335
H	-2.536912	-0.017380	1.932615
C	-2.024579	-0.728822	-1.417614
H	-1.383575	-0.295294	-2.186323
C	-0.762235	1.950332	-1.199432
H	-0.003892	2.735626	-1.095589
H	-3.034996	-0.353570	-1.620997
C	-0.838859	1.802274	1.324067
H	-1.873236	2.158846	1.428349
H	-0.670574	1.124806	2.173430
H	-3.311331	0.824297	0.612878
H	-0.489575	1.418058	-2.119569
O	-0.576584	-1.180591	0.484908
C	-0.720542	-1.871682	1.717797
H	-1.761564	-2.160776	1.866765
H	-0.118777	-2.782634	1.655958
H	-0.382134	-1.255897	2.560544
C	-2.121496	2.627912	-1.380453
H	-2.926093	1.895117	-1.524950
H	-2.380917	3.242127	-0.506980
H	-2.115915	3.288865	-2.256812
C	-3.938763	-1.205728	0.800396
H	-4.345358	-1.298233	-0.213702
H	-3.602801	-2.200100	1.115783
H	-4.767412	-0.918581	1.459424
C	-1.992804	-2.248024	-1.572537
H	-2.504571	-2.767041	-0.753880
H	-2.478904	-2.539284	-2.511722
H	-0.958367	-2.610045	-1.597452
C	0.079747	3.010616	1.483193
H	0.002339	3.416522	2.500217
H	1.128411	2.751603	1.305545
H	-0.188494	3.818084	0.789419

58

D'_Et_conf_4 Eopt -845.400308

C	1.426036	-2.050191	1.197384
C	1.493621	-0.502967	1.285190
C	1.633380	-0.419734	-1.308856
C	1.568829	-1.970069	-1.329654
H	0.372670	-2.362563	1.196453
H	2.129830	-2.340252	-2.201406
C	2.980862	-0.037809	1.368870
H	3.637508	-0.909822	1.506198
C	3.113132	0.065945	-1.193276
H	3.327230	0.768803	-2.009539
H	0.958769	-0.202143	2.194394
H	1.220287	-0.057107	-2.258832
C	3.439799	0.752962	0.138655
H	2.938642	1.734403	0.155950
H	4.518237	0.955831	0.199279
C	2.113814	-2.603387	-0.050352
H	1.982049	-3.693557	-0.094403
H	3.198175	-2.431326	0.013346
H	3.124047	0.585545	2.261692
H	3.794605	-0.785406	-1.341077
H	1.891057	-2.478941	2.098748
H	0.527304	-2.300495	-1.466030
B	0.822513	0.084702	-0.033603
C	-0.472258	1.054425	-0.092120
C	-1.736993	0.085575	-0.066552
C	-3.031696	0.880137	0.209760
H	-3.026303	1.212046	1.254111
C	-1.845376	-0.700340	-1.398125
H	-0.850488	-0.781035	-1.846451
C	-0.506012	1.888502	-1.394701
H	-0.544895	1.210011	-2.253268
H	-2.439107	-0.106963	-2.107173
C	-0.554047	1.968136	1.159600

H -1.316933 2.739776 0.975980
H -0.926677 1.356507 1.993016
H -2.977010 1.794002 -0.393612
H -1.436009 2.474644 -1.431174
O -1.412971 -0.821496 0.993827
C -2.394997 -1.400243 1.810134
H -3.007200 -0.646925 2.327858
H -3.060568 -2.086152 1.267501
H -1.856898 -1.977026 2.573034
C 0.658408 2.851184 -1.630477
H 0.644706 3.204240 -2.669704
H 0.600186 3.737618 -0.989547
H 1.630183 2.368865 -1.460306
C -4.375478 0.218960 -0.101273
H -5.181549 0.930801 0.118937
H -4.461423 -0.049903 -1.161312
H -4.562374 -0.681146 0.493838
C -2.414039 -2.112638 -1.297132
H -3.453334 -2.132267 -0.952583
H -2.382367 -2.591210 -2.284266
H -1.813784 -2.721870 -0.609107
C 0.715758 2.670301 1.648100
H 1.174509 3.312478 0.890718
H 0.467713 3.308380 2.507193
H 1.473959 1.958123 1.987911
58
D'_Et_conf_5 Eopt -845.397292
C -2.072670 -1.789539 -0.958112
C -1.735365 -0.279837 -1.180089
C -1.603289 0.043655 1.393644
C -1.986752 -1.452592 1.609983
H -1.563362 -2.381160 -1.729270
H -3.055288 -1.523871 1.861080
C -3.045674 0.545946 -1.205453
H -3.694193 0.165037 -2.009695
C -2.884531 0.915759 1.287122
H -2.610649 1.974658 1.157132
H -1.239295 -0.173993 -2.151853
H -1.041424 0.397030 2.267583
C -3.789507 0.503589 0.127822
H -4.665177 1.166319 0.088416
H -4.180987 -0.508998 0.304860
C -1.670987 -2.358612 0.411283
H -0.586765 -2.533579 0.392703
H -2.144559 -3.340119 0.555357
H -2.827632 1.594257 -1.451883
H -3.435750 0.853805 2.237890
H -3.151155 -1.942646 -1.116114
H -1.448962 -1.841493 2.485278
B -0.814523 0.205867 0.020733
C 0.696438 0.781568 -0.123027
C 1.631525 -0.490360 -0.159988
C 3.044419 -0.273501 -0.754742
H 3.579476 -1.226591 -0.629985
C 1.678225 -1.221022 1.210248
H 1.719313 -2.295139 0.994471
C 1.093767 1.659969 1.086080
H 0.919407 1.093855 2.008141
H 0.722616 -1.061345 1.725046
C 0.853117 1.558131 -1.454722
H 1.786742 2.133723 -1.415848
H 0.979829 0.825478 -2.260588
H 2.911830 -0.147591 -1.837380
H 2.169951 1.860903 1.056687
O 0.926115 -1.356409 -1.075714
C 1.534209 -2.530931 -1.541119
H 2.012666 -3.119949 -0.744048
H 0.738867 -3.144677 -1.982260
H 2.284945 -2.329707 -2.320246
C 0.377563 3.001436 1.217471
H 0.687785 3.498362 2.145558
H 0.617159 3.677597 0.387167
H -0.712787 2.883353 1.254863
C 3.967759 0.844487 -0.265173
H 3.598890 1.839502 -0.535540
H 4.132978 0.831225 0.816195
H 4.945830 0.720847 -0.748314
C 2.823073 -0.925664 2.178140
H 2.860039 0.122234 2.494294
H 2.693997 -1.537933 3.079811
H 3.795862 -1.187464 1.744231
C -0.300136 2.503657 -1.841695
H 0.082594 3.452615 -2.237690
H -0.936285 2.061402 -2.616554
H -0.949932 2.749154 -0.993509
58
D'_Et_conf_6 Eopt -845.400530
C 1.497051 -1.914616 1.389351
C 1.594417 -0.369472 1.301673
C 1.519876 -0.553915 -1.289547
C 1.383749 -2.092075 -1.139329
H 0.447346 -2.199510 1.535945
H 1.835431 -2.581490 -2.015927
C 3.089009 0.070669 1.216637
H 3.741533 -0.793417 1.413957
C 3.022776 -0.135599 -1.338646
H 3.206860 0.446876 -2.251275
H 1.152203 0.037991 2.218975
H 1.048682 -0.263688 -2.237116
C 3.470828 0.694011 -0.130354
H 2.997485 1.686646 -0.199970
H 4.555696 0.864640 -0.172940
C 2.033159 -2.616554 0.141829
H 1.863123 -3.699138 0.224252
H 3.123361 -2.485160 0.082370
H 3.303150 0.796771 2.012639
H 3.656396 -1.031056 -1.422158
H 2.054722 -2.256334 2.275185
H 0.319036 -2.371199 -1.135043
B 0.820565 0.093100 -0.012649
C -0.466880 1.074157 -0.045904
C -1.731938 0.105307 -0.074901
C -3.019111 0.884386 0.271171
H -2.997344 1.153549 1.332637
C -1.880658 -0.604007 -1.444547
H -0.898037 -0.680779 -1.917582
C -0.489794 2.010243 -1.281578
H -0.827092 1.450498 -2.161570
H -2.480715 0.034923 -2.106590
C -0.538404 1.897786 1.263543
H -1.410863 2.563199 1.228665
H -0.718307 1.192117 2.085665
H -2.958839 1.832399 -0.279765
H -1.249506 2.789452 -1.113049
O -1.380631 -0.871984 0.916878
C -2.345290 -1.478153 1.735093
H -2.923370 -0.742497 2.313471
H -3.042525 -2.119496 1.177921
H -1.795530 -2.107265 2.446419
C 0.819158 2.697852 -1.679929
H 0.637435 3.367107 -2.531383
H 1.250071 3.302889 -0.876726
H 1.574455 1.970345 -1.997967
C -4.375715 0.257867 -0.054012
H -4.562391 -0.672891 0.492422
H -5.167768 0.965039 0.224319
H -4.486796 0.052519 -1.125712
C -2.482970 -2.006680 -1.407144
H -2.467866 -2.436679 -2.416786
H -1.897731 -2.664573 -0.753107
H -3.520809 -2.017783 -1.058644
C 0.672616 2.766571 1.613749
H 0.680481 3.702114 1.042979
H 0.642504 3.041155 2.676353
H 1.626466 2.256417 1.434419
58
D'_Et_conf_7 Eopt -845.408602
C -2.332199 1.036349 1.635309
C -1.442477 -0.219600 1.385777
C -1.784609 0.084029 -1.174298

C	-2.737952	1.283609	-0.889432
H	-1.931871	1.606892	2.485199
H	-3.781771	0.935176	-0.891778
C	-2.303426	-1.498670	1.326956
H	-2.875984	-1.604846	2.262853
C	-2.609289	-1.220057	-1.201271
H	-1.966694	-2.060956	-1.495574
H	-0.775631	-0.322648	2.250990
H	-1.338817	0.214166	-2.171721
C	-3.276014	-1.524178	0.144520
H	-3.781940	-2.499398	0.099909
H	-4.070203	-0.784110	0.324124
C	-2.455849	1.990395	0.439093
H	-1.515652	2.550006	0.338890
H	-3.237116	2.737660	0.641079
H	-1.648822	-2.379899	1.273687
H	-3.388256	-1.151646	-1.978039
H	-3.340560	0.713861	1.941711
H	-2.680015	2.020150	-1.702240
B	-0.657659	-0.032540	-0.016406
C	0.815242	0.728267	-0.076931
C	1.548887	-0.650220	0.046970
C	2.733374	-0.977249	-0.884831
H	3.449925	-0.151344	-0.787158
C	1.908290	-1.120405	1.469123
H	2.035392	-2.208445	1.404949
C	1.127123	1.720638	1.051618
H	0.343905	2.490237	1.074699
H	1.059480	-0.946950	2.136988
C	1.096586	1.369048	-1.447864
H	2.178508	1.532558	-1.569763
H	0.805428	0.665192	-2.234897
H	3.243477	-1.854167	-0.463819
H	1.050093	1.211017	2.020161
O	0.355808	-1.416767	-0.369567
C	0.355913	-2.834634	-0.282139
H	1.302493	-3.216228	-0.684868
H	0.233024	-3.181565	0.749248
H	-0.463950	-3.213683	-0.895280
C	2.481809	2.428671	0.932247
H	2.753448	2.921963	1.874781
H	3.288430	1.730394	0.674284
H	2.461379	3.199948	0.152472
C	2.443753	-1.254764	-2.365785
H	2.563297	-0.362312	-2.987338
H	3.140753	-2.012448	-2.743651
H	1.425661	-1.633511	-2.520554
C	3.183255	-0.567544	2.105960
H	4.041652	-0.616584	1.424800
H	3.072717	0.465587	2.446017
H	3.430903	-1.174387	2.985748
C	0.377853	2.689188	-1.715710
H	0.613774	3.457161	-0.968227
H	0.672054	3.084767	-2.696802
H	-0.708299	2.557998	-1.728172

58

D'_Et_conf_8 Eopt -845.408211

C	-2.558958	-1.214572	-1.302302
C	-1.870559	0.157227	-1.151067
C	-1.384496	-0.309169	1.348032
C	-2.064456	-1.689751	1.248268
H	-1.881208	-1.861711	-1.876201
H	-2.669186	-1.875789	2.151247
C	-2.856402	1.264866	-0.719231
H	-3.667766	1.353791	-1.460293
C	-2.368381	0.816037	1.725877
H	-1.796957	1.746267	1.875300
H	-1.489534	0.429713	-2.148420
H	-0.672920	-0.370972	2.182563
C	-3.452275	1.084012	0.679567
H	-4.006346	1.993375	0.954365
H	-4.190754	0.273727	0.683666
C	-2.964884	-1.923435	0.011618
H	-3.022009	-3.005940	-0.178965
H	-3.989345	-1.629961	0.264804
H	-2.333214	2.232078	-0.720102
H	-2.840358	0.587133	2.695289
H	-3.460577	-1.114541	-1.929097
H	-1.270880	-2.450650	1.270812
B	-0.683106	0.032722	-0.069875
C	0.730466	0.890480	-0.156827
C	1.558605	-0.448615	-0.090592
C	2.705442	-0.614927	-1.097730
H	2.281586	-0.578165	-2.107418
C	1.978205	-0.942128	1.299837
H	2.196127	-2.012806	1.205423
C	0.974114	1.884797	0.983991
H	0.125415	2.582832	1.016936
H	1.129260	-0.865456	1.983074
C	0.953932	1.571981	-1.523270
H	2.017214	1.829209	-1.631222
H	0.738356	0.853583	-2.325744
H	3.342510	0.274737	-1.004321
H	0.939492	1.354275	1.944100
O	0.384996	-1.249696	-0.515876
C	0.413423	-2.656716	-0.701272
H	-0.616956	-3.012703	-0.747743
H	0.914261	-2.891807	-1.647798
H	0.921865	-3.159406	0.128786
C	2.262372	2.706147	0.873987
H	2.177036	3.475703	0.096117
H	2.486348	3.217331	1.819540
H	3.126702	2.080380	0.617531
C	3.593494	-1.855437	-0.970435
H	4.319903	-1.861282	-1.792614
H	4.161541	-1.865354	-0.033183
H	3.029309	-2.792711	-1.027039
C	3.201655	-0.271877	1.931451
H	4.002173	-0.085985	1.205371
H	2.951725	0.682840	2.403194
H	3.610669	-0.925319	2.711850
C	0.142236	2.837189	-1.798413
H	0.544668	3.352306	-2.680793
H	-0.906841	2.610048	-2.008335
H	0.173045	3.548537	-0.963235

58

D'_Et_conf_9 Eopt -845.402392

C	2.578151	0.322667	-1.817683
C	1.392500	-0.505233	-1.267135
C	1.963420	0.329772	1.111413
C	3.214260	1.049288	0.552444
H	2.183503	1.292952	-2.161677
H	4.032304	0.976691	1.286667
C	1.762640	-1.949568	-0.855248
H	2.263431	-2.459030	-1.693970
C	2.224015	-1.133403	1.548879
H	1.302247	-1.516706	2.013308
H	0.671356	-0.570408	-2.088951
H	1.668252	0.890356	2.005604
C	2.628627	-2.080195	0.409713
H	2.565735	-3.115882	0.772781
H	3.683473	-1.922814	0.161021
C	3.711485	0.575863	-0.819858
H	4.388628	1.336844	-1.233638
H	4.318638	-0.329654	-0.714325
H	0.820430	-2.491902	-0.680668
H	3.000141	-1.151837	2.329890
H	2.982122	-0.174403	-2.713500
H	2.973559	2.121275	0.478928
B	0.821508	0.243499	0.012043
C	-0.642863	0.910212	0.091217
C	-1.689588	-0.265208	-0.012689
C	-3.084191	0.083680	0.540961
H	-3.684103	-0.834547	0.527834
C	-1.838133	-0.865536	-1.436924
H	-0.971228	-1.510385	-1.609947
C	-0.719093	1.902806	-1.101659
H	-0.418580	1.380345	-2.018201
H	-1.776899	-0.062664	-2.177894
C	-0.807983	1.704933	1.414167

H 0.023521 2.420407 1.449616
H -1.717956 2.323893 1.364746
H -2.999526 0.366079 1.591835
H -1.751790 2.234417 -1.266045
O -1.058210 -1.233003 0.858611
C -1.549007 -2.543987 0.932121
H -1.422000 -3.092395 -0.013730
H -0.962875 -3.055852 1.705114
H -2.608913 -2.582652 1.226709
C 0.172034 3.138796 -0.960455
H -0.213668 3.842571 -0.213479
H 1.196944 2.871565 -0.665288
H 0.232128 3.674117 -1.916215
C -3.848427 1.176670 -0.208854
H -4.906969 1.156812 0.079315
H -3.463998 2.175629 0.027149
H -3.798530 1.052039 -1.298085
C -3.104074 -1.672236 -1.748170
H -3.284290 -2.485979 -1.037807
H -4.002533 -1.044919 -1.767592
H -3.001254 -2.122516 -2.743788
C -0.795742 0.949500 2.751294
H -0.053843 0.146798 2.766165
H -0.561393 1.650479 3.564031
H -1.756121 0.484882 2.993785
46
D'_Me_conf_1 Eopt -688.352813
C 1.785344 1.585483 -1.147737
C 1.245262 0.136038 -1.298962
C 1.214832 -0.072380 1.295731
C 1.775849 1.370253 1.404481
H 1.379555 2.206895 -1.957554
H 2.868836 1.345645 1.536739
C 2.397971 -0.904683 -1.351562
H 3.043490 -0.694928 -2.218069
C 2.356395 -1.125606 1.173517
H 1.919575 -2.137533 1.139600
H 0.683694 0.053905 -2.236839
H 0.659813 -0.320441 2.208024
C 3.227413 -0.920696 -0.066728
H 3.985237 -1.714419 -0.121594
H 3.780845 0.025302 0.025519
C 1.425669 2.238560 0.191939
H 0.339855 2.419181 0.202869
H 1.913842 3.220127 0.275604
H 1.976702 -1.911112 -1.512459
H 2.975749 -1.085593 2.082205
H 2.879433 1.597956 -1.275477
H 1.374656 1.849565 2.308012
B 0.396818 -0.307562 -0.042026
C -0.972624 -1.142043 -0.121509
C -2.079742 -0.075155 0.180777
C -3.464529 -0.511070 -0.301968
H -4.242858 0.170922 0.061633
H -3.509719 -0.538470 -1.396060
C -2.165873 0.290043 1.663617
H -2.453353 -0.586010 2.256133
H -2.937420 1.052707 1.822378
C -1.001904 -2.299469 0.885670
H -0.700582 -1.995959 1.895094
H -0.317190 -3.094239 0.558848
H -1.213894 0.677656 2.043263
C -1.176070 -1.728713 -1.527136
H -1.343520 -0.942973 -2.273137
H -0.289680 -2.299490 -1.835415
H -2.031839 -2.418122 -1.554698
H -3.700358 -1.511198 0.083914
H -2.004850 -2.747769 0.951561
O -1.620694 1.057458 -0.579346
C -2.325185 2.266050 -0.460564
H -1.912104 2.949593 -1.211877
H -3.399636 2.141865 -0.662624
H -2.199676 2.725407 0.531905
46
D'_Me_conf_2 Eopt -688.356748
C 1.904268 -1.032377 1.451071
C 0.977426 0.209137 1.260690
C 1.167651 -0.088336 -1.321309
C 2.128696 -1.294844 -1.125345
H 1.560379 -1.621474 2.313060
H 3.170853 -0.968931 -1.269630
C 1.821556 1.495308 1.154304
H 2.443564 1.607532 2.057619
C 1.960429 1.231995 -1.376993
H 1.271454 2.066130 -1.584755
H 0.344681 0.303027 2.156220
H 0.648519 -0.201052 -2.284237
C 2.724157 1.516451 -0.081493
H 3.238436 2.485761 -0.152207
H 3.517802 0.763057 0.039505
C 1.991886 -1.981977 0.241996
H 1.083278 -2.597748 0.217668
H 2.825890 -2.683197 0.390970
H 1.163916 2.376133 1.130301
H 2.672010 1.209684 -2.218678
H 2.918113 -0.689379 1.712209
H 1.945752 -2.049821 -1.903084
B 0.130080 -0.049625 -0.086967
C -1.190902 -1.044448 -0.041058
C -2.112714 0.208598 0.002022
C -3.237726 0.292811 -1.012591
H -3.778750 1.243712 -0.922371
H -2.866920 0.193701 -2.037070
C -2.630000 0.567331 1.386397
H -3.339721 -0.204005 1.706042
H -3.168029 1.522573 1.369527
C -1.368019 -1.965723 1.166959
H -1.106157 -1.484936 2.117098
H -0.735381 -2.855933 1.063292
H -1.825349 0.623916 2.126076
C -1.369307 -1.879092 -1.310663
H -1.280564 -1.285659 -2.227937
H -0.592294 -2.655827 -1.350419
H -2.343502 -2.392406 -1.332927
H -3.953708 -0.516736 -0.819105
H -2.405910 -2.326837 1.246748
O -1.035394 1.149198 -0.395598
C -1.144498 2.524478 -0.057679
H -0.343113 3.059056 -0.572751
H -2.111096 2.900202 -0.413648
H -1.058820 2.678129 1.024233
30
E_Et_conf_1 Eopt -392.628615
C -0.673004 -0.000223 -0.000078
C 0.673006 0.000226 -0.000070
C 1.491691 -1.272442 -0.005676
H 2.408172 -1.102629 0.579972
H 0.955917 -2.090255 0.491835
C 1.490843 1.273470 0.005619
H 2.407997 1.104012 -0.579054
C -1.491727 1.272426 -0.005689
H -2.408224 1.102580 0.579925
H 0.954973 2.090657 -0.492841
C -1.490803 -1.273490 0.005625
H -2.407930 -1.104094 -0.579107
H -0.954864 -2.090677 -0.492763
H -0.956000 2.090244 0.491859
C -1.879340 1.696887 -1.424597
H -2.530249 2.581305 -1.412783
H -0.983070 1.935398 -2.013467
H -2.414162 0.888116 -1.941458
C -1.876910 -1.698830 1.424681
H -0.979983 -1.936825 2.012754
H -2.411960 -0.890650 1.942241
H -2.527133 -2.583756 1.413060
C 1.876899 1.698874 1.424669
H 0.979954 1.936935 2.012688
H 2.411902 0.890710 1.942300
H 2.527152 2.583778 1.413021
C 1.879343 -1.696897 -1.424576

H 2.414245 -0.888151 -1.941395
H 2.530194 -2.581358 -1.412742
H 0.983088 -1.935342 -2.013495
30
E_Et_conf_2 Eopt -392.626336
C 0.513116 -0.436911 -0.023616
C -0.513219 0.436987 0.023485
C -0.440284 1.828432 -0.578636
H 0.022940 1.804727 -1.573936
H -1.461691 2.203037 -0.729525
C -1.827246 0.130355 0.706355
H -1.746228 -0.761297 1.338041
C 0.440268 -1.828392 0.578510
H 1.461758 -2.202790 0.729504
H -2.073619 0.969649 1.378186
C 1.827209 -0.130117 -0.706284
H 2.073599 -0.969389 -1.378133
H 1.746220 0.761496 -1.338060
H -0.022897 -1.804715 1.573856
C -0.316497 -2.814493 -0.314237
H -1.374902 -2.535439 -0.393917
H 0.105583 -2.823141 -1.328939
H -0.261225 -3.835512 0.086869
C 2.985445 0.057666 0.279380
H 3.150330 -0.844198 0.883376
H 3.918717 0.276213 -0.256298
H 2.782094 0.887626 0.968702
C -2.985478 -0.057750 -0.279259
H -3.150023 0.843616 -0.884058
H -2.782222 -0.888392 -0.967811
H -3.918867 -0.275616 0.256490
C 0.316605 2.814338 0.314247
H -0.105703 2.823217 1.328845
H 1.374877 2.534889 0.394118
H 0.261753 3.835337 -0.086975
30
E_Et_conf_3 Eopt -392.622922
C -0.676952 -0.000318 0.002259
C 0.676908 0.000307 0.002367
C 1.520546 -1.262233 0.026611
H 0.898692 -2.154515 0.120833
H 2.047919 -1.360536 -0.935928
C 1.519505 1.263370 -0.023630
H 0.896858 2.155799 -0.110921
C -1.520613 1.262209 0.026258
H -0.898827 2.154609 0.119753
H 2.053482 1.358954 0.935468
C -1.519436 -1.263513 -0.023574
H -0.896612 -2.155783 -0.111226
H -2.053031 -1.359317 0.935733
H -2.048353 1.360023 -0.936111
C -2.552441 1.280153 1.158168
H -2.064401 1.120398 2.129892
H -3.064305 2.250293 1.190250
H -3.319278 0.504742 1.033212
C -2.544321 -1.284158 -1.161746
H -2.050259 -1.127270 -2.130888
H -3.056643 -2.254043 -1.194309
H -3.311334 -0.507856 -1.043481
C 2.543920 1.283835 -1.162225
H 3.056808 2.253427 -1.194689
H 3.310480 0.507008 -1.044536
H 2.049328 1.127550 -2.131193
C 2.552860 -1.279647 1.158078
H 3.064543 -2.249864 1.190529
H 3.319804 -0.504456 1.032290
H 2.065282 -1.119204 2.129924
30
E_Et_conf_4 Eopt -392.626998
C 0.757064 0.166416 -0.102725
C -0.501760 -0.182129 -0.434930
C -1.005241 -1.605412 -0.274617
H -1.837908 -1.766086 -0.975571
H -0.221814 -2.317998 -0.566251
C -1.510350 0.798045 -0.991703
H -1.072103 1.797208 -1.082359
C 1.287109 1.581987 -0.216675
H 2.382231 1.542469 -0.308778
H -1.772788 0.477668 -2.014324
C 1.766871 -0.829044 0.426741
H 2.361645 -0.338771 1.213475
H 1.277117 -1.686787 0.900328
H 0.923310 2.074000 -1.127430
C 0.920134 2.425105 1.006735
H 1.271308 1.942803 1.929753
H 1.368552 3.426282 0.952175
H -0.170130 2.537907 1.083210
C 2.708234 -1.324287 -0.674777
H 2.145497 -1.870350 -1.444282
H 3.217589 -0.483634 -1.165442
H 3.476186 -1.995477 -0.268034
C -2.801162 0.896930 -0.174602
H -3.343543 -0.057541 -0.157806
H -2.588579 1.187013 0.863762
H -3.469809 1.650481 -0.610207
C -1.479117 -1.936872 1.144612
H -1.836347 -2.973935 1.204710
H -0.663616 -1.813729 1.869495
H -2.297486 -1.275955 1.455213
30
E_Et_conf_5 Eopt -392.627218
C -0.668496 -0.088588 -0.608116
C 0.668351 0.088706 -0.608181
C 1.310022 1.465085 -0.572304
H 2.366345 1.367895 -0.861305
H 0.850829 2.121902 -1.324120
C 1.650961 -1.060376 -0.608613
H 1.137780 -2.019011 -0.739358
C -1.310066 -1.465004 -0.572226
H -2.366503 -1.367855 -0.860830
H 2.317711 -0.941480 -1.478749
C -1.651170 1.060448 -0.608471
H -1.138047 2.019121 -0.739142
H -2.317896 0.941582 -1.478629
H -0.851082 -2.121665 -1.324307
C -1.229219 -2.137027 0.801173
H -1.677723 -1.501698 1.576188
H -1.756029 -3.101110 0.799723
H -0.185040 -2.319634 1.087004
C -2.514670 1.115929 0.655255
H -3.196857 1.975121 0.619703
H -3.125300 0.209301 0.762163
H -1.889056 1.209549 1.553703
C 2.514404 -1.115989 0.655146
H 3.196583 -1.975185 0.619545
H 3.125032 -0.209374 0.762173
H 1.888746 -1.209693 1.553555
C 1.229796 2.136884 0.801242
H 0.185760 2.319567 1.087534
H 1.678544 1.501364 1.575963
H 1.756722 3.100904 0.799749
30
E_Et_conf_6 Eopt -392.626147
C 0.616233 -0.051626 0.269215
C -0.616242 0.051656 -0.269424
C -1.394290 -1.155000 -0.764917
H -2.172998 -0.810677 -1.460986
H -0.739586 -1.815591 -1.349772
C -1.340112 1.372194 -0.417196
H -0.715183 2.203354 -0.075918
C 1.394241 1.154990 0.764879
H 0.739483 1.815545 1.349707
H -1.522503 1.544045 -1.491222
C 1.340012 -1.372212 0.417035
H 1.522085 -1.544214 1.491093
H 0.715110 -2.203280 0.075475
H 2.172867 0.810619 1.461019
C 2.052200 1.963064 -0.357187
H 2.737596 1.339059 -0.944743
H 1.297194 2.361827 -1.047656

H 2.623347 2.808395 0.050449
C 2.682396 -1.421683 -0.317547
H 3.153594 -2.404409 -0.187897
H 2.544137 -1.247152 -1.393563
H 3.380265 -0.665092 0.064487
C -2.682301 1.421614 0.317751
H -3.153660 2.404265 0.188116
H -3.380176 0.664884 -0.064002
H -2.543711 1.247220 1.393747
C -2.052134 -1.962976 0.357281
H -2.623339 -2.808331 -0.050226
H -1.297075 -2.361695 1.047716
H -2.737464 -1.338897 0.944843
30
E_Et_conf_7 Eopt -392.626147
C 0.616222 0.051476 0.269537
C -0.616176 -0.051487 -0.269273
C -1.393812 1.155356 -0.764873
H -0.738641 1.816397 -1.348731
H -2.171945 0.811302 -1.461742
C -1.340115 -1.371994 -0.417396
H -1.522198 -1.543666 -1.491541
C 1.393942 -1.155328 0.765101
H 2.172169 -0.811217 1.461838
H -0.715259 -2.203172 -0.075955
C 1.340220 1.371966 0.417504
H 0.715244 2.203220 0.076480
H 1.522870 1.543477 1.491575
H 0.738868 -1.816364 1.349064
C 2.052579 -1.962574 -0.357275
H 1.297768 -2.361304 -1.048041
H 2.737801 -1.337791 -0.944278
H 2.624075 -2.807869 0.050067
C 2.682192 1.421662 -0.317892
H 2.543146 1.247681 -1.393955
H 3.153469 2.404375 -0.188037
H 3.380248 0.664792 0.063350
C -2.682450 -1.421687 0.317309
H -3.380336 -0.664877 -0.064361
H -3.153633 -2.404420 0.187266
H -2.543984 -1.247627 1.393433
C -2.052583 1.962606 0.357414
H -2.737886 1.337846 0.944352
H -1.297874 2.361331 1.048295
H -2.624010 2.807906 -0.050010
30
E_Et_conf_8 Eopt -392.626734
C 0.577088 -0.254512 -0.383533
C -0.712761 0.008341 -0.091624
C -1.370835 1.337903 -0.383911
H -0.758350 1.938615 -1.066256
H -2.323616 1.146574 -0.905094
C -1.646123 -1.023613 0.511057
H -1.125894 -1.644991 1.251131
C 1.227718 -1.590398 -0.101080
H 0.488798 -2.334803 0.212835
H -2.446002 -0.505827 1.058345
C 1.503692 0.770919 -1.015141
H 2.391841 0.247463 -1.396592
H 1.022983 1.226418 -1.892795
H 1.662046 -1.964616 -1.042510
C 2.337866 -1.500130 0.949374
H 3.132988 -0.811927 0.631988
H 1.935984 -1.137638 1.905830
H 2.794679 -2.483825 1.118569
C 1.955822 1.878683 -0.060608
H 2.704614 2.525137 -0.538370
H 1.109876 2.508630 0.241312
H 2.398171 1.456367 0.851431
C -2.278276 -1.915683 -0.560387
H -2.799465 -1.308675 -1.313742
H -1.508397 -2.502691 -1.079297
H -3.006294 -2.611319 -0.121631
C -1.667577 2.151214 0.880576
H -0.746228 2.355051 1.441845
H -2.135023 3.111257 0.624620
H -2.352410 1.614465 1.550043
30
E_Et_conf_9 Eopt -392.626734
C -0.712435 0.008518 -0.091630
C 0.577254 -0.254701 -0.383910
C 1.227436 -1.590867 -0.101823
H 0.488078 -2.335709 0.210031
H 1.663389 -1.964030 -1.042905
C 1.504159 0.770621 -1.015240
H 2.392388 0.247025 -1.396336
C -1.370223 1.338282 -0.383624
H -0.757676 1.938830 -1.066080
H 1.023805 1.226120 -1.893102
C -1.645933 -1.023401 0.510910
H -1.125614 -1.645341 1.250443
H -2.445388 -0.505613 1.058823
H -2.323145 1.147245 -0.904693
C -1.666564 2.151774 0.880809
H -0.744996 2.356364 1.441442
H -2.134726 3.111469 0.624829
H -2.350665 1.614902 1.550920
C -2.278976 -1.914680 -0.560637
H -1.509631 -2.501678 -1.080359
H -3.006966 -2.610319 -0.121832
H -2.800446 -1.307103 -1.313341
C 1.956194 1.878448 -0.060703
H 1.110442 2.509149 0.240160
H 2.397424 1.456215 0.851907
H 2.705879 2.524185 -0.538041
C 2.335946 -1.501362 0.950411
H 3.131496 -0.812895 0.634633
H 1.932625 -1.139469 1.906489
H 2.792578 -2.485138 1.119636
18
E_Me_conf_1 Eopt -235.562998
C -0.000001 0.673969 -0.000063
C 0.000001 -0.673969 -0.000063
C 1.251377 -1.516449 0.027868
H 1.402824 -2.019134 -0.940696
H 2.159152 -0.957733 0.270771
C -1.251377 -1.516476 -0.027839
H -1.402826 -2.019022 0.940795
H -1.140347 -2.314941 -0.777309
C -1.251377 1.516449 0.027868
H -2.159152 0.957733 0.270771
H -1.402824 2.019134 -0.940696
H -2.159158 -0.957799 -0.270816
C 1.251377 1.516476 -0.027839
H 2.159158 0.957799 -0.270816
H 1.140347 2.314941 -0.777309
H 1.402826 2.019022 0.940795
H 1.140336 -2.314798 0.777459
H -1.140336 2.314798 0.777459
28
F_conf_1 Eopt -452.807818
C 0.428809 1.301878 -1.343321
C -0.381608 0.001174 -1.126543
C 0.359952 0.000187 1.384940
C 1.160076 1.300113 1.136060
H -0.277487 2.146825 -1.294183
H 2.065955 1.312477 1.762846
C 0.424330 -1.302517 -1.342793
H 0.844466 -1.317912 -2.360986
C 1.157490 -1.301386 1.136030
H 0.535922 -2.144158 1.479812
H -1.210341 0.002428 -1.848776
H 0.022816 0.000455 2.431913
C 1.551227 -1.559063 -0.328174
H 1.873455 -2.605777 -0.424111
H 2.430273 -0.960561 -0.589663
C 1.555606 1.556036 -0.327938
H 1.880606 2.601862 -0.424195
H 2.433379 0.955143 -0.588250
H -0.284623 -2.145176 -1.292171

H 2.063871 -1.315081 1.762066
H 0.849912 1.314905 -2.361157
H 0.539829 2.144286 1.478809
B -0.852730 0.001236 0.379700
O -2.134563 0.001197 0.825025
C -3.230049 0.001730 -0.075507
H -3.216053 0.897214 -0.711491
H -3.215915 -0.892800 -0.712830
H -4.150909 0.001197 0.517148
28
F_conf_2 Eopt -452.807960
C -0.724202 1.172611 1.405451
C 0.351950 0.081581 1.140258
C -0.465292 -0.024952 -1.357295
C -1.520526 1.067853 -1.029002
H -0.352085 1.877173 2.160966
H -2.492969 0.603276 -0.802174
C -0.212526 -1.352080 1.340316
H -0.547032 -1.473453 2.381992
C -1.008194 -1.452977 -1.085432
H -0.246774 -2.192405 -1.383914
H 1.189506 0.212865 1.837184
H -0.172040 0.036026 -2.413772
C -1.367595 -1.675397 0.387085
H -1.683411 -2.717318 0.535528
H -2.237702 -1.054551 0.647183
C -1.102127 1.961189 0.145286
H -0.224471 2.554276 -0.167258
H -1.897971 2.683339 0.375840
H 0.595712 -2.086031 1.183696
H -1.891381 -1.641913 -1.714949
H -1.630153 0.716702 1.834341
H -1.683976 1.699952 -1.911797
B 0.771948 0.104943 -0.385047
O 2.040359 0.082522 -0.867942
C 3.163126 0.101841 -0.003005
H 3.168542 -0.777439 0.655602
H 4.065250 0.085022 -0.623736
H 3.170659 1.011582 0.612070
5
G_conf_1 Eopt -114.916098
C -0.571352 -0.001622 -0.014575
H -0.871112 0.055278 1.057121
H -0.999237 0.892974 -0.499647
H -1.002437 -0.935213 -0.414421
O 0.787612 -0.000414 -0.006950
2
HI Eopt -298.224903
I 0.000000 0.000000 0.029850
H 0.000000 0.000000 -1.582049
20
HI_cluster_conf_1 Eopt -645.060344
I 1.634612 0.000830 -0.196178
C -1.211414 2.351938 1.556634
H -0.773783 1.573261 2.195440
H -2.271459 2.477276 1.798103
H -0.684376 3.299564 1.713455
O -1.130344 1.976429 0.177981
H -0.212453 1.699574 -0.024444
O -1.127297 -1.978965 0.179390
H -0.210190 -1.701016 -0.025083
O -2.428719 -0.002440 -0.483608
H -1.899153 -0.867643 -0.162068
C -1.205619 -2.349460 1.559569
H -0.672767 -3.293217 1.719998
H -0.772538 -1.565576 2.195128
H -2.264816 -2.480307 1.801818
C -2.541436 -0.002712 -1.922859
H -3.094835 0.893790 -2.214252
H -1.542004 -0.002288 -2.374456
H -3.093980 -0.899832 -2.213975
H -1.900407 0.863653 -0.162410
20
HI_cluster_conf_2 Eopt -645.060600
I -1.574314 -0.230930 -0.149017
C 1.660754 -2.683762 0.677690
H 2.749096 -2.797921 0.652957
H 1.245430 -2.835868 -0.327739
H 1.233495 -3.418355 1.369047
O 1.380431 -1.360375 1.145489
H 0.422234 -1.180081 1.048921
O 0.763497 2.271972 -0.175828
H -0.082187 1.808955 -0.348709
O 2.364895 0.408660 -0.255604
H 1.706430 1.245969 -0.210998
C 0.695565 2.889127 1.115260
H -0.042449 3.698335 1.099856
H 0.423241 2.151018 1.882021
H 1.686883 3.299609 1.330411
C 2.425774 -0.092256 -1.608561
H 1.429344 -0.418660 -1.931479
H 2.787988 0.717889 -2.246707
H 3.133305 -0.925737 -1.625634
H 1.982677 -0.346581 0.387142
20
HI_cluster_conf_3 Eopt -645.060601
I -1.574404 -0.231862 0.149188
C 1.664202 -2.680895 -0.679237
H 1.240102 -3.415301 -1.372777
H 1.246666 -2.835284 0.324953
H 2.752713 -2.793123 -0.651872
O 1.382660 -1.357352 -1.145745
H 0.424172 -1.178415 -1.050311
O 0.761302 2.272545 0.175588
H -0.083390 1.807885 0.349092
O 2.364771 0.411451 0.257907
H 1.705271 1.248163 0.213107
C 0.693046 2.884129 -1.118151
H -0.051578 3.687360 -1.108568
H 1.681821 3.301742 -1.331392
H 0.429368 2.140903 -1.883064
C 2.425342 -0.090441 1.610412
H 1.429331 -0.419535 1.931979
H 3.134829 -0.922285 1.627697
H 2.784889 0.720046 2.249683
H 1.983844 -0.343353 -0.385606
20
HI_cluster_conf_4 Eopt -645.060769
I 1.577832 -0.002905 0.102903
C -1.186681 3.066341 0.043529
H -0.844827 2.775061 1.046118
H -2.250558 3.322454 0.071559
H -0.613291 3.932013 -0.305545
O -1.032170 1.981993 -0.877934
H -0.119412 1.632912 -0.813670
O -1.040008 -1.978336 -0.877743
H -0.125742 -1.633373 -0.813076
O -2.368579 0.004573 -0.279400
H -1.832150 -0.861266 -0.588336
C -1.200023 -3.062101 0.043500
H -0.857200 -2.772584 1.046261
H -2.265124 -3.313136 0.071054
H -0.630637 -3.930435 -0.305510
C -2.477493 0.004718 1.160623
H -3.033207 0.899378 1.454245
H -3.035473 -0.888531 1.454257
H -1.477419 0.003396 1.611878
H -1.828800 0.868473 -0.588377
20
HI_cluster_conf_5 Eopt -645.060600
I -1.574233 0.231641 0.149189
C 1.663309 2.681010 -0.678945
H 1.246973 2.834442 0.325876
H 1.237666 3.415405 -1.371520
H 2.751749 2.794079 -0.652991
O 1.382230 1.357481 -1.145826
H 0.423774 1.178260 -1.050429
O 0.761404 -2.272526 0.175823
H -0.083323 -1.807839 0.349222
O 2.364600 -0.411043 0.257637

H 1.705292 -1.247876 0.213081
C 0.693481 -2.883850 -1.118055
H -0.052125 -3.686157 -1.109231
H 0.431336 -2.140211 -1.883080
H 1.681921 -3.302616 -1.330504
C 2.425309 0.090793 1.610165
H 1.429217 0.419241 1.932071
H 2.785624 -0.719519 2.249196
H 3.134236 0.923100 1.627237
H 1.983517 0.343695 -0.386004
20
HI_cluster_conf_6 Eopt -645.060360
I 1.629919 -0.007518 0.166636
C -1.384358 -2.206168 -1.484266
H -0.807710 -1.519088 -2.117397
H -2.451200 -2.115094 -1.712855
H -1.057282 -3.235923 -1.665983
O -1.209861 -1.881198 -0.101307
H -0.270143 -1.656538 0.059880
O -1.116564 1.960306 -0.358462
H -0.195301 1.716595 -0.129958
O -2.376279 0.128972 0.696809
H -1.872276 0.953419 0.252074
C -1.263864 1.874529 -1.780622
H -1.015627 0.863907 -2.133986
H -2.308087 2.104872 -2.013103
H -0.607805 2.604776 -2.266365
C -2.269422 0.190979 2.135569
H -2.738236 1.122109 2.463927
H -2.806060 -0.668013 2.546502
H -1.213903 0.164951 2.432638
H -1.914578 -0.758187 0.332494
20
HI_cluster_conf_7 Eopt -645.045439
I -1.515578 -0.304600 -0.206473
C 3.115774 0.405579 -1.132326
H 3.921456 -0.325552 -1.240587
H 3.202870 0.914891 -0.164506
H 3.165346 1.130760 -1.950528
O 1.882529 -0.323156 -1.220568
H 1.102748 0.261823 -1.242086
O -0.285677 2.886633 0.909930
H -0.926180 2.193143 0.698452
O 1.267220 -1.963408 0.443485
H 0.282936 -1.818380 0.420216
C 0.975775 2.454679 0.472360
H 1.237655 1.472855 0.900578
H 1.030701 2.397518 -0.629884
H 1.721420 3.185870 0.808836
C 1.791393 -1.580341 1.740390
H 1.458591 -0.565214 1.986381
H 1.421627 -2.297399 2.476448
H 2.880529 -1.633310 1.667496
H 1.615704 -1.253251 -0.333055
20
HI_cluster_conf_8 Eopt -645.024004
I 0.197598 -1.212128 -0.380300
C -2.449614 1.034652 1.243208
H -3.231140 1.654252 1.699856
H -2.323750 1.353400 0.196536
H -1.507963 1.219885 1.787781
O -2.857366 -0.309714 1.342304
H -2.198402 -0.855274 0.893556
O -0.865214 1.879000 -1.925312
H -0.815217 0.937159 -1.713299
O 1.597438 0.945765 1.459303
H 1.033667 0.265088 0.873955
C 0.027145 2.565821 -1.081792
H -0.268476 2.497511 -0.021728
H 1.060123 2.196350 -1.189786
H 0.011286 3.623540 -1.371279
C 2.985481 0.986912 0.995422
H 3.442240 0.004903 1.144924
H 2.933526 1.244742 -0.064243
H 3.487528 1.767828 1.569332
H 1.526943 0.688668 2.398907
20
HI_cluster_conf_9 Eopt -645.060601
I -1.574403 -0.232977 -0.148994
C 1.667360 -2.681219 0.678195
H 2.756092 -2.790919 0.649709
H 1.249166 -2.836639 -0.325541
H 1.245677 -3.416489 1.372269
O 1.383284 -1.358269 1.144823
H 0.424604 -1.180723 1.048908
O 0.759695 2.272281 -0.175235
H -0.084893 1.807043 -0.347943
O 2.364653 0.412673 -0.256490
H 1.704360 1.248919 -0.211321
C 0.690741 2.888222 1.116350
H -0.050402 3.694585 1.102293
H 0.422037 2.148527 1.882890
H 1.680719 3.302328 1.330821
C 2.425461 -0.087976 -1.609488
H 1.429477 -0.416630 -1.931539
H 2.785320 0.723065 -2.247849
H 3.134759 -0.919951 -1.627387
H 1.983995 -0.342950 0.386226
23
H_conf_1 Eopt -337.665759
C 1.181208 1.282334 -0.362162
C 0.012401 1.318491 0.665454
C 0.011805 -1.318853 0.665333
C 1.180457 -1.282578 -0.362548
H 1.839020 2.144457 -0.192547
H 0.785190 -1.392149 -1.384454
C -1.384551 1.273570 -0.033672
H -1.481898 2.161231 -0.675997
C -1.385281 -1.272783 -0.033556
H -2.178083 -1.343934 0.728001
H 0.056071 2.241201 1.256425
H 0.054994 -2.242055 1.255527
C -1.592886 0.000380 -0.857070
H -2.606806 0.000543 -1.279885
H -0.903604 0.000086 -1.714095
C 2.016830 -0.000372 -0.275530
H 2.552345 -0.000667 0.689848
H 2.786554 -0.000580 -1.059692
H -2.177494 1.345272 0.727678
H -1.483324 -2.160440 -0.675783
H 0.786235 1.392442 -1.384104
H 1.837913 -2.145045 -0.193299
B -0.021402 -0.000298 1.492976
23
H_conf_2 Eopt -337.665738
C 1.306776 1.290569 -0.090334
C -0.001093 1.312561 0.746401
C 0.001123 -1.312449 0.746461
C 1.309459 -1.288229 -0.089386
H 2.149726 1.442333 0.602225
H 1.317118 -2.151470 -0.772944
C -1.309457 1.288167 -0.089614
H -1.317111 2.151438 -0.773129
C -1.306699 -1.290599 -0.090331
H -2.149809 -1.442271 0.602055
H -0.001834 2.237196 1.339769
H 0.001707 -2.237113 1.339791
C -1.558858 -0.001225 -0.886893
H -2.604754 -0.002249 -1.224908
H -0.957057 -0.000288 -1.801463
C 1.559015 0.001140 -0.886722
H 2.604959 0.002126 -1.224588
H 0.957354 0.000089 -1.801386
H -2.152457 1.437397 0.603451
H -1.311893 -2.153384 -0.774490
H 1.312104 2.153316 -0.774542
H 2.152479 -1.437458 0.603652
B -0.000424 0.000146 1.579805
2
I2 Eopt -595.280286

I 0.000000 0.000000 1.328531
I 0.000000 0.000000 -1.328531
1
I_anion Eopt -297.806502
I 0.000000 0.000000 0.000000
25
I_anion_MeOH4 Eopt -760.202544
I -0.016670 0.741877 -0.590715
C 2.156496 -2.350283 0.216205
H 2.808219 -1.529493 -0.124230
H 2.789646 -3.190034 0.528200
H 1.542578 -2.677535 -0.639157
C 3.496754 0.940698 1.022766
H 3.012450 0.039805 1.433835
H 4.525042 0.983663 1.402669
H 2.951317 1.824883 1.389841
C -1.762716 -2.610165 0.007571
H -2.273005 -3.573489 0.132057
H -0.716475 -2.804016 -0.275488
H -1.765021 -2.090350 0.979940
C -3.540594 0.788475 0.901228
H -4.523141 0.818205 1.388838
H -3.690542 0.886351 -0.186268
H -3.083797 -0.193189 1.102308
O -2.453275 -1.885295 -0.982869
H -2.009754 -1.031462 -1.083862
O -2.771305 1.846852 1.424349
H -1.895259 1.801427 1.017136
O 3.563166 0.912067 -0.385548
H 2.657046 0.869395 -0.722347
O 1.367151 -1.977475 1.322608
H 0.808691 -1.235201 1.049501
24
I_conf_1 Eopt -635.418997
C -2.162821 1.044089 1.279421
C -0.953908 0.066907 1.315984
C -0.953862 0.067608 -1.316114
C -2.162774 1.044808 -1.279008
H -2.108022 1.715642 2.145573
H -3.104520 0.484576 -1.381990
C -1.410933 -1.426116 1.275300
H -2.032849 -1.625632 2.160091
C -1.410956 -1.425506 -1.275779
H -0.527503 -2.078585 -1.355738
H -0.369320 0.213793 2.230876
H -0.369546 0.214626 -2.231136
C -2.183590 -1.772858 -0.000331
H -2.424080 -2.844496 -0.000668
H -3.146984 -1.242728 -0.000206
C -2.209893 1.886645 0.000444
H -1.340509 2.566961 0.000634
H -3.105705 2.522542 0.000555
H -0.527493 -2.079248 1.354890
H -2.032906 -1.624639 -2.160634
H -3.104564 0.483774 1.381970
H -2.107820 1.716891 -2.144742
B -0.122936 0.174097 -0.000077
I 2.030357 0.071558 0.000026
24
I_conf_2 Eopt -635.418621
C 1.558644 1.337268 -1.292427
C 0.908778 -0.075965 -1.310359
C 0.908762 -0.071905 1.310572
C 1.557577 1.341735 1.288245
H 0.756596 2.076270 -1.447503
H 2.234178 1.438484 2.150877
C 1.918541 -1.248402 -1.287952
H 2.599595 -1.159187 -2.148073
C 1.919471 -1.243594 1.291899
H 1.351200 -2.173748 1.448934
H 0.308602 -0.154501 -2.225730
H 0.308605 -0.148026 2.226158
C 2.741280 -1.387299 0.001918
H 3.218850 -2.377068 0.003592
H 3.565546 -0.666644 0.000186
C 2.312532 1.697628 -0.002375
H 2.503624 2.779773 -0.004171
H 3.300212 1.225721 -0.001066
H 1.349492 -2.178780 -1.440825
H 2.601146 -1.150492 2.151117
H 2.236135 1.430493 -2.154751
H 0.754928 2.080808 1.439877
B 0.067603 -0.124121 0.000177
I -2.082646 -0.047157 0.000064
1
J_conf_1 Eopt -297.613749
I 0.000000 0.000000 0.000000
6
MeOH Eopt -115.590008
O -0.744006 0.123491 0.000002
H -1.149909 -0.748964 0.000007
C 0.661607 -0.020509 0.000001
H 1.093640 0.987202 -0.000573
H 1.019387 -0.551072 0.896114
H 1.019291 -0.552042 -0.895571
24
MeOH_cluster Eopt -462.401934
C 2.435869 0.162782 1.460934
H 2.918603 1.128631 1.650608
H 3.192577 -0.630532 1.545496
H 1.663892 0.000129 2.229223
O 1.875256 0.198346 0.162240
H 1.359143 -0.627014 0.016775
O -1.877870 -0.196897 0.162641
O -0.198715 1.876516 -0.161366
H -1.361506 0.628269 0.016936
C -2.430292 -0.164429 1.464966
H -3.181635 0.632999 1.558101
H -2.917642 -1.128297 1.652845
H -1.652605 -0.010208 2.229264
C -0.164636 2.429359 -1.463462
H 0.631663 3.182119 -1.554827
H -0.007561 1.652172 -2.227682
H -1.128979 2.915092 -1.653111
H 0.626609 1.360734 -0.014539
C 0.163219 -2.430301 -1.463461
H 1.128826 -2.913718 -1.652610
H -0.631014 -3.185465 -1.552992
H 0.003647 -1.655031 -2.229084
O 0.196358 -1.874864 -0.162447
H -0.629212 -1.359151 -0.016803
54
TS-I-Anti_Et_conf_1 Eopt -1028.032797
C -3.196217 1.863553 0.592113
C -2.453444 0.629179 1.155708
C -2.749105 -0.599777 -1.103257
C -3.625522 0.569605 -1.625692
H -2.448879 2.604496 0.277698
H -4.481499 0.141604 -2.169246
C -3.412563 -0.400031 1.819536
H -4.013538 0.125133 2.576757
C -3.598748 -1.681354 -0.374696
H -2.947026 -2.511058 -0.064964
H -1.804549 0.982262 1.965394
H -2.293975 -1.072076 -1.981223
C -4.333597 -1.167506 0.864458
H -4.762068 -2.024154 1.402440
H -5.185733 -0.548550 0.566952
C -4.149245 1.570578 -0.577468
H -4.379005 2.516963 -1.085763
H -5.105652 1.219662 -0.177436
H -2.797451 -1.129450 2.366952
H -4.317107 -2.103922 -1.092237
H -3.765280 2.334905 1.407050
H -3.044884 1.115289 -2.381443
B -1.706103 -0.096278 -0.031154
C -0.092600 -0.556035 -0.039484
C 0.708292 0.682174 0.014933
C 0.896688 1.477355 -1.246075
H 1.103994 0.821977 -2.094188

C 0.856490 1.416483 1.309958
H -0.178502 1.660309 1.602155
C 0.223977 -1.399582 -1.301733
H 1.314907 -1.438748 -1.408434
H 1.209230 0.720239 2.078776
C -0.140047 -1.400007 1.270429
H -0.450462 -0.764074 2.107207
H -0.942543 -2.142172 1.148904
I 3.527420 -0.145965 -0.137679
H 1.752350 2.145102 -1.128560
H -0.154250 -0.872346 -2.185854
C -0.305451 -2.830447 -1.325383
H -0.016206 -3.297495 -2.274984
H 0.116330 -3.442656 -0.520236
H -1.395237 -2.885999 -1.254683
C 1.142152 -2.134701 1.660301
H 1.891272 -1.449157 2.068135
H 0.904574 -2.877679 2.432316
H 1.603522 -2.652763 0.812582
C -0.356026 2.318859 -1.526914
H -0.191884 2.908371 -2.436017
H -1.236839 1.689784 -1.699925
H -0.572502 3.008451 -0.702683
C 1.673081 2.700108 1.316345
H 1.236325 3.467007 0.665440
H 1.695934 3.103180 2.334979
H 2.705227 2.514345 0.998419
54
TS-I-Anti_Et_conf_10 Eopt -1028.028850
C 3.040296 -1.950980 -0.586983
C 2.444914 -1.124697 0.587767
C 2.744374 1.017747 -0.851092
C 3.319727 0.133407 -1.990696
H 2.220001 -2.349008 -1.206139
H 4.041412 0.731774 -2.565446
C 3.549892 -0.592668 1.548915
H 4.522937 -1.031327 1.283338
C 3.854199 1.492070 0.131593
H 3.857356 2.589004 0.173070
H 1.809604 -1.806420 1.152646
H 2.298319 1.892782 -1.327703
C 3.664108 0.933590 1.542947
H 2.734623 1.359109 1.964820
H 4.482120 1.258218 2.199726
C 3.982222 -1.141396 -1.474079
H 4.313656 -1.757799 -2.320473
H 4.889093 -0.883147 -0.908996
H 3.327058 -0.932429 2.568923
H 4.844293 1.197211 -0.245734
H 3.564614 -2.822564 -0.169543
H 2.510146 -0.133910 -2.688475
B 1.705708 0.101698 -0.077879
C 0.100639 0.506307 -0.098906
C -0.632860 -0.591633 0.556230
C -0.568764 -1.989302 0.021393
H 0.008663 -2.513065 0.802234
C -1.013685 -0.510605 2.008232
H -1.466387 0.456670 2.230826
C -0.267513 0.786428 -1.589823
H 0.502565 1.447790 -1.996017
H -1.761875 -1.283184 2.213825
C -0.054305 1.833949 0.708523
H -1.139200 2.003962 0.763262
H 0.288307 1.682827 1.739047
I -3.480573 0.298355 -0.133897
H 0.030985 -2.030989 -0.887458
H -1.193592 1.374589 -1.549377
C -0.470747 -0.361733 -2.569435
H -0.664690 0.065645 -3.560819
H 0.413514 -1.006961 -2.657751
H -1.336165 -0.980441 -2.306096
C 0.594640 3.112475 0.185838
H 1.681901 3.116751 0.314714
H 0.364284 3.305803 -0.868106
H 0.198538 3.955794 0.764674
C -1.858528 -2.792544 -0.152977
H -2.497836 -2.731859 0.733713
H -2.431562 -2.433215 -1.012476
H -1.591504 -3.842993 -0.317202
C 0.187232 -0.735170 2.949751
H -0.151755 -0.554209 3.975540
H 0.557995 -1.764818 2.891006
H 1.019351 -0.052366 2.739188
54
TS-I-Anti_Et_conf_11 Eopt -1028.029391
C 3.810538 -0.773040 1.251144
C 2.544653 0.143080 1.291512
C 2.550028 0.227584 -1.312968
C 3.838383 -0.651833 -1.332713
H 3.735899 -1.519011 2.052449
H 4.718616 -0.009662 -1.477861
C 2.985555 1.635087 1.319326
H 3.629619 1.782356 2.198188
C 2.917299 1.738454 -1.204302
H 1.999525 2.349444 -1.204524
H 1.989333 -0.047348 2.215546
H 2.008698 0.090919 -2.255507
C 3.721201 2.061202 0.052224
H 3.929433 3.138799 0.090867
H 4.699504 1.562362 0.004726
C 4.023455 -1.512185 -0.076382
H 3.305983 -2.344719 -0.124093
H 5.020807 -1.971797 -0.085586
H 2.110919 2.282897 1.463171
H 3.480779 2.023392 -2.104236
H 4.697389 -0.164775 1.480078
H 3.801750 -1.317036 -2.204637
B 1.708843 -0.096791 -0.024427
C 0.112376 -0.626020 -0.086121
C -0.569067 0.673550 -0.100658
C -0.467392 1.555499 1.110034
H 0.101219 2.426419 0.740026
C -0.841692 1.332876 -1.417739
H 0.165390 1.453401 -1.862476
C -0.228943 -1.484363 1.165516
H -1.104841 -2.066421 0.852396
H -1.352637 0.625193 -2.076312
C -0.101557 -1.428554 -1.389043
H -1.185796 -1.588262 -1.478707
H 0.200041 -0.828190 -2.251337
I -3.455053 -0.349331 0.007476
H 0.147716 1.064262 1.867696
H -0.594491 -0.843316 1.974889
C 0.843382 -2.435500 1.716488
H 1.590698 -2.716562 0.967651
H 1.377558 -2.002779 2.568388
H 0.372152 -3.361999 2.065668
C 0.605655 -2.775239 -1.477460
H 0.388120 -3.232263 -2.450219
H 1.695865 -2.672017 -1.398990
H 0.266122 -3.473098 -0.703136
C -1.727965 2.090435 1.794400
H -2.427881 2.545263 1.090623
H -2.245465 1.278819 2.314627
H -1.422647 2.845126 2.528969
C -1.562213 2.671544 -1.425756
H -2.601194 2.553280 -1.097466
H -1.073050 3.419927 -0.790112
H -1.573718 3.060445 -2.449879
54
TS-I-Anti_Et_conf_12 Eopt -1028.029804
C 3.113546 1.348987 -1.489827
C 2.635001 -0.127429 -1.295441
C 2.514026 0.257191 1.277581
C 2.963498 1.742927 1.064892
H 2.685687 1.740840 -2.421583
H 4.029178 1.826056 1.319649
C 3.857366 -1.055468 -1.048203
H 4.501154 -1.025829 -1.939001
C 3.768485 -0.646020 1.438778

H 3.463921 -1.675190 1.670575
H 2.148522 -0.463857 -2.215998
H 1.941943 0.190987 2.208528
C 4.651671 -0.664187 0.194786
H 5.479079 -1.371129 0.341406
H 5.114732 0.322326 0.048215
C 2.731892 2.299185 -0.347462
H 1.665697 2.552609 -0.456836
H 3.272579 3.248097 -0.459884
H 3.522091 -2.097935 -0.939251
H 4.340502 -0.284989 2.305625
H 4.203287 1.356433 -1.629658
H 2.428574 2.380251 1.780708
B 1.725336 -0.201204 -0.009174
C 0.107497 -0.688895 -0.066354
C -0.536536 0.628736 -0.028359
C -0.475593 1.443657 1.229875
H 0.165567 2.295432 0.943826
C -0.731603 1.351061 -1.325860
H 0.289529 1.399663 -1.755997
C -0.238498 -1.580576 1.159914
H -1.073488 -2.202369 0.811277
H -1.283078 0.704294 -2.014614
C -0.149308 -1.451905 -1.385426
H -1.238880 -1.566836 -1.468874
H 0.167641 -0.848724 -2.240164
I -3.462398 -0.328659 -0.035466
H 0.054326 0.886461 2.002759
H -0.659262 -0.975037 1.969010
C 0.867309 -2.475859 1.724628
H 1.558444 -2.832874 0.950312
H 1.457768 -1.956608 2.486834
H 0.422765 -3.358007 2.200800
C 0.499589 -2.826396 -1.494472
H 0.123361 -3.519930 -0.732839
H 0.266656 -3.258343 -2.475184
H 1.592048 -2.778296 -1.406376
C -1.748551 2.026457 1.850520
H -1.450863 2.753675 2.615362
H -2.386705 2.525464 1.119025
H -2.331288 1.232289 2.325615
C -1.343678 2.741442 -1.304166
H -0.828429 3.419731 -0.612124
H -1.275616 3.173706 -2.308500
H -2.403749 2.695110 -1.029492
54
TS-I-Anti_Et_conf_13 Eopt -1028.028996
C -3.110174 1.544144 1.285822
C -2.638106 0.057549 1.313791
C -2.629516 0.031439 -1.292663
C -3.060283 1.537400 -1.279448
H -2.685294 2.069355 2.151197
H -4.144749 1.602325 -1.447367
C -3.839427 -0.928165 1.264032
H -4.449541 -0.784361 2.167270
C -3.880881 -0.888402 -1.269420
H -3.572077 -1.936205 -1.387688
H -2.107819 -0.124707 2.253177
H -2.090133 -0.174068 -2.223976
C -4.698172 -0.749817 0.013297
H -5.509263 -1.490193 0.013780
H -5.184719 0.235919 0.038301
C -2.703520 2.288094 0.009591
H -1.613135 2.459064 0.034365
H -3.161583 3.285907 -0.005503
H -3.464657 -1.964275 1.301604
H -4.503600 -0.647765 -2.143099
H -4.201570 1.589653 1.407015
H -2.588153 2.049597 -2.128183
B -1.796816 -0.239589 0.017381
C -0.211727 -0.818566 0.053970
C 0.501564 0.472906 0.054463
C 0.568364 1.177418 -1.277613
H -0.474638 1.216260 -1.635480
C 0.628530 1.146428 1.390315
H -0.413428 1.229211 1.753841
C 0.023325 -1.673941 -1.212230
H 1.105881 -1.849342 -1.294484
H 1.088095 0.415830 2.065018
C -0.151070 -1.678860 1.340853
H -0.358371 -1.066258 2.220449
H -0.987148 -2.388731 1.284953
I 3.416210 -0.145603 -0.141333
H 1.055863 0.489750 -1.974222
H -0.266933 -1.112004 -2.103141
C -0.717907 -3.007299 -1.217986
H -1.789877 -2.879681 -1.017766
H -0.617826 -3.482206 -2.201063
H -0.322574 -3.705684 -0.471463
C 1.142236 -2.456857 1.577731
H 1.478661 -2.995144 0.683726
H 1.958573 -1.797876 1.889360
H 0.975843 -3.192483 2.375168
C 1.179587 2.561343 -1.432885
H 2.204957 2.599494 -1.052965
H 1.209975 2.793323 -2.503679
H 0.584236 3.339563 -0.945538
C 1.314446 2.492056 1.571912
H 1.380177 2.689104 2.648057
H 2.332214 2.484443 1.169740
H 0.753945 3.315850 1.121155
54
TS-I-Anti_Et_conf_14 Eopt -1028.029458
C -3.224981 0.116054 -1.990101
C -2.387833 0.935225 -0.951291
C -2.742958 -0.954336 0.793523
C -3.660289 -1.707097 -0.221094
H -2.766570 0.227264 -2.981438
H -4.711739 -1.481024 0.001545
C -3.333646 1.846012 -0.118697
H -3.884191 2.489489 -0.820018
C -3.588932 0.047279 1.631282
H -2.946468 0.579985 2.348112
H -1.735555 1.616399 -1.503315
H -2.306642 -1.682445 1.483624
C -4.310800 1.071014 0.758598
H -4.868939 1.771650 1.393986
H -5.058306 0.569090 0.128265
C -3.363618 -1.382535 -1.688508
H -2.429403 -1.888705 -1.971171
H -4.144540 -1.812490 -2.329857
H -2.741860 2.517782 0.515192
H -4.314522 -0.525762 2.227012
H -4.225767 0.566423 -2.064104
H -3.551571 -2.788910 -0.073107
B -1.662453 -0.084644 0.022533
C -0.051831 -0.439057 0.208018
C 0.776258 0.731800 -0.137785
C 0.950425 1.115949 -1.577976
H -0.065696 1.224701 -1.985174
C 1.075373 1.778244 0.888052
H 1.283917 1.326845 1.861183
C 0.060859 -1.525632 -0.928673
H 1.125510 -1.571050 -1.193609
H 1.967080 2.330496 0.583185
C 0.231948 -1.025136 1.613826
H -0.134533 -2.054333 1.617696
H 1.320386 -1.095867 1.714658
I 3.506967 -0.386770 0.037968
H 1.359651 0.257603 -2.119645
H -0.453122 -1.167198 -1.831958
C -0.395039 -2.944086 -0.602844
H 0.309721 -3.439523 0.074096
H -1.391416 -2.993248 -0.151933
H -0.419076 -3.527038 -1.531808
C -0.358332 -0.315550 2.844842
H -0.637052 0.724655 2.654558
H -1.255504 -0.826411 3.210956
H 0.369874 -0.310360 3.664766
C 1.765124 2.362666 -1.885517

H 1.323986 3.267361 -1.450299
H 2.792822 2.259575 -1.515905
H 1.808210 2.501514 -2.971413
C -0.105388 2.759214 0.996449
H -0.426188 3.129229 0.014308
H -0.968398 2.288798 1.479320
H 0.200858 3.617358 1.604696
54
TS-I-Anti_Et_conf_15 Eopt -1028.029965
C 3.545076 0.419598 -1.580101
C 2.730090 -0.796441 -1.058797
C 2.416956 0.431788 1.210905
C 3.227782 1.637391 0.661896
H 2.878697 1.028728 -2.209308
H 3.797409 2.085546 1.488686
C 3.585619 -1.873256 -0.351896
H 4.363684 -2.224972 -1.045387
C 3.281184 -0.659650 1.891188
H 2.593059 -1.376297 2.366429
H 2.277862 -1.260209 -1.938944
H 1.747802 0.828094 1.979510
C 4.231219 -1.432466 0.967447
H 4.587060 -2.324661 1.500030
H 5.126090 -0.835796 0.764813
C 4.171197 1.317063 -0.504696
H 4.481385 2.260756 -0.973845
H 5.091031 0.861084 -0.126214
H 2.935536 -2.740379 -0.154785
H 3.855655 -0.199274 2.708321
H 4.330091 0.044377 -2.252906
H 2.512254 2.406776 0.331058
B 1.700159 -0.205868 -0.026980
C 0.048188 -0.376126 -0.308122
C -0.416041 0.961649 -0.161204
C -0.446250 1.741065 1.115957
H 0.505763 2.299633 1.118109
C -0.737782 1.781836 -1.371805
H -0.833038 1.176082 -2.270608
C -0.059010 -1.461164 0.829540
H 0.830494 -2.112957 0.775260
H -1.718513 2.239278 -1.187845
C -0.221676 -1.015956 -1.693955
H -1.276697 -0.835860 -1.933788
H 0.369569 -0.486513 -2.449736
I -3.645925 -0.399216 0.024629
H -0.414779 1.088279 1.981566
H -0.879389 -2.087433 0.458519
C -0.356477 -1.156154 2.288908
H 0.416825 -0.564245 2.790048
H -1.328864 -0.665315 2.404888
H -0.414577 -2.120468 2.808582
C 0.031986 -2.518609 -1.827417
H 0.039863 -2.781386 -2.891876
H 0.987116 -2.842306 -1.398225
H -0.764979 -3.105196 -1.356707
C -1.581200 2.758332 1.261590
H -2.557006 2.272776 1.152409
H -1.522778 3.197572 2.263334
H -1.507308 3.574659 0.535137
C 0.304807 2.889159 -1.601079
H 1.300833 2.459070 -1.762644
H 0.029095 3.454281 -2.497718
H 0.359994 3.585733 -0.756739
54
TS-I-Anti_Et_conf_16 Eopt -1028.029659
C -3.889195 -1.210810 -0.918544
C -2.716934 -0.244042 -1.253387
C -2.547663 0.351948 1.279082
C -3.677934 -0.683407 1.546288
H -3.508213 -2.236240 -0.812470
H -4.231826 -0.371203 2.443193
C -3.253174 1.200691 -1.524234
H -4.347730 1.164967 -1.623396
C -3.137079 1.762400 0.978899
H -2.697225 2.488666 1.674988
H -2.229460 -0.620421 -2.159122
H -1.943262 0.409294 2.188183
C -2.877671 2.234604 -0.454378
H -1.804842 2.473651 -0.548199
H -3.413520 3.173993 -0.644880
C -4.630366 -0.833416 0.361742
H -5.383451 -1.599989 0.588016
H -5.184721 0.103675 0.209462
H -2.868499 1.544554 -2.493201
H -4.217544 1.764966 1.179795
H -4.582436 -1.223455 -1.771872
H -3.230927 -1.663430 1.778712
B -1.785089 -0.198922 0.014316
C -0.201901 -0.776992 0.033815
C 0.493314 0.519436 0.060923
C 0.572601 1.243979 -1.263694
H -0.457551 1.211171 -1.664030
C 0.479804 1.324526 1.327695
H -0.349384 2.036641 1.142170
C 0.098436 -1.597098 -1.240678
H 1.188009 -1.724870 -1.294753
H 0.168028 0.693044 2.159879
C -0.202587 -1.664533 1.306282
H -0.540735 -1.102160 2.180419
H -0.969905 -2.434503 1.149289
I 3.395804 -0.151777 -0.169387
H 1.147808 0.636874 -1.966938
H -0.185195 -1.021189 -2.125950
C -0.570807 -2.964045 -1.319990
H -1.658210 -2.898865 -1.199175
H -0.374218 -3.408639 -2.303030
H -0.186306 -3.657779 -0.562724
C 1.118650 -2.350273 1.641500
H 1.855586 -1.631734 2.015362
H 0.946872 -3.102687 2.421757
H 1.559458 -2.852432 0.771795
C 1.065587 2.680549 -1.292879
H 0.886399 3.090936 -2.293091
H 0.542027 3.318995 -0.569720
H 2.142416 2.732926 -1.097997
C 1.693942 2.131705 1.790146
H 1.365641 2.812257 2.585416
H 2.453169 1.459628 2.199396
H 2.155432 2.720822 0.997144
54
TS-I-Anti_Et_conf_2 Eopt -1028.035072
C -3.676791 1.006220 1.327816
C -2.786874 -0.252999 1.177623
C -2.369472 0.366786 -1.304333
C -3.063629 1.726572 -1.048065
H -3.108466 1.753930 1.898173
H -3.491762 2.093546 -1.992072
C -3.595072 -1.501903 0.723096
H -4.370051 -1.705541 1.476163
C -3.357967 -0.748167 -1.752057
H -2.759848 -1.545945 -2.212571
H -2.374636 -0.483497 2.166676
H -1.667009 0.501363 -2.135091
C -4.253409 -1.362543 -0.657300
H -4.579127 -2.356765 -0.992546
H -5.171782 -0.775883 -0.552372
C -4.150826 1.666546 0.024881
H -4.487501 2.687499 0.251221
H -5.027945 1.149148 -0.377581
H -2.930590 -2.377960 0.732818
H -3.992397 -0.346248 -2.556391
H -4.547600 0.748818 1.949725
H -2.304798 2.467853 -0.745666
B -1.706028 -0.088304 0.046385
C -0.102548 -0.586557 0.208304
C 0.662098 0.670005 0.173478
C 0.697507 1.489659 -1.084994
H -0.356942 1.734111 -1.286807
C 0.963440 1.385141 1.450417
H 1.293973 0.687477 2.223596

C 0.139814 -1.484073 -1.044628
H 1.226415 -1.617193 -1.128842
H 1.766827 2.104858 1.282226
C -0.020101 -1.387499 1.526362
H -0.264338 -0.730707 2.368331
H -0.829697 -2.126937 1.499080
I 3.539488 -0.126113 -0.230694
H 1.005448 0.856162 -1.922626
H -0.180064 -0.956919 -1.948726
C -0.526192 -2.856236 -1.002243
H -0.113743 -3.489102 -0.207987
H -1.610829 -2.796976 -0.853797
H -0.353368 -3.369055 -1.956089
C 1.289407 -2.114448 1.820784
H 1.133804 -2.809515 2.656092
H 1.646149 -2.693448 0.960349
H 2.090396 -1.423142 2.100947
C 1.477454 2.795914 -1.062380
H 2.533427 2.629620 -0.822054
H 1.421483 3.259135 -2.053634
H 1.057319 3.506691 -0.339623
C -0.304919 2.123169 1.919167
H -1.042805 1.416324 2.312677
H -0.039422 2.820490 2.721111
H -0.764758 2.699819 1.104558
54
TS-I-Anti_Et_conf_3 Eopt -1028.035406
C -2.944192 0.470082 -2.026971
C -2.348658 1.081162 -0.724581
C -2.724316 -1.209678 0.453737
C -3.320912 -1.761567 -0.873800
H -2.129527 0.098955 -2.668935
H -4.085989 -2.511279 -0.624794
C -3.474150 1.693746 0.162892
H -4.416190 1.719780 -0.403412
C -3.842282 -0.556533 1.322426
H -3.842074 -1.021581 2.316651
H -1.683852 1.894167 -1.025899
H -2.299142 -2.052713 1.006830
C -3.691884 0.957406 1.486118
H -2.826620 1.153208 2.139276
H -4.568484 1.369052 2.004090
C -3.928099 -0.665866 -1.749422
H -4.267720 -1.099388 -2.699363
H -4.825427 -0.259524 -1.261461
H -3.221507 2.739705 0.380911
H -4.825264 -0.779636 0.882991
H -3.444050 1.271794 -2.589213
H -2.544186 -2.288081 -1.446908
B -1.649784 -0.129625 0.016884
C -0.044362 -0.486403 0.197571
C 0.721466 0.727548 -0.113825
C 0.861487 1.153904 -1.546421
H -0.159735 1.196113 -1.956262
C 1.008880 1.743051 0.946921
H 1.267755 1.247027 1.885346
C 0.114521 -1.550751 -0.958799
H 1.139635 -1.448914 -1.333584
H 1.874426 2.335988 0.640868
C 0.200855 -1.067335 1.615026
H -0.070607 -2.125406 1.586781
H 1.281615 -1.037316 1.802666
I 3.498959 -0.339880 0.020507
H 1.332400 0.337249 -2.102453
H -0.534332 -1.287880 -1.807031
C -0.080705 -3.020310 -0.603912
H -0.049039 -3.607325 -1.530128
H 0.732891 -3.377641 0.038065
H -1.031135 -3.235041 -0.105274
C -0.545283 -0.456805 2.803475
H -1.620740 -0.662149 2.756534
H -0.171878 -0.916066 3.727339
H -0.410969 0.624956 2.905028
C 1.596371 2.455112 -1.826909
H 1.107387 3.321104 -1.365489
H 2.631760 2.402289 -1.467775
H 1.623041 2.624319 -2.908973
C -0.182226 2.694922 1.163613
H 0.099865 3.426405 1.928936
H -0.444273 3.237640 0.248424
H -1.073017 2.161865 1.512104
54
TS-I-Anti_Et_conf_4 Eopt -1028.033940
C -3.674027 1.128129 1.166682
C -2.834010 -0.184062 1.152884
C -2.371305 0.195114 -1.385376
C -3.207361 1.508770 -1.322779
H -3.559537 1.613090 2.144955
H -4.225121 1.314527 -1.690715
C -3.712161 -1.412494 0.765883
H -4.504652 -1.521314 1.520028
C -3.296852 -1.011062 -1.720163
H -2.690155 -1.909171 -1.895595
H -2.442600 -0.370989 2.157465
H -1.644819 0.274818 -2.199924
C -4.331576 -1.284928 -0.627242
H -4.887068 -2.201289 -0.867878
H -5.073430 -0.473386 -0.619774
C -3.276775 2.125333 0.075013
H -2.283493 2.529366 0.321806
H -3.966821 2.979848 0.076265
H -3.106616 -2.329992 0.819666
H -3.806570 -0.793762 -2.669720
H -4.742232 0.885728 1.070847
H -2.770416 2.243614 -2.012175
B -1.738890 -0.131701 0.025568
C -0.140430 -0.599662 0.275187
C 0.598747 0.672057 0.205796
C 0.547221 1.502281 -1.041111
H -0.506354 1.838872 -1.081811
C 0.979219 1.384999 1.465707
H 1.303151 0.676741 2.230017
C 0.145250 -1.573271 -0.914739
H 1.234215 -1.681780 -0.978166
H 1.816895 2.054588 1.256279
C -0.070736 -1.328980 1.634341
H -0.335290 -0.631052 2.436846
H -0.870694 -2.080367 1.636084
I 3.489335 -0.152407 -0.298992
H 0.677856 0.862993 -1.920043
H -0.176872 -1.123307 -1.858366
C -0.479500 -2.959642 -0.767296
H -0.372626 -3.506583 -1.711787
H 0.016003 -3.548167 0.012693
H -1.547229 -2.924736 -0.523872
C 1.248271 -2.018345 1.980268
H 1.102686 -2.653781 2.863359
H 1.613017 -2.652027 1.163465
H 2.044004 -1.301740 2.208183
C 1.417687 2.748342 -1.120894
H 1.155434 3.479793 -0.347578
H 2.479878 2.499699 -1.026815
H 1.260945 3.226145 -2.094329
C -0.202894 2.217985 1.992703
H -0.460321 3.023598 1.295387
H -1.093558 1.601773 2.168491
H 0.089501 2.670945 2.946247
54
TS-I-Anti_Et_conf_5 Eopt -1028.032619
C -3.028455 1.808814 1.003484
C -2.599136 0.340170 1.271966
C -2.657438 -0.078553 -1.304251
C -3.133145 1.389463 -1.502042
H -2.129666 2.444979 0.940910
H -3.804516 1.417577 -2.372405
C -3.833107 -0.602993 1.432226
H -4.752095 -0.001999 1.493904
C -3.890193 -1.018594 -1.097353
H -3.856284 -1.826390 -1.839356
H -2.033971 0.330036 2.209424

H	-2.119028	-0.381009	-2.209372
C	-3.964716	-1.632771	0.304403
H	-3.147269	-2.366696	0.408154
H	-4.900294	-2.196416	0.417663
C	-3.848863	1.957072	-0.276980
H	-4.081643	3.016892	-0.446241
H	-4.815995	1.450162	-0.148813
H	-3.748622	-1.137259	2.386878
H	-4.813131	-0.456380	-1.298502
H	-3.606350	2.170578	1.865939
H	-2.275685	2.030012	-1.750406
B	-1.787120	-0.126516	0.008809
C	-0.201908	-0.691876	0.090649
C	0.484095	0.598301	0.183166
C	0.323728	1.551443	-0.970516
H	-0.358184	2.310357	-0.539644
C	0.756135	1.170828	1.537175
H	-0.253382	1.251655	1.983642
C	0.120507	-1.497557	-1.188593
H	1.181738	-1.767601	-1.130804
H	1.274078	0.419741	2.142921
C	-0.217871	-1.587484	1.352057
H	-0.428915	-0.981138	2.237269
H	-1.076396	-2.267721	1.256582
I	3.431180	-0.186039	-0.170574
H	-0.226086	1.059333	-1.776572
H	0.021153	-0.861008	-2.073748
C	-0.708846	-2.763633	-1.376243
H	-1.786125	-2.562106	-1.402063
H	-0.439316	-3.236706	-2.328200
H	-0.520769	-3.496245	-0.581869
C	1.033822	-2.424016	1.610304
H	1.323277	-3.020337	0.736814
H	1.890564	-1.799217	1.881819
H	0.837167	-3.114507	2.440292
C	1.503860	2.297072	-1.592927
H	2.165043	2.747508	-0.850892
H	2.095707	1.611363	-2.205616
H	1.104275	3.089832	-2.237194
C	1.445213	2.521624	1.643487
H	2.481823	2.462766	1.293723
H	0.921161	3.301326	1.076594
H	1.458691	2.827384	2.695503

54

TS-I-Anti_Et_conf_6 Eopt -1028.031079

C	-3.753714	0.639486	1.506042
C	-2.564516	-0.323184	1.254885
C	-2.673114	0.261588	-1.271435
C	-3.868803	1.211008	-0.987474
H	-3.337654	1.599251	1.851459
H	-4.546569	1.190529	-1.853617
C	-2.980726	-1.782443	0.938135
H	-3.602205	-2.164187	1.761654
C	-3.137492	-1.191569	-1.578553
H	-2.281513	-1.747123	-1.984089
H	-1.989078	-0.346984	2.187835
H	-2.155774	0.635765	-2.164341
C	-3.721963	-1.977937	-0.393142
H	-3.709246	-3.046946	-0.645874
H	-4.778399	-1.724975	-0.262853
C	-4.653586	0.906073	0.294040
H	-5.300186	1.764906	0.520476
H	-5.331721	0.062414	0.133395
H	-2.067666	-2.399793	0.938505
H	-3.881817	-1.152033	-2.387742
H	-4.357327	0.250519	2.339328
H	-3.494347	2.242012	-0.920287
B	-1.769961	0.219866	0.014812
C	-0.173916	0.750849	0.092576
C	0.484607	-0.556831	0.177347
C	0.744971	-1.147548	1.525025
H	-0.260923	-1.196517	1.981034
C	0.295939	-1.487286	-0.989426
H	-0.427694	-2.215830	-0.573663
C	-0.175367	1.635772	1.361204
H	-0.434365	1.033968	2.237681
H	-0.221469	-0.960940	-1.794871
C	0.188563	1.555806	-1.176912
H	1.268137	1.742388	-1.137094
H	0.020774	0.948859	-2.072367
I	3.452374	0.132961	-0.166974
H	1.290801	-0.417404	2.131848
H	-0.996574	2.358721	1.248004
C	1.111617	2.407002	1.651575
H	1.453035	2.986634	0.785869
H	0.932517	3.105810	2.478514
H	1.929131	1.738582	1.940810
C	-0.539257	2.886444	-1.327810
H	-0.256620	3.598880	-0.543718
H	-0.274769	3.338010	-2.291610
H	-1.628098	2.773756	-1.303999
C	1.387161	-2.521862	1.619888
H	0.833937	-3.277919	1.048688
H	2.424345	-2.497787	1.267897
H	1.392017	-2.835479	2.669682
C	1.445440	-2.281567	-1.608154
H	2.079269	-2.768102	-0.865178
H	1.016104	-3.049852	-2.262821
H	2.072868	-1.618189	-2.209838

54

TS-I-Anti_Et_conf_7 Eopt -1028.035725

C	3.870484	1.056024	-1.012769
C	2.605597	0.175673	-1.268964
C	2.566921	-0.425078	1.269341
C	3.835991	0.458224	1.475002
H	3.852412	1.915508	-1.694902
H	4.734953	-0.175439	1.468438
C	3.015493	-1.294851	-1.576067
H	3.663631	-1.292070	-2.464331
C	2.939147	-1.887364	0.895006
H	2.019711	-2.489776	0.808587
H	2.071057	0.564975	-2.141554
H	2.017454	-0.459268	2.215221
C	3.731203	-1.974215	-0.409216
H	3.919907	-3.027105	-0.657387
H	4.718951	-1.513894	-0.265309
C	3.980147	1.566220	0.427455
H	3.182924	2.310315	0.597006
H	4.931850	2.095944	0.567198
H	2.125408	-1.881352	-1.844457
H	3.520456	-2.324694	1.719381
H	4.773264	0.481473	-1.265639
H	3.790015	0.917569	2.470396
B	1.746698	0.134623	0.050104
C	0.164699	0.687441	0.172517
C	-0.522600	-0.604270	0.249722
C	-0.549470	-1.610909	-0.859813
H	-1.392174	-2.278803	-0.666460
C	-0.759530	-1.187849	1.614343
H	0.241424	-1.242989	2.075733
C	-0.165158	1.584804	-1.043301
H	-1.231758	1.831773	-0.980147
H	-1.302172	-0.446800	2.211213
C	0.190914	1.529333	1.475770
H	0.438292	0.901950	2.335344
H	1.024776	2.239755	1.389518
I	-3.453987	0.051895	-0.216880
H	0.341878	-2.229756	-0.619510
H	-0.035526	1.035506	-1.973305
C	0.648957	2.873946	-1.123145
H	0.405668	3.394783	-2.057037
H	0.426567	3.561167	-0.298524
H	1.730150	2.685744	-1.124829
C	-1.087821	2.308980	1.778241
H	-0.929311	2.929824	2.668966
H	-1.374880	2.971178	0.952914
H	-1.935294	1.642812	1.974124
C	-0.537476	-1.252817	-2.337076
H	0.387173	-0.766088	-2.659526
H	-0.636681	-2.183956	-2.906934

H -1.390537 -0.611669 -2.587337
C -1.423688 -2.551579 1.722252
H -0.815317 -3.345260 1.272107
H -1.556855 -2.794949 2.782007
H -2.412433 -2.550247 1.247348
54
TS-I-Anti_Et_conf_8 Eopt -1028.032877
C -2.917877 0.554370 -1.959187
C -2.363608 1.109045 -0.616774
C -2.706572 -1.215803 0.474004
C -3.260458 -1.763717 -0.873203
H -2.060269 0.322408 -2.610631
H -4.056608 -2.489984 -0.651907
C -3.458561 1.675603 0.320756
H -3.989278 2.482123 -0.206762
C -3.830797 -0.635068 1.365656
H -3.422416 -0.458187 2.370163
H -1.711235 1.953238 -0.865600
H -2.277676 -2.062092 1.022269
C -4.473099 0.662050 0.859856
H -5.025206 1.127882 1.687606
H -5.225356 0.440678 0.096743
C -3.797821 -0.699302 -1.843202
H -3.891793 -1.151449 -2.839819
H -4.814071 -0.412886 -1.557618
H -2.957723 2.154147 1.175698
H -4.607615 -1.404263 1.491107
H -3.479563 1.351704 -2.467935
H -2.466169 -2.330612 -1.377843
B -1.643722 -0.107134 0.080681
C -0.028436 -0.435113 0.250157
C 0.753261 0.764575 -0.080151
C 0.850645 1.196839 -1.512977
H -0.187173 1.287093 -1.870479
C 1.117444 1.764766 0.968959
H 1.429321 1.255271 1.882180
C 0.127141 -1.518177 -0.891183
H 1.134885 -1.396571 -1.301839
H 1.969700 2.350682 0.616773
C 0.183560 -0.990964 1.678908
H -0.215819 -2.007158 1.683817
H 1.262127 -1.084048 1.864282
I 3.511369 -0.380382 -0.016715
H 1.259329 0.368225 -2.099790
H -0.556780 -1.295150 -1.723859
C -0.005878 -2.982337 -0.492125
H 0.075445 -3.595272 -1.398316
H 0.810430 -3.276287 0.178389
H -0.955888 -3.229184 -0.007737
C -0.486451 -0.237937 2.836370
H 0.095585 0.614763 3.199415
H -1.480794 0.141509 2.565843
H -0.625218 -0.917557 3.685611
C 1.619207 2.473127 -1.816748
H 1.180819 3.350534 -1.326631
H 2.667048 2.380762 -1.504684
H 1.603163 2.651333 -2.897544
C -0.049922 2.722453 1.266755
H -0.911169 2.188754 1.679609
H 0.288603 3.455561 2.007253
H -0.373260 3.263485 0.369505
54
TS-I-Anti_Et_conf_9 Eopt -1028.029488
C -3.663905 -1.071355 1.354378
C -2.775015 -1.373282 0.121761
C -2.438593 1.172303 -0.191473
C -3.536305 1.438595 0.872567
H -3.025932 -1.050934 2.253558
H -4.161628 2.275657 0.526823
C -3.555770 -1.438697 -1.215366
H -4.423579 -2.104005 -1.088663
C -3.022932 1.051881 -1.626430
H -2.197630 0.928459 -2.345207
H -2.333065 -2.363962 0.277294
H -1.783098 2.049438 -0.188534
C -4.023216 -0.095282 -1.803677
H -4.218460 -0.231628 -2.876073
H -4.983092 0.198415 -1.366662
C -4.429768 0.248029 1.253844
H -4.903130 0.461797 2.221847
H -5.253454 0.135399 0.541399
H -2.901469 -1.931608 -1.948580
H -3.506826 2.004410 -1.885900
H -4.369941 -1.902272 1.499532
H -3.036845 1.793395 1.784520
B -1.714033 -0.205556 0.072098
C -0.113534 -0.598387 0.282748
C 0.668059 0.647360 0.176479
C 0.708869 1.389479 -1.132274
H -0.332500 1.463724 -1.476050
C 1.011844 1.407313 1.422451
H 1.382134 0.719578 2.185350
C 0.179061 -1.701395 -0.778808
H -0.433420 -2.558155 -0.476421
H 1.812391 2.116508 1.201198
C -0.107081 -1.260739 1.695144
H -0.407924 -0.528684 2.455331
H -0.891034 -2.025640 1.692586
I 3.505242 -0.216912 -0.182279
H 1.195541 0.756740 -1.875823
H 1.223563 -2.002120 -0.642060
C -0.063040 -1.428831 -2.265626
H -0.230195 -2.381690 -2.782084
H -0.941273 -0.798936 -2.454417
H 0.800686 -0.957753 -2.746117
C 1.186437 -1.943120 2.140283
H 1.506027 -2.711812 1.426851
H 2.018635 -1.244259 2.267189
H 1.008141 -2.436579 3.104338
C 1.347586 2.769989 -1.132958
H 2.406550 2.715817 -0.852718
H 1.288276 3.187947 -2.144015
H 0.837177 3.463056 -0.453494
C -0.191207 2.178958 1.994442
H -1.014611 1.504496 2.252345
H 0.137217 2.684001 2.909333
H -0.563812 2.938409 1.298700
42
TS-I-Anti_Me_conf_1 Eopt -870.977362
C 3.156533 1.101204 -1.533661
C 2.401071 -0.232750 -1.315850
C 2.424039 0.203255 1.254058
C 3.112614 1.566021 0.997534
H 2.413830 1.846489 -1.857304
H 3.772867 1.801338 1.845120
C 3.326130 -1.465003 -1.093389
H 3.971631 -1.586758 -1.975267
C 3.405440 -0.990919 1.435199
H 2.815556 -1.853750 1.781633
H 1.801960 -0.431177 -2.212374
H 1.847669 0.287834 2.184097
C 4.184489 -1.407084 0.177593
H 4.616989 -2.401816 0.351009
H 5.037170 -0.739531 0.020341
C 3.904007 1.657395 -0.312819
H 4.144544 2.712291 -0.503251
H 4.869235 1.154767 -0.197432
H 2.692629 -2.365457 -1.050923
H 4.107191 -0.752699 2.247866
H 3.858423 0.983624 -2.372699
H 2.330909 2.343203 0.994555
B 1.572832 -0.160074 0.001410
C -0.051538 -0.669872 0.066144
C -0.545552 0.670739 0.155853
C -0.754100 1.334728 1.470323
H -1.398689 2.210592 1.353806
H -1.162976 0.662878 2.227503
C -0.510476 1.594430 -1.010469
H 0.302480 2.317501 -0.814999
H -1.434549 2.180691 -1.045170

C -0.192439 -1.546817 1.309564
H 0.338090 -2.494364 1.157637
H -1.259637 -1.775315 1.447823
H -0.337083 1.107968 -1.971124
C -0.360067 -1.475309 -1.195158
H -1.338825 -1.948773 -1.045157
H -0.410035 -0.873772 -2.104957
H 0.388251 -2.264027 -1.337488
I -3.608483 -0.037345 -0.067910
H 0.229352 1.697518 1.817344
H 0.187567 -1.079680 2.222054
42
TS-I-Anti_Me_conf_2 Eopt -870.976718
C -3.472663 1.075497 1.295844
C -2.471815 -0.128888 1.277085
C -2.386227 0.033385 -1.338816
C -3.388039 1.237067 -1.272857
H -3.290635 1.674983 2.196362
H -4.406804 0.870055 -1.463408
C -3.198226 -1.488806 1.119832
H -3.860461 -1.639862 1.984454
C -3.139708 -1.318578 -1.412077
H -2.411085 -2.134261 -1.534631
H -1.918186 -0.148917 2.221871
H -1.766275 0.129982 -2.235890
C -3.997701 -1.590603 -0.177787
H -4.448134 -2.589118 -0.255452
H -4.836323 -0.879751 -0.147849
C -3.353501 1.988509 0.065857
H -2.396882 2.536716 0.128912
H -4.143296 2.750599 0.088265
H -2.452781 -2.300475 1.150629
H -3.768244 -1.322113 -2.314386
H -4.500931 0.694045 1.374564
H -3.153520 1.942590 -2.079348
B -1.591285 0.100267 0.008174
C 0.024398 0.625965 0.081532
C 0.556557 -0.701947 0.177055
C 0.746344 -1.344861 1.506340
H 1.323372 -2.268756 1.411644
H 1.215095 -0.679163 2.234080
C 0.537148 -1.643756 -0.975205
H -0.271684 -2.368815 -0.771946
H 1.464702 -2.224251 -0.994047
C 0.178466 1.514931 1.314392
H -0.377194 2.449231 1.170978
H 1.243586 1.766016 1.423618
H 0.366532 -1.172546 -1.944030
C 0.290096 1.431452 -1.190805
H 1.243445 1.957527 -1.052327
H 0.366520 0.820456 -2.092555
H -0.500946 2.178020 -1.340519
I 3.549266 0.035551 -0.078208
H -0.258039 -1.611496 1.883089
H -0.167254 1.047556 2.239742
54
TS-I-Gauche_Et_conf_1 Eopt -1028.018385
C 0.387753 1.153334 0.845808
C -0.880076 1.372533 0.087512
I -2.365938 -1.343898 -0.172284
C 0.233912 0.823185 2.357208
H 1.254760 0.828348 2.761443
H -0.274666 1.665347 2.849327
C -2.063414 1.879520 0.841822
H -1.655854 2.777079 1.346186
H -2.287494 1.192551 1.662988
C 1.248518 -0.921085 -0.951857
C 2.948893 0.099490 0.736186
H 0.252285 -0.893152 -1.401992
C 1.358932 -2.271431 -0.180711
C 2.326495 -0.845933 -2.069409
H 3.166137 0.875979 1.481402
C 3.021937 -1.275294 1.461445
C 3.996841 0.181014 -0.414497
H 1.197804 -3.089530 -0.898112
H 0.544414 -2.347153 0.552342
C 2.702607 -2.444457 0.528467
H 1.835159 -0.639060 -3.029567
H 2.813959 -1.825513 -2.184443
C 3.385720 0.229219 -1.820928
H 2.317310 -1.284904 2.307722
H 4.027682 -1.397176 1.890482
H 4.618572 1.075161 -0.271681
H 4.684048 -0.675551 -0.349738
H 2.698074 -3.382005 1.100776
H 2.908466 1.210347 -1.969609
B 1.476230 0.165316 0.169269
H 4.182632 0.158054 -2.573980
H 3.503921 -2.546243 -0.217732
C 1.049649 2.623861 0.882677
H 0.260345 3.343715 1.131045
H 1.698946 2.574455 1.763372
C -0.718150 1.696928 -1.359896
H -0.811572 2.802978 -1.327907
H 0.308506 1.480469 -1.668370
C -3.326409 2.275323 0.097071
H -3.828167 1.397641 -0.325068
H -4.014508 2.752262 0.804016
H -3.121407 2.993034 -0.707171
C -0.430253 -0.482520 2.776459
H -0.007130 -1.344953 2.249564
H -0.265608 -0.632847 3.851252
H -1.509373 -0.491758 2.597734
C 1.889215 3.166427 -0.264239
H 2.781303 2.556218 -0.436212
H 1.347025 3.280231 -1.208028
H 2.238444 4.166046 0.025727
C -1.664802 1.182783 -2.443355
H -1.453655 0.128881 -2.648401
H -2.718265 1.271064 -2.175098
H -1.482755 1.762597 -3.356155
54
TS-I-Gauche_Et_conf_10 Eopt -1028.018215
C -0.647300 1.390434 -0.247155
C 0.521091 1.577088 0.624959
I 2.698355 -1.335425 -0.497105
C -0.325721 1.507071 -1.755601
H 0.353030 2.342882 -1.960504
H 0.214822 0.596729 -2.042232
C 1.491991 2.661378 0.376767
H 1.502754 3.232985 1.321963
H 1.140326 3.313912 -0.421136
C -1.190059 -1.370940 -0.645894
C -2.676704 -0.095490 1.052378
H -0.323281 -1.358399 -1.316902
C -2.439643 -1.719289 -1.503936
C -0.920339 -2.442278 0.439540
H -2.865560 0.813975 1.636214
C -3.875636 -0.255064 0.072020
C -2.519113 -1.258026 2.057558
H -2.359402 -2.769194 -1.824207
H -2.396442 -1.123212 -2.425164
C -3.806754 -1.494483 -0.829704
H 0.020853 -2.197584 0.953228
H -0.740538 -3.407975 -0.056137
C -2.038500 -2.595103 1.474199
H -3.933956 0.645318 -0.562199
H -4.810033 -0.273434 0.652445
H -1.789131 -0.935011 2.816470
H -3.468517 -1.408295 2.594592
H -4.572998 -1.400434 -1.611670
H -1.675298 -3.225928 2.297344
B -1.448925 0.007694 0.073797
H -2.877015 -3.144439 1.033443
H -4.092040 -2.376439 -0.247401
C -1.591192 2.607408 0.212030
H -2.550917 2.348659 -0.250741
H -1.751605 2.547143 1.292983
C 0.560844 0.816773 1.893198
H -0.401199 1.073655 2.380261

H 0.440493 -0.246884 1.628968
C 2.946826 2.263801 0.058495
H 3.536369 3.186646 0.033233
H 3.371467 1.586823 0.801373
H 2.995301 1.776645 -0.919351
C -1.581779 1.663495 -2.609533
H -2.006876 2.671434 -2.528131
H -1.347647 1.482740 -3.664706
H -2.364923 0.952361 -2.316169
C -1.224346 4.035752 -0.171440
H -0.862963 4.121605 -1.203373
H -2.128073 4.652063 -0.087184
H -0.474389 4.472934 0.496149
C 1.720124 0.993393 2.857816
H 1.471309 0.501696 3.804033
H 2.627764 0.522163 2.465983
H 1.927359 2.048377 3.071928
54
TS-I-Gauche_Et_conf_11 Eopt -1028.020435
C 0.377062 1.166511 0.658944
C -0.822817 1.367407 -0.206210
I -2.626595 -1.435506 0.150726
C -0.024404 0.606200 2.059562
H -0.940852 1.098949 2.409072
H -0.283111 -0.451013 1.928425
C -1.881377 2.299858 0.249420
H -1.645944 3.173586 -0.394928
H -1.694647 2.613458 1.278140
C 3.026201 0.553522 -0.173701
C 1.289044 -1.364772 -0.175820
H 3.265249 1.606040 0.021945
C 3.229913 0.304528 -1.692114
C 3.965865 -0.293923 0.722575
H 0.242369 -1.666081 -0.055475
C 1.745487 -1.790656 -1.593095
C 2.102842 -2.059697 0.949142
H 4.232034 0.652367 -1.983894
H 2.516130 0.927431 -2.258200
C 3.052005 -1.158578 -2.105719
H 3.965749 0.167169 1.720970
H 4.998376 -0.200938 0.351087
C 3.608004 -1.783417 0.881599
H 0.941555 -1.531010 -2.298091
H 1.827008 -2.887827 -1.630466
H 1.712542 -1.740017 1.928148
H 1.925291 -3.143839 0.894171
H 3.070145 -1.223801 -3.202278
H 4.082470 -2.161689 1.797719
B 1.517885 0.171912 0.078830
H 4.039960 -2.370826 0.064010
H 3.920134 -1.731715 -1.762844
C 0.958665 2.624650 0.854549
H 0.289535 3.201643 1.504772
H 1.884942 2.471681 1.418262
C -0.677031 1.017283 -1.633402
H 0.237010 1.582565 -1.914910
H -0.350495 -0.029978 -1.677564
C -3.374761 2.005649 0.088830
H -3.690993 1.304818 0.866279
H -3.919915 2.948046 0.216308
H -3.632082 1.576689 -0.880054
C 1.056177 0.748951 3.128438
H 0.771510 0.164176 4.011131
H 2.032593 0.378068 2.788888
H 1.184293 1.789665 3.448179
C 1.278263 3.417620 -0.406730
H 1.864168 4.305066 -0.139873
H 1.872941 2.830593 -1.118554
H 0.378216 3.769272 -0.928241
C -1.790046 1.294023 -2.625565
H -2.152668 2.327263 -2.561380
H -1.407621 1.132797 -3.639088
H -2.630368 0.608732 -2.468499
54
TS-I-Gauche_Et_conf_12 Eopt -1028.019565
C 0.558221 1.155875 0.742866
C -0.548844 1.612827 -0.126847
I -2.789223 -1.248183 0.136182
C 0.111737 0.597863 2.132712
H -0.817844 1.077361 2.451164
H -0.144544 -0.462582 1.993058
C -0.306990 1.748939 -1.580652
H 0.726181 1.492022 -1.827150
H -0.441954 2.828973 -1.773328
C 1.006457 -1.121815 -0.866361
C 3.016010 -0.211152 0.515650
H -0.040614 -1.013713 -1.157006
C 1.097972 -2.444188 -0.056091
C 1.907049 -1.170071 -2.134777
H 3.423193 0.558884 1.181809
C 3.053345 -1.565650 1.281433
C 3.899668 -0.281731 -0.768755
H 0.745628 -3.268861 -0.692869
H 0.404204 -2.395256 0.797483
C 2.509185 -2.727633 0.452678
H 1.300380 -0.917575 -3.015528
H 2.266562 -2.196850 -2.297469
C 3.101805 -0.210091 -2.075964
H 2.459448 -1.490763 2.205042
H 4.089762 -1.767920 1.589947
H 4.620092 0.546445 -0.754644
H 4.497318 -1.205021 -0.755458
H 2.507459 -3.642550 1.060237
H 2.723723 0.819508 -2.188896
B 1.505631 0.012807 0.105569
H 3.768284 -0.384259 -2.931528
H 3.178004 -2.925086 -0.397739
C 1.391829 2.494723 0.974210
H 0.759763 3.212774 1.513390
H 2.171616 2.175607 1.670137
C -1.753373 2.237986 0.460948
H -2.165284 1.575537 1.228480
H -1.303394 3.068141 1.045576
C -1.256061 1.009597 -2.546338
H -1.016675 1.351545 -3.559284
H -1.105174 -0.070803 -2.492928
H -2.307611 1.216244 -2.345834
C 1.109510 0.739595 3.281454
H 1.171983 1.776188 3.633584
H 0.768906 0.126708 4.124065
H 2.121020 0.412187 3.017439
C 2.073637 3.160623 -0.210802
H 2.768862 3.918340 0.170132
H 2.662826 2.439605 -0.793526
H 1.379284 3.670906 -0.886770
C -2.867279 2.776234 -0.421514
H -2.497089 3.375381 -1.262047
H -3.486045 1.959043 -0.807956
H -3.510915 3.417918 0.189819
54
TS-I-Gauche_Et_conf_13 Eopt -1028.022636
C -0.301465 1.247399 -0.198754
C 0.932893 1.057914 0.614925
I 2.359011 -1.443582 -0.587923
C -0.045294 1.386609 -1.725514
H 0.679585 0.612699 -2.005901
H -0.981447 1.114551 -2.235960
C 2.209375 1.771775 0.337063
H 2.394065 1.851440 -0.735252
H 3.024732 1.192686 0.773693
C -1.388805 -1.277697 -0.814944
C -2.556780 0.044103 1.104331
H -0.558759 -1.340141 -1.525487
C -2.737840 -1.310804 -1.600935
C -1.274216 -2.485052 0.152491
H -2.575151 0.928986 1.753145
C -3.893929 -0.022417 0.289260
C -2.437955 -1.207282 2.012612
H -3.238593 -2.276543 -1.435803
H -2.528211 -1.253363 -2.676742

C -3.688623 -0.169024 -1.223395
H -0.291439 -2.460683 0.646474
H -1.299264 -3.414411 -0.436028
C -2.371306 -2.510163 1.215805
H -4.487411 0.879913 0.484265
H -4.499300 -0.864628 0.653228
H -1.545969 -1.126392 2.651111
H -3.301946 -1.229446 2.693630
H -3.268963 0.773194 -1.614473
H -2.201363 -3.351905 1.900826
B -1.366573 0.039189 0.055519
H -3.344095 -2.699211 0.739120
H -4.658780 -0.303555 -1.720846
C -0.896236 2.607592 0.362699
H -1.018993 2.543540 1.449400
H -0.179373 3.408110 0.168533
C 0.764743 0.433397 1.959988
H -0.043927 1.001925 2.453289
H 0.331277 -0.563645 1.797183
C 2.206424 3.175950 0.998582
H 3.243094 3.447465 1.221582
H 1.789725 3.931822 0.325929
H 1.638885 3.197942 1.937643
C 0.407198 2.741172 -2.274658
H -0.366254 3.511152 -2.172813
H 1.315085 3.123310 -1.796139
H 0.623729 2.634216 -3.344740
C -2.232458 2.994660 -0.264742
H -3.053500 2.369544 0.094271
H -2.462713 4.031203 0.012329
H -2.214522 2.940539 -1.358663
C 1.978365 0.327417 2.866928
H 2.408457 1.309188 3.096253
H 1.680337 -0.139202 3.811938
H 2.751767 -0.299688 2.406888
54
TS-I-Gauche_Et_conf_14 Eopt -1028.020535
C -0.456737 1.275879 -0.387108
C 0.753721 1.396698 0.454688
I 2.679635 -1.278661 -0.484324
C -0.088041 1.312454 -1.895999
H 0.203264 2.341804 -2.151130
H 0.804066 0.688299 -2.034460
C 1.956101 2.151338 0.034863
H 2.103197 2.125735 -1.045426
H 2.823953 1.690068 0.512645
C -2.569006 0.061383 1.114600
C -1.210756 -1.455658 -0.511475
H -2.726115 1.050653 1.561188
C -2.259047 -0.929455 2.270528
C -3.886675 -0.375700 0.391053
H -0.384800 -1.547494 -1.225601
C -0.894823 -2.385910 0.693392
C -2.547011 -1.897321 -1.190767
H -3.119536 -0.940609 2.955844
H -1.403654 -0.572048 2.860445
C -1.974164 -2.347105 1.775051
H -4.583217 0.472612 0.370261
H -4.380295 -1.159651 0.983227
C -3.680216 -0.875206 -1.043611
H 0.079201 -2.113561 1.125742
H -0.778473 -3.412719 0.316438
H -2.368831 -2.075539 -2.258939
H -2.867358 -2.863051 -0.774167
H -1.671373 -2.978680 2.621136
H -3.447849 -0.009266 -1.683389
B -1.383432 0.005167 0.060788
H -4.619006 -1.297346 -1.427171
H -2.902063 -2.788704 1.383806
C -1.193065 2.633312 0.024990
H -1.377199 2.630786 1.103735
H -0.538239 3.481989 -0.191206
C 0.687397 0.910833 1.854008
H -0.240268 1.317127 2.290876
H 0.482708 -0.168575 1.759863
C 1.860833 3.626731 0.522506
H 1.308315 3.731282 1.464142
H 2.878072 3.998095 0.677887
H 1.374688 4.249840 -0.233718
C -1.135693 0.835365 -2.899787
H -0.744641 0.997149 -3.912204
H -1.334458 -0.236153 -2.801306
H -2.086623 1.370422 -2.826729
C -2.508068 2.858394 -0.713720
H -3.164454 1.981016 -0.684194
H -3.036803 3.690695 -0.232866
H -2.339330 3.135347 -1.759259
C 1.881106 1.117810 2.770079
H 2.134360 2.176543 2.892462
H 1.644373 0.710707 3.758710
H 2.759770 0.583357 2.388874
54
TS-I-Gauche_Et_conf_15 Eopt -1028.024440
C -0.664721 1.255943 -0.437384
C 0.403740 1.626191 0.485607
I 2.690913 -1.276029 -0.294066
C -0.276732 1.224237 -1.930643
H 0.557806 0.520754 -2.044628
H -1.119505 0.760340 -2.455409
C 1.662675 2.211889 -0.012688
H 1.311265 3.212152 -0.348994
H 1.954822 1.703668 -0.936658
C -1.197983 -1.488052 -0.863866
C -2.348855 -0.410124 1.210166
H -0.471714 -1.398664 -1.678000
C -2.609823 -1.667048 -1.499668
C -0.818596 -2.707640 0.026432
H -2.489508 0.455973 1.866090
C -3.736230 -0.686005 0.559533
C -1.894208 -1.625249 2.073216
H -2.584908 -2.544726 -2.161476
H -2.843179 -0.802967 -2.142891
C -3.710572 -1.831903 -0.451444
H 0.132220 -3.119653 -0.330779
H -1.568748 -3.504749 -0.082804
C -0.669527 -2.344242 1.505045
H -4.105067 0.227083 0.072349
H -4.449597 -0.913383 1.365004
H -1.664258 -1.278969 3.089670
H -2.727386 -2.337190 2.172712
H -4.687532 -1.904996 -0.948202
H 0.207269 -1.681963 1.604981
B -1.338294 -0.190045 0.010282
H -0.448621 -3.242668 2.097317
H -3.567742 -2.782714 0.081114
C -1.794869 2.346522 -0.159152
H -2.233656 2.166721 0.825247
H -1.337870 3.344313 -0.124204
C 0.216650 1.594410 1.954003
H -0.610028 0.949090 2.249469
H 1.147100 1.187701 2.374468
C 2.843239 2.357634 0.934475
H 3.169696 1.373411 1.289969
H 3.677480 2.810269 0.388063
H 2.622252 2.993466 1.798032
C 0.060221 2.558327 -2.620991
H -0.352178 3.429532 -2.096616
H 1.140400 2.712415 -2.711223
H -0.351176 2.568455 -3.637057
C -2.876684 2.334808 -1.234804
H -2.517394 2.759723 -2.177773
H -3.240184 1.322980 -1.445072
H -3.726680 2.939009 -0.895501
C -0.010767 3.014065 2.531336
H -1.039327 3.340374 2.351281
H 0.158956 2.976532 3.611891
H 0.668312 3.755853 2.098693
54
TS-I-Gauche_Et_conf_16 Eopt -1028.023462
C 0.120162 1.204992 0.522734

C -1.096643 1.185602 -0.330938
I -1.980108 -1.818895 0.121229
C -0.186160 0.947991 2.031934
H -0.947266 0.161225 2.082993
H 0.709553 0.516482 2.495555
C -2.446736 1.538070 0.199854
H -2.532490 1.293232 1.259236
H -3.206905 0.966479 -0.337416
C 2.679962 0.242624 0.926959
C 1.391157 -0.932078 -1.005954
H 2.680213 0.999469 1.720616
C 3.794942 0.631172 -0.078171
C 2.952712 -1.149165 1.572957
H 0.472110 -1.052331 -1.587802
C 2.522707 -0.493458 -1.976330
C 1.724960 -2.302738 -0.339608
H 4.760292 0.641060 0.449674
H 3.627051 1.659242 -0.433697
C 3.871671 -0.314471 -1.277468
H 2.955494 -1.045627 2.666066
H 3.958862 -1.497687 1.293651
C 1.918460 -2.205190 1.174913
H 2.244883 0.449947 -2.474195
H 2.612534 -1.245620 -2.773637
H 0.905647 -3.001165 -0.546815
H 2.628869 -2.732332 -0.796852
H 4.614078 0.058871 -1.996058
H 0.947258 -1.938990 1.627810
B 1.315964 0.178114 0.116491
H 2.196236 -3.184877 1.587170
H 4.239693 -1.294911 -0.942508
C 0.772328 2.650791 0.391239
H 0.062533 3.390978 0.773023
H 1.590334 2.620511 1.119206
C -0.944921 1.238648 -1.802848
H -0.901527 2.333998 -1.965089
H 0.032691 0.865993 -2.113129
C -2.725670 3.040139 -0.035053
H -2.780448 3.266688 -1.106596
H -3.694177 3.279183 0.417626
H -1.967239 3.686215 0.419704
C -0.612395 2.141266 2.889870
H -1.450438 2.703478 2.460307
H -0.928691 1.776812 3.874740
H 0.214770 2.843250 3.050239
C 1.322116 3.123462 -0.952933
H 1.782933 2.317761 -1.536018
H 0.555741 3.595954 -1.576114
H 2.092411 3.882794 -0.772506
C -2.070723 0.700581 -2.679988
H -2.196260 -0.376519 -2.527592
H -3.024209 1.200512 -2.476266
H -1.816238 0.881331 -3.729939
54
TS-I-Gauche_Et_conf_17 Eopt -1028.020807
C -0.259204 1.258909 -0.422902
C 0.902580 1.212547 0.490621
I 2.398568 -1.639958 -0.533324
C 0.087269 1.166391 -1.924348
H 0.859232 1.894252 -2.197696
H 0.514354 0.173404 -2.104591
C 2.174839 1.912642 0.209810
H 2.238868 2.217539 -0.834486
H 2.977703 1.188823 0.395187
C -2.516074 0.428019 1.063959
C -1.550137 -1.273064 -0.636431
H -2.416619 1.387890 1.587696
C -2.594158 -0.684021 2.137126
C -3.785612 0.520144 0.169865
H -0.797790 -1.499677 -1.403035
C -1.356729 -2.279638 0.528514
C -2.955100 -1.436176 -1.284175
H -3.553707 -0.607658 2.671705
H -1.816841 -0.467892 2.885260
C -2.395248 -2.130825 1.645443
H -3.638971 1.305571 -0.591495
H -4.634954 0.852446 0.784575
C -4.136319 -0.795222 -0.529622
H -0.341523 -2.175504 0.938107
H -1.403307 -3.300428 0.121056
H -2.919092 -1.008396 -2.294246
H -3.151641 -2.509984 -1.425744
H -2.090289 -2.751036 2.499434
H -4.951790 -0.611382 -1.242534
B -1.385991 0.160967 0.000884
H -4.545051 -1.489809 0.212025
H -3.346879 -2.552135 1.303502
C -0.851940 2.718792 -0.112312
H -1.898004 2.659748 -0.437612
H -0.885749 2.883014 0.970865
C 0.691217 0.627606 1.840315
H -0.135923 1.213110 2.283921
H 0.249266 -0.365271 1.676478
C 2.382610 3.123562 1.169304
H 1.455188 3.440579 1.662537
H 3.120395 2.887558 1.938468
H 2.759069 3.969578 0.586272
C -1.130766 1.375496 -2.819052
H -1.506248 2.405116 -2.781718
H -0.873179 1.153432 -3.861235
H -1.957656 0.713382 -2.538140
C -0.199045 3.910689 -0.800612
H -0.723626 4.819292 -0.478253
H 0.855641 4.039788 -0.547433
H -0.280496 3.854312 -1.890842
C 1.859469 0.511053 2.806103
H 2.148280 1.477023 3.228983
H 1.562181 -0.138617 3.636327
H 2.729097 0.053482 2.318778
54
TS-I-Gauche_Et_conf_2 Eopt -1028.024121
C -0.175024 1.218924 -0.479511
C 1.018649 1.139667 0.397123
I 2.275933 -1.651694 -0.349330
C 0.140189 1.104946 -1.985819
H 0.960348 1.773955 -2.269329
H 0.493907 0.083124 -2.168913
C 2.299071 1.769070 -0.020104
H 2.030045 2.825488 -0.197126
H 2.564703 1.398842 -1.015405
C -2.478269 0.458599 0.991176
C -1.485534 -1.307388 -0.620654
H -2.385273 1.427015 1.497844
C -2.632650 -0.618330 2.089347
C -3.711130 0.556359 0.044473
H -0.701961 -1.578484 -1.338504
C -1.368876 -2.269419 0.591284
C -2.854203 -1.441460 -1.343444
H -3.605284 -0.491300 2.589744
H -1.872423 -0.411308 2.858547
C -2.468195 -2.079483 1.637916
H -3.526511 1.335140 -0.713943
H -4.578711 0.902634 0.625168
C -4.056223 -0.758455 -0.661728
H -0.386883 -2.128628 1.066118
H -1.380408 -3.306708 0.224235
H -2.742067 -1.023173 -2.352628
H -3.074405 -2.510572 -1.487069
H -2.232396 -2.694380 2.517421
H -4.826102 -0.559871 -1.420128
B -1.314850 0.140911 -0.025843
H -4.524780 -1.434515 0.061371
H -3.413233 -2.473472 1.248701
C -0.799601 2.665194 -0.192705
H -1.785686 2.608050 -0.667463
H -0.992865 2.785837 0.873354
C 0.870777 0.750916 1.829227
H 0.092642 -0.009074 1.952620
H 1.814546 0.309711 2.159697
C 3.480916 1.689785 0.931763

H	3.261681	2.152396	1.901657
H	3.779274	0.647278	1.095541
H	4.330970	2.221117	0.490406
C	-1.065826	1.412705	-2.869025
H	-1.278725	2.488688	-2.907506
H	-0.883779	1.072655	-3.894880
H	-1.974149	0.910453	-2.509817
C	-0.080888	3.901901	-0.719019
H	0.711999	4.250613	-0.047293
H	0.346957	3.758657	-1.718481
H	-0.810519	4.718504	-0.789678
C	0.552087	1.971553	2.727866
H	-0.524945	2.164234	2.757757
H	0.890331	1.750026	3.744816
H	1.061870	2.881775	2.387845

54

TS-I-Gauche_Et_conf_3 Eopt -1028.015805

C	-0.706310	1.413414	-0.240492
C	0.453539	1.672875	0.637134
I	2.813929	-1.140218	-0.487122
C	-0.274843	1.608154	-1.728896
H	-0.162303	2.683164	-1.919961
H	0.718599	1.156810	-1.854368
C	0.558977	0.937296	1.908719
H	0.486822	-0.133747	1.664841
H	-0.413028	1.159749	2.396718
C	-2.671562	-0.168950	1.092896
C	-1.088079	-1.390126	-0.573989
H	-2.963736	0.755883	1.604578
C	-2.276878	-1.201436	2.184769
C	-3.904341	-0.702425	0.288672
H	-0.231043	-1.334123	-1.254116
C	-0.717848	-2.381152	0.564457
C	-2.339205	-1.924642	-1.346178
H	-3.141889	-1.352729	2.847397
H	-1.472258	-0.798324	2.816569
C	-1.829350	-2.540466	1.600445
H	-4.698244	0.055682	0.301655
H	-4.317189	-1.582372	0.802534
C	-3.593847	-1.061607	-1.169153
H	0.208387	-2.055277	1.058363
H	-0.483945	-3.355211	0.110074
H	-2.104901	-1.991099	-2.416144
H	-2.553618	-2.952771	-1.021099
H	-1.487499	-3.199453	2.409954
H	-3.452141	-0.127183	-1.734419
B	-1.456857	-0.001575	0.084815
H	-4.460659	-1.563808	-1.619835
H	-2.691844	-3.045323	1.141849
C	-1.628722	2.630964	0.228105
H	-1.860683	2.505911	1.291538
H	-1.077471	3.572679	0.124989
C	1.348617	2.821305	0.374996
H	0.946429	3.457399	-0.414420
H	1.337402	3.393342	1.318955
C	1.710837	1.192745	2.863460
H	1.494781	0.698378	3.816364
H	2.642476	0.770237	2.472486
H	1.856333	2.260294	3.064460
C	-1.188785	1.028319	-2.806147
H	-1.207970	-0.064645	-2.784004
H	-2.216838	1.396575	-2.739308
H	-0.796551	1.324475	-3.787078
C	-2.922809	2.746303	-0.567707
H	-3.473739	1.799617	-0.611180
H	-3.566822	3.489014	-0.080814
H	-2.733741	3.090023	-1.590050
C	2.818966	2.503081	0.042302
H	2.886872	2.019709	-0.936437
H	3.358975	3.455696	0.012666
H	3.286945	1.848942	0.780281

54

TS-I-Gauche_Et_conf_4 Eopt -1028.016782

C	-0.266298	1.308828	-0.190173
C	0.992511	1.220819	0.597338
I	2.399379	-1.308206	-0.583256
C	0.036936	1.566745	-1.686969
H	0.434050	2.588250	-1.782025
H	0.849285	0.886831	-1.974494
C	0.891812	0.912845	2.052763
H	-0.113621	0.567682	2.303715
H	0.989883	1.919315	2.504985
C	-2.451525	0.040665	1.171622
C	-1.310983	-1.292065	-0.746844
H	-2.498714	0.945607	1.788018
C	-2.205114	-1.158635	2.128347
C	-3.828998	-0.139241	0.452348
H	-0.520805	-1.346559	-1.503396
C	-1.055615	-2.431377	0.282333
C	-2.702629	-1.484669	-1.426721
H	-3.021826	-1.189731	2.864647
H	-1.277527	-1.010637	2.702136
C	-2.107494	-2.488392	1.386341
H	-4.435144	0.762993	0.607248
H	-4.382253	-0.960935	0.929382
C	-3.721655	-0.402664	-1.053793
H	-0.067161	-2.295785	0.743379
H	-1.015610	-3.387334	-0.260797
H	-2.576552	-1.488383	-2.517138
H	-3.104503	-2.474860	-1.168332
H	-1.853411	-3.288174	2.095136
H	-3.420546	0.533578	-1.548934
B	-1.298191	0.055462	0.076464
H	-4.710625	-0.660420	-1.456682
H	-3.087703	-2.752828	0.964697
C	-0.910408	2.607834	0.489566
H	-1.097465	2.395714	1.544987
H	-0.196857	3.434247	0.452909
C	2.258552	1.901723	0.160614
H	3.090152	1.459096	0.714176
H	2.441889	1.733152	-0.901300
C	1.935947	0.011286	2.709236
H	1.830828	0.091890	3.797047
H	1.777244	-1.030375	2.413794
H	2.958626	0.294262	2.440261
C	-1.084159	1.378358	-2.708476
H	-0.703010	1.658574	-3.698602
H	-1.402299	0.333380	-2.772785
H	-1.966170	1.994264	-2.513066
C	-2.203912	3.075031	-0.166954
H	-2.923510	2.259999	-0.304932
H	-2.669941	3.828522	0.480189
H	-2.017833	3.545567	-1.137814
C	2.259648	3.423392	0.426773
H	1.640351	3.963910	-0.296760
H	1.923155	3.671473	1.441183
H	3.291802	3.772419	0.315293

54

TS-I-Gauche_Et_conf_5 Eopt -1028.016184

C	0.559316	1.109638	0.708536
C	-0.410247	1.718068	-0.226637
I	-2.849429	-1.243896	0.086433
C	-0.222218	0.742872	2.032816
H	-1.018334	1.469657	2.229500
H	-0.723559	-0.209865	1.846195
C	-0.640165	1.224667	-1.598215
H	-1.725171	1.098783	-1.717031
H	-0.176827	0.252271	-1.762948
C	1.534840	-1.536097	0.877816
C	2.243424	-0.130731	-1.195957
H	0.910482	-1.629776	1.772527
C	3.023970	-1.542742	1.337206
C	1.241749	-2.740498	-0.063448
H	2.130493	0.785280	-1.785590
C	3.710556	-0.174548	-0.677054
C	1.931093	-1.352183	-2.109173
H	3.216020	-2.471523	1.893941
H	3.207916	-0.714593	2.040107
C	3.999381	-1.414067	0.167778
H	0.385156	-3.298909	0.334580

H	2.094782	-3.435852	-0.058502
C	0.919311	-2.329644	-1.502026
H	3.920851	0.720809	-0.069727
H	4.391924	-0.132636	-1.539454
H	1.535882	-0.990133	-3.068341
H	2.862790	-1.885611	-2.348989
H	5.028624	-1.369105	0.548126
H	-0.076335	-1.856915	-1.497853
B	1.362815	-0.162145	0.118854
H	0.831867	-3.221160	-2.137955
H	3.944246	-2.312990	-0.462278
C	1.601047	2.246443	1.088929
H	1.106209	2.931290	1.788255
H	2.384799	1.734193	1.662791
C	-1.137782	2.944955	0.161099
H	-0.988765	3.184357	1.215581
H	-0.599962	3.728587	-0.409146
C	-0.158909	2.241770	-2.659448
H	0.905608	2.471600	-2.543198
H	-0.311762	1.789329	-3.644228
H	-0.734197	3.172378	-2.610134
C	0.653758	0.634435	3.276795
H	0.993339	1.613140	3.634712
H	0.062365	0.176462	4.079035
H	1.535930	0.005756	3.114003
C	2.241535	3.057489	-0.031159
H	2.771451	2.436316	-0.758902
H	1.510335	3.669547	-0.572277
H	2.972213	3.745681	0.411232
C	-2.619818	3.001512	-0.225037
H	-3.160947	2.155817	0.215803
H	-3.046847	3.933269	0.159232
H	-2.759933	2.983719	-1.311235

54

TS-I-Gauche_Et_conf_6 Eopt -1028.021833

C	-0.410252	1.440121	-0.127169
C	0.757286	1.440114	0.764833
I	2.412159	-1.385417	-0.618764
C	-0.109328	1.658795	-1.629505
H	0.602380	0.883863	-1.941557
H	-1.039501	1.428891	-2.159887
C	2.018860	2.048714	0.303244
H	1.674397	3.103698	0.212140
H	2.222719	1.740573	-0.725710
C	-1.497850	-0.995819	-1.064681
C	-2.340507	-0.202029	1.250509
H	-0.765390	-0.895979	-1.874547
C	-2.904745	-0.688925	-1.653658
C	-1.384610	-2.438946	-0.513821
H	-2.321488	0.532982	2.063984
C	-3.804125	-0.216358	0.722424
C	-1.914546	-1.569123	1.839735
H	-3.069812	-1.339719	-2.524614
H	-2.928396	0.342394	-2.039987
C	-4.042983	-0.883618	-0.646422
H	-0.313486	-2.657104	-0.417642
H	-1.775234	-3.139884	-1.268185
C	-2.050301	-2.721513	0.843843
H	-4.145335	0.826086	0.668589
H	-4.441346	-0.699517	1.479088
H	-0.871033	-1.508279	2.182846
H	-2.520876	-1.778276	2.733743
H	-4.971419	-0.481275	-1.074625
H	-1.592315	-3.621827	1.275943
B	-1.343640	0.090258	0.059860
H	-3.112295	-2.960929	0.720256
H	-4.218999	-1.957115	-0.520334
C	-1.279466	2.648648	0.447752
H	-1.625290	2.408528	1.456108
H	-0.641317	3.538190	0.534559
C	0.597832	1.134905	2.208444
H	0.929017	2.049213	2.731383
H	-0.449770	0.970121	2.457243
C	3.276064	2.011589	1.153824
H	3.723459	1.011678	1.139860
H	4.002611	2.710545	0.725427
H	3.093888	2.311608	2.192585
C	0.375314	3.039000	-2.107755
H	1.465741	3.095442	-2.183721
H	-0.025081	3.239333	-3.108629
H	0.047637	3.860489	-1.459092
C	-2.472359	2.974324	-0.443868
H	-2.164244	3.394070	-1.407466
H	-3.089037	2.091247	-0.645846
H	-3.103607	3.716380	0.059957
C	1.426089	-0.048716	2.743791
H	2.500348	0.096251	2.620723
H	1.202893	-0.148324	3.811930
H	1.151722	-0.974525	2.231516

54

TS-I-Gauche_Et_conf_7 Eopt -1028.026960

C	0.189951	1.230630	0.525417
C	-1.017015	1.222058	-0.334825
I	-2.056262	-1.739601	0.321624
C	-0.163565	1.164758	2.028589
H	-0.959355	1.879219	2.261539
H	-0.577341	0.166130	2.222148
C	-2.316098	1.709128	0.201150
H	-2.098146	2.763532	0.464771
H	-2.504848	1.232215	1.166519
C	1.734494	-1.152456	0.872437
C	2.167110	0.258041	-1.272980
H	1.141263	-1.309744	1.782119
C	3.216534	-0.920580	1.294753
C	1.650364	-2.429969	-0.023950
H	1.898449	1.113219	-1.903890
C	3.608472	0.518917	-0.745421
C	2.124997	-1.034633	-2.137041
H	3.563589	-1.812160	1.837014
H	3.294817	-0.082874	2.000398
C	4.135072	-0.631831	0.109554
H	0.881648	-3.095946	0.386079
H	2.603294	-2.976959	0.036165
C	1.309219	-2.158359	-1.493385
H	3.629851	1.447187	-0.151923
H	4.270305	0.685468	-1.607937
H	1.683977	-0.800750	-3.115625
H	3.148448	-1.378528	-2.344314
H	5.142218	-0.390018	0.475251
H	0.245701	-1.878973	-1.545933
B	1.282705	0.108520	0.036936
H	1.414634	-3.084211	-2.075510
H	4.242321	-1.533710	-0.509312
C	0.942085	2.608837	0.230441
H	1.860906	2.540984	0.825122
H	1.255927	2.647261	-0.813080
C	-0.945504	1.019793	-1.808090
H	-0.112487	0.377727	-2.095573
H	-1.872120	0.523347	-2.113074
C	-3.549840	1.625938	-0.681925
H	-3.444231	2.192228	-1.613922
H	-3.776402	0.580505	-0.925484
H	-4.404012	2.038149	-0.134077
C	0.981978	1.450504	2.991171
H	1.285770	2.505161	2.963986
H	0.659694	1.229546	4.015857
H	1.864112	0.837926	2.787515
C	0.207399	3.891136	0.594119
H	0.921421	4.723624	0.573240
H	-0.587541	4.128493	-0.123372
H	-0.229124	3.855368	1.600126
C	-0.844251	2.369042	-2.560556
H	0.178685	2.757253	-2.543521
H	-1.128640	2.198501	-3.603813
H	-1.512857	3.131360	-2.144985

54

TS-I-Gauche_Et_conf_8 Eopt -1028.016782

C	-0.424678	1.291115	-0.448475
C	0.730842	1.384722	0.475194
I	2.758121	-1.297681	-0.439791

C 0.019018 1.226773 -1.928433
H 0.695529 2.061521 -2.144924
H 0.597828 0.305182 -2.062082
C 1.980760 2.104847 0.142318
H 2.226788 2.038615 -0.917572
H 2.792903 1.646046 0.711595
C -2.525932 0.058217 1.081847
C -1.349688 -1.363142 -0.732840
H -2.596723 1.006849 1.630915
C -2.281027 -1.055251 2.134391
C -3.865395 -0.154040 0.319758
H -0.604374 -1.422792 -1.537247
C -0.902298 -2.351405 0.379519
C -2.726540 -1.749180 -1.333788
H -3.196116 -1.178141 2.733974
H -1.514380 -0.696999 2.835234
C -1.839894 -2.426533 1.589431
H -4.014217 0.664250 -0.398088
H -4.695252 -0.084817 1.038769
C -3.949628 -1.479920 -0.441374
H 0.105362 -2.068672 0.714341
H -0.793052 -3.352220 -0.064203
H -2.860944 -1.191828 -2.271296
H -2.703015 -2.812511 -1.617518
H -1.327766 -2.972366 2.393629
H -4.847518 -1.462618 -1.074503
B -1.412806 0.051990 -0.040476
H -4.106090 -2.301491 0.264095
H -2.713299 -3.035817 1.337199
C -1.073759 2.734377 -0.171211
H -0.899426 3.062929 0.861465
H -0.520729 3.425694 -0.814379
C 0.567080 0.887075 1.862496
H -0.403557 1.259471 2.226473
H 0.404366 -0.198183 1.738769
C 1.882076 3.597274 0.566510
H 2.903471 3.980760 0.650293
H 1.350409 4.186608 -0.185112
H 1.383523 3.730436 1.533214
C -1.118572 1.277243 -2.943050
H -1.934980 0.589301 -2.689604
H -1.539899 2.285894 -3.030934
H -0.740781 0.990996 -3.931689
C -2.568691 2.874843 -0.462050
H -2.883776 2.321146 -1.350185
H -3.184529 2.555387 0.382077
H -2.780671 3.936924 -0.638934
C 1.681236 1.108712 2.871404
H 1.366172 0.703360 3.838753
H 2.593257 0.580370 2.568647
H 1.915626 2.169520 3.010231
54
TS-I-Gauche_Et_conf_9 Eopt -1028.027706
C -0.688066 1.279231 -0.423136
C 0.618030 1.569244 0.173945
I 2.632750 -1.333533 -0.269391
C -0.730444 1.470936 -1.953150
H -0.249325 2.415649 -2.231252
H -0.127865 0.675043 -2.411854
C 1.688254 2.160742 -0.656064
H 1.229267 3.123831 -0.968004
H 1.748848 1.597680 -1.596455
C -1.659635 -1.344918 -1.018452
C -1.798189 -0.580104 1.466534
H -1.317776 -1.141539 -2.039162
C -3.210730 -1.552266 -1.026097
C -0.973782 -2.657539 -0.551624
H -1.598619 0.168048 2.239374
C -3.345453 -0.802956 1.421251
C -1.045590 -1.876992 1.866580
H -3.426932 -2.616643 -0.857256
H -3.607443 -1.317373 -2.022387
C -3.957012 -0.718608 0.017924
H 0.110790 -2.561788 -0.663692
H -1.295426 -3.465935 -1.225187
C -1.300525 -3.028253 0.894150
H -3.829468 -0.050977 2.057651
H -3.584220 -1.780354 1.865290
H 0.036166 -1.672970 1.907719
H -1.354878 -2.167209 2.881639
H -3.953972 0.334227 -0.307344
H -0.698709 -3.897715 1.191399
B -1.301387 -0.201530 0.017153
H -2.351924 -3.343594 0.969934
H -5.012392 -1.020838 0.054163
C -1.715908 2.260392 0.292440
H -2.717079 1.959654 -0.035307
H -1.683025 2.100514 1.372670
C 0.869582 1.475723 1.632813
H 0.184813 0.779145 2.115682
H 1.890164 1.091929 1.748096
C 3.065164 2.402512 -0.061750
H 3.528693 1.458066 0.243583
H 3.701675 2.857995 -0.828049
H 3.041269 3.078962 0.798784
C -2.129870 1.478920 -2.564024
H -2.052000 1.448785 -3.657217
H -2.736277 0.622429 -2.254432
H -2.681892 2.389741 -2.299933
C -1.501179 3.732105 -0.038484
H -2.268339 4.329245 0.469888
H -0.522050 4.095524 0.296122
H -1.586628 3.928545 -1.113483
C 0.787945 2.851252 2.341702
H -0.250909 3.158147 2.496509
H 1.267810 2.750257 3.320154
H 1.303283 3.641909 1.786941
42
TS-I-Gauche_Me_conf_1 Eopt -870.966166
C -0.866156 1.771462 -0.468209
C 0.094792 2.214096 0.520113
I 2.834625 -0.610540 -0.097473
C -0.371771 1.852245 -1.912013
H -0.159579 2.893074 -2.192342
H 0.532364 1.254113 -2.069361
H -1.147868 1.476005 -2.587528
C 1.410054 2.723930 0.130157
H 1.170318 3.788661 -0.092784
H 2.129736 2.716530 0.953118
H 1.820370 2.286252 -0.779878
C -2.306639 -0.145838 1.113569
C -1.068060 -0.978663 -1.031578
H -2.481827 0.647340 1.846488
C -1.864012 -1.441093 1.849953
C -3.664171 -0.368282 0.370732
H -0.324952 -0.773709 -1.808890
C -0.671477 -2.268596 -0.259155
C -2.457915 -1.133357 -1.727120
H -2.683821 -2.175056 1.837537
H -1.679394 -1.207010 2.906579
C -0.600869 -2.066487 1.256214
H -4.012916 0.578337 -0.066739
H -4.414367 -0.660066 1.119480
C -3.579268 -1.425591 -0.729514
H 0.313034 -2.598241 -0.612162
H -1.377624 -3.079631 -0.491646
H -2.701370 -0.214541 -2.285805
H -2.387009 -1.940021 -2.470501
H 0.250729 -1.398869 1.466953
H -4.539965 -1.480043 -1.258872
B -1.315780 0.218037 -0.054714
H -3.421119 -2.414909 -0.278006
H -0.379724 -3.020303 1.754264
C -2.187286 2.600545 -0.325622
H -2.889400 2.261072 -1.093972
H -2.662170 2.506269 0.653705
H -1.962040 3.656819 -0.517414
C -0.200500 2.250646 1.960545
H 0.319369 3.090057 2.436366
H -1.256133 2.229070 2.225700

H 0.288782 1.337126 2.356901
42
TS-I-Gauche_Me_conf_2 Eopt -870.967822
C -0.637514 1.866197 -0.108984
C 0.782921 2.034003 0.130857
I 2.332329 -0.797298 -0.043678
C -1.104073 2.388922 -1.474296
H -0.935321 3.472587 -1.538920
H -0.593530 1.909702 -2.314301
H -2.179234 2.214276 -1.589606
C 1.682162 2.444302 -0.961710
H 1.414328 3.506371 -1.137303
H 2.735891 2.378983 -0.687748
H 1.483285 1.916753 -1.898695
C -1.337751 -0.628685 -1.298610
C -1.396914 -0.475003 1.317644
H -0.914574 -0.249022 -2.235619
C -2.902765 -0.495367 -1.357231
C -0.972315 -2.123509 -1.164333
H -1.029779 0.011209 2.229419
C -2.963774 -0.320227 1.261218
C -1.024841 -1.971019 1.378684
H -3.316532 -1.492038 -1.562192
H -3.182983 0.131939 -2.212705
C -3.569205 0.090314 -0.098487
H 0.115037 -2.241217 -1.229694
H -1.407587 -2.664706 -2.018228
C -1.475125 -2.744583 0.139158
H -3.278151 0.421400 2.006216
H -3.401519 -1.276602 1.578452
H 0.061523 -2.068563 1.498163
H -1.489322 -2.412310 2.273779
H -3.540808 1.184859 -0.181066
H -1.125896 -3.783903 0.208634
B -0.926145 0.196037 -0.020248
H -2.575473 -2.795195 0.121119
H -4.635737 -0.170017 -0.102812
C -1.450480 2.556757 1.018925
H -2.518512 2.488001 0.801999
H -1.269677 2.134496 2.009978
H -1.188621 3.623092 1.042278
C 1.297841 2.120270 1.511604
H 1.067302 3.156665 1.828058
H 0.776272 1.450956 2.202599
H 2.377897 1.974670 1.557734
58
TS-III_Et_conf_1 Eopt -845.384064
C -2.292355 0.945200 1.691214
C -1.547214 -0.329496 1.237813
C -1.715588 0.449044 -1.216998
C -2.237047 1.813000 -0.721252
H -1.572012 1.624119 2.165247
H -2.876410 2.271697 -1.493020
C -2.523134 -1.490493 0.943466
H -3.102467 -1.733250 1.849089
C -2.884256 -0.543846 -1.451486
H -2.530884 -1.335320 -2.122899
H -0.941508 -0.659561 2.095261
H -1.260166 0.616823 -2.204278
C -3.505791 -1.207029 -0.202799
H -3.982205 -2.150695 -0.506662
H -4.322557 -0.585342 0.181185
C -3.021799 1.735079 0.590773
H -3.216314 2.754479 0.954953
H -4.009935 1.303479 0.395718
H -1.931104 -2.385375 0.704610
H -3.685331 -0.027592 -2.007351
H -3.017129 0.685115 2.481212
H -1.394220 2.503125 -0.576936
B -0.674836 -0.163768 -0.131038
C 0.906574 0.784237 -0.032612
C 1.589853 -0.475526 0.137092
C 2.548389 -1.031108 -0.878020
H 2.435647 -2.117396 -0.941751
C 1.755094 -1.071187 1.509223
H 0.959670 -0.724762 2.168101
C 0.905295 1.732060 1.176417
H 0.250334 2.580399 0.934323
H 2.679512 -0.616938 1.899245
C 1.092733 1.475827 -1.392971
H 0.906102 0.740026 -2.184464
H 0.290768 2.213665 -1.503232
H 2.357781 -0.619777 -1.871845
H 0.440545 1.239381 2.036044
O -0.053331 -1.525869 -0.464006
C -0.127620 -2.130716 -1.722068
H -1.024473 -2.765330 -1.796758
H -0.145770 -1.401874 -2.546856
H 0.740645 -2.788754 -1.864869
C 3.990514 -0.722318 -0.435582
H 4.303773 -1.390591 0.374999
H 4.672366 -0.869255 -1.280560
H 4.103616 0.309633 -0.081707
C 1.870846 -2.590422 1.579916
H 1.940230 -2.901404 2.628677
H 0.985961 -3.059673 1.135382
H 2.763416 -2.966384 1.065946
C 2.411132 2.192612 -1.703227
H 2.425230 2.484415 -2.762083
H 2.524382 3.111523 -1.118167
H 3.291037 1.565731 -1.523288
C 2.266569 2.275498 1.626622
H 2.969310 1.466605 1.863082
H 2.734647 2.913558 0.871469
H 2.145161 2.878789 2.536290
58
TS-III_Et_conf_10 Eopt -845.381931
C -2.283728 1.257019 1.480354
C -1.510666 -0.073336 1.341501
C -1.754717 0.021836 -1.225420
C -2.286538 1.464226 -1.109560
H -1.588056 2.029509 1.829448
H -2.988448 1.666524 -1.935302
C -2.468394 -1.285305 1.376769
H -3.000920 -1.313140 2.341310
C -2.908793 -1.009964 -1.151014
H -2.548472 -1.962475 -1.559296
H -0.885295 -0.163054 2.241058
H -1.328730 -0.072723 -2.235529
C -3.502514 -1.281586 0.244887
H -4.023405 -2.250277 0.226055
H -4.276936 -0.540883 0.476624
C -2.999313 1.773709 0.214419
H -3.134679 2.861826 0.300771
H -4.014077 1.362204 0.167209
H -1.870924 -2.206912 1.323145
H -3.721378 -0.689970 -1.825345
H -3.032843 1.158077 2.284245
H -1.456682 2.169436 -1.247800
B -0.664463 -0.240291 -0.046312
C 0.852019 0.722761 -0.267169
C 1.591379 -0.413777 0.241399
C 2.551444 -1.185875 -0.620464
H 2.132715 -1.302288 -1.625159
C 1.809267 -0.597272 1.717250
H 1.847066 -1.668213 1.936456
C 0.779277 1.993379 0.594709
H -0.163162 2.500957 0.360340
H 0.984424 -0.177455 2.290286
C 1.070527 1.027588 -1.759844
H 0.741591 0.167165 -2.354107
H 0.378252 1.835677 -2.022585
H 3.407853 -0.506017 -0.746633
H 0.708881 1.724818 1.655030
O 0.009984 -1.635188 -0.014853
C -0.093297 -2.520685 -1.097742
H -0.924377 -3.224061 -0.933498
H -0.258638 -2.004097 -2.053858
H 0.820970 -3.119163 -1.189776
C 3.053258 -2.517122 -0.068240

H 3.752470 -2.371429 0.763592
H 2.231988 -3.145412 0.294606
H 3.584038 -3.066019 -0.854869
C 3.118211 0.070302 2.160838
H 3.976844 -0.262021 1.564876
H 3.052509 1.161044 2.088906
H 3.314883 -0.186804 3.208042
C 2.493303 1.421771 -2.220313
H 2.946372 0.634162 -2.834429
H 2.471329 2.333410 -2.829937
H 3.172749 1.608233 -1.382329
C 1.892589 3.028360 0.405609
H 1.743385 3.858318 1.108589
H 2.894353 2.616860 0.579527
H 1.876913 3.450988 -0.607079
58
TS-III_Et_conf_11 Eopt -845.379059
C 2.397728 1.723317 -0.368528
C 1.696264 0.565573 -1.107535
C 1.434299 -0.622303 1.170860
C 2.064860 0.592100 1.882476
H 1.653486 2.506892 -0.173205
H 2.609581 0.243805 2.775221
C 2.663248 -0.525332 -1.606695
H 3.423849 -0.085147 -2.272825
C 2.577501 -1.611433 0.788861
H 2.168675 -2.622920 0.719647
H 1.227395 0.987946 -2.009796
H 0.834431 -1.154098 1.925549
C 3.387984 -1.313218 -0.498405
H 3.738640 -2.267591 -0.918199
H 4.301591 -0.770585 -0.230963
C 3.025452 1.377184 0.986929
H 3.315665 2.309907 1.492480
H 3.955409 0.812412 0.855618
H 2.081669 -1.217313 -2.232780
H 3.286874 -1.650377 1.631843
H 3.161040 2.183185 -1.019194
H 1.289016 1.272827 2.248653
B 0.607328 -0.200278 -0.181242
C -0.947508 0.800371 -0.014246
C -1.702692 -0.423775 -0.026632
C -1.945484 -1.169562 1.257438
H -1.021263 -1.189881 1.837954
C -2.610837 -0.809961 -1.155354
H -2.646903 -1.901140 -1.239750
C -1.217740 1.669771 -1.265532
H -0.912883 1.111507 -2.158490
H -2.247539 -0.419431 -2.106061
C -1.012091 1.597518 1.293690
H -0.797613 0.947550 2.148788
H -0.218559 2.353761 1.290381
H -2.622726 -0.517093 1.832764
H -2.303643 1.804222 -1.359131
O -0.068140 -1.360049 -0.917368
C 0.224754 -2.719100 -0.812403
H 1.204651 -2.967132 -1.250058
H 0.216308 -3.073706 0.229647
H -0.539076 -3.279123 -1.372593
C -2.554161 -2.566559 1.175674
H -1.959226 -3.239681 0.550441
H -2.595778 -2.995108 2.183809
H -3.576371 -2.551695 0.781258
C -4.021429 -0.269018 -0.865627
H -4.028856 0.824924 -0.791080
H -4.693012 -0.557163 -1.682473
H -4.429653 -0.673354 0.068775
C -2.357695 2.295814 1.506321
H -2.361376 2.854722 2.451100
H -2.573120 3.006662 0.697972
H -3.185830 1.574181 1.540684
C -0.590225 3.062355 -1.347810
H -1.126077 3.649599 -2.104699
H -0.652855 3.618592 -0.404180
H 0.459202 3.029177 -1.652841
58
TS-III_Et_conf_12 Eopt -845.381704
C 2.429996 1.553251 -1.055077
C 1.764433 0.163581 -1.273636
C 1.574056 -0.152558 1.301434
C 2.258939 1.221988 1.525451
H 2.070935 2.265139 -1.811542
H 3.308469 1.063580 1.817842
C 2.898598 -0.894800 -1.301240
H 3.635364 -0.601053 -2.066451
C 2.635984 -1.271144 1.203339
H 2.129245 -2.239792 1.071569
H 1.299498 0.157208 -2.270729
H 0.948339 -0.388062 2.176206
C 3.610387 -1.046185 0.045019
H 4.331799 -1.874372 -0.008203
H 4.204552 -0.141363 0.245291
C 2.213520 2.185162 0.326691
H 1.239104 2.683754 0.320346
H 2.951552 2.986668 0.477415
H 2.519360 -1.872212 -1.614142
H 3.201405 -1.335855 2.147173
H 3.511168 1.461556 -1.239910
H 1.798196 1.735839 2.380938
B 0.725329 -0.230343 -0.078175
C -0.849496 0.773483 -0.021632
C -1.564314 -0.472964 0.034592
C -1.934617 -1.058149 1.370808
H -1.199126 -0.744393 2.118119
C -2.371198 -0.990210 -1.122951
H -2.271169 -2.075287 -1.204649
C -0.906765 1.592606 -1.318753
H -0.036948 2.260663 -1.312269
H -2.030510 -0.554894 -2.065059
C -0.973203 1.676965 1.224807
H -0.386911 1.284247 2.061749
H -0.507173 2.631506 0.962849
H -2.876533 -0.558400 1.641604
H -0.756933 0.941624 -2.186961
O 0.093363 -1.620352 -0.193868
C 0.223139 -2.519560 -1.254374
H 0.292725 -2.022205 -2.234510
H 1.106264 -3.161647 -1.121135
H -0.648589 -3.188345 -1.268858
C -2.137081 -2.567795 1.428739
H -1.206003 -3.091178 1.187089
H -2.440297 -2.854539 2.442663
H -2.921515 -2.911353 0.743684
C -3.860253 -0.673534 -0.898361
H -4.411492 -0.839261 -1.830611
H -4.289826 -1.325268 -0.128485
H -4.024304 0.364227 -0.587721
C -2.400914 1.982963 1.723720
H -2.681325 1.354265 2.576360
H -2.476451 3.025434 2.058137
H -3.155115 1.833638 0.941137
C -2.147075 2.458696 -1.545006
H -2.039343 3.027465 -2.478097
H -3.062304 1.861121 -1.626652
H -2.284476 3.185304 -0.733790
58
TS-III_Et_conf_13 Eopt -845.377660
C 2.530571 1.525049 -0.942716
C 1.746470 0.229278 -1.278793
C 1.439721 -0.276323 1.251473
C 2.254945 1.005687 1.588009
H 2.254034 2.321768 -1.648476
H 3.279744 0.720074 1.871618
C 2.749273 -0.951772 -1.355535
H 3.524313 -0.712303 -2.101824
C 2.397902 -1.479568 1.103353
H 1.812754 -2.386430 0.885080
H 1.323350 0.348663 -2.285974
H 0.787627 -0.492942 2.107355
C 3.418237 -1.265849 -0.016511

H	4.055469	-2.155855	-0.121364
H	4.094117	-0.441293	0.257293
C	2.322802	2.064659	0.477393
H	1.389794	2.635481	0.484766
H	3.115273	2.790072	0.714044
H	2.252625	-1.860766	-1.714445
H	2.927291	-1.659593	2.053140
H	3.604739	1.349322	-1.111262
H	1.837640	1.488121	2.483465
B	0.625971	-0.170048	-0.157860
C	-0.870223	0.902278	-0.086195
C	-1.644849	-0.281701	0.188758
C	-1.790358	-0.762778	1.613742
H	-1.250454	-0.093264	2.284356
C	-2.698040	-0.748509	-0.768146
H	-2.364565	-0.614696	-1.801009
C	-0.993612	1.529789	-1.482324
H	-0.088221	2.135807	-1.630800
H	-3.470295	0.028322	-0.622262
C	-0.905918	1.978573	1.021965
H	-0.247895	1.722120	1.860377
H	-0.489366	2.889611	0.578865
H	-2.861116	-0.634983	1.838022
H	-0.941256	0.746029	-2.249019
O	-0.078273	-1.499479	-0.466800
C	-0.147597	-2.067235	-1.745594
H	0.473857	-2.975100	-1.793728
H	-1.174027	-2.375832	-1.979063
H	0.180669	-1.382326	-2.538960
C	-1.388968	-2.200973	1.950856
H	-0.317857	-2.349023	1.792562
H	-1.620153	-2.382939	3.007751
H	-1.920361	-2.945363	1.354484
C	-3.355167	-2.109054	-0.571587
H	-4.118885	-2.248452	-1.345834
H	-2.640320	-2.934701	-0.650005
H	-3.854343	-2.181715	0.402057
C	-2.295958	2.336384	1.566317
H	-3.030732	2.458020	0.762011
H	-2.682520	1.579611	2.257700
H	-2.249674	3.283778	2.119624
C	-2.199343	2.431488	-1.759500
H	-2.166056	2.786482	-2.797859
H	-3.157029	1.916178	-1.616491
H	-2.191163	3.317651	-1.112268

58

TS-III_Et_conf_14 Eopt -845.378455

C	2.794047	1.099125	-0.833408
C	1.896995	-0.150033	-1.072084
C	1.285357	-0.190364	1.447506
C	2.098086	1.118629	1.656980
H	2.787770	1.753921	-1.714855
H	3.040132	0.876481	2.174844
C	2.809581	-1.389065	-0.841605
H	3.670200	-1.324940	-1.527119
C	2.189861	-1.430952	1.624949
H	1.571884	-2.336502	1.531231
H	1.597641	-0.156739	-2.131416
H	0.548313	-0.243353	2.257700
C	3.320916	-1.487305	0.598380
H	3.895875	-2.416567	0.722334
H	4.027536	-0.667607	0.798206
C	2.425636	1.931743	0.393701
H	1.561821	2.550713	0.128915
H	3.238635	2.638115	0.617665
H	2.295551	-2.321945	-1.089980
H	2.619113	-1.438108	2.640186
H	3.840738	0.776144	-0.739473
H	1.553311	1.780686	2.345318
B	0.627567	-0.313868	-0.038815
C	-0.849715	0.777546	-0.275885
C	-1.708280	-0.339397	0.033005
C	-2.054395	-0.728873	1.443101
H	-2.143281	-1.818384	1.467314
C	-2.609051	-0.932084	-1.005426
H	-2.086810	-1.002659	-1.963216
C	-0.961074	1.250765	-1.735865
H	-0.678085	0.428229	-2.406676
H	-3.354067	-0.129729	-1.164446
C	-0.992237	1.927280	0.735770
H	-0.973075	1.530814	1.756950
H	-0.138371	2.605138	0.670214
H	-1.256884	-0.456449	2.130987
H	-2.013756	1.479418	-1.968684
O	-0.081457	-1.674990	-0.185713
C	-0.032530	-2.460888	-1.344537
H	0.385716	-1.927134	-2.209461
H	0.571350	-3.365101	-1.171564
H	-1.035301	-2.806572	-1.623498
C	-3.372109	-0.102788	1.932846
H	-3.229312	0.936600	2.245080
H	-4.159745	-0.126676	1.169867
H	-3.728466	-0.668640	2.801002
C	-3.326686	-2.234532	-0.669008
H	-2.628374	-3.008381	-0.329220
H	-4.077626	-2.096433	0.117103
H	-3.842581	-2.609161	-1.560719
C	-2.266814	2.753084	0.518592
H	-3.152513	2.116539	0.395545
H	-2.446455	3.421680	1.370617
H	-2.191412	3.379124	-0.378887
C	-0.120746	2.466673	-2.110183
H	-0.353401	3.344337	-1.494761
H	0.947435	2.261914	-2.011990
H	-0.310732	2.739099	-3.156308

58

TS-III_Et_conf_15 Eopt -845.376512

C	2.332263	1.647271	-1.005706
C	1.705926	0.271540	-1.300754
C	1.567574	-0.171991	1.244048
C	2.196572	1.210628	1.531140
H	1.538852	2.406854	-1.070595
H	2.861186	1.118454	2.405672
C	2.735010	-0.867499	-1.453452
H	3.455088	-0.622487	-2.251838
C	2.731843	-1.201136	1.145501
H	2.357184	-2.205483	1.356277
H	1.205488	0.346848	-2.277101
H	0.996362	-0.450943	2.144233
C	3.525627	-1.225146	-0.181650
H	3.965098	-2.225475	-0.310337
H	4.383007	-0.547692	-0.101644
C	3.011823	1.788100	0.364857
H	3.208284	2.853184	0.556880
H	3.998834	1.312329	0.338487
H	2.188436	-1.754420	-1.806390
H	3.442656	-0.988932	1.960674
H	3.061668	1.909411	-1.790608
H	1.430112	1.930863	1.826708
B	0.678842	-0.194948	-0.139368
C	-0.904636	0.782206	-0.167970
C	-1.614415	-0.447945	0.057650
C	-1.814880	-1.003845	1.437744
H	-1.823720	-2.097026	1.367843
C	-2.525985	-1.002415	-1.000912
H	-2.069846	-0.843091	-1.981971
C	-1.103023	1.431057	-1.549836
H	-0.429312	2.294758	-1.586493
H	-3.400083	-0.331754	-0.966291
C	-0.904876	1.826932	0.955221
H	-0.731186	1.344194	1.924722
H	-0.047640	2.487441	0.786921
H	-0.984453	-0.723821	2.088573
H	-0.739685	0.751266	-2.326243
O	0.016555	-1.542673	-0.488051
C	0.374011	-2.781667	0.053358
H	0.351362	-2.782579	1.154035
H	-0.329925	-3.542671	-0.304375
H	1.378654	-3.090801	-0.270466
C	-3.137649	-0.537305	2.059475

H	-3.983847	-0.672147	1.374012
H	-3.338620	-1.125675	2.962054
H	-3.093018	0.517243	2.349230
C	-3.005337	-2.439997	-0.865443
H	-3.678432	-2.674320	-1.698642
H	-2.171958	-3.147516	-0.904516
H	-3.561776	-2.608587	0.064447
C	-2.136431	2.735958	1.048263
H	-2.184250	3.428650	0.199102
H	-3.079772	2.177307	1.074296
H	-2.076245	3.342693	1.961269
C	-2.524291	1.896471	-1.948221
H	-2.929217	1.281586	-2.761086
H	-3.239397	1.847031	-1.121564
H	-2.511295	2.934121	-2.303913

58

TS-III_Et_conf_16 Eopt -845.382581

C	2.362512	1.686312	-0.471118
C	1.722167	0.427869	-1.099181
C	1.410019	-0.456847	1.315847
C	2.133103	0.791656	1.859262
H	1.592361	2.437528	-0.252281
H	2.686828	0.520198	2.772914
C	2.835729	-0.628347	-1.367665
H	3.811362	-0.119014	-1.371228
C	2.469160	-1.573521	1.068466
H	2.086119	-2.525234	1.460516
H	1.294918	0.722131	-2.069707
H	0.766458	-0.821445	2.126755
C	2.893155	-1.826566	-0.394553
H	2.249551	-2.621516	-0.786900
H	3.911414	-2.240693	-0.404644
C	3.092812	1.392127	0.836774
H	3.540654	2.315173	1.232082
H	3.928792	0.698424	0.657908
H	2.719912	-1.030904	-2.383585
H	3.360914	-1.345233	1.673242
H	3.056606	2.147622	-1.192815
H	1.411172	1.562718	2.156208
B	0.614022	-0.202141	-0.092611
C	-0.926722	0.781695	-0.019205
C	-1.652063	-0.462717	0.086904
C	-1.881811	-1.107177	1.424057
H	-1.043199	-0.900947	2.085777
C	-2.591974	-0.942184	-0.980229
H	-2.602256	-2.035485	-1.008446
C	-1.239981	1.545044	-1.329591
H	-0.951018	0.928553	-2.188265
H	-2.302870	-0.586188	-1.969070
C	-0.992352	1.687240	1.218257
H	-0.740583	1.122177	2.121703
H	-0.220037	2.459724	1.129832
H	-2.731939	-0.549590	1.852440
H	-2.330419	1.665808	-1.406602
O	0.008452	-1.543620	-0.546654
C	-0.003381	-1.909639	-1.899354
H	-0.204867	-1.063646	-2.572475
H	0.955777	-2.353003	-2.201883
H	-0.776959	-2.672262	-2.061399
C	-2.189355	-2.599811	1.415168
H	-1.408342	-3.143440	0.869781
H	-2.215000	-2.973346	2.445143
H	-3.160426	-2.824050	0.957799
C	-4.004534	-0.433896	-0.646598
H	-4.024081	0.654411	-0.511002
H	-4.681735	-0.685161	-1.470583
H	-4.390588	-0.900015	0.268022
C	-2.349896	2.367732	1.406281
H	-2.616387	2.984288	0.537567
H	-3.152120	1.631190	1.550693
H	-2.336372	3.022767	2.287101
C	-0.620778	2.927855	-1.535960
H	-0.704288	3.571052	-0.650979
H	0.434566	2.871293	-1.817385
H	-1.146516	3.434819	-2.355322

58

TS-III_Et_conf_17 Eopt -845.375906

C	-2.175198	-0.742650	-1.827094
C	-1.502358	0.498002	-1.200706
C	-1.688001	-0.576291	1.139641
C	-2.407359	-1.778294	0.497671
H	-1.423865	-1.427523	-2.230801
H	-3.158778	-2.183924	1.196015
C	-2.616223	1.521304	-0.830602
H	-3.339116	1.554998	-1.662284
C	-2.639840	0.545401	1.602307
H	-2.036319	1.276745	2.160120
H	-0.922717	0.976284	-2.006182
H	-1.208332	-0.954736	2.050721
C	-3.404824	1.263818	0.475870
H	-3.772445	2.227546	0.858291
H	-4.308241	0.687828	0.246577
C	-3.074833	-1.515817	-0.857988
H	-3.352783	-2.478225	-1.312270
H	-4.018381	-0.976039	-0.718404
H	-2.185852	2.525376	-0.790703
H	-3.374002	0.148524	2.322976
H	-2.779649	-0.420785	-2.690917
H	-1.670572	-2.583637	0.353583
B	-0.625996	0.141178	0.140760
C	0.918866	-0.869414	-0.071200
C	1.686497	0.342845	-0.065519
C	2.625378	0.734352	1.033328
H	2.803659	1.813462	1.006691
C	1.902051	1.075362	-1.367876
H	0.937140	1.186610	-1.869123
C	0.911153	-1.630872	-1.407978
H	0.134918	-2.404498	-1.354096
H	2.467683	0.378329	-2.004061
C	1.086772	-1.892475	1.074080
H	0.235545	-2.575908	0.973974
H	1.967743	-2.513268	0.858668
H	2.209543	0.503200	2.008154
H	0.618049	-0.966989	-2.227633
O	0.079903	1.330909	0.805365
C	-0.170995	2.688456	0.608132
H	-1.136705	2.998133	1.037840
H	0.616971	3.259745	1.121083
H	-0.162523	2.968611	-0.457404
C	3.943464	-0.023594	0.820140
H	4.660769	0.260120	1.598841
H	3.788390	-1.108753	0.874390
H	4.391793	0.207521	-0.155397
C	2.638452	2.410331	-1.333458
H	3.678784	2.296941	-1.005612
H	2.655609	2.830835	-2.345762
H	2.150815	3.138929	-0.677001
C	1.176047	-1.468384	2.548479
H	2.214838	-1.336914	2.874460
H	0.632730	-0.541043	2.757535
H	0.743073	-2.255232	3.179026
C	2.238885	-2.312536	-1.760606
H	3.085576	-1.613683	-1.718987
H	2.463032	-3.143935	-1.081793
H	2.196779	-2.725064	-2.776947

58

TS-III_Et_conf_18 Eopt -845.377820

C	2.453315	1.562025	-0.523567
C	1.753481	0.322551	-1.116742
C	1.342473	-0.505028	1.295463
C	1.973300	0.785488	1.855748
H	1.726894	2.384841	-0.498102
H	2.469842	0.558417	2.813390
C	2.707347	-0.854426	-1.394411
H	3.512406	-0.539620	-2.079271
C	2.472301	-1.564974	1.127062
H	2.035108	-2.565281	1.190663
H	1.341222	0.612515	-2.095628
H	0.689648	-0.907649	2.083455
C	3.353886	-1.482014	-0.144624

H 3.698427 -2.495554 -0.398280
H 4.267851 -0.923614 0.087344
C 2.995360 1.412455 0.903872
H 3.283341 2.405254 1.279896
H 3.916394 0.818128 0.910087
H 2.134752 -1.618755 -1.939348
H 3.137650 -1.492187 2.003183
H 3.267769 1.895517 -1.189343
H 1.199656 1.525167 2.086682
B 0.590349 -0.249015 -0.142805
C -0.903911 0.828707 -0.198273
C -1.725784 -0.346489 -0.047241
C -1.985864 -0.961264 1.295755
H -2.029045 -2.048649 1.165140
C -2.658642 -0.793648 -1.135423
H -2.159908 -0.695549 -2.101375
C -1.100451 1.503008 -1.575261
H -0.837307 0.790765 -2.366320
H -3.448251 -0.018665 -1.133011
C -1.014609 1.838287 0.952816
H -0.959192 1.321493 1.917759
H -0.153667 2.516458 0.928154
H -1.175125 -0.744357 1.989190
H -2.176228 1.697559 -1.699564
O -0.116605 -1.478413 -0.737381
C 0.155448 -2.811701 -0.424008
H -0.511298 -3.453890 -1.014347
H 1.187010 -3.096801 -0.677701
H -0.006916 -3.033452 0.643678
C -3.309223 -0.476331 1.911936
H -3.586826 -1.148079 2.732009
H -3.204423 0.532337 2.324701
H -4.133315 -0.467050 1.188791
C -3.291292 -2.172953 -1.012702
H -3.912130 -2.366067 -1.895250
H -2.527105 -2.955937 -0.964371
H -3.933322 -2.265621 -0.129004
C -2.295663 2.677911 0.887064
H -3.183910 2.049374 0.738956
H -2.432746 3.249133 1.814448
H -2.263907 3.396288 0.058581
C -0.368910 2.816010 -1.869184
H -0.329592 3.492518 -1.006881
H 0.656847 2.651228 -2.211556
H -0.894883 3.346485 -2.673356
58
TS-III_Et_conf_19 Eopt -845.374792
C 2.037300 1.165733 -1.693847
C 1.526923 -0.235110 -1.301074
C 1.712165 0.353096 1.205433
C 2.283002 1.730895 0.810718
H 1.196014 1.811253 -1.964643
H 3.050072 2.042347 1.539967
C 2.744237 -1.195391 -1.153680
H 3.412598 -1.037813 -2.016532
C 2.781918 -0.737951 1.399631
H 2.271584 -1.642862 1.765193
H 0.965116 -0.621632 -2.164067
H 1.243345 0.488020 2.187506
C 3.583035 -1.076526 0.135315
H 4.121253 -2.022935 0.293080
H 4.365482 -0.320981 0.001932
C 2.871633 1.853298 -0.604205
H 2.979087 2.919633 -0.851974
H 3.890344 1.449701 -0.620375
H 2.400263 -2.229983 -1.245788
H 3.486097 -0.445506 2.196219
H 2.653319 1.082969 -2.604429
H 1.472397 2.469711 0.902916
B 0.667737 -0.211306 0.095761
C -0.937585 0.699600 0.185465
C -1.609662 -0.522954 -0.171872
C -2.570897 -1.270842 0.720125
H -2.828760 -2.224066 0.240067
C -1.697703 -0.955815 -1.612252
H -1.502922 -2.035883 -1.638870
C -1.041847 1.841624 -0.839544
H -0.231371 2.556437 -0.648733
H -0.939995 -0.465024 -2.219498
C -1.140231 1.281159 1.600465
H -0.352243 2.035403 1.719054
H -2.076226 1.854751 1.621194
H -2.089055 -1.509592 1.667181
H -0.876661 1.464313 -1.853433
O 0.032241 -1.559826 0.479809
C 0.350344 -2.828983 -0.002489
H 0.425159 -2.861788 -1.099736
H 1.298549 -3.201172 0.416632
H -0.445881 -3.522374 0.309064
C -3.850876 -0.464928 0.980210
H -4.384505 -0.220677 0.055500
H -4.524415 -1.059675 1.609029
H -3.639696 0.473048 1.504291
C -3.076452 -0.713469 -2.240096
H -3.034217 -1.013123 -3.293310
H -3.858266 -1.308978 -1.755891
H -3.363041 0.343548 -2.202139
C -1.123415 0.371428 2.838376
H -0.729474 0.929080 3.697412
H -2.130222 0.036432 3.114303
H -0.495782 -0.514397 2.698369
C -2.371939 2.606783 -0.803364
H -2.432893 3.280054 0.059820
H -2.481006 3.223564 -1.704545
H -3.230348 1.923446 -0.751927
58
TS-III_Et_conf_2 Eopt -845.385082
C 2.264108 1.699563 -0.413914
C 1.690303 0.438791 -1.095422
C 1.407855 -0.589440 1.255952
C 2.165827 0.595793 1.883644
H 1.447198 2.397465 -0.199650
H 2.821876 0.231192 2.692064
C 2.807728 -0.580387 -1.429153
H 3.644880 -0.052005 -1.916297
C 2.361156 -1.742627 0.861963
H 1.745473 -2.594015 0.540523
H 1.262578 0.756747 -2.059997
H 0.765793 -1.006055 2.046141
C 3.370455 -1.401741 -0.247798
H 3.791100 -2.338855 -0.641260
H 4.221499 -0.874040 0.196907
C 2.997277 1.436387 0.903126
H 3.240039 2.400450 1.373833
H 3.962595 0.958490 0.704192
H 2.424259 -1.275828 -2.185153
H 2.921013 -2.081776 1.748856
H 2.942213 2.224523 -1.107249
H 1.439125 1.262239 2.367616
B 0.590245 -0.241854 -0.116198
C -0.932417 0.777393 -0.008341
C -1.680093 -0.451473 0.086970
C -1.916770 -1.098945 1.421609
H -1.075765 -0.901966 2.084212
C -2.617461 -0.917172 -0.987800
H -2.645721 -2.010427 -1.007460
C -1.235511 1.549049 -1.316561
H -0.943889 0.928458 -2.173054
H -2.300411 -0.578025 -1.975336
C -0.958371 1.666850 1.240757
H -0.721467 1.079278 2.133521
H -0.160169 2.414161 1.164310
H -2.760985 -0.532993 1.851197
H -2.325259 1.667848 -1.397910
O -0.060403 -1.552348 -0.576496
C 0.001677 -2.033520 -1.887041
H 0.078457 -1.229811 -2.635585
H 0.863646 -2.708138 -2.013134
H -0.899964 -2.621986 -2.107333
C -2.240817 -2.587888 1.410315

H -1.463140 -3.140559 0.869097
H -2.276551 -2.961697 2.439915
H -3.211902 -2.800696 0.947541
C -4.026247 -0.387088 -0.676466
H -4.034349 0.701992 -0.545660
H -4.699083 -0.634432 -1.505248
H -4.427142 -0.843557 0.237051
C -2.293894 2.385126 1.446231
H -3.114728 1.668752 1.588673
H -2.256915 3.030942 2.333171
H -2.548758 3.016390 0.584580
C -0.622266 2.935978 -1.523914
H -1.192236 3.464530 -2.298736
H -0.652070 3.557409 -0.620003
H 0.415512 2.886157 -1.866323
58
TS-III_Et_conf_20 Eopt -845.380416
C -2.306001 0.883083 1.619451
C -1.440394 -0.333055 1.210459
C -1.659758 0.371201 -1.264246
C -2.277368 1.706853 -0.810749
H -1.665124 1.626651 2.109356
H -2.937555 2.105060 -1.598536
C -2.314394 -1.578187 0.950389
H -2.870760 -1.849804 1.862335
C -2.739207 -0.724620 -1.466121
H -2.308635 -1.513173 -2.095853
H -0.810754 -0.564861 2.082151
H -1.213065 0.544546 -2.254923
C -3.317367 -1.390369 -0.197910
H -3.732616 -2.370016 -0.476225
H -4.171505 -0.812309 0.172751
C -3.069924 1.603132 0.493987
H -3.336517 2.613648 0.836568
H -4.024467 1.103897 0.293141
H -1.656571 -2.428433 0.720219
H -3.574366 -0.304950 -2.052443
H -3.028241 0.568532 2.391870
H -1.480143 2.452410 -0.676632
B -0.578307 -0.131206 -0.163586
C 0.905063 0.921846 -0.066752
C 1.670208 -0.273296 0.215779
C 2.738080 -0.744995 -0.726143
H 2.418622 -0.611267 -1.762999
C 1.798461 -0.761407 1.640647
H 1.258042 -0.093369 2.311838
C 0.828119 1.967756 1.060478
H 0.245412 2.817255 0.677631
H 2.868281 -0.636706 1.872841
C 1.109719 1.550776 -1.454726
H 1.085158 0.763262 -2.217708
H 0.232185 2.172109 -1.666053
H 3.522668 0.016383 -0.571906
H 0.256746 1.579267 1.906834
O 0.136983 -1.452524 -0.497631
C 0.169512 -1.993346 -1.788486
H -0.567981 -2.806581 -1.881645
H -0.037088 -1.248965 -2.570248
H 1.155118 -2.430973 -1.993645
C 3.364555 -2.119382 -0.527203
H 2.630366 -2.927304 -0.612357
H 4.128754 -2.274091 -1.298111
H 3.857339 -2.206124 0.448490
C 1.392414 -2.200310 1.970530
H 0.332909 -2.361692 1.758264
H 1.962726 -2.945914 1.413326
H 1.567821 -2.369263 3.040200
C 2.343232 2.432034 -1.682486
H 2.436640 2.666655 -2.751067
H 2.252239 3.386791 -1.150836
H 3.279043 1.957252 -1.363866
C 2.168748 2.482781 1.597563
H 1.997198 3.229830 2.384256
H 2.760135 1.672341 2.042336
H 2.781462 2.953163 0.822860
58
TS-III_Et_conf_21 Eopt -845.377772
C -2.272961 1.482724 1.304415
C -1.582662 0.100815 1.305178
C -1.753753 -0.004272 -1.268169
C -2.172860 1.480432 -1.276952
H -1.535750 2.242005 1.591998
H -2.807977 1.680309 -2.155379
C -2.616532 -1.041290 1.420895
H -3.179285 -0.942522 2.363275
C -2.996189 -0.926117 -1.146410
H -2.724414 -1.921138 -1.517656
H -0.979135 0.059328 2.224640
H -1.314757 -0.219225 -2.253805
C -3.618618 -1.081975 0.258239
H -4.174632 -2.030322 0.295108
H -4.371119 -0.301301 0.420535
C -2.930695 1.919660 -0.019155
H -3.029565 3.014994 -0.024051
H -3.956575 1.537681 -0.075345
H -2.078710 -1.998004 1.479739
H -3.777016 -0.562643 -1.835746
H -3.038540 1.505269 2.098435
H -1.286324 2.115589 -1.397796
B -0.723515 -0.257050 -0.038048
C 0.899731 0.561787 -0.262545
C 1.514133 -0.639834 0.260864
C 2.342169 -1.556054 -0.597776
H 2.171020 -2.586869 -0.272525
C 1.710107 -0.865488 1.745687
H 1.922972 -1.932880 1.881880
C 0.910447 1.820979 0.611023
H 0.076584 2.455794 0.288439
H 0.766461 -0.666215 2.257141
C 1.134300 0.868823 -1.755313
H 0.693245 0.075033 -2.370492
H 0.543511 1.764633 -1.978870
H 2.038962 -1.483459 -1.645684
H 0.686353 1.549105 1.648528
O -0.169896 -1.688937 0.132699
C -0.388633 -2.710612 -0.797935
H 0.392375 -3.475079 -0.693200
H -1.351068 -3.207276 -0.601889
H -0.385317 -2.348639 -1.837310
C 3.844578 -1.268385 -0.442839
H 4.388480 -1.743577 -1.267235
H 4.072844 -0.196759 -0.457749
H 4.224733 -1.687549 0.495475
C 2.813252 -0.042122 2.429086
H 3.006868 -0.487409 3.412531
H 3.756431 -0.038570 1.875145
H 2.509676 0.995830 2.591935
C 2.558600 1.114199 -2.283293
H 3.179307 1.700508 -1.599860
H 3.089877 0.180691 -2.497111
H 2.499521 1.667767 -3.229996
C 2.179326 2.678626 0.559663
H 3.092139 2.080700 0.678314
H 2.254725 3.219902 -0.391359
H 2.158416 3.429669 1.360597
58
TS-III_Et_conf_22 Eopt -845.373623
C 2.055242 1.220959 -1.615261
C 1.399548 -0.153069 -1.359916
C 1.762708 0.104106 1.180963
C 2.442184 1.462919 0.920360
H 1.289172 1.983240 -1.782707
H 3.257280 1.621918 1.646811
C 2.509933 -1.242531 -1.409912
H 3.156008 -1.032853 -2.278580
C 2.727993 -1.096519 1.181122
H 2.152359 -1.981060 1.494917
H 0.763340 -0.359506 -2.233405
H 1.363219 0.159770 2.198833
C 3.412374 -1.379800 -0.164883

H	3.834116	-2.395791	-0.145171
H	4.277083	-0.713794	-0.264534
C	2.995606	1.686373	-0.494910
H	3.213987	2.756316	-0.628104
H	3.961824	1.179193	-0.598057
H	2.055591	-2.213677	-1.624616
H	3.508362	-0.958919	1.948362
H	2.631045	1.171340	-2.554179
H	1.703283	2.252316	1.127943
B	0.605842	-0.215556	0.083317
C	-0.858327	0.848571	0.344630
C	-1.726061	-0.212245	-0.094678
C	-2.739259	-0.832940	0.819354
H	-2.294138	-0.995062	1.797835
C	-1.945992	-0.494582	-1.550848
H	-2.058879	-1.578121	-1.673177
C	-0.868523	2.094142	-0.559724
H	0.030734	2.685601	-0.351082
H	-1.089861	-0.181884	-2.145946
C	-1.008813	1.306518	1.813493
H	-0.269696	2.106909	1.946609
H	-1.983755	1.804660	1.912980
H	-3.476537	-0.019705	0.963768
H	-0.800971	1.799200	-1.612310
O	-0.157340	-1.529479	0.354761
C	0.072877	-2.757787	-0.267498
H	-0.016106	-2.696406	-1.364526
H	1.066755	-3.165151	-0.027234
H	-0.670719	-3.479066	0.097703
C	-3.438915	-2.105358	0.359674
H	-4.027459	-1.963680	-0.553947
H	-2.713227	-2.906745	0.180187
H	-4.122640	-2.445631	1.146036
C	-3.207179	0.206577	-2.084114
H	-3.471208	-0.226250	-3.055631
H	-4.068042	0.088314	-1.415612
H	-3.030998	1.277559	-2.228859
C	-0.867775	0.312180	2.986599
H	-1.832243	0.140205	3.479510
H	-0.470017	-0.658100	2.672504
H	-0.190417	0.715531	3.749047
C	-2.090202	3.001857	-0.361284
H	-2.179492	3.709429	-1.195613
H	-3.023677	2.425225	-0.302729
H	-2.009072	3.592064	0.559291

58

TS-III_Et_conf_23 Eopt -845.381645

C	2.258544	0.909692	-1.702133
C	1.448569	-0.353744	-1.327092
C	1.697054	0.229410	1.170735
C	2.183200	1.637085	0.785035
H	1.594084	1.607005	-2.223976
H	2.837746	2.045855	1.572393
C	2.375638	-1.572567	-1.135917
H	2.883452	-1.811736	-2.084298
C	2.862517	-0.791524	1.266696
H	2.511982	-1.641595	1.866602
H	0.814401	-0.588986	-2.195047
H	1.289600	0.285366	2.183800
C	3.433286	-1.345916	-0.051836
H	3.954276	-2.291987	0.156862
H	4.202171	-0.671404	-0.447766
C	2.943526	1.670922	-0.546227
H	3.094577	2.716948	-0.850373
H	3.950565	1.271395	-0.376651
H	1.762178	-2.448644	-0.878763
H	3.684576	-0.341914	1.849378
H	3.030458	0.636802	-2.441655
H	1.316881	2.312908	0.726906
B	0.606457	-0.238099	0.068399
C	-0.895084	0.869138	-0.036584
C	-1.680848	-0.330172	-0.138057
C	-2.621153	-0.745884	0.951778
H	-2.681823	-1.836659	1.007756
C	-1.949582	-0.968643	-1.473829
H	-1.063246	-0.871919	-2.101653
C	-0.841121	1.727124	-1.313978
H	-0.035191	2.461898	-1.192370
H	-2.717044	-0.334240	-1.946657
C	-1.230451	1.800287	1.160788
H	-0.839472	2.792896	0.901416
H	-2.320227	1.935356	1.176135
H	-2.293190	-0.376690	1.923977
H	-0.572175	1.122267	-2.184798
O	-0.098914	-1.568982	0.323520
C	-0.040062	-2.245613	1.543936
H	0.835110	-2.912870	1.580665
H	0.007391	-1.567724	2.410082
H	-0.932160	-2.878585	1.652989
C	-4.016459	-0.184411	0.627536
H	-3.994844	0.901372	0.474522
H	-4.432089	-0.646507	-0.275778
H	-4.694238	-0.395500	1.462121
C	-2.412192	-2.420210	-1.456318
H	-1.687570	-3.042979	-0.918357
H	-3.398008	-2.543158	-0.992241
H	-2.484717	-2.791257	-2.485225
C	-0.780850	1.507458	2.599645
H	0.215897	1.914227	2.799907
H	-1.474900	1.990186	3.299695
H	-0.752742	0.441108	2.847524
C	-2.139496	2.483060	-1.613279
H	-2.994872	1.800824	-1.711385
H	-2.378964	3.208271	-0.825749
H	-2.049051	3.039070	-2.555533

58

TS-III_Et_conf_24 Eopt -845.375934

C	2.216492	1.826235	-0.902308
C	1.508520	0.510872	-1.287180
C	1.719962	-0.346454	1.136634
C	2.212575	1.056380	1.552388
H	1.473541	2.633693	-0.893540
H	2.878615	0.967798	2.426223
C	2.517536	-0.562933	-1.754550
H	3.080907	-0.191572	-2.626086
C	2.906262	-1.253494	0.717881
H	2.573888	-2.297824	0.761074
H	0.891672	0.738323	-2.168384
H	1.292252	-0.804996	2.038598
C	3.519993	-0.996372	-0.674694
H	4.032175	-1.911049	-1.008068
H	4.307955	-0.238201	-0.600599
C	2.948115	1.826799	0.451603
H	3.093890	2.866528	0.779446
H	3.957633	1.415919	0.338850
H	1.952815	-1.439346	-2.102454
H	3.705512	-1.170300	1.473705
H	2.936761	2.099053	-1.692203
H	1.360225	1.661734	1.887579
B	0.659707	-0.210675	-0.088410
C	-0.927534	0.546836	0.424436
C	-1.605835	-0.376746	-0.454513
C	-2.523354	-1.467271	0.031609
H	-2.760511	-2.134170	-0.807122
C	-1.754831	-0.094429	-1.930866
H	-1.634621	-1.052336	-2.448844
C	-0.966093	2.027437	0.007773
H	-0.126202	2.543136	0.489432
H	-0.961781	0.564242	-2.278651
C	-1.164776	0.464057	1.950026
H	-0.375154	1.076383	2.399486
H	-2.092814	1.000001	2.191334
H	-2.025936	-2.071019	0.791210
H	-0.793365	2.123091	-1.069049
O	0.064201	-1.503787	-0.678049
C	0.248561	-2.792863	-0.177555
H	-0.614430	-3.414999	-0.456570
H	1.144130	-3.258472	-0.620464
H	0.356777	-2.823129	0.916644
C	-3.822850	-0.895985	0.618630

H -4.425326 -1.720700 1.018054
H -3.627733 -0.193738 1.435389
H -4.424002 -0.376718 -0.133753
C -3.110751 0.505307 -2.321855
H -3.931690 -0.202346 -2.163454
H -3.330084 1.430252 -1.777202
H -3.084636 0.744436 -3.391266
C -1.204282 -0.869369 2.709452
H -0.885172 -0.702827 3.745943
H -2.213997 -1.293165 2.753955
H -0.539698 -1.627288 2.286701
C -2.256870 2.767041 0.388252
H -2.270504 3.038018 1.450764
H -2.348998 3.698327 -0.184831
H -3.149286 2.156660 0.193624
58
TS-III_Et_conf_25 Eopt -845.378270
C -2.358887 1.489387 1.024150
C -1.812832 0.057280 1.188273
C -1.458618 -0.052187 -1.365437
C -2.273639 1.251867 -1.534017
H -1.534008 2.210789 1.108378
H -2.964454 1.141200 -2.387147
C -2.947540 -0.993715 1.074629
H -3.808617 -0.658747 1.677796
C -2.374398 -1.293617 -1.442132
H -1.748594 -2.194692 -1.377137
H -1.424775 -0.021614 2.214970
H -0.781804 -0.117181 -2.231212
C -3.448932 -1.336378 -0.345816
H -3.902268 -2.338359 -0.326518
H -4.263190 -0.659008 -0.628261
C -3.076988 1.722889 -0.307396
H -3.295833 2.795121 -0.416388
H -4.055005 1.229454 -0.274527
H -2.604902 -1.920614 1.550013
H -2.875521 -1.331634 -2.423105
H -3.053094 1.723312 1.847753
H -1.594445 2.065860 -1.817002
B -0.672832 -0.198904 0.059311
C 0.829005 0.766464 0.339703
C 1.596526 -0.365466 -0.150216
C 1.851877 -0.491408 -1.631752
H 1.558946 -1.500062 -1.945525
C 2.531218 -1.162749 0.714835
H 2.143646 -1.210841 1.735226
C 0.955388 1.055081 1.843808
H 0.095806 1.667491 2.139707
H 3.432402 -0.530860 0.779531
C 0.842504 2.057800 -0.500999
H 0.281501 1.917852 -1.427667
H 0.270319 2.797007 0.074535
H 1.220753 0.210714 -2.175748
H 0.847303 0.122867 2.411659
O 0.010534 -1.589144 0.072806
C -0.128729 -2.454692 1.168279
H -0.324031 -1.920554 2.108611
H -0.955415 -3.159704 0.989560
H 0.779572 -3.053174 1.301103
C 3.328199 -0.253657 -2.004937
H 3.784770 0.545018 -1.411588
H 3.931880 -1.155645 -1.877327
H 3.387863 0.038671 -3.059437
C 2.918591 -2.557987 0.210693
H 3.798303 -2.534425 -0.437806
H 3.161009 -3.206810 1.060268
H 2.099134 -3.024424 -0.347724
C 2.207459 2.663820 -0.871532
H 2.555346 2.290163 -1.841212
H 2.130378 3.755345 -0.958983
H 2.985388 2.443804 -0.132135
C 2.217284 1.787652 2.307254
H 3.139015 1.283225 1.990287
H 2.244731 2.814196 1.920513
H 2.232855 1.851625 3.403188
58
TS-III_Et_conf_26 Eopt -845.380663
C 2.450815 1.458283 -0.506252
C 1.674704 0.287080 -1.141482
C 1.239897 -0.627797 1.234828
C 2.193406 0.438020 1.816184
H 1.759928 2.287107 -0.310386
H 2.789783 -0.008987 2.629539
C 2.604018 -0.917700 -1.438894
H 3.512360 -0.556538 -1.950646
C 1.989358 -1.932064 0.884989
H 1.247586 -2.683817 0.581219
H 1.298560 0.637939 -2.116538
H 0.540961 -0.881920 2.044390
C 3.036099 -1.782735 -0.232958
H 3.306284 -2.783205 -0.601348
H 3.959397 -1.380837 0.198570
C 3.141810 1.112645 0.813733
H 3.537973 2.035346 1.262766
H 4.017585 0.483608 0.620989
H 2.100615 -1.568335 -2.163951
H 2.491001 -2.332437 1.781333
H 3.199624 1.844501 -1.217749
H 1.595120 1.227607 2.289660
B 0.483536 -0.190988 -0.149491
C -0.870014 0.997745 -0.018355
C -1.776283 -0.119433 0.174303
C -2.011503 -0.693800 1.549586
H -1.421211 -0.154171 2.288695
C -2.852404 -0.400961 -0.831135
H -2.470495 -0.269732 -1.845885
C -1.138916 1.746367 -1.345635
H -0.978748 1.058326 -2.185948
H -3.532704 0.458262 -0.678701
C -0.723684 1.956793 1.171653
H -0.439848 1.416591 2.077790
H 0.111162 2.638439 0.972832
H -3.066323 -0.450493 1.759281
H -2.205299 2.015099 -1.385026
O -0.341765 -1.399992 -0.627182
C -0.400053 -1.816407 -1.960903
H -1.422729 -2.121761 -2.221518
H -0.091411 -1.033179 -2.668367
H 0.244795 -2.696416 -2.117431
C -1.799797 -2.194720 1.772892
H -1.962556 -2.409877 2.836056
H -2.486692 -2.816337 1.195858
H -0.780207 -2.483895 1.507077
C -3.658358 -1.688464 -0.734243
H -4.194342 -1.770124 0.218754
H -4.405844 -1.697170 -1.536332
H -3.027193 -2.576229 -0.851255
C -1.989532 2.770864 1.440580
H -2.274456 3.373464 0.567823
H -2.836045 2.111654 1.681845
H -1.846896 3.455790 2.286454
C -0.353514 3.023225 -1.643330
H -0.810410 3.525812 -2.505733
H -0.368947 3.736055 -0.809097
H 0.689759 2.823086 -1.903465
58
TS-III_Et_conf_27 Eopt -845.376654
C 2.200399 1.668658 -0.983799
C 1.761388 0.209570 -1.232271
C 1.578010 -0.096467 1.327822
C 2.274081 1.265360 1.542596
H 1.320360 2.326585 -0.943053
H 2.997750 1.183029 2.371217
C 2.990863 -0.736827 -1.261754
H 3.787626 -0.266058 -1.862091
C 2.603392 -1.250394 1.273364
H 2.054373 -2.200098 1.215411
H 1.311591 0.164136 -2.236750
H 0.945546 -0.284380 2.211370
C 3.594478 -1.155152 0.100519

H	4.094011	-2.127388	-0.021838
H	4.393584	-0.458361	0.378126
C	2.988146	1.856234	0.314559
H	3.154231	2.930302	0.482492
H	3.987468	1.422011	0.197909
H	2.714453	-1.642507	-1.813299
H	3.180639	-1.285108	2.211660
H	2.810853	2.025280	-1.829149
H	1.529797	2.000759	1.874535
B	0.733591	-0.230755	-0.059926
C	-0.885258	0.625775	-0.159810
C	-1.496610	-0.653900	0.130602
C	-1.748107	-1.080104	1.561839
H	-1.282388	-2.064889	1.678858
C	-2.289728	-1.439103	-0.883595
H	-2.426448	-2.454014	-0.493337
C	-1.109344	1.184613	-1.579046
H	-0.189267	1.673545	-1.911779
H	-1.730934	-1.529722	-1.817186
C	-0.969494	1.719065	0.920448
H	-0.373766	1.443354	1.793402
H	-0.476208	2.606200	0.501147
H	-1.246841	-0.401653	2.256088
H	-1.259043	0.360774	-2.287455
O	0.186932	-1.670111	-0.112734
C	0.425986	-2.570408	-1.155265
H	-0.368816	-3.328838	-1.166232
H	0.460669	-2.084620	-2.142495
H	1.378646	-3.097995	-0.993518
C	-3.229506	-1.216852	1.936907
H	-3.727449	-1.989788	1.339102
H	-3.292524	-1.518483	2.988798
H	-3.786944	-0.281819	1.821808
C	-3.657176	-0.792423	-1.179561
H	-3.979929	-0.104247	-0.390235
H	-3.624984	-0.228410	-2.117286
H	-4.424033	-1.568670	-1.278757
C	-2.370564	2.106394	1.420568
H	-2.666688	1.484344	2.273079
H	-2.383019	3.148654	1.765694
H	-3.141307	2.001999	0.649625
C	-2.225948	2.218157	-1.764911
H	-3.217342	1.841135	-1.493250
H	-2.035088	3.120056	-1.168219
H	-2.262023	2.526374	-2.818172

58

TS-III_Et_conf_28 Eopt -845.377068

C	-2.311424	1.109546	1.291806
C	-1.770725	-0.314499	1.073358
C	-1.368942	0.228998	-1.412561
C	-2.205484	1.522960	-1.271197
H	-1.485091	1.757024	1.619057
H	-2.927758	1.576665	-2.103557
C	-2.885225	-1.307974	0.650850
H	-3.748633	-1.188373	1.327443
C	-2.259689	-0.965178	-1.815046
H	-1.629170	-1.861201	-1.914730
H	-1.422393	-0.671054	2.048011
H	-0.688012	0.396343	-2.259096
C	-3.378175	-1.234494	-0.805293
H	-3.882243	-2.179190	-1.057481
H	-4.144694	-0.457467	-0.911992
C	-2.979417	1.716631	0.051285
H	-3.137788	2.792370	0.217083
H	-3.985126	1.289275	-0.039221
H	-2.515561	-2.327341	0.825517
H	-2.708476	-0.784150	-2.805324
H	-3.043072	1.113813	2.116520
H	-1.541681	2.383165	-1.413196
B	-0.596915	-0.246143	-0.048317
C	0.844007	0.765177	0.440150
C	1.701034	-0.208956	-0.194779
C	1.996629	-0.172011	-1.668345
H	2.141504	-1.201097	-2.007134
C	2.661786	-1.034294	0.609110
H	2.169297	-1.409285	1.508338
C	1.086601	0.986552	1.953446
H	2.143927	1.264839	2.073081
H	3.388530	-0.290135	0.983360
C	0.805352	2.144721	-0.242390
H	0.729607	2.031500	-1.329022
H	-0.111122	2.657992	0.072716
H	1.153308	0.232926	-2.224365
H	0.535630	1.894898	2.224813
O	0.150913	-1.561245	-0.341769
C	0.014991	-2.710958	0.443010
H	0.990097	-3.201135	0.574258
H	-0.392978	-2.504206	1.441236
H	-0.651679	-3.436626	-0.051150
C	3.254543	0.654185	-1.983931
H	3.610078	0.388863	-2.986103
H	3.040535	1.727575	-1.977216
H	4.071239	0.463439	-1.276471
C	3.396740	-2.161384	-0.106632
H	4.096030	-1.783892	-0.861661
H	3.973925	-2.742263	0.621982
H	2.698921	-2.842455	-0.607814
C	1.994442	3.051684	0.103793
H	2.044609	3.899750	-0.591300
H	1.903742	3.464460	1.115623
H	2.950808	2.514737	0.053745
C	0.758137	-0.094396	3.001297
H	1.586737	-0.203024	3.712295
H	-0.132938	0.175386	3.580068
H	0.566071	-1.079298	2.569842

58

TS-III_Et_conf_29 Eopt -845.378349

C	2.139263	1.312085	-1.343926
C	1.557775	-0.112182	-1.254269
C	1.479839	0.053612	1.333622
C	2.114230	1.454659	1.253093
H	1.353930	2.019297	-1.620487
H	2.823562	1.596973	2.085789
C	2.731977	-1.126713	-1.152640
H	3.487744	-0.859625	-1.909601
C	2.513849	-1.086166	1.439040
H	1.962909	-2.027073	1.587905
H	1.048431	-0.318162	-2.211778
H	0.884885	0.015928	2.259436
C	3.436000	-1.224436	0.217821
H	3.960605	-2.189664	0.274311
H	4.224086	-0.465908	0.283391
C	2.835664	1.803136	-0.063182
H	2.950030	2.896067	-0.120620
H	3.858282	1.410851	-0.036867
H	2.384304	-2.123953	-1.436496
H	3.137920	-0.951715	2.337691
H	2.869988	1.350367	-2.168451
H	1.320646	2.196092	1.416086
B	0.582996	-0.297724	0.035761
C	-1.037202	0.687719	0.025606
C	-1.681104	-0.566672	-0.229545
C	-2.524589	-1.378833	0.727957
H	-3.545571	-0.983158	0.602004
C	-1.941446	-0.974898	-1.658314
H	-1.832326	-2.059937	-1.752939
C	-1.102075	1.712559	-1.136159
H	-0.518004	1.355591	-1.989955
H	-1.220796	-0.507452	-2.332955
C	-1.338612	1.344672	1.376124
H	-1.094753	0.669720	2.197016
H	-0.672970	2.203030	1.506629
H	-2.558586	-2.392986	0.308857
H	-2.135318	1.791570	-1.501612
O	-0.028563	-1.687352	0.184711
C	0.230051	-2.834451	-0.562786
H	0.358918	-2.630238	-1.636240
H	1.128891	-3.356829	-0.197919
H	-0.620211	-3.524289	-0.443159
C	-2.197120	-1.483576	2.215316

H	-2.564944	-0.630874	2.794748
H	-2.686940	-2.380281	2.613943
H	-1.118703	-1.590022	2.373548
C	-3.376916	-0.605450	-2.069084
H	-4.103545	-1.281729	-1.604603
H	-3.640934	0.420591	-1.786062
H	-3.480509	-0.694415	-3.156181
C	-2.782810	1.830862	1.513815
H	-2.928408	2.360029	2.465014
H	-3.052623	2.522823	0.704895
H	-3.498737	1.000107	1.484456
C	-0.649661	3.131940	-0.786414
H	-1.371767	3.641835	-0.137986
H	0.321250	3.153400	-0.278660
H	-0.559878	3.724258	-1.706097

58

TS-III_Et_conf_3 Eopt -845.377398

C	-2.293581	1.634501	0.813473
C	-1.748202	0.234103	1.154863
C	-1.462049	-0.197564	-1.381794
C	-2.164424	1.146659	-1.675354
H	-1.460014	2.355086	0.765658
H	-2.788014	1.035002	-2.577439
C	-2.913557	-0.806298	1.178511
H	-3.874858	-0.272138	1.160550
C	-2.533907	-1.327827	-1.338771
H	-2.189850	-2.182739	-1.937132
H	-1.334535	0.263587	2.165789
H	-0.822940	-0.407667	-2.250385
C	-2.905229	-1.857294	0.056130
H	-2.193713	-2.649247	0.317130
H	-3.891201	-2.341832	0.010278
C	-3.036306	1.649652	-0.524862
H	-3.393395	2.665558	-0.746760
H	-3.938342	1.024267	-0.448580
H	-2.904133	-1.339909	2.139685
H	-3.444956	-0.964322	-1.841081
H	-2.969033	1.987498	1.609879
H	-1.429311	1.919819	-1.916038
B	-0.648250	-0.186215	0.039351
C	0.902916	0.881516	-0.028044
C	1.639952	-0.351027	-0.123861
C	1.893441	-1.002317	-1.457666
H	1.011154	-0.879614	-2.086839
C	2.565168	-0.790962	0.969956
H	2.575604	-1.882219	1.052422
C	1.245161	1.800644	1.177965
H	2.339512	1.882146	1.234574
H	2.269178	-0.379134	1.934548
C	0.964607	1.749081	-1.302670
H	0.658069	1.182693	-2.187114
H	0.241403	2.564400	-1.190976
H	2.683086	-0.400259	-1.934050
H	0.912445	2.808760	0.896227
O	0.003295	-1.545635	0.300682
C	-0.000432	-2.109962	1.581034
H	0.595325	-3.032824	1.562733
H	0.423053	-1.442586	2.345554
H	-1.015628	-2.372935	1.908252
C	2.305330	-2.469117	-1.430257
H	3.283414	-2.624120	-0.959499
H	1.556234	-3.063251	-0.893881
H	2.371606	-2.846932	-2.457180
C	3.980324	-0.291978	0.627909
H	4.369165	-0.769633	-0.278817
H	4.002067	0.794097	0.477223
H	4.654496	-0.532061	1.457556
C	2.336543	2.382137	-1.560803
H	3.131099	1.629753	-1.651344
H	2.317658	2.957272	-2.495714
H	2.620958	3.072725	-0.757152
C	0.730010	1.557997	2.601611
H	1.363804	2.113228	3.305463
H	-0.293235	1.924680	2.730015
H	0.744128	0.507340	2.910000

58

TS-III_Et_conf_30 Eopt -845.376974

C	-2.123087	0.983677	1.631012
C	-1.373738	-0.350769	1.333008
C	-1.792634	0.014063	-1.207748
C	-2.692295	1.236417	-0.883580
H	-1.581622	1.547382	2.403383
H	-3.746491	0.924620	-0.868442
C	-2.416686	-1.501148	1.341636
H	-3.016333	-1.421428	2.262735
C	-2.682774	-1.254901	-1.213854
H	-2.081689	-2.129809	-1.494509
H	-0.694714	-0.544863	2.177399
H	-1.376639	0.123247	-2.221467
C	-3.369744	-1.488370	0.139261
H	-3.936603	-2.430450	0.111691
H	-4.119600	-0.698783	0.294735
C	-2.359298	1.926499	0.441001
H	-1.461523	2.530982	0.284591
H	-3.157443	2.638265	0.699090
H	-1.922763	-2.473267	1.403661
H	-3.460707	-1.154489	-1.988262
H	-3.097866	0.740397	2.082430
H	-2.639350	1.980433	-1.689317
B	-0.625606	-0.318914	-0.117326
C	0.868149	0.838000	-0.161456
C	1.705799	-0.326677	-0.138460
C	2.507137	-0.704333	-1.350619
H	1.878596	-0.602375	-2.238916
C	2.111191	-1.003465	1.140466
H	2.228763	-2.070277	0.929494
C	1.006175	1.725939	1.085465
H	0.136851	2.380048	1.189545
H	1.324433	-0.905977	1.892491
C	0.980463	1.641707	-1.473567
H	2.042500	1.884233	-1.634349
H	0.691590	1.008483	-2.320072
H	3.256136	0.105026	-1.431821
H	1.015450	1.107344	1.989388
O	0.101935	-1.599135	-0.555097
C	-0.004690	-2.862146	0.032104
H	0.802078	-3.499268	-0.353581
H	0.081917	-2.832247	1.128721
H	-0.955486	-3.355579	-0.220736
C	3.210479	-2.052923	-1.365907
H	3.934843	-2.158559	-0.549428
H	2.491154	-2.876306	-1.302838
H	3.757440	-2.162783	-2.309676
C	3.439391	-0.477981	1.706211
H	3.831328	-1.205391	2.426192
H	4.195042	-0.340598	0.922310
H	3.308636	0.474777	2.227781
C	0.196400	2.947181	-1.593543
H	0.553551	3.497540	-2.473611
H	-0.871947	2.772521	-1.735880
H	0.319092	3.605021	-0.724948
C	2.261059	2.609412	1.046365
H	2.151443	3.432577	0.330334
H	2.452241	3.054531	2.031562
H	3.152455	2.041576	0.750951

58

TS-III_Et_conf_4 Eopt -845.385325

C	-2.314020	0.799555	1.571829
C	-1.492173	-0.452930	1.207418
C	-1.603355	0.189097	-1.296555
C	-2.282650	1.519812	-0.923907
H	-1.645197	1.524229	2.051795
H	-2.988186	1.806978	-1.720783
C	-2.390547	-1.678579	0.931801
H	-2.995841	-1.909219	1.823610
C	-2.669096	-0.914037	-1.529078
H	-2.212550	-1.732943	-2.096086
H	-0.898647	-0.709896	2.098508
H	-1.105309	0.346754	-2.266188
C	-3.332257	-1.504796	-0.268409

H	-3.770051	-2.480752	-0.525093
H	-4.180412	-0.879853	0.034050
C	-3.043481	1.503814	0.410505
H	-3.259124	2.541434	0.707041
H	-4.024898	1.042513	0.254585
H	-1.741643	-2.549716	0.761547
H	-3.460070	-0.511184	-2.183839
H	-3.059904	0.538506	2.341501
H	-1.531467	2.316637	-0.896749
B	-0.578692	-0.287961	-0.128132
C	0.919906	0.815553	0.043884
C	1.700911	-0.377461	0.218048
C	2.702922	-0.852659	-0.790031
H	2.707826	-1.945246	-0.833182
C	1.898459	-0.946688	1.594383
H	1.014260	-0.753519	2.202176
C	0.906084	1.727297	1.293218
H	0.418780	1.212539	2.127849
H	2.697621	-0.325063	2.035849
C	1.128523	1.584806	-1.267415
H	1.020662	0.903421	-2.119662
H	0.301729	2.292636	-1.371129
H	2.470192	-0.479277	-1.790100
H	1.946311	1.893974	1.618239
O	0.144509	-1.613550	-0.370993
C	0.184235	-2.279498	-1.598396
H	0.143920	-1.595369	-2.460322
H	1.111637	-2.864439	-1.669617
H	-0.647617	-2.995814	-1.683225
C	4.102566	-0.382952	-0.346666
H	4.100250	0.657353	-0.002275
H	4.484360	-1.006061	0.469830
H	4.798817	-0.460919	-1.189283
C	2.269186	-2.422679	1.675582
H	3.245171	-2.639382	1.226462
H	2.314541	-2.730488	2.726597
H	1.507230	-3.029010	1.171551
C	2.441740	2.363988	-1.407370
H	3.275517	1.718170	-1.703422
H	2.343249	3.138319	-2.179509
H	2.722337	2.865280	-0.471235
C	0.262218	3.101050	1.121860
H	-0.788852	3.037029	0.822661
H	0.301224	3.642818	2.075735
H	0.786496	3.711595	0.376516

58

TS-III_Et_conf_5 Eopt -845.376854

C	-2.301597	1.430246	1.329816
C	-1.566950	0.074097	1.292674
C	-1.529009	0.146647	-1.293815
C	-2.006150	1.612052	-1.224442
H	-1.607915	2.196914	1.695594
H	-2.564701	1.868159	-2.139448
C	-2.563311	-1.104313	1.253360
H	-3.211916	-1.075423	2.144065
C	-2.744012	-0.821455	-1.335492
H	-2.419598	-1.773924	-1.768530
H	-1.013531	-0.026882	2.240769
H	-0.993026	0.014785	-2.248455
C	-3.458541	-1.116994	0.004112
H	-3.953406	-2.096387	-0.071452
H	-4.271521	-0.397637	0.156235
C	-2.889776	1.915219	-0.009425
H	-3.057871	3.000581	0.048260
H	-3.881520	1.477456	-0.171937
H	-1.997446	-2.043675	1.318665
H	-3.489533	-0.422100	-2.043537
H	-3.113071	1.388994	2.076256
H	-1.139029	2.282751	-1.200168
B	-0.603944	-0.156036	-0.001493
C	1.002823	0.800925	0.011447
C	1.658882	-0.457854	0.272941
C	2.475065	-1.251627	-0.711743
H	3.515164	-0.935963	-0.517932
C	1.914492	-0.831517	1.714588
H	1.019319	-0.580340	2.289493
C	0.966552	1.816131	1.167178
H	0.226972	2.579886	0.898056
H	2.697735	-0.139755	2.062598
C	1.323119	1.511309	-1.318401
H	0.924497	0.944922	-2.162944
H	0.768500	2.456456	-1.320632
H	2.439434	-2.292159	-0.378964
H	0.587259	1.352114	2.080763
O	-0.003769	-1.559824	0.107042
C	-0.260003	-2.651149	-0.726272
H	-0.421963	-2.367674	-1.773884
H	0.596772	-3.340761	-0.690610
H	-1.145723	-3.207552	-0.378592
C	2.176503	-1.218570	-2.210679
H	2.541654	-2.148255	-2.662667
H	1.103612	-1.151360	-2.411757
H	2.668530	-0.388703	-2.725028
C	2.318139	-2.267402	2.018780
H	3.307834	-2.519992	1.620205
H	2.356284	-2.407892	3.105306
H	1.581421	-2.972040	1.612488
C	2.796837	1.861740	-1.594650
H	2.950611	2.013692	-2.671465
H	3.098565	2.787406	-1.096053
H	3.491764	1.078740	-1.270478
C	2.284735	2.537150	1.488129
H	2.479970	3.354776	0.786051
H	2.237914	2.979067	2.491973
H	3.151051	1.862730	1.460249

58

TS-III_Et_conf_6 Eopt -845.382866

C	-2.175224	0.925947	1.705065
C	-1.406293	-0.369905	1.331335
C	-1.784575	0.124343	-1.190647
C	-2.607016	1.391730	-0.815910
H	-1.666304	1.427003	2.540662
H	-3.677691	1.156841	-0.905425
C	-2.384721	-1.561826	1.214800
H	-2.896746	-1.717647	2.178188
C	-2.796031	-1.055301	-1.245580
H	-2.328220	-1.967980	-1.624878
H	-0.735859	-0.612293	2.167171
H	-1.389533	0.258816	-2.209148
C	-3.428210	-1.357135	0.115788
H	-4.069792	-2.246652	0.035606
H	-4.095214	-0.529962	0.403248
C	-2.345599	1.961670	0.581130
H	-1.441339	2.575934	0.531959
H	-3.156465	2.655497	0.847679
H	-1.811400	-2.477319	1.005370
H	-3.591609	-0.802499	-1.964919
H	-3.169267	0.654350	2.094120
H	-2.438018	2.188100	-1.552553
B	-0.637076	-0.291085	-0.100443
C	0.910207	0.780284	-0.038902
C	1.673791	-0.431577	0.044208
C	2.554274	-0.882934	-1.079663
H	2.653365	-1.970516	-1.080058
C	1.979110	-1.065542	1.373998
H	1.110122	-0.960101	2.024663
C	1.003922	1.662750	1.215597
H	0.247234	2.449355	1.171396
H	2.763778	-0.439035	1.827289
C	1.109644	1.560474	-1.355836
H	2.183654	1.695644	-1.548660
H	0.731991	0.957755	-2.191821
H	2.157220	-0.580143	-2.051170
H	0.771978	1.081948	2.114348
O	0.026214	-1.644044	-0.380069
C	0.011064	-2.327979	-1.599924
H	-0.197817	-1.672683	-2.458980
H	0.982854	-2.809984	-1.769140
H	-0.737908	-3.134338	-1.586856
C	3.949063	-0.270191	-0.861734

H 3.911107 0.822077 -0.778703
H 4.414507 -0.664985 0.050031
H 4.592329 -0.525336 -1.711363
C 2.429921 -2.520589 1.346584
H 2.511256 -2.895448 2.373327
H 1.695711 -3.137525 0.814279
H 3.410230 -2.646844 0.871357
C 0.462449 2.939573 -1.437617
H -0.624722 2.891352 -1.345566
H 0.837852 3.621745 -0.664640
H 0.690785 3.391272 -2.411447
C 2.371173 2.334534 1.377706
H 3.177276 1.600769 1.507960
H 2.621775 2.947129 0.501311
H 2.376857 2.993523 2.255722
58
TS-III_Et_conf_7 Eopt -845.375250
C 2.311462 1.810989 -0.827916
C 1.752688 0.445281 -1.267804
C 1.602082 -0.261596 1.214650
C 2.151098 1.117382 1.642980
H 1.485082 2.537850 -0.830313
H 2.791903 0.983195 2.529838
C 2.836912 -0.625107 -1.515165
H 3.551423 -0.271555 -2.276888
C 2.800074 -1.234973 1.022105
H 2.443262 -2.267200 1.077697
H 1.259171 0.594393 -2.238765
H 1.038975 -0.651571 2.077633
C 3.627554 -1.064503 -0.270804
H 4.132783 -2.016491 -0.491422
H 4.437350 -0.348001 -0.094893
C 2.962027 1.845679 0.562478
H 3.099726 2.894208 0.864807
H 3.972070 1.424028 0.512877
H 2.336857 -1.500630 -1.955228
H 3.479865 -1.120043 1.882364
H 3.037488 2.182500 -1.570505
H 1.332654 1.766126 1.968529
B 0.740630 -0.193579 -0.176508
C -0.918183 0.669452 -0.147024
C -1.534704 -0.629247 -0.048778
C -1.697615 -1.338720 1.277656
H -1.882896 -2.397781 1.057222
C -2.348415 -1.244917 -1.160032
H -2.056673 -2.297095 -1.254282
C -1.093043 1.399629 -1.487153
H -0.378717 2.231857 -1.505187
H -2.131571 -0.761417 -2.114349
C -1.010760 1.585837 1.075203
H -0.714287 1.026710 1.970233
H -0.261491 2.377685 0.957571
H -0.753252 -1.284308 1.824086
H -0.774364 0.731556 -2.294165
O 0.164499 -1.524889 -0.680383
C 0.484531 -2.822364 -0.282795
H -0.271826 -3.505281 -0.699587
H 1.464497 -3.138195 -0.673782
H 0.492469 -2.944210 0.810727
C -2.809286 -0.834016 2.212599
H -3.761829 -0.671147 1.701390
H -2.970946 -1.594563 2.985864
H -2.528805 0.094900 2.716109
C -3.856116 -1.197124 -0.866302
H -4.405128 -1.449670 -1.780534
H -4.132377 -1.926650 -0.096112
H -4.190993 -0.207342 -0.531816
C -2.372827 2.260243 1.316542
H -3.210912 1.616799 1.019196
H -2.494941 2.505329 2.379881
H -2.460737 3.198760 0.759291
C -2.472811 1.957014 -1.857829
H -3.264739 1.204571 -1.778941
H -2.761416 2.811476 -1.238877
H -2.454826 2.304585 -2.899730
58
TS-III_Et_conf_8 Eopt -845.379556
C -2.346486 0.510159 1.526102
C -1.333525 -0.596136 1.153475
C -1.536101 0.082315 -1.336731
C -2.418313 1.281550 -0.957912
H -1.806971 1.316691 2.036682
H -3.161444 1.460516 -1.752502
C -2.032421 -1.938075 0.855394
H -2.596087 -2.279716 1.739007
C -2.395706 -1.183501 -1.590663
H -1.789082 -1.919771 -2.131402
H -0.711713 -0.754652 2.047478
H -1.062702 0.335484 -2.298516
C -2.985592 -1.875224 -0.345973
H -3.286896 -2.896408 -0.622169
H -3.911158 -1.373645 -0.041173
C -3.165655 1.131439 0.375933
H -3.524733 2.121423 0.695846
H -4.073078 0.541462 0.206532
H -1.260839 -2.697429 0.660727
H -3.222572 -0.923870 -2.273171
H -3.052431 0.116809 2.277036
H -1.796818 2.184490 -0.929393
B -0.451821 -0.254459 -0.172062
C 0.844042 1.021030 0.070820
C 1.774095 -0.050319 0.344183
C 2.929508 -0.319628 -0.571104
H 2.631623 -0.188972 -1.615074
C 1.936554 -0.577537 1.750036
H 1.288021 -0.037534 2.437372
C 0.639514 1.983343 1.269093
H 0.193061 1.449446 2.111420
H 2.969492 -0.299927 2.019320
C 1.042262 1.772711 -1.253842
H 1.125145 1.058349 -2.082585
H 0.119030 2.326323 -1.455630
H 3.579794 0.544499 -0.350422
H 1.630719 2.315375 1.619520
O 0.455260 -1.449840 -0.493297
C 0.667994 -1.922068 -1.793590
H 0.098507 -2.849641 -1.962862
H 0.381644 -1.195773 -2.567217
H 1.726096 -2.170050 -1.945258
C 3.761931 -1.583142 -0.392489
H 4.570946 -1.578870 -1.132520
H 4.223200 -1.628235 0.601125
H 3.175708 -2.495905 -0.537728
C 1.741933 -2.075231 2.003788
H 2.412246 -2.705025 1.416205
H 1.932819 -2.271363 3.066066
H 0.715722 -2.370404 1.769796
C 2.211757 2.762693 -1.313059
H 2.101750 3.426692 -2.180641
H 2.254285 3.395913 -0.417363
H 3.182017 2.264020 -1.410817
C -0.200140 3.234247 1.020471
H -1.243766 2.993960 0.795143
H -0.196221 3.852373 1.927783
H 0.187815 3.852623 0.202458
58
TS-III_Et_conf_9 Eopt -845.380950
C -2.257329 1.644314 0.837691
C -1.658503 0.269669 1.203712
C -1.500866 -0.257334 -1.317811
C -2.315070 1.010623 -1.647690
H -1.452551 2.386242 0.753807
H -3.044511 0.780913 -2.442837
C -2.771288 -0.799115 1.364259
H -3.584100 -0.380041 1.981190
C -2.418525 -1.485654 -1.122197
H -1.784083 -2.372931 -0.990167
H -1.199217 0.382719 2.195156
H -0.893492 -0.479750 -2.208764
C -3.386651 -1.366317 0.065849

H	-3.809639	-2.358519	0.281598
H	-4.241668	-0.751864	-0.238321
C	-3.058265	1.657972	-0.466736
H	-3.303514	2.698423	-0.725636
H	-4.023317	1.165187	-0.303645
H	-2.365156	-1.633451	1.948464
H	-3.009096	-1.659443	-2.036412
H	-2.906535	1.994361	1.656698
H	-1.639970	1.761085	-2.077203
B	-0.610278	-0.190332	0.052040
C	0.912299	0.863119	0.013433
C	1.662512	-0.339150	-0.221629
C	1.891209	-0.812332	-1.633451
H	0.985368	-0.645394	-2.216819
C	2.598823	-0.930576	0.786811
H	2.761328	-1.990272	0.573185
C	1.161547	1.687092	1.299074
H	2.058412	2.301611	1.138257
H	2.193473	-0.865374	1.793485
C	0.834299	1.842370	-1.171997
H	0.524551	1.321821	-2.082765
H	0.039349	2.569496	-0.960742
H	2.638859	-0.118091	-2.050743
H	0.341743	2.411985	1.343085
O	0.070538	-1.558206	0.200145
C	0.013272	-2.351413	1.347595
H	-0.060183	-1.763863	2.275505
H	-0.850033	-3.034943	1.306748
H	0.917795	-2.973443	1.406541
C	2.360463	-2.249681	-1.814204
H	1.680162	-2.942541	-1.303265
H	2.362553	-2.498604	-2.881606
H	3.377859	-2.409473	-1.437192
C	3.932829	-0.177600	0.699884
H	3.800024	0.895444	0.885746
H	4.628316	-0.572265	1.449408
H	4.393684	-0.299323	-0.289128
C	2.129467	2.613441	-1.453664
H	2.994539	1.941496	-1.539750
H	2.044658	3.169579	-2.396180
H	2.352627	3.342360	-0.665438
C	1.297127	1.061522	2.695208
H	0.695290	0.157429	2.827491
H	2.337799	0.814346	2.936901
H	0.961659	1.786707	3.447173

46

TS-III_Me_conf_1 Eopt -688.323264

C	2.105890	1.304986	-1.007489
C	1.047033	0.187875	-1.254798
C	0.957975	-0.309747	1.291660
C	1.972275	0.839766	1.573421
H	1.902690	2.154900	-1.674352
H	2.938663	0.392262	1.855324
C	1.758941	-1.168722	-1.438238
H	2.405125	-1.131963	-2.330439
C	1.746557	-1.613550	1.048907
H	1.068789	-2.471842	0.979188
H	0.530428	0.414381	-2.198364
H	0.372965	-0.455728	2.211242
C	2.594696	-1.561852	-0.220421
H	3.074563	-2.536180	-0.391776
H	3.414160	-0.838593	-0.087717
C	2.193592	1.852415	0.432362
H	1.448100	2.646923	0.548442
H	3.170274	2.338850	0.568180
H	1.019034	-1.962600	-1.622976
H	2.399080	-1.809346	1.915552
H	3.095520	0.931104	-1.311686
H	1.645939	1.408981	2.454427
B	0.038555	0.119382	0.014887
C	-1.403308	-0.965509	-0.301909
C	-2.222228	0.010826	0.351217
C	-3.251103	0.802346	-0.377776
H	-3.522528	1.717212	0.157821
H	-3.001917	1.024068	-1.415733
C	-2.443339	-0.034052	1.825156
H	-3.264066	-0.750934	1.991597
H	-2.761778	0.941395	2.204203
C	-1.353209	-2.322927	0.400430
H	-1.044595	-2.260221	1.448389
H	-0.656981	-2.991316	-0.118402
H	-1.574767	-0.386531	2.380775
C	-1.678496	-1.147237	-1.793435
H	-1.602419	-0.214611	-2.362093
H	-0.959995	-1.850722	-2.227890
H	-2.683765	-1.565814	-1.961098
H	-4.137405	0.145038	-0.387931
H	-2.345492	-2.800540	0.365444
O	-0.690608	1.437028	0.324238
C	-0.845265	2.412115	-0.669059
H	-1.033544	1.983617	-1.664613
H	-1.690594	3.063208	-0.407753
H	0.046529	3.051464	-0.746263

46

TS-III_Me_conf_2 Eopt -688.325537

C	-1.805753	-1.478947	-1.124840
C	-1.010306	-0.165967	-1.326809
C	-0.971161	0.257720	1.243865
C	-1.725845	-1.083602	1.471879
H	-1.441719	-2.246458	-1.822623
H	-2.763528	-0.855662	1.760218
C	-1.978016	1.031615	-1.460164
H	-2.622493	0.896171	-2.343942
C	-2.023475	1.379825	1.058148
H	-1.546397	2.365888	1.039214
H	-0.442734	-0.224978	-2.268757
H	-0.404459	0.495682	2.158634
C	-2.852325	1.210203	-0.216900
H	-3.518401	2.075793	-0.344391
H	-3.511690	0.334673	-0.109199
C	-1.743119	-2.076193	0.293601
H	-0.843423	-2.696869	0.358858
H	-2.583707	-2.772201	0.428983
H	-1.396151	1.950712	-1.631518
H	-2.697032	1.385783	1.930504
H	-2.858140	-1.314695	-1.403273
H	-1.296192	-1.607796	2.336800
B	-0.040170	0.193508	-0.082955
C	1.360052	-1.017280	0.083145
C	2.223029	0.103175	-0.098506
C	2.865911	0.751549	1.081144
H	3.294717	1.726131	0.833687
H	2.187669	0.841950	1.934352
C	2.873365	0.372787	-1.413370
H	3.732471	-0.314058	-1.490681
H	3.243508	1.400704	-1.470814
C	1.408870	-2.060693	-1.033473
H	1.168222	-1.654872	-2.020559
H	0.705262	-2.874651	-0.832636
H	2.204118	0.173568	-2.253679
C	1.439566	-1.681872	1.458326
H	1.208087	-1.004584	2.286161
H	0.743799	-2.524386	1.518664
H	2.448620	-2.089014	1.630508
H	3.688009	0.084428	1.385441
H	2.411542	-2.513005	-1.092394
O	0.734764	1.468240	-0.428115
C	0.755403	2.661752	0.295745
H	0.647710	2.511965	1.380643
H	1.712238	3.172012	0.111248
H	-0.044351	3.339880	-0.040497

54

TS-II_Et_conf_1 Eopt -1028.027935

C	-3.626830	1.041566	0.516541
C	-2.615221	0.008219	1.091673
C	-1.850077	-0.566905	-1.318112
C	-2.882989	0.432458	-1.909486
H	-4.535086	1.032977	1.138432
H	-3.317336	-0.007364	-2.820282
C	-3.197393	-1.413211	1.231305

H -4.091432 -1.381615 1.873116
C -2.448381 -1.982227 -1.155346
H -1.639033 -2.682608 -0.903028
H -2.353043 0.356256 2.099370
H -1.022373 -0.634072 -2.039726
C -3.544568 -2.109170 -0.089825
H -3.724381 -3.175390 0.107335
H -4.492382 -1.720487 -0.477255
C -4.025732 0.843523 -0.958889
H -4.465353 1.781285 -1.326959
H -4.831208 0.104740 -1.018527
H -2.461190 -2.030800 1.770688
H -2.845561 -2.317222 -2.125916
H -3.193310 2.045290 0.638984
H -2.343713 1.329440 -2.240582
B -1.408042 0.056867 0.072543
C -0.009577 0.845226 0.339221
C 1.116167 -0.267750 0.554158
C 1.929592 -0.180916 1.862093
H 2.387344 0.806929 1.945696
C 0.547427 -1.688795 0.376296
H -0.386663 -1.738343 0.964363
C -0.184896 1.751106 1.590033
H -1.120971 2.299782 1.431752
H 0.260804 -1.824141 -0.669050
C 0.223410 1.779104 -0.881344
H 1.250504 2.158962 -0.863831
H 0.139774 1.193970 -1.805525
I 2.743471 -0.168477 -1.010157
H -0.383810 1.133585 2.466786
C -0.705460 2.993721 -0.955657
H -0.427853 3.753931 -0.215248
H -0.629644 3.457496 -1.947055
H -1.760061 2.743356 -0.788447
C 0.924955 2.770758 1.906327
H 0.502989 3.770746 2.065692
H 1.469739 2.507187 2.821195
H 1.664542 2.855006 1.099955
C 1.417856 -2.861606 0.812943
H 2.390025 -2.853170 0.305704
H 1.591458 -2.864406 1.895301
H 0.914262 -3.801197 0.555108
H 2.763592 -0.881053 1.760342
C 1.164866 -0.539407 3.153509
H 1.003787 0.341762 3.784477
H 0.182414 -0.988055 2.956495
H 1.740496 -1.261971 3.743598
54
TS-II_Et_conf_10 Eopt -1028.023685
C -3.703436 0.977546 0.637720
C -2.644949 0.000916 1.225716
C -1.923964 -0.657051 -1.175888
C -3.012028 0.268833 -1.786881
H -3.303749 1.999399 0.714998
H -3.469184 -0.246025 -2.645756
C -3.186940 -1.427772 1.438995
H -4.067278 -1.389313 2.099158
C -2.468826 -2.082869 -0.930289
H -1.635092 -2.744658 -0.654185
H -2.365754 0.396340 2.211445
H -1.113785 -0.731406 -1.916900
C -3.544846 -2.187076 0.156663
H -3.699424 -3.247170 0.402229
H -4.506350 -1.833569 -0.231177
C -4.131806 0.710671 -0.819858
H -4.593327 1.625887 -1.216757
H -4.929549 -0.038735 -0.825661
H -2.424262 -2.004585 1.986319
H -2.868324 -2.479710 -1.876211
H -4.597040 0.961066 1.280335
H -2.516130 1.154568 -2.203133
B -1.462647 0.041344 0.174479
C -0.082100 0.886922 0.369014
C 1.103802 -0.181563 0.603213
C 1.894058 0.040925 1.911543
H 1.252297 -0.385366 2.699815
C 0.514279 -1.605261 0.567859
H -0.374908 -1.576698 1.225808
C -0.246849 1.828184 1.597298
H -1.250789 2.264241 1.526288
H 0.159803 -1.805438 -0.445004
C 0.077005 1.777835 -0.891299
H 1.078505 2.223989 -0.907549
H 0.016374 1.148938 -1.787412
I 2.582886 -0.184323 -1.084136
H -0.272809 1.224595 2.511114
C -0.940090 2.917597 -1.008869
H -0.714614 3.735748 -0.316723
H -0.916343 3.331855 -2.024625
H -1.969428 2.599036 -0.810077
C 0.762883 2.984621 1.752447
H 0.308492 3.952254 1.511933
H 1.128485 3.053200 2.784783
H 1.641124 2.871571 1.103969
C 1.352265 -2.791312 1.027276
H 1.629389 -2.716805 2.085021
H 0.759142 -3.706000 0.904261
H 2.264193 -2.902756 0.428679
H 1.967069 1.104560 2.127051
C 3.309324 -0.518644 2.069645
H 4.030867 0.104778 1.529459
H 3.577868 -0.491659 3.134071
H 3.428131 -1.544767 1.715443
54
TS-II_Et_conf_11 Eopt -1028.024507
C -2.448796 -1.669220 -1.682699
C -2.152447 -0.162456 -1.469216
C -2.374097 -0.547973 1.092798
C -2.730263 -2.030835 0.816945
H -1.506926 -2.234775 -1.749308
H -3.446177 -2.368686 1.581526
C -3.499315 0.635578 -1.450654
H -4.318184 -0.028070 -1.761768
C -3.680066 0.314524 1.105928
H -3.705496 0.915423 2.024462
H -1.548706 0.184496 -2.316597
H -1.914897 -0.498770 2.089471
C -3.839325 1.262316 -0.092314
H -3.181552 2.131361 0.062340
H -4.862960 1.660661 -0.112979
C -3.320415 -2.258999 -0.573573
H -3.459789 -3.335433 -0.743429
H -4.324796 -1.813073 -0.621215
H -3.460996 1.433031 -2.203917
H -4.550176 -0.357129 1.156096
H -2.950491 -1.796562 -2.653813
H -1.838918 -2.663351 0.931013
B -1.437478 0.027414 -0.060891
C -0.051585 0.848130 0.208841
C 1.112950 -0.179377 0.595943
C 1.868012 0.106916 1.912744
H 2.100622 1.172569 1.966837
C 0.598523 -1.620675 0.584912
H -0.134706 -1.651936 1.399994
C -0.422975 1.829120 1.367983
H -1.382998 2.277150 1.081070
H 0.044089 -1.802102 -0.347167
C 0.287068 1.709570 -1.034527
H 1.271596 2.172172 -0.903186
H 0.381005 1.050190 -1.904574
I 2.793998 -0.177780 -0.929204
H -0.652410 1.267320 2.273938
C -0.703913 2.821541 -1.368522
H -0.417509 3.289130 -2.319157
H -1.728356 2.453188 -1.482662
H -0.711475 3.610821 -0.606096
C 0.543180 2.982503 1.702646
H 0.005333 3.937125 1.752924
H 1.024501 2.835793 2.676892
H 1.340804 3.098443 0.958828

C 1.586142 -2.763317 0.798581
H 2.174722 -2.961429 -0.102986
H 2.285090 -2.559383 1.618504
H 1.034456 -3.678576 1.048374
H 2.836100 -0.402828 1.877915
C 1.172669 -0.369929 3.195269
H 1.677891 0.072186 4.062713
H 0.112289 -0.107560 3.256831
H 1.242451 -1.460609 3.294868
54
TS-II_Et_conf_12 Eopt -1028.022085
C -2.452648 -1.559441 -1.587531
C -2.305226 -0.038841 -1.337237
C -2.353724 -0.456368 1.215610
C -2.759061 -1.932492 0.968368
H -1.455178 -1.997699 -1.744566
H -3.572032 -2.199628 1.661715
C -3.686342 0.666060 -1.233063
H -4.306714 0.359998 -2.090116
C -3.591154 0.472361 1.323132
H -3.259431 1.504504 1.515101
H -1.790365 0.387132 -2.209105
H -1.831604 -0.418519 2.182593
C -4.472288 0.451260 0.071841
H -5.232461 1.240468 0.155463
H -5.029591 -0.490973 0.040032
C -3.186566 -2.308449 -0.466452
H -3.036725 -3.388239 -0.604087
H -4.262797 -2.148010 -0.589724
H -3.529680 1.747059 -1.350304
H -4.188830 0.176639 2.198524
H -2.994556 -1.713789 -2.532867
H -1.908525 -2.566597 1.252099
B -1.496343 0.137660 0.017041
C -0.086107 0.934907 0.200112
C 1.066493 -0.112946 0.600950
C 1.788113 0.279351 1.898020
H 0.997425 0.479940 2.634027
C 0.495741 -1.542079 0.665364
H -0.021328 -1.587232 1.635372
C -0.429115 1.971510 1.319852
H -1.365826 2.443576 0.994696
H -0.281966 -1.644244 -0.101940
C 0.239838 1.734451 -1.085470
H 1.247085 2.159680 -1.009703
H 0.273447 1.038988 -1.932470
I 2.728783 -0.198603 -0.928953
H -0.693108 1.452934 2.248409
C -0.716827 2.877799 -1.416931
H -0.518356 3.241078 -2.433202
H -1.768583 2.576992 -1.373628
H -0.587601 3.727253 -0.734378
C 0.569883 3.100703 1.623438
H 1.256199 3.291393 0.788965
H 0.033489 4.036336 1.826042
H 1.179403 2.882288 2.506291
C 1.372912 -2.784906 0.514934
H 0.796459 -3.659878 0.844034
H 1.646393 -2.942214 -0.533884
H 2.295321 -2.748491 1.098067
H 2.309659 1.226619 1.730380
C 2.749482 -0.713483 2.542008
H 2.229374 -1.610068 2.900086
H 3.553292 -1.020114 1.861949
H 3.213469 -0.235916 3.413761
54
TS-II_Et_conf_13 Eopt -1028.022585
C -2.535305 -1.724849 -1.542704
C -2.249122 -0.211769 -1.364414
C -2.386960 -0.549279 1.211674
C -2.734659 -2.039860 0.975822
H -1.590384 -2.282209 -1.637641
H -3.427431 -2.368946 1.765161
C -3.605765 0.568943 -1.319697
H -4.423628 -0.111833 -1.593743
C -3.700917 0.300613 1.250173
H -3.700352 0.924427 2.153747
H -1.674854 0.127078 -2.235500
H -1.898336 -0.477637 2.192350
C -3.915568 1.217511 0.035801
H -3.271843 2.102878 0.150155
H -4.947166 1.595200 0.038702
C -3.360756 -2.304424 -0.392883
H -3.491249 -3.385723 -0.536904
H -4.372104 -1.872523 -0.417948
H -3.601751 1.352295 -2.088542
H -4.560734 -0.378894 1.348878
H -3.072238 -1.873531 -2.491642
H -1.832832 -2.658680 1.082855
B -1.495577 0.021152 0.019248
C -0.123193 0.881954 0.233991
C 1.065017 -0.140005 0.592526
C 1.770331 0.209986 1.910736
H 0.974356 0.360009 2.652467
C 0.526919 -1.581961 0.584524
H -0.002499 -1.679806 1.544432
C -0.491542 1.864348 1.392152
H -1.461108 2.295170 1.111033
H -0.235495 -1.664447 -0.202356
C 0.169780 1.726961 -1.029404
H 1.146410 2.213976 -0.925449
H 0.263073 1.053401 -1.888976
I 2.739332 -0.105000 -0.924233
H -0.702055 1.304309 2.309409
C -0.854882 2.807222 -1.366186
H -0.879237 3.605221 -0.613110
H -0.592051 3.270928 -2.325545
H -1.869213 2.407676 -1.465191
C 0.449703 3.035809 1.710856
H 1.153742 2.794518 2.513449
H 1.037022 3.353286 0.840485
H -0.132787 3.903943 2.045094
C 1.430766 -2.798701 0.389244
H 1.725415 -2.897532 -0.660923
H 2.341732 -2.772237 0.990616
H 0.866885 -3.700054 0.664341
H 2.271178 1.175071 1.787132
C 2.751993 -0.788787 2.514134
H 2.250503 -1.710680 2.832466
H 3.563501 -1.048706 1.823889
H 3.203778 -0.339015 3.406825
54
TS-II_Et_conf_14 Eopt -1028.026371
C -2.787511 -1.754971 -1.392670
C -2.177724 -0.333999 -1.361673
C -2.393492 -0.310034 1.221752
C -2.857422 -1.781140 1.192880
H -1.983430 -2.473238 -1.603227
H -3.565317 -1.944233 2.020117
C -3.279212 0.757495 -1.473034
H -3.863044 0.570078 -2.386636
C -3.614568 0.651108 1.126689
H -3.313120 1.628904 1.519394
H -1.536592 -0.229450 -2.247778
H -1.928609 -0.125808 2.200458
C -4.244950 0.826049 -0.274293
H -4.768794 1.792086 -0.303197
H -5.025621 0.071307 -0.414676
C -3.528598 -2.201338 -0.120695
H -3.628397 -3.295793 -0.136524
H -4.554345 -1.815724 -0.133894
H -2.800160 1.733740 -1.626734
H -4.390532 0.284313 1.816217
H -3.475992 -1.825811 -2.249082
H -2.001506 -2.434716 1.397038
B -1.433000 0.016078 -0.004518
C -0.046536 0.876104 0.114589
C 1.152300 0.006986 0.762548
C 1.918137 0.756631 1.868512
H 1.163987 1.058105 2.608381

C 0.722143 -1.372379 1.279929
H 1.549878 -1.809990 1.845771
C -0.394009 2.079758 1.056229
H -0.773311 1.699965 2.013739
H -0.080322 -1.183544 2.010630
C 0.289151 1.479103 -1.272186
H 0.564314 0.670611 -1.956624
H -0.636559 1.900936 -1.686653
I 2.772686 -0.505174 -0.752688
H 0.514379 2.643844 1.289032
C 1.356539 2.577053 -1.297101
H 1.699289 2.746882 -2.326202
H 0.967558 3.530201 -0.919447
H 2.238067 2.321954 -0.699708
C -1.396631 3.096469 0.500225
H -2.302194 2.642321 0.092392
H -1.708309 3.774269 1.305155
H -0.959504 3.712026 -0.293171
C 0.276396 -2.399598 0.247187
H -0.390999 -1.978069 -0.513024
H 1.140178 -2.806152 -0.287236
H -0.240377 -3.236777 0.728547
H 2.329796 1.683327 1.452986
C 3.016260 0.020469 2.632696
H 3.531725 0.738254 3.282720
H 2.613829 -0.767874 3.279327
H 3.765038 -0.424693 1.967481
54
TS-II_Et_conf_15 Eopt -1028.017394
C -3.597919 0.250304 1.414955
C -2.321674 -0.602560 1.184629
C -2.394937 0.017613 -1.333613
C -3.655302 0.883523 -1.020760
H -3.318801 1.263725 1.740778
H -4.280764 0.924747 -1.924864
C -2.726888 -2.068129 0.840310
H -3.772761 -2.223458 1.142918
C -2.850283 -1.428135 -1.690119
H -2.361109 -1.734814 -2.623496
H -1.769289 -0.606031 2.132649
H -1.899678 0.455514 -2.208547
C -2.552185 -2.499443 -0.628751
H -1.520444 -2.847151 -0.776451
H -3.179794 -3.380574 -0.819226
C -4.460911 0.348624 0.160464
H -5.321463 1.005259 0.347694
H -4.876361 -0.640571 -0.082317
H -2.134364 -2.754621 1.460814
H -3.927920 -1.417643 -1.909755
H -4.175105 -0.195607 2.238957
H -3.369752 1.920194 -0.790242
B -1.515512 0.074796 -0.012777
C -0.126932 0.908344 0.178675
C 1.067083 -0.083234 0.612004
C 1.790277 0.397656 1.876151
H 1.002703 0.590273 2.617631
C 0.552934 -1.528422 0.728304
H 0.009368 -1.560613 1.683567
C -0.550759 1.922021 1.294815
H -1.506901 2.340879 0.948609
H -0.191966 -1.674842 -0.061388
C 0.222609 1.731378 -1.088149
H 1.228164 2.152484 -0.972331
H 0.282629 1.047869 -1.943810
I 2.713370 -0.190226 -0.941568
H -0.803471 1.384046 2.215946
C -0.704277 2.888355 -1.456369
H -0.697277 3.684405 -0.701750
H -0.359683 3.332180 -2.399429
H -1.741116 2.576843 -1.606698
C 0.373561 3.106132 1.627106
H 1.067886 3.341280 0.811220
H -0.222084 4.007219 1.820386
H 0.972455 2.917661 2.523748
C 1.472433 -2.747398 0.650496
H 0.911980 -3.624907 1.000331
H 1.779929 -2.940271 -0.382596
H 2.376152 -2.661559 1.257638
H 2.259231 1.362928 1.658775
C 2.810687 -0.517047 2.544531
H 3.608586 -0.827156 1.859231
H 3.276241 0.025963 3.376245
H 2.338631 -1.413126 2.964676
54
TS-II_Et_conf_16 Eopt -1028.024405
C -3.671147 0.523361 0.947174
C -2.488657 -0.485327 0.958193
C -2.061132 -0.035908 -1.564547
C -3.206046 1.024645 -1.513773
H -3.344557 1.492029 1.345515
H -3.677268 1.072370 -2.506548
C -3.019831 -1.907491 0.602234
H -4.118149 -1.901809 0.664214
C -2.642940 -1.434426 -1.912043
H -2.087971 -1.849091 -2.763841
H -2.089017 -0.504273 1.980678
H -1.370946 0.273398 -2.357832
C -2.597206 -2.455607 -0.768330
H -1.575382 -2.849217 -0.693299
H -3.228724 -3.318167 -1.022250
C -4.263418 0.706692 -0.452635
H -5.019455 1.503561 -0.435190
H -4.796775 -0.211440 -0.739937
H -2.685150 -2.617242 1.371843
H -3.680520 -1.322629 -2.260701
H -4.448954 0.156503 1.633363
H -2.787468 2.025176 -1.326611
B -1.447639 0.067724 -0.110650
C -0.065157 0.862622 0.256811
C 1.040451 -0.240166 0.621530
C 1.700365 -0.149643 2.011095
H 2.141704 0.843619 2.148112
C 0.505016 -1.654644 0.356071
H -0.432957 -1.741454 0.919135
C -0.360619 1.782318 1.477905
H -0.874663 1.199864 2.248401
H 0.243591 -1.725754 -0.708626
C 0.312410 1.766211 -0.945825
H 0.586903 1.125004 -1.788925
H -0.599889 2.285654 -1.267243
I 2.858946 -0.156507 -0.745832
H 0.575399 2.130702 1.931299
C 1.407958 2.819131 -0.697693
H 2.083315 2.891588 -1.558279
H 0.980747 3.815447 -0.535326
H 2.028088 2.580362 0.174686
C -1.210096 3.021772 1.172669
H -1.615072 3.434002 2.105474
H -0.625038 3.812488 0.693528
H -2.058549 2.804081 0.515652
C 1.358735 -2.849047 0.764409
H 0.839739 -3.774597 0.485403
H 2.336911 -2.849868 0.271516
H 1.516029 -2.874794 1.849941
H 2.542698 -0.847477 2.005404
C 0.799124 -0.507578 3.207691
H 0.400624 0.381674 3.705192
H -0.052239 -1.138168 2.921429
H 1.380182 -1.066800 3.950542
54
TS-II_Et_conf_17 Eopt -1028.024083
C -3.625707 1.057340 0.544445
C -2.655547 -0.051270 1.061292
C -1.887270 -0.411974 -1.395284
C -2.909361 0.657856 -1.874067
H -3.148661 2.045471 0.623486
H -3.289718 0.358448 -2.862029
C -3.370678 -1.431418 1.040814
H -4.451092 -1.276675 0.902093
C -2.567213 -1.813408 -1.395157

H -1.933093 -2.518207 -1.950050
H -2.399206 0.205261 2.095993
H -1.061659 -0.417085 -2.118967
C -2.860446 -2.422273 -0.015424
H -1.947905 -2.901973 0.362369
H -3.589213 -3.236518 -0.129603
C -4.078483 0.820706 -0.899045
H -4.722140 1.649448 -1.224385
H -4.704596 -0.083166 -0.935509
H -3.264648 -1.900641 2.028071
H -3.505632 -1.749689 -1.965949
H -4.501367 1.086957 1.209573
H -2.405795 1.622254 -2.019022
B -1.443301 0.071646 0.050580
C -0.036185 0.831107 0.377190
C 1.077586 -0.311714 0.519921
C 1.830844 -0.358520 1.866875
H 2.277074 0.616244 2.070890
C 0.503130 -1.701320 0.201306
H -0.407899 -1.787027 0.813507
C -0.196024 1.653096 1.686547
H -1.123648 2.226777 1.571841
H 0.181982 -1.736428 -0.843848
C 0.227442 1.843965 -0.773801
H 1.267751 2.180987 -0.730259
H 0.123720 1.333066 -1.739123
I 2.766765 -0.081628 -0.959310
H -0.398258 0.981783 2.521838
C -0.641813 3.104714 -0.756216
H -0.559389 3.623491 -1.719525
H -1.703740 2.901716 -0.581104
H -0.310573 3.803258 0.021517
C 0.936424 2.629720 2.061327
H 1.420548 2.343895 3.003393
H 1.721408 2.679712 1.295878
H 0.551555 3.647512 2.198476
C 1.361487 -2.922911 0.507215
H 2.317502 -2.890782 -0.028532
H 1.566710 -3.022534 1.579260
H 0.828548 -3.826745 0.186935
H 2.673247 -1.043617 1.735963
C 1.005266 -0.844793 3.078307
H 1.495587 -1.700702 3.556189
H 0.909870 -0.058705 3.836272
H -0.010014 -1.161143 2.807412
54
TS-II_Et_conf_18 Eopt -1028.020129
C -3.727500 0.962328 0.706283
C -2.673926 -0.071325 1.211156
C -2.009033 -0.478142 -1.267514
C -3.123783 0.498816 -1.734783
H -3.301995 1.977068 0.721174
H -3.542187 0.121193 -2.679664
C -3.313926 -1.487487 1.275741
H -4.406856 -1.392128 1.195132
C -2.601924 -1.918604 -1.185168
H -1.945878 -2.606579 -1.735879
H -2.380616 0.234224 2.222513
H -1.222155 -0.464655 -2.033386
C -2.812379 -2.490731 0.225598
H -1.866984 -2.924420 0.575610
H -3.513963 -3.334247 0.168607
C -4.243169 0.640494 -0.699504
H -4.951242 1.416879 -1.020135
H -4.816867 -0.297640 -0.663226
H -3.128984 -1.915323 2.270000
H -3.560429 -1.932146 -1.724620
H -4.568149 0.974519 1.415790
H -2.695285 1.481786 -1.964458
B -1.511434 0.064652 0.143389
C -0.110882 0.871666 0.396726
C 1.054757 -0.238980 0.559608
C 1.766412 -0.213766 1.933374
H 1.079397 -0.747271 2.610525
C 0.447688 -1.633659 0.333173
H -0.411479 -1.661993 1.024997
C -0.244820 1.734595 1.680756
H -1.223572 2.230535 1.638243
H 0.053913 -1.695856 -0.685360
C 0.082818 1.844711 -0.797386
H 1.094258 2.265785 -0.766184
H 0.023283 1.281509 -1.736196
I 2.628106 -0.057282 -1.028535
H -0.307000 1.077011 2.554564
C -0.897707 3.022715 -0.848512
H -0.618454 3.812809 -0.143862
H -0.895111 3.465515 -1.852404
H -1.929765 2.740686 -0.619186
C 0.832552 2.813426 1.902842
H 1.771545 2.586846 1.381418
H 0.502403 3.796485 1.550716
H 1.066079 2.917899 2.970229
C 1.264017 -2.888731 0.613218
H 0.655994 -3.764824 0.354757
H 2.176826 -2.926311 0.006769
H 1.536967 -2.977126 1.670701
H 1.827366 0.802303 2.315149
C 3.170406 -0.803063 2.085259
H 3.377058 -0.942203 3.154694
H 3.310427 -1.763400 1.583839
H 3.921410 -0.109853 1.689364
54
TS-II_Et_conf_19 Eopt -1028.020112
C -3.661492 0.682348 1.204266
C -2.427070 -0.278153 1.283779
C -2.331612 -0.366254 -1.312723
C -3.621898 0.518669 -1.380002
H -3.572396 1.447526 1.986674
H -4.495341 -0.129590 -1.533487
C -2.911891 -1.749611 1.292620
H -3.605266 -1.885799 2.136333
C -2.748795 -1.857701 -1.239589
H -1.858511 -2.504519 -1.244581
H -1.907140 -0.097855 2.234542
H -1.755644 -0.222670 -2.235336
C -3.597121 -2.168713 -0.006373
H -3.825334 -3.242849 0.026725
H -4.566623 -1.655768 -0.089660
C -3.849930 1.398149 -0.143709
H -3.149447 2.245291 -0.191603
H -4.854746 1.840171 -0.184834
H -2.067105 -2.427555 1.479813
H -3.314159 -2.109839 -2.149399
H -4.569786 0.108394 1.440834
H -3.575648 1.166537 -2.264664
B -1.524574 0.021950 0.005336
C -0.085508 0.806571 0.065564
C 1.025485 -0.221833 0.603027
C 1.607904 0.080743 1.997747
H 1.879290 -0.875631 2.458398
C 0.420372 -1.635757 0.628123
H -0.287520 -1.595766 1.466024
C -0.391689 2.005828 1.013370
H -1.328076 2.438071 0.639241
H -0.164351 -1.792418 -0.290115
C 0.250454 1.370136 -1.335502
H 1.255677 1.805453 -1.321011
H 0.296533 0.537402 -2.048630
I 2.809562 -0.414676 -0.773522
H -0.645769 1.641651 2.013768
C -0.697703 2.439121 -1.873041
H -0.430478 2.676085 -2.910714
H -1.745034 2.122349 -1.869940
H -0.627755 3.370839 -1.296895
C 0.619448 3.156655 1.128921
H 1.297553 3.213018 0.268330
H 0.090302 4.116060 1.190793
H 1.231812 3.071361 2.031270
C 1.303296 -2.862120 0.835944
H 2.021440 -2.729288 1.653637

H 0.666675 -3.719910 1.088678
H 1.863860 -3.114990 -0.069376
H 0.769145 0.458021 2.596702
C 2.814293 1.012452 2.138818
H 2.827259 1.463011 3.139506
H 3.746332 0.450801 2.012779
H 2.817063 1.817289 1.398765
54
TS-II_Et_conf_2 Eopt -1028.028302
C -2.274045 -1.943167 -1.448691
C -1.771848 -0.478681 -1.472149
C -2.706866 -0.164738 0.936802
C -3.131101 -1.655145 0.930413
H -1.450206 -2.620425 -1.183624
H -4.024078 -1.771572 1.562934
C -2.894719 0.463901 -2.024260
H -3.680300 -0.151346 -2.488014
C -3.873112 0.702974 0.351132
H -4.165156 1.468741 1.080739
H -0.911182 -0.432438 -2.152330
H -2.530556 0.133789 1.978451
C -3.529651 1.391266 -0.975857
H -2.829913 2.215046 -0.765585
H -4.430151 1.859507 -1.396241
C -3.430958 -2.173213 -0.477163
H -3.669232 -3.244855 -0.434867
H -4.334277 -1.678516 -0.862886
H -2.478849 1.086336 -2.827275
H -4.760934 0.070650 0.213727
H -2.586850 -2.225421 -2.465351
H -2.348472 -2.277133 1.391944
B -1.438972 0.038924 -0.005308
C -0.069609 0.828603 0.382916
C 1.077142 -0.262173 0.631477
C 1.808451 -0.193405 1.987480
H 2.202941 0.815945 2.140936
C 0.553255 -1.684138 0.367488
H -0.408933 -1.774537 0.897464
C -0.273526 1.689098 1.659013
H -0.553821 1.043759 2.494917
H 0.329374 -1.777176 -0.697886
C 0.194817 1.799558 -0.802783
H 0.486346 1.215887 -1.680594
H -0.757749 2.273693 -1.079327
I 2.795683 -0.110343 -0.841332
H 0.675446 2.163540 1.938733
C 1.213612 2.914841 -0.545485
H 0.773151 3.742842 0.022513
H 2.086358 2.560489 0.013513
H 1.576351 3.322423 -1.497936
C -1.335692 2.785208 1.566540
H -1.349632 3.353430 2.505679
H -1.149729 3.498652 0.756319
H -2.339560 2.374955 1.426511
C 1.421458 -2.858048 0.804919
H 0.943960 -3.795894 0.495675
H 2.415360 -2.820668 0.344006
H 1.545221 -2.892496 1.893416
H 2.683868 -0.844051 1.912408
C 0.980352 -0.650240 3.209244
H 0.813213 0.174256 3.910224
H -0.004777 -1.046766 2.930381
H 1.508679 -1.442084 3.752366
54
TS-II_Et_conf_20 Eopt -1028.023159
C -3.703312 0.764607 1.001344
C -2.520365 -0.229753 1.166808
C -2.201089 -0.305806 -1.409912
C -3.341656 0.752096 -1.529766
H -3.366355 1.791389 1.187121
H -3.856603 0.598258 -2.489596
C -3.078485 -1.686704 1.133290
H -4.170651 -1.646199 1.256968
C -2.803287 -1.739601 -1.445140
H -2.273043 -2.328401 -2.205202
H -2.077091 -0.047914 2.155294
H -1.544576 -0.173218 -2.277627
C -2.745422 -2.511509 -0.118906
H -1.741182 -2.938049 -0.003864
H -3.424767 -3.373377 -0.171556
C -4.351945 0.665711 -0.382254
H -5.108527 1.454142 -0.495857
H -4.894586 -0.288501 -0.455499
H -2.703900 -2.233008 2.010086
H -3.846684 -1.684195 -1.789386
H -4.453039 0.544805 1.776005
H -2.915680 1.765789 -1.572178
B -1.516308 0.070485 -0.033191
C -0.094726 0.879933 0.111714
C 0.990519 -0.171749 0.672934
C 1.588539 0.156561 2.047738
H 2.008120 -0.766459 2.460889
C 0.355198 -1.573074 0.700042
H -0.478474 -1.495764 1.412202
C -0.339123 2.047538 1.114136
H -0.789564 1.649441 2.031102
H -0.074018 -1.758634 -0.294942
C 0.262710 1.486350 -1.267682
H 0.511638 0.668956 -1.952752
H -0.649305 1.938343 -1.682427
I 2.769837 -0.474133 -0.693514
H 0.607855 2.502923 1.418118
C 1.369085 2.548448 -1.294519
H 0.984319 3.544471 -1.049314
H 2.181194 2.328763 -0.595123
H 1.813660 2.607679 -2.296542
C -1.237536 3.175248 0.590067
H -1.618184 3.770771 1.429340
H -0.697744 3.857258 -0.073821
H -2.103684 2.809062 0.031260
C 1.158673 -2.803406 1.108567
H 1.961178 -3.030128 0.400204
H 1.596078 -2.704293 2.108362
H 0.481027 -3.666843 1.135435
H 0.731546 0.403116 2.691017
C 2.665002 1.236675 2.167599
H 3.650190 0.824986 1.921286
H 2.498427 2.093626 1.507488
H 2.705911 1.608427 3.199314
54
TS-II_Et_conf_21 Eopt -1028.023458
C -3.746559 0.993298 0.260074
C -2.726654 0.010477 0.917216
C -1.778301 -0.433482 -1.459312
C -2.834453 0.546423 -2.044824
H -3.346344 2.016308 0.251533
H -3.090484 0.218965 -3.063482
C -3.339521 -1.420094 0.952032
H -4.427616 -1.341224 0.811387
C -2.380778 -1.870489 -1.440242
H -1.661963 -2.565432 -1.894933
H -2.555744 0.348820 1.947552
H -0.911078 -0.422858 -2.132386
C -2.763813 -2.429687 -0.058177
H -1.874831 -2.896012 0.384519
H -3.482114 -3.250002 -0.191339
C -4.090339 0.613963 -1.178587
H -4.792042 1.349509 -1.594980
H -4.611385 -0.354502 -1.200047
H -3.202923 -1.837719 1.958451
H -3.261314 -1.890026 -2.098438
H -4.655326 1.015840 0.879950
H -2.410742 1.557239 -2.139628
B -1.441907 0.113497 -0.002130
C -0.051398 0.868703 0.377312
C 1.067526 -0.260131 0.623196
C 1.767714 -0.220723 1.997652
H 2.143791 0.788249 2.187918
C 0.503309 -1.666052 0.373710
H -0.389977 -1.730728 1.014426

C	-0.170855	1.772157	1.638074
H	-0.381852	1.168866	2.523392
H	0.157740	-1.743216	-0.661045
C	0.214023	1.815866	-0.831409
H	0.498188	1.215688	-1.700442
H	-0.739505	2.285716	-1.108684
I	2.795221	-0.120924	-0.825671
H	0.804394	2.238848	1.817493
C	1.225991	2.943180	-0.608749
H	0.789890	3.763281	-0.024648
H	2.124530	2.602981	-0.083142
H	1.544049	3.356409	-1.574633
C	-1.219541	2.889747	1.602071
H	-0.949241	3.661791	2.333842
H	-1.301219	3.382936	0.626409
H	-2.213222	2.525262	1.878834
C	1.379195	-2.873425	0.688636
H	2.281945	-2.889597	0.067769
H	1.681941	-2.907576	1.740612
H	0.812729	-3.789216	0.477297
H	2.654270	-0.858237	1.936897
C	0.905134	-0.718760	3.178520
H	1.225426	-1.714737	3.504364
H	1.000966	-0.044080	4.037263
H	-0.162196	-0.785876	2.935948

54

TS-II_Et_conf_22 Eopt -1028.021659

C	3.861168	-0.830574	0.926068
C	2.756562	0.253251	1.021809
C	1.975184	-0.329797	-1.370549
C	2.885489	-1.584761	-1.347076
H	3.595493	-1.642822	1.615737
H	3.205387	-1.791469	-2.379477
C	3.261241	1.628437	0.511124
H	4.109488	1.946838	1.135701
C	2.753079	0.888606	-1.935961
H	2.025682	1.607588	-2.325179
H	2.527561	0.366684	2.085882
H	1.167400	-0.561343	-2.073312
C	3.703675	1.615561	-0.959272
H	3.834141	2.651908	-1.300503
H	4.701283	1.164927	-1.010542
C	4.129982	-1.427536	-0.467626
H	4.612397	-2.407621	-0.346756
H	4.857401	-0.807973	-1.004464
H	2.480204	2.387312	0.667805
H	3.334339	0.554535	-2.809452
H	4.798101	-0.409460	1.323008
H	2.301948	-2.460432	-1.035799
B	1.483343	-0.061939	0.123004
C	-0.026598	-0.293009	0.706255
C	-1.203309	0.555246	0.046806
C	-1.803602	1.599391	0.999194
H	-0.971989	2.260728	1.269410
C	-0.875317	1.216454	-1.301945
H	-0.387698	0.498360	-1.964626
C	-0.029086	-0.209450	2.257870
H	-1.004704	-0.558520	2.631384
H	-1.808476	1.496887	-1.799892
C	-0.154905	-1.821501	0.362165
H	-1.148672	-2.146579	0.680412
H	-0.125690	-1.943905	-0.728878
I	-3.021720	-0.737900	-0.476059
H	0.689742	-0.960022	2.598682
C	0.832932	-2.786499	1.031484
H	0.541983	-2.992429	2.067639
H	0.820299	-3.744055	0.496248
H	1.868679	-2.436482	1.048169
C	0.353770	1.113879	2.949149
H	-0.515268	1.640668	3.354948
H	1.033874	0.921288	3.787991
H	0.867612	1.804393	2.267466
C	-0.032926	2.484493	-1.145700
H	0.292487	2.842520	-2.128918
H	-0.607553	3.289391	-0.673694
H	0.862812	2.325104	-0.534705
H	-2.125352	1.110461	1.923328
C	-2.947627	2.474439	0.490867
H	-3.883602	1.912175	0.408596
H	-3.111642	3.290080	1.206579
H	-2.741512	2.929908	-0.484676

54

TS-II_Et_conf_3 Eopt -1028.027471

C	-3.666548	0.357555	1.047434
C	-2.443134	-0.595371	0.937520
C	-2.062895	0.088474	-1.531214
C	-3.210445	1.132029	-1.411353
H	-3.384699	1.208188	1.679762
H	-3.740478	1.187739	-2.374351
C	-2.881667	-2.001515	0.462014
H	-3.644029	-2.387052	1.156112
C	-2.547831	-1.306063	-1.979313
H	-1.659405	-1.921657	-2.190913
H	-2.033182	-0.701813	1.951754
H	-1.379016	0.461060	-2.304863
C	-3.426796	-2.052564	-0.969002
H	-3.510633	-3.103473	-1.279607
H	-4.448324	-1.657637	-0.993554
C	-4.237720	0.874848	-0.291017
H	-4.781598	1.810620	-0.099962
H	-4.993556	0.172957	-0.657142
H	-2.032352	-2.696300	0.533589
H	-3.085976	-1.214925	-2.935627
H	-4.465900	-0.164729	1.595042
H	-2.757690	2.124018	-1.266023
B	-1.422995	0.071000	-0.085555
C	-0.029473	0.832808	0.307993
C	1.071126	-0.299375	0.595792
C	1.667931	-0.328820	2.019028
H	1.940247	0.688067	2.320195
C	0.524860	-1.680417	0.215333
H	-0.298155	-1.844859	0.920109
C	-0.308226	1.714814	1.557179
H	-0.702034	1.097320	2.369974
H	0.081513	-1.632225	-0.790927
C	0.338407	1.791991	-0.855641
H	0.644156	1.194546	-1.720406
H	-0.581750	2.301693	-1.170975
I	2.923266	-0.086389	-0.702009
H	0.625411	2.154557	1.929347
C	1.390687	2.869171	-0.550422
H	1.944556	3.133471	-1.459985
H	0.928175	3.787589	-0.170472
H	2.126853	2.543765	0.192209
C	-1.294686	2.864107	1.316125
H	-2.182843	2.550797	0.757435
H	-1.640264	3.267293	2.276255
H	-0.841743	3.689735	0.758835
C	1.441331	-2.896915	0.297334
H	2.022895	-2.912673	1.227159
H	0.832887	-3.809853	0.267715
H	2.143511	-2.934326	-0.541330
H	2.607459	-0.889323	1.990953
C	0.792137	-0.992759	3.088153
H	1.236843	-0.814657	4.074846
H	-0.234863	-0.617367	3.115036
H	0.743510	-2.078925	2.938393

54

TS-II_Et_conf_4 Eopt -1028.026195

C	-3.607950	0.694886	1.143788
C	-2.449133	-0.339650	1.178354
C	-2.239636	-0.266963	-1.404329
C	-3.361076	0.809674	-1.462434
H	-4.372423	0.389130	1.874358
H	-3.976751	0.632199	-2.357411
C	-2.983496	-1.787020	1.077116
H	-3.715769	-1.953536	1.882104
C	-2.779353	-1.710284	-1.473849
H	-1.920604	-2.393106	-1.570997
H	-1.955762	-0.234253	2.156760

H	-1.606172	-0.110285	-2.287174
C	-3.619206	-2.149867	-0.269670
H	-3.760305	-3.238982	-0.312759
H	-4.625144	-1.721601	-0.338285
C	-4.285322	0.891082	-0.229898
H	-4.782527	1.871307	-0.235716
H	-5.092026	0.159780	-0.340465
H	-2.160215	-2.490478	1.268966
H	-3.368556	-1.834537	-2.395545
H	-3.222447	1.656537	1.504575
H	-2.884424	1.787916	-1.621576
B	-1.496678	0.028084	-0.039696
C	-0.073397	0.824003	0.089975
C	0.997666	-0.240574	0.637149
C	1.515012	0.019959	2.061062
H	1.895462	-0.927726	2.457988
C	0.381315	-1.652740	0.595264
H	-0.355374	-1.638807	1.408060
C	-0.310616	1.994212	1.089035
H	-0.752156	1.601907	2.013230
H	-0.172274	-1.779152	-0.348586
C	0.277381	1.421243	-1.295571
H	0.532466	0.597389	-1.971162
H	-0.640670	1.857020	-1.711560
I	2.846394	-0.414127	-0.645271
H	0.637937	2.455221	1.380269
C	1.372236	2.501197	-1.330783
H	1.985045	2.405875	-2.235384
H	0.941953	3.509627	-1.333667
H	2.053355	2.437996	-0.475467
C	-1.219592	3.112350	0.567301
H	-1.508896	3.770759	1.396138
H	-0.719834	3.732251	-0.183405
H	-2.143071	2.737089	0.113688
C	1.246683	-2.893011	0.797861
H	1.842933	-3.123574	-0.090173
H	1.929115	-2.791922	1.649772
H	0.592677	-3.751903	0.996587
H	0.621150	0.247209	2.658619
C	2.589332	1.085549	2.291186
H	2.517005	1.480351	3.312557
H	3.589694	0.655803	2.167654
H	2.516015	1.928498	1.596500

54

TS-II_Et_conf_5 Eopt -1028.017119

C	-3.866935	0.889999	0.310489
C	-2.670024	0.257375	1.073335
C	-1.895460	-0.846080	-1.135105
C	-3.041742	-0.140891	-1.905976
H	-3.633929	1.946914	0.118310
H	-3.381099	-0.808617	-2.712261
C	-3.005064	-1.137205	1.655200
H	-3.864246	-1.044022	2.336869
C	-2.375123	-2.229723	-0.614418
H	-1.498058	-2.841395	-0.370616
H	-2.450288	0.913475	1.925924
H	-1.087736	-1.029560	-1.858917
C	-3.314771	-2.215442	0.607197
H	-3.272050	-3.200715	1.091928
H	-4.352663	-2.101317	0.278296
C	-4.243395	0.239746	-1.031069
H	-4.880894	0.939642	-1.589229
H	-4.864002	-0.645600	-0.860685
H	-2.162920	-1.464335	2.284382
H	-2.879473	-2.754152	-1.440576
H	-4.746803	0.894629	0.972503
H	-2.651694	0.754858	-2.403230
B	-1.451763	0.101633	0.069794
C	-0.016128	0.880965	0.210404
C	1.127574	-0.181219	0.626260
C	1.843441	0.044000	1.969926
H	2.429781	-0.858184	2.156024
C	0.555706	-1.607934	0.593793
H	-0.381261	-1.571957	1.175946
C	-0.086064	1.962219	1.323968
H	-0.318866	1.458185	2.270173
H	0.277286	-1.834682	-0.437006
C	0.256063	1.526919	-1.174056
H	1.210787	2.067040	-1.128073
H	0.406191	0.712514	-1.891117
I	2.858144	-0.296051	-0.834450
H	0.901833	2.408779	1.450740
C	-0.816346	2.476260	-1.742045
H	-1.811119	2.325024	-1.306829
H	-0.554804	3.526011	-1.577354
H	-0.909009	2.333260	-2.825430
C	-1.061159	3.133682	1.181026
H	-2.065294	2.836781	0.868396
H	-1.155862	3.629286	2.156037
H	-0.697600	3.885884	0.474445
C	1.377151	-2.766738	1.149495
H	1.469625	-2.724874	2.240644
H	0.874943	-3.709606	0.900062
H	2.384049	-2.799761	0.716340
H	1.058381	0.042045	2.740669
C	2.780653	1.251100	2.152880
H	3.792938	0.910381	2.398588
H	2.859215	1.861804	1.246720
H	2.441579	1.902622	2.967374

54

TS-II_Et_conf_6 Eopt -1028.018395

C	-2.516772	-1.745989	-1.488276
C	-2.021278	-0.274083	-1.395870
C	-2.688885	-0.158024	1.109172
C	-3.146552	-1.648746	1.074074
H	-1.793797	-2.325081	-2.078089
H	-4.241237	-1.670632	1.175423
C	-3.206639	0.682154	-1.708632
H	-3.545829	0.497568	-2.738723
C	-3.894837	0.741451	0.707117
H	-3.635583	1.802920	0.801119
H	-1.255716	-0.114264	-2.165418
H	-2.414369	0.098327	2.141687
C	-4.360179	0.497785	-0.725451
H	-5.176966	1.190007	-0.971345
H	-4.776102	-0.516434	-0.821629
C	-2.729646	-2.490755	-0.153421
H	-1.801088	-3.020853	0.090347
H	-3.475780	-3.281794	-0.305495
H	-2.875831	1.732582	-1.671913
H	-4.716315	0.554833	1.415018
H	-3.447514	-1.764339	-2.072756
H	-2.760287	-2.154283	1.969314
B	-1.529116	0.125529	0.061642
C	-0.117063	0.892410	0.363602
C	1.019235	-0.236918	0.590582
C	1.611580	-0.246674	2.013382
H	0.818487	-0.684399	2.642845
C	0.407574	-1.599703	0.233288
H	-0.495616	-1.657934	0.862945
C	-0.238640	1.797985	1.617544
H	-0.394707	1.181449	2.509320
H	0.078456	-1.568427	-0.811275
C	0.115494	1.813535	-0.869004
H	0.452913	1.200597	-1.709604
H	-0.854690	2.214962	-1.191103
I	2.762821	-0.033422	-0.818969
H	0.714812	2.316093	1.769941
C	1.057159	3.001844	-0.668241
H	0.577162	3.795194	-0.080177
H	1.984199	2.720928	-0.156552
H	1.331531	3.430683	-1.640769
C	-1.352113	2.854879	1.575982
H	-1.025910	3.767808	2.089263
H	-1.640213	3.141773	0.556769
H	-2.252615	2.505031	2.090080
C	1.167784	-2.899503	0.465158
H	1.321663	-3.100332	1.530997
H	0.573444	-3.727331	0.058176
H	2.139670	-2.905889	-0.041993

H 1.744527 0.778063 2.363653
C 2.931883 -0.964196 2.306105
H 3.020022 -1.102380 3.391799
H 3.029855 -1.942633 1.831778
H 3.780837 -0.352796 1.980849
54
TS-II_Et_conf_7 Eopt -1028.018688
C 3.866632 -1.218947 0.642547
C 2.962921 -0.006773 0.972322
C 1.998126 -0.129799 -1.434058
C 2.950524 -1.318850 -1.711752
H 3.369011 -2.147587 0.965960
H 3.235769 -1.298731 -2.774622
C 3.683723 1.327816 0.602088
H 4.734376 1.122265 0.349504
C 2.688097 1.228327 -1.775846
H 2.035826 1.809429 -2.442375
H 2.803395 -0.018252 2.053399
H 1.145788 -0.273957 -2.103210
C 3.019392 2.092469 -0.549054
H 2.086385 2.535499 -0.163181
H 3.649324 2.940299 -0.850923
C 4.203983 -1.316269 -0.842692
H 4.795209 -2.222003 -1.036083
H 4.844186 -0.467895 -1.127953
H 3.706244 1.982689 1.483297
H 3.610634 1.036200 -2.343843
H 4.793367 -1.143850 1.231615
H 2.402943 -2.261487 -1.557012
B 1.613343 -0.087274 0.115195
C 0.148194 -0.282637 0.765148
C -1.072304 0.579403 0.202978
C -1.718038 1.474460 1.278613
H -1.026641 2.326489 1.381105
C -0.655210 1.402780 -1.026525
H 0.275265 1.906005 -0.729251
C 0.279687 -0.248609 2.309272
H -0.650689 -0.586302 2.784669
H -0.395691 0.719620 -1.836866
C 0.160878 -1.797268 0.340425
H -0.178219 -1.874495 -0.697297
H 1.207894 -2.154741 0.325045
I -2.749979 -0.729609 -0.585019
H 1.014506 -1.021715 2.561822
C -0.596159 -2.778733 1.240000
H -1.592561 -2.414090 1.507499
H -0.717276 -3.735009 0.715636
H -0.044954 -2.976296 2.166745
C 0.748263 1.077304 2.935904
H 1.186251 1.748176 2.183206
H -0.066659 1.621690 3.422952
H 1.515748 0.897791 3.698805
C -1.587137 2.472628 -1.582323
H -2.560393 2.064820 -1.880636
H -1.749736 3.290272 -0.870735
H -1.120182 2.904493 -2.476339
H -1.707279 0.953658 2.235865
C -3.144268 2.009244 1.121047
H -3.284591 2.835454 1.830675
H -3.384954 2.376495 0.122114
H -3.873935 1.231454 1.371884
54
TS-II_Et_conf_8 Eopt -1028.027436
C -3.584370 1.067322 0.828414
C -2.702628 -0.175122 1.135268
C -1.924192 -0.271019 -1.335116
C -2.906704 0.879233 -1.701639
H -3.060682 1.967664 1.176752
H -3.408171 0.615259 -2.645553
C -3.489684 -1.489989 0.926427
H -4.440955 -1.423855 1.477055
C -2.593923 -1.653804 -1.478197
H -1.845470 -2.439626 -1.306617
H -2.433271 -0.117316 2.198995
H -1.098887 -0.217386 -2.059029
C -3.778914 -1.874234 -0.532572
H -4.072624 -2.932805 -0.567583
H -4.646568 -1.317596 -0.904084
C -3.986317 1.229233 -0.651224
H -4.304674 2.268764 -0.813815
H -4.878861 0.623313 -0.837015
H -2.930598 -2.309912 1.397614
H -2.933919 -1.780400 -2.517441
H -4.498274 1.005082 1.438062
H -2.311161 1.772778 -1.924894
B -1.459327 -0.014983 0.160238
C -0.008509 0.614719 0.569984
C 1.156942 -0.487073 0.423942
C 1.901488 -0.775433 1.741413
H 1.132627 -1.075389 2.466260
C 0.690840 -1.818660 -0.192879
H 0.157132 -1.628195 -1.127939
C -0.094998 1.180911 2.014112
H -1.043162 1.727550 2.079831
H 1.566039 -2.416801 -0.465661
C 0.176149 1.832379 -0.387742
H 1.194491 2.216321 -0.274249
H 0.100196 1.491150 -1.427491
I 2.785221 0.147346 -1.014014
H -0.206834 0.356782 2.725713
C -0.759541 3.024115 -0.171758
H -0.607707 3.747717 -0.982809
H -1.820407 2.760519 -0.169368
H -0.544938 3.541758 0.770111
C 1.034909 2.129167 2.464579
H 1.881494 2.138883 1.766732
H 0.680012 3.162923 2.551264
H 1.427585 1.840358 3.447254
C -0.164013 -2.633903 0.780409
H -0.682462 -3.444541 0.256174
H 0.454422 -3.085521 1.564316
H -0.918783 -2.024102 1.290924
H 2.343473 0.144368 2.128666
C 2.975774 -1.859951 1.730852
H 2.598211 -2.832882 1.395849
H 3.825122 -1.586292 1.095138
H 3.353694 -1.990336 2.752730
54
TS-II_Et_conf_9 Eopt -1028.026663
C -3.898676 0.870710 0.400490
C -2.725559 0.092561 1.089788
C -1.957216 -0.744633 -1.251599
C -3.085586 0.108855 -1.921894
H -4.127588 1.778263 0.973106
H -3.921354 -0.552472 -2.195533
C -3.200092 -1.346858 1.414127
H -4.059672 -1.289147 2.098894
C -2.497779 -2.151909 -0.897496
H -1.677818 -2.793365 -0.544837
H -2.475249 0.588300 2.036807
H -1.142610 -0.874911 -1.975803
C -3.598601 -2.130291 0.162268
H -3.864863 -3.159750 0.438420
H -4.509172 -1.688971 -0.268684
C -3.613087 1.261427 -1.054510
H -2.865423 2.069652 -1.054536
H -4.518173 1.683865 -1.511957
H -2.415933 -1.895384 1.957866
H -2.881731 -2.623306 -1.814830
H -4.810718 0.259362 0.438865
H -2.707873 0.529839 -2.862818
B -1.510104 0.043626 0.058933
C -0.088958 0.838382 0.202126
C 1.019123 -0.220448 0.674209
C 1.705612 0.043884 2.022428
H 2.296410 -0.846961 2.248341
C 0.402376 -1.631723 0.683012
H -0.541787 -1.547038 1.247796
C -0.204718 1.984558 1.241916
H -0.398613 1.564461 2.236660

H 0.137368 -1.888847 -0.345361
C 0.154542 1.471152 -1.197186
H 0.432820 0.677370 -1.897530
H -0.804999 1.857406 -1.572351
I 2.751404 -0.458317 -0.761978
H 0.752792 2.506735 1.308606
C 1.169917 2.613161 -1.272261
H 0.775384 3.537648 -0.833831
H 2.106000 2.374712 -0.756948
H 1.416392 2.823443 -2.321189
C -1.274895 3.041644 0.967758
H -1.171284 3.856141 1.696413
H -1.185842 3.484390 -0.031202
H -2.289142 2.648070 1.069543
C 1.176720 -2.790501 1.302124
H 0.646192 -3.727055 1.090674
H 2.187314 -2.876490 0.885236
H 1.256347 -2.698226 2.391191
H 0.901467 0.056615 2.774298
C 2.617365 1.271120 2.184070
H 2.214044 1.981214 2.915298
H 3.609418 0.961937 2.532919
H 2.761079 1.808636 1.240410
42
TS-II_Me_conf_1 Eopt -870.976351
C -2.343009 -2.024629 0.523944
C -1.639997 -1.148084 -0.534576
C -2.567999 0.977392 0.630106
C -3.261447 0.086301 1.679769
H -1.598424 -2.322552 1.276772
H -4.210339 0.549873 1.992016
C -2.538873 -0.862909 -1.772817
H -2.885647 -1.820313 -2.190892
C -3.429761 1.270857 -0.633060
H -2.891299 2.007596 -1.251527
H -0.757396 -1.696243 -0.897763
H -2.358691 1.947225 1.101345
C -3.748899 0.048104 -1.506851
H -4.139344 0.401227 -2.471576
H -4.562103 -0.529037 -1.056315
C -3.523812 -1.364274 1.250983
H -3.759562 -1.957088 2.146048
H -4.422891 -1.412277 0.628005
H -1.916933 -0.398455 -2.551825
H -4.366462 1.760952 -0.326325
H -2.686087 -2.959282 0.053534
H -2.622011 0.073815 2.576921
B -1.287588 0.288242 0.024439
C 0.106131 1.055472 -0.250036
C 1.192439 0.489730 0.759646
C 1.856830 1.553841 1.628698
H 2.620794 1.094324 2.263916
H 2.324476 2.349367 1.040338
C 0.632608 -0.601717 1.670015
H -0.264327 -0.208765 2.182107
H 1.358298 -0.876380 2.444359
C -0.074549 2.575085 -0.065568
H -0.898205 2.918870 -0.703697
H 0.823433 3.128369 -0.370794
H 0.356953 -1.505427 1.121208
C 0.428287 0.855539 -1.743376
H 1.364802 1.363081 -2.010161
H 0.517292 -0.199790 -2.022489
H -0.371457 1.303042 -2.349995
I 2.914663 -0.505259 -0.260694
H 1.099711 2.002101 2.287560
H -0.321465 2.859061 0.962558
42
TS-II_Me_conf_2 Eopt -870.974936
C 2.525322 1.987208 -0.626645
C 1.715087 0.736820 -1.072821
C 2.602930 -0.549599 1.008946
C 3.439722 0.695133 1.408599
H 1.948649 2.892680 -0.859503
H 4.481894 0.567492 1.078952
C 2.591341 -0.167335 -1.991604
H 2.921312 0.426629 -2.856757
C 3.407348 -1.466460 0.030761
H 2.811818 -2.357207 -0.227435
H 0.847400 1.055942 -1.665321
H 2.381358 -1.147520 1.901290
C 3.803224 -0.744430 -1.258543
H 4.339572 -1.438542 -1.919757
H 4.510758 0.063953 -1.024428
C 2.890588 2.018421 0.862779
H 1.991674 2.283313 1.436627
H 3.614369 2.824671 1.046780
H 1.987383 -0.993386 -2.392920
H 4.303897 -1.833288 0.551837
H 3.445784 2.057954 -1.226304
H 3.480125 0.763755 2.503881
B 1.334900 -0.220049 0.130577
C -0.073368 -1.013008 0.238993
C -1.154774 -0.035081 0.869926
C -1.756849 -0.530102 2.182345
H -2.505955 0.182671 2.542590
H -2.227399 -1.513459 2.089957
C -0.620563 1.374635 1.102570
H 0.214727 1.295931 1.817511
H -1.382717 2.014646 1.560857
C 0.083303 -2.284910 1.095253
H 0.338929 -2.077086 2.139180
H 0.888258 -2.903864 0.680059
H -0.263828 1.859625 0.188474
C -0.404227 -1.515286 -1.179917
H -0.495618 -0.704084 -1.910135
H 0.389267 -2.194845 -1.518686
H -1.342420 -2.084815 -1.178630
I -2.940675 0.273719 -0.442508
H -0.961575 -0.590578 2.939040
H -0.830893 -2.893462 1.079024
42
TS-II_Me_conf_3 Eopt -870.974975
C -2.387735 -2.118437 -0.481041
C -1.760791 -0.838508 -1.072586
C -2.465006 0.434152 1.094561
C -3.063593 -0.885037 1.627336
H -1.608980 -2.710869 0.024038
H -3.916280 -0.655960 2.284855
C -2.827082 -0.024932 -1.886565
H -3.715855 -0.655303 -2.037468
C -3.533803 1.234108 0.272012
H -3.595819 2.259693 0.658233
H -0.953334 -1.137511 -1.752458
H -2.157796 1.042089 1.955016
C -3.247716 1.306492 -1.238319
H -2.442780 2.040598 -1.401155
H -4.126436 1.707951 -1.760909
C -3.511652 -1.827702 0.511869
H -3.877930 -2.768691 0.944861
H -4.367046 -1.385228 -0.021040
H -2.430291 0.191819 -2.886758
H -4.523129 0.782749 0.437318
H -2.769402 -2.745961 -1.300986
H -2.319335 -1.401792 2.253546
B -1.298750 0.168036 0.057433
C 0.068427 1.028882 -0.003105
C 1.158703 0.253746 0.851877
C 1.779064 1.071068 1.979651
H 2.537348 0.477940 2.501670
H 2.243759 1.995914 1.623199
C 0.615524 -1.051045 1.429096
H 0.247482 -1.729621 0.650455
H -0.210258 -0.807698 2.116354
C -0.198764 2.433092 0.578021
H 0.674722 3.088844 0.464401
H -0.482872 2.418138 1.635000
H 1.380111 -1.580280 2.008652
C 0.436753 1.250775 -1.480044
H 0.620020 0.314918 -2.016426

H -0.385761 1.774598 -1.986829
H 1.333022 1.878990 -1.570403
I 2.924439 -0.397430 -0.357337
H 0.996183 1.328591 2.706907
H -1.024819 2.899773 0.025027

43
B_b_Me_conf_1 Eopt -946.194392
C -1.702416 -1.709257 -1.146740
C -1.457392 -0.196111 -1.316343
C -2.456001 -0.102061 1.514087
C -2.456060 -1.616668 1.300046
H -0.752472 -2.205555 -0.902435
H -3.214379 -2.027915 1.981999
C -2.777977 0.577309 -1.605343
H -3.541751 -0.126823 -1.969662
C -3.649034 0.639731 0.868810
H -4.026891 1.378520 1.587296
H -0.826727 -0.107859 -2.208869
H -2.511805 0.054587 2.597963
C -3.346814 1.389361 -0.433404
H -2.648535 2.208102 -0.200749
H -4.274787 1.877333 -0.761850
C -2.748601 -2.107561 -0.112794
H -2.821905 -3.203972 -0.092405
H -3.739320 -1.748293 -0.427957
H -2.602247 1.283811 -2.428320
H -4.466391 -0.077132 0.709432
H -2.016702 -2.108326 -2.123739
H -1.482498 -2.002128 1.641478
B -0.709140 0.535424 -0.111747
C 0.567178 1.516740 -0.308595
C 1.829217 0.976268 0.422252
C 3.136166 1.608199 -0.036932
H 3.974202 1.215122 0.550153
H 3.343281 1.435798 -1.096969
C 1.739671 1.019490 1.941335
H 0.805435 0.590117 2.312804
H 1.793100 2.070371 2.261493
O -1.216606 0.507345 1.148133
C 0.188773 2.884384 0.306361
H 1.040222 3.579358 0.296415
H -0.177450 2.788776 1.333809
H 2.587887 0.492255 2.390877
C 0.850547 1.752339 -1.797899
H 1.257113 0.857415 -2.287829
H -0.076279 2.028796 -2.317629
H 1.561689 2.576299 -1.943311
I 2.024918 -1.196125 -0.093193
H 3.080251 2.693893 0.135949
H -0.608709 3.343254 -0.294091
43
B_b_Me_conf_2 Eopt -946.192619
C -3.350043 1.386272 0.032453
C -2.168299 0.963027 0.933041
C -1.932336 -1.211491 -1.131349
C -3.133670 -0.476405 -1.725119
H -3.009019 2.157745 -0.677623
H -3.732772 -1.228700 -2.258363
C -2.509650 -0.225107 1.870898
H -3.601079 -0.331307 1.967494
C -2.259996 -2.114511 0.079534
H -1.725412 -3.064945 -0.046009
H -1.968230 1.833676 1.569014
H -1.540563 -1.855374 -1.928166
C -1.889279 -1.565481 1.460575
H -0.792108 -1.473192 1.512651
H -2.159016 -2.327075 2.205179
C -4.033964 0.264762 -0.743787
H -4.879339 0.690455 -1.302270
H -4.474606 -0.454645 -0.038007
H -2.138523 0.005178 2.878847
H -3.331618 -2.358493 0.051136
H -4.107158 1.870783 0.667858
H -2.751627 0.219625 -2.488126
B -0.848382 0.637965 0.099419
C 0.494371 1.497738 0.319064
C 1.755282 1.014121 -0.449389
C 1.611657 1.060212 -1.965527
H 1.595473 2.114233 -2.279625
H 2.473809 0.587570 -2.448379
C 3.037685 1.735329 -0.042518
H 3.296659 1.581418 1.008996
H 2.915163 2.814678 -0.216611
O -0.849867 -0.322828 -0.853210
C 0.793046 1.645242 1.820674
H -0.117444 1.881767 2.382021
H 1.503098 2.462910 2.004810
H 3.876055 1.390103 -0.658380
C 0.090469 2.898155 -0.217456
H -0.292198 2.862396 -1.245973
H -0.697780 3.318968 0.419743
H 0.935938 3.598841 -0.192332
I 2.166370 -1.121400 0.076640
H 0.695354 0.574494 -2.309406
H 1.218531 0.724719 2.241643
43
B_b_Me_conf_3 Eopt -946.189609
C 1.442872 -1.908442 -0.229605
C 1.215141 -0.785292 0.802504
C 3.075883 0.761441 -0.932412
C 2.973257 -0.493601 -1.803158
H 0.547280 -1.950717 -0.869280
H 3.921937 -0.584396 -2.351565
C 2.327144 -0.601749 1.861448
H 2.336636 -1.483853 2.520055
C 3.972926 0.713461 0.308197
H 3.869984 1.699507 0.787122
H 0.328866 -1.098474 1.368376
H 3.503539 1.540243 -1.578024
C 3.760024 -0.381045 1.361962
H 4.377846 -0.114171 2.231336
H 4.170939 -1.329491 1.001899
C 2.679804 -1.839388 -1.135456
H 2.545440 -2.578601 -1.938148
H 3.567419 -2.172382 -0.588527
H 2.044965 0.247178 2.506945
H 5.012486 0.653312 -0.045587
H 1.469491 -2.873237 0.301614
H 2.202436 -0.278195 -2.559597
B 0.875601 0.627026 0.163003
C -0.491094 1.443103 0.439618
C -1.618770 0.960972 -0.527433
C -2.836468 1.877376 -0.558340
H -3.645986 1.422817 -1.139374
H -3.212994 2.106617 0.444867
C -1.107074 0.723770 -1.944407
H -0.665212 1.654992 -2.326569
H -1.927884 0.435125 -2.610620
O 1.782087 1.276452 -0.604287
C -0.236219 2.941767 0.156456
H 0.657434 3.264816 0.704182
H -1.077785 3.557265 0.500075
H -0.338298 -0.057745 -1.984598
C -0.876953 1.342807 1.923710
H -1.845192 1.824332 2.119365
H -0.938536 0.309350 2.280081
H -0.126414 1.865229 2.533059
I -2.424155 -1.017112 0.119447
H -2.558947 2.818739 -1.053031
H -0.056757 3.150828 -0.903991
43
B_b_Me_conf_4 Eopt -946.192312
C -3.333900 1.313037 0.210609
C -2.126826 0.834102 1.060026
C -1.919827 -1.207766 -1.135798
C -3.135351 -0.443337 -1.703087
H -3.427136 2.401700 0.323614
H -4.051436 -0.980512 -1.418608
C -2.442247 -0.409180 1.922555

H -3.125934 -0.109371 2.731243
C -2.236536 -2.146047 0.030313
H -1.277292 -2.523467 0.417259
H -1.905955 1.637723 1.771539
H -1.519210 -1.840855 -1.936791
C -3.075143 -1.578131 1.171748
H -3.273616 -2.387902 1.887613
H -4.059015 -1.274321 0.784854
C -3.246087 1.025428 -1.290999
H -2.382438 1.569952 -1.703575
H -4.131267 1.448696 -1.785652
H -1.517840 -0.756102 2.414854
H -2.765240 -3.010378 -0.396846
H -4.267678 0.890212 0.612220
H -3.087047 -0.477702 -2.799174
B -0.823810 0.574653 0.180301
C 0.510703 1.445333 0.412946
C 1.764712 1.001563 -0.389978
C 1.601251 1.099646 -1.901650
H 2.458742 0.647411 -2.411545
H 0.682244 0.623832 -2.251882
C 3.046799 1.719449 0.023386
H 3.325397 1.528830 1.063747
H 2.909380 2.803204 -0.107651
O -0.835453 -0.331607 -0.824804
C 0.839027 1.553336 1.911894
H 1.284279 0.626934 2.297360
H -0.059824 1.763634 2.500753
H 3.877898 1.406702 -0.619132
C 0.077330 2.856209 -0.069776
H -0.718298 3.235314 0.585074
H 0.908600 3.572512 -0.017926
H -0.306975 2.853564 -1.098074
I 2.191825 -1.146993 0.064436
H 1.578842 2.164316 -2.177164
H 1.543363 2.374425 2.102545
43
B_b_Me_conf_5 Eopt -946.191156
C 1.675872 -0.770430 1.781317
C 1.545191 0.631114 1.152799
C 2.362383 -0.725259 -1.366245
C 2.225138 -1.967794 -0.481376
H 0.656142 -1.152867 1.943156
H 2.920793 -2.721980 -0.876595
C 2.871152 1.401294 0.944286
H 3.277916 1.670280 1.931359
C 3.619030 0.136261 -1.215728
H 3.497028 0.968253 -1.926307
H 0.997914 1.228135 1.892591
H 2.376693 -1.094395 -2.400510
C 3.987197 0.703144 0.160601
H 4.792017 1.435587 0.003848
H 4.432056 -0.080208 0.782191
C 2.472867 -1.844214 1.024905
H 2.226435 -2.816911 1.474031
H 3.545109 -1.713957 1.203471
H 2.637208 2.358287 0.448598
H 4.467434 -0.458681 -1.584471
H 2.119738 -0.667374 2.784404
H 1.212427 -2.359319 -0.660468
B 0.707640 0.673537 -0.199550
C -0.637996 1.547507 -0.400305
C -1.860135 0.951720 0.361121
C -1.765959 1.018707 1.879858
H -2.634462 0.529584 2.334065
H -0.862354 0.545592 2.272810
C -3.200741 1.517129 -0.091229
H -3.195764 2.606778 0.063996
H -4.013407 1.094264 0.510252
O 1.182072 0.081969 -1.319081
C -0.943400 1.683735 -1.898568
H -0.040691 1.996784 -2.435277
H -1.718580 2.439463 -2.082145
H -3.410328 1.318617 -1.145969
C -0.348093 2.968500 0.137834
H -1.236998 3.611769 0.077563
H 0.005099 2.971454 1.175058
H 0.429476 3.435218 -0.482399
I -1.970605 -1.235509 -0.104598
H -1.776739 2.073429 2.191039
H -1.271897 0.732265 -2.335809
43
B_b_Me_conf_6 Eopt -946.195009
C -1.591855 -1.824201 -0.747761
C -1.346829 -0.367162 -1.217566
C -2.754629 0.260837 1.368321
C -2.776513 -1.275252 1.508674
H -0.764276 -2.447148 -1.111340
H -3.761671 -1.639828 1.183912
C -2.610818 0.283367 -1.832706
H -2.808685 -0.194437 -2.804031
C -3.791853 0.826963 0.395822
H -3.595332 1.906099 0.295320
H -0.607238 -0.407185 -2.026431
H -2.990326 0.688158 2.350352
C -3.876162 0.179228 -0.983666
H -4.704602 0.648741 -1.532174
H -4.155273 -0.878376 -0.871183
C -1.671294 -2.027437 0.767326
H -0.703043 -1.744140 1.206970
H -1.794769 -3.099372 0.976162
H -2.411798 1.346324 -2.050359
H -4.770383 0.730993 0.887874
H -2.500735 -2.225045 -1.222346
H -2.691635 -1.528089 2.573519
B -0.776770 0.591162 -0.083438
C 0.520229 1.543014 -0.279893
C 1.771727 1.001901 0.464375
C 3.069597 1.707691 0.095253
H 3.903955 1.297665 0.675874
H 3.310196 1.618238 -0.968255
C 1.609338 0.951631 1.977007
H 1.597859 1.981419 2.362182
H 2.455503 0.433152 2.440079
O -1.449758 0.772900 1.082840
C 0.150405 2.925271 0.307484
H -0.174285 2.859698 1.349939
H -0.684498 3.345934 -0.271102
H 0.677226 0.462045 2.277438
C 0.807395 1.750171 -1.774759
H 1.243999 0.858008 -2.241245
H -0.120733 1.989779 -2.310297
H 1.497798 2.589719 -1.932129
I 2.094716 -1.127516 -0.157586
H 2.976856 2.775480 0.343441
H 0.985445 3.635359 0.236871
43
B'_b_Me_conf_1 Eopt -946.195195
C 3.087957 1.116550 1.495181
C 2.309069 1.384698 0.179365
C 2.918871 -1.516306 -0.320789
C 3.591745 -1.409076 1.065850
H 2.662596 1.748259 2.286719
H 4.670401 -1.256573 0.920017
C 3.226910 1.571221 -1.048854
H 3.744872 2.536671 -0.945201
C 3.721721 -0.913933 -1.473342
H 3.082460 -0.935761 -2.369680
H 1.794517 2.344392 0.301030
H 2.799805 -2.581367 -0.552264
C 4.280007 0.488545 -1.261798
H 4.885461 0.755666 -2.139048
H 4.975818 0.480123 -0.410031
C 3.039178 -0.329796 2.000485
H 1.994690 -0.582096 2.246636
H 3.590038 -0.394009 2.948728
H 2.606779 1.653256 -1.956869
H 4.556550 -1.602680 -1.666932
H 4.135771 1.435146 1.384930
H 3.484419 -2.373971 1.577730

B 1.210513 0.253708 -0.050996
C -0.363615 0.636940 0.022846
C -1.273115 -0.594668 -0.142869
C -1.196656 -1.249849 -1.515173
H -1.467472 -0.561665 -2.321585
H -0.168534 -1.597797 -1.677410
C -1.134575 -1.622632 0.971803
H -1.819240 -2.461583 0.806909
H -1.342016 -1.198148 1.958789
O 1.574229 -1.027340 -0.293617
C -0.581938 1.676772 -1.093034
H -1.628118 2.011368 -1.114539
H -0.330185 1.286086 -2.087241
H -0.107199 -2.009625 0.960635
C -0.558755 1.323299 1.389178
H -1.605820 1.622236 1.529186
H -0.275939 0.677613 2.230490
H 0.051147 2.231652 1.450096
I -3.462530 0.035985 0.002070
H -1.866048 -2.115693 -1.562672
H 0.042088 2.560884 -0.917336
43
B'_b_Me_conf_2 Eopt -946.197009
C 2.609687 1.098788 -1.602856
C 1.965834 -0.276015 -1.280940
C 3.218350 0.226645 1.407788
C 3.650455 1.550569 0.741933
H 1.985772 1.613395 -2.345881
H 4.688485 1.443514 0.396151
C 2.990676 -1.435525 -1.320440
H 3.283662 -1.601486 -2.368019
C 4.037263 -0.997632 0.994325
H 3.552949 -1.882367 1.436299
H 1.242996 -0.492387 -2.076994
H 3.357696 0.337451 2.489501
C 4.259272 -1.214114 -0.499736
H 4.913105 -2.088182 -0.625852
H 4.819872 -0.362189 -0.910701
C 2.770330 2.042726 -0.408218
H 1.772579 2.276195 -0.004368
H 3.180234 2.999037 -0.761094
H 2.506428 -2.367791 -0.986738
H 5.016838 -0.899864 1.483820
H 3.585449 0.952778 -2.089082
H 3.661964 2.336295 1.508075
B 1.184205 -0.291855 0.105588
C -0.376776 -0.732381 0.245808
C -1.235329 0.532873 0.041854
C -1.144232 1.514661 1.202772
H -1.708940 2.426295 0.978498
H -1.519645 1.094079 2.139859
C -0.977073 1.258145 -1.273282
H -1.685519 2.082497 -1.403462
H -1.045080 0.599504 -2.144910
O 1.814286 -0.017449 1.272924
C -0.582462 -1.352043 1.634428
H 0.005491 -2.276710 1.713937
H -1.638566 -1.613716 1.791550
H 0.032580 1.694318 -1.244861
C -0.655261 -1.824006 -0.800896
H 0.148994 -2.572755 -0.794371
H -1.591877 -2.345623 -0.564491
H -0.736449 -1.433488 -1.821155
I -3.417050 -0.033471 -0.052742
H -0.088093 1.787629 1.350908
H -0.256986 -0.689768 2.442142
43
B'_b_Me_conf_3 Eopt -946.192421
C -2.674213 -0.890228 -1.764330
C -1.992108 0.389796 -1.239006
C -3.084425 -0.478096 1.392838
C -3.404347 -1.742400 0.591726
H -1.877622 -1.613308 -1.992512
H -4.262417 -2.226419 1.079368
C -2.937607 1.600009 -1.033756
H -3.266699 1.964630 -2.018391
C -3.989021 0.745004 1.210325
H -3.580126 1.515748 1.881523
H -1.301874 0.686220 -2.039741
H -3.171010 -0.757044 2.451194
C -4.190034 1.357839 -0.184311
H -4.685229 2.327882 -0.036528
H -4.902308 0.758817 -0.760263
C -3.724584 -1.599459 -0.896142
H -3.860692 -2.612027 -1.301876
H -4.701426 -1.117460 -1.004627
H -2.360265 2.427706 -0.587672
H -4.976277 0.480007 1.616070
H -3.142439 -0.655814 -2.733608
H -2.550331 -2.423832 0.731241
B -1.137215 0.270575 0.093748
C 0.415677 0.750291 0.210105
C 1.268006 -0.537577 0.165048
C 1.243024 -1.341625 1.458329
H 1.799551 -2.277288 1.333926
H 1.671187 -0.791780 2.301470
C 0.917571 -1.439100 -1.011699
H -0.069899 -1.888755 -0.816763
H 1.634222 -2.260730 -1.108978
O -1.707139 -0.108321 1.258703
C 0.592433 1.510180 1.531557
H 0.024332 2.450271 1.495706
H 1.650059 1.767499 1.692200
H 0.870770 -0.896820 -1.962498
C 0.736272 1.721628 -0.935986
H 1.666339 2.264278 -0.724979
H 0.852688 1.219225 -1.903300
H -0.063536 2.467187 -1.039543
I 3.442561 -0.017502 -0.116071
H 0.198483 -1.584951 1.700262
H 0.232092 0.940488 2.393546
43
B'_b_Me_conf_4 Eopt -946.192504
C -2.590285 1.483281 1.219503
C -2.024348 0.047815 1.251862
C -3.057226 -0.075496 -1.540774
C -3.387509 1.392810 -1.263960
H -1.741897 2.172021 1.077961
H -4.268523 1.645533 -1.871093
C -3.054096 -1.079897 1.498906
H -3.440308 -0.985422 2.525307
C -3.974712 -1.150747 -0.951309
H -3.530628 -2.114126 -1.245730
H -1.363981 0.019584 2.128333
H -3.117037 -0.197306 -2.630398
C -4.259975 -1.167948 0.555791
H -4.781254 -2.111228 0.772513
H -4.978663 -0.382072 0.807579
C -3.659858 1.832630 0.176377
H -3.768611 2.926478 0.167899
H -4.635699 1.453085 0.494344
H -2.514615 -2.041371 1.472016
H -4.935364 -1.082861 -1.482105
H -3.000408 1.720983 2.213557
H -2.552034 1.977876 -1.679170
B -1.142633 -0.327549 -0.014234
C 0.415956 -0.790844 0.060434
C 1.258701 0.500111 0.180656
C 0.925391 1.532785 -0.889420
H 1.613723 2.382813 -0.839917
H 0.948623 1.117187 -1.901557
C 1.221595 1.153100 1.556565
H 0.188072 1.444551 1.788719
H 1.832606 2.062357 1.560674
O -1.685390 -0.365345 -1.250307
C 0.731863 -1.594029 -1.210332
H -0.030334 -2.369341 -1.357803
H 1.705307 -2.092092 -1.119031
H 1.582758 0.489753 2.348055
C 0.609943 -1.728040 1.261207

H 1.670941 -1.997942 1.369818
H 0.270687 -1.297353 2.209477
H 0.050825 -2.659585 1.099622
I 3.441665 0.026478 -0.121817
H -0.090996 1.915948 -0.699372
H 0.742215 -0.973512 -2.112789
43
TS-I-Anti_b_Me_conf_1 Eopt -946.179353
C -2.840135 -0.853284 -1.777352
C -2.138463 0.418216 -1.250837
C -3.251834 -0.479386 1.390075
C -3.657078 -1.696811 0.559534
H -2.058559 -1.603257 -1.967614
H -4.556879 -2.118454 1.028637
C -3.057474 1.644781 -1.021773
H -3.409295 2.002473 -2.000317
C -4.064912 0.809899 1.255314
H -3.572948 1.542945 1.912928
H -1.441025 0.711603 -2.044548
H -3.322733 -0.779620 2.442285
C -4.286463 1.442394 -0.126870
H -4.731729 2.431562 0.047646
H -5.047373 0.880876 -0.676906
C -3.941328 -1.509447 -0.932205
H -4.125889 -2.507117 -1.354321
H -4.885885 -0.970989 -1.053989
H -2.445507 2.462876 -0.606807
H -5.049075 0.612612 1.703259
H -3.270101 -0.616535 -2.762492
H -2.861830 -2.445960 0.699542
B -1.315323 0.212543 0.074850
C 0.319785 0.683744 0.208558
C 0.851226 -0.652845 0.154328
C 1.045947 -1.434270 1.406607
H 1.563828 -2.375556 1.204466
H 1.579028 -0.864693 2.171455
C 0.778949 -1.467032 -1.090721
H -0.068283 -2.162966 -0.949505
H 1.672271 -2.090779 -1.186048
O -1.845586 -0.198082 1.224603
C 0.473746 1.409462 1.544215
H -0.068762 2.362639 1.511576
H 1.539405 1.628262 1.704918
H 0.623171 -0.888132 -2.003006
C 0.641575 1.629484 -0.949260
H 1.607174 2.100737 -0.727499
H 0.726067 1.129505 -1.917099
H -0.118007 2.417071 -1.025845
I 3.805427 0.015579 -0.089258
H 0.040711 -1.663784 1.800391
H 0.094658 0.831504 2.390833
43
TS-I-Anti_b_Me_conf_2 Eopt -946.184225
C 2.748932 1.008207 -1.654538
C 2.099444 -0.354117 -1.283943
C 3.366756 0.258439 1.398314
C 3.799546 1.549775 0.674199
H 2.121198 1.496446 -2.411455
H 4.835535 1.422294 0.330722
C 3.119218 -1.519161 -1.269231
H 3.413046 -1.723669 -2.308832
C 4.164274 -0.992066 1.031806
H 3.670971 -1.853083 1.508692
H 1.370771 -0.599521 -2.064047
H 3.493556 0.410593 2.475118
C 4.385293 -1.267404 -0.453236
H 5.034916 -2.148495 -0.542712
H 4.950572 -0.435458 -0.896814
C 2.918036 1.995670 -0.495569
H 1.920840 2.253667 -0.102521
H 3.332412 2.933699 -0.887903
H 2.627456 -2.434261 -0.902229
H 5.144096 -0.885566 1.518004
H 3.721164 0.835919 -2.137622
H 3.815519 2.367579 1.405135
B 1.356704 -0.278617 0.105343
C -0.287859 -0.691891 0.281985
C -0.803077 0.630146 0.045412
C -0.939512 1.582141 1.185390
H -1.411770 2.515206 0.867832
H -1.493095 1.144661 2.020039
C -0.792573 1.255850 -1.305906
H 0.111716 1.889538 -1.346989
H -1.644718 1.933148 -1.407828
O 1.950168 0.028224 1.259902
C -0.479617 -1.240050 1.694290
H 0.050846 -2.194987 1.793237
H -1.551507 -1.422785 1.855514
H -0.772591 0.546718 -2.134400
C -0.575662 -1.778899 -0.755887
H -1.536366 -2.239384 -0.491383
H -0.650830 -1.405238 -1.779347
H 0.198187 -2.556593 -0.724934
I -3.763131 -0.048011 -0.053190
H 0.078225 1.807566 1.549659
H -0.108240 -0.565937 2.469650
43
TS-I-Anti_b_Me_conf_3 Eopt -946.183466
C 3.028694 -0.880253 1.709815
C 2.088609 0.277526 1.269078
C 3.407365 -0.201918 -1.415311
C 4.141062 -1.307355 -0.625935
H 2.524291 -1.456310 2.496259
H 5.126864 -0.922180 -0.330902
C 2.822936 1.631823 1.135680
H 3.072839 1.977950 2.149166
C 3.912420 1.215027 -1.154653
H 3.226080 1.914844 -1.656361
H 1.341973 0.414382 2.058743
H 3.541952 -0.397118 -2.484209
C 4.101671 1.616565 0.304170
H 4.543055 2.621877 0.329771
H 4.841318 0.953400 0.774712
C 3.415211 -1.868311 0.601978
H 2.505121 -2.388445 0.260711
H 4.053882 -2.649398 1.034995
H 2.138875 2.384959 0.713343
H 4.877816 1.298921 -1.673188
H 3.935397 -0.462670 2.171164
H 4.329704 -2.148396 -1.304336
B 1.363148 -0.141591 -0.065616
C -0.276126 -0.604225 -0.114770
C -0.838531 0.717918 -0.137136
C -0.912005 1.582981 1.071705
H -1.769703 2.255832 0.989331
H -0.953411 1.037867 2.015064
C -0.981527 1.434357 -1.438286
H 0.036311 1.659069 -1.804397
H -1.516437 2.378810 -1.311563
O 1.979680 -0.295488 -1.238020
C -0.469288 -1.459563 1.140669
H 0.271107 -2.269981 1.161105
H -1.468332 -1.912889 1.084457
H -1.474603 0.822899 -2.197732
C -0.490663 -1.443708 -1.371828
H -1.558919 -1.688260 -1.449839
H -0.168785 -0.938299 -2.285147
H 0.075087 -2.379198 -1.290427
I -3.801466 -0.049456 0.033844
H -0.012410 2.223292 1.059188
H -0.393512 -0.903113 2.077565
43
TS-I-Gauche_b_Me_conf_1 Eopt -
946.173976
C 0.285878 2.034105 -0.263968
C 1.569648 1.798159 0.371881
I 2.076095 -1.374998 -0.077234
C 0.379224 2.464347 -1.727076
H 0.952318 3.396622 -1.821955
H 0.851344 1.699828 -2.354419

H -0.626127 2.649267 -2.121965
C 2.827476 1.980764 -0.373537
H 2.929137 3.085726 -0.417774
H 3.692632 1.571717 0.151262
H 2.782898 1.627281 -1.405430
C -2.078044 -0.489337 1.549540
C -1.425862 0.050104 -1.324687
H -2.049338 -0.485369 2.646446
C -1.783403 -1.923464 1.114549
C -3.421741 0.133609 1.158197
H -0.917323 0.387164 -2.235138
C -1.485801 -1.484951 -1.455867
C -2.815582 0.740274 -1.327719
H -2.355228 -2.582252 1.783656
H -0.721179 -2.102419 1.333654
C -2.098579 -2.330398 -0.326368
H -3.411450 1.152030 1.576808
H -4.201377 -0.413502 1.707827
C -3.840257 0.205147 -0.319772
H -0.458770 -1.829479 -1.622123
H -2.037877 -1.714464 -2.381546
H -2.686244 1.823539 -1.172206
H -3.250260 0.633809 -2.332594
H -1.735642 -3.359631 -0.457165
H -4.725152 0.855299 -0.367453
B -0.666614 0.661115 -0.078984
H -4.187199 -0.776316 -0.656983
O -1.007963 0.402174 1.189116
H -3.185810 -2.394997 -0.443845
C -0.510566 3.104398 0.546382
H -1.496534 3.239221 0.088426
H -0.651295 2.823169 1.592086
H 0.021984 4.063473 0.494562
C 1.678543 1.690279 1.839241
H 1.774008 2.738248 2.185492
H 0.785302 1.267422 2.304646
H 2.585297 1.158345 2.136359
43
TS-I-Gauche_b_Me_conf_2 Eopt -
946.173677
C -0.397632 2.069359 -0.426474
C 0.608338 2.304015 0.560625
I 2.755547 -0.914215 -0.073055
C 0.051975 2.192843 -1.878548
H 0.400904 3.213847 -2.083462
H 0.855871 1.490695 -2.125306
H -0.794444 1.987844 -2.543853
C 2.000976 2.582285 0.176972
H 1.975966 3.676467 -0.013370
H 2.708653 2.398974 0.989680
H 2.319016 2.101163 -0.748239
C -2.557668 -0.593158 1.434699
C -1.157764 -0.546920 -1.257814
H -2.870538 -0.304623 2.443785
C -2.030788 -2.037804 1.481902
C -3.764002 -0.376537 0.518685
H -0.486271 -0.286004 -2.081866
C -0.840215 -2.010301 -0.844124
C -2.603511 -0.405226 -1.802488
H -2.842943 -2.710017 1.170928
H -1.802038 -2.291218 2.524587
C -0.772963 -2.303619 0.656025
H -3.957041 0.706626 0.468805
H -4.625450 -0.821941 1.035668
C -3.692216 -0.969326 -0.889028
H 0.139072 -2.272807 -1.260196
H -1.568214 -2.683497 -1.321197
H -2.822192 0.650154 -2.027231
H -2.652163 -0.938008 -2.763106
H 0.055363 -1.722788 1.084372
H -4.665506 -0.811209 -1.372962
B -0.977423 0.492008 -0.086338
H -3.571985 -2.058835 -0.812304
O -1.516465 0.360003 1.127603
H -0.496813 -3.358374 0.790051
C -1.711554 2.869444 -0.196440
H -2.506508 2.474783 -0.838708
H -2.056120 2.844911 0.838675
H -1.525538 3.909627 -0.493092
C 0.332401 2.364730 2.008705
H 0.854085 3.235478 2.429877
H -0.718494 2.359196 2.288531
H 0.835911 1.484178 2.446544
43
TS-I-Gauche_b_Me_conf_3 Eopt -
946.174913
C 0.167014 1.972349 -0.276375
C 1.506988 1.796701 0.278131
I 2.176419 -1.287012 -0.102348
C 0.159903 2.362901 -1.755360
H 0.707309 3.300639 -1.919059
H 0.596315 1.579776 -2.384481
H -0.873220 2.510723 -2.087406
C 2.698568 2.074054 -0.544053
H 2.704140 3.182850 -0.604866
H 3.624244 1.744579 -0.069042
H 2.619946 1.703688 -1.568054
C -2.128882 -0.961846 -1.383259
C -1.461770 0.226716 1.303966
H -2.077990 -1.223235 -2.445974
C -3.525717 -0.366561 -1.101498
C -1.809517 -2.238992 -0.606474
H -1.002322 0.798737 2.116353
C -2.949816 0.691449 1.228938
C -1.340252 -1.251836 1.722843
H -4.171396 -1.159754 -0.700408
H -3.973498 -0.059566 -2.055161
C -3.552737 0.851936 -0.173284
H -0.745394 -2.468846 -0.760302
H -2.382128 -3.041599 -1.093489
C -2.139143 -2.243214 0.883044
H -3.036977 1.666311 1.727052
H -3.568548 -0.004263 1.815174
H -0.281634 -1.544002 1.718591
H -1.685904 -1.331131 2.765034
H -3.045176 1.683287 -0.686481
H -1.956013 -3.253969 1.272975
B -0.765780 0.604871 -0.072405
H -3.215249 -2.060602 1.020957
O -1.115095 0.043552 -1.238991
H -4.598962 1.168639 -0.065127
C -0.572120 3.071093 0.550119
H -1.581005 3.198495 0.142065
H -0.652997 2.831583 1.613661
H -0.042264 4.026753 0.441589
C 1.724905 1.728435 1.737535
H 1.802987 2.788115 2.050948
H 0.897574 1.279849 2.289894
H 2.673109 1.242678 1.978292
43
TS-I-Gauche_b_Me_conf_4 Eopt -
946.175538
C 0.235808 2.125998 0.263949
C -1.066536 2.092371 -0.356055
I -2.470808 -1.071808 0.040269
C 0.236835 2.610555 1.712438
H -0.184193 3.622616 1.780208
H -0.336086 1.950443 2.373136
H 1.265330 2.650896 2.087252
C -2.273269 2.419382 0.419851
H -2.199913 3.519916 0.545733
H -3.196891 2.179381 -0.108243
H -2.268111 1.995759 1.427342
C 1.848586 -1.019350 -1.486259
C 1.616348 -0.089719 1.382666
H 1.677921 -1.106146 -2.564543
C 1.316175 -2.301402 -0.851275
C 3.353038 -0.769373 -1.251291
H 1.321256 0.440827 2.292290
C 1.193675 -1.557761 1.611209

C 3.160751 0.060256 1.238899
H 1.690283 -3.125141 -1.476278
H 0.221637 -2.299531 -0.948120
C 1.717980 -2.568364 0.596180
H 3.801686 -0.427512 -2.192554
H 3.829184 -1.731002 -1.016754
C 3.706957 0.267927 -0.180725
H 0.098037 -1.618043 1.648601
H 1.560957 -1.850298 2.606545
H 3.484932 0.925675 1.832980
H 3.644293 -0.816741 1.694542
H 1.344664 -3.562770 0.876764
H 3.381502 1.252628 -0.544019
B 0.924789 0.585748 0.128912
H 4.802320 0.324676 -0.122660
O 1.069343 0.138018 -1.121694
H 2.814102 -2.629137 0.667846
C 1.227252 2.981135 -0.582560
H 2.204476 2.988561 -0.087929
H 1.350182 2.609658 -1.602890
H 0.859857 4.015211 -0.614027
C -1.213966 1.983734 -1.816843
H -1.154331 3.030580 -2.176275
H -0.401449 1.428598 -2.290909
H -2.195332 1.591512 -2.094426
43
B_Me_b_conf_1 Eopt -
946.194392
C -1.702416 -1.709257 -1.146740
C -1.457392 -0.196111 -1.316343
C -2.456001 -0.102061 1.514087
C -2.456060 -1.616668 1.300046
H -0.752472 -2.205555 -0.902435
H -3.214379 -2.027915 1.981999
C -2.777977 0.577309 -1.605343
H -3.541751 -0.126823 -1.969662
C -3.649034 0.639731 0.868810
H -4.026891 1.378520 1.587296
H -0.826727 -0.107859 -2.208869
H -2.511805 0.054587 2.597963
C -3.346814 1.389361 -0.433404
H -2.648535 2.208102 -0.200749
H -4.274787 1.877333 -0.761850
C -2.748601 -2.107561 -0.112794
H -2.821905 -3.203972 -0.092405
H -3.739320 -1.748293 -0.427957
H -2.602247 1.283811 -2.428320
H -4.466391 -0.077132 0.709432
H -2.016702 -2.108326 -2.123739
H -1.482498 -2.002128 1.641478
B -0.709140 0.535424 -0.111747
C 0.567178 1.516740 -0.308595
C 1.829217 0.976268 0.422252
C 3.136166 1.608199 -0.036932
H 3.974202 1.215122 0.550153
H 3.343281 1.435798 -1.096969
C 1.739671 1.019490 1.941335
H 0.805435 0.590117 2.312804
H 1.793100 2.070371 2.261493
O -1.216606 0.507345 1.148133
C 0.188773 2.884384 0.306361
H 1.040222 3.579358 0.296415
H -0.177450 2.788776 1.333809
H 2.587887 0.492255 2.390877
C 0.850547 1.752339 -1.797899
H 1.257113 0.857415 -2.287829
H -0.076279 2.028796 -2.317629
H 1.561689 2.576299 -1.943311
I 2.024918 -1.196125 -0.093193
H 3.080251 2.693893 0.135949
H -0.608709 3.343254 -0.294091
43
B_Me_b_conf_2 Eopt -
946.192619
C -3.350043 1.386272 0.032453
C -2.168299 0.963027 0.933041
C -1.932336 -1.211491 -1.131349
C -3.133670 -0.476405 -1.725119
H -3.009019 2.157745 -0.677623
H -3.732772 -1.228700 -2.258363
C -2.509650 -0.225107 1.870898
H -3.601079 -0.331307 1.967494
C -2.259996 -2.114511 0.079534
H -1.725412 -3.064945 -0.046009
H -1.968230 1.833676 1.569014
H -1.540563 -1.855374 -1.928166
C -1.889279 -1.565481 1.460575
H -0.792108 -1.473192 1.512651
H -2.159016 -2.327075 2.205179
C -4.033964 0.264762 -0.743787
H -4.879339 0.690455 -1.302270
H -4.474606 -0.454645 -0.038007
H -2.138523 0.005178 2.878847
H -3.331618 -2.358493 0.051136
H -4.107158 1.870783 0.667858
H -2.751627 0.219625 -2.488126
B -0.848382 0.637965 0.099419
C 0.494371 1.497738 0.319064
C 1.755282 1.014121 -0.449389
C 1.611657 1.060212 -1.965527
H 1.595473 2.114233 -2.279625
H 2.473809 0.587570 -2.448379
C 3.037685 1.735329 -0.042518
H 3.296659 1.581418 1.008996
H 2.915163 2.814678 -0.216611
O -0.849867 -0.322828 -0.853210
C 0.793046 1.645242 1.820674
H -0.117444 1.881767 2.382021
H 1.503098 2.462910 2.004810
H 3.876055 1.390103 -0.658380
C 0.090469 2.898155 -0.217456
H -0.292198 2.862396 -1.245973
H -0.697780 3.318968 0.419743
H 0.935938 3.598841 -0.192332
I 2.166370 -1.121400 0.076640
H 0.695354 0.574494 -2.309406
H 1.218531 0.724719 2.241643
43
B_Me_b_conf_3 Eopt -
946.195009
C -1.591855 -1.824201 -0.747761
C -1.346829 -0.367162 -1.217566
C -2.754629 0.260837 1.368321
C -2.776513 -1.275252 1.508674
H -0.764276 -2.447148 -1.111340
H -3.761671 -1.639828 1.183912
C -2.610818 0.283367 -1.832706
H -2.808685 -0.194437 -2.804031
C -3.791853 0.826963 0.395822
H -3.595332 1.906099 0.295320
H -0.607238 -0.407185 -2.026431
H -2.990326 0.688158 2.350352
C -3.876162 0.179228 -0.983666
H -4.704602 0.648741 -1.532174
H -4.155273 -0.878376 -0.871183
C -1.671294 -2.027437 0.767326
H -0.703043 -1.744140 1.206970
H -1.794769 -3.099372 0.976162
H -2.411798 1.346324 -2.050359
H -4.770383 0.730993 0.887874
H -2.500735 -2.225045 -1.222346
H -2.691635 -1.528089 2.573519
B -0.776770 0.591162 -0.083438
C 0.520229 1.543014 -0.279893
C 1.771727 1.001901 0.464375
C 3.069597 1.707691 0.095253
H 3.903955 1.297665 0.675874
H 3.310196 1.618238 -0.968255
C 1.609338 0.951631 1.977007
H 1.597859 1.981419 2.362182

H 2.455503 0.433152 2.440079
O -1.449758 0.772900 1.082840
C 0.150405 2.925271 0.307484
H -0.174285 2.859698 1.349939
H -0.684498 3.345934 -0.271102
H 0.677226 0.462045 2.277438
C 0.807395 1.750171 -1.774759
H 1.243999 0.858008 -2.241245
H -0.120733 1.989779 -2.310297
H 1.497798 2.589719 -1.932129
I 2.094716 -1.127516 -0.157586
H 2.976856 2.775480 0.343441
H 0.985445 3.635359 0.236871
43
B_Me_b_conf_4 Eopt -
946.192312
C -3.333900 1.313037 0.210609
C -2.126826 0.834102 1.060026
C -1.919827 -1.207766 -1.135798
C -3.135351 -0.443337 -1.703087
H -3.427136 2.401700 0.323614
H -4.051436 -0.980512 -1.418608
C -2.442247 -0.409180 1.922555
H -3.125934 -0.109371 2.731243
C -2.236536 -2.146047 0.030313
H -1.277292 -2.523467 0.417259
H -1.905955 1.637723 1.771539
H -1.519210 -1.840855 -1.936791
C -3.075143 -1.578131 1.171748
H -3.273616 -2.387902 1.887613
H -4.059015 -1.274321 0.784854
C -3.246087 1.025428 -1.290999
H -2.382438 1.569952 -1.703575
H -4.131267 1.448696 -1.785652
H -1.517840 -0.756102 2.414854
H -2.765240 -3.010378 -0.396846
H -4.267678 0.890212 0.612220
H -3.087047 -0.477702 -2.799174
B -0.823810 0.574653 0.180301
C 0.510703 1.445333 0.412946
C 1.764712 1.001563 -0.389978
C 1.601251 1.099646 -1.901650
H 2.458742 0.647411 -2.411545
H 0.682244 0.623832 -2.251882
C 3.046799 1.719449 0.023386
H 3.325397 1.528830 1.063747
H 2.909380 2.803204 -0.107651
O -0.835453 -0.331607 -0.824804
C 0.839027 1.553336 1.911894
H 1.284279 0.626934 2.297360
H -0.059824 1.763634 2.500753
H 3.877898 1.406702 -0.619132
C 0.077330 2.856209 -0.069776
H -0.718298 3.235314 0.585074
H 0.908600 3.572512 -0.017926
H -0.306975 2.853564 -1.098074
I 2.191825 -1.146993 0.064436
H 1.578842 2.164316 -2.177164
H 1.543363 2.374425 2.102545
43
B_Me_b_conf_5 Eopt -
946.191156
C 1.675872 -0.770430 1.781317
C 1.545191 0.631114 1.152799
C 2.362383 -0.725259 -1.366245
C 2.225138 -1.967794 -0.481376
H 0.656142 -1.152867 1.943156
H 2.920793 -2.721980 -0.876595
C 2.871152 1.401294 0.944286
H 3.277916 1.670280 1.931359
C 3.619030 0.136261 -1.215728
H 3.497028 0.968253 -1.926307
H 0.997914 1.228135 1.892591
H 2.376693 -1.094395 -2.400510
C 3.987197 0.703144 0.160601
H 4.792017 1.435587 0.003848
H 4.432056 -0.080208 0.782191
C 2.472867 -1.844214 1.024905
H 2.226435 -2.816911 1.474031
H 3.545109 -1.713957 1.203471
H 2.637208 2.358287 0.448598
H 4.467434 -0.458681 -1.584471
H 2.119738 -0.667374 2.784404
H 1.212427 -2.359319 -0.660468
B 0.707640 0.673537 -0.199550
C -0.637996 1.547507 -0.400305
C -1.860135 0.951720 0.361121
C -1.765959 1.018707 1.879858
H -2.634462 0.529584 2.334065
H -0.862354 0.545592 2.272810
C -3.200741 1.517129 -0.091229
H -3.195764 2.606778 0.063996
H -4.013407 1.094264 0.510252
O 1.182072 0.081969 -1.319081
C -0.943400 1.683735 -1.898568
H -0.040691 1.996784 -2.435277
H -1.718580 2.439463 -2.082145
H -3.410328 1.318617 -1.145969
C -0.348093 2.968500 0.137834
H -1.236998 3.611769 0.077563
H 0.005099 2.971454 1.175058
H 0.429476 3.435218 -0.482399
I -1.970605 -1.235509 -0.104598
H -1.776739 2.073429 2.191039
H -1.271897 0.732265 -2.335809
43
B_Me_b_conf_6 Eopt -
946.189609
C 1.442872 -1.908442 -0.229605
C 1.215141 -0.785292 0.802504
C 3.075883 0.761441 -0.932412
C 2.973257 -0.493601 -1.803158
H 0.547280 -1.950717 -0.869280
H 3.921937 -0.584396 -2.351565
C 2.327144 -0.601749 1.861448
H 2.336636 -1.483853 2.520055
C 3.972926 0.713461 0.308197
H 3.869984 1.699507 0.787122
H 0.328866 -1.098474 1.368376
H 3.503539 1.540243 -1.578024
C 3.760024 -0.381045 1.361962
H 4.377846 -0.114171 2.231336
H 4.170939 -1.329491 1.001899
C 2.679804 -1.839388 -1.135456
H 2.545440 -2.578601 -1.938148
H 3.567419 -2.172382 -0.588527
H 2.044965 0.247178 2.506945
H 5.012486 0.653312 -0.045587
H 1.469491 -2.873237 0.301614
H 2.202436 -0.278195 -2.559597
B 0.875601 0.627026 0.163003
C -0.491094 1.443103 0.439618
C -1.618770 0.960972 -0.527433
C -2.836468 1.877376 -0.558340
H -3.645986 1.422817 -1.139374
H -3.212994 2.106617 0.444867
C -1.107074 0.723770 -1.944407
H -0.665212 1.654992 -2.326569
H -1.927884 0.435125 -2.610620
O 1.782087 1.276452 -0.604287
C -0.236219 2.941767 0.156456
H 0.657434 3.264816 0.704182
H -1.077785 3.557265 0.500075
H -0.338298 -0.057745 -1.984598
C -0.876953 1.342807 1.923710
H -1.845192 1.824332 2.119365
H -0.938536 0.309350 2.280081
H -0.126414 1.865229 2.533059
I -2.424155 -1.017112 0.119447
H -2.558947 2.818739 -1.053031

H -0.056757 3.150828 -0.903991
43
B’_Me_b_conf_1 Eopt -
946.192504
C -2.590285 1.483281 1.219503
C -2.024348 0.047815 1.251862
C -3.057226 -0.075496 -1.540774
C -3.387509 1.392810 -1.263960
H -1.741897 2.172021 1.077961
H -4.268523 1.645533 -1.871093
C -3.054096 -1.079897 1.498906
H -3.440308 -0.985422 2.525307
C -3.974712 -1.150747 -0.951309
H -3.530628 -2.114126 -1.245730
H -1.363981 0.019584 2.128333
H -3.117037 -0.197306 -2.630398
C -4.259975 -1.167948 0.555791
H -4.781254 -2.111228 0.772513
H -4.978663 -0.382072 0.807579
C -3.659858 1.832630 0.176377
H -3.768611 2.926478 0.167899
H -4.635699 1.453085 0.494344
H -2.514615 -2.041371 1.472016
H -4.935364 -1.082861 -1.482105
H -3.000408 1.720983 2.213557
H -2.552034 1.977876 -1.679170
B -1.142633 -0.327549 -0.014234
C 0.415956 -0.790844 0.060434
C 1.258701 0.500111 0.180656
C 0.925391 1.532785 -0.889420
H 1.613723 2.382813 -0.839917
H 0.948623 1.117187 -1.901557
C 1.221595 1.153100 1.556565
H 0.188072 1.444551 1.788719
H 1.832606 2.062357 1.560674
O -1.685390 -0.365345 -1.250307
C 0.731863 -1.594029 -1.210332
H -0.030334 -2.369341 -1.357803
H 1.705307 -2.092092 -1.119031
H 1.582758 0.489753 2.348055
C 0.609943 -1.728040 1.261207
H 1.670941 -1.997942 1.369818
H 0.270687 -1.297353 2.209477
H 0.050825 -2.659585 1.099622
I 3.441665 0.026478 -0.121817
H -0.090996 1.915948 -0.699372
H 0.742215 -0.973512 -2.112789
43
B’_Me_b_conf_2 Eopt -
946.197009
C 2.609687 1.098788 -1.602856
C 1.965834 -0.276015 -1.280940
C 3.218350 0.226645 1.407788
C 3.650455 1.550569 0.741933
H 1.985772 1.613395 -2.345881
H 4.688485 1.443514 0.396151
C 2.990676 -1.435525 -1.320440
H 3.283662 -1.601486 -2.368019
C 4.037263 -0.997632 0.994325
H 3.552949 -1.882367 1.436299
H 1.242996 -0.492387 -2.076994
H 3.357696 0.337451 2.489501
C 4.259272 -1.214114 -0.499736
H 4.913105 -2.088182 -0.625852
H 4.819872 -0.362189 -0.910701
C 2.770330 2.042726 -0.408218
H 1.772579 2.276195 -0.004368
H 3.180234 2.999037 -0.761094
H 2.506428 -2.367791 -0.986738
H 5.016838 -0.899864 1.483820
H 3.585449 0.952778 -2.089082
H 3.661964 2.336295 1.508075
B 1.184205 -0.291855 0.105588
C -0.376776 -0.732381 0.245808
C -1.235329 0.532873 0.041854
C -1.144232 1.514661 1.202772
H -1.708940 2.426295 0.978498
H -1.519645 1.094079 2.139859
C -0.977073 1.258145 -1.273282
H -1.685519 2.082497 -1.403462
H -1.045080 0.599504 -2.144910
O 1.814286 -0.017449 1.272924
C -0.582462 -1.352043 1.634428
H 0.005491 -2.276710 1.713937
H -1.638566 -1.613716 1.791550
H 0.032580 1.694318 -1.244861
C -0.655261 -1.824006 -0.800896
H 0.148994 -2.572755 -0.794371
H -1.591877 -2.345623 -0.564491
H -0.736449 -1.433488 -1.821155
I -3.417050 -0.033471 -0.052742
H -0.088093 1.787629 1.350908
H -0.256986 -0.689768 2.442142
43
B’_Me_b_conf_3 Eopt -
946.192421
C -2.674213 -0.890228 -1.764330
C -1.992108 0.389796 -1.239006
C -3.084425 -0.478096 1.392838
C -3.404347 -1.742400 0.591726
H -1.877622 -1.613308 -1.992512
H -4.262417 -2.226419 1.079368
C -2.937607 1.600009 -1.033756
H -3.266699 1.964630 -2.018391
C -3.989021 0.745004 1.210325
H -3.580126 1.515748 1.881523
H -1.301874 0.686220 -2.039741
H -3.171010 -0.757044 2.451194
C -4.190034 1.357839 -0.184311
H -4.685229 2.327882 -0.036528
H -4.902308 0.758817 -0.760263
C -3.724584 -1.599459 -0.896142
H -3.860692 -2.612027 -1.301876
H -4.701426 -1.117460 -1.004627
H -2.360265 2.427706 -0.587672
H -4.976277 0.480007 1.616070
H -3.142439 -0.655814 -2.733608
H -2.550331 -2.423832 0.731241
B -1.137215 0.270575 0.093748
C 0.415677 0.750291 0.210105
C 1.268006 -0.537577 0.165048
C 1.243024 -1.341625 1.458329
H 1.799551 -2.277288 1.333926
H 1.671187 -0.791780 2.301470
C 0.917571 -1.439100 -1.011699
H -0.069899 -1.888755 -0.816763
H 1.634222 -2.260730 -1.108978
O -1.707139 -0.108321 1.258703
C 0.592433 1.510180 1.531557
H 0.024332 2.450271 1.495706
H 1.650059 1.767499 1.692200
H 0.870770 -0.896820 -1.962498
C 0.736272 1.721628 -0.935986
H 1.666339 2.264278 -0.724979
H 0.852688 1.219225 -1.903300
H -0.063536 2.467187 -1.039543
I 3.442561 -0.017502 -0.116071
H 0.198483 -1.584951 1.700262
H 0.232092 0.940488 2.393546
43
B’_Me_b_conf_4 Eopt -
946.195195
C 3.087957 1.116550 1.495181
C 2.309069 1.384698 0.179365
C 2.918871 -1.516306 -0.320789
C 3.591745 -1.409076 1.065850
H 2.662596 1.748259 2.286719
H 4.670401 -1.256573 0.920017
C 3.226910 1.571221 -1.048854
H 3.744872 2.536671 -0.945201

C 3.721721 -0.913933 -1.473342
H 3.082460 -0.935761 -2.369680
H 1.794517 2.344392 0.301030
H 2.799805 -2.581367 -0.552264
C 4.280007 0.488545 -1.261798
H 4.885461 0.755666 -2.139048
H 4.975818 0.480123 -0.410031
C 3.039178 -0.329796 2.000485
H 1.994690 -0.582096 2.246636
H 3.590038 -0.394009 2.948728
H 2.606779 1.653256 -1.956869
H 4.556550 -1.602680 -1.666932
H 4.135771 1.435146 1.384930
H 3.484419 -2.373971 1.577730
B 1.210513 0.253708 -0.050996
C -0.363615 0.636940 0.022846
C -1.273115 -0.594668 -0.142869
C -1.196656 -1.249849 -1.515173
H -1.467472 -0.561665 -2.321585
H -0.168534 -1.597797 -1.677410
C -1.134575 -1.622632 0.971803
H -1.819240 -2.461583 0.806909
H -1.342016 -1.198148 1.958789
O 1.574229 -1.027340 -0.293617
C -0.581938 1.676772 -1.093034
H -1.628118 2.011368 -1.114539
H -0.330185 1.286086 -2.087241
H -0.107199 -2.009625 0.960635
C -0.558755 1.323299 1.389178
H -1.605820 1.622236 1.529186
H -0.275939 0.677613 2.230490
H 0.051147 2.231652 1.450096
I -3.462530 0.035985 0.002070
H -1.866048 -2.115693 -1.562672
H 0.042088 2.560884 -0.917336
29
F_b_conf_1 Eopt -528.005206
C -0.127265 1.446180 -1.219182
C 0.605841 1.012257 0.068073
C -1.184721 -1.372392 -0.091224
C -1.674480 -0.664076 -1.359386
H 0.527103 1.205309 -2.073109
H -2.741872 -0.904354 -1.473329
C -0.137946 1.277587 1.394543
H -0.245179 2.365381 1.530284
C -1.673759 -0.841470 1.261029
H -1.150639 -1.437145 2.024926
H 1.505444 1.642282 0.112684
H -1.566316 -2.400042 -0.161126
C -1.524921 0.649329 1.586698
H -1.800774 0.777460 2.643314
H -2.271662 1.222243 1.027864
C -1.517002 0.855763 -1.491096
H -1.791122 1.118792 -2.522907
H -2.260325 1.358505 -0.864139
H 0.514569 0.936616 2.215017
H -2.739136 -1.101374 1.346597
H -0.227443 2.543022 -1.219158
H -1.158587 -1.158214 -2.197036
B 1.097634 -0.492209 -0.030248
O 0.232028 -1.540016 -0.102405
O 2.414922 -0.861759 -0.052977
C 3.473257 0.068125 0.006877
H 3.448153 0.761360 -0.846094
H 3.445816 0.649423 0.939630
H 4.414173 -0.491837 -0.027111
29
F_b_conf_2 Eopt -528.008530
C 0.131060 -0.521994 1.804126
C -0.600988 0.517862 0.914650
C 1.290263 -0.523762 -1.186635
C 1.750966 -1.439879 -0.029572
H -0.579664 -0.914535 2.543715
H 2.654147 -1.006460 0.424182
C 0.210708 1.817863 0.719460
H 0.236527 2.356360 1.679226
C 1.803743 0.916354 -1.096111
H 1.306231 1.494373 -1.890361
H -1.512710 0.817514 1.446375
H 1.704970 -0.934362 -2.115315
C 1.648871 1.627343 0.245182
H 2.124599 2.615097 0.168023
H 2.218297 1.082872 1.012877
C 0.710748 -1.727712 1.056605
H -0.120646 -2.288854 0.601009
H 1.167301 -2.411217 1.785805
H -0.318377 2.476400 0.011220
H 2.873026 0.887150 -1.352389
H 0.926291 -0.028118 2.383653
H 2.055216 -2.406022 -0.453045
B -1.027856 -0.117941 -0.479421
O -0.118774 -0.579015 -1.387901
O -2.325937 -0.259458 -0.885682
C -3.421635 0.139356 -0.092386
H -3.402283 1.220812 0.104386
H -4.338630 -0.098618 -0.642280
H -3.439758 -0.397493 0.866783
48
G_b_conf_1 Eopt -1061.287106
C 0.555299 1.277928 0.580420
C 1.680937 0.316036 1.061532
I 2.661606 -0.698482 -0.723035
C 1.116032 2.198213 -0.519053
H 0.538436 3.126602 -0.556615
H 2.163343 2.478023 -0.338315
H 1.073938 1.733682 -1.512557
C 2.859793 0.999516 1.756763
H 3.240792 1.872288 1.217872
H 2.524470 1.326092 2.752004
H 3.683617 0.292883 1.904202
C -3.055337 -0.168948 1.251717
C -1.132967 -0.521693 -1.013536
H -3.596832 0.238841 2.123084
C -3.008653 -1.704602 1.425400
C -3.935165 0.203387 0.036691
H -0.266845 -0.460526 -1.695252
C -1.109488 -1.974374 -0.447106
C -2.366257 -0.399799 -1.933837
H -3.881803 -2.165764 0.942628
H -3.097343 -1.949088 2.494393
C -1.694765 -2.321908 0.943184
H -3.828373 1.275858 -0.161965
H -4.972984 0.027335 0.362885
C -3.715287 -0.601968 -1.245999
H -0.067015 -2.329070 -0.425889
H -1.601340 -2.627490 -1.189352
H -2.375605 0.566442 -2.441870
H -2.286697 -1.155957 -2.735550
H -0.966865 -2.052770 1.709289
H -4.517819 -0.352567 -1.957795
B -1.012898 0.726690 0.093810
H -3.846517 -1.671733 -1.024688
O -1.766728 0.384330 1.317322
H -1.787913 -3.418583 0.992664
C 0.261148 2.202073 1.790530
H 0.052216 1.639023 2.708136
H 1.087319 2.902433 1.988491
H -0.635157 2.785676 1.553602
C 1.218444 -0.842575 1.923703
H 2.068128 -1.325715 2.422373
H 0.524261 -0.460661 2.685394
H 0.696865 -1.589053 1.332431
O -1.685259 1.987769 -0.420972
C -1.599038 2.573458 -1.668040
H -2.596186 2.636850 -2.147977
H -1.224605 3.612296 -1.600132
H -0.936410 2.045482 -2.374705
48
G_b_conf_2 Eopt -1061.301943
C -0.463991 1.244738 0.720179

C	-1.727610	1.107286	-0.183786
I	-2.475449	-1.026094	-0.184952
C	-0.704656	0.634585	2.109844
H	0.089865	0.961427	2.793927
H	-1.657205	0.971264	2.548674
H	-0.716268	-0.457925	2.108710
C	-2.952606	1.886280	0.297433
H	-3.186043	1.710130	1.352973
H	-2.760763	2.959893	0.157782
H	-3.835043	1.630048	-0.299173
C	1.593302	-1.484642	-1.151650
C	2.202569	0.420907	1.076895
H	1.187751	-1.968653	-2.057439
C	1.698688	-2.586674	-0.067406
C	2.973637	-0.943568	-1.578909
H	2.168410	1.247812	1.809465
C	2.102898	-0.889841	1.911416
C	3.609850	0.505932	0.451257
H	2.698235	-3.045997	-0.082142
H	0.992169	-3.392509	-0.315675
C	1.333241	-2.087468	1.330384
H	2.803568	-0.035451	-2.173795
H	3.413235	-1.700950	-2.247352
C	3.981426	-0.655598	-0.467003
H	1.631805	-0.669851	2.879938
H	3.123291	-1.227720	2.166443
H	3.717147	1.435278	-0.117678
H	4.364436	0.551526	1.257471
H	0.268452	-1.834680	1.290445
H	4.960088	-0.444982	-0.925816
B	0.974280	0.692212	-0.024992
H	4.129496	-1.565912	0.132755
O	0.650502	-0.521322	-0.791022
H	1.427961	-2.927751	2.036761
C	-0.312297	2.758877	1.001258
H	-0.339198	3.367727	0.091324
H	-1.095840	3.126168	1.681143
H	0.649326	2.944314	1.496180
C	-1.483314	1.427692	-1.651719
H	-2.416941	1.351502	-2.223126
H	-1.117980	2.463036	-1.721496
H	-0.715401	0.778928	-2.076372
O	1.409318	1.697619	-1.070145
C	2.041392	2.905324	-0.853318
H	2.941380	2.994605	-1.492439
H	1.393322	3.765346	-1.111373
H	2.374415	3.061970	0.187371

48

G_b_conf_3 Eopt -1061.288408

C	0.395498	1.294708	0.030329
C	1.838291	1.009418	0.526247
I	2.722004	-0.809727	-0.508369
C	0.478562	1.815228	-1.414219
H	-0.521339	1.992566	-1.816758
H	1.037306	2.762800	-1.474987
H	0.960885	1.087457	-2.080063
C	2.893232	2.059403	0.159322
H	3.054692	2.146408	-0.918460
H	2.581642	3.040814	0.544550
H	3.851583	1.807351	0.629542
C	-2.564543	-0.583293	-1.590477
C	-2.045243	0.261795	1.230431
H	-2.483761	-0.923497	-2.637454
C	-3.570647	0.598192	-1.595380
C	-3.083511	-1.813833	-0.820457
H	-1.574693	0.574121	2.178821
C	-3.064109	1.377021	0.864743
C	-2.842209	-1.009932	1.601388
H	-4.591778	0.227821	-1.418830
H	-3.581909	1.038903	-2.603312
C	-3.225042	1.726729	-0.619884
H	-2.228331	-2.489933	-0.674546
H	-3.801167	-2.326135	-1.480642
C	-3.768858	-1.550126	0.516765
H	-2.798138	2.309691	1.381757
H	-4.056396	1.108309	1.266919
H	-2.165607	-1.819288	1.889752
H	-3.458293	-0.795124	2.493294
H	-2.289520	2.176844	-0.970748
H	-4.219568	-2.488907	0.874500
B	-0.796494	-0.022304	0.141491
H	-4.611820	-0.859779	0.364621
O	-1.284805	-0.157506	-1.243385
H	-3.994783	2.509615	-0.712364
C	-0.085027	2.501687	0.878171
H	-0.316130	2.236363	1.916261
H	0.645530	3.324337	0.897713
H	-0.995847	2.911792	0.431725
C	1.913784	0.769118	2.029002
H	2.841675	0.266282	2.321938
H	1.904242	1.751662	2.524106
H	1.058941	0.204326	2.401385
O	-0.173222	-1.344135	0.464561
C	0.135852	-1.881290	1.696581
H	-0.273458	-2.905694	1.784237
H	1.229119	-1.968361	1.843222
H	-0.258789	-1.309506	2.555167

48

G_b_conf_4 Eopt -1061.290122

C	-0.478317	1.172372	0.849550
C	-1.690083	1.207088	-0.119433
I	-2.607779	-0.854116	-0.429421
C	-0.985855	0.965426	2.287309
H	-0.133367	0.811260	2.957080
H	-1.552236	1.836947	2.651900
H	-1.637923	0.086032	2.373564
C	-2.913243	1.978493	0.382994
H	-3.334309	1.554422	1.299037
H	-2.627763	3.020564	0.584066
H	-3.696974	1.990449	-0.383552
C	3.367111	0.614437	0.668147
C	1.154605	-0.695357	-0.859442
H	3.984829	1.131485	1.423341
C	3.734433	1.250223	-0.698356
C	3.782072	-0.866098	0.781284
H	0.178541	-1.041172	-1.231296
C	1.791135	0.119634	-2.024965
C	1.992001	-1.987973	-0.695521
H	4.551712	0.684301	-1.171547
H	4.131953	2.259848	-0.516582
C	2.556092	1.394357	-1.663974
H	3.307986	-1.267988	1.687204
H	4.871741	-0.876258	0.944303
C	3.477173	-1.771491	-0.409538
H	1.022913	0.401542	-2.758198
H	2.469983	-0.537142	-2.594276
H	1.584391	-2.615551	0.102260
H	1.914650	-2.581783	-1.624055
H	1.850357	2.106996	-1.223254
H	3.947383	-2.751433	-0.232695
B	0.893909	0.066175	0.623787
H	3.975017	-1.367354	-1.303625
O	2.047760	0.897806	1.015770
H	2.925169	1.858317	-2.592693
C	0.070956	2.626336	0.842826
H	0.602682	2.887960	-0.075700
H	-0.721248	3.373204	0.999688
H	0.788465	2.723890	1.662061
C	-1.340275	1.680006	-1.520610
H	-2.174635	1.536553	-2.215557
H	-1.124412	2.757590	-1.477943
H	-0.458722	1.169190	-1.909390
O	0.827559	-0.987605	1.694236
C	-0.029743	-2.069242	1.731504
H	0.462615	-2.909312	2.256139
H	-0.966423	-1.857653	2.281630
H	-0.330553	-2.438515	0.737769

48

G_b_conf_5 Eopt -1061.290537

C	-0.456016	1.592805	0.007103

C -1.719956 0.934347 0.634379
I -2.348820 -0.980601 -0.433113
C -0.144621 2.801442 0.926357
H 0.632102 3.420656 0.471033
H -1.014026 3.458704 1.078750
H 0.219726 2.497223 1.914833
C -1.565969 0.573379 2.106289
H -0.592908 0.137367 2.330041
H -1.662152 1.497855 2.694403
H -2.353810 -0.109629 2.441189
C 1.533009 -1.620722 -1.029455
C 2.028392 0.424273 1.102690
H 1.212710 -2.165898 -1.934163
C 1.298799 -2.584218 0.162706
C 3.027856 -1.318458 -1.261934
H 1.998106 1.324598 1.737913
C 1.685684 -0.772510 2.034692
C 3.515429 0.286343 0.698869
H 2.202187 -3.187848 0.338731
H 0.510385 -3.297988 -0.119146
C 0.832867 -1.903179 1.450634
H 3.089857 -0.486614 -1.976738
H 3.456369 -2.208196 -1.750423
C 3.873636 -1.008713 -0.027671
H 1.162829 -0.410504 2.932624
H 2.622269 -1.204094 2.426910
H 3.825021 1.126204 0.069386
H 4.137957 0.338970 1.610434
H -0.164889 -1.505858 1.243303
H 4.931224 -0.961686 -0.330885
B 1.041240 0.714446 -0.231816
H 3.808774 -1.854856 0.672943
O 0.700593 -0.502729 -0.960428
H 0.701777 -2.678236 2.222983
C -0.847545 2.157114 -1.369712
H -1.233586 1.361922 -2.023460
H -1.616413 2.941825 -1.297766
H 0.024628 2.588338 -1.866654
C -3.015213 1.742155 0.484550
H -3.826263 1.254581 1.038526
H -2.871797 2.743277 0.915342
H -3.333457 1.855886 -0.554892
O 1.857013 1.534618 -1.209142
C 2.456335 2.748777 -0.946616
H 3.407980 2.827649 -1.503997
H 1.835421 3.610504 -1.268187
H 2.694266 2.913322 0.120302
48
G_b_conf_6 Eopt -1061.290950
C -0.431054 1.402819 0.097813
C -1.896829 1.055936 0.464506
I -2.549567 -0.906714 -0.516969
C -0.098206 2.645516 0.963899
H 0.820864 3.099215 0.580626
H -0.889682 3.409946 0.939981
H 0.095101 2.370801 2.007316
C -2.150064 0.889683 1.957620
H -1.334221 0.355512 2.445895
H -2.208069 1.898117 2.394718
H -3.108884 0.396789 2.151261
C 3.314222 0.616028 0.810628
C 1.245557 -0.734841 -0.858974
H 3.831393 1.182912 1.604656
C 4.017509 0.971811 -0.523331
C 3.518421 -0.865158 1.194854
H 0.308815 -1.052224 -1.342732
C 2.086148 -0.070180 -1.995240
C 1.948727 -2.051699 -0.463048
H 4.812321 0.245748 -0.747814
H 4.521436 1.943329 -0.412974
C 3.034480 1.103356 -1.686281
H 2.830398 -1.096961 2.019374
H 4.543963 -0.941896 1.589807
C 3.367845 -1.896507 0.077656
H 1.406554 0.291713 -2.780204
H 2.681927 -0.859782 -2.486954
H 1.362297 -2.598379 0.279001
H 1.996622 -2.710096 -1.348881
H 2.439492 1.998098 -1.475897
H 3.715270 -2.873096 0.449182
B 0.836755 0.247369 0.437866
H 4.048915 -1.639015 -0.746713
O 1.999439 1.085411 0.812099
H 3.603917 1.323753 -2.603412
C -0.392429 1.862168 -1.365027
H -0.760154 1.082147 -2.048154
H -0.994535 2.768586 -1.533068
H 0.628239 2.110986 -1.658656
C -2.961740 1.997827 -0.104917
H -3.951400 1.719948 0.276938
H -2.748495 3.022993 0.231194
H -3.002232 1.995040 -1.197346
O 0.511324 -0.513181 1.693914
C -0.095667 -1.739863 1.880038
H 0.585483 -2.439513 2.404718
H -0.999608 -1.651590 2.514275
H -0.420087 -2.233335 0.954640
48
G_b_conf_7 Eopt -1061.294148
C 0.488223 0.891929 1.038792
C 1.796687 1.038334 0.208834
I 2.612315 -0.996031 -0.417572
C 0.315984 2.242844 1.783917
H -0.687763 2.258550 2.222549
H 1.048938 2.362433 2.596666
H 0.390703 3.104963 1.111092
C 1.628963 1.769146 -1.111000
H 0.965341 1.231188 -1.790210
H 1.178473 2.750600 -0.905813
H 2.598762 1.919607 -1.600685
C -3.262129 0.002624 1.058282
C -1.073080 -0.590214 -0.877155
H -3.916575 0.468899 1.815606
C -3.263747 -1.520718 1.343700
C -3.923131 0.347365 -0.294916
H -0.126031 -0.602365 -1.440267
C -1.169422 -1.993790 -0.212626
C -2.156519 -0.491596 -1.970214
H -4.042602 -2.024566 0.753229
H -3.537255 -1.682243 2.397028
C -1.892571 -2.166100 1.136756
H -3.665079 1.388691 -0.534440
H -5.011642 0.312038 -0.130329
C -3.599113 -0.565400 -1.477313
H -0.151067 -2.380869 -0.055124
H -1.626682 -2.688648 -0.940135
H -2.033842 0.426756 -2.548524
H -2.004781 -1.315011 -2.690948
H -1.247556 -1.766470 1.922985
H -4.274918 -0.319150 -2.311134
B -0.992445 0.695056 0.182441
H -3.837720 -1.604329 -1.206201
O -1.994386 0.544855 1.270118
H -1.983472 -3.246722 1.332029
C 0.663025 -0.152327 2.148554
H 0.703891 -1.176160 1.760288
H 1.581701 0.012020 2.734624
H -0.188419 -0.084171 2.835887
C 2.979211 1.637890 0.968994
H 3.899730 1.566706 0.379756
H 2.775908 2.705021 1.137669
H 3.147516 1.159590 1.939632
O -1.272304 2.044870 -0.438046
C -1.377614 2.513194 -1.725678
H -2.419708 2.487487 -2.107831
H -1.061612 3.572976 -1.759466
H -0.759793 1.980617 -2.468926
59
H_b_conf_1 Eopt -3396.645961
C -2.302737 2.288838 0.404773

C -2.224016 0.917646 1.100541
C -4.150652 0.074193 -1.015674
C -3.884660 1.522759 -1.469792
H -1.449949 2.426399 -0.271787
H -4.784193 1.852492 -2.013607
C -3.455737 0.714765 2.026087
H -3.895064 1.695509 2.281641
C -5.169810 0.011627 0.149804
H -5.865295 -0.818860 -0.041998
H -1.364872 0.984869 1.785510
H -4.646646 -0.396769 -1.882060
C -4.566724 -0.224916 1.535475
H -4.163387 -1.242033 1.545590
H -5.387288 -0.208252 2.270588
C -3.584909 2.548498 -0.381065
H -3.525345 3.545596 -0.843751
H -4.436539 2.595591 0.313917
H -3.109559 0.292697 2.980413
H -5.784637 0.924890 0.143172
H -2.211359 3.085452 1.164947
H -3.066495 1.501533 -2.203015
B -1.929322 -0.427713 0.182669
C -0.460187 -0.498398 -0.741386
C 0.826013 -0.156615 0.054614
C 1.072728 1.336692 0.240628
H 1.158438 1.871962 -0.711601
H 0.244556 1.769958 0.811754
C 0.935257 -0.847568 1.409595
H 1.918732 -0.656282 1.858235
H 0.776563 -1.927916 1.338265
O -3.004638 -0.694622 -0.800881
C -0.568288 0.362986 -2.007743
H 0.336082 0.285638 -2.633188
H -0.736392 1.426393 -1.806749
H 0.163575 -0.450665 2.079835
C -0.404234 -1.956008 -1.235686
H -0.081102 -2.651400 -0.453266
H -1.400213 -2.267289 -1.574640
H 0.280276 -2.071140 -2.092771
H 1.989419 1.511196 0.816482
H -1.417348 0.005715 -2.602779
Se 2.442636 -0.867469 -1.052795
C 3.893690 -0.009255 -0.136965
C 4.507383 -0.625118 0.961541
C 4.374263 1.231566 -0.577500
C 5.568627 -0.001398 1.619462
H 4.150819 -1.598347 1.299642
C 5.435764 1.852269 0.080067
H 3.906431 1.712498 -1.436849
C 6.035109 1.237936 1.180954
H 6.034955 -0.490601 2.475217
H 5.796853 2.819521 -0.271031
H 6.865586 1.723371 1.694027
O -1.864162 -1.546865 1.188450
C -2.393217 -2.804555 0.969479
H -2.975973 -3.137305 1.849972
H -3.066717 -2.835899 0.096591
H -1.608475 -3.569770 0.811192
59
H_b_conf_10 Eopt -3396.644529
C -3.271551 -1.636512 -0.790502
C -2.605033 -0.303609 -1.177918
C -3.690888 0.645840 1.433478
C -4.114768 -0.834456 1.499605
H -2.533871 -2.323375 -0.356677
H -5.008272 -0.878532 2.142511
C -3.664436 0.677751 -1.751462
H -4.541675 0.110399 -2.110405
C -4.653616 1.484841 0.555837
H -4.842718 2.441113 1.065704
H -1.950143 -0.540921 -2.030568
H -3.820116 1.013227 2.466313
C -4.138471 1.822614 -0.844394
H -3.298695 2.513756 -0.724036
H -4.928520 2.380862 -1.371796
C -4.444712 -1.522522 0.178760
H -4.829986 -2.531094 0.393838
H -5.272741 -0.988011 -0.310248
H -3.244360 1.161603 -2.644813
H -5.628854 0.976925 0.500424
H -3.641761 -2.134721 -1.704583
H -3.323774 -1.387594 2.025176
B -1.618936 0.446331 -0.079708
C -0.216171 -0.402977 0.498181
C 0.675062 -1.020498 -0.606369
C 0.150321 -2.347436 -1.160171
H 0.093009 -3.130235 -0.393329
H -0.853038 -2.200286 -1.572468
C 0.978578 -0.083404 -1.767457
H 1.671369 -0.557704 -2.473565
H 1.397101 0.873782 -1.440515
O -2.340543 0.857419 1.147453
C -0.606429 -1.459658 1.539637
H -1.274645 -2.238167 1.155479
H -1.126564 -0.962172 2.367061
H 0.041205 0.144306 -2.286711
C 0.571802 0.664765 1.273755
H 1.295498 0.198550 1.959229
H 1.125430 1.341817 0.613252
H -0.118842 1.268241 1.875839
H 0.797558 -2.709502 -1.968179
H 0.283964 -1.961207 1.954915
Se 2.512284 -1.663637 0.131669
C 3.639068 -0.105248 0.108893
C 4.164652 0.382576 -1.094371
C 4.009368 0.513006 1.310376
C 5.011966 1.491384 -1.098466
H 3.911359 -0.109807 -2.032320
C 4.867966 1.611640 1.305995
H 3.613418 0.135243 2.252765
C 5.366285 2.109048 0.100824
H 5.403238 1.866135 -2.044798
H 5.144773 2.082967 2.249576
H 6.031828 2.972606 0.097304
O -1.120924 1.663599 -0.813589
C -0.947642 2.899102 -0.219621
H 0.120856 3.152628 -0.077907
H -1.372793 3.696833 -0.858744
H -1.438569 2.968715 0.765492
59
H_b_conf_11 Eopt -3396.641691
C -2.983741 0.893885 1.995494
C -3.046380 -0.230786 0.944321
C -2.353198 1.927860 -0.997385
C -2.292735 2.747951 0.305969
H -2.043419 0.847235 2.558316
H -2.569478 3.781233 0.040931
C -4.416590 -0.161851 0.209782
H -5.116597 0.464522 0.789126
C -3.807319 1.718804 -1.496016
H -3.826585 1.883790 -2.583314
H -3.071398 -1.157414 1.538430
H -1.870178 2.581652 -1.744622
C -4.399329 0.331522 -1.241578
H -3.822729 -0.385643 -1.837667
H -5.430837 0.323974 -1.628777
C -3.195461 2.310673 1.457950
H -3.078681 3.026488 2.286308
H -4.241863 2.410961 1.132060
H -4.875164 -1.160606 0.201439
H -4.454558 2.497638 -1.061954
H -3.776251 0.716433 2.744831
H -1.242624 2.783630 0.624545
B -1.774895 -0.415951 -0.119424
C -0.255344 -0.736767 0.652651
C 0.752908 -1.418022 -0.317558
C 0.535499 -2.923521 -0.488140
H 1.202562 -3.321722 -1.262858
H 0.722907 -3.482557 0.437703
C 0.827309 -0.754562 -1.685075

H -0.151835 -0.872344 -2.162848
H 1.596291 -1.230973 -2.306207
O -1.581938 0.766385 -0.988879
C -0.477274 -1.630216 1.880865
H -1.053552 -2.535255 1.642687
H -1.033923 -1.082796 2.652270
H 1.032500 0.318710 -1.620217
C 0.337612 0.587038 1.147393
H 0.699448 1.208203 0.318648
H -0.408065 1.175354 1.685154
H 1.174358 0.410673 1.839159
H -0.498295 -3.093649 -0.795868
H 0.476452 -1.947743 2.335357
Se 2.690606 -1.466037 0.439751
C 3.453647 0.249195 0.021845
C 3.707306 1.175501 1.041932
C 3.831715 0.565202 -1.289262
C 4.305704 2.401213 0.753120
H 3.424941 0.935961 2.066938
C 4.416258 1.798857 -1.579410
H 3.670004 -0.159287 -2.086285
C 4.655325 2.720222 -0.560044
H 4.495142 3.111401 1.558685
H 4.694150 2.034039 -2.607276
H 5.116485 3.681804 -0.786686
O -2.087060 -1.561432 -1.052576
C -2.873793 -2.654761 -0.750078
H -2.447184 -3.572441 -1.195525
H -2.982480 -2.847984 0.331059
H -3.896419 -2.562498 -1.166973
59
H_b_conf_12 Eopt -3396.642638
C -3.688108 -0.091240 1.884765
C -3.268364 -0.846904 0.604329
C -2.878403 1.853741 -0.519780
C -3.158559 2.279450 0.932812
H -2.893491 -0.175530 2.631928
H -3.592502 3.291777 0.913326
C -4.452931 -0.973626 -0.372818
H -5.278362 -1.524386 0.113969
C -4.113161 1.463956 -1.358147
H -3.725912 1.238210 -2.360664
H -3.086687 -1.882455 0.944836
H -2.508381 2.772083 -1.011702
C -5.060256 0.333167 -0.914705
H -5.685271 0.081207 -1.785963
H -5.765253 0.730188 -0.175687
C -4.068651 1.395051 1.784972
H -4.078805 1.809694 2.805184
H -5.098881 1.503071 1.428478
H -4.124960 -1.600139 -1.210883
H -4.722461 2.375298 -1.472079
H -4.556274 -0.613428 2.328371
H -2.172539 2.374689 1.414416
B -1.868013 -0.458766 -0.225862
C -0.426792 -0.903019 0.607571
C 0.818938 -0.215061 -0.019193
C 0.870244 -0.267375 -1.541027
H 0.695736 -1.277485 -1.924673
H 0.082235 0.389277 -1.928897
C 1.005215 1.229586 0.436403
H 1.820175 1.712055 -0.117584
H 1.225809 1.309095 1.506755
O -1.813651 0.955258 -0.615404
C -0.305081 -2.432386 0.507413
H 0.429109 -2.834371 1.225763
H -0.021367 -2.756964 -0.499329
H 0.072007 1.762390 0.220382
C -0.494687 -0.558638 2.100746
H 0.483130 -0.686958 2.592632
H -0.827801 0.471933 2.282884
H -1.197190 -1.231612 2.607737
H 1.838313 0.098098 -1.906755
H -1.267521 -2.906825 0.737670
Se 2.502091 -1.257811 0.627955
C 3.869982 -0.019845 0.101552
C 4.323309 0.960240 0.994879
C 4.447084 -0.097133 -1.172780
C 5.319976 1.857992 0.613104
H 3.886470 1.020037 1.991919
C 5.443088 0.803356 -1.553782
H 4.112324 -0.867711 -1.867522
C 5.881786 1.782637 -0.662511
H 5.660136 2.618289 1.317002
H 5.880521 0.733949 -2.550252
H 6.661490 2.484252 -0.959924
O -1.841953 -1.342200 -1.452028
C -2.099787 -0.884291 -2.725980
H -3.171432 -0.962618 -3.007343
H -1.810995 0.169738 -2.872067
H -1.536645 -1.487874 -3.460564
59
H_b_conf_13 Eopt -3396.641789
C -2.377546 1.463103 1.674483
C -2.457347 -0.005608 1.218060
C -3.462605 1.196357 -1.290611
C -3.442009 2.504979 -0.478162
H -1.364589 1.842962 1.493050
H -4.322600 3.102787 -0.765892
C -3.872717 -0.602975 1.361431
H -4.217120 -0.491626 2.406289
C -4.638981 0.243767 -1.001690
H -4.427385 -0.684907 -1.548014
H -1.880521 -0.570886 1.965599
H -3.627352 1.526121 -2.333497
C -4.999388 -0.080282 0.451246
H -5.797540 -0.839021 0.429706
H -5.469736 0.803628 0.897545
C -3.377372 2.447137 1.054464
H -3.121941 3.457674 1.409759
H -4.373908 2.248161 1.463690
H -3.770265 -1.681101 1.182778
H -5.534291 0.681455 -1.471338
H -2.520895 1.517089 2.768963
H -2.561686 3.054755 -0.846496
B -1.755519 -0.342403 -0.249548
C -0.020146 -0.234291 -0.358146
C 0.731425 -0.975475 0.775280
C 0.861634 -0.159332 2.059041
H 1.288475 -0.769976 2.864505
H 1.496065 0.722648 1.922312
C 0.161295 -2.361756 1.084720
H -0.851268 -2.257830 1.487491
H 0.782783 -2.882705 1.825445
O -2.210147 0.580271 -1.302148
C 0.428048 1.235075 -0.451506
H 0.225195 1.818700 0.452413
H -0.115520 1.711959 -1.275403
H 0.080543 -2.981474 0.184850
C 0.306797 -0.852680 -1.726358
H 0.233649 -1.946343 -1.717378
H -0.392867 -0.470075 -2.479497
H 1.325494 -0.591257 -2.050065
H -0.124690 0.193817 2.381577
H 1.503737 1.324824 -0.660958
Se 2.673678 -1.553229 0.305070
C 3.639221 0.071408 -0.038728
C 4.032325 0.910386 1.011548
C 3.984505 0.415598 -1.352383
C 4.714354 2.098625 0.746604
H 3.803113 0.631595 2.039675
C 4.678531 1.596122 -1.613059
H 3.690376 -0.237546 -2.173922
C 5.034918 2.447182 -0.565479
H 5.002535 2.750033 1.572271
H 4.934226 1.855769 -2.640774
H 5.567899 3.375766 -0.771060
O -2.165197 -1.755693 -0.559246
C -2.430397 -2.220553 -1.832436
H -1.673271 -2.951361 -2.177178

H -3.404682 -2.746561 -1.858280
H -2.473921 -1.412628 -2.581573
59
H_b_conf_14 Eopt -3396.643884
C -3.736954 -1.542160 -0.953931
C -3.107355 -0.169171 -1.243280
C -3.362408 0.335672 1.655500
C -3.856814 -1.123408 1.663435
H -2.964943 -2.314779 -1.063290
H -4.615990 -1.208475 2.457717
C -4.172622 0.934749 -1.388670
H -4.843505 0.688513 -2.232081
C -4.385118 1.386589 1.182423
H -3.861239 2.352596 1.213760
H -2.686161 -0.255885 -2.258941
H -3.214520 0.571765 2.725504
C -5.071852 1.224334 -0.178995
H -5.626387 2.154751 -0.380023
H -5.843305 0.449179 -0.102629
C -4.460247 -1.744713 0.393201
H -4.548842 -2.827787 0.572026
H -5.494493 -1.395796 0.297943
H -3.651026 1.856749 -1.677624
H -5.173680 1.443963 1.950082
H -4.476158 -1.772338 -1.743565
H -2.998717 -1.720618 2.001718
B -1.802549 0.347228 -0.344795
C -0.427165 -0.684293 -0.504778
C 0.894899 0.056156 -0.147232
C 1.430116 0.946927 -1.264658
H 1.743279 0.374056 -2.144939
H 0.623653 1.629826 -1.553598
C 0.825743 0.857905 1.146543
H 0.428548 0.268137 1.978083
H 0.155234 1.711032 0.994187
O -2.094302 0.478649 1.088114
C -0.335839 -1.224962 -1.939083
H -0.399492 -0.421458 -2.685180
H -1.160127 -1.921004 -2.138081
H 1.817854 1.241185 1.417415
C -0.624056 -1.878425 0.441741
H -1.636203 -2.282754 0.357918
H 0.067818 -2.706372 0.212217
H -0.483287 -1.592906 1.490902
H 2.289124 1.534754 -0.917773
H 0.602858 -1.775957 -2.114253
Se 2.357705 -1.393179 0.182179
C 3.922104 -0.282577 0.169276
C 4.634444 -0.071344 -1.019037
C 4.382067 0.310852 1.352018
C 5.771648 0.735909 -1.027376
H 4.288810 -0.538096 -1.941598
C 5.519111 1.120128 1.341381
H 3.844315 0.136785 2.284240
C 6.216104 1.335176 0.152347
H 6.313408 0.895360 -1.960290
H 5.863230 1.579137 2.268759
H 7.105326 1.965978 0.144984
O -1.423624 1.682365 -0.936695
C -1.627407 2.887484 -0.295985
H -0.804786 3.586999 -0.536717
H -2.568225 3.387029 -0.608259
H -1.668589 2.785306 0.800896
59
H_b_conf_15 Eopt -3396.644882
C -3.546659 0.589002 1.804811
C -3.266319 -0.556090 0.786809
C -2.892815 1.780884 -1.042997
C -3.354327 2.639305 0.161701
H -3.136504 0.312843 2.786299
H -4.427546 2.866918 0.082845
C -4.552023 -0.827426 -0.020926
H -5.350107 -1.159503 0.667451
C -4.063487 1.041525 -1.722947
H -3.634637 0.303349 -2.414272
H -3.116085 -1.466610 1.393692
H -2.521712 2.482859 -1.809637
C -5.084935 0.366563 -0.808434
H -5.938519 0.035569 -1.420205
H -5.496250 1.111895 -0.111557
C -3.018951 2.002899 1.510306
H -1.926100 1.992758 1.582258
H -3.374978 2.671515 2.310237
H -4.393273 -1.653545 -0.721583
H -4.595251 1.793349 -2.327490
H -4.637064 0.659818 1.966450
H -2.837462 3.609617 0.122799
B -1.910292 -0.382960 -0.177741
C -0.461699 -0.790003 0.681849
C 0.789774 -0.196203 -0.019286
C 0.814027 -0.416500 -1.527294
H 1.770165 -0.083103 -1.950544
H 0.653822 -1.467451 -1.791639
C 1.008459 1.282022 0.288815
H 1.823147 1.693258 -0.320150
H 1.248721 1.457885 1.343649
O -1.790653 0.994115 -0.696097
C -0.364350 -2.322574 0.736033
H 0.387953 -2.651577 1.471541
H -0.102198 -2.765563 -0.230417
H 0.081167 1.808589 0.035951
C -0.506895 -0.340301 2.150184
H -1.293511 -0.890422 2.682292
H 0.443006 -0.561388 2.663931
H -0.704686 0.727606 2.276627
H -0.000903 0.168259 -1.969661
H -1.318386 -2.758902 1.058568
Se 2.470373 -1.195528 0.711111
C 3.845418 -0.030208 0.054107
C 4.397710 -0.227809 -1.218140
C 4.326677 1.019246 0.848760
C 5.396512 0.622349 -1.695012
H 4.041394 -1.051913 -1.836404
C 5.326214 1.866476 0.371276
H 3.908631 1.173423 1.843684
C 5.862947 1.671231 -0.902389
H 5.814236 0.459174 -2.689017
H 5.687885 2.681809 0.998746
H 6.644573 2.333787 -1.274884
O -2.013903 -1.239405 -1.411661
C -2.367969 -2.572107 -1.483058
H -3.155951 -2.722553 -2.246227
H -1.516493 -3.210674 -1.787026
H -2.755711 -2.989693 -0.538292
59
H_b_conf_16 Eopt -3396.642272
C -3.684341 0.320040 1.832898
C -3.330616 -0.615724 0.656098
C -2.758059 1.937545 -0.781839
C -3.053032 2.495555 0.623969
H -2.863519 0.374307 2.553302
H -3.400777 3.532892 0.495604
C -4.543733 -0.662192 -0.318505
H -5.451266 -0.321228 0.209281
C -4.048910 1.599047 -1.570506
H -3.917929 1.941381 -2.607444
H -3.271349 -1.616650 1.117193
H -2.287050 2.780840 -1.317196
C -4.413983 0.112972 -1.638436
H -3.643380 -0.381271 -2.240947
H -5.364354 0.023038 -2.188513
C -4.083851 1.744505 1.458414
H -4.274274 2.312559 2.382028
H -5.041803 1.731828 0.917682
H -4.752176 -1.705862 -0.593349
H -4.886292 2.188171 -1.165169
H -4.533525 -0.123887 2.382937
H -2.094573 2.550145 1.163987
B -1.902884 -0.415559 -0.185420
C -0.460708 -0.735042 0.722388

C	0.792865	-0.193769	-0.023142
C	0.843058	-0.519862	-1.511007
H	0.686521	-1.585571	-1.706044
H	0.038396	0.032168	-2.010534
C	1.010746	1.304242	0.170924
H	1.237749	1.566707	1.210413
H	0.087500	1.808748	-0.135786
O	-1.800735	0.924826	-0.801372
C	-0.369607	-2.259299	0.891436
H	-0.130798	-2.768021	-0.049840
H	-1.325639	-2.660879	1.253160
H	1.833411	1.664522	-0.459171
C	-0.498436	-0.142146	2.136993
H	0.483836	-0.213762	2.631618
H	-0.801308	0.912610	2.148620
H	-1.208084	-0.701007	2.759499
H	1.807331	-0.212788	-1.936141
H	0.389171	-2.547246	1.637772
Se	2.468528	-1.135658	0.794493
C	3.851142	-0.014080	0.080150
C	4.424010	-0.295273	-1.167043
C	4.318712	1.087199	0.810167
C	5.430085	0.522765	-1.683446
H	4.078229	-1.159427	-1.734505
C	5.325332	1.902314	0.293389
H	3.884987	1.306867	1.785881
C	5.882874	1.623123	-0.955454
H	5.864177	0.294072	-2.657337
H	5.676243	2.758401	0.870653
H	6.670109	2.260527	-1.358765
O	-1.875163	-1.381997	-1.341381
C	-2.423942	-2.645568	-1.375686
H	-1.676468	-3.393312	-1.703779
H	-2.818085	-2.996217	-0.407391
H	-3.260975	-2.703238	-2.101938

59

H_b_conf_17 Eopt -3396.644820

C	-2.398415	2.247697	-0.282968
C	-2.163486	1.141211	0.762257
C	-4.231838	-0.280686	-0.802653
C	-4.364615	1.066052	-1.539091
H	-1.673616	2.134452	-1.098885
H	-5.431718	1.232235	-1.761224
C	-3.203315	1.159629	1.901977
H	-3.219892	2.160730	2.371158
C	-4.898647	-0.354298	0.584675
H	-4.593818	-1.312418	1.025388
H	-1.234729	1.423993	1.280123
H	-4.812001	-0.988686	-1.423499
C	-4.662333	0.777890	1.589045
H	-5.145725	0.483509	2.533963
H	-5.222197	1.659230	1.254941
C	-3.799647	2.341583	-0.898595
H	-3.775105	3.117084	-1.680075
H	-4.496209	2.723545	-0.144286
H	-2.831221	0.466106	2.667058
H	-5.985666	-0.422395	0.419028
H	-2.185302	3.233654	0.168132
H	-3.868081	0.911196	-2.509881
B	-1.898870	-0.377816	0.148681
C	-0.410632	-0.650516	-0.706607
C	0.853714	-0.147472	0.035322
C	1.125617	1.343743	-0.154944
H	0.223313	1.915425	0.086232
H	1.926628	1.684899	0.512161
C	0.883870	-0.495002	1.520222
H	0.099217	0.064524	2.043744
H	1.853235	-0.225800	1.958856
O	-2.920920	-0.760316	-0.839626
C	-0.468572	-0.072359	-2.129106
H	-0.548557	1.019029	-2.159945
H	-1.348284	-0.479391	-2.640898
H	0.692306	-1.558457	1.695168
C	-0.354203	-2.178016	-0.885490
H	-1.332443	-2.546176	-1.217623
H	0.377971	-2.471796	-1.655980
H	-0.092855	-2.699270	0.042447
H	1.412825	1.585775	-1.184111
H	0.424028	-0.348921	-2.714927
Se	2.498670	-1.126043	-0.782226
C	3.911298	-0.031423	-0.083303
C	4.404974	1.048113	-0.828400
C	4.481700	-0.313746	1.164695
C	5.435196	1.841906	-0.325114
H	3.973631	1.267181	-1.805271
C	5.511378	0.483166	1.667557
H	4.116984	-1.163129	1.742532
C	5.989958	1.562699	0.924977
H	5.806983	2.681040	-0.914066
H	5.943993	0.253850	2.641940
H	6.795821	2.183317	1.317514
O	-1.934620	-1.289171	1.344011
C	-2.429899	-2.578306	1.317746
H	-2.950884	-2.816602	0.375509
H	-1.634210	-3.335667	1.456728
H	-3.153153	-2.732802	2.142010

59

H_b_conf_2 Eopt -3396.643053

C	-3.762992	-1.547481	-1.011359
C	-3.136593	-0.166869	-1.266104
C	-3.403423	0.236956	1.672678
C	-3.861554	-1.230934	1.563755
H	-2.997872	-2.333785	-1.036538
H	-4.558912	-1.397619	2.400320
C	-4.259598	0.905626	-1.323602
H	-5.225807	0.415166	-1.538456
C	-4.534496	1.220509	1.281548
H	-4.531353	2.055020	1.998482
H	-2.732191	-0.213781	-2.288973
H	-3.230915	0.386550	2.752410
C	-4.429300	1.835939	-0.114915
H	-3.580034	2.522832	-0.102746
H	-5.322974	2.459694	-0.277638
C	-4.573818	-1.657286	0.280158
H	-4.921777	-2.694792	0.400884
H	-5.487811	-1.053037	0.173612
H	-4.070646	1.565210	-2.182610
H	-5.505877	0.718975	1.412256
H	-4.447911	-1.786748	-1.844924
H	-2.991986	-1.871092	1.758848
B	-1.843557	0.349523	-0.352484
C	-0.459743	-0.698267	-0.485926
C	0.861203	0.021106	-0.097246
C	1.387560	0.956271	-1.183288
H	0.561714	1.593512	-1.512596
H	2.203561	1.583669	-0.804209
C	0.802753	0.735745	1.247845
H	1.777998	1.176430	1.492454
H	0.508375	0.061050	2.058031
O	-2.170546	0.521365	1.075286
C	-0.358009	-1.204981	-1.932406
H	-0.409324	-0.382081	-2.657914
H	-1.186331	-1.889073	-2.155277
H	0.059252	1.535796	1.205994
C	-0.659855	-1.914921	0.427912
H	-0.616036	-1.643060	1.489485
H	-1.631782	-2.381197	0.256850
H	0.092677	-2.699251	0.240963
H	1.760456	0.404708	-2.054151
H	0.577889	-1.758806	-2.113010
Se	2.347656	-1.432052	0.151613
C	3.894203	-0.296767	0.156068
C	4.588677	-0.035222	-1.032780
C	4.358338	0.266388	1.352042
C	5.710979	0.792810	-1.028292
H	4.240817	-0.478877	-1.965805
C	5.480170	1.096520	1.354301
H	3.835118	0.052582	2.284268
C	6.158654	1.362481	0.164751
H	6.238548	0.991761	-1.961747

H 5.827343 1.531642 2.292009
H 7.036055 2.009655 0.167341
O -1.504969 1.680111 -0.987720
C -1.276587 2.856758 -0.301096
H -1.617237 2.811267 0.747551
H -0.203623 3.136286 -0.284004
H -1.807211 3.695469 -0.788367
59
H_b_conf_3 Eopt -3396.646902
C -2.575796 2.298043 -0.160422
C -2.168351 1.160907 0.812683
C -4.302346 -0.313124 -0.665127
C -4.568760 1.045300 -1.361929
H -1.669609 2.834371 -0.486700
H -5.404383 1.564793 -0.870146
C -3.133337 1.109246 2.011845
H -3.068529 2.057910 2.574496
C -4.863889 -0.338574 0.772750
H -4.502236 -1.247199 1.271884
H -1.219605 1.477466 1.267692
H -4.895648 -1.070012 -1.207404
C -4.597829 0.881303 1.653233
H -5.184817 0.778379 2.579261
H -4.992940 1.779198 1.153761
C -3.332967 1.941328 -1.451033
H -2.636619 1.438530 -2.126814
H -3.622223 2.881375 -1.947520
H -2.801438 0.310864 2.688381
H -5.955327 -0.448061 0.670038
H -3.163013 3.052064 0.394168
H -4.900226 0.853475 -2.393180
B -1.906075 -0.343921 0.144334
C -0.434688 -0.560051 -0.740647
C 0.840853 -0.122199 0.031174
C 1.145078 1.371621 -0.080273
H 1.943371 1.661180 0.613888
H 1.451115 1.660369 -1.091616
C 0.866858 -0.544991 1.495569
H 1.844732 -0.323505 1.941793
H 0.645109 -1.609842 1.617571
O -2.969537 -0.711385 -0.817671
C -0.488484 0.152524 -2.097392
H -1.344591 -0.225126 -2.669176
H 0.422861 -0.033786 -2.689997
H 0.099025 0.010347 2.048505
C -0.388678 -2.067108 -1.046302
H -1.376328 -2.404247 -1.383593
H 0.325553 -2.299867 -1.853537
H -0.108460 -2.660927 -0.168522
H 0.252182 1.951311 0.176778
H -0.602555 1.239254 -2.003293
Se 2.465766 -1.085841 -0.841000
C 3.896945 -0.053607 -0.085960
C 4.413787 1.050970 -0.776345
C 4.456491 -0.406553 1.148948
C 5.455703 1.800774 -0.231276
H 3.991349 1.324590 -1.743306
C 5.497397 0.346763 1.694061
H 4.073436 -1.276191 1.683151
C 5.998655 1.452086 1.006397
H 5.845561 2.660274 -0.777608
H 5.920862 0.063211 2.658126
H 6.813285 2.038581 1.431909
O -1.901409 -1.301635 1.301849
C -2.376463 -2.596375 1.230797
H -3.059576 -2.804324 2.077199
H -2.935200 -2.795657 0.301107
H -1.564289 -3.346146 1.297106
59
H_b_conf_4 Eopt -3396.645374
C -3.243295 -1.320828 -1.588461
C -2.398606 -0.030343 -1.356667
C -4.250541 0.183374 0.964840
C -4.982966 -1.045325 0.372832
H -2.577259 -2.120509 -1.945491
H -5.849779 -0.720613 -0.221641
C -3.139013 1.184039 -1.944008
H -3.255836 1.059210 -3.035709
C -4.594720 1.467762 0.174617
H -3.977857 2.296208 0.540809
H -1.516719 -0.158313 -1.996766
H -4.676055 0.359734 1.968835
C -4.528744 1.410302 -1.352680
H -4.944838 2.348796 -1.751119
H -5.203078 0.616927 -1.710000
C -4.058376 -1.946271 -0.442759
H -3.370286 -2.407389 0.272720
H -4.655753 -2.770099 -0.865069
H -2.528053 2.082428 -1.785521
H -5.634799 1.704758 0.451014
H -3.932928 -1.137317 -2.431894
H -5.390642 -1.651143 1.195471
B -1.855239 0.276150 0.197320
C -0.432917 -0.595550 0.627470
C 0.880876 0.057605 0.095182
C 0.857259 0.410652 -1.387388
H 1.834048 0.797173 -1.704698
H 0.603673 -0.445460 -2.021366
C 1.340284 1.290151 0.864130
H 0.591624 2.073481 0.717662
H 2.293509 1.659986 0.468224
O -2.894676 -0.102923 1.180529
C -0.553628 -2.030958 0.097354
H -1.539204 -2.435992 0.340247
H 0.180843 -2.709846 0.560974
H 1.465001 1.100165 1.935681
C -0.394480 -0.690913 2.161392
H -0.498598 0.287818 2.645772
H -1.236085 -1.303158 2.504517
H 0.537064 -1.154394 2.525375
H 0.111077 1.202460 -1.540356
H -0.423249 -2.093613 -0.991150
Se 2.399816 -1.346934 0.313051
C 3.932719 -0.264510 -0.086966
C 4.640345 0.374605 0.940042
C 4.381078 -0.130574 -1.407435
C 5.763766 1.147978 0.648674
H 4.301016 0.269443 1.970773
C 5.503883 0.645851 -1.697465
H 3.845440 -0.638102 -2.209976
C 6.197961 1.286172 -0.670692
H 6.302367 1.643585 1.457047
H 5.839039 0.744945 -2.730393
H 7.076491 1.890656 -0.897496
O -1.589979 1.754971 0.306120
C -1.734083 2.417940 1.514535
H -0.792832 2.482587 2.093817
H -2.061236 3.457414 1.333468
H -2.477235 1.939852 2.173452
59
H_b_conf_5 Eopt -3396.647818
C 3.452942 0.491443 -1.924160
C 3.245680 -0.640969 -0.880372
C 2.975227 1.691631 0.962341
C 3.297862 2.538594 -0.294483
H 3.019333 0.187573 -2.887330
H 4.367791 2.794823 -0.313283
C 4.568427 -0.921776 -0.145046
H 5.321425 -1.292973 -0.863614
C 4.223768 0.993970 1.542359
H 3.895496 0.289393 2.317903
H 3.047712 -1.563339 -1.452281
H 2.664531 2.399601 1.750144
C 5.165865 0.287875 0.568992
H 6.063624 -0.026935 1.123532
H 5.522079 1.015046 -0.176593
C 2.872309 1.875821 -1.604868
H 1.778685 1.800682 -1.581677
H 3.118118 2.557494 -2.434568
H 4.397937 -1.723384 0.585991

H 4.798610 1.780363 2.056879
H 4.532112 0.609401 -2.126253
H 2.759297 3.494909 -0.220809
B 1.955367 -0.478540 0.169488
C 0.469874 -0.925709 -0.628178
C -0.773916 -0.217896 -0.029692
C -0.854091 -0.283219 1.488513
H -1.806551 0.126997 1.846910
H -0.753764 -1.307655 1.860302
C -0.913916 1.233434 -0.478240
H -1.746155 1.724812 0.040799
H -1.080040 1.327266 -1.557173
O 1.858905 0.875397 0.745122
C 0.363794 -2.451366 -0.466515
H 1.324907 -2.920346 -0.715916
H -0.395243 -2.884376 -1.139232
H 0.015271 1.749604 -0.207220
C 0.518531 -0.675365 -2.146026
H -0.436158 -0.943253 -2.626093
H 0.735943 0.363037 -2.412928
H 1.291725 -1.302438 -2.606455
H -0.039375 0.325871 1.895808
H 0.126445 -2.749952 0.561057
Se -2.469488 -1.217664 -0.709929
C -3.828535 -0.017281 -0.082399
C -4.281836 1.033490 -0.891415
C -4.401087 -0.196113 1.183820
C -5.274530 1.899831 -0.433811
H -3.847568 1.173709 -1.881453
C -5.394009 0.671829 1.639991
H -4.063096 -1.019077 1.813904
C -5.833277 1.721400 0.832780
H -5.614293 2.716337 -1.071878
H -5.827794 0.522775 2.629328
H -6.610236 2.398274 1.188942
O 2.205209 -1.487614 1.254572
C 1.966840 -1.302375 2.599586
H 1.095827 -1.885598 2.957743
H 2.836742 -1.643489 3.193674
H 1.783983 -0.247637 2.865377
59
H_b_conf_6 Eopt -3396.642965
C -3.777141 -1.071736 -1.380576
C -3.168681 0.318105 -1.113921
C -3.326928 -0.473590 1.759886
C -3.827049 -1.783891 1.120692
H -3.006261 -1.775140 -1.719091
H -4.519560 -2.243033 1.844625
C -4.301880 1.296786 -0.689951
H -5.282376 0.865785 -0.956479
C -4.435529 0.609459 1.826319
H -4.391478 1.089020 2.815177
H -2.832182 0.661154 -2.104409
H -3.115921 -0.752750 2.807104
C -4.334288 1.722879 0.782490
H -3.418712 2.288014 0.993812
H -5.183393 2.408920 0.931775
C -4.559181 -1.682662 -0.216145
H -4.901439 -2.688211 -0.505787
H -5.477302 -1.095710 -0.062242
H -4.228208 2.217431 -1.285817
H -5.421899 0.121563 1.772539
H -4.481516 -0.988721 -2.227933
H -2.968130 -2.463744 1.044548
B -1.835321 0.442123 -0.119565
C -0.455789 -0.442845 -0.682326
C 0.866854 0.100939 -0.064029
C 1.410577 1.354542 -0.739562
H 1.676707 1.186016 -1.789174
H 0.642974 2.129677 -0.683624
C 0.794479 0.329798 1.440540
H 1.779467 0.608235 1.837272
H 0.428780 -0.552700 1.974586
O -2.108905 -0.011556 1.262078
C -0.384647 -0.365069 -2.214196
H -1.222225 -0.917037 -2.659506
H 0.543819 -0.808781 -2.610159
H 0.088117 1.148203 1.624665
C -0.625052 -1.918336 -0.293174
H 0.093700 -2.569545 -0.818301
H -0.504187 -2.069656 0.786196
H -1.619137 -2.283810 -0.556190
H 2.302119 1.722012 -0.216911
H -0.443542 0.669176 -2.580875
Se 2.339434 -1.354589 -0.342280
C 3.903958 -0.329630 0.085944
C 4.354079 -0.238943 1.409567
C 4.627485 0.318860 -0.924135
C 5.493112 0.505624 1.719540
H 3.807221 -0.754973 2.198925
C 5.767198 1.059898 -0.612955
H 4.288338 0.245772 -1.957674
C 6.202110 1.156755 0.709906
H 5.829286 0.571327 2.754829
H 6.317847 1.563057 -1.408501
H 7.092919 1.736639 0.952543
O -1.451008 1.896825 -0.005340
C -1.659839 2.863558 -0.968551
H -0.785686 3.537296 -1.038058
H -1.830953 2.462336 -1.981939
H -2.525113 3.512458 -0.726681
59
H_b_conf_7 Eopt -3396.646802
C -3.613975 -1.069521 -1.406400
C -2.805438 0.256849 -1.418255
C -3.453743 0.183510 1.494846
C -4.255154 -1.093136 1.135326
H -3.188176 -1.766951 -2.141578
H -5.318581 -0.846036 0.998118
C -3.770629 1.454079 -1.486555
H -4.338908 1.418394 -2.433651
C -4.172528 1.477851 1.058463
H -3.476939 2.317651 1.188863
H -2.264693 0.286760 -2.379062
H -3.427458 0.241091 2.596818
C -4.779260 1.521330 -0.343076
H -5.369745 2.446170 -0.435838
H -5.504356 0.699439 -0.444976
C -3.707796 -1.839037 -0.082040
H -2.706579 -2.196125 0.186576
H -4.323612 -2.738085 -0.243427
H -3.179788 2.379255 -1.499945
H -4.988216 1.630472 1.783031
H -4.640146 -0.874860 -1.764909
H -4.213717 -1.785165 1.989406
B -1.636960 0.446991 -0.237709
C -0.195692 -0.441628 -0.664764
C 0.635263 -0.892632 0.562007
C 0.893307 0.210567 1.574487
H 1.354337 1.094631 1.123029
H -0.074519 0.497557 1.998943
C 0.031300 -2.104250 1.275439
H 0.058159 -3.013136 0.661196
H -1.012220 -1.863973 1.511540
O -2.115071 0.061860 1.103531
C 0.617237 0.507016 -1.556504
H 1.079944 1.325573 -0.991456
H -0.040120 0.973081 -2.302186
H 0.569722 -2.310823 2.208707
C -0.503637 -1.665668 -1.543935
H 0.419454 -2.218346 -1.783878
H -1.200617 -2.374041 -1.085679
H -0.938396 -1.345792 -2.498730
H 1.541800 -0.146501 2.383894
H 1.406816 -0.036482 -2.096567
Se 2.500163 -1.650317 0.036001
C 3.648728 -0.108330 -0.027557
C 4.053734 0.424580 -1.258157
C 4.153367 0.453273 1.152657
C 4.924852 1.512616 -1.306585

H 3.678039 -0.012910 -2.182486
C 5.015724 1.549107 1.103150
H 3.870236 0.027989 2.114799
C 5.403986 2.082102 -0.126055
H 5.228054 1.917190 -2.272756
H 5.391563 1.981061 2.031189
H 6.080835 2.935981 -0.164313
O -1.300614 1.910833 -0.268000
C -0.994149 2.688862 0.827651
H -1.504478 3.668099 0.751937
H -1.305407 2.232650 1.782967
H 0.089481 2.905231 0.905826
59
H_b_conf_8 Eopt -3396.645783
C -3.486301 -1.573209 -0.563463
C -2.574928 -0.442311 -1.108021
C -3.680592 1.021927 1.246803
C -4.582772 -0.225579 1.425759
H -2.966358 -2.539074 -0.672240
H -5.568596 -0.048752 0.970976
C -3.396126 0.547024 -1.955691
H -3.818590 0.017651 -2.828674
C -4.163586 1.916751 0.085039
H -3.399264 2.681301 -0.106790
H -1.911845 -0.916505 -1.845233
H -3.814934 1.643520 2.149294
C -4.548199 1.225526 -1.222138
H -5.011445 1.970349 -1.887934
H -5.336043 0.483922 -1.019306
C -3.951830 -1.515619 0.901283
H -3.089287 -1.718345 1.541144
H -4.662074 -2.341452 1.066814
H -2.716277 1.316600 -2.344148
H -5.053852 2.446340 0.460254
H -4.376959 -1.659978 -1.211723
H -4.770277 -0.373941 2.499530
B -1.594454 0.331176 -0.002250
C -0.200177 -0.536151 0.547103
C 0.685729 -1.101665 -0.594121
C 0.208038 -2.460607 -1.122060
H -0.867617 -2.431318 -1.324380
H 0.728203 -2.718992 -2.052555
C 0.898661 -0.139104 -1.754349
H -0.062869 0.038072 -2.249140
H 1.600537 -0.562400 -2.483232
O -2.325872 0.672396 1.238294
C -0.584449 -1.664002 1.511756
H -1.135003 -1.240648 2.360566
H 0.311477 -2.170951 1.909533
H 1.264170 0.838429 -1.422982
C 0.585228 0.483610 1.384023
H -0.105392 1.035646 2.032931
H 1.322477 -0.018850 2.027417
H 1.123589 1.208777 0.762598
H 0.380185 -3.271077 -0.402249
H -1.219923 -2.429793 1.050087
Se 2.575037 -1.661083 0.061990
C 3.605384 -0.036647 0.070784
C 3.976059 0.554876 1.285394
C 4.052941 0.535762 -1.126516
C 4.754629 1.711238 1.301142
H 3.640005 0.112337 2.222824
C 4.818766 1.702327 -1.109827
H 3.800464 0.066282 -2.076281
C 5.171589 2.293525 0.103050
H 5.031897 2.161340 2.254878
H 5.148017 2.143467 -2.051088
H 5.773362 3.202580 0.115469
O -1.132789 1.585186 -0.689639
C -0.877577 2.773843 -0.034611
H 0.202735 3.015954 -0.008573
H -1.370894 3.615826 -0.557783
H -1.242257 2.774888 1.006313
59
H_b_conf_9 Eopt -3396.644065
C 3.598432 -0.543800 1.671267
C 2.728676 0.678273 1.252472
C 3.512322 -0.275204 -1.472592
C 4.398962 -1.300279 -0.721097
H 3.151343 -1.025152 2.552437
H 5.433972 -0.934617 -0.650967
C 3.657861 1.875718 0.966841
H 4.206169 2.138886 1.889480
C 4.113792 1.145269 -1.460229
H 3.340565 1.837818 -1.819735
H 2.163904 0.968011 2.156210
H 3.516148 -0.571342 -2.535913
C 4.688156 1.646923 -0.136090
H 5.224704 2.590366 -0.321708
H 5.453780 0.940312 0.217231
C 3.847359 -1.670737 0.655285
H 2.904219 -2.198736 0.478082
H 4.528389 -2.402022 1.119137
H 3.067591 2.759304 0.702792
H 4.927949 1.142592 -2.202381
H 4.580203 -0.177154 2.020501
H 4.446553 -2.226767 -1.312359
B 1.606446 0.418229 0.039308
C 0.191672 -0.381968 0.642245
C -0.618773 -1.033580 -0.507579
C -0.823981 -0.117408 -1.705525
H -1.459793 -0.599989 -2.458110
H -1.268415 0.844557 -1.426922
C -0.032994 -2.371781 -0.966729
H -0.535722 -2.722029 -1.877022
H -0.131961 -3.154438 -0.203257
O 2.179768 -0.380097 -1.063440
C -0.665148 0.661179 1.370934
H -1.437181 0.168466 1.979426
H -1.169416 1.352924 0.687547
H 1.030622 -2.216222 -1.178804
C 0.524370 -1.432188 1.711922
H -0.388576 -1.953228 2.045629
H 1.235355 -2.193782 1.379609
H 0.950622 -0.940519 2.595692
H 0.161137 0.090797 -2.136692
H -0.048236 1.255845 2.057010
Se -2.505197 -1.671727 0.091902
C -3.624930 -0.110292 0.004265
C -4.047873 0.402175 -1.228836
C -4.090923 0.488361 1.182339
C -4.888967 1.514549 -1.281964
H -3.719516 -0.074186 -2.151602
C -4.943799 1.590027 1.127892
H -3.774589 0.092069 2.147096
C -5.339728 2.111536 -0.104767
H -5.200057 1.907970 -2.250208
H -5.296707 2.044769 2.054077
H -6.000781 2.977508 -0.147026
O 1.209984 1.718008 -0.609390
C 0.819823 2.885944 0.015031
H -0.253784 3.103206 -0.144793
H 0.985625 2.894087 1.105872
H 1.376474 3.748658 -0.399413
59
H'_b_conf_1 Eopt -3396.643492
C -1.539588 -1.994286 -0.874069
C -1.762681 -0.474602 -1.089656
C -3.713382 -0.869706 1.133721
C -3.374173 -2.374842 0.985962
H -0.464886 -2.210952 -0.984771
H -4.129550 -2.873724 0.360755
C -2.994848 -0.237110 -1.980806
H -2.824890 -0.690771 -2.973981
C -4.640882 -0.379389 -0.000265
H -4.715514 0.714310 0.057898
H -0.919776 -0.137843 -1.712581
H -4.322287 -0.769691 2.049690
C -4.299380 -0.803749 -1.428811
H -5.130490 -0.507152 -2.087813

H -4.265302 -1.902955 -1.477956
C -1.961587 -2.632190 0.460246
H -1.275344 -2.286662 1.238058
H -1.815335 -3.721377 0.379124
H -3.109597 0.843624 -2.135449
H -5.640934 -0.777143 0.236249
H -2.027943 -2.548767 -1.695717
H -3.441728 -2.851040 1.975463
B -1.767093 0.490294 0.270039
C -0.239080 0.825878 0.946890
C 0.829870 1.442589 -0.024839
C 0.240701 2.457049 -1.008184
H -0.466217 1.999794 -1.706859
H -0.320847 3.217292 -0.449729
C 1.989898 2.124661 0.704573
H 1.618694 3.038233 1.187427
H 2.774677 2.423726 -0.000357
O -2.561595 -0.111685 1.369207
C 0.337553 -0.401403 1.660748
H -0.358590 -0.716746 2.448080
H 1.309215 -0.190294 2.137468
H 2.445444 1.491565 1.475143
C -0.496978 1.860296 2.065761
H -0.698912 2.864398 1.673930
H -1.372911 1.544234 2.645410
H 0.352046 1.935304 2.763664
H 1.043875 2.948114 -1.572703
H 0.481338 -1.245925 0.978554
Se 1.665209 0.040747 -1.254545
C 3.314908 -0.408626 -0.368374
C 4.478273 0.329909 -0.620253
C 3.386755 -1.521313 0.478323
C 5.683560 -0.018353 -0.009949
H 4.437472 1.181896 -1.298504
C 4.593121 -1.870011 1.086766
H 2.493687 -2.117311 0.661406
C 5.743173 -1.117396 0.847630
H 6.579830 0.569228 -0.211275
H 4.632286 -2.736839 1.746841
H 6.685396 -1.390297 1.323539
O -2.404548 1.788019 -0.161527
C -3.151752 2.588096 0.678202
H -2.628145 3.527974 0.943897
H -4.095741 2.891878 0.184974
H -3.422400 2.083368 1.621334
59
H'_b_conf_10 Eopt -3396.642441
C -3.826133 1.229654 -0.892240
C -3.207561 0.840354 0.479182
C -2.441071 -1.602223 -1.059672
C -3.195179 -0.930498 -2.235555
H -3.752024 2.317103 -1.031537
H -4.214498 -1.339801 -2.305738
C -4.147231 -0.111762 1.242285
H -5.071361 0.423560 1.526311
C -3.426891 -2.230648 -0.055167
H -2.859396 -2.650070 0.782134
H -3.188682 1.760569 1.088348
H -1.900903 -2.465937 -1.484977
C -4.567563 -1.359746 0.467693
H -5.209614 -1.982677 1.109864
H -5.203464 -1.060889 -0.379533
C -3.226145 0.592546 -2.154186
H -2.189105 0.927619 -2.259413
H -3.767559 0.979170 -3.032362
H -3.657025 -0.407883 2.180988
H -3.875044 -3.090529 -0.578607
H -4.910705 1.022354 -0.882459
H -2.695507 -1.199730 -3.177880
B -1.636734 0.287862 0.468772
C -0.513756 1.583586 0.276071
C 0.882910 1.178990 -0.312636
C 0.813529 0.838679 -1.800853
H 1.769562 0.430028 -2.152971
H 0.013381 0.117958 -1.992095
C 1.967152 2.242406 -0.110350
H 2.885763 1.965941 -0.642549
H 2.217727 2.392862 0.945579
O -1.439201 -0.777146 -0.534225
C -0.316589 2.219380 1.666518
H -1.289592 2.359166 2.155442
H 0.162365 3.209526 1.599187
H 1.631134 3.202770 -0.524098
C -1.067104 2.737219 -0.588323
H -0.347775 3.566332 -0.675473
H -1.348988 2.448303 -1.603831
H -1.958048 3.154262 -0.102572
H 0.625884 1.755636 -2.379367
H 0.282743 1.584030 2.327395
Se 1.508675 -0.477735 0.677373
C 3.341798 -0.529039 0.086103
C 4.356755 0.061994 0.850665
C 3.689108 -1.204701 -1.091063
C 5.686926 -0.001598 0.435454
H 4.097735 0.579125 1.774880
C 5.019727 -1.265956 -1.508598
H 2.909123 -1.688144 -1.679849
C 6.021428 -0.664220 -0.746754
H 6.465004 0.466082 1.039724
H 5.273494 -1.792715 -2.429096
H 7.061361 -0.715437 -1.070525
O -1.304238 -0.231373 1.851105
C -1.149897 -1.553288 2.209964
H -0.349135 -1.643964 2.966745
H -2.066228 -1.980696 2.666534
H -0.869692 -2.198849 1.361013
59
H'_b_conf_11 Eopt -3396.640072
C -4.076620 -0.984422 0.897421
C -3.175607 -0.896186 -0.353946
C -2.599962 1.848800 0.674750
C -3.489529 1.339152 1.824636
H -3.574083 -1.523757 1.706518
H -3.964223 2.220995 2.283640
C -3.934193 -0.111976 -1.463346
H -5.017090 -0.127699 -1.249392
C -3.432761 2.310227 -0.548609
H -2.992349 3.242394 -0.931374
H -3.110471 -1.937034 -0.713058
H -2.127638 2.761671 1.079116
C -3.495107 1.336747 -1.729784
H -2.493111 1.302505 -2.171284
H -4.168353 1.769175 -2.487245
C -4.582537 0.344197 1.453227
H -5.197425 0.143510 2.344193
H -5.261542 0.813927 0.725997
H -3.827252 -0.643639 -2.419937
H -4.446342 2.576529 -0.210038
H -4.964969 -1.590236 0.643382
H -2.825794 0.906899 2.589473
B -1.604312 -0.352519 -0.212786
C -0.523318 -1.357393 0.642943
C 0.895587 -0.720334 0.894613
C 0.888329 0.346702 1.990700
H 0.708136 -0.128372 2.966656
H 1.866752 0.839729 2.054355
C 1.952832 -1.765835 1.271126
H 1.585726 -2.379988 2.105013
H 2.875566 -1.280065 1.610128
O -1.535625 1.004008 0.365573
C -0.345769 -2.674482 -0.131709
H -1.321553 -3.072578 -0.438617
H 0.134930 -3.454303 0.479551
H 2.205671 -2.434292 0.440859
C -1.095884 -1.735559 2.020913
H -1.519938 -0.870623 2.546656
H -1.892204 -2.478652 1.895402
H -0.347181 -2.197261 2.683601
H 0.109314 1.088986 1.803308
H 0.260901 -2.537884 -1.036901

Se 1.556212 0.172893 -0.816704
C 3.410181 0.410360 -0.312610
C 3.826922 1.574685 0.346277
C 4.375313 -0.542832 -0.665961
C 5.171143 1.768286 0.670424
H 3.089720 2.336240 0.602971
C 5.719283 -0.350165 -0.345701
H 4.065898 -1.445886 -1.193392
C 6.120518 0.805840 0.326289
H 5.477111 2.678863 1.186830
H 6.456070 -1.104216 -0.625135
H 7.171091 0.958224 0.575452
O -1.017086 -0.222953 -1.598142
C -1.214461 -1.081345 -2.659364
H -0.248331 -1.454405 -3.051014
H -1.826076 -1.965419 -2.414663
H -1.715096 -0.564319 -3.502365
59
H’_b_conf_12 Eopt -3396.639708
C -3.952056 0.337633 -1.377357
C -3.220498 0.879196 -0.135765
C -2.385968 -1.960123 0.280797
C -3.116999 -2.105807 -1.068609
H -3.395714 0.580446 -2.291495
H -3.512045 -3.133971 -1.103508
C -4.078063 0.589373 1.129933
H -5.114313 0.356155 0.828655
C -3.364445 -1.902135 1.482463
H -2.965090 -2.539064 2.285187
H -3.243924 1.971943 -0.268801
H -1.826351 -2.905893 0.386832
C -3.580182 -0.509558 2.077803
H -2.623498 -0.181906 2.501995
H -4.293757 -0.601284 2.912283
C -4.280342 -1.155913 -1.348077
H -4.742153 -1.439004 -2.306638
H -5.057343 -1.328795 -0.587928
H -4.158702 1.506722 1.730908
H -4.327313 -2.351613 1.191758
H -4.913825 0.872679 -1.478243
H -2.360575 -2.038955 -1.861898
B -1.617139 0.490104 0.115199
C -0.536233 0.975443 -1.115163
C 0.983366 1.015380 -0.693720
C 1.930351 1.090742 -1.898377
H 1.931655 0.176716 -2.501272
H 1.634267 1.928245 -2.545951
C 1.344317 2.172969 0.236396
H 2.404880 2.120808 0.514252
H 0.733899 2.158567 1.139070
O -1.412513 -0.963413 0.306740
C -0.938419 2.391405 -1.567798
H -1.906812 2.347280 -2.084359
H -0.222515 2.842647 -2.273812
H 1.200340 3.129711 -0.287503
C -0.657196 0.049304 -2.335234
H -0.162056 0.466949 -3.226240
H -0.218738 -0.936240 -2.129377
H -1.701942 -0.113761 -2.604391
H 2.959143 1.282266 -1.570701
H -1.049860 3.080630 -0.719366
Se 1.423859 -0.711903 0.293857
C 3.350156 -0.566828 0.284031
C 4.018946 0.110453 1.312375
C 4.109112 -1.186041 -0.718403
C 5.412341 0.193244 1.321881
H 3.440018 0.575398 2.110911
C 5.501905 -1.107114 -0.708435
H 3.599769 -1.728449 -1.515566
C 6.157297 -0.414404 0.310921
H 5.916584 0.728399 2.127292
H 6.076948 -1.591057 -1.498725
H 7.245954 -0.353199 0.319890
O -1.175706 1.139332 1.403159
C -1.640170 2.332698 1.914399
H -0.803129 3.011221 2.171289
H -2.297767 2.894611 1.230991
H -2.209130 2.179132 2.853762
59
H’_b_conf_13 Eopt -3396.640650
C -3.952203 1.045789 -0.943967
C -3.159970 0.902236 0.377715
C -2.524743 -1.721398 -0.858275
C -3.638785 -1.369197 -1.858684
H -3.312023 1.467969 -1.725058
H -4.226179 -2.279648 -2.063952
C -3.974821 0.093104 1.413230
H -4.997655 0.509382 1.470262
C -3.014663 -2.221644 0.512462
H -2.127023 -2.297228 1.150280
H -3.116118 1.922495 0.801071
H -2.013651 -2.594960 -1.305202
C -4.120275 -1.438955 1.235992
H -4.226927 -1.884074 2.237553
H -5.070185 -1.661135 0.734013
C -4.597540 -0.223296 -1.511130
H -5.129610 0.054456 -2.434486
H -5.379495 -0.570858 -0.826220
H -3.540542 0.273610 2.401493
H -3.396070 -3.244191 0.360751
H -4.769372 1.774212 -0.795911
H -3.119281 -1.116161 -2.796608
B -1.593180 0.356838 0.199962
C -0.469992 1.540273 -0.287839
C 0.942851 0.973987 -0.692787
C 0.952845 0.296269 -2.064255
H 1.918262 -0.194056 -2.243140
H 0.143122 -0.432557 -2.142999
C 2.031450 2.053766 -0.703831
H 1.697682 2.903103 -1.316036
H 2.954031 1.671557 -1.156743
O -1.557478 -0.712732 -0.806969
C -0.287108 2.576514 0.834199
H 0.207158 3.490844 0.470178
H 0.313420 2.184260 1.664649
H 2.272981 2.427655 0.297147
C -1.011278 2.320974 -1.498720
H -1.882026 2.914962 -1.190318
H -0.273651 3.031961 -1.904833
H -1.326525 1.658433 -2.313552
H 0.831710 1.056411 -2.850558
H -1.259836 2.885037 1.237495
Se 1.519447 -0.423875 0.678493
C 3.392529 -0.522956 0.206509
C 4.342640 0.254978 0.882235
C 3.834631 -1.422020 -0.773063
C 5.698235 0.156721 0.568227
H 4.012672 0.946746 1.657977
C 5.190620 -1.519532 -1.091031
H 3.108055 -2.052064 -1.287056
C 6.125648 -0.729479 -0.422123
H 6.423592 0.772403 1.101392
H 5.516652 -2.221547 -1.859317
H 7.185250 -0.808011 -0.667229
O -1.103886 -0.252647 1.490369
C -1.151519 0.333834 2.740695
H -0.135006 0.544737 3.125081
H -1.710174 1.283778 2.769287
H -1.628920 -0.346738 3.471449
59
H’_b_conf_14 Eopt -3396.642291
C -3.027227 0.127136 -1.847293
C -3.074975 0.452915 -0.328817
C -1.412632 -2.016811 -0.177354
C -1.621395 -2.085163 -1.709944
H -3.002907 1.063615 -2.422694
H -2.438473 -2.783337 -1.947368
C -4.172686 -0.383927 0.351640
H -5.162607 -0.097763 -0.047843
C -2.668101 -2.456219 0.606213

H	-2.502961	-2.246477	1.670419
H	-3.429583	1.494444	-0.243495
H	-0.658985	-2.786463	0.072260
C	-4.018868	-1.891442	0.171981
H	-4.807725	-2.407529	0.741165
H	-4.196092	-2.155999	-0.881649
C	-1.855180	-0.721195	-2.358415
H	-0.927702	-0.154202	-2.223626
H	-1.976941	-0.869072	-3.443400
H	-4.178037	-0.147116	1.424441
H	-2.714590	-3.552576	0.509207
H	-3.970543	-0.362336	-2.147908
H	-0.715426	-2.509935	-2.167801
B	-1.624821	0.411059	0.503997
C	-0.718636	1.845268	0.216522
C	0.824112	1.728620	0.475330
C	1.542120	3.080411	0.602844
H	1.219454	3.588811	1.522789
H	2.626486	2.926212	0.671735
C	1.151748	0.923404	1.723931
H	0.779340	-0.098447	1.652350
H	0.666715	1.408659	2.580930
O	-0.835178	-0.800072	0.202763
C	-1.278117	2.901904	1.195033
H	-0.996970	2.701851	2.235465
H	-2.375463	2.886570	1.162018
H	2.232985	0.908079	1.914573
C	-0.960104	2.453652	-1.178564
H	-0.363880	3.366411	-1.336172
H	-0.733284	1.769213	-2.002883
H	-2.011894	2.749071	-1.281590
H	1.351180	3.748896	-0.244688
H	-0.953248	3.920979	0.931490
Se	1.684442	0.849958	-1.151068
C	2.854903	-0.443204	-0.352712
C	2.325384	-1.636451	0.157532
C	4.237632	-0.234948	-0.325350
C	3.172956	-2.599864	0.701866
H	1.244470	-1.778051	0.133114
C	5.083948	-1.208222	0.211080
H	4.650623	0.691516	-0.724547
C	4.553826	-2.390330	0.726763
H	2.753165	-3.523040	1.102335
H	6.161060	-1.038290	0.227205
H	5.214850	-3.148535	1.147426
O	-2.004438	0.423120	1.969259
C	-1.476667	-0.384040	2.951889
H	-0.900184	0.194399	3.700932
H	-2.281517	-0.901299	3.511282
H	-0.803549	-1.165467	2.559631

59

H’_b_conf_15 Eopt -3396.640927

C	-3.978492	1.468853	-0.050980
C	-3.215278	0.525809	0.893032
C	-2.515140	-1.005182	-1.569181
C	-3.232399	0.205496	-2.199010
H	-3.417850	2.398769	-0.209730
H	-3.664872	-0.154299	-3.146406
C	-4.089759	-0.724645	1.186724
H	-5.148661	-0.490867	0.974988
C	-3.521547	-2.049046	-1.024325
H	-3.160203	-3.052398	-1.294475
H	-3.150345	1.062273	1.852816
H	-2.000535	-1.487674	-2.417820
C	-3.720996	-2.042459	0.492104
H	-2.793389	-2.405023	0.940304
H	-4.491061	-2.791951	0.736170
C	-4.361879	0.856316	-1.399156
H	-4.833926	1.628794	-2.025819
H	-5.144073	0.100390	-1.229036
H	-4.048483	-0.935962	2.265094
H	-4.483939	-1.927842	-1.544905
H	-4.918233	1.781865	0.439543
H	-2.469280	0.944622	-2.472953
B	-1.634439	0.108096	0.566140
C	-0.562187	1.443170	0.468012
C	0.965036	1.106190	0.643354
C	1.895552	2.241257	0.196440
H	1.888267	2.403994	-0.886441
H	1.595251	3.177557	0.687616
C	1.344633	0.760128	2.084023
H	0.621897	0.078987	2.535603
H	1.363271	1.686381	2.677976
O	-1.493562	-0.690303	-0.668057
C	-0.942382	2.432577	1.586582
H	-1.916103	2.886446	1.361475
H	-0.224121	3.261407	1.694141
H	2.354153	0.333352	2.133252
C	-0.725945	2.176856	-0.871736
H	-0.319385	1.585249	-1.702946
H	-1.776841	2.366440	-1.093748
H	-0.223073	3.156787	-0.870941
H	2.928309	2.031994	0.501514
H	-1.030245	1.929567	2.558832
Se	1.399578	-0.483875	-0.557355
C	3.323116	-0.439188	-0.418490
C	4.094307	0.252827	-1.362219
C	3.977326	-1.145008	0.599839
C	5.486430	0.260216	-1.274781
H	3.595362	0.793672	-2.166870
C	5.370282	-1.135887	0.689953
H	3.388451	-1.707770	1.324882
C	6.127993	-0.432675	-0.246677
H	6.071909	0.807280	-2.014574
H	5.863611	-1.686602	1.491383
H	7.216288	-0.428037	-0.178970
O	-1.250960	-0.711143	1.779427
C	-0.662725	-1.960248	1.750863
H	0.397047	-1.934672	2.072542
H	-1.186287	-2.647820	2.442825
H	-0.675864	-2.414203	0.746855

59

H’_b_conf_16 Eopt -3396.644300

C	2.470045	-1.519126	1.818471
C	2.844200	-0.084517	1.355705
C	2.324420	-1.267288	-1.341522
C	2.074574	-2.579148	-0.557937
H	1.927715	-1.467250	2.773326
H	2.977089	-3.208181	-0.589756
C	4.319362	-0.046250	0.917484
H	4.972045	-0.257786	1.783841
C	3.828149	-0.937144	-1.450152
H	3.936217	0.063349	-1.887072
H	2.803420	0.557865	2.253228
H	2.004870	-1.453807	-2.381442
C	4.676737	-1.040785	-0.184195
H	5.732717	-0.904505	-0.465285
H	4.607669	-2.065699	0.210876
C	1.600229	-2.365629	0.879377
H	0.613139	-1.898311	0.807989
H	1.444293	-3.353507	1.341869
H	4.557560	0.971205	0.577587
H	4.238605	-1.643365	-2.189277
H	3.391420	-2.082571	2.049085
H	1.289278	-3.150502	-1.074688
B	1.834581	0.650471	0.247901
C	0.390903	1.218951	0.968210
C	-0.790327	1.365174	-0.051719
C	-2.010373	2.087616	0.520058
H	-2.375062	1.661368	1.459924
H	-1.735489	3.135237	0.703943
C	-0.387867	2.099065	-1.332964
H	0.353290	1.531076	-1.897070
H	0.039073	3.080392	-1.084697
O	1.498876	-0.229634	-0.889501
C	0.704844	2.614541	1.552460
H	0.872594	3.372431	0.780031
H	1.632622	2.561624	2.138362
H	-1.270613	2.262259	-1.965572
C	-0.056580	0.395444	2.189395

H -0.958315 0.817146 2.661093
H -0.269945 -0.652596 1.960756
H 0.728551 0.416592 2.956186
H -2.841686 2.090108 -0.195153
H -0.090755 2.967682 2.227904
Se -1.357407 -0.488588 -0.693900
C -3.238598 -0.507339 -0.271499
C -3.670962 -0.747718 1.039658
C -4.195268 -0.338321 -1.279685
C -5.031836 -0.793182 1.340160
H -2.932315 -0.894928 1.828686
C -5.558117 -0.395326 -0.981005
H -3.867386 -0.154259 -2.303104
C -5.979614 -0.619339 0.329537
H -5.352941 -0.973349 2.366512
H -6.292029 -0.260842 -1.776469
H -7.043739 -0.661042 0.563477
O 2.586443 1.871146 -0.233855
C 2.789846 2.246123 -1.542504
H 3.870406 2.374095 -1.755749
H 2.402361 1.514822 -2.271215
H 2.309149 3.217971 -1.772180
59
H’_b_conf_17 Eopt -3396.640637
C 1.691584 0.657444 2.064615
C 1.041374 0.857825 0.685406
C 3.634216 -0.161374 -0.376230
C 3.887784 -0.316616 1.137589
H 1.355970 -0.286788 2.513046
H 4.977601 -0.237519 1.279546
C 1.537775 2.184934 0.046520
H 1.925061 2.854535 0.835534
C 3.849092 1.299042 -0.848160
H 4.401021 1.279427 -1.799717
H -0.026799 1.028026 0.881215
H 4.437544 -0.755978 -0.845972
C 2.566174 2.094318 -1.089669
H 2.061978 1.646889 -1.952194
H 2.848059 3.117116 -1.387660
C 3.217891 0.688529 2.070577
H 3.578352 0.510021 3.095554
H 3.560217 1.701260 1.809983
H 0.670459 2.707293 -0.378984
H 4.502921 1.821427 -0.132614
H 1.340579 1.451215 2.748832
H 3.611860 -1.340064 1.425360
B 1.066102 -0.385653 -0.409213
C 0.344246 -1.890319 0.009796
C -1.097359 -1.906821 0.624658
C -1.722082 -3.314738 0.634858
H -1.929405 -3.689761 -0.374952
H -1.046183 -4.027488 1.124287
C -1.166367 -1.358143 2.046692
H -2.193330 -1.404303 2.429523
H -0.814817 -0.326184 2.119018
O 2.448881 -0.730043 -0.844207
C 1.287018 -2.663978 0.956000
H 1.475811 -2.156711 1.907783
H 2.253014 -2.773640 0.449538
H -0.541826 -1.978014 2.705418
C 0.333571 -2.709605 -1.294823
H 0.228488 -3.790296 -1.104990
H -0.483550 -2.402936 -1.959476
H 1.280362 -2.563115 -1.828942
H -2.666735 -3.310220 1.192300
H 0.923568 -3.677218 1.188563
Se -2.496573 -0.959479 -0.508698
C -2.432282 0.905144 -0.059733
C -2.840205 1.361244 1.199903
C -2.053133 1.834557 -1.035199
C -2.818522 2.724145 1.496751
H -3.173050 0.648390 1.952712
C -2.066023 3.199148 -0.745650
H -1.714377 1.476442 -2.004246
C -2.430960 3.647133 0.524248
H -3.119089 3.064829 2.487931
H -1.766068 3.914032 -1.512440
H -2.418644 4.712867 0.753899
O 0.298169 0.171682 -1.570344
C 0.580510 -0.088677 -2.894365
H -0.154445 -0.777293 -3.356149
H 0.542205 0.846719 -3.487561
H 1.583994 -0.524669 -3.038648
59
H’_b_conf_18 Eopt -3396.642740
C -2.647720 -1.175008 -1.936722
C -2.958283 0.133610 -1.153213
C -2.156063 -1.595265 1.164086
C -2.063798 -2.732410 0.116664
H -2.177816 -0.921941 -2.897912
H -2.983462 -3.335943 0.128208
C -4.382901 0.034648 -0.570383
H -5.109455 -0.057008 -1.398082
C -3.612723 -1.245016 1.530399
H -3.595685 -0.305088 2.099255
H -3.015503 0.934302 -1.913700
H -1.713379 -1.985624 2.096971
C -4.612475 -1.139190 0.379174
H -5.625864 -1.058947 0.802430
H -4.608033 -2.079805 -0.191584
C -1.739088 -2.235055 -1.292488
H -0.719295 -1.840405 -1.246317
H -1.702538 -3.105789 -1.966689
H -4.642066 0.956772 -0.039677
H -3.962084 -2.037937 2.210530
H -3.602239 -1.658831 -2.210764
H -1.252208 -3.414373 0.410561
B -1.823211 0.619689 -0.027151
C -0.433947 1.323882 -0.714141
C 0.785577 1.282008 0.276088
C 1.976557 2.117622 -0.194578
H 2.837021 1.996733 0.473724
H 2.303554 1.880184 -1.212641
C 0.426502 1.751258 1.689790
H 1.327119 1.760018 2.318814
H -0.335429 1.111939 2.139285
O -1.353923 -0.511658 0.797930
C -0.728460 2.802613 -1.040029
H -1.667813 2.879360 -1.604137
H 0.055815 3.243962 -1.674664
H 0.026204 2.774873 1.654464
C -0.058377 0.714151 -2.074670
H 0.115265 -0.363444 -2.038771
H -0.867439 0.901406 -2.793783
H 0.847037 1.179243 -2.497235
H 1.690660 3.177499 -0.164886
H -0.830230 3.426658 -0.146245
Se 1.394832 -0.655680 0.503160
C 3.290367 -0.518336 0.159676
C 3.779674 -0.527184 -1.153531
C 4.203288 -0.464508 1.220056
C 5.150577 -0.459033 -1.401115
H 3.077098 -0.582762 -1.986218
C 5.576220 -0.404349 0.973530
H 3.832459 -0.463037 2.245570
C 6.053033 -0.398461 -0.337418
H 5.515365 -0.459886 -2.428823
H 6.274775 -0.360312 1.809973
H 7.125066 -0.349695 -0.530534
O -2.444683 1.581607 0.961024
C -3.212457 2.687215 0.654443
H -3.506884 2.752286 -0.407675
H -4.148804 2.687245 1.245390
H -2.693602 3.634288 0.898160
59
H’_b_conf_19 Eopt -3396.644653
C 1.762570 -1.617553 -1.441904
C 1.433838 -1.185820 0.010818
C 4.138943 0.057601 -0.185928
C 4.217015 -0.651611 -1.561914

H	0.847075	-1.537014	-2.049759
H	4.764557	-1.601144	-1.467419
C	2.079150	-2.155701	1.016830
H	1.660696	-3.168307	0.871784
C	4.346769	-0.932693	0.980998
H	4.116289	-0.415329	1.921522
H	0.354181	-1.354220	0.128297
H	5.008553	0.736030	-0.129441
C	3.597850	-2.263601	0.920666
H	3.968170	-2.908976	1.732763
H	3.865457	-2.783980	-0.011811
C	2.852892	-0.861845	-2.220042
H	2.471221	0.132925	-2.464690
H	3.007899	-1.378204	-3.181091
H	1.806377	-1.837031	2.031520
H	5.425726	-1.155636	0.999326
H	2.011277	-2.694494	-1.448934
H	4.812373	-0.029077	-2.246350
B	1.705001	0.411071	0.402332
C	0.567250	1.543838	-0.185354
C	-0.941728	1.286032	0.182966
C	-1.135724	0.701797	1.578402
H	-0.687581	-0.289895	1.684223
H	-0.634212	1.354398	2.304775
C	-1.818794	2.539421	0.075820
H	-2.879530	2.277342	0.177929
H	-1.684921	3.063421	-0.877748
O	3.016323	0.887698	-0.104252
C	0.713717	1.759735	-1.698858
H	1.737068	2.092689	-1.912169
H	0.024204	2.530865	-2.079488
H	-1.577556	3.234668	0.892233
C	0.966785	2.891631	0.454107
H	0.695245	2.948348	1.515160
H	2.054195	3.011585	0.379297
H	0.501730	3.747929	-0.059168
H	-2.201728	0.654512	1.835385
H	0.540585	0.845983	-2.283376
Se	-1.709847	-0.012225	-1.187115
C	-3.335174	-0.508223	-0.292588
C	-4.525349	0.191095	-0.525990
C	-3.346693	-1.604988	0.579401
C	-5.703810	-0.186806	0.120159
H	-4.527420	1.033943	-1.217336
C	-4.524559	-1.980476	1.224471
H	-2.424892	-2.159929	0.754610
C	-5.705606	-1.272045	0.996311
H	-6.624077	0.367177	-0.067668
H	-4.519119	-2.832496	1.904678
H	-6.626848	-1.567911	1.498864
O	1.695210	0.459050	1.909593
C	2.457433	1.334662	2.654888
H	2.984228	0.796157	3.467005
H	3.224395	1.852724	2.054374
H	1.843259	2.112570	3.150655

59

H'_b_conf_2 Eopt -3396.647147

C	-2.593237	-0.365551	2.175716
C	-1.770127	-0.847002	0.953445
C	-4.132989	0.191066	-0.545776
C	-4.751171	0.407600	0.859089
H	-1.911823	0.088831	2.913862
H	-5.407769	-0.436569	1.116304
C	-2.245938	-2.239445	0.499971
H	-2.083946	-2.964315	1.318078
C	-4.148691	-1.296882	-0.957348
H	-3.544698	-1.410650	-1.867476
H	-0.751803	-1.037477	1.319039
H	-4.806942	0.685038	-1.267347
C	-3.712724	-2.320713	0.089949
H	-3.924102	-3.329270	-0.298432
H	-4.346087	-2.212325	0.983889
C	-3.717337	0.661782	1.957310
H	-3.258456	1.627561	1.730228
H	-4.252612	0.791114	2.911660
H	-1.618760	-2.560722	-0.341517
H	-5.189578	-1.524489	-1.237712
H	-3.018532	-1.245375	2.691625
H	-5.402703	1.293362	0.823287
B	-1.617772	0.218451	-0.318331
C	-0.520841	1.510909	-0.112343
C	0.934601	1.151989	0.350284
C	1.956807	2.240175	0.002161
H	2.922603	2.034139	0.479351
H	2.121619	2.338905	-1.075809
C	1.051260	0.904694	1.856791
H	2.029761	0.478331	2.107907
H	0.271219	0.244869	2.244300
O	-2.907069	0.853128	-0.673268
C	-1.083689	2.541534	0.881529
H	-2.050058	2.896457	0.503454
H	-0.426755	3.420558	0.990073
H	0.976743	1.865123	2.386929
C	-0.456492	2.234002	-1.470238
H	-1.470577	2.347001	-1.871291
H	-0.022574	3.242643	-1.381142
H	0.141066	1.677741	-2.203607
H	1.603139	3.207417	0.386671
H	-1.247869	2.125416	1.882796
Se	1.528435	-0.539410	-0.625274
C	3.386007	-0.533998	-0.090617
C	4.358547	0.078975	-0.892219
C	3.794878	-1.203650	1.070218
C	5.704826	0.046196	-0.528238
H	4.054146	0.589368	-1.806542
C	5.141513	-1.235976	1.436651
H	3.049589	-1.704692	1.688970
C	6.099568	-0.610278	0.638743
H	6.448344	0.532245	-1.160941
H	5.442098	-1.758344	2.345524
H	7.152065	-0.638246	0.922287
O	-1.118946	-0.621100	-1.465189
C	-1.407725	-0.371050	-2.793217
H	-1.769076	-1.292407	-3.288952
H	-2.186556	0.398881	-2.920267
H	-0.513213	-0.044905	-3.357768

59

H'_b_conf_3 Eopt -3396.641730

C	-3.653157	1.455860	1.217596
C	-2.766854	0.201163	1.298655
C	-3.535836	-0.264610	-1.512773
C	-4.250679	1.088177	-1.329712
H	-3.006867	2.342836	1.185239
H	-5.154961	1.073304	-1.959390
C	-3.583916	-1.070619	1.593928
H	-4.103198	-0.958155	2.563471
C	-4.249221	-1.490386	-0.910095
H	-3.591366	-2.347252	-1.113943
H	-2.164807	0.336599	2.212213
H	-3.579059	-0.443198	-2.603318
C	-4.647981	-1.495819	0.571405
H	-4.970511	-2.519502	0.819823
H	-5.545671	-0.880526	0.703101
C	-4.678582	1.548040	0.071686
H	-5.001226	2.597750	-0.013154
H	-5.585199	1.002037	0.355384
H	-2.867922	-1.891468	1.732085
H	-5.162966	-1.661764	-1.501917
H	-4.223858	1.557496	2.159446
H	-3.580057	1.835125	-1.778366
B	-1.609011	-0.052137	0.129122
C	-0.406615	1.144720	0.077268
C	0.924073	0.647586	-0.608429
C	0.688226	-0.074700	-1.936173
H	1.649983	-0.378823	-2.372677
H	0.053127	-0.952768	-1.809776
C	1.917772	1.781573	-0.871198
H	2.844411	1.404551	-1.317924
H	2.182378	2.345274	0.030968
O	-2.172370	-0.201672	-1.222534

C -0.117331 1.670844 1.492508
H -0.966411 2.269424 1.849001
H 0.770426 2.321475 1.533324
H 1.476293 2.478307 -1.595581
C -0.923693 2.342462 -0.744516
H -1.936173 2.607713 -0.425922
H -0.298920 3.240412 -0.613315
H -0.982811 2.114793 -1.815574
H 0.190759 0.596101 -2.650558
H 0.037107 0.858117 2.212285
Se 1.823317 -0.721931 0.607824
C 3.683126 -0.346116 0.249092
C 4.341608 -0.985236 -0.809625
C 4.404667 0.544628 1.054361
C 5.686038 -0.719513 -1.074313
H 3.791647 -1.691511 -1.432742
C 5.749566 0.808177 0.792822
H 3.902932 1.040227 1.886384
C 6.392828 0.178966 -0.274348
H 6.182846 -1.219087 -1.906767
H 6.297329 1.507406 1.425671
H 7.443562 0.385822 -0.479539
O -0.874861 -1.308764 0.542843
C -1.044154 -2.530407 -0.078623
H -1.308900 -2.431064 -1.144618
H -0.105809 -3.109580 -0.017155
H -1.835280 -3.148984 0.392649
59
H’_b_conf_4 Eopt -3396.645832
C -2.338689 2.087262 0.861061
C -1.821510 0.657315 1.095256
C -4.157143 -0.156437 -0.571380
C -4.281957 1.377583 -0.668157
H -1.772225 2.574775 0.057222
H -5.347421 1.595372 -0.844659
C -2.639408 -0.029417 2.224419
H -3.131025 0.738813 2.847908
C -4.725456 -0.701094 0.763366
H -5.308226 -1.608854 0.547752
H -0.807121 0.769358 1.507909
H -4.824724 -0.534196 -1.365174
C -3.677800 -1.082185 1.810435
H -3.131967 -1.948373 1.423613
H -4.207783 -1.424660 2.713746
C -3.826552 2.194386 0.537812
H -4.080842 3.251209 0.363094
H -4.412609 1.891435 1.418281
H -1.939413 -0.546611 2.896097
H -5.443148 0.024623 1.176284
H -2.150328 2.693238 1.765727
H -3.744307 1.703995 -1.569481
B -1.622675 -0.351884 -0.202377
C -0.524773 0.096691 -1.437818
C 0.912512 0.544711 -1.004096
C 1.927819 0.487117 -2.151466
H 2.132577 -0.535955 -2.484112
H 1.544288 1.056795 -3.009445
C 0.962127 1.955976 -0.420216
H 1.973063 2.191454 -0.065109
H 0.264199 2.091951 0.408561
O -2.897113 -0.665391 -0.893395
C -1.137596 1.194543 -2.327691
H -0.485705 1.464350 -3.174622
H -1.378923 2.118221 -1.791765
H 0.720062 2.687833 -1.205004
C -0.407656 -1.137910 -2.352762
H 0.226900 -1.917578 -1.913479
H -1.404416 -1.562989 -2.520929
H 0.010782 -0.880481 -3.338955
H 2.878678 0.946716 -1.856031
H -2.073725 0.806431 -2.747572
Se 1.584165 -0.738997 0.434191
C 3.429545 -0.165841 0.482183
C 3.834254 0.864728 1.340667
C 4.399706 -0.819684 -0.289354
C 5.173639 1.252957 1.404997
H 3.091646 1.364245 1.963813
C 5.739029 -0.434847 -0.224057
H 4.098451 -1.633520 -0.949679
C 6.129088 0.604452 0.622257
H 5.471086 2.060208 2.075203
H 6.480883 -0.951314 -0.834246
H 7.176119 0.904261 0.675125
O -1.057415 -1.602271 0.428013
C -1.345579 -2.883662 -0.003373
H -1.662519 -3.518220 0.846605
H -2.155447 -2.906403 -0.750378
H -0.460478 -3.379387 -0.446341
59
H’_b_conf_5 Eopt -3396.639788
C -2.783354 0.811103 2.091304
C -1.920133 -0.239980 1.341652
C -4.099299 0.027359 -0.679061
C -4.700421 1.034625 0.334802
H -2.133988 1.465768 2.689664
H -5.467844 0.535023 0.946074
C -2.490847 -1.650425 1.578735
H -2.397029 -1.914936 2.647395
C -4.307349 -1.437447 -0.245248
H -3.759808 -2.079673 -0.946760
H -0.932753 -0.252437 1.830383
H -4.696557 0.123600 -1.602502
C -3.958994 -1.819086 1.192491
H -4.255742 -2.867442 1.352491
H -4.587343 -1.230136 1.877935
C -3.666202 1.719348 1.227838
H -3.020502 2.316252 0.571419
H -4.190967 2.433987 1.881765
H -1.880928 -2.373714 1.019414
H -5.376786 -1.653178 -0.400336
H -3.426923 0.306626 2.832456
H -5.224493 1.823449 -0.224787
B -1.592305 0.039572 -0.273609
C -0.419940 1.298679 -0.527172
C 0.968696 0.795671 -1.066964
C 0.907326 0.376222 -2.537409
H 1.816276 -0.163967 -2.829199
H 0.039033 -0.250432 -2.748657
C 2.085087 1.835351 -0.945777
H 1.759134 2.765877 -1.430527
H 2.991410 1.495630 -1.462197
O -2.798832 0.378718 -1.051216
C -0.169546 2.124454 0.745429
H -1.113090 2.466768 1.176531
H 0.421646 3.029688 0.537612
H 2.354913 2.067673 0.088793
C -0.987637 2.288774 -1.565523
H -1.316881 1.789642 -2.483318
H -1.874439 2.782299 -1.149071
H -0.266144 3.078525 -1.832737
H 0.840837 1.273639 -3.169834
H 0.361971 1.548108 1.516679
Se 1.540919 -0.845589 0.010545
C 3.398430 -0.442486 0.370458
C 3.775167 0.209032 1.552105
C 4.398994 -0.859027 -0.517218
C 5.119748 0.457847 1.830099
H 3.005199 0.528054 2.255842
C 5.744335 -0.608925 -0.242197
H 4.118756 -1.381886 -1.432498
C 6.108060 0.050328 0.932659
H 5.396008 0.969049 2.753037
H 6.510769 -0.934943 -0.946358
H 7.159036 0.242989 1.150216
O -0.990651 -1.245248 -0.784971
C -1.404786 -1.957373 -1.891179
H -0.533883 -2.281387 -2.490664
H -1.943686 -2.883179 -1.609443
H -2.073051 -1.375204 -2.548747
59

H’_b_conf_6 Eopt -3396.644794

C	-2.397497	-1.374145	1.721011
C	-1.733949	-1.169439	0.347523
C	-4.122652	0.493568	-0.175172
C	-4.596032	-0.061251	1.182296
H	-2.002937	-0.636537	2.431331
H	-5.681451	-0.244332	1.118759
C	-2.294672	-2.117457	-0.731916
H	-2.214547	-3.163518	-0.382107
C	-4.239160	-0.470029	-1.372685
H	-3.729564	0.017836	-2.213890
H	-0.697576	-1.519832	0.460362
H	-4.841123	1.302791	-0.403673
C	-3.738407	-1.910756	-1.227859
H	-3.830444	-2.389682	-2.215509
H	-4.441612	-2.457581	-0.588549
C	-3.929250	-1.313264	1.769766
H	-4.239103	-1.384779	2.824161
H	-4.336439	-2.212344	1.294311
H	-1.623867	-2.029992	-1.596016
H	-5.305507	-0.521181	-1.645058
H	-2.107815	-2.360006	2.128066
H	-4.464933	0.771629	1.891127
B	-1.607840	0.403383	-0.167299
C	-0.487766	1.415814	0.647683
C	0.954629	0.853423	0.880297
C	1.996276	1.948332	1.136571
H	1.650753	2.594535	1.956058
H	2.952801	1.510929	1.447967
C	1.039842	-0.127723	2.051181
H	0.957366	0.431030	2.994722
H	2.011567	-0.635866	2.066888
O	-2.884604	1.130172	-0.065780
C	-1.055000	1.873623	2.006040
H	-1.195685	1.060757	2.725229
H	-2.036133	2.330787	1.831072
H	0.249018	-0.881654	2.033935
C	-0.398072	2.699584	-0.198772
H	-1.406542	3.015323	-0.491353
H	0.056233	3.531532	0.362392
H	0.191544	2.550501	-1.112102
H	2.176732	2.575464	0.257211
H	-0.415264	2.630143	2.490597
Se	1.535501	-0.114749	-0.820156
C	3.380518	-0.437043	-0.348783
C	4.383114	0.470503	-0.716317
C	3.748604	-1.618063	0.309202
C	5.720141	0.215024	-0.410890
H	4.109508	1.385593	-1.242528
C	5.085821	-1.874023	0.617027
H	2.978654	-2.341665	0.579365
C	6.074651	-0.957820	0.258010
H	6.488013	0.933414	-0.700068
H	5.354860	-2.796336	1.132974
H	7.119894	-1.158935	0.494562
O	-1.163680	0.288233	-1.603629
C	-1.506555	1.181751	-2.600040
H	-2.275449	1.903700	-2.278457
H	-0.632886	1.758599	-2.959264
H	-1.907140	0.643308	-3.480475

59

H’_b_conf_7 Eopt -3396.640096

C	-4.071743	-0.885971	1.003026
C	-3.178508	-0.922053	-0.256598
C	-2.588290	1.907184	0.497602
C	-3.468152	1.512891	1.698995
H	-3.566590	-1.348633	1.856738
H	-3.935447	2.436023	2.077343
C	-3.944476	-0.247411	-1.431056
H	-5.025585	-0.238811	-1.207877
C	-3.429714	2.250056	-0.758346
H	-2.990140	3.139444	-1.232991
H	-3.117541	-1.993191	-0.513005
H	-2.111496	2.853936	0.807757
C	-3.504515	1.167434	-1.839553
H	-2.506747	1.088056	-2.284778
H	-4.183711	1.526908	-2.629166
C	-4.568131	0.491760	1.434450
H	-5.174866	0.379022	2.346227
H	-5.252581	0.893602	0.672427
H	-3.846819	-0.869345	-2.332653
H	-4.439901	2.551175	-0.439270
H	-4.964372	-1.508736	0.812992
H	-2.797154	1.152708	2.494181
B	-1.604509	-0.371800	-0.180346
C	-0.520783	-1.297256	0.758524
C	0.896854	-0.638320	0.951840
C	0.885087	0.523727	1.947492
H	0.100977	1.241682	1.695310
H	0.708687	0.136596	2.962261
C	1.956443	-1.643332	1.420463
H	2.212068	-2.381476	0.652392
H	1.590867	-2.182560	2.305199
O	-1.528030	1.033607	0.264611
C	-0.342370	-2.675151	0.098747
H	0.259298	-2.614079	-0.817677
H	-1.318278	-3.100837	-0.168210
H	2.877734	-1.127006	1.715493
C	-1.090160	-1.560041	2.164506
H	-0.340654	-1.968814	2.860189
H	-1.510928	-0.655292	2.621384
H	-1.888730	-2.308744	2.101065
H	1.860750	1.025920	1.965212
H	0.143805	-3.398412	0.772079
Se	1.556743	0.093548	-0.836794
C	3.408730	0.381816	-0.353082
C	3.819643	1.601175	0.201614
C	4.377544	-0.596621	-0.615259
C	5.161562	1.825826	0.514916
H	3.079729	2.381028	0.384787
C	5.719289	-0.373273	-0.305693
H	4.072657	-1.543713	-1.061875
C	6.114515	0.838556	0.263639
H	5.462981	2.779317	0.950152
H	6.458962	-1.147573	-0.512690
H	7.163266	1.014656	0.504717
O	-1.025535	-0.376467	-1.575526
C	-1.237348	-1.325805	-2.553013
H	-0.276978	-1.739908	-2.916376
H	-1.852849	-2.179666	-2.225019
H	-1.741455	-0.883174	-3.435568

59

H’_b_conf_8 Eopt -3396.642708

C	-3.993792	-0.441822	1.277475
C	-3.184619	-0.948250	0.045514
C	-2.505314	1.959986	-0.124470
C	-3.509805	2.142018	1.040961
H	-3.936551	-1.188359	2.082855
H	-4.492214	2.452495	0.655632
C	-4.023782	-0.706805	-1.225961
H	-4.965191	-1.281341	-1.155947
C	-3.215629	1.730349	-1.474901
H	-2.457506	1.415738	-2.204022
H	-3.140973	-2.046543	0.152011
H	-1.979378	2.922845	-0.246258
C	-4.390562	0.752386	-1.487848
H	-4.892826	0.821697	-2.465287
H	-5.140203	1.078180	-0.751166
C	-3.627214	0.903008	1.928693
H	-2.663224	0.794863	2.435157
H	-4.368295	1.109122	2.717599
H	-3.499175	-1.091560	-2.106590
H	-3.594421	2.714735	-1.793029
H	-5.063881	-0.407132	1.005256
H	-3.163505	2.966949	1.681209
B	-1.608630	-0.398833	-0.107241
C	-0.500773	-1.248488	0.872169
C	0.906166	-0.563382	1.017572
C	0.888827	0.655301	1.941724
H	1.849133	1.185227	1.898855

H 0.076549 1.334494 1.672240
C 1.987316 -1.519955 1.533068
H 2.904252 -0.972430 1.782392
H 2.244346 -2.303478 0.811865
O -1.514829 1.035025 0.218546
C -0.307179 -2.670506 0.316306
H 0.171665 -3.332169 1.054934
H 0.310874 -2.684483 -0.589454
H 1.637522 -2.003609 2.455748
C -1.073277 -1.447906 2.287208
H -1.970308 -2.077708 2.228586
H -0.367169 -1.969043 2.953798
H -1.352780 -0.509749 2.774344
H 0.754371 0.322814 2.982153
H -1.275573 -3.124238 0.070842
Se 1.507950 0.064677 -0.827339
C 3.373906 0.366958 -0.424953
C 3.805558 1.608977 0.059109
C 4.328886 -0.630752 -0.663712
C 5.156311 1.838261 0.327533
H 3.075033 2.401914 0.222360
C 5.679350 -0.402257 -0.399301
H 4.006217 -1.596267 -1.054754
C 6.096230 0.832750 0.100582
H 5.474660 2.809396 0.708190
H 6.408793 -1.191102 -0.586519
H 7.151765 1.012432 0.306897
O -1.153456 -0.492909 -1.544773
C -1.214401 -1.600162 -2.368768
H -1.654394 -1.327703 -3.346898
H -0.207021 -2.006377 -2.581726
H -1.818683 -2.429619 -1.963736
59
H’_b_conf_9 Eopt -3396.641928
C -3.956343 1.435904 -0.032550
C -3.149259 0.535988 0.918132
C -2.533606 -1.094389 -1.470852
C -3.294108 0.036420 -2.188949
H -3.420247 2.385816 -0.157145
H -3.817509 -0.412762 -3.048457
C -3.957223 -0.694412 1.373938
H -4.857773 -0.360501 1.921569
C -3.404855 -2.135482 -0.742457
H -2.701762 -2.836982 -0.270777
H -3.035500 1.126500 1.843012
H -2.064301 -1.665607 -2.292788
C -4.436059 -1.676085 0.295101
H -4.817478 -2.578080 0.799533
H -5.306216 -1.255234 -0.222271
C -4.325116 0.883026 -1.424854
H -4.587313 1.735639 -2.070899
H -5.251857 0.304105 -1.343584
H -3.342416 -1.236184 2.104651
H -3.939436 -2.713185 -1.513791
H -4.909012 1.714375 0.455369
H -2.516149 0.685546 -2.614268
B -1.576531 0.134118 0.539096
C -0.528152 1.474298 0.409778
C 1.002396 1.148995 0.583531
C 1.914868 2.266146 0.063960
H 1.868394 2.385918 -1.023595
H 1.626446 3.220638 0.526936
C 1.407570 0.862732 2.030237
H 0.746178 0.120424 2.482817
H 1.351621 1.793920 2.613733
O -1.459436 -0.627241 -0.711894
C -0.901379 2.506025 1.491058
H -0.980927 2.045557 2.484985
H -1.876824 2.950984 1.256441
H 2.449498 0.521884 2.079431
C -0.726871 2.143398 -0.960038
H -1.789406 2.241422 -1.196946
H -0.292572 3.155649 -0.996862
H -0.274536 1.547014 -1.763353
H 2.958574 2.073764 0.341373
H -0.182520 3.338528 1.559365
Se 1.427007 -0.509694 -0.520070
C 3.350830 -0.472310 -0.379701
C 4.129277 0.165326 -1.355087
C 3.997487 -1.131177 0.674291
C 5.521335 0.166738 -1.264538
H 3.636439 0.668593 -2.187425
C 5.390188 -1.128560 0.767153
H 3.401478 -1.649208 1.426545
C 6.155378 -0.478720 -0.201593
H 6.112567 0.671847 -2.029178
H 5.877718 -1.641712 1.596670
H 7.243519 -0.478792 -0.131312
O -1.089619 -0.683927 1.708533
C -0.858054 -2.042906 1.652862
H 0.044565 -2.299497 2.240294
H -1.695316 -2.634518 2.080494
H -0.690908 -2.403227 0.626666
12
PhSe_anion Eopt -2633.103034
Se 1.859526 -0.000001 -0.000035
C -0.055338 -0.000052 0.000528
C -0.787100 -1.202579 -0.000075
C -0.787049 1.202561 -0.000074
C -2.181575 -1.201849 0.000036
H -0.249146 -2.151457 -0.000434
C -2.181498 1.201893 0.000050
H -0.248964 2.151361 -0.000294
C -2.892225 0.000026 -0.000084
H -2.717305 -2.152858 0.000006
H -2.717246 2.152890 -0.000185
H -3.982525 0.000097 -0.000175
48
TS-IV-Gauche_b_conf_1 Eopt -
1061.284192
C -0.239239 1.773154 -0.017360
C -1.477034 1.371676 0.672768
I -2.520665 -0.967643 -0.400930
C 0.315409 2.906136 0.901923
H 1.189477 3.356350 0.426744
H -0.429166 3.707627 1.041344
H 0.618130 2.545469 1.889494
C -1.419402 1.016946 2.130452
H -0.454492 0.617288 2.438380
H -1.597042 1.952601 2.687200
H -2.221732 0.325626 2.403770
C 1.256526 -1.772093 -0.907614
C 2.175819 0.335622 1.022655
H 0.799292 -2.323838 -1.745265
C 0.915307 -2.565128 0.379637
C 2.767482 -1.750523 -1.212453
H 2.325569 1.281865 1.565255
C 1.672131 -0.696194 2.066530
C 3.596365 -0.081114 0.571467
H 1.719513 -3.283882 0.598175
H 0.015102 -3.165413 0.187452
C 0.609787 -1.694435 1.598495
H 2.932992 -1.006890 -2.005246
H 3.023098 -2.737101 -1.629725
C 3.705984 -1.477543 -0.037810
H 1.258074 -0.170051 2.939951
H 2.534308 -1.252992 2.470914
H 3.993668 0.638338 -0.152879
H 4.270059 -0.043611 1.445709
H -0.298442 -1.137543 1.351507
H 4.742501 -1.631794 -0.375000
B 1.206653 0.648848 -0.294835
H 3.536173 -2.234624 0.742120
O 0.645525 -0.513993 -0.927426
H 0.351319 -2.356007 2.440734
C -0.569131 2.362866 -1.391314
H -1.072367 1.610162 -2.015519
H -1.225538 3.244146 -1.319161
H 0.341265 2.663921 -1.911549
C -2.768586 2.066442 0.328285

H -3.604262 1.667835 0.910558
H -2.617440 3.122653 0.620792
H -3.017615 2.043105 -0.732833
O 2.011939 1.345261 -1.330332
C 2.801870 2.464707 -1.137617
H 3.706337 2.393883 -1.766295
H 2.285528 3.399056 -1.434060
H 3.138216 2.601390 -0.095302
48
TS-IV-Gauche_b_conf_2 Eopt -
1061.291217
C 0.332601 1.552964 0.596487
C 1.495084 1.523241 -0.289903
I 2.651273 -1.041661 -0.304925
C -0.460854 2.823404 0.183698
H -1.254132 2.987321 0.920363
H 0.191684 3.712548 0.202720
H -0.910873 2.755442 -0.807026
C 1.283711 1.656474 -1.766947
H 0.394653 1.110752 -2.090830
H 1.143386 2.733149 -1.968190
H 2.167130 1.332564 -2.324011
C -1.820004 -1.080418 -1.465235
C -2.333755 0.567218 0.987248
H -1.334147 -1.667302 -2.262033
C -2.906403 -0.206929 -2.137874
C -2.421883 -2.129618 -0.509637
H -2.224305 1.228567 1.864339
C -3.249054 1.329531 -0.018411
C -3.103552 -0.665364 1.509266
H -3.877533 -0.722323 -2.116347
H -2.655013 -0.083281 -3.201394
C -3.008312 1.193121 -1.532506
H -1.598820 -2.571957 0.066653
H -2.838057 -2.922554 -1.150393
C -3.532347 -1.660530 0.431807
H -3.210613 2.406821 0.196196
H -4.296387 1.043487 0.181426
H -2.506664 -1.199979 2.254897
H -4.014086 -0.325452 2.033557
H -2.079057 1.709340 -1.795099
H -3.968977 -2.543990 0.922111
B -0.808500 0.138664 0.464948
H -4.348041 -1.223926 -0.163410
O -0.804407 -0.275881 -0.926813
H -3.814883 1.736817 -2.049111
C 0.727928 1.740023 2.060164
H 1.415848 0.957309 2.399930
H 1.212727 2.715473 2.226590
H -0.162154 1.714284 2.699589
C 2.794700 2.106762 0.181182
H 3.592774 1.974302 -0.554424
H 2.595669 3.191181 0.287221
H 3.121983 1.733305 1.152808
O -0.242713 -0.955272 1.255819
C -0.188482 -1.082641 2.631466
H -0.583326 -2.068835 2.936894
H 0.852636 -1.034226 2.998185
H -0.765672 -0.318329 3.178214
48
TS-IV-Gauche_b_conf_3 Eopt -
1061.288925
C -0.387564 1.758053 0.098924
C -1.586433 1.243061 0.779994
I -2.412774 -1.082607 -0.461611
C 0.084694 2.934905 1.006628
H 0.995897 3.343935 0.554583
H -0.674271 3.733311 1.050134
H 0.316229 2.626507 2.030479
C -1.481534 0.816450 2.214883
H -0.477382 0.502044 2.493556
H -1.751165 1.694065 2.825681
H -2.205535 0.030440 2.449875
C 1.533355 -1.548179 -0.912097
C 2.071465 0.488589 1.217352
H 1.204245 -2.087073 -1.815744
C 1.182878 -2.457892 0.292054
C 3.053330 -1.363202 -1.092361
H 2.060553 1.408762 1.821442
C 1.659916 -0.680350 2.151027
C 3.554826 0.305773 0.823598
H 2.040375 -3.110308 0.517934
H 0.362654 -3.125152 -0.008136
C 0.716983 -1.720578 1.547111
H 3.212573 -0.603668 -1.869707
H 3.434731 -2.311616 -1.502295
C 3.880583 -1.026250 0.148263
H 1.179839 -0.287651 3.059531
H 2.563518 -1.189608 2.524843
H 3.853594 1.129537 0.163381
H 4.180283 0.377746 1.730969
H -0.220068 -1.221037 1.283412
H 4.944216 -1.029089 -0.135992
B 1.156927 0.801810 -0.141967
H 3.771122 -1.843511 0.876898
O 0.792724 -0.357806 -0.915544
H 0.466877 -2.470686 2.314343
C -0.753814 2.343171 -1.267527
H -1.082418 1.552915 -1.958570
H -1.556558 3.092153 -1.199821
H 0.109858 2.848530 -1.701910
C -2.931984 1.855148 0.486631
H -3.725336 1.363876 1.057858
H -2.855604 2.903049 0.832748
H -3.197302 1.866121 -0.570590
O 1.972449 1.772440 -0.925117
C 2.140059 1.689869 -2.298559
H 1.889520 2.648369 -2.787724
H 3.193990 1.473421 -2.557252
H 1.515988 0.905282 -2.754978
48
TS-V-Rotation_b_conf_1 Eopt -
1061.286719
C -0.387204 0.837058 -0.414669
C -1.493598 0.590164 0.691530
I -3.480673 -0.316082 -0.174793
C -0.035251 2.337275 -0.433043
H 0.759465 2.503469 -1.166568
H -0.885672 2.960923 -0.748892
H 0.317068 2.714477 0.533125
C -1.199767 -0.432709 1.776547
H -1.071862 -1.437108 1.378432
H -0.258462 -0.135950 2.256784
H -2.007083 -0.439171 2.518326
C 2.940672 -0.100571 1.483617
C 2.175607 0.078910 -1.391271
H 2.939281 -0.227571 2.580067
C 3.763333 1.180799 1.196251
C 3.669690 -1.352049 0.959096
H 1.629982 0.123911 -2.344650
C 3.055298 1.356508 -1.328992
C 3.074178 -1.165908 -1.548119
H 4.817913 0.907387 1.039760
H 3.748254 1.817353 2.093238
C 3.245697 2.029187 0.036946
H 3.037076 -2.229363 1.130695
H 4.548992 -1.480031 1.610605
C 4.159707 -1.327743 -0.486547
H 2.633731 2.124158 -1.995914
H 4.050605 1.138595 -1.754711
H 2.436797 -2.060172 -1.566079
H 3.581654 -1.126567 -2.528613
H 2.284740 2.440167 0.354148
H 4.716882 -2.257656 -0.679814
B 1.011895 -0.156912 -0.210741
H 4.895585 -0.516658 -0.597557
O 1.586643 0.030617 1.139877
H 3.922315 2.889646 -0.088957
C -0.903896 0.561456 -1.837696
H -1.080632 -0.505533 -2.007194

H -1.832956 1.112734 -2.042646
H -0.162917 0.907549 -2.568067
C -2.035391 1.848994 1.362779
H -2.841211 1.596410 2.058906
H -1.219244 2.300213 1.946804
H -2.406985 2.594674 0.653884
O 0.446483 -1.540634 -0.419878
C 0.693522 -2.637804 0.377831
H -0.203562 -3.282956 0.415410
H 1.518287 -3.271888 -0.005236
H 0.947550 -2.365397 1.416911
48
TS-V-Rotation_b_conf_2 Eopt - 1061.277710
C -0.438668 1.029293 0.522168
C -1.409817 -0.179960 0.867896
I -3.280085 -0.308230 -0.547427
C -0.224905 1.882223 1.794676
H 0.480018 2.686895 1.557725
H -1.147817 2.365222 2.148839
H 0.205813 1.316200 2.630628
C -0.912728 -1.606749 0.690305
H -0.639584 -1.822431 -0.339779
H -0.017288 -1.743879 1.304731
H -1.673711 -2.316042 1.037446
C 2.016927 -1.342984 -1.345095
C 2.357869 0.473877 0.966801
H 1.680385 -1.896085 -2.240883
C 2.185021 -2.447819 -0.277101
C 3.326273 -0.653839 -1.771788
H 2.248260 1.305088 1.681895
C 2.258501 -0.787347 1.831796
C 3.786683 0.669242 0.410026
H 2.894973 -3.184236 -0.689151
H 1.219651 -2.967249 -0.233486
C 2.644486 -2.123123 1.164479
H 3.039404 0.251144 -2.325109
H 3.840834 -1.323541 -2.478671
C 4.311210 -0.298822 -0.661199
H 1.248463 -0.842192 2.260318
H 2.925766 -0.685211 2.706787
H 3.849968 1.677524 -0.015600
H 4.504334 0.655157 1.251629
H 2.286166 -2.940262 1.810162
H 5.200992 0.157174 -1.123195
B 1.139244 0.679066 -0.153741
H 4.673892 -1.228103 -0.204202
O 0.975255 -0.460956 -1.053369
H 3.737115 -2.198583 1.206297
C -1.113776 1.994110 -0.470078
H -1.180379 1.567666 -1.477127
H -2.126409 2.266712 -0.136667
H -0.534530 2.917000 -0.542118
C -2.099889 -0.070365 2.223891
H -2.810497 -0.890786 2.364821
H -1.330605 -0.154710 3.006627
H -2.628212 0.877913 2.362632
O 1.532152 1.843852 -1.031380
C 1.957476 3.073510 -0.568478
H 2.778894 3.455939 -1.202032
H 1.160559 3.843546 -0.600710
H 2.334605 3.060361 0.468745
48
TS-V-Rotation_b_conf_3 Eopt - 1061.275495
C -0.493145 0.862691 0.728085
C -1.473906 -0.360936 0.822021
I -3.361219 -0.189038 -0.572304
C -0.377928 1.537908 2.114572
H 0.410688 2.296568 2.050361
H -1.308687 2.047168 2.410591
H -0.104879 0.847393 2.919943
C -0.914552 -1.670614 0.330846
H -0.470871 -1.573244 -0.661511
H -0.120645 -1.929487 1.040861
H -1.661052 -2.472455 0.350438
C 2.462444 -0.507245 -1.629644
C 2.175273 -0.135568 1.311959
H 2.362222 -0.413783 -2.724875
C 2.717199 -2.001523 -1.339252
C 3.735016 0.306438 -1.293523
H 1.787385 0.105084 2.314200
C 2.336104 -1.687198 1.303977
C 3.588854 0.479301 1.282111
H 3.793362 -2.199221 -1.453412
H 2.222425 -2.600835 -2.117561
C 2.203522 -2.524071 0.009344
H 3.546206 1.373303 -1.430525
H 4.459602 0.021628 -2.073111
C 4.405400 0.064714 0.060117
H 1.616653 -2.127370 2.012210
H 3.318358 -1.915733 1.751349
H 3.512239 1.572097 1.315718
H 4.146645 0.162006 2.181594
H 1.150715 -2.765546 -0.140487
H 5.368512 0.598525 0.066743
B 1.166499 0.610848 0.193308
H 4.661866 -1.003135 0.144134
O 1.228219 -0.067919 -1.108679
H 2.690905 -3.493614 0.197628
C -1.064232 1.974046 -0.169931
H -1.035472 1.716251 -1.236742
H -2.103612 2.204981 0.099543
H -0.485068 2.885940 -0.015527
C -2.144229 -0.579405 2.172582
H -2.843682 -1.420391 2.125510
H -1.361965 -0.835519 2.902825
H -2.677183 0.304604 2.536373
O 1.697295 2.023125 0.065283
C 1.676010 2.745772 -1.114890
H 0.997672 3.616384 -1.064426
H 2.677825 3.158799 -1.337994
H 1.366870 2.136836 -1.981343
48
TS-V-Rotation_b_conf_4 Eopt - 1061.288806
C 0.367949 0.571524 0.721967
C 1.435878 0.812632 -0.423311
I 3.459451 -0.325391 -0.033002
C 0.031674 1.943898 1.354323
H -0.641894 1.791849 2.201721
H 0.923519 2.443306 1.758865
H -0.458235 2.640976 0.665279
C 1.117633 0.324642 -1.826317
H 0.815045 -0.721100 -1.864191
H 0.265618 0.915950 -2.185211
H 1.974784 0.488611 -2.488974
C -2.772004 -0.192786 -1.589621
C -2.353948 0.136505 1.347102
H -2.654652 -0.404712 -2.667174
C -3.567269 1.133928 -1.480434
C -3.546898 -1.408453 -1.038999
H -1.905526 0.235208 2.348762
C -3.102459 1.470566 1.069169
C -3.373262 -1.007022 1.507755
H -4.642786 0.930220 -1.369582
H -3.459972 1.688417 -2.424351
C -3.066512 2.039166 -0.355599
H -2.847519 -2.254457 -0.981701
H -4.300821 -1.663241 -1.800875
C -4.272549 -1.240942 0.295885
H -2.706849 2.257750 1.726911
H -4.160606 1.361338 1.366555
H -2.829698 -1.931611 1.737098
H -4.028331 -0.794407 2.372061
H -2.032254 2.312294 -0.599828
H -4.887486 -2.137265 0.471997
B -1.110876 -0.274608 0.302999
H -4.988162 -0.408860 0.209169
O -1.473441 -0.028380 -1.102543

H -3.650431 2.973087 -0.372080
C 0.925283 -0.237018 1.908315
H 1.236060 -1.245364 1.624473
H 1.779076 0.274825 2.379441
H 0.137818 -0.344461 2.665530
C 1.960579 2.242043 -0.550473
H 2.697842 2.305220 -1.357395
H 1.115224 2.893681 -0.816839
H 2.416410 2.624808 0.366568
O -0.904977 -1.735072 0.535283
C -0.144758 -2.482076 -0.341607
H -0.222228 -3.548876 -0.074043
H -0.481786 -2.376791 -1.390732
H 0.932623 -2.219629 -0.318211
48
TS-V-Rotation_b_conf_5 Eopt -
1061.278798
C 0.399985 0.740965 0.867318
C 1.632323 0.992780 -0.096580
I 3.117308 -0.816175 -0.202351
C 0.289500 1.919480 1.873622
H -0.616238 1.751823 2.465975
H 1.143180 1.960603 2.566373
H 0.180719 2.899399 1.399392
C 1.373058 1.146969 -1.588241
H 0.980422 0.235834 -2.041740
H 0.648475 1.953587 -1.742092
H 2.301216 1.416373 -2.106271
C -3.185090 -0.682134 1.259637
C -1.578092 -0.133935 -1.194950
H -3.614862 -0.677379 2.276129
C -2.953192 -2.171588 0.890202
C -4.260969 -0.022778 0.375858
H -0.877083 0.244863 -1.943886
C -1.394182 -1.669362 -1.191832
C -2.945331 0.257407 -1.798463
H -3.826923 -2.568168 0.352713
H -2.886285 -2.754958 1.820503
C -1.659705 -2.433223 0.116077
H -4.209607 1.061956 0.546671
H -5.237777 -0.370596 0.747847
C -4.181558 -0.316062 -1.118355
H -0.351618 -1.902121 -1.467401
H -2.009009 -2.111562 -1.996751
H -3.035407 1.351451 -1.798573
H -2.966937 -0.058017 -2.857117
H -0.842404 -2.211238 0.806166
H -5.079340 0.090245 -1.609763
B -1.258666 0.641379 0.242864
H -4.222254 -1.403404 -1.283496
O -1.985726 0.022627 1.370286
H -1.595587 -3.512275 -0.095839
C 0.689133 -0.452842 1.797741
H 0.785477 -1.400878 1.263251
H 1.630362 -0.295065 2.348893
H -0.125414 -0.546762 2.521678
C 2.577139 2.100523 0.357139
H 3.448985 2.162802 -0.301612
H 2.038283 3.057783 0.293486
H 2.923152 1.969954 1.387244
O -1.811484 2.038328 0.162907
C -1.368537 2.964757 -0.754207
H -2.040376 3.839599 -0.744603
H -0.349111 3.345796 -0.536825
H -1.347222 2.587368 -1.795421
48
TS-V-Rotation_b_conf_6 Eopt -
1061.275581
C 0.428220 0.188299 0.999734
C 1.616919 0.895389 0.235901
I 3.301590 -0.603492 -0.392097
C 0.348733 0.747434 2.438276
H -0.482250 0.250791 2.946648
H 1.262661 0.550426 3.019534
H 0.142800 1.823400 2.456891
C 1.328101 1.551702 -1.105409
H 0.833888 0.897138 -1.823276
H 0.675029 2.405686 -0.901878
H 2.253559 1.926934 -1.555827
C -3.417527 -0.316641 1.103566
C -1.446625 -0.345858 -1.126280
H -3.945825 -0.107220 2.050406
C -3.643009 -1.816676 0.790769
C -4.102225 0.602551 0.072021
H -0.538368 -0.299845 -1.741327
C -1.762542 -1.874261 -1.051510
C -2.535799 0.316200 -1.991379
H -4.520339 -1.942757 0.138743
H -3.886198 -2.342859 1.725586
C -2.410518 -2.495046 0.195427
H -3.764791 1.627041 0.270680
H -5.178927 0.575544 0.303414
C -3.949414 0.245520 -1.409232
H -0.832461 -2.437778 -1.216659
H -2.396655 -2.126857 -1.919945
H -2.277393 1.355581 -2.203905
H -2.562119 -0.183203 -2.976201
H -1.661656 -2.546041 0.989109
H -4.612764 0.899010 -1.997064
B -1.135848 0.454525 0.317173
H -4.340045 -0.772314 -1.558579
O -2.070688 -0.048681 1.356851
H -2.669210 -3.538343 -0.046641
C 0.633828 -1.318075 1.195121
H 0.650954 -1.879052 0.253441
H 1.569156 -1.533345 1.733068
H -0.190704 -1.691383 1.809139
C 2.418517 1.897968 1.060028
H 3.237071 2.317648 0.465839
H 1.741477 2.720840 1.333102
H 2.833865 1.472843 1.977572
O -1.243006 1.964231 0.306482
C -1.647740 2.927978 -0.590273
H -1.415551 3.921343 -0.166850
H -1.140302 2.880048 -1.572765
H -2.733901 2.925855 -0.804215
48
TS-V-Rotation_b_conf_7 Eopt -
1061.291238
C 0.408012 0.508771 0.872118
C 1.606136 0.930946 -0.063358
I 3.258465 -0.721214 -0.201381
C 0.335267 1.456012 2.094027
H -0.569428 1.206088 2.657353
H 1.196859 1.324138 2.768214
H 0.274861 2.517799 1.837138
C 1.323311 1.155403 -1.543420
H 0.793578 0.326475 -2.016147
H 0.681629 2.040532 -1.625240
H 2.257544 1.340011 -2.084989
C -3.481872 0.182366 0.890081
C -1.342020 -0.843628 -0.923080
H -4.072001 0.780484 1.606687
C -3.789699 -1.305821 1.187801
C -4.022538 0.606510 -0.495070
H -0.365457 -1.077822 -1.374820
C -1.819134 -2.194265 -0.305834
C -2.266387 -0.474808 -2.096828
H -4.622487 -1.652622 0.557395
H -4.138633 -1.397551 2.227070
C -2.572199 -2.215037 1.032591
H -3.688462 1.629493 -0.705739
H -5.117768 0.644805 -0.377568
C -3.733372 -0.305272 -1.693046
H -0.948612 -2.852674 -0.169411
H -2.444233 -2.714920 -1.052814
H -1.906801 0.451088 -2.562068
H -2.224823 -1.263792 -2.869257
H -1.873898 -1.953686 1.832743
H -4.302796 0.073598 -2.556221

B -1.128171 0.476324 0.085104
H -4.157273 -1.299179 -1.481244
O -2.140511 0.481051 1.168715
H -2.886157 -3.253076 1.227454
C 0.586627 -0.874915 1.512758
H 0.608206 -1.688508 0.780298
H 1.509885 -0.928258 2.108491
H -0.252787 -1.043611 2.194608
C 2.432599 2.113078 0.434550
H 3.276366 2.304115 -0.236752
H 1.791006 3.006338 0.425561
H 2.814428 1.972330 1.450020
O -1.328670 1.691359 -0.759276
C -1.425499 2.935120 -0.166053
H -0.438500 3.424390 -0.034423
H -2.026228 3.606848 -0.804110
H -1.907751 2.893842 0.826515
48
TS-V-Rotation_b_conf_8 Eopt -1061.289819
C 0.349983 0.652271 0.560613
C 1.474062 0.756434 -0.554411
I 3.470946 -0.290015 0.059610
C -0.100142 2.080197 0.934755
H -0.880159 2.005533 1.698609
H 0.711878 2.677124 1.375502
H -0.519905 2.626249 0.084131
C 1.199697 0.082783 -1.890817
H 1.084626 -0.997890 -1.812313
H 0.241642 0.479932 -2.254353
H 1.996892 0.322765 -2.604638
C -2.978483 0.456391 -1.471509
C -2.180336 -0.478081 1.256222
H -2.988819 0.764437 -2.531498
C -3.927539 1.423772 -0.720705
C -3.520818 -0.988067 -1.480889
H -1.623742 -0.760899 2.163117
C -3.108852 0.693078 1.686149
C -3.045158 -1.718899 0.950405
H -4.943828 1.003717 -0.689129
H -4.008165 2.357403 -1.297207
C -3.442830 1.807143 0.678558
H -2.726686 -1.645984 -1.856038
H -4.339424 -1.002200 -2.218255
C -4.072798 -1.533581 -0.163472
H -2.671324 1.200259 2.560769
H -4.058705 0.270644 2.059856
H -2.399360 -2.566587 0.691289
H -3.592693 -2.012728 1.864106
H -2.551920 2.424682 0.538454
H -4.560703 -2.500456 -0.362766
B -1.058185 -0.198542 0.031814
H -4.875859 -0.870094 0.192192
O -1.651720 0.613217 -1.051988
H -4.200766 2.461809 1.138075
C 0.876154 0.093084 1.896201
H 1.187926 -0.953626 1.852080
H 1.735159 0.679983 2.256540
H 0.090963 0.178003 2.657319
C 1.983248 2.169175 -0.830829
H 2.796297 2.147458 -1.563348
H 1.156522 2.750462 -1.265150
H 2.336523 2.685800 0.066520
O -0.706242 -1.521382 -0.569606
C 0.013292 -2.465991 0.134769
H -0.156285 -3.463868 -0.305202
H 1.105726 -2.280883 0.102422
H -0.268186 -2.530456 1.203011
59
TS-VI-Anti_b_conf_1 Eopt -3396.638083
C -2.554223 2.230191 -0.563936
C -2.221163 1.281005 0.615493
C -4.344352 -0.391388 -0.661630
C -4.534403 0.819855 -1.604603
H -1.621400 2.678355 -0.941620
H -5.373734 1.437179 -1.253296
C -3.230555 1.470860 1.764595
H -3.155895 2.503609 2.146531
C -4.954479 -0.142151 0.732987
H -4.635271 -0.952742 1.402649
H -1.271252 1.630967 1.045043
H -4.928439 -1.225176 -1.082886
C -4.685788 1.213323 1.387071
H -5.310682 1.293606 2.289481
H -5.033584 2.012900 0.715817
C -3.271020 1.659180 -1.799063
H -2.564281 1.029532 -2.344433
H -3.514990 2.498731 -2.468418
H -2.948227 0.802574 2.588966
H -6.043493 -0.247648 0.610455
H -3.140478 3.082703 -0.178929
H -4.828467 0.450375 -2.597543
B -2.034965 -0.314846 0.263782
C -0.411121 -0.732311 -0.629758
C 0.654778 -0.124548 0.133460
C 1.107284 1.267837 -0.231994
H 1.909748 1.609005 0.430503
H 1.454048 1.331174 -1.267725
C 0.829738 -0.432312 1.594102
H 1.841527 -0.173657 1.923500
H 0.607363 -1.470770 1.847384
O -3.011183 -0.846940 -0.667151
C -0.460833 -0.268168 -2.078038
H -1.312210 -0.734244 -2.587492
H 0.462685 -0.571544 -2.601421
H 0.117025 0.201982 2.144973
C -0.425265 -2.256511 -0.603811
H -1.398701 -2.639956 -0.931999
H 0.339706 -2.633795 -1.303140
H -0.204049 -2.668750 0.384363
H 0.261578 1.960163 -0.122829
H -0.555945 0.818689 -2.179527
Se 2.749223 -1.385008 -0.696728
C 3.989253 -0.068069 -0.092227
C 4.412130 0.970154 -0.940911
C 4.474801 -0.081472 1.226823
C 5.274501 1.964907 -0.482644
H 4.051920 0.995930 -1.969918
C 5.335884 0.916425 1.684845
H 4.171537 -0.885737 1.897975
C 5.739364 1.946467 0.834263
H 5.585109 2.761264 -1.160544
H 5.695654 0.884814 2.714268
H 6.411822 2.726161 1.192668
O -1.981967 -1.050897 1.523552
C -2.481727 -2.334392 1.696447
H -3.196845 -2.353234 2.538389
H -3.003379 -2.708623 0.802252
H -1.678769 -3.049943 1.946306
59
TS-VI-Anti_b_conf_10 Eopt -3396.637296
C 3.465308 -1.506296 0.936129
C 2.587582 -0.264740 1.238340
C 3.888315 0.753612 -1.254060
C 4.720480 -0.548325 -1.182894
H 2.895022 -2.418865 1.171493
H 5.692563 -0.344044 -0.711362
C 3.412151 0.815382 1.966066
H 3.757446 0.416726 2.935508
C 4.354574 1.800128 -0.221546
H 3.621368 2.618463 -0.200518
H 1.845787 -0.574763 1.988821
H 4.088516 1.213014 -2.235207
C 4.634420 1.311946 1.200117
H 5.101857 2.133698 1.763544
H 5.389894 0.512670 1.164316
C 4.000718 -1.706702 -0.491301
H 3.162267 -1.978635 -1.136972

H 4.676687 -2.575829 -0.481843
H 2.753654 1.666343 2.185082
H 5.287732 2.225985 -0.621473
H 4.317751 -1.518909 1.637406
H 4.946825 -0.877976 -2.207106
B 1.757043 0.391569 -0.020833
C 0.247180 -0.625641 -0.585629
C -0.528906 -0.920520 0.597999
C -0.237837 -2.204774 1.343776
H 0.815577 -2.214522 1.654489
H -0.862923 -2.288040 2.238516
C -1.013267 0.190258 1.485916
H -0.156142 0.507768 2.098968
H -1.806855 -0.161901 2.152143
O 2.505882 0.483148 -1.258819
C 0.684384 -1.867879 -1.347610
H 1.301384 -1.578393 -2.206275
H -0.204407 -2.401936 -1.728731
H -1.358161 1.070063 0.936137
C -0.409149 0.335450 -1.570238
H 0.339387 0.754988 -2.252609
H -1.146517 -0.223377 -2.166730
H -0.933115 1.160150 -1.078827
H -0.399531 -3.090859 0.721733
H 1.259042 -2.572986 -0.736720
Se -2.827035 -1.710954 -0.206622
C -3.791927 -0.064510 -0.093311
C -3.875618 0.811719 -1.188537
C -4.421513 0.314560 1.104357
C -4.549365 2.027990 -1.084492
H -3.399435 0.535948 -2.129340
C -5.095747 1.531624 1.207610
H -4.378078 -0.354844 1.964002
C -5.162167 2.396833 0.114832
H -4.595340 2.691589 -1.948998
H -5.572538 1.803410 2.150467
H -5.686868 3.349018 0.196120
O 1.215159 1.673249 0.418986
C 1.055222 2.778919 -0.405003
H -0.009584 3.048863 -0.516680
H 1.560841 3.656250 0.036641
H 1.472526 2.620154 -1.411140
59
TS-VI-Anti_b_conf_11 Eopt -
3396.636806
C -3.711858 -0.848840 -1.415205
C -2.902444 0.468413 -1.236242
C -3.472841 -0.120094 1.650170
C -4.316295 -1.298443 1.106822
H -3.294080 -1.422042 -2.253572
H -5.376760 -1.013475 1.049989
C -3.888731 1.643697 -1.057763
H -4.497668 1.740984 -1.973229
C -4.166218 1.244595 1.472751
H -3.423940 2.027675 1.683949
H -2.399822 0.657397 -2.199372
H -3.387493 -0.258590 2.739989
C -4.841540 1.512864 0.127961
H -5.429356 2.439474 0.211497
H -5.573926 0.717830 -0.074414
C -3.812815 -1.828487 -0.235557
H -2.823238 -2.259753 -0.048453
H -4.459426 -2.664581 -0.544047
H -3.342924 2.587600 -0.955193
H -4.936365 1.305468 2.257012
H -4.736297 -0.590283 -1.733640
H -4.265397 -2.129499 1.825102
B -1.743715 0.470008 -0.057665
C -0.237428 -0.453047 -0.739154
C 0.464687 -0.865118 0.460168
C 0.941179 0.171168 1.438056
H 1.651149 -0.262639 2.149158
H 1.394232 1.046285 0.963473
C 0.053826 -2.161937 1.111037
H 0.679055 -2.378345 1.983019
H 0.104595 -3.012044 0.423078
O -2.155795 -0.189578 1.168868
C 0.507857 0.558963 -1.600265
H 1.286519 0.027751 -2.168361
H 0.994224 1.349698 -1.023605
H -0.987480 -2.032947 1.439940
C -0.627108 -1.610701 -1.652612
H 0.284517 -2.147882 -1.968141
H -1.300213 -2.339346 -1.195315
H -1.108602 -1.228653 -2.560930
H 0.047790 0.510533 1.982431
H -0.166493 1.028855 -2.327273
Se 2.757175 -1.715911 -0.275504
C 3.785903 -0.125788 -0.013001
C 4.353655 0.158636 1.240436
C 3.976851 0.803626 -1.049274
C 5.071428 1.335617 1.453942
H 4.225054 -0.552648 2.056775
C 4.694612 1.979771 -0.835319
H 3.551239 0.601584 -2.032156
C 5.244323 2.254957 0.418389
H 5.497799 1.533832 2.438377
H 4.824381 2.686035 -1.656461
H 5.802996 3.176119 0.585544
O -1.277589 1.804028 0.323028
C -1.035099 2.890841 -0.504924
H 0.026589 3.189766 -0.468017
H -1.288067 2.713642 -1.561669
H -1.622550 3.763369 -0.167681
59
TS-VI-Anti_b_conf_12 Eopt -
3396.634068
C -3.052959 0.785231 2.012278
C -3.142326 -0.301133 0.925205
C -2.497638 1.939086 -0.949477
C -2.403421 2.702645 0.382698
H -2.099281 0.722174 2.547800
H -2.682863 3.745381 0.163967
C -4.532187 -0.207920 0.226335
H -5.212791 0.396763 0.848081
C -3.960480 1.740205 -1.422057
H -4.005283 1.944166 -2.501375
H -3.140174 -1.252439 1.477202
H -2.025176 2.611210 -1.683725
C -4.548895 0.343181 -1.204777
H -3.997444 -0.353187 -1.848589
H -5.589866 0.354892 -1.563056
C -3.282853 2.219659 1.534129
H -3.148079 2.902131 2.386789
H -4.336104 2.330069 1.235523
H -4.990002 -1.205812 0.192840
H -4.597750 2.500583 -0.944277
H -3.824771 0.571992 2.772240
H -1.347587 2.723821 0.680734
B -1.948035 -0.419926 -0.206246
C -0.270880 -0.744296 0.644588
C 0.545617 -1.347705 -0.392098
C 0.460396 -2.835800 -0.616191
H 1.197824 -3.161127 -1.356486
H 0.608249 -3.410836 0.303607
C 0.839560 -0.577500 -1.648785
H -0.097692 -0.560926 -2.224950
H 1.614002 -1.077271 -2.239336
O -1.731432 0.766062 -1.004280
C -0.506879 -1.660791 1.841059
H -1.075153 -2.562659 1.579592
H -1.065461 -1.135534 2.624492
H 1.134447 0.459094 -1.469002
C 0.264822 0.601118 1.117332
H 0.495459 1.276645 0.287785
H -0.445585 1.114441 1.767547
H 1.184934 0.432800 1.697794
H -0.540652 -3.052621 -1.006239
H 0.454817 -1.979964 2.279457
Se 2.930167 -1.486275 0.502388

C 3.651828 0.227864 0.052217
C 3.799778 1.234781 1.021749
C 4.053961 0.514759 -1.262780
C 4.320454 2.483184 0.686151
H 3.497229 1.032862 2.049681
C 4.570169 1.767042 -1.599238
H 3.959398 -0.254801 -2.028971
C 4.707582 2.758437 -0.627491
H 4.424454 3.246955 1.458142
H 4.869874 1.965065 -2.629329
H 5.112180 3.736227 -0.889892
O -2.184650 -1.539447 -1.128819
C -2.926236 -2.673744 -0.831337
H -2.532638 -3.533221 -1.398728
H -2.906218 -2.952562 0.235061
H -3.985626 -2.564061 -1.126630
59
TS-VI-Anti_b_conf_13 Eopt -3396.634738
C 2.394731 1.355877 -1.722998
C 2.542449 -0.088599 -1.214239
C 3.475824 1.252335 1.256077
C 3.377557 2.525209 0.398116
H 1.364044 1.690074 -1.555877
H 4.214993 3.185790 0.674805
C 3.982269 -0.633087 -1.326977
H 4.324522 -0.557622 -2.374365
C 4.696544 0.348533 1.002096
H 4.528302 -0.568312 1.584668
H 1.981409 -0.715411 -1.925193
H 3.609624 1.621439 2.287653
C 5.076600 -0.014587 -0.437776
H 5.911206 -0.730408 -0.383039
H 5.502189 0.872493 -0.919927
C 3.337642 2.410402 -1.131787
H 3.032441 3.392434 -1.524242
H 4.348069 2.251470 -1.523051
H 3.925268 -1.705216 -1.096880
H 5.568311 0.844791 1.455843
H 2.538949 1.374665 -2.817590
H 2.460703 3.031107 0.738205
B 1.901263 -0.397110 0.262177
C 0.001408 -0.269675 0.358756
C -0.538753 -1.011655 -0.763893
C -0.768170 -0.276197 -2.064393
H -1.203403 -0.941294 -2.816849
H -1.424725 0.590038 -1.936150
C -0.150725 -2.457393 -0.958534
H 0.867287 -2.478209 -1.373590
H -0.823689 -2.948430 -1.669775
O 2.258060 0.553242 1.288140
C -0.417555 1.199023 0.427050
H -0.230801 1.761135 -0.492819
H 0.140440 1.688937 1.233006
H -0.127650 -3.023654 -0.024205
C -0.261832 -0.891755 1.727469
H -0.134333 -1.977751 1.735002
H 0.409732 -0.458875 2.478624
H -1.298413 -0.675292 2.030001
H 0.193781 0.090721 -2.448013
H -1.490624 1.287565 0.653515
Se -2.940333 -1.595952 -0.312685
C -3.756543 0.097856 0.034911
C -4.018307 0.999280 -1.009587
C -4.066403 0.492349 1.347647
C -4.531769 2.269536 -0.744896
H -3.809007 0.701866 -2.037361
C -4.586645 1.757910 1.608981
H -3.874185 -0.197334 2.170089
C -4.811736 2.659154 0.565152
H -4.714165 2.958260 -1.571027
H -4.809722 2.045940 2.637168
H -5.208418 3.653461 0.771592
O 2.225832 -1.780900 0.604699
C 2.520746 -2.214177 1.890052
H 1.792392 -2.967985 2.237089
H 3.514551 -2.697212 1.911469
H 2.530819 -1.392024 2.621536
59
TS-VI-Anti_b_conf_14 Eopt -3396.634468
C 3.645454 -0.361226 -1.895783
C 3.299302 -0.921056 -0.499951
C 2.908074 1.924847 0.220546
C 3.107933 2.120658 -1.290775
H 2.823092 -0.573856 -2.583649
H 3.519045 3.129804 -1.448435
C 4.523780 -0.875037 0.438584
H 5.335501 -1.486931 0.008036
C 4.178079 1.679101 1.056852
H 3.831174 1.591364 2.095895
H 3.117954 -1.997321 -0.662765
H 2.528395 2.895058 0.584420
C 5.127444 0.508531 0.739692
H 5.798801 0.401253 1.605606
H 5.785720 0.797001 -0.086872
C 3.996342 1.128486 -2.041228
H 3.947040 1.381164 -3.111463
H 5.039642 1.306021 -1.759129
H 4.243633 -1.364499 1.379483
H 4.773393 2.604976 1.017193
H 4.504843 -0.933356 -2.289156
H 2.098285 2.126590 -1.730718
B 1.974778 -0.405419 0.347233
C 0.405330 -1.047751 -0.456678
C -0.626841 -0.239306 0.157244
C -0.831229 -0.244857 1.645367
H -0.647424 -1.216824 2.106810
H -0.112029 0.479521 2.058039
C -0.956875 1.089265 -0.461313
H -1.775881 1.584517 0.071170
H -1.214817 1.012510 -1.521896
O 1.872468 1.022066 0.507750
C 0.349909 -2.529608 -0.103795
H -0.452137 -3.011689 -0.688153
H 0.160277 -2.704651 0.957765
H -0.048985 1.703100 -0.359601
C 0.452949 -0.892968 -1.971509
H -0.546266 -1.078154 -2.400274
H 0.778862 0.104322 -2.292693
H 1.136200 -1.630682 -2.407464
H -1.837990 0.105354 1.895568
H 1.291716 -3.031890 -0.358588
Se -2.801356 -1.505997 -0.452611
C -3.949010 -0.021860 -0.114445
C -4.290996 0.876126 -1.141220
C -4.443045 0.233394 1.176586
C -5.083720 1.993252 -0.882973
H -3.922011 0.694328 -2.151203
C -5.233994 1.353891 1.434162
H -4.202560 -0.457671 1.985286
C -5.558141 2.241156 0.407112
H -5.331721 2.676021 -1.696952
H -5.601871 1.530717 2.445893
H -6.176230 3.116296 0.608841
O 1.936472 -1.112864 1.637842
C 2.190844 -0.488184 2.848534
H 3.258169 -0.554487 3.138931
H 1.920416 0.578922 2.842604
H 1.608932 -0.980572 3.645289
59
TS-VI-Anti_b_conf_2 Eopt -3396.639032
C -2.325641 2.292104 0.032433
C -2.277662 1.069940 0.964283
C -4.210042 -0.097504 -0.991057
C -3.924227 1.245697 -1.684474
H -1.471898 2.278286 -0.655769
H -4.822283 1.489372 -2.273269
C -3.513008 1.050035 1.907915

H	-3.944444	2.063321	1.976400
C	-5.223927	0.045797	0.170502
H	-5.927486	-0.797841	0.121691
H	-1.409699	1.226516	1.623074
H	-4.702860	-0.711155	-1.761919
C	-4.626241	0.044033	1.580115
H	-4.232526	-0.960506	1.768734
H	-5.450079	0.189985	2.295824
C	-3.604633	2.437256	-0.786948
H	-3.525643	3.337394	-1.414854
H	-4.454244	2.620281	-0.112438
H	-3.174258	0.805471	2.924595
H	-5.828205	0.951167	0.008503
H	-2.207847	3.208027	0.637578
H	-3.111457	1.088020	-2.406261
B	-2.055084	-0.411009	0.307973
C	-0.461145	-0.628507	-0.700534
C	0.642834	-0.099543	0.078774
C	0.989724	1.366011	-0.010329
H	0.963969	1.752151	-1.033215
H	0.273107	1.932918	0.600678
C	0.928878	-0.648343	1.452935
H	1.936406	-0.367692	1.778845
H	0.808014	-1.731101	1.522880
O	-3.066346	-0.832876	-0.638481
C	-0.628488	-0.006551	-2.081898
H	0.273451	-0.185463	-2.692010
H	-0.811726	1.071992	-2.072990
H	0.205326	-0.191668	2.144511
C	-0.426147	-2.143910	-0.880962
H	-0.121824	-2.677336	0.022827
H	-1.412345	-2.515866	-1.185630
H	0.290971	-2.400845	-1.678801
H	1.982165	1.552687	0.413320
H	-1.478424	-0.480956	-2.586401
Se	2.669181	-1.024618	-1.097975
C	3.992109	-0.026451	-0.153561
C	4.521782	-0.498946	1.059575
C	4.450354	1.211285	-0.638243
C	5.468889	0.243157	1.765728
H	4.184357	-1.460442	1.448464
C	5.398320	1.950807	0.067809
H	4.050818	1.594856	-1.577814
C	5.913510	1.471091	1.273762
H	5.863840	-0.144743	2.705663
H	5.736738	2.909232	-0.328499
H	6.654676	2.050057	1.825206
O	-1.901919	-1.350162	1.423170
C	-2.435472	-2.631973	1.429797
H	-3.020409	-2.795070	2.352807
H	-3.100953	-2.815181	0.572160
H	-1.644440	-3.402412	1.416148

59

TS-VI-Anti_b_conf_3 Eopt -3396.635327

C	3.340219	-1.222354	1.623785
C	2.516144	0.072299	1.354686
C	4.295184	0.131647	-1.042506
C	5.009378	-1.084987	-0.411429
H	2.674844	-1.985586	2.052053
H	5.901847	-0.752464	0.138235
C	3.301004	1.300156	1.854818
H	3.455803	1.220296	2.944618
C	4.683538	1.447512	-0.332111
H	4.066280	2.266109	-0.722099
H	1.641272	0.003967	2.013000
H	4.679186	0.243031	-2.069548
C	4.670820	1.468528	1.198896
H	5.115793	2.418406	1.531959
H	5.345421	0.682728	1.570819
C	4.091910	-1.921055	0.478322
H	3.364697	-2.395758	-0.186781
H	4.683978	-2.739783	0.916051
H	2.700907	2.202463	1.680026
H	5.715442	1.654956	-0.655697
H	4.068014	-1.005459	2.424693
H	5.375208	-1.741322	-1.214031
B	1.985571	0.341441	-0.184131
C	0.415529	-0.666847	-0.551308
C	-0.675985	0.089156	0.014137
C	-0.815093	0.273939	1.499176
H	-1.843496	0.547582	1.757161
H	-0.516813	-0.596974	2.086309
C	-1.271124	1.246394	-0.731822
H	-0.602166	2.101474	-0.565233
H	-2.250665	1.509569	-0.319596
O	2.914318	-0.122166	-1.195559
C	0.598073	-2.046546	0.062786
H	1.527759	-2.504690	-0.283071
H	-0.226574	-2.702153	-0.264364
H	-1.369016	1.068815	-1.806161
C	0.376021	-0.791359	-2.072079
H	0.477531	0.171728	-2.584634
H	1.206284	-1.420462	-2.409024
H	-0.570590	-1.257460	-2.393245
H	-0.170616	1.125983	1.773315
H	0.604147	-2.042822	1.157319
Se	-2.697119	-1.574220	-0.249984
C	-4.060508	-0.280265	0.068679
C	-4.661137	0.418352	-0.994060
C	-4.471040	0.031243	1.376749
C	-5.627150	1.394835	-0.756748
H	-4.356881	0.192991	-2.016731
C	-5.435771	1.011344	1.613009
H	-4.026681	-0.504902	2.216128
C	-6.020069	1.698850	0.548570
H	-6.075348	1.923165	-1.599497
H	-5.734366	1.235079	2.638146
H	-6.774330	2.464021	0.733383
O	1.648419	1.755167	-0.358828
C	1.728548	2.374626	-1.604083
H	0.759136	2.396678	-2.131585
H	2.045950	3.421681	-1.470084
H	2.449374	1.878570	-2.271428

59

TS-VI-Anti_b_conf_4 Eopt -3396.637174

C	-2.345655	2.189720	-0.467657
C	-2.232230	1.193897	0.698303
C	-4.257762	-0.333075	-0.828599
C	-4.230208	0.900363	-1.748335
H	-1.544608	1.986405	-1.188252
H	-5.255985	1.072405	-2.111987
C	-3.338611	1.365206	1.761045
H	-3.319554	2.399148	2.148861
C	-5.030020	-0.188453	0.495795
H	-4.817802	-1.099615	1.074455
H	-1.317836	1.474429	1.244522
H	-4.820353	-1.091314	-1.400167
C	-4.792713	1.046478	1.371058
H	-5.366673	0.903662	2.299626
H	-5.256301	1.913471	0.887513
C	-3.676903	2.234452	-1.228023
H	-3.545042	2.893505	-2.099736
H	-4.429457	2.733921	-0.608752
H	-3.062863	0.714953	2.602157
H	-6.103721	-0.220653	0.254499
H	-2.153972	3.211792	-0.096009
H	-3.641456	0.590793	-2.625693
B	-2.034021	-0.380854	0.293118
C	-0.415901	-0.785621	-0.615317
C	0.661603	-0.128134	0.095143
C	1.037762	1.286098	-0.271342
H	0.262312	1.968645	0.103081
H	1.977726	1.573589	0.211294
C	0.883322	-0.413402	1.557748
H	0.139647	0.167954	2.123965
H	1.881147	-0.085042	1.868124
O	-2.980903	-0.893211	-0.667193
C	-0.535600	-0.413077	-2.089486

H -0.695341 0.653338 -2.273901
H -1.382481 -0.952100 -2.528008
H 0.743248 -1.464142 1.818829
C -0.391818 -2.308616 -0.524176
H -1.357184 -2.724950 -0.836767
H 0.379406 -2.701533 -1.208106
H -0.167565 -2.677259 0.480322
H 1.135404 1.431376 -1.351017
H 0.379109 -0.709364 -2.631340
Se 2.736222 -1.284012 -0.795274
C 4.009543 -0.039512 -0.110089
C 4.457450 1.042020 -0.889104
C 4.504164 -0.160216 1.199916
C 5.357346 1.973088 -0.372824
H 4.087230 1.152047 -1.908951
C 5.402644 0.774181 1.716067
H 4.178772 -0.998156 1.817287
C 5.834364 1.845884 0.933545
H 5.687780 2.804743 -0.996787
H 5.769854 0.659398 2.736849
H 6.537113 2.575166 1.337126
O -1.981662 -1.150250 1.535894
C -2.554508 -2.405515 1.689042
H -3.058597 -2.754294 0.775245
H -1.797038 -3.159162 1.967561
H -3.303460 -2.389347 2.502014
59
TS-VI-Anti_b_conf_5 Eopt -
3396.635412
C 3.667454 0.108533 -1.879582
C 3.366304 -0.684753 -0.591395
C 2.805821 2.026152 0.544991
C 3.052038 2.409647 -0.924579
H 2.821063 0.071364 -2.569876
H 3.392439 3.456752 -0.931276
C 4.606260 -0.605287 0.349010
H 5.492875 -0.325228 -0.243823
C 4.118597 1.790859 1.332483
H 4.012650 2.253484 2.324023
H 3.295044 -1.733110 -0.922705
H 2.331230 2.915678 0.990858
C 4.503624 0.325741 1.567586
H 3.762562 -0.098565 2.255239
H 5.470696 0.312651 2.093617
C 4.066729 1.568877 -1.690279
H 4.226110 2.021352 -2.680604
H 5.039054 1.626082 -1.179314
H 4.829505 -1.606878 0.742084
H 4.937818 2.332125 0.835321
H 4.499454 -0.396106 -2.400710
H 2.078774 2.393055 -1.439640
B 2.005758 -0.376025 0.292503
C 0.437456 -0.850459 -0.646986
C -0.622854 -0.217335 0.120817
C -0.820522 -0.539596 1.576392
H -0.665174 -1.594311 1.813250
H -0.069748 0.044632 2.129163
C -0.971418 1.210175 -0.200772
H -1.218853 1.360217 -1.255991
H -0.076619 1.801596 0.043111
O 1.863088 1.004523 0.714471
C 0.404456 -2.373514 -0.625519
H 0.231779 -2.782413 0.374217
H 1.342977 -2.794856 -1.008975
H -1.805044 1.565223 0.414590
C 0.464700 -0.380653 -2.097330
H -0.536983 -0.486748 -2.547431
H 0.772143 0.666329 -2.206311
H 1.150888 -0.999145 -2.686973
H -1.817649 -0.227457 1.904522
H -0.404873 -2.733123 -1.283188
Se -2.737382 -1.347191 -0.747279
C -3.934017 -0.013192 -0.094758
C -4.434956 -0.069954 1.217056
C -4.311521 1.075469 -0.900301
C -5.270525 0.933120 1.709751
H -4.164823 -0.912457 1.854531
C -5.148564 2.075334 -0.407495
H -3.937002 1.136026 -1.922707
C -5.631372 2.011842 0.901505
H -5.643762 0.867244 2.732708
H -5.424612 2.911355 -1.051733
H -6.284191 2.795365 1.286898
O 1.940666 -1.208876 1.498069
C 2.490817 -2.467792 1.667739
H 1.748250 -3.154839 2.110042
H 2.846752 -2.931252 0.734866
H 3.348403 -2.433893 2.366863
59
TS-VI-Anti_b_conf_6 Eopt -
3396.636970
C 3.737182 -1.459692 1.183861
C 3.120134 -0.061034 1.334236
C 3.538514 0.149828 -1.615107
C 3.980114 -1.307231 -1.388014
H 2.957040 -2.230039 1.190923
H 4.713875 -1.529782 -2.178499
C 4.247995 1.010267 1.375925
H 5.199603 0.527357 1.657105
C 4.646140 1.159974 -1.229495
H 4.680353 1.946369 -1.997425
H 2.659173 -0.042176 2.332850
H 3.406925 0.233084 -2.705786
C 4.469728 1.863866 0.118669
H 3.619520 2.545902 0.023288
H 5.352742 2.500844 0.283233
C 4.624765 -1.646973 -0.045485
H 4.973837 -2.689980 -0.079991
H 5.533108 -1.037174 0.073535
H 4.027857 1.719580 2.185952
H 5.622005 0.653453 -1.276004
H 4.358964 -1.666250 2.072519
H 3.117933 -1.958524 -1.577631
B 1.925211 0.433672 0.315578
C 0.444123 -0.731469 0.449993
C -0.698642 0.047523 -0.012668
C -1.350975 0.982151 0.974589
H -0.567814 1.637451 1.375125
H -2.133692 1.586724 0.504202
C -0.731218 0.519667 -1.445212
H -1.703246 0.966142 -1.682419
H -0.523650 -0.281800 -2.158151
O 2.278655 0.480055 -1.086077
C 0.314128 -1.147114 1.912679
H 0.341284 -0.284944 2.591300
H 1.130658 -1.819095 2.199964
H 0.047968 1.281762 -1.576304
C 0.718033 -1.941661 -0.434704
H 0.894684 -1.659230 -1.476940
H 1.590869 -2.504127 -0.095050
H -0.140622 -2.633956 -0.404146
H -1.788794 0.437764 1.817951
H -0.635250 -1.683196 2.082631
Se -2.580856 -1.612883 -0.222716
C -3.981044 -0.319715 -0.129411
C -4.594173 -0.008421 1.096033
C -4.397616 0.378709 -1.275347
C -5.575858 0.978525 1.175608
H -4.288350 -0.543892 1.995403
C -5.376938 1.368890 -1.193401
H -3.944774 0.141471 -2.238601
C -5.970001 1.676228 0.032150
H -6.034398 1.204742 2.139280
H -5.680229 1.900954 -2.096179
H -6.734901 2.450529 0.095399
O 1.511381 1.750878 0.837001
C 1.223361 2.844385 0.030088
H 1.668250 2.753423 -0.973862
H 0.136400 2.996924 -0.103737
H 1.618916 3.761675 0.497197

59
TS-VI-Anti_b_conf_7 Eopt -
3396.640431
C 3.392083 0.271972 -2.011743
C 3.276667 -0.722138 -0.825709
C 3.006542 1.827151 0.721707
C 3.221436 2.504742 -0.652988
H 2.939301 -0.172371 -2.908353
H 4.276745 2.793674 -0.766530
C 4.640860 -0.863644 -0.122052
H 5.370602 -1.302597 -0.824327
C 4.304496 1.249329 1.320502
H 4.036643 0.643537 2.197886
H 3.077122 -1.717469 -1.253555
H 2.697560 2.616840 1.425343
C 5.223398 0.448201 0.398181
H 6.155930 0.235363 0.942544
H 5.517415 1.081723 -0.452097
C 2.758996 1.658220 -1.839689
H 1.673239 1.538417 -1.744854
H 2.926367 2.235691 -2.761966
H 4.535273 -1.569281 0.712751
H 4.875493 2.110984 1.699400
H 4.456138 0.406639 -2.269730
H 2.646816 3.441961 -0.674889
B 2.082607 -0.447384 0.281422
C 0.461102 -1.083659 -0.496241
C -0.571992 -0.245854 0.077086
C -0.796202 -0.226281 1.563547
H -1.785175 0.180638 1.798725
H -0.690688 -1.207807 2.030004
C -0.854825 1.091652 -0.550295
H -1.740959 1.555284 -0.104029
H -0.987913 1.039622 -1.634590
O 1.919122 0.935992 0.681501
C 0.410885 -2.547824 -0.074033
H 1.348489 -3.054511 -0.337170
H -0.409905 -3.057013 -0.606961
H 0.014392 1.726786 -0.321167
C 0.499853 -1.042757 -2.022176
H -0.469518 -1.383166 -2.423042
H 0.698551 -0.052051 -2.438681
H 1.268698 -1.725561 -2.402623
H -0.042422 0.455310 1.984812
H 0.264082 -2.684011 1.000850
Se -2.732657 -1.443136 -0.555726
C -3.911321 -0.019646 -0.082934
C -4.296752 0.946612 -1.029188
C -4.397339 0.110943 1.229513
C -5.128641 2.007098 -0.673197
H -3.932043 0.862483 -2.053499
C -5.227831 1.174291 1.584831
H -4.118701 -0.633076 1.976708
C -5.599021 2.128144 0.636210
H -5.411250 2.744545 -1.425873
H -5.589812 1.253745 2.610852
H -6.249208 2.958072 0.913778
O 2.317843 -1.333646 1.417091
C 2.103826 -1.009271 2.747548
H 1.243041 -1.558320 3.169876
H 2.988126 -1.289090 3.346985
H 1.921830 0.066116 2.898984
59
TS-VI-Anti_b_conf_8 Eopt -
3396.635449
C -3.768118 -1.121401 -1.363555
C -3.190849 0.290224 -1.157406
C -3.436852 -0.355818 1.753034
C -3.893817 -1.702055 1.164552
H -2.975452 -1.829506 -1.630042
H -4.599916 -2.135535 1.890305
C -4.351833 1.274530 -0.822739
H -5.313860 0.809379 -1.094150
C -4.554414 0.717975 1.724806
H -4.545159 1.250986 2.686251
H -2.813253 0.586885 -2.146845
H -3.244921 -0.573307 2.816089
C -4.436181 1.776047 0.624360
H -3.541585 2.375088 0.835052
H -5.301264 2.451617 0.710207
C -4.585160 -1.677598 -0.196814
H -4.904210 -2.701135 -0.444885
H -5.514892 -1.096501 -0.104347
H -4.273646 2.161079 -1.466583
H -5.532614 0.215625 1.667542
H -4.437312 -1.093379 -2.241111
H -3.023677 -2.370555 1.145846
B -1.943442 0.509378 -0.101298
C -0.444781 -0.538790 -0.629527
C 0.682783 0.120950 0.003805
C 1.349850 1.273875 -0.691757
H 1.595051 1.059561 -1.736141
H 0.649820 2.117014 -0.654343
C 0.735781 0.251905 1.500943
H 1.748001 0.508538 1.831767
H 0.391342 -0.638916 2.029322
O -2.207759 0.107931 1.262600
C -0.372977 -0.537152 -2.153480
H -1.205090 -1.110882 -2.577935
H 0.564068 -1.004320 -2.502391
H 0.059975 1.081575 1.759993
C -0.668168 -1.953706 -0.111497
H 0.145863 -2.608437 -0.466064
H -0.693627 -1.997941 0.981158
H -1.603257 -2.380851 -0.478606
H 2.262567 1.573655 -0.166371
H -0.421033 0.474449 -2.576581
Se 2.593327 -1.573183 -0.242237
C 4.011749 -0.341586 0.088276
C 4.417693 -0.042863 1.400214
C 4.656066 0.322984 -0.970034
C 5.420810 0.895740 1.644743
H 3.939021 -0.554540 2.235860
C 5.660198 1.258219 -0.724325
H 4.357054 0.104215 -1.995642
C 6.047773 1.552103 0.584817
H 5.715568 1.111656 2.672665
H 6.142555 1.761970 -1.563236
H 6.831802 2.285028 0.776377
O -1.497518 1.909931 -0.077218
C -1.649234 2.815096 -1.117460
H -0.814647 3.535653 -1.108082
H -1.663076 2.348951 -2.116093
H -2.575790 3.409271 -1.015248
59
TS-VI-Anti_b_conf_9 Eopt -
3396.639721
C 3.724507 -1.313325 1.054982
C 2.980078 0.018435 1.338134
C 3.404878 0.384611 -1.602872
C 4.157263 -0.964256 -1.505244
H 3.333782 -2.100033 1.714561
H 5.238965 -0.783481 -1.420017
C 3.997485 1.164338 1.506351
H 4.630683 0.964436 2.388121
C 4.210702 1.569531 -1.033433
H 3.543844 2.441882 -0.979530
H 2.502539 -0.079321 2.325942
H 3.288211 0.611261 -2.674846
C 4.919000 1.373815 0.307043
H 5.554053 2.253395 0.492330
H 5.612094 0.523194 0.224266
C 3.667945 -1.868820 -0.373235
H 2.630593 -2.140182 -0.601916
H 4.245445 -2.805586 -0.406450
H 3.450250 2.094550 1.708721
H 4.976539 1.808280 -1.787444
H 4.783946 -1.203795 1.342455
H 4.015829 -1.512747 -2.447690
B 1.783514 0.459329 0.288284

C 0.245050 -0.568821 0.754570
C -0.486883 -0.732279 -0.485688
C -0.987712 0.473150 -1.229304
H -1.408732 1.244990 -0.579658
H -0.119477 0.888414 -1.760518
C -0.076066 -1.846898 -1.415797
H -0.037335 -2.824016 -0.924871
H 0.928276 -1.590023 -1.784272
O 2.090951 0.267143 -1.114944
C -0.437280 0.291831 1.812230
H -0.881638 1.205886 1.406574
H 0.287876 0.599790 2.576345
H -0.756221 -1.910613 -2.270884
C 0.616582 -1.895853 1.412029
H -0.304091 -2.464190 1.631174
H 1.262932 -2.533134 0.802846
H 1.124446 -1.720382 2.367837
H -1.737586 0.186452 -1.973375
H -1.226360 -0.297610 2.304819
Se -2.743695 -1.726778 0.111556
C -3.796152 -0.132871 0.031109
C -3.914562 0.720953 1.140291
C -4.442771 0.238197 -1.159816
C -4.637146 1.910641 1.055514
H -3.427564 0.450439 2.076692
C -5.167748 1.427131 -1.242822
H -4.368562 -0.412933 -2.031368
C -5.266730 2.272534 -0.136918
H -4.707915 2.558552 1.930171
H -5.656496 1.694034 -2.180950
H -5.829388 3.203988 -0.203060
O 1.416496 1.837881 0.596891
C 1.045602 2.801588 -0.327259
H 1.582899 3.743206 -0.117845
H 1.274760 2.508012 -1.363803
H -0.034366 3.029103 -0.273444
59
TS-VI-Gauche_b_conf_1 Eopt -3396.604602
C 3.274239 1.027829 -1.746343
C 2.293784 -0.097597 -1.360769
C 3.993500 -0.154058 1.075699
C 4.880542 0.825052 0.292344
H 2.875383 1.991290 -1.406964
H 5.931857 0.584313 0.517189
C 2.838429 -1.512243 -1.650769
H 3.117960 -1.589471 -2.716019
C 4.109066 -1.633812 0.663353
H 3.345103 -2.181316 1.229746
H 1.425394 -0.000049 -2.030708
H 4.373459 -0.119968 2.110762
C 4.033833 -2.023635 -0.820496
H 4.024553 -3.123413 -0.860946
H 4.975943 -1.737161 -1.300814
C 4.713039 0.924711 -1.229208
H 5.251785 1.826084 -1.559180
H 5.223989 0.092288 -1.723896
H 1.998398 -2.202598 -1.502390
H 5.079303 -1.990708 1.041814
H 3.329393 1.098061 -2.846156
H 4.688701 1.814951 0.735165
B 1.710822 -0.025927 0.158910
C 0.446463 1.590924 0.153897
C -0.728604 1.331312 0.924766
C -0.572162 1.005563 2.380319
H -1.381499 0.354316 2.726665
H 0.398284 0.554102 2.602037
C -2.037265 1.986238 0.600682
H -2.860235 1.592016 1.206010
H -2.311014 1.939990 -0.455074
O 2.666503 0.315177 1.171185
C 0.117739 2.097200 -1.245897
H 1.015207 2.253442 -1.849072
H -0.412705 3.064153 -1.208436
H -1.901418 3.053245 0.866032
C 1.328719 2.616504 0.900710
H 2.270828 2.772706 0.364802
H 0.800889 3.586092 0.951588
H 1.587931 2.302208 1.912937
H -0.652499 1.951585 2.943946
H -0.526372 1.382837 -1.777349
Se -1.792639 -1.025950 -0.163281
C -3.650241 -0.576156 -0.303241
C -4.544154 -0.830650 0.754106
C -4.168171 0.048454 -1.453227
C -5.889129 -0.472003 0.669037
H -4.171088 -1.316712 1.656979
C -5.513325 0.407141 -1.540364
H -3.498428 0.255339 -2.289651
C -6.384217 0.148928 -0.479833
H -6.556338 -0.681582 1.506770
H -5.883947 0.890378 -2.445788
H -7.436107 0.427892 -0.547777
O 0.953851 -1.206759 0.455759
C 0.901664 -1.821428 1.704975
H 1.587215 -1.356787 2.429384
H -0.118766 -1.788067 2.115208
H 1.179130 -2.884493 1.606168
59
TS-VI-Gauche_b_conf_10 Eopt -3396.612395
C -4.046844 1.225969 -0.695234
C -3.336253 0.753911 0.579983
C -2.323492 -1.505082 -1.092048
C -3.008616 -0.642372 -2.165405
H -3.524121 2.084024 -1.133622
H -3.302827 -1.336350 -2.968002
C -4.164884 -0.384798 1.248509
H -5.204207 -0.336204 0.881391
C -3.328731 -2.360638 -0.285121
H -2.903246 -3.365718 -0.154755
H -3.384547 1.596500 1.283546
H -1.683170 -2.201885 -1.654248
C -3.654584 -1.826993 1.112358
H -2.748363 -1.925479 1.718388
H -4.397055 -2.499650 1.568361
C -4.253354 0.144931 -1.755606
H -4.688727 0.603165 -2.656284
H -5.011724 -0.564982 -1.392207
H -4.224172 -0.185936 2.328010
H -4.247929 -2.494856 -0.874426
H -5.044300 1.608470 -0.416851
H -2.250957 0.022011 -2.598180
B -1.756342 0.298684 0.563326
C -0.605336 1.790377 -0.115416
C 0.777465 1.623325 0.228571
C 1.853197 2.052316 -0.732298
H 1.738197 1.653523 -1.742096
H 1.775578 3.155046 -0.793440
C 1.205448 1.785038 1.657585
H 0.443147 1.438410 2.356912
H 1.367375 2.864543 1.828630
O -1.430499 -0.816269 -0.251622
C -1.222622 2.944675 0.697068
H -2.220486 3.175688 0.308323
H -0.616858 3.863516 0.605902
H 2.162195 1.283304 1.840301
C -0.794670 1.981431 -1.612729
H -0.374242 1.131952 -2.167474
H -1.846547 2.068035 -1.887240
H -0.295352 2.898183 -1.972542
H 2.853043 1.811898 -0.358422
H -1.320742 2.700070 1.760368
Se 1.459984 -0.990420 -0.246869
C 3.354513 -0.716664 -0.209590
C 4.099436 -0.556056 -1.393274
C 4.048512 -0.616478 1.011318
C 5.467855 -0.291265 -1.358360
H 3.588598 -0.633307 -2.354082
C 5.416447 -0.345243 1.047995

H 3.500213 -0.752835 1.944745
C 6.135907 -0.178998 -0.136768
H 6.016590 -0.169040 -2.293563
H 5.923945 -0.267557 2.010764
H 7.205230 0.032395 -0.108947
O -1.348318 0.179445 1.951041
C -0.687912 -0.924103 2.488042
H 0.338950 -0.670493 2.799332
H -1.229317 -1.277162 3.383044
H -0.610273 -1.753529 1.772437
59
TS-VI-Gauche_b_conf_2 Eopt -
3396.612994
C -2.627547 -0.797181 2.101198
C -1.671912 -0.924040 0.878839
C -4.136091 0.007212 -0.535083
C -4.824453 -0.098618 0.844597
H -2.054582 -0.451118 2.972299
H -5.343204 -1.064396 0.931394
C -1.894184 -2.280567 0.184372
H -1.644671 -3.096711 0.883551
C -3.905026 -1.378648 -1.175809
H -3.286589 -1.254153 -2.073428
H -0.647279 -0.963638 1.274569
H -4.836056 0.519299 -1.213841
C -3.326687 -2.495791 -0.302784
H -3.374316 -3.434049 -0.875562
H -3.986689 -2.648209 0.564394
C -3.861778 0.114028 2.010897
H -3.536163 1.158781 1.957723
H -4.426562 0.015534 2.950911
H -1.199637 -2.362923 -0.661831
H -4.895990 -1.709076 -1.523872
H -2.973739 -1.807399 2.378351
H -5.604561 0.672933 0.914825
B -1.708395 0.267057 -0.247573
C -0.598573 1.769179 0.464866
C 0.778215 1.724672 0.047442
C 1.159602 2.352741 -1.263826
H 2.186557 2.101181 -1.544329
H 0.484816 2.070004 -2.074633
C 1.867841 1.809241 1.077705
H 1.801495 2.819563 1.522806
H 2.861450 1.700363 0.634610
O -3.007494 0.855143 -0.470180
C -0.758681 1.775426 1.982102
H -1.813639 1.819151 2.259913
H -0.275988 2.662419 2.427575
H 1.756054 1.087072 1.891549
C -1.342350 2.980887 -0.124784
H -1.384350 2.986632 -1.217564
H -2.374680 2.983154 0.236895
H -0.858265 3.919382 0.199192
H 1.096974 3.448952 -1.138853
H -0.321677 0.889466 2.456267
Se 1.575876 -0.739127 -0.750599
C 3.384039 -0.711479 -0.116616
C 3.701076 -1.121473 1.191945
C 4.435137 -0.232396 -0.920392
C 5.005174 -1.043550 1.679293
H 2.905017 -1.500825 1.834406
C 5.740571 -0.154741 -0.434471
H 4.219028 0.086439 -1.941216
C 6.034523 -0.558101 0.869511
H 5.218925 -1.365155 2.699826
H 6.534353 0.223908 -1.080383
H 7.054444 -0.496383 1.250162
O -1.083813 -0.143019 -1.475022
C -1.368431 0.464678 -2.694462
H -0.471983 0.423511 -3.332624
H -2.176868 -0.058356 -3.235515
H -1.681764 1.513813 -2.594965
59
TS-VI-Gauche_b_conf_3 Eopt -
3396.612069
C 2.920505 -0.729847 2.010521
C 3.219492 0.341977 0.929918
C 1.966704 -1.740725 -0.839800
C 1.878750 -2.562909 0.467064
H 2.690682 -0.236943 2.964395
H 2.733142 -3.252938 0.533173
C 4.526193 -0.002988 0.183926
H 5.369917 0.037135 0.894065
C 3.384695 -1.669975 -1.436343
H 3.367901 -0.923216 -2.245373
H 3.432693 1.291005 1.446567
H 1.356087 -2.262088 -1.593126
C 4.541736 -1.380414 -0.478823
H 5.484846 -1.494602 -1.034374
H 4.562088 -2.159432 0.298022
C 1.771655 -1.704148 1.726834
H 0.836298 -1.136495 1.644383
H 1.657858 -2.373921 2.593137
H 4.711876 0.766583 -0.577002
H 3.574579 -2.644515 -1.911340
H 3.835456 -1.314215 2.206277
H 0.976780 -3.189215 0.421000
B 2.040451 0.641864 -0.166360
C 0.637361 1.779742 0.816747
C -0.718512 1.664706 0.347186
C -1.844121 1.664976 1.344600
H -1.726627 0.922687 2.138666
H -1.830176 2.663999 1.820230
C -1.098646 2.322277 -0.950754
H -0.348663 2.170421 -1.730274
H -1.189269 3.407670 -0.765063
O 1.355007 -0.482687 -0.692244
C 1.230677 3.156343 0.463632
H 1.195198 3.387637 -0.602819
H 2.284125 3.196247 0.766692
H -2.069703 1.963971 -1.305728
C 0.766762 1.584738 2.325091
H 0.174638 2.338770 2.871803
H 0.426643 0.598914 2.663018
H 1.805333 1.712997 2.644473
H -2.819272 1.535746 0.867545
H 0.689807 3.948199 1.013159
Se -1.357826 -0.833964 -0.458634
C -3.258575 -0.703620 -0.247210
C -3.895047 -1.123419 0.936069
C -4.065034 -0.139984 -1.253562
C -5.269945 -0.973581 1.111200
H -3.294129 -1.568608 1.730267
C -5.440694 0.013536 -1.078621
H -3.599535 0.183130 -2.185920
C -6.052628 -0.400881 0.105671
H -5.734299 -1.306413 2.040837
H -6.038986 0.457291 -1.875968
H -7.127799 -0.282079 0.242892
O 2.559446 1.520463 -1.184410
C 2.049961 1.552708 -2.478956
H 2.862130 1.391903 -3.207977
H 1.282370 0.782740 -2.648281
H 1.608462 2.539832 -2.705125
59
TS-VI-Gauche_b_conf_4 Eopt -
3396.608477
C -4.122724 0.795525 -0.894359
C -3.276880 0.738927 0.400289
C -2.248670 -1.709560 -0.966962
C -3.399675 -1.469097 -1.953468
H -3.612285 1.377499 -1.665872
H -3.822774 -2.446791 -2.235341
C -3.923561 -0.233376 1.418465
H -4.994807 0.016836 1.512602
C -2.646676 -2.343875 0.375728
H -1.742142 -2.343501 0.995059
H -3.372385 1.736056 0.862316
H -1.597979 -2.457060 -1.452421
C -3.832463 -1.761307 1.166102

H -3.831226 -2.259485 2.147114
H -4.755533 -2.104430 0.684250
C -4.539522 -0.537479 -1.525942
H -5.131983 -0.306728 -2.424527
H -5.230627 -1.061232 -0.856556
H -3.493013 -0.029120 2.403983
H -2.891245 -3.396888 0.168283
H -5.054542 1.343680 -0.672208
H -2.930852 -1.059919 -2.862379
B -1.678454 0.357596 0.235896
C -0.627148 1.915572 -0.308049
C 0.715659 1.538782 -0.635626
C 0.988395 0.918575 -1.971583
H 1.986187 0.470326 -2.008110
H 0.224892 0.175498 -2.218416
C 1.871477 2.308952 -0.069761
H 1.833847 3.304714 -0.553727
H 2.833481 1.851812 -0.318237
O -1.447297 -0.559048 -0.837836
C -0.737729 2.984618 0.774340
H -0.343438 3.949966 0.412135
H -0.187157 2.728136 1.684074
H 1.809798 2.467899 1.009035
C -1.378893 2.384637 -1.565590
H -2.332162 2.834264 -1.269887
H -0.802457 3.167143 -2.087611
H -1.577480 1.572523 -2.271271
H 0.954317 1.723833 -2.727648
H -1.785715 3.150166 1.051159
Se 1.593265 -0.714068 0.799147
C 3.416669 -0.677787 0.209015
C 4.416633 -0.007049 0.939935
C 3.802733 -1.288050 -1.000460
C 5.732585 0.056626 0.483031
H 4.149081 0.475019 1.881606
C 5.118184 -1.224042 -1.460246
H 3.050622 -1.819968 -1.585432
C 6.093672 -0.551555 -0.721410
H 6.482499 0.585614 1.073545
H 5.382939 -1.706665 -2.402424
H 7.122495 -0.502144 -1.079240
O -1.088589 -0.108995 1.463298
C -1.149289 0.538462 2.689533
H -0.158025 0.933579 2.969911
H -1.864768 1.375894 2.709182
H -1.445873 -0.176850 3.474970
59
TS-VI-Gauche_b_conf_5 Eopt -3396.610063
C 4.252139 0.894862 0.521316
C 3.270400 0.624262 -0.657146
C 2.401089 -1.585130 1.185691
C 3.565305 -1.121068 2.091011
H 4.389506 1.979744 0.638792
H 4.439219 -1.769759 1.932364
C 3.869820 -0.502962 -1.531442
H 4.843501 -0.166770 -1.927152
C 2.895294 -2.386222 -0.034231
H 2.045139 -2.531938 -0.713159
H 3.299588 1.524241 -1.289740
H 1.795129 -2.295912 1.769152
C 4.098007 -1.825759 -0.798558
H 4.423912 -2.580167 -1.530388
H 4.941560 -1.711835 -0.101329
C 3.937684 0.354081 1.925766
H 3.115551 0.937829 2.346454
H 4.811082 0.559028 2.564548
H 3.234369 -0.691334 -2.403449
H 3.178638 -3.378187 0.349740
H 5.248323 0.522253 0.228393
H 3.273631 -1.262225 3.141859
B 1.688955 0.242376 -0.315885
C 0.656289 1.855595 0.021765
C -0.692293 1.548546 0.409245
C -0.968633 1.140502 1.825359
H -1.977513 0.731221 1.931985
H -0.225011 0.418888 2.176395
C -1.838869 2.247164 -0.259570
H -2.806550 1.854685 0.065063
H -1.785827 2.232560 -1.351014
O 1.530277 -0.517035 0.891210
C 0.767128 2.788299 -1.182393
H 0.375518 3.789466 -0.932698
H 0.216632 2.434774 -2.058235
H -1.775888 3.305542 0.060296
C 1.425306 2.477992 1.197440
H 2.453479 2.704407 0.897880
H 0.951474 3.430293 1.492871
H 1.450302 1.827448 2.071723
H -0.904170 2.046563 2.454763
H 1.814486 2.920920 -1.476797
Se -1.616922 -0.892500 -0.607294
C -3.451725 -0.692992 -0.089506
C -3.892761 -1.039973 1.201766
C -4.406360 -0.152100 -0.973056
C -5.217325 -0.845523 1.594615
H -3.178479 -1.469945 1.905806
C -5.730748 0.041856 -0.583219
H -4.094411 0.124305 -1.981549
C -6.146467 -0.302790 0.705071
H -5.525657 -1.124193 2.603553
H -6.444259 0.465613 -1.291899
H -7.182008 -0.151253 1.011079
O 1.044767 -0.427914 -1.416675
C 0.983645 0.058933 -2.715012
H 1.144526 -0.767428 -3.426513
H -0.012278 0.481230 -2.930512
H 1.735166 0.837209 -2.926618
59
TS-VI-Gauche_b_conf_6 Eopt -3396.616022
C -2.386163 -0.769029 2.070829
C -1.646858 -0.940545 0.732497
C -4.154597 0.216079 -0.356211
C -4.654993 0.074293 1.089501
H -2.111731 0.191402 2.521060
H -5.714439 -0.225926 1.051782
C -2.035744 -2.238321 -0.005754
H -1.887078 -3.102616 0.664962
C -4.100137 -1.080627 -1.186358
H -3.606338 -0.821970 -2.132944
H -0.586914 -1.086455 0.984083
H -4.911741 0.841038 -0.859319
C -3.459524 -2.343976 -0.591441
H -3.440059 -3.099048 -1.391988
H -4.144570 -2.753937 0.159428
C -3.916288 -0.865140 2.051553
H -4.281499 -0.646846 3.066747
H -4.210178 -1.902946 1.861912
H -1.314348 -2.356937 -0.824140
H -5.138990 -1.337104 -1.444973
H -2.027514 -1.539903 2.774548
H -4.639132 1.093753 1.505721
B -1.699857 0.312984 -0.307855
C -0.586606 1.849189 0.309073
C 0.761013 1.516657 0.653503
C 1.901893 2.334523 0.116626
H 1.830225 3.317231 0.622124
H 2.875183 1.901368 0.367411
C 1.059377 0.918933 1.998941
H 1.165389 1.748065 2.720213
H 2.011532 0.378038 1.987191
O -2.968626 0.975595 -0.422045
C -1.367513 2.410797 1.510572
H -1.205523 1.868115 2.443666
H -2.441019 2.419892 1.291653
H 0.264440 0.258891 2.357289
C -0.712295 2.820397 -0.858562
H -1.768155 2.956560 -1.119418
H -0.302563 3.811586 -0.598556

H -0.181971 2.464693 -1.748960
H 1.847702 2.512552 -0.959052
H -1.045293 3.452451 1.686802
Se 1.607911 -0.604525 -0.890136
C 3.408727 -0.644262 -0.239201
C 4.443596 0.045519 -0.899527
C 3.743985 -1.343690 0.935721
C 5.747888 0.041527 -0.406578
H 4.213115 0.595588 -1.813153
C 5.047853 -1.346281 1.431921
H 2.964244 -1.894777 1.463878
C 6.059627 -0.654222 0.763811
H 6.527239 0.586328 -0.941842
H 5.274577 -1.897678 2.345744
H 7.079426 -0.657640 1.149707
O -1.123165 -0.074694 -1.563599
C -1.464502 0.474460 -2.794636
H -2.261670 1.228245 -2.713425
H -0.586900 0.942209 -3.270465
H -1.813245 -0.320182 -3.476325
59
TS-VI-Gauche_b_conf_7 Eopt -
3396.611552
C -4.067209 1.173285 -0.572043
C -3.282557 0.706543 0.663109
C -2.299933 -1.519221 -1.029716
C -2.956194 -0.673771 -2.131804
H -3.668582 2.146287 -0.885166
H -3.297212 -1.374097 -2.909983
C -4.010259 -0.436741 1.409459
H -4.948121 -0.042159 1.836740
C -3.246891 -2.370562 -0.169940
H -2.601698 -2.900004 0.548163
H -3.315173 1.548202 1.371027
H -1.675803 -2.247615 -1.573643
C -4.383079 -1.679647 0.593722
H -4.814292 -2.421218 1.283457
H -5.195646 -1.427518 -0.097418
C -4.146551 0.241412 -1.802076
H -4.326880 0.865940 -2.690416
H -5.041694 -0.385249 -1.719767
H -3.392432 -0.740422 2.266645
H -3.684935 -3.144702 -0.819364
H -5.106723 1.385624 -0.264477
H -2.144391 -0.094393 -2.586719
B -1.691701 0.303108 0.579897
C -0.619859 1.816567 -0.110788
C 0.778424 1.657757 0.157265
C 1.796065 2.047482 -0.877696
H 1.606637 1.633431 -1.869950
H 1.734197 3.150322 -0.957060
C 1.287945 1.824361 1.557388
H 0.595085 1.406751 2.290888
H 1.383414 2.909112 1.746055
O -1.381077 -0.805023 -0.242343
C -1.212309 2.943841 0.756951
H -1.248016 2.676896 1.818582
H -2.232401 3.166736 0.426102
H 2.285429 1.385591 1.667983
C -0.910297 2.022242 -1.590034
H -1.982732 2.060882 -1.792967
H -0.475914 2.967411 -1.960737
H -0.489664 1.204797 -2.188718
H 2.813602 1.794169 -0.565557
H -0.625548 3.872747 0.650834
Se 1.486732 -0.996594 -0.218705
C 3.381455 -0.721592 -0.185322
C 4.127582 -0.580644 -1.371211
C 4.077247 -0.607989 1.033626
C 5.497964 -0.325690 -1.340480
H 3.616007 -0.667987 -2.330876
C 5.447490 -0.348192 1.066164
H 3.527763 -0.726588 1.968847
C 6.168101 -0.204185 -0.120879
H 6.046857 -0.219754 -2.277638
H 5.956057 -0.262360 2.027689
H 7.239375 -0.002507 -0.096400
O -1.194664 0.195163 1.939698
C -0.772902 -1.005496 2.502041
H 0.264844 -0.925288 2.867761
H -1.411102 -1.268952 3.365052
H -0.800159 -1.835122 1.782261
59
TS-VI-Gauche_b_conf_8 Eopt -
3396.617561
C -2.473001 -0.656505 2.147777
C -1.615112 -0.862375 0.870340
C -4.156617 -0.002380 -0.452853
C -4.759044 -0.037235 0.969899
H -1.843791 -0.213399 2.935790
H -5.292663 -0.987065 1.118967
C -1.893476 -2.255205 0.270503
H -1.612874 -3.029500 1.004423
C -3.969736 -1.419811 -1.034004
H -3.401161 -1.344336 -1.970576
H -0.567853 -0.910399 1.193988
H -4.897889 0.479551 -1.109494
C -3.350775 -2.486121 -0.125684
H -3.435023 -3.459375 -0.632241
H -3.962660 -2.575030 0.784901
C -3.745233 0.206021 2.089575
H -3.451121 1.256224 2.023809
H -4.269101 0.098488 3.052204
H -1.246615 -2.396803 -0.604528
H -4.978944 -1.762999 -1.309136
H -2.747937 -1.650322 2.540553
H -5.521367 0.751064 1.050334
B -1.712742 0.254148 -0.324496
C -0.638410 1.863058 0.087139
C 0.732000 1.606378 0.398515
C 1.820209 2.357141 -0.314782
H 2.815754 1.984335 -0.055029
H 1.707854 2.366416 -1.400823
C 1.110243 1.223391 1.799985
H 2.105321 0.768975 1.835194
H 0.383604 0.555896 2.271317
O -3.019877 0.835522 -0.514532
C -1.389195 2.462910 1.283936
H -2.422805 2.665858 0.988130
H -0.925645 3.421138 1.577775
H 1.148467 2.151559 2.397929
C -0.856080 2.742037 -1.139049
H -1.926301 2.808682 -1.364732
H -0.485686 3.766902 -0.962369
H -0.345293 2.355039 -2.026672
H 1.747922 3.404449 0.037529
H -1.401152 1.815823 2.165159
Se 1.607059 -0.728501 -0.806890
C 3.407011 -0.658943 -0.154915
C 4.435765 -0.049514 -0.898488
C 3.746283 -1.190008 1.104382
C 5.737645 0.031267 -0.405051
H 4.202873 0.369790 -1.878579
C 5.047392 -1.107965 1.600251
H 2.970225 -1.673352 1.700080
C 6.053120 -0.496924 0.848737
H 6.512059 0.510928 -1.005811
H 5.276866 -1.527784 2.580922
H 7.070826 -0.433559 1.234978
O -1.127899 -0.257242 -1.530979
C -1.476167 0.153636 -2.812658
H -1.782733 -0.717959 -3.416150
H -2.305382 0.876978 -2.812782
H -0.612798 0.608737 -3.325556
59
TS-VI-Gauche_b_conf_9 Eopt -
3396.606698
C 2.798788 -0.833193 1.968166
C 3.183874 0.193754 0.867355
C 1.807424 -1.844471 -0.864979

C 1.653541 -2.636432 0.454260
H 2.583577 -0.304709 2.905466
H 2.455165 -3.385221 0.537117
C 4.470113 -0.288118 0.153801
H 5.299325 -0.289246 0.881674
C 3.232807 -1.892240 -1.444018
H 3.286562 -1.148774 -2.253956
H 3.465629 1.127532 1.381444
H 1.166274 -2.335321 -1.613117
C 4.387477 -1.682903 -0.464824
H 5.331703 -1.885962 -0.992074
H 4.326716 -2.439036 0.331138
C 1.600291 -1.749438 1.696354
H 0.698597 -1.134251 1.600982
H 1.449718 -2.393452 2.576627
H 4.758568 0.416124 -0.634625
H 3.354652 -2.881960 -1.909680
H 3.676963 -1.460974 2.194097
H 0.710391 -3.198258 0.414806
B 2.026502 0.552223 -0.235456
C 0.647193 1.730797 0.767539
C -0.653452 1.702941 0.166371
C -1.872511 1.853129 1.028602
H -2.800175 1.777507 0.454457
H -1.906387 1.152427 1.867398
C -0.813052 2.256039 -1.220793
H -1.745219 1.911155 -1.678041
H 0.040202 1.991248 -1.854893
O 1.288408 -0.544102 -0.749879
C 1.318449 3.090745 0.507666
H 2.341741 3.092266 0.904276
H 0.762761 3.881715 1.043307
H -0.865007 3.356977 -1.146562
C 0.639600 1.506421 2.274196
H 0.192627 0.549901 2.568816
H 1.659722 1.545680 2.671467
H 0.072850 2.302953 2.787762
H -1.815595 2.874944 1.451823
H 1.358556 3.359892 -0.547658
Se -1.456552 -0.851786 -0.468130
C -3.348094 -0.635795 -0.250774
C -3.987672 -0.931472 0.967841
C -4.144512 -0.125085 -1.293577
C -5.354846 -0.717032 1.139490
H -3.395761 -1.331786 1.792222
C -5.511662 0.092024 -1.122715
H -3.676284 0.106899 -2.251375
C -6.126949 -0.201100 0.096091
H -5.821156 -0.954208 2.097153
H -6.100870 0.492564 -1.949262
H -7.195661 -0.031670 0.230690
O 2.444774 1.374745 -1.347827
C 3.487857 2.294499 -1.383774
H 3.980889 2.444270 -0.411556
H 4.258615 1.968661 -2.103081
H 3.127112 3.278186 -1.725158
59
TS-VII-Rotation_b_conf_1 Eopt -3396.629522
C 3.537510 -0.606109 1.915276
C 2.432997 0.356099 1.380538
C 4.113218 0.134501 -1.077182
C 5.099664 -0.677481 -0.201248
H 3.073444 -1.346385 2.583757
H 5.899539 -0.023024 0.176648
C 2.927836 1.808406 1.491084
H 3.137468 2.054751 2.547959
C 4.209324 1.648019 -0.774151
H 3.422868 2.171186 -1.331172
H 1.612993 0.270305 2.111574
H 4.463492 0.044175 -2.120966
C 4.203384 2.088460 0.693696
H 4.420994 3.167539 0.729247
H 5.050778 1.605727 1.204958
C 4.408902 -1.417237 0.942262
H 3.794966 -2.198344 0.484310
H 5.177060 -1.940183 1.534497
H 2.131923 2.484115 1.154306
H 5.173576 1.967105 -1.202476
H 4.213809 -0.027532 2.570005
H 5.597730 -1.432306 -0.827627
B 1.783990 0.066243 -0.138278
C 0.527158 -1.101271 -0.142242
C -0.889428 -0.592571 0.423032
C -1.462519 -1.476754 1.536663
H -2.457680 -1.122179 1.830047
H -1.546971 -2.528747 1.246614
C -0.936597 0.848103 0.941489
H -1.904043 1.039306 1.423276
H -0.766242 1.586089 0.157296
O 2.837470 -0.443226 -1.057390
C 1.016259 -2.294106 0.700796
H 1.988517 -2.609902 0.312900
H 0.350401 -3.167353 0.626711
H -0.157427 0.991026 1.699144
C 0.413981 -1.690414 -1.563668
H 1.401983 -2.050533 -1.869054
H -0.280313 -2.546695 -1.597430
H 0.094070 -0.965102 -2.320052
H -0.821542 -1.409923 2.427518
H 1.144454 -2.055462 1.762942
Se -2.276577 -0.684119 -1.087867
C -3.802031 0.085305 -0.209647
C -4.728956 -0.730455 0.451375
C -4.029650 1.466485 -0.268477
C -5.853213 -0.171913 1.061152
H -4.565499 -1.807771 0.488380
C -5.152995 2.023348 0.342188
H -3.317165 2.105563 -0.790498
C -6.067441 1.205484 1.007929
H -6.565533 -0.817906 1.575328
H -5.315335 3.100500 0.293759
H -6.946864 1.641462 1.482531
O 1.309754 1.384551 -0.651719
C 0.926042 1.513764 -1.972856
H 1.026615 2.568085 -2.286335
H 1.545402 0.902355 -2.654121
H -0.132277 1.230319 -2.140315
59
TS-VII-Rotation_b_conf_2 Eopt -3396.628317
C 3.477902 -0.642852 1.909976
C 2.427047 0.345089 1.349740
C 4.099344 -0.043256 -1.066405
C 5.070423 -0.781468 -0.131924
H 3.132817 -1.673284 1.772086
H 6.102486 -0.529479 -0.427073
C 2.933787 1.798029 1.391359
H 3.300863 2.040985 2.405878
C 4.168677 1.495383 -0.968714
H 3.418180 1.891714 -1.661450
H 1.610870 0.307449 2.090509
H 4.469496 -0.260624 -2.086989
C 4.047809 2.205309 0.394730
H 3.930800 3.278607 0.178420
H 5.021292 2.132923 0.895329
C 4.910329 -0.558582 1.373740
H 5.508700 -1.324193 1.892396
H 5.357060 0.398564 1.666311
H 2.064298 2.442857 1.215069
H 5.150825 1.779033 -1.381235
H 3.545757 -0.495487 3.002851
H 4.935772 -1.853189 -0.348147
B 1.769636 -0.010218 -0.142874
C 0.496512 -1.163110 -0.123387
C -0.910230 -0.635273 0.442106
C -1.496216 -1.508567 1.556778
H -0.856899 -1.446411 2.449030
H -2.488260 -1.142491 1.847039
C -0.928382 0.806779 0.954161

H -0.177950 0.921898 1.744847
H -1.905560 1.036797 1.397355
O 2.817965 -0.602457 -1.009091
C 0.979639 -2.360253 0.718906
H 1.079164 -2.132249 1.786451
H 1.968002 -2.658069 0.348531
H -0.695556 1.532941 0.174493
C 0.365942 -1.765624 -1.538557
H 1.345606 -2.146199 -1.845417
H -0.342277 -2.611024 -1.555382
H 0.050676 -1.046407 -2.301849
H -1.589996 -2.560655 1.268700
H 0.320124 -3.236476 0.620643
Se -2.300805 -0.704292 -1.068524
C -3.806575 0.110904 -0.197734
C -4.746147 -0.672041 0.484706
C -4.005966 1.495168 -0.282696
C -5.854205 -0.077867 1.090594
H -4.605693 -1.751733 0.541430
C -5.113558 2.087527 0.323208
H -3.282766 2.108645 -0.820523
C -6.040100 1.302366 1.011242
H -6.576386 -0.698237 1.622261
H -5.253738 3.166689 0.254279
H -6.906880 1.765973 1.482912
O 1.325763 1.296174 -0.716009
C 0.942064 1.384220 -2.040691
H 1.523703 0.710015 -2.695597
H -0.130838 1.151198 -2.190855
H 1.097074 2.415904 -2.404123
59
TS-VII-Rotation_b_conf_3 Eopt -
3396.623581
C -4.002099 0.113450 -1.516634
C -3.370941 0.852839 -0.325870
C -2.664750 -1.851799 0.697257
C -3.231814 -2.215310 -0.692525
H -3.362053 0.182088 -2.405769
H -3.616707 -3.244334 -0.607761
C -4.357667 0.830741 0.877467
H -5.382128 0.644842 0.507454
C -3.792933 -1.598564 1.728899
H -3.511442 -2.081570 2.676429
H -3.339363 1.905304 -0.649055
H -2.151286 -2.772294 1.024193
C -4.061012 -0.126285 2.043529
H -3.174718 0.259408 2.557670
H -4.900336 -0.075458 2.755972
C -4.358265 -1.347532 -1.250224
H -4.728914 -1.805577 -2.180316
H -5.205868 -1.380048 -0.548825
H -4.388588 1.837508 1.322068
H -4.712617 -2.105249 1.396565
H -4.937626 0.629688 -1.799611
H -2.394466 -2.258960 -1.400827
B -1.818532 0.519096 0.209826
C -0.553760 0.784317 -0.923134
C 0.917715 0.825409 -0.268313
C 1.749947 2.033871 -0.702211
H 1.890481 2.088170 -1.787438
H 1.255618 2.957451 -0.369384
C 0.983814 0.747285 1.256976
H 0.372122 -0.071161 1.644085
H 0.611347 1.679670 1.693404
O -1.670713 -0.870209 0.703286
C -0.829082 2.080898 -1.715933
H -0.824814 2.990087 -1.105144
H -1.822734 2.006070 -2.175484
H 2.024866 0.626557 1.581458
C -0.627294 -0.323175 -1.987428
H 0.122868 -0.180939 -2.781791
H -0.497523 -1.319138 -1.549921
H -1.602494 -0.318205 -2.478620
H 2.738403 2.009903 -0.227456
H -0.115028 2.229722 -2.540587
Se 1.983271 -0.827213 -0.902888
C 3.672399 -0.529632 -0.036866
C 4.675185 0.214463 -0.672303
C 3.942257 -1.108520 1.210283
C 5.915962 0.396421 -0.060989
H 4.478500 0.654462 -1.650248
C 5.184589 -0.928745 1.819078
H 3.168951 -1.695466 1.706387
C 6.173755 -0.175651 1.185409
H 6.685363 0.982589 -0.564549
H 5.379563 -1.381547 2.791747
H 7.144643 -0.036151 1.661547
O -1.656530 1.403871 1.417065
C -1.678429 2.777746 1.354872
H -0.704948 3.218067 1.049815
H -2.437161 3.182892 0.658566
H -1.907981 3.188733 2.353653
59
TS-VII-Rotation_b_conf_4 Eopt -
3396.619058
C 3.931037 -1.610055 0.478768
C 3.364076 -0.280628 0.997669
C 2.736303 0.368557 -1.827673
C 3.161819 -1.111553 -1.945685
H 3.258371 -2.442902 0.717313
H 3.527416 -1.232458 -2.978032
C 4.434110 0.832346 0.805022
H 5.433013 0.361945 0.764559
C 3.974952 1.293627 -1.767482
H 3.788193 2.173565 -2.400784
H 3.285799 -0.404610 2.088909
H 2.235757 0.581286 -2.787198
C 4.305076 1.816375 -0.370739
H 3.521201 2.532940 -0.124618
H 5.237322 2.400649 -0.431751
C 4.262839 -1.617947 -1.012464
H 4.542026 -2.637479 -1.320253
H 5.164621 -1.007189 -1.172101
H 4.447439 1.459652 1.709401
H 4.837543 0.780048 -2.217461
H 4.865487 -1.836445 1.024090
H 2.268029 -1.739622 -1.866816
B 1.838304 0.263815 0.569611
C 0.539354 -0.801661 0.933718
C -0.915524 -0.113316 0.830443
C -1.747117 -0.220218 2.109957
H -1.207778 0.271670 2.931035
H -2.702966 0.304639 1.990391
C -0.936767 1.354090 0.406656
H -1.954557 1.660424 0.136392
H -0.260478 1.540035 -0.431433
O 1.774149 0.669717 -0.853506
C 0.783985 -1.307273 2.368876
H 0.882403 -0.482918 3.086519
H 1.725595 -1.870451 2.388619
H -0.626729 1.971372 1.253694
C 0.585252 -2.059117 0.053109
H 0.426114 -1.829433 -1.007078
H 1.555525 -2.552142 0.126981
H -0.167470 -2.802094 0.362461
H -1.956976 -1.254565 2.403562
H 0.002598 -1.993940 2.727346
Se -2.025646 -1.076295 -0.626614
C -3.704031 -0.154058 -0.478737
C -4.687935 -0.607424 0.410076
C -3.988422 0.939194 -1.307492
C -5.923630 0.035410 0.485045
H -4.480194 -1.467998 1.046400
C -5.226110 1.579292 -1.234108
H -3.231471 1.289918 -2.009385
C -6.195946 1.129261 -0.337827
H -6.678145 -0.324635 1.185187
H -5.432800 2.430850 -1.883101
H -7.163547 1.628585 -0.281867
O 1.624698 1.430967 1.512144

C 1.647827 2.772081 1.200587
H 1.499949 2.969560 0.124361
H 0.846140 3.308667 1.742319
H 2.595378 3.260658 1.503728
59
TS-VII-Rotation_b_conf_5 Eopt -
3396.626924
C -3.911426 -0.177858 -1.562983
C -3.359741 0.791404 -0.477556
C -2.712058 -1.792195 0.881607
C -3.459136 -2.455406 -0.302601
H -3.694228 0.233448 -2.561269
H -4.492579 -2.693019 -0.008801
C -4.430377 0.964435 0.620224
H -5.341827 1.400717 0.172578
C -3.688075 -1.157999 1.895151
H -3.099970 -0.556546 2.599699
H -3.315519 1.778423 -0.963902
H -2.223534 -2.608969 1.441064
C -4.838932 -0.324510 1.330234
H -5.527334 -0.076170 2.153202
H -5.425879 -0.947142 0.637849
C -3.426408 -1.636865 -1.594304
H -2.391704 -1.649386 -1.946167
H -4.015223 -2.172710 -2.356306
H -4.082810 1.682528 1.372390
H -4.128142 -1.995451 2.460163
H -5.014539 -0.184032 -1.502575
H -2.981476 -3.421345 -0.524192
B -1.840298 0.474148 0.190264
C -0.520848 0.960889 -0.799484
C 0.912020 0.828125 -0.078194
C 1.742363 2.115140 -0.092117
H 2.704027 1.952490 0.409495
H 1.940082 2.489358 -1.102188
C 0.887139 0.332685 1.368135
H 1.909850 0.153331 1.722849
H 0.285072 -0.572583 1.472237
O -1.669829 -0.972739 0.434422
C -0.701423 2.400366 -1.333823
H 0.048774 2.642228 -2.103089
H -0.650379 3.182228 -0.571002
H 0.440113 1.102590 2.006231
C -0.595086 0.107149 -2.076377
H -1.567901 0.259267 -2.557204
H 0.168425 0.400177 -2.814369
H -0.493528 -0.962912 -1.867194
H 1.216882 2.897332 0.472714
H -1.678871 2.486137 -1.822269
Se 2.041176 -0.560267 -1.099882
C 3.688907 -0.486908 -0.115147
C 4.704080 0.400350 -0.496269
C 3.916261 -1.361978 0.955139
C 5.915903 0.426952 0.194378
H 4.539178 1.074500 -1.337167
C 5.129210 -1.335495 1.644035
H 3.133449 -2.061303 1.249803
C 6.131399 -0.441452 1.265247
H 6.695763 1.125858 -0.109742
H 5.291441 -2.019312 2.477761
H 7.079749 -0.422529 1.802835
O -1.798631 1.141642 1.534576
C -1.788036 2.514567 1.653760
H -2.181269 2.806874 2.643261
H -0.767340 2.943584 1.578506
H -2.403983 3.031099 0.893151
59
TS-VII-Rotation_b_conf_6 Eopt -
3396.622742
C 3.919723 -1.633602 0.134043
C 3.326259 -0.460828 0.933157
C 2.724617 0.808091 -1.658420
C 3.173963 -0.572362 -2.179448
H 3.299846 -2.526625 0.280505
H 3.621427 -0.415743 -3.174213
C 4.372747 0.661111 1.085316
H 5.259471 0.256075 1.606947
C 3.849910 1.771584 -1.235737
H 3.341364 2.679545 -0.880141
H 3.215568 -0.840262 1.962341
H 2.278731 1.293213 -2.545823
C 4.889379 1.329089 -0.196870
H 5.467643 2.221882 0.090095
H 5.618223 0.666836 -0.679150
C 4.165222 -1.428298 -1.375615
H 4.201975 -2.418090 -1.857054
H 5.169365 -1.013541 -1.521207
H 3.956956 1.422044 1.754714
H 4.393454 2.064978 -2.148523
H 4.895815 -1.904125 0.578352
H 2.247327 -1.135867 -2.352194
B 1.797784 0.161240 0.611658
C 0.514070 -0.949603 0.822625
C -0.949029 -0.272254 0.842820
C -1.785942 -0.632189 2.072408
H -1.978153 -1.706798 2.163459
H -1.263247 -0.294855 2.978162
C -0.987283 1.248225 0.705097
H -0.362625 1.587417 -0.124916
H -0.615626 1.699881 1.629899
O 1.676097 0.734903 -0.738573
C 0.763415 -1.701922 2.143054
H 0.866947 -1.018994 2.997165
H 1.702535 -2.262973 2.059927
H -2.018273 1.591956 0.557047
C 0.594281 -2.014785 -0.282117
H 1.575896 -2.491751 -0.288264
H -0.145094 -2.819398 -0.138855
H 0.450986 -1.580101 -1.278084
H -2.751048 -0.111585 2.043083
H -0.017734 -2.439140 2.379766
Se -2.032956 -0.957934 -0.776050
C -3.710819 -0.063945 -0.498572
C -4.714859 -0.659851 0.275899
C -3.971533 1.163450 -1.121990
C -5.947610 -0.028433 0.443034
H -4.525623 -1.622604 0.751172
C -5.205859 1.792476 -0.956701
H -3.197761 1.628168 -1.733513
C -6.196353 1.198288 -0.173923
H -6.718041 -0.500948 1.053185
H -5.393667 2.749518 -1.444504
H -7.161175 1.689759 -0.046048
O 1.617782 1.219352 1.678435
C 1.706040 2.570122 1.407414
H 1.117099 3.138197 2.150001
H 2.741472 2.962723 1.464770
H 1.324490 2.834543 0.407557
59
TS-VII-Rotation_b_conf_7 Eopt
3396.626091
C 3.247583 0.575556 -1.915446
C 3.255152 -0.553409 -0.846687
C 2.680315 1.753654 0.955275
C 2.853848 2.610775 -0.325665
H 2.836641 0.186640 -2.858121
H 3.880710 3.000480 -0.393006
C 4.630002 -0.607369 -0.155031
H 5.408347 -0.843515 -0.903482
C 4.020538 1.249841 1.531799
H 3.792117 0.505242 2.307580
H 3.198945 -1.505782 -1.398656
H 2.280440 2.426807 1.734529
C 5.045290 0.681328 0.550723
H 5.987411 0.507111 1.093771
H 5.280447 1.449289 -0.202212
C 2.470164 1.860767 -1.600666
H 1.403146 1.617940 -1.518902
H 2.572439 2.547999 -2.455509
H 4.624019 -1.428955 0.571907

H	4.482549	2.112702	2.037782
H	4.288705	0.855865	-2.156531
H	2.199161	3.491564	-0.250995
B	1.987616	-0.565811	0.250001
C	0.561874	-1.289670	-0.436145
C	-0.873202	-0.908379	0.194939
C	-1.654608	-2.118880	0.715441
H	-2.648387	-1.811028	1.061819
H	-1.780985	-2.902881	-0.037780
C	-0.888738	0.138542	1.309697
H	-1.904386	0.232255	1.715378
H	-0.535124	1.113209	0.971841
O	1.696575	0.782170	0.766758
C	0.807238	-2.809687	-0.297625
H	1.816648	-3.033490	-0.664171
H	0.103053	-3.406190	-0.898706
H	-0.230756	-0.181159	2.122194
C	0.552072	-1.080975	-1.965599
H	-0.276072	-1.630215	-2.441170
H	0.480208	-0.030496	-2.268964
H	1.470070	-1.486094	-2.402201
H	-1.132453	-2.548924	1.581995
H	0.769220	-3.164865	0.736982
Se	-2.065041	-0.094593	-1.280819
C	-3.621506	0.384068	-0.261322
C	-3.741963	1.667511	0.287687
C	-4.676515	-0.524278	-0.107135
C	-4.888482	2.027802	0.995372
H	-2.927918	2.381951	0.163675
C	-5.822318	-0.163154	0.603061
H	-4.596732	-1.519085	-0.546206
C	-5.930937	1.113392	1.154984
H	-4.967708	3.028549	1.421133
H	-6.634522	-0.881428	0.719100
H	-6.827544	1.396609	1.706916
O	2.488812	-1.443824	1.361058
C	1.861596	-1.460687	2.587343
H	0.871037	-1.961148	2.567680
H	2.483407	-2.015621	3.310296
H	1.699670	-0.445386	2.997348

3.6 References

1. Frisch, M. J. *et al.* Gaussian 16, Revision C.01. (2016).
2. Neese, F. The ORCA program system. *WIREs* **2**, 73–78 (2012).
3. Zhao, Y. & Truhlar, D. G. The M06 suite of density functionals for main group thermochemistry, thermochemical kinetics, noncovalent interactions, excited states, and transition elements: Two new functionals and systematic testing of four M06-class functionals and 12 other functions. *Theor. Chem. Acc.* **120**, 215 (2008).
4. Grimme, S., Antony, J., Ehrlich, S. & Krieg, H. A consistent and accurate ab initio parametrization of density functional dispersion correction (DFT-D) for the 94 elements H-Pu. *J. Chem. Phys.* **132**, 154104 (2010).
5. Chen, M., Knox, C. D., Madhusudhanan, M. C., Tugwell, T. H., Liu, C., Liu, P. & Dong, G. Stereospecific alkenylidene homologation of organoboronates by S_NV reaction. *Nature* **631**, 328–334 (2024).
6. Brémond, E. & Adamo, C. Seeking for parameter-free double-hybrid functionals: The PBE0-DH model. *J. Chem. Phys.* **135**, 024106 (2011).
7. Grimme, S., Ehrlich, S. & Goerigk, L. Effect of the damping function in dispersion corrected density functional theory. *J. Comp. Chem.* **32**, 1456–1565 (2011).
8. Neese, F. An improvement of the resolution of the identity approximation for the formation of the Coulomb matrix. *J. Comp. Chem.* **24**, 1740–1747 (2003).
9. Weigend, F. & Ahlrichs, R. Balanced basis sets of split valence, triple zeta valence and quadruple zeta valence quality for H to Rn: Design and assessment of accuracy. *Phys. Chem. Chem. Phys.*, **7**, 3297 (2005).
10. Rappoport, D. & Furche, F. Property-optimized Gaussian basis sets for molecular response calculations, *J. Chem. Phys.*, **133**, 134105 (2010).
11. Marenich, A. V., Cramer, C. J. & Truhlar, D. G. Universal solvation model based on solute electron density and on a continuum model of the solvent defined by the bulk dielectric constant and atomic surface tensions. *J. Phys. Chem. B* **113**, 6378–6396 (2009).
12. Glendening, E. D., Landis, C. R. & Weinhold, F. *NBO* 7.0: New vistas in localized and delocalized chemical bonding theory. *J. Comp. Chem.* **40**, 2234–2241 (2019).
13. The PyMOL Molecular Graphics System, Version 2.0.7.
14. Pracht, P., Bohle, F. & Grimme, S. Automated exploration of the low-energy chemical space with fast quantum chemical methods. *Phys. Chem. Chem. Phys.* **22**, 7169–7192 (2020).
15. Grimme, S. Supramolecular binding thermodynamics by dispersion-corrected density functional theory. *Chem. - A Eur. J.* **18**, 9955–9964 (2012).
16. Luchini, G., Alegre-Requena, J. V., Funes-Ardoiz, I. & Paton, R. S. GoodVibes: automated thermochemistry for heterogeneous computational chemistry data. *F1000Research* **9**, 291 (2020).
17. Bryantsev, V. S., Diallo, M. S. & Goddard III, W. A. Calculation of solvation free energies of charged solutes using mixed cluster/continuum models. *J. Phys. Chem. B* **112**, 9709–9719 (2008).
18. Fukui, K. The Path of Chemical Reactions - the IRC Approach. *Acc. Chem. Res.* **14**, 363−368 (1981).
19. Alegre-Requena, J. V., S. V., S. S., Pérez-Soto, R., Alturaifi, T. M. & Paton, R. S. AQME: Automated quantum mechanical environments for researchers and educators. *WIREs* **13**, e1663 (2023).
